# On the extent and role of the small proteome in the parasitic eukaryote *Trypanosoma brucei*

**DOI:** 10.1186/1741-7007-12-14

**Published:** 2014-02-19

**Authors:** Megan Ericson, Michael A Janes, Falk Butter, Matthias Mann, Elisabetta Ullu, Christian Tschudi

**Affiliations:** 1 Department of Epidemiology of Microbial Diseases, School of Public Health, Yale University, New Haven, CT, USA; 2 Department of Proteomics and Signal Transduction, Max Planck Institute of Biochemistry, Martinsried, Germany; 3 Department of Cell Biology and Internal Medicine, School of Medicine, Yale University, New Haven, CT, USA; 4 Current address: San Francisco General Hospital, Pulmonary & Critical Care, San Francisco, CA, USA; 5 Current address: Institute of Molecular Biology gGmbH, Mainz, Germany

**Keywords:** Genomics, Proteomics, Mass spectrometry data, Non-coding RNA, Mitochondria

## Abstract

**Background:**

Although technical advances in genomics and proteomics research have yielded a better understanding of the coding capacity of a genome, one major challenge remaining is the identification of all expressed proteins, especially those less than 100 amino acids in length. Such information can be particularly relevant to human pathogens, such as *Trypanosoma brucei*, the causative agent of African trypanosomiasis, since it will provide further insight into the parasite biology and life cycle.

**Results:**

Starting with 993 *T. brucei* transcripts, previously shown by RNA-Sequencing not to coincide with annotated coding sequences (CDS), homology searches revealed that 173 predicted short open reading frames in these transcripts are conserved across kinetoplastids with 13 also conserved in representative eukaryotes. Mining mass spectrometry data sets revealed 42 transcripts encoding at least one matching peptide. RNAi-induced down-regulation of these 42 transcripts revealed seven to be essential in insect-form trypanosomes with two also required for the bloodstream life cycle stage. To validate the specificity of the RNAi results, each lethal phenotype was rescued by co-expressing an RNAi-resistant construct of each corresponding CDS. These previously non-annotated essential small proteins localized to a variety of cell compartments, including the cell surface, mitochondria, nucleus and cytoplasm, inferring the diverse biological roles they are likely to play in *T. brucei*. We also provide evidence that one of these small proteins is required for replicating the kinetoplast (mitochondrial) DNA.

**Conclusions:**

Our studies highlight the presence and significance of small proteins in a protist and expose potential new targets to block the survival of trypanosomes in the insect vector and/or the mammalian host.

## Background

Recent advances in high-throughput sequencing technologies have led to the discovery of a large number of transcripts originating from regions of the genome previously thought to be silent [1]. One major challenge arising from these observations is to determine whether these transcripts code for a protein or should be classified as non-coding RNAs. This task is rather overwhelming, since a majority of these transcripts only have the potential to encode small proteins, generally less than 100 amino acids (aa) [2,3]. Historically, an arbitrary cutoff for open reading frames of 100 aa was applied in genome annotation projects [4,5] and thus the extent and functional significance of small open reading frames (sORFs) remains a largely unexplored territory in many organisms. Nevertheless, copious reports clearly indicate that they play crucial biological roles, including protection against pathogens [6,7], signal transduction [8], serving as molecular chaperones [9], developmental regulation [10-13] and even calcium transport in cardiac muscle contraction [14].

Several proteins encoded by sORFs have been identified serendipitously by biochemical methods as part of a complex or the product of a processed precursor protein. One example is the *Drosophila tarsal-less* (*tal*) gene, originally annotated as non-coding*,* but later shown to encode three small proteins with a crucial role in fly development [13]. Several studies have used genome-wide approaches to gauge the prevalence of sORFs. When examining potential small proteins in *Drosophila melanogaster*, Ladoukakis *et al*. identified 4,561 sORFs that were conserved in a closely related species, *Drosophila pseudoobscura*[15]. Synteny, evidence of transcription and nucleotide substitution, narrowed the 4,561 to a more conservative estimate of 401 sORFs. A study on the *Arabidopsis* small proteome assessed evolutionary conservation and examined evidence of transcription to predict the expression of as many as 3,241 sORFs [16]. A report on the mammalian small proteome by Frith *et al*. used FANTOM cDNA data to identify a potential 1,240 sORFs using a CRITICA gene-detection program [17]. Additionally, 25 sORFs were GFP-tagged and, following transfection into cells, 14 of the fusion proteins were detected, providing evidence of translation [17]. More recently, using a novel combination of peptidomics and RNA-Sequencing (RNA-Seq), Slavoff *et al*. identified 86 novel small proteins in humans and two were tagged and shown to localize to the mitochondria and cytoplasm [18]. Nevertheless, to date few functional studies of proteins encoded by sORFs have been performed. In yeast, 140 small proteins were tested by generating gene deletions and 22 had an effect on *Saccharomyces cerevisiae* growth under various conditions [19], whereas overexpression of 473 small proteins in *Arabidopsis* resulted in 49 recognizable phenotypes [20].

Mass spectrometry, a powerful technique in proteomics to validate the existence of putative protein candidates, has been applied in several studies [18,21-25]. High-resolution mass spectrometry provides very accurate precursor ion masses and combined with stringent statistical methods enhances the certainty of peptide identification [26]. This is a key issue in the validation of newly identified sORFs. In general, a protein database derived from the genome is used in shotgun proteomics to identify peptides and proteins from mass spectrometric raw data, but six frame translation of the genome is also frequently employed [24,25]. In either case, the certainty of the existence of any protein can be increased by an observed corresponding RNA transcript. Recently, we used a combination of stringent methods, that is, ribosome footprinting, next generation sequencing and advanced mass spectrometric technology, to discover a plethora of novel sORFs in cytomegalovirus, many of which we determined to exist at the protein level [23].

The question of whether functional small proteins exist is particularly relevant in organisms with a tightly organized genome, such as the parasitic protozoan *Trypanosoma brucei*. Protein-coding genes are arranged in long unidirectional clusters with intergenic regions only a few hundred nucleotides in length, thus leaving little space for sORFs or non-coding RNAs. The initial sequencing and annotation of the 11 megabase-sized chromosomes, published in 2005, predicted 9,068 protein-coding genes [27]. As of November 2013, this number has increased to 10,574 (TriTrypDB); however, a major challenge remains to identify all expressed proteins. This quest was addressed by several RNA-Seq studies using Illumina high-throughput cDNA sequencing [28-31]. In particular, we provided evidence that the coding potential of the *T. brucei* genome was larger than originally anticipated by identifying 1,114 transcripts mapping to regions of the genome with no annotated ORFs [28]. A total of 993 of these transcripts have the potential to contain a coding sequence (CDS) of at least 25 amino acids and the remaining 121 transcripts either have no coding potential at all or no ORF larger than 75 nucleotides. However, it remains to be established whether these transcripts encode functional proteins.

Founded on the set of transcripts identified by our transcriptome analysis [28], we applied bioinformatics approaches to identify small proteins conserved across kinetoplastid species and representative eukaryotes. Combined with mass spectrometry data, we pinpointed 42 high-confidence small proteins ranging in size from 49 to 219 amino acids. RNAi-knockdown revealed seven essential proteins in the insect-stage of the life cycle and their diverse subcellular localizations suggested involvement in many aspects of *T. brucei* biology.

## Results

### *T. brucei* transcripts encoding evolutionarily conserved potential small proteins

**Figure 1:**
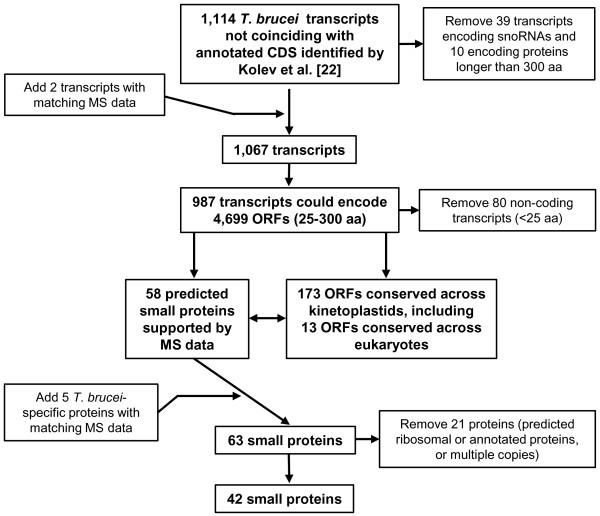
**Flowchart of the strategy used to analyze *T. brucei *** **transcripts not coinciding with annotated coding sequences (CDS).**

We previously published a single-nucleotide resolution genomic map of the *T. brucei* transcriptome, which included 1,114 transcripts not originating from annotated CDS ( [28]; original RNA-Seq data have been submitted to the National Center for Biotechnology Information (NCBI) Sequence Read Archive - SRA at [32] - under accession no. SRA012290 and the 1,114 transcripts are accessible through a community file, Tbrucei_novel_transcripts.fasta, on TriTrypDB at [33]). After a reexamination of this data set using the latest *T. brucei* genome annotation (GeneDB version 5, [34]), we excluded 39 and 10 transcripts coding for snoRNAs and annotated proteins larger than 300 amino acids, respectively, and added two novel transcripts coding for proteins identified by mass spectrometry (MS) data (Figure 1). Setting a lower limit of 25 aa, 987 of the remaining transcripts contain between one (112 transcripts) and 31 (1 transcript) ORFs for a total of 4,699 ORFs [see Additional file 1]. Eighty transcripts were classified as non-coding RNAs, since the predicted ORFs were less than 75 nucleotides. However, we cannot exclude the possibility that the latter category has coding potential by using alternative initiation codons or encoding proteins smaller than 25 aa.

The selected 4,699 ORFs were highly enriched in short ORFs (sORFs), that is, less than 100 amino acids, with 4,499 ORFs (96%) falling into this category [see Additional file 2: Figure S1]. Since proteins encoded by sORFs largely escape standard genome annotations, we examined evolutionary conservation in combination with computational approaches to screen for ORFs conserved in kinetoplastidae and representative eukaryotes as a benchmark for protein expression. Kinetoplastid protists belong to the phylum *Euglenozoa* and include a significant number of disease-causing parasites, such as *T. brucei* and *T. cruzi*, the causative agent of African trypanosomiasis and Chagas disease, respectively, and the Old and New World *Leishmania* parasites, which cause various forms of leishmaniasis worldwide. First, we conducted Basic Local Alignment Search Tool (BLAST) analyses [35] of kinetoplastid genomes and annotated proteins, excluding the *T. brucei* subspecies (see Methods for details). Of the 987 transcripts, 157 encoded one ORF that was conserved in at least one kinetoplastid organism and four transcripts (*Tb4.NT.51, Tb5.NT.84, Tb6.NT.58* and *Tb8.NT.142*) encoded between two and twelve conserved ORFs for a total of 173 conserved ORFs [see Additional file 3]. Second, we compared the selected 4,699 ORFs to the annotated proteins from representative eukaryotes, namely *S. cerevisiae*, *Caenorhabditis elegans*, *Arabidopsis thaliana*, *D. melanogaster*, *Mus musculus* and *Homo sapiens*. We found that 13 ORFs had significant alignments with BLAST bit scores ranging from 34 to 227, with 6 coding for ribosomal proteins [see Additional file 4]. It is worth noting that these 13 ORFs were part of the set conserved in kinetoplastids. We next surveyed the 173 conserved ORFs for known protein domain(s) using the CD-Search Tool (cdsearch/cdd v3.10 [36]) and detected domains in 61 ORFs covering a broad spectrum [see Additional file 5]. However, the ribosomal protein superfamily (six hits), various Zn finger domains (five hits) and the RNA recognition motif (RRM) superfamily (three hits) were overrepresented. Finally, our analysis of SignalP [37] and TMHMM [38] predictions revealed that 5 of the 173 potential small proteins have a predicted signal peptide and that a considerable number (43 or 25%) have a predicted trans-membrane domain with seven having more than one predicted domain.

### Identification of small protein candidates through mass spectrometry data

For an alternative approach based on peptide evidence to recognize transcripts coding for small proteins, we surveyed MS data ( [21,22] and this study) for peptides matching the 4,699 selected ORFs described above. As reported previously [28], searching the proteome data of Panigrahi *et al*. [21] provided evidence for the expression of 16 small proteins, with all 16 being part of the 173 small protein candidates identified bioinformatically. In addition, MS data from Butter *et al*. [22] and this study revealed 63 hits. As well as providing validation for 58 of the 173 small protein candidates, our data also predicted five small proteins specific to *T. brucei* with no recognizable homologues in other kinetoplastids. We also performed a search against hexatranslations of the trypanosome genome, which revealed the same set of newly identified proteins (data not shown). Taken together, we were able to provide supporting MS data for 63 predicted small proteins with 22 being represented in more than one MS data set, and, except for the *T. brucei*-specific hits, all the other matches were among the evolutionarily conserved 173 small protein candidates [see Additional file 3].

The 63 small protein candidates with supporting MS data were filtered further by removing predicted ribosomal proteins or annotated proteins with a predicted function and CDS with multiple copies in the genome, leaving us with 42 small proteins for further analysis [see Additional file 3]. This final group of selected proteins ranges in size from 49 to 219 amino acids and 35 qualify as small proteins. Transcript lengths vary from 333 to 4,100 nucleotides and the average 5’ UTR length is 119 nucleotides with a median of 110 nucleotides. This is similar to the global analysis of the transcriptome [28,30], where a median length of 128 to 130 nucleotides was reported. On the other hand, the 3’ UTR is on average 390 nucleotides long with a median of 237 nucleotides, with the latter being notably smaller than the medians reported in the aforementioned transcriptome studies, namely 400 nucleotides [30] and 388 nucleotides [28].

Noteworthy characteristics of this collection of 42 small proteins are as follows: three have putative homologues in representative eukaryotes (*Tb*7.NT.49, *Tb*11.NT.47 and *Tb*11.NT.220); predicted domains include two RRMs and two Zn-finger domains; sixteen have a predicted trans-membrane domain; and one has a predicted signal peptide [see Additional file 3].

### RNAi screen of the 42 small proteins revealed 7 to be essential in the insect life-cycle stage

RNAi knock-down strategies have revolutionized the functional analysis of genes in *T. brucei*[39]. mRNA degradation is triggered most efficiently by double-stranded RNA (dsRNA) produced *in vivo* as a hairpin RNA transcribed from a tetracycline-inducible promoter. Thus, we generated a hairpin construct for each of the 42 ORFs using the pTrypRNAiGate plasmid [40]. Each construct was stably integrated in the non-transcribed rRNA spacer region of a special procyclic-form recipient strain, named 29.13.6, expressing the tet repressor and T7 RNA polymerase [41], and clonal cell lines were established. Upon RNAi induction with tetracycline, 12 had a growth phenotype that differed from un-induced control cells. Three of the knockdowns resulted in a slow-growth phenotype. For example, RNAi of *Tb*5.NT.58 resulted in a cell division time of 16 hours as compared to 8.5 hours for un-induced cells and this phenotype was not accompanied by noticeable changes in cell morphology [see Additional file 2: Figure S2]. In addition, knockdown of two small proteins (*Tb*11.NT.222 and *Tb*11.NT.66) resulted in faster growth with no obvious changes in cell morphology [see Additional file 2: Figure S2]. Monitoring cell growth after RNAi induction of the remaining seven revealed that all cell lines stopped dividing and eventually died demonstrating that *Tb3.NT.18, Tb10.NT.86, Tb10.NT.87*, *Tb10.NT.90, Tb11.NT.28, Tb11.NT.29* and *Tb11.NT.108* are essential genes (Table 1; Additional file 2: Figure S3). For subsequent analyses we focused on the seven essential predicted small proteins and the RNAi knockdowns revealing a change in the doubling time were not pursued further.

**Table 1:** Characteristics of the seven essential proteins in procyclics

**ORF ID**	**Transcript**	**AA/MW**	**pI**	**Predicted**	**Predicted**	**Localization**
	**size (nt)**			**signal peptide**	**TM domain**	
Tb10.NT.87	478	64/8.2	11.46	No	No	Mitochondria
Tb11.NT.28	1,285	56/6.3	9.3	No	Yes	Mitochondria
Tb11.NT.29	796	62/7.6	6.96	No	Yes	Surface
Tb3.NT.18	709	85/9.3	7.91	No	Yes	Cytoplasm
Tb10.NT.86	605	93/10.5	9.67	No	Yes	Cytoplasm
Tb11.NT.108	461	96/10.5	6.49	No	No	Cytoplasm
Tb10.NT.90	529	67/7.3	4.49	No	Yes	Nucleus

AA, number of amino acids; MW, molecular weight; ORF, open reading frame; pI, isoelectric point; TM, transmembrane.

To confirm that the observed essentiality of the seven small proteins was specific to RNAi knockdown of the predicted transcript, we performed the following experiments. First, we verified the transcript length expected by the RNA-Seq data [28] using Northern blot analysis. In six of the seven cases a single predominant hybridizing band was detected and the observed size matched the predicted size within the limits of resolution of Northern blotting [see Additional file 2: Figure S4]. The seventh transcript, *Tb3.NT.18*, had two bands detected by Northern blot. One band corresponded to the size of the predicted novel transcript of 709 nucleotides. Further interrogation by Northern blotting and RT-PCR with probes specific for the upstream (*Tb*927.3.1080) and downstream annotated gene (*Tb*927.3.1090) led us to conclude that the longer RNA contained both *Tb3.NT.18* and the downstream transcript encoding a component of the *T. brucei* U1 small nuclear ribonucleoprotein (snRNP). This finding was reminiscent of the presence of an upstream open reading frame (uORF) described in organisms from fungi to humans [42,43]. uORFs are defined as predominantly short ORFs found in the 5’ UTR of a previously annotated gene and experiments are ongoing to investigate whether *Tb*3.NT.18 qualifies as an uORF.

Second, semi-quantitative RT-PCR verified that the knockdown of the seven essential transcripts was efficient [see Additional file 2: Figure S5]. Third, to confirm the specificity of the RNAi knockdown, we set out to rescue each lethal phenotype with the expression of an RNAi-resistant construct. To do this, the CDS targeted by RNAi was assembled as a synthetic sequence bearing at least one silent mutation per 12 contiguous base-pairs [44] and flanked by heterologous UTR sequences (see Methods). In addition, an HA-TEV-FLAG epitope tag or a GFP tag was added to the C-terminus [see Additional file 2: Figure S6]. Upon co-expression of the hairpin targeting the endogenous transcript and the corresponding modified CDS in a stable cell line, the endogenous transcript was destroyed, as shown by RT-PCR, and in all seven cases the cells survived on the RNAi-resistant transcript encoding the same small protein [see Additional file 2: Figure S3]. These results led us to conclude that the essential phenotype was a direct consequence of the knockdown of the targeted CDS.

### Initial characterization of the essential small proteins

**Figure 2:**
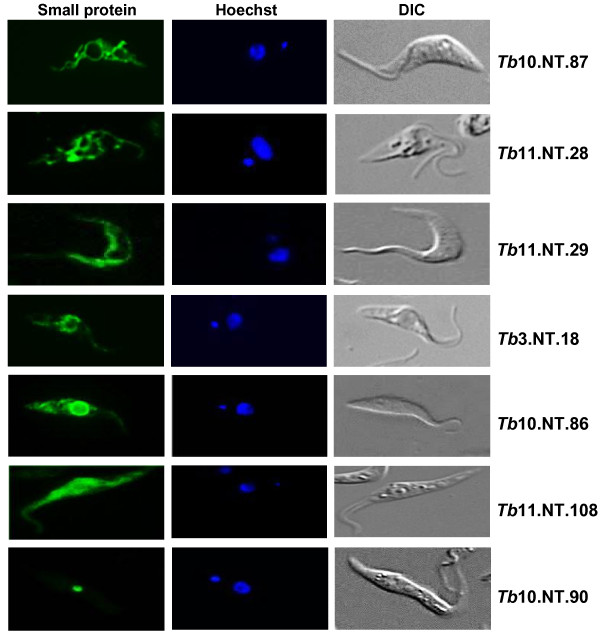
**Localization of the seven small proteins essential in the procyclic life cycle stage.** Anti-HA or anti-GFP antibodies were used to detect C-terminal HA- (*Tb11.NT.28* and *Tb11.NT.29*) or GFP- (*Tb10.NT.87, Tb10.NT.90, Tb10.NT.86, Tb3.NT.18,* and *Tb11.NT.108*) tagged versions of the proteins by fluorescence microscopy. DNA was stained with Hoechst (blue) and DIC images are shown in the right panels. DIC, differential interference contrast.

The RNAi rescue experiments described above established that the epitope-tagged small proteins were functional and, thus, could be used for biochemical and cell biological experiments. Using fluorescence microscopy we detected expression of all seven small proteins (Figure 2) and Western blot analysis confirmed that the proteins had the predicted relative molecular mass [see Additional file 2: Figure S7]. *Tb*11.NT.28 and *Tb*10.NT.87 revealed a fluorescence pattern typical of the procyclic trypanosome branched tubular mitochondrion (Figure 2). *Tb*11.NT.29 appeared to be a surface protein, an observation supported by subsequent experiments (see below). By immunofluorescence, three proteins (*Tb*3.NT.18, *Tb*10.NT.86 and *Tb*11.NT.108) were shown to be enriched in the cytoplasm, with *Tb*11.NT.108 distributed throughout this compartment, whereas *Tb*3.NT.18 and *Tb*10.NT.86 appeared somewhat concentrated around the nucleus. Finally, *Tb*10.NT.90 had a distinct localization in the nucleus, possibly indicative of the nucleolus.

**Figure 3:**
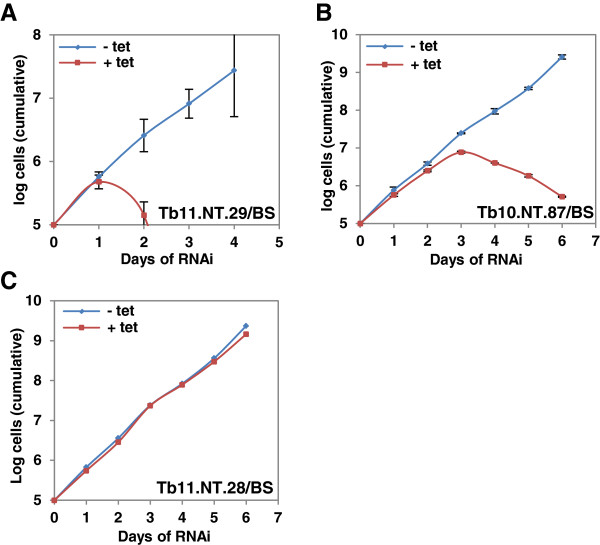
**Bloodstream-form growth following RNAi. (A)***Tb11.NT.29* RNAi in bloodstream-form cells (BS). **(B)***Tb10.NT.87* RNAi in BS cells. **(C)***Tb11.NT.28* RNAi in BS cells. Growth of un-induced (-tet) and induced cells (+ tet) shown in log scale. The data are based on three independent experiments, and average values with standard deviations are presented.

Since *T. brucei* undergoes extensive morphological and metabolic changes during its life cycle alternating between the mammalian (bloodstream) and insect (procyclic) hosts, it was of interest to gauge the essentiality of the seven small proteins in bloodstream forms. Thus, the hairpin constructs were transfected into a bloodstream form cell line competent for RNAi and, following induction, *Tb*11.NT.29, a potential surface protein, and *Tb*10.NT.87, a probable mitochondrial protein, were shown to be essential in this stage of the life cycle (Figure 3A and B). Growth curves for the five nonessential proteins can be found in Figure 3C and Additional file 2: Figure S8. Based on the above results, we selected *Tb*11.NT.29, *Tb*11.NT.28 and *Tb*10.NT.87 for further analysis.

### *Tb*11.NT.29: a putative surface protein

**Figure 4:**
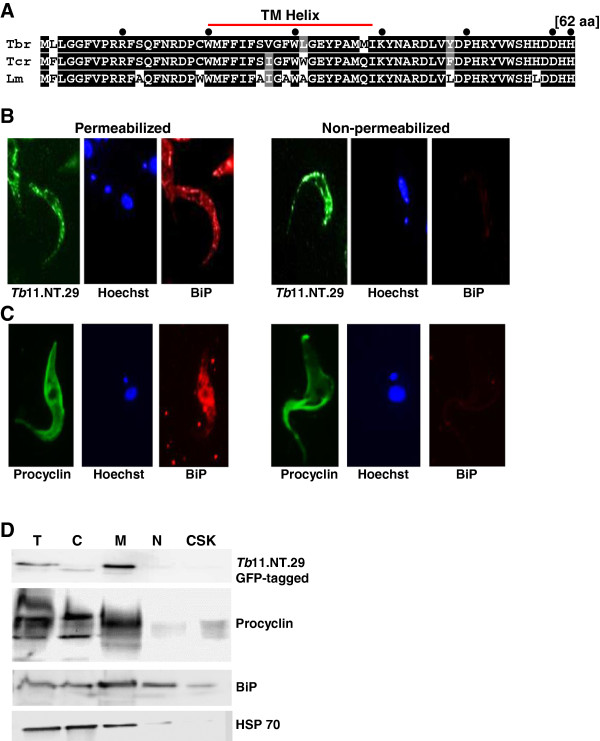
**Characterization of *Tb*11.NT.29*. *(A)** Sequence conservation of *Tb*11.NT.29 across kinetoplastids. Amino acid sequences from *T. brucei* (Tbr), *T. cruzi* (Tcr) and *L. major* (Lm) were aligned with ClustalW and conserved residues are shaded with black boxes while similar residues are shaded in grey. The predicted transmembrane domain (TM helix) is indicated. **(B)** Immunofluorescence analysis on cells expressing a C-terminal HA-tagged version of *Tb*11.NT.29. Permeabilized cells (left three panels) and non-permeabilized cells (right three panels) were probed for HA (green) and BiP (red) and DNA was stained with Hoechst (blue). **(C)** Immunofluorescence analysis of procyclin as described in panel (B). **(D)** Cell fractionation of parasites expressing a GFP-tagged version of *Tb*11.NT.29. Western blot analysis was performed against GFP-tagged *Tb*11.NT.29 (top panel), procyclin (second from top), BiP (third panel from top) and HSP 70 (bottom panel) on total (T), cytoplasmic (C), membrane (M), nuclear (N) and cytoskeleton (CSK) fractions.

The 796 nucleotide-long *Tb11.NT.29* transcript encodes a 62 aa protein, which is highly conserved in kinetoplastids (84% identity between *T. brucei* and *L. major*) and has a predicted trans-membrane domain (Figure 4A). Initial immunofluorescence suggested that *Tb*11.NT.29 might be localized on the surface (Figure 2). To address this possibility, we compared the signal for cells expressing epitope-tagged *Tb*11.NT.29 that were either permeabilized by detergent prior to antibody exposure or remained non-permeabilized. Under permeabilized conditions we detected both *Tb*11.NT.29 and the endoplasmic reticulum (ER) protein BiP (Figure 4B). However, when we omitted the permeabilization step, thus limiting access of antibodies to potential surface molecules, no signal was detected for BiP, whereas the signal for *Tb*11.NT.29 was still visible (Figure 4B). This behavior was similar to procyclin (Figure 4C), a well-characterized *T. brucei* surface protein specific for the procyclic life-cycle stage [45]. Since the epitope tag was at the C-terminus of the *Tb*11.NT.29, this result also suggested that this portion of the protein was exposed on the surface. To corroborate this localization, we performed cell fractionation experiments and by Western blot analysis *Tb*11.NT.29 was enriched in the membrane fraction, similar to procyclin, whereas HSP70 was, as expected, enriched in the cytoplasmic fraction (Figure 4D).

**Figure 5:**
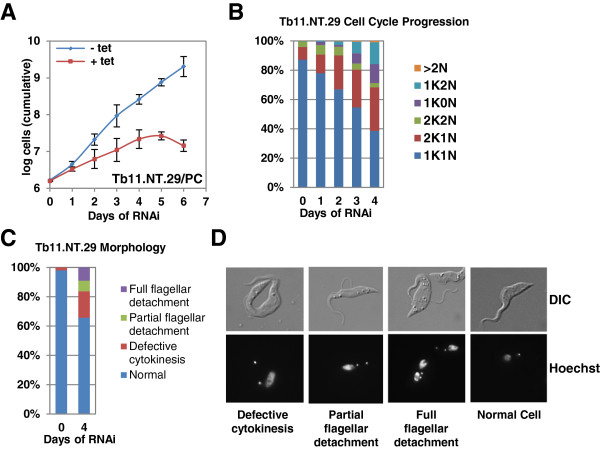
**Growth and cell cycle analysis following RNAi of *Tb11.NT.29. *(A)***Tb11.NT.29* RNAi in procyclics (PC). Growth for un-induced (-tet) and induced cells (+tet) shown in log scale. The data are based on three independent experiments, and average values with standard deviations are presented. **(B)** Analysis of the K/N configurations after induction of *Tb11.NT.29* RNAi in procyclic cells. Cells were scored as having one kinetoplast and one nucleus (1K1N), two kinetoplasts and one nucleus (2K1N), two kinetoplasts and two nuclei (2K2N), one kinetoplast and no nucleus (1K0N), one kinetoplast and two nuclei (1K2N) and more than two nuclei (>2 N). The data are based on three independent experiments and at each time point 1,000 cells were counted. **(C)** Accumulation of cells with altered morphology after induction of *Tb11.NT.29* RNAi in procyclic cells. **(D)** Example images of a normal cell and cells with altered morphology. Images for DNA content (Hoechst) and DIC are displayed. At each time point 500 cells were counted. DIC, differential interference contrast.

To begin to probe the potential role of *Tb*11.NT.29, we monitored the effect of RNAi knockdown on cell cycle progression and cell morphology. In procyclic cells RNAi resulted in a slowdown in growth after two days followed by cell death between day five and six post-induction (Figure 5A), whereas the RNAi effect was more pronounced in bloodstream form cells with cell death occurring between day one and two (Figure 3A). For cell cycle analysis in procyclics, parasites were stained with *Hoechst* at various time points after induction and the number and position of nuclei and kinetoplasts (mitochondrial kDNA) in each cell were recorded. Cells with one kinetoplast and one nucleus (1K1N) are in G1 of the cell cycle, cells with two kinetoplasts and one nucleus (2K1N) have segregated the kinetoplast and are at the end of S phase, and cells with two kinetoplasts and two nuclei (2K2N) have completed mitosis and are poised for cytokinesis [46]. Any other arrangement is aberrant and might point to defects in cell cycle progression. In wild-type cells, as expected for an asynchronously growing cell population, the majority of parasites will be 1K1N with about 10% of cells having either a 2K1N or 2K2N configuration.

RNAi-induced down-regulation of *Tb11.NT.29* in procyclics resulted in the accumulation of cells containing either 1K2N or 1K0N, the latter referred to as zoids (Figure 5B). Zoids and 1K2N cells increased nearly equally in number and after three days of induction they comprised 7.9% and 6.9% of the cell population, respectively. Several morphological changes were observed after the knockdown, including flagellar detachment both as specific areas of separation between the cell membrane and the flagellum, and complete separation with only one visible contact point between the cell body and flagellum (Figures 5C and D). We also noted a change in the shape of the cell body that appeared to be specific to 2K1N or 2K2N cells. After kinetoplast replication and separation, a narrowing of the cell body was evident between the two daughter kinetoplasts (Figure 5D), which might indicate a defect in cytokinesis.

### *Tb*11.NT.28: a mitochondrial inner membrane protein

**Figure 6:**
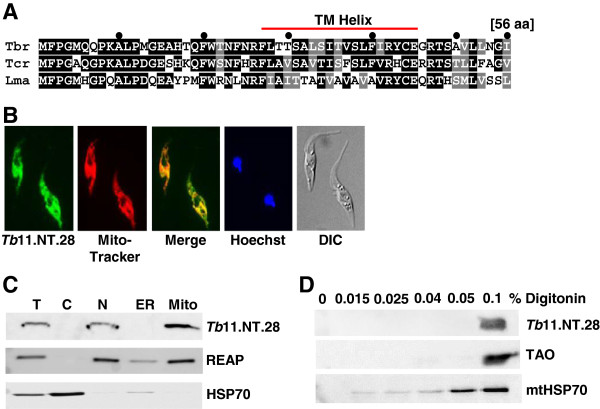
**Characterization of *Tb*11.NT.28. (A)** Sequence conservation of *Tb*11.NT.28 across kinetoplastids. Amino acid sequences from *T. brucei* (Tbr), *T. cruzi* (Tcr) and *L. major* (Lma) were aligned with ClustalW and conserved residues are shaded with black boxes while similar residues are shaded in grey. The predicted transmembrane domain (TM helix) is indicated. **(B)** Immunofluorescence analysis of cells expressing a C-terminal HA-tagged *Tb.*11.NT.28. Cells were stained with an anti-HA antibody (green) and MitoTracker (red) and a merge of signal for HA and MitoTracker is shown in yellow. **(C)** Cell fractionation of cells expressing C-terminal HA-tagged *Tb*11.NT.28. Western blot analysis was performed against C-terminal HA-tagged *Tb*11.NT.28 (top panel), RNA-editing associated protein (REAP, middle panel) and cytoplasmic HSP70 (bottom panel) on total (T), cytoplasmic (C), nuclear (N), endoplasmic reticulum (ER) and mitochondrial (Mito) fractions. **(D)** Digitonin extraction analysis of cells expressing C-terminal HA-tagged *Tb*11.NT.28. Parasites were exposed to increasing concentrations of the detergent digitonin as indicated above the panels and solubilized proteins were analyzed by Western blot for HA-tagged *Tb*11.NT.28 (top panel), trypanosome alternative oxidase (TAO, middle panel) and mitochondrial HSP 70 (mtHSP70, bottom panel).

The 56-amino acid *Tb*11.NT.28 protein is 45% identical between *T. brucei* and *L. major* and contains a predicted trans-membrane domain (Figure 6A). Initial immunofluorescence staining revealed a similar localization pattern between *Tb*11.NT.28 and the fluorescent dye MitoTracker Red, a cell-permeable mitochondrion selective probe (Figure 6B). Cell fractionation experiments corroborated the potential mitochondrial localization in that *Tb*11.NT.28 was enriched in the mitochondrial fraction similar to RNA-editing associated protein (REAP), a known mitochondrial marker [47], whereas the cytoplasmic HSP70 was excluded from this fraction (Figure 6C). To determine in which mitochondrial compartment *Tb*11.NT.28 might be localized, we exposed whole cells to increasing concentrations of digitonin, a detergent that preferentially solubilizes the plasma membrane and the outer membrane of the mitochondria. As the digitonin concentration is increased, specific mitochondrial compartments have been shown to be solubilized with 0.015% digitonin releasing proteins from the inter-membrane space, 0.025% digitonin solubilizing matrix proteins, 0.04% digitonin resulting in release of outer membrane proteins and 0.1% digitonin solubilizing inner membrane proteins [48,49]. As *Tb*11.NT.28 was only released upon exposure to 0.1% digitonin, its likely localization is the inner membrane (Figure 6D), since solubilization of trypanosome alternative oxidase (TAO), a known inner membrane protein of *T. brucei* mitochondria [50], occurred with the same digitonin concentration, whereas a portion of mitochondrial HSP70, a matrix protein, was released with as little as 0.015% digitonin.

**Figure 7:**
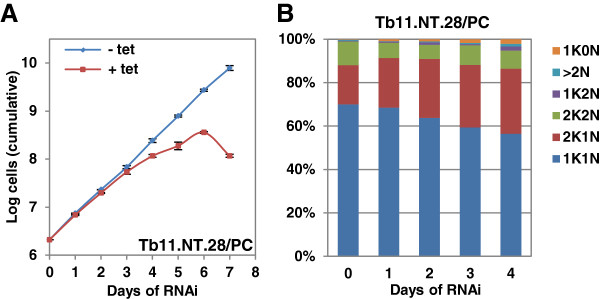
**Analysis of growth and cell cycle progression following RNAi of *Tb11.NT.28*. (A)***Tb11.NT.28* RNAi in procyclics (PC). Growth for uninduced (-tet) and induced (+tet) cells shown in log scale. **(B)** Analysis of the K/N configurations after induction of *Tb11.NT.28* RNAi. Cells were scored every 24 hours as having normal DNA (one kinetoplast and one nucleus (1K1N), two kinetoplasts and one nucleus (2K1N) or two kinetoplasts and two nuclei (2K2N)) or abnormal DNA content (one kinetoplast and no nucleus (1K0N), one kinetoplast and two nuclei (1K2N) and more than two nuclei (>2 N). Cell counts were done as described in the legend for Figure 5.

RNAi-induced knockdown of *Tb11.NT.28* resulted in cell death in procyclic forms (Figure 7A), but did not affect growth in bloodstream forms (Figure 3C). An analysis of cell cycle progression in the procyclic cells following RNAi did not result in an accumulation of cells containing aberrant DNA amounts. However, a steady increase in the number of 2K1N cells was observed with this cell type constituting one third of all cells four days post-induction (Figure 7B). In this category, 50% of cells had duplicated the kinetoplast, but the daughter kinetoplast remained linked in a dumbbell-shaped body, a larger number than seen in wild-type cells [46]. This indicated that although cells entered S phase it had not been completed.

### *Tb*10.NT.87: a mitochondrial matrix protein

**Figure 8:**
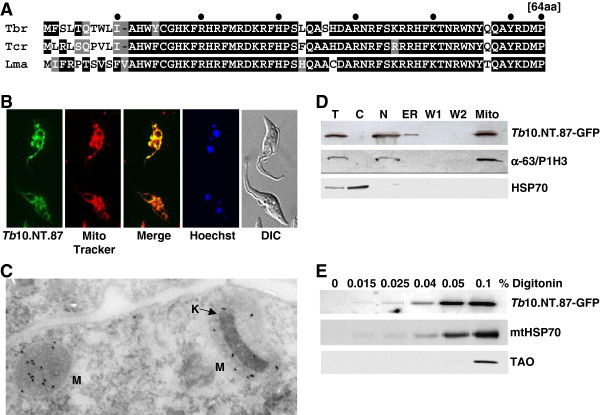
**Characterization of *Tb10.NT.87. *(A)** Sequence conservation of *Tb*10.NT.87 across kinetoplastids. Amino acid sequences from *T. brucei* (Tbr), *T. cruzi* (Tcr) and *L. major* (Lma) were aligned with ClustalW and conserved residues are shaded with black boxes while similar residues are shaded in grey. **(B)** Live fluorescence microscopy of cells expressing a C-terminal GFP-tagged *Tb*10.NT.87. GFP-signal (green), MitoTracker (red), GFP and MitoTracker merge (yellow), Hoechst (blue) and DIC images are shown. **(C)** Immunogold electron microscopy of C-terminal HA-tagged *Tb*10.NT.87. Mitochondrial tubules indicated with the letter M and kinetoplast (mitochondrial DNA) indicated by an arrow and K. **(D)** Cellular fractionation of cells expressing C-terminal GFP-tagged *Tb*10.NT.87. Western blot against C-terminal GFP-tagged *Tb*10.NT.87 (top panel), editing protein (α-63/P1H3, middle panel) and cytoplasmic HSP70 (bottom panel) on total (T), cytoplasmic (C), nuclear (N), endoplasmic reticulum (ER) and mitochondria (Mito) fractions. **(E)** Digitonin extraction analysis of cells expressing C-terminal GFP-tagged *Tb*10.NT.87. Parasites were exposed to increasing concentrations of the detergent digitonin as indicated and solubilized proteins were analyzed by Western blot for GFP-tagged *Tb*10.NT.87 (top panel), mitochondrial HSP 70 (mtHSP70, middle panel), and trypanosome alternative oxidase (TAO, bottom panel). DIC, differential interference contrast.

*Tb*10.NT.87, a very basic protein of 64 aa, is highly conserved in kinetoplastids except for the first 10 aa (Figure 8A). Performing fluorescence microscopy on live cells expressing the GFP-tagged RNAi-resistant construct revealed similar localization patterns between *Tb*10.NT.87 and MitoTracker, indicative of a mitochondrial localization (Figure 8B). Cell fractionation experiments were consistent with the immunofluorescence data (Figure 8D) and digitonin solubilization assays, as described above, suggested that *Tb*10.NT.87 was a matrix protein, since the solubilization properties were similar to mitochondrial HSP70, an established marker for the matrix (Figure 8E). To further pinpoint its cellular localization, we used immunogold electron microscopy of cells expressing HA-TEV-FLAG tagged *Tb*10.NT.87. Micrographs of thin sections showed that the protein was in the mitochondrial matrix and its distribution appeared to be uniform (Figure 8C).

**Figure 9:**
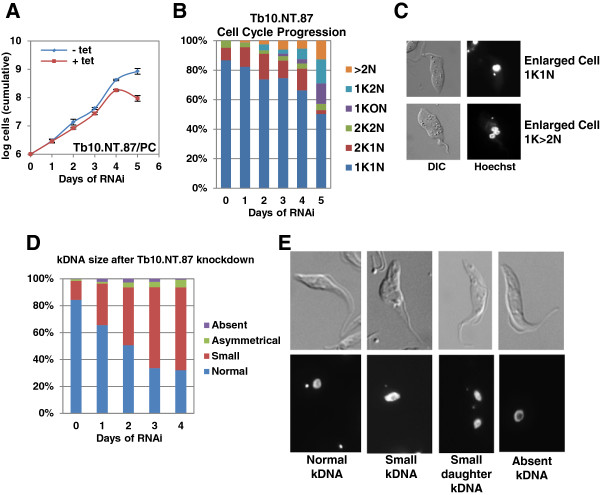
**Analysis of growth and cell cycle progression following RNAi of *Tb*10.NT.87*. *(A)***Tb10.NT.87* RNAi in procyclics (PC). Growth for uninduced (-tet) and induced (+tet) cells shown in log scale. **(B)** Cell cycle progression in procyclic cells after RNAi of *Tb10.NT.87*. Cells were scored every 24 hours as having normal DNA (one kinetoplast and one nucleus (1K1N), two kinetoplasts and one nucleus (2K1N) or two kinetoplasts and two nuclei (2K2N)) or abnormal DNA content (one kinetoplast and no nucleus (1K0N), one kinetoplast and two nuclei (1K2N) and more than two nuclei (>2 N)). **(C)** Example images of abnormal morphology noted after RNAi of *Tb10.NT.87*. Hoechst staining and DIC are shown. **(D)** Examination of kDNA size after *Tb10.NT.87* RNAi in procyclic cells. Cells were scored as having normal kDNA size, small kDNA, absent kDNA or asymmetrical kDNA, which is one cell with two daughter kDNA of different sizes, by visually comparing the kDNA size to that in the uninduced control. **(E)** Examples of cells with normal kDNA, small kDNA (both in G1 and dividing) and absent kDNA. Hoechst and DIC images are displayed. Cell counts were done as described in the legend for Figure 5. DIC, differential interference contrast.

*Tb10.NT.87* was shown to be essential in both procyclic and bloodstream forms (Figures 9A and 3B) and a significant increase in cells with more than two nuclei and grossly enlarged cell bodies was noted after knockdown of *Tb10.NT.87* in procyclics (Figures 9B and C). After four days of induction, 12.5% of cells had between three and eight nuclei. Most of these cells contained a single kinetoplast, indicating that although mitosis was occurring there was not a corresponding replication and division of mitochondrial DNA. The enlarged cell body remained as one unit with a single flagellum as expected in a cell with one kinetoplast. A substantial number of zoids (13.7%) and 1K2N cells (16%) also accumulated by this time point. In accordance with the presence of a single kinetoplast in many cells containing multiple nuclei, a potential defect in kDNA replication was further manifested by the appearance of cells with small kDNA or no detectable kDNA (Figures 9D and E). For example, after three days of *Tb10.NT.87* RNAi, 32% of cells had normal-sized kDNA, whereas 65% of the cells were scored as having a small kDNA.

## Discussion

Although genome sequencing projects have provided a wealth of information about genome structure and organization, they also encountered a challenge to catalogue all protein-coding genes. Since gene annotation programs do not perform well in predicting small proteins, that is, less than 100 aa, it has been common practice to set an artificial length cutoff of 100 aa to avoid flawed predictions. However, recent computational and functional studies highlighted the existence and importance of small proteins in numerous organisms [16-20,51]. Our approach to gauge the extent of the small proteome in *T. brucei* integrated experimental data (transcriptome and MS data) and evolutionary conservation. Starting with 1,117 transcribed sequences not mapping to an annotated CDS [28], 987 have the potential to code for one or more proteins of at least 25 amino acids. This data set was then examined for conserved proteins in related kinetoplastids and representative eukaryotes. A similar strategy was applied in yeast, where more than 60% of the 299 small proteins identified had significant similarities with annotated proteins in other eukaryotes, including humans [19]. Likewise, an analysis of the *M. musculus* small proteome revealed that two-thirds of the potential sORFs identified were conserved in rat and 50% were conserved in human [17]. In contrast, only 1.3% (13) of the predicted ORFs in *T. brucei* had potential homologues in representative eukaryotes, with half of them coding for ribosomal proteins. One possible explanation for this stark difference in evolutionary conservation might be the early divergence of *T. brucei* from other eukaryotes. We observed a comparable lack of conservation when searching for potential homologues in related kinetoplastid species: 16.3% (161) of the 987 transcripts have predicted ORFs with a significant homology to annotated proteins or a six-frame translation of the available genomes. This result was somewhat unexpected, since more than half of the small proteins identified in *Arabidopsis* had homologues in four closely related plants [16] and more than 3,000 sORFs were found to be conserved between *D. melanogaster* and a closely related species, *D. pseudoobscura*[15]. On the other hand, since it has been argued that conservation in closely related species is evidence for translation, the 173 small proteins identified by evolutionary conservation might represent a substantial proportion of the small proteome. The currently available MS data support this view, since the peptide hits were largely confined to the set of 173 proteins. The only exception was the identification of five *T. brucei*-specific proteins. Thus, to fully expose the catalogue of small proteins, future approaches will have to concentrate on the identification of *T. brucei*-specific ORFs through MS analysis or ribosome profiling [52,53].

Examining the functional importance of small proteins in yeast, revealed that 22 (15.5%) of 140 sORF knockout cell lines had an essential phenotype in specific growth conditions [19] while in *Arabidopsis*, overexpression of 10% of a handpicked set of almost 500 small proteins resulted in an abnormal phenotype [20]. Our results showed that 16.7% of the ORFs tested were essential in procyclic cells, while an additional 12% altered normal growth patterns. For example, *Tb*11.NT.28 was essential in procyclics but not in bloodstream-form parasites. We further provided evidence that this small protein of 56 amino acids is likely localized to the inner membrane of the mitochondria. In *T. brucei*, both the size and activity of the mitochondria vary dramatically between life cycle stages. In the bloodstream form, mitochondrial respiratory activity is repressed and a single tubule of the organelle is maintained. On the other hand, in procyclic cells the mitochondrion forms an extensive, branching network and has active respiration. Although further experiments will be required to elucidate the function of *Tb*11.NT.28, examination of cell cycle progression following RNAi induction revealed a decreased ability of the cells to divide replicated kinetoplasts. In contrast, *Tb*10.NT.87, a mitochondrial matrix protein of 64 amino acids, is essential in both procyclic- and bloodstream-form parasites. Ablation of this protein in procyclics resulted in the accumulation of cells containing multiple nuclei and a single kinetoplast, as well as cells with small or no kinetoplast, suggesting that kinetoplast replication is impaired. A similar scenario was observed in bloodstream-form cells upon RNAi of *Tb*10.NT.87 (data not shown). The basic nature of *Tb*10.NT.87 (pI of 11.5) is intriguing and will need further investigation. Nevertheless, our current data are consistent with *Tb*10.NT.87 playing a role in kinetoplast replication, which is an essential process in both life cycle stages [54]. Finally, *Tb*11.NT.29, indispensable in both procyclic and bloodstream stages, is most likely localized on the cell surface. Two days post-induction cells appeared with an asymmetrical hourglass shape and a kinetoplast sequestered in the smaller half of the cell, while the remaining portion contains 1K1N or 1K2N. The proportional accumulation of zoids and 1K2N cells that began after these cells arose suggested that an aberrant cytokinesis of these asymmetrical hourglass cells occurred.

## Conclusions

Our study provides evidence for the existence and importance of small proteins in the human pathogen *T. brucei*. At the same time, it is somewhat puzzling that an unexpectedly low number of transcripts not matching annotated proteins or having conservation with closely related species were identified as containing functional ORFs. Even though there may be small proteins expressed at low levels not yet detectable by MS and others that might be expressed at specific stages of the life cycle, it is tempting to speculate that the *T. brucei* transcriptome includes a substantial number of non-coding RNAs. As all the small proteins identified as essential are unique to kinetoplastids, they may become new targets to block the survival of trypanosomes in the insect vector and/or the mammalian host. The next important question to be tackled is the mechanism of action of these small proteins.

## Methods

### Standard methods

Western blots [55], transfection of procyclic cells [56] and RNA isolation [56] were performed using previously published protocols. Oligonucleotides used to prepare clones and probes for Northern blots are listed in Additional file 2: Figure S9.

### Bioinformatics

The 987 transcripts were translated using the getorf program of the European Molecular Biology Open Software Suite setting a lower limit of 25 aa and including only ORFs that contained a start and stop codon [57].

We used the NCBI BLAST suite (BLAST 2.2.28, [35]) for our protein searches. The predicted 4,699 *T. brucei* ORFs were used as queries for blastp to search all non-redundant kinetoplastid protein sequences (taxid: 5653), using an e-value cutoff of 0.1. *T. brucei* (taxid: 5691). *T. b. gambiense* (taxid: 31285) and *T. b. brucei* (taxid: 5702) sequences were excluded from the search. Similarly, tblastn was used to search the kinetoplastid translated nucleotide database with an e-value cutoff of 0.1. The annotated proteins of *S. cerevisiae* (taxid: 4932), *C. elegans* (taxid: 6239), *A. thaliana* (taxid: 3702), *D. melanogaster* (taxid: 7227), *M. musculus* (taxid: 10090) and *H. sapiens* (taxid: 9606) were queried with the same strategy. All alignments were manually inspected and verified to exclude false positives due to the relaxed threshold.

The predicted *T. brucei* proteins were scanned for domains using the NCBI CD-Search Tool (cdsearch/cdd v3.10; [36]). Transmembrane helices in proteins were predicted using the TMHMM Server v. 2.0 [38,58] and the presence and location of signal peptide cleavage sites were scanned at the SignalP 4.1 Server [37,59]; both servers are at the Technical University of Denmark.

### Mass spectrometry analysis

Procyclic-form trypanosomes (MiTat 1.4) were lysed in 50 mM Tris (pH 7.3) with 4% SDS. To decrease sample complexity, the samples were filtered directly using different (3 kDa, 10 kDa, 30 kDa and 50 kDa molecular weight cut off (MWCO)) Amicon Ultracel centrifugal filter units (Millipore, Billerica, MA, USA) and under strong denaturing conditions with 8 M urea. The filtrate was precipitated using 4 volumes of ethanol and subsequently resuspended in 20 μl 8 M urea. The samples were reduced with 1 mM dithiothreitol (DTT) and alkylated using 5 mM iodoacetamide, prior to digestion with 0.2 μg Lys-C (Wako, Richmond, VA, USA) for three hours followed by digestion with 0.2 μg trypsin (Promega, Madison, WI, USA) overnight. The peptides were desalted using a StageTip [60]. For MS analysis, peptides were separated by a nanoflow liquid chromatography EASY-nLC system on a capillary packed with Reprosil-C18 (Dr. Maisch) with an acetonitrile gradient from 2% to 60% at a flow rate of 250 nl/minute for 230 minutes. The Orbitrap XL mass spectrometer was operated in a data-dependent acquisition mode performing Top10 MS/MS per full cycle. Data analysis was done with MaxQuant version 1.2.0.18 [26] using a concatenated database of TREU 927 v.2.3 (10,533 entries, tritrypDB.org) and the hits generated by the RNA-Seq analysis (987 entries, Additional file 1). Enzyme search specificity was set for tryptic peptides. Up to two miscleavages for each peptide were allowed. Carbamidomethylation on cysteines was set as fixed modification, while methionine oxidation and protein N-acetylation were considered as variable modifications. The search was performed with an initial mass tolerance of 6 ppm mass accuracy for the precursor ion and 0.5 Da. False discovery rate was fixed at one percent on peptide and protein level. For the second data set [22], we reanalyzed previously generated MS data using MaxQuant standard settings with the above described concatenated database. The MS proteomics data have been deposited to the ProteomeXchange Consortium [61] via the PRIDE partner repository [62] with the dataset identifier PXD000711 [22] and PXD000712 (this study).

### Cell culture

*T. brucei* 29.13.6 Lister 427 procyclic cells [41] were cultured at 28°C with 5% CO_2_ in Cunningham’s media supplemented with 10% Tet-system approved heat inactivated fetal bovine serum (FBS, Clontech, Mountain View, CA, USA), 2 mM L-glutamine, 100 units/ml penicillin, 100 μg/ml streptomycin, 50 μg/ml gentamicin, 15 μg/mL G418 and 50 μg/ml hygromycin B. A total of 1 × 10^7^ 29.13.6 cells were used for each procyclic-form transfection. Cells were spun down, washed in Cytomix (20 mM KCl, 0.15 mM CaCl_2_, 10 mM K_2_HPO_4_, 25 mM 4-(2-hydroxyethyl)piperazine-1-ethanesulfonic acid (Hepes), 2 mM ethylenediaminetetraacetic acid (EDTA) and 5 mM MgCl_2,_ pH7.6), then resuspended in 500 μl Cytomix. Then, 25 μg of linearized plasmid DNA was added to the solution and cells were pulsed twice at 1,600 V with a time constant of 0.6 ms on a GenePulser Xcell (BioRad, Hercules, CA, USA). Cells were allowed to rescue for 24 hours before selective drug was added. Phleomycin and blasticidin were added to a final concentration of 2.5 μg/ml and 10 μg/ml, respectively. Transfected cells were cloned at least 24 hours after transfection. Serial dilutions of the cells were then made in media with 20% serum and the presence of 3 × 10^7^ 29.13.6 cells that had not been transfected. A total of 200 μl of transfected cells were added to 1.8 ml of the cloning media and further diluted in six, five-fold dilutions. Each dilution was plated in a 96-well plate and clones were selected from dilutions where fewer than 30% of wells had growth. *T. brucei* SM Lister 427 bloodstream-form cells [41] were maintained at 37 °C with 5% CO_2_ in HMI-9 media supplemented with 10% Tet-system approved heat inactivated FBS (Clontech), 100 units/ml penicillin, 100 μg/ml streptomycin, 50 μg/ml gentamicin, and 2.5 μg/ml G418. For bloodstream form transfections, 3 × 10^7^ cells were centrifuged, washed quickly with Tb-BSF buffer [63], resuspended in 100 μl Tb-BSF buffer containing 10 μg of plasmid DNA and transfected with protocol X-01 in an AMAXA Nucleofector®. Transfected cells were placed in 30 ml pre-warmed HMI-9 media and two 10-fold serial dilutions were plated in a 24-well plate. Six hours after transfection, pre-warmed medium supplemented with appropriate selectable drugs was added. The final concentration of selectable markers was 2.5 μg/ml phleomycin and 5 μg/ml blasticidin. For induction of hairpin or RNAi-resistant construct expression, 10 μg/ml and 1 μg/ml of doxycycline was added to procyclic- and bloodstream-form cells, respectively.

### Plasmid constructions

#### Constructs encoding hairpin RNAs

We followed the Gateway®-adapted cloning scheme developed by Margaret Phillips and colleagues [40]. Briefly, a 300 to 400 base pair region was PCR amplified and TA-cloned (deoxythymidine, T, deoxyadenosine, A) into plasmid pCR/8GW/TOPO (Invitrogen, Grand Island, NY, USA) to generate an entry clone. The entry clone was then recombined with the destination vector pTrypRNAiGate. Final constructs were verified by restriction enzyme digestions and DNA sequencing.

#### RNAi-resistant constructs

To rescue the lethal RNAi phenotype, RNAi-resistant versions of the CDS were synthesized by GeneWiz, Inc, Cambridge, MA, USA. A silent mutation was introduced every twelfth nucleotide [44], a C-terminal HA-TEV-FLAG tag was added and the CDS was flanked at the 5’ and 3’ end by a Hind III and Bam HI restriction site, respectively. The RNAi-resistant construct was cloned into the inducible pLew100v5 BSR plasmid flanked by the GPEET procyclin 5’ UTR and an aldolase 3’UTR. In addition, inducible GFP-tagged RNAi-resistant constructs were generated in pLew100v5 BSR. The plasmids containing the RNAi-resistant constructs were digested with Not I to allow for recombination into a *T. brucei* ribosomal RNA spacer region following transfection. For each knock-down, ten clones were tested for a phenotype.

### Northern blotting

Total RNA was separated on 1.5% agarose gels in the presence of 6.3% formaldehyde in 40 mM 3-morpholinopropane-1-sulfonic acid (MOPS) and 2 mM EDTA. RNA ladder 0.5 to 10 kb (Invitrogen) was used as marker. The RNA was transferred overnight to a Hybond-N nylon membrane (GE Healthcare, Little Chalfont, United Kingdom) by capillary transfer with 10× SSC (0.15 M sodium citrate, 1.5 M sodium chloride), UV cross-linked to the membrane and stained with methylene blue. The membrane was pre-hybridized for one hour in 5× SET (0.75 M sodium chloride, 5 mm EDTA, 0.15 M Tris-HCl, pH 7.4), 10× Denhardt’s solution, 1% SDS and 100 μg/ml yeast RNA, and then hybridized overnight in the same solution. DNA probes were internally labeled by synthesis with specific dsDNA templates, sense and antisense primers and Pfx DNA polymerase (Invitrogen) in the presence of [α-^32^P]dCTP. The membrane was washed two to three times (10 minutes each) with 2× SSC, 0.1% SDS and hybridization signals were detected by PhosphorImager.

### Semi-quantitative reverse transcriptase (RT)-PCR

For each sample, 5 μg of total RNA was treated with 2 units of DNase RQ (Promega), phenol extracted and ethanol precipitated. DNase-treated RNA was reverse transcribed using random primers (Promega) and Superscript II enzyme (Invitrogen) according to the manufacturer’s protocol. Twenty-two cycles of PCR were then performed using Platinum Pfx (Invitrogen), according to the manufacturer’s instructions, for each knockdown with histone 4 used as a control. An annealing temperature of 50°C was used for all oligonucleotides with an extension time of 30 seconds.

### Immunofluorescence

A total of 5 to 8 × 10^6^ cells were spun down and washed twice with cell wash (20 mM Tris–HCl (pH 7.5), 100 mM NaCl and 3 mM MgCl_2_) before being placed on slides coated with poly-L-lysine and settling for five minutes. Then, 4% paraformaldehyde (PFA) was added and the cells were fixed for 30 minutes at 4°C. Cells were washed twice and then exposed to 0.1% NP-40 detergent in a solution of 2% goat serum. Slides were washed again and blocked with 10% goat serum for 10 minutes. Upon removal of blocking solution, primary antibody, diluted in 2% goat serum, was administered for one hour. Anti-GFP (Roche, Basel, Switzerland), anti-HA (Covance, Princeton, NJ, USA), and α-GPEET procyclin (Cedarlane labs, Burlington, Ontario, Canada) antibodies were obtained commercially. The antibodies to BiP were generously provided by Jay Bangs. Cells were washed five times, before the addition of secondary antibody and 5 μg/ml Hoechst (Cell Signaling Technology, Inc, Beverly, MA, USA). Cells were exposed to secondary antibody, diluted in 2% goat serum, for one hour. All secondary antibodies (Alexa Fluor 488-conjugated goat anti-mouse, 594-conjugated goat anti-mouse, 488-conjugated goat anti-rabbit and 594-conjugated goat anti-rabbit (Invitrogen)) were used at a 1:1,000 dilution. Samples were washed five times for 10 minutes total. Wash solution was removed and FluorSave (Calbiochem, Darmstadt, Germany) was added. Next, the coverslip was placed on the slide, and FluorSave reagent dried for two hours to overnight before cells were imaged on a Zeiss (Jena, Germany) Axioplan 2 fluorescence microscope. For kDNA size estimation, cells were fixed and stained with Hoechst, following the protocol outlined above. Cells were imaged and kDNA size was compared visually between un-induced cells and induced cells. 

### MitoTracker

A total of 1 × 10^7^ cells was spun down, washed once in cell wash (20 mM Tris–HCl (pH 7.5), 100 mM NaCl and 3 mM MgCl_2_) and resuspended in Cunningham’s media with no added serum. MitoTracker Red CM-H2xRos (Invitrogen) was added to a final concentration of 1 μM. Cells were incubated at 28°C and 5% CO_2_ for 10 to 15 minutes, centrifuged, washed and placed in fresh media without serum. Cells were rescued in media without MitoTracker for 25 minutes, then Hoechst (5 μg/ml) was added; cells were spun down and finally resuspended in 20 to 50 μl of PBSG.

### Live cell imaging

A total of 5 to 8 × 10^6^ cells was collected, Hoechst (5 μg/ml) was added and the cells were incubated in the dark for two minutes. Cells were centrifuged, washed once in phosphate-buffered saline with glucose (PBSG), resuspended in 20 to 50 μl of PBSG, and imaged on a Zeiss Axioplan 2 fluorescence microscope.

### Cell fractionations

#### Cell compartment qproteome kit (Qiagen)

Each fractionation used 1 × 10^9^ cells expressing GFP-tagged *Tb11.NT.29* and the manufacturer’s (Qiagen, Venlo, Limburg, The Netherlands) instructions were followed. The anti-HSP70 and anti-BiP antibodies were generously provided by Jay Bangs.

#### Mitochondrial isolation qproteome kit (Qiagen)

Each fractionation used 1 × 10^9^ cells expressing GFP-tagged *Tb10.NT.87* or *Tb11.NT.28* and the manufacturer’s (Qiagen) instructions were followed. The antibodies to REAP and *Tb*MP63 were generously provided by Steve Hajduk and Ken Stuart, respectively.

#### Digitonin solubilization assay

Cells (1 × 10^8^) expressing GFP-tagged *Tb10.NT.87* or HA-tagged *Tb11.NT.28* were spun down for each assay, washed twice with 20 mM sodium phosphate (pH 7.9), 20 mM glucose and 0.15 M NaCl, and then resuspended in 500 μl SoTE buffer (20 mM Tris–HCl (pH 7.5), 0.6 M sorbitol, 2 mM EDTA). Next, 500 μl of SoTE buffer with varying digitonin amounts was added to each sample to a final concentration of detergent of 0.015%, 0.025%, 0.04%, 0.05% or 0.1% [48,49]. Samples were incubated for five minutes at 4°C followed by centrifugation for three minutes at 5,000 g at 4°C. SDS-sample buffer was added to the supernatants and samples were analyzed by SDS-PAGE and Western blotting. The antibodies to TAO and mtHSP70 were generously provided by Minu Chaudhuri and Jay Bangs, respectively.

### Electron microscopy

Samples were fixed in 4% PFA/0.1% gluteraldehyde in PBS for 30 minutes followed by further fixation in 4% PFA for one hour, rinsed in PBS, scraped and re-suspended in 10% gelatin. Chilled blocks were trimmed, placed in 2.3 M sucrose overnight on a rotor at 4°C, transferred to aluminum pins and frozen rapidly in liquid nitrogen. The frozen blocks were cut on a Leica Cryo-EMUC6 UltraCut and 65 nm thick sections were collected using the Tokoyasu method [64] and placed on carbon/formvar coated grids and floated in a dish of PBS for immunolabeling. Grids were placed section side down on drops of 0.1 M ammonium chloride to quench untreated aldehyde groups, then blocked for nonspecific binding on 1% fish skin gelatin in PBS. Single labeled grids were incubated on a primary antibody mouse anti-HA (Covance) 1:50 dilution, which required a rabbit anti-mouse bridge (JacksonImmuno, West Grove, PA, USA). The secondary antibody was 10 nm Protein A gold (Utrecht Medical Center). All grids were rinsed in PBS, fixed using 1% gluteraldehyde for five minutes, rinsed again and transferred to a UA/methylcellulose drop before being collected and dried. Samples were viewed using a FEI Tencai Biotwin TEM at 80 Kv. Images were taken using Morada CCD and iTEM (Olympus) software.

## Abbreviations

aa: amino acids; BLAST: Basic Local Alignment Search Tool; CDS: coding sequences; dsRNA: double-stranded RNA; EDTA: ethylenediaminetetraacetic acid; ER: endoplastic reticulum; GFP: green fluorescent protein; MS: mass spectrometry; PBS: phosphate-buffered saline; PFA: paraformaldehyde; REAP: RNA-editing associated protein; RNA-Seq: RNA sequencing; RRM: RNA recognition motif; RT-PCR: reverse transcriptase-polymerase chain reaction; snoRNA: small nucleolar RNA; snRNA: small nuclear RNA; sORF: short open reading frame; TAO: trypanosome alternative oxidase; uORF: upstream open reading frame; UTR: untranslated region.

## Competing interests

The authors declare that they have no competing interests.

## Authors’ contributions

ME, MAJ, FB, MM, EU and CT participated in the study design. ME, MAJ and FB performed the experiments. CT and FB performed the bioinformatics analyses. MM contributed novel instrumentation. ME, MAJ, FB, EU and CT contributed to the data analysis. ME and CT wrote the manuscript. All authors read and approved the final manuscript.

## Supplementary Material

Additional file 1 **Coding potential of the 987 transcripts described in Figure **1**.** Setting a lower limit of 25 aa, all the potential ORFs are listed. For example Tb1.NT.1_1 refers to the novel transcript Tb1.NT.1 according to the nomenclature by Kolev *et al*. [28] and _1 indicates ORF #1. The numbers in parenthesis specify the CDS. Click here for file

Additional file 2: Figure S1 Length distribution of the predicted 4,699 ORFs. **Figure S2.** Growth analysis of the five small proteins with faster or slower growth in procyclics following induction of RNAi. **Figure S3.** Growth analysis of the seven small proteins essential in procyclics following induction of RNAi with and without expression of an RNAi-resistant construct. The data are based on three independent experiments. **Figure S4.** Northern blot analysis of essential ORFs. Northern blots for six (*Tb11.NT.108*, *Tb10.NT.90*, *Tb10.NT.86*, *Tb10.NT.87*, *Tb11.NT.28* and *Tb3.NT.18*) of the seven essential transcripts. Marker sizes in kb are indicated on the left. The predicted transcript size is indicated below each panel. Northern blot analysis for *Tb11.NT.29* can be found in Kolev NG, Franklin JB, Carmi S, Shi H, Michaeli S, Tschudi C: The transcriptome of the human pathogen *Trypanosoma brucei* at single-nucleotide resolution. *PLoS Pathog* 2010, 6:e1001090 [28]. **Figure S5.** Semi-quantitative RT-PCR analysis of RNAi knockdown. RT-PCR was performed on the transcripts encoding each essential small protein from procyclic cells un-induced (-) or induced (+) for RNAi. Histone 4 (H4) was used as a control. The small protein transcript and H4 are indicated to the right of each panel. **Figure S6.** Listing of the seven essential proteins in procyclics with a C-terminal GFP or HA tag. All constructs rescued the lethal RNAi phenotype. **Figure S7.** Western blot analysis of GFP-tagged proteins encoded by an RNAi-resistant construct. Size markers (kDa) are shown at the left. **Figure S8.** Growth analysis of bloodstream-form cells following RNAi against four small proteins. Growth for un-induced (tet-) and induced (tet+) cells are shown in log scale. The data are based on three independent experiments. **Figure S9.** Listing of oligonucleotides used in this study. Click here for file

Additional file 3 **Listing of the 178 transcripts (sheet 178 ORFs) and 42 transcripts (sheet 42 ORFs) encoding potential ORFs with matching peptides and hits in Kinetoplastids.** Panigrahi *et al*.: A comprehensive analysis of *Trypanosoma brucei* mitochondrial proteome. *Proteomics* 2009, 9:434–450 [21]. Butter *et al*.: Comparative proteomics of two life cycle stages of stable isotope-labeled *Trypanosoma brucei* reveals novel components of the parasite's host adaptation machinery. *Mol Cell Proteomics* 2013, 12:172–179 [22]. viv, *T. vivax*; cru, *T. cruzi*; Leish, *Leishmania*; con, *T. congolense*; evansi, *T. evansi*. PTM Abbreviations: P, phosphorylation; S, sumoylation; PM, palmitoylation; G, glycosylation. Phosphorylation prediction: NetPhos http://www.cbs.dtu.dk/services/NetPhos/; Sumoylation prediction: http://sumosp.biocuckoo.org/; Palmitoylation Prediction: http://csspalm.biocuckoo.org/; Glycosylation Prediction: http://www.cbs.dtu.dk/services/YinOYang/. Click here for file

Additional file 4 ***T. brucei *** **predicted ORFs conserved in representative eukaryotes.** BLAST bit scores of *T. brucei* predicted ORFs with potential homologs in representative eukaryotes. Bit scores are shown after the accession number and they represent a normalized version of the raw BLAST alignment score. Click here for file

Additional file 5 **Survey of conserved domains in 173 *T. brucei *** **predicted ORFs.** Click here for file
