# Supplementary material for: On the extent and role of the small proteome in the parasitic eukaryote Trypanosoma brucei
Source: BMC Biol. 2014 Feb 19;12:14. doi: 10.1186/1741-7007-12-14 (PMC3942054; doi:10.1186/1741-7007-12-14)
Supplement: Additional file 1 — Coding potential of the 987 transcripts described in Figure 1. Setting a lower limit of 25 aa, all the potential ORFs are listed. For example Tb1.NT.1_1 refers to the novel transcript Tb1.NT.1 according to the nomenclature by Kolev et al. [28] and _1 indicates ORF #1. The numbers in parenthesis specify the CDS. [file 1741-7007-12-14-S1.docx]

**Additional File 1. Coding potential of the 987 transcripts described in Fig. 1.**

Setting a lower limit of 25 aa, all the potential ORFs are listed. For example Tb1.NT.1_1 refers to the novel transcript Tb1.NT.1 according to the nomenclature by Kolev et al. [22] and _1 indicates ORF #1. The numbers in parenthesis specify the CDS.

>Tb1.NT.1_1 [175 - 438]

MQTLEAVTGCAVSYTSCYNYIIHVSYGCSYGAALDGVLRRHAAGDVIVSIFLSLSLSPSLSFFNYVSVGKHAMICCLLCPYNHFCYPG

>Tb1.NT.2_1 [8 - 82]

MKEGRCKFKKLVLKCQTCPLQYVHH

>Tb1.NT.2_2 [58 - 165]

MSTSVRPSLTETTNLHGVVSLCGSFRTDGKRCECTK

>Tb1.NT.2_3 [767 - 919]

MGCNCTFICFCKFTTLREQRHFSTGELFHHFYWTHRRGCCFWGPSLQYGNR

>Tb1.NT.2_4 [720 - 950]

MRCFYALAENHYRMPAWDATALLFVFVSLRLSESNAISVQENSFIISIGHTDADVVFGVRLYNTVTGEPIKQTQHML

>Tb1.NT.2_5 [1045 - 1122]

MAKRNYTAPKKKRKMLYHTHTAGKPQ

>Tb1.NT.2_6 [1040 - 1270]

MRWQKEITQLRKKREKCYTTLTRQASLNSGFWGEPSKEIQKRYPQSHTMRNYQLGKGHCQCEKSHREEPLEERGRSS

>Tb1.NT.2_7 [1011 - 1295]

MSSDLNYTQPCDGKKKLHSSEKKEKNVIPHSHGRQASIVDFGGNLPKKFKSDTHRVIRCVIINWEKDIASVRKVIGRNLWRKGEEVHNDYHKGKM

>Tb1.NT.2_8 [1324 - 1398]

MIEHSIKRRALLTRGAAVVAMEARH

>Tb1.NT.2_9 [1442 - 1525]

MELPRSTVWYLRCVTTAVSKSILWVFLM

>Tb1.NT.2_10 [1477 - 1560]

MCDNCRQQVDSLGVFDVRYNWKECAAPV

>Tb1.NT.2_11 [1662 - 1757]

MRKKKDFTSPIGNECGNDFPSVDLTEDDASSE

>Tb1.NT.2_12 [1741 - 1857]

MMQAASKRFDEGWSIKNSTDDAERNKERKYQRRSQNCAL

>Tb1.NT.2_13 [1769 - 1897]

MRVGALRIAQMMQKETKRESIKEGVRIAHCRKVCPNNEAAAYH

>Tb1.NT.2_14 [1928 - 2005]

MGCVPLFVMEEKQNWKKALVYCMTKG

>Tb1.NT.2_15 [2320 - 2439]

MDMQHMYQCEAHDVVKCRGNKIVTSMKKCGAQPLLVTHKR

>Tb1.NT.2_16 [2367 - 2474]

MSWKQNSYQHEEMWGTTIIGDSQKMTQQCDFIGVLV

>Tb1.NT.2_17 [2480 - 2566]

MKSRQYGWDNESDTTIPSIPCYVKKTTIK

>Tb1.NT.2_18 [2587 - 2787]

MHDVRGSRSEGKTIAFSLLLLLLLIFLMKACIMNGFGLRSAEYYFVRLHFSKHTCTPRLFTQLSARE

>Tb1.NT.2_19 [2687 - 2845]

MALDLGVQSIILYGYISLNTPVLPVYLHSCLQESEIFRKPTQHNPLRPAQETS

>Tb1.NT.2_20 [2912 - 3028]

MMRKPYCLFSMIIVSYIFLVGSKKGKVRTKLFIICYYFC

>Tb1.NT.2_21 [3241 - 3315]

MIPSLHPKLTLLYRLTLNGWKDVHA

>Tb1.NT.2_22 [3888 - 3980]

MLLAVVLYVYRNKITCEKSCVIFLDFYSSTF

>Tb1.NT.2_23 [4084 - 4167]

MIMLRRWHICVLCTFCNSSVRVANYNMW

>Tb1.NT.2_24 [4478 - 4588]

MLSNIMSKLYICVNVYIICVVTYGILKAYERNCSNSL

>Tb1.NT.2_25 [5133 - 5237]

MYIFQKWKPVAIIFRYSIFMRDHFLLEATSLFLQL

>Tb1.NT.2_26 [5024 - 5248]

MSYSNTRNKIRKTLLIVNCNMRSDTLQIWLHIPHVVYVYISKMETRCYHFQILNIYARSFPIGSNEFISAAMKFG

>Tb1.NT.2_27 [5107 - 5277]

MASYTSCSLCIYFKNGNPLLSFSDTQYLCAIISYWKQRVYFCSYEVWMNELHSSVSK

>Tb1.NT.2_28 [5252 - 5362]

MNCTHLSVNEFGCYLYLYFLLHAAVALCYGFSSMSKL

>Tb1.NT.2_29 [5277 - 5369]

MNSDVIYIYIFYCMQQSLCVMDSRACQSYDQ

>Tb1.NT.2_30 [5287 - 5418]

MLFIFIFFIACSSRFVLWILEHVKVMISNFLHAYKSVIRHWKCN

>Tb1.NT.2_31 [5700 - 5798]

MLFFMAMPHFFLLFHKYIYISIFFFSGMIVCSS

>Tb1.NT.3_1 [242 - 322]

MKGDRMRKIGNLLIISTITFLCNSTMC

>Tb1.NT.3_2 [764 - 868]

MYIFQKWKPVAIIFRYSIFMRDHFLLEATSLFLQL

>Tb1.NT.3_3 [655 - 879]

MSYINTRNKIRQTLLIVNCNMRSDTLQIWLHIHHVVYVYISKMETRCYHFQILNIYARSFPIGSNEFISAAMKFG

>Tb1.NT.3_4 [738 - 908]

MASYTSCSLCIYFKNGNPLLSFSDTQYLCAIISYWKQRVYFCSYEVWMNELHSSVSK

>Tb1.NT.3_5 [883 - 993]

MNCTHLSVNEFGCYLYLYFLLHAAVALCYGFSSMSKL

>Tb1.NT.3_6 [908 - 1000]

MNSDVIYIYIFYCMQQSLCVMDSRACQSYDQ

>Tb1.NT.3_7 [918 - 1049]

MLFIFIFFIACSSRFVLWILEHVKVMISNFLHAYKSVIRHWKCN

>Tb1.NT.3_8 [1245 - 1328]

MLSSITIVFRQFISTMTHCYISTCRCIM

>Tb1.NT.3_9 [1402 - 1527]

MRDVFHFIICQFIHPLLHLLYIFSSSLDVLFYVFVIPIALCI

>Tb1.NT.3_10 [1861 - 1956]

MFLFIMKVNRNTLWLSDAKRLSVFFTCPTDII

>Tb1.NT.3_11 [2100 - 2177]

MCVYLCVVPILFFFVSLVSINFLFFV

>Tb1.NT.3_12 [2018 - 2182]

MSLFNASEYYVLTEVRKLQWNSCVVRIHVCISMCCADTLFLRFISIHKFFVFCLT

>Tb1.NT.5_1 [286 - 435]

MEGQGHLCFCAHNILHMMSHSSFYLFVQVQFICLRYVCWCYLFELFIGCK

>Tb1.NT.5_2 [392 - 472]

MYAGVICLSCLLGVNNTFAMDDTACVH

>Tb1.NT.6_1 [8 - 88]

MMLVKIHRGISKEAFMVPITCAPHNFP

>Tb1.NT.6_2 [383 - 502]

MSYINTRNKIRQTLLIVNCNMRSDTLPIWLHIPHVVYAYI

>Tb1.NT.6_3 [509 - 607]

METRCYHFQILNIYAGLFPIGSSEFIFAAMKFG

>Tb1.NT.6_4 [466 - 636]

MASYTSCSLCIYLNNGNPLLSFSDTQYLCGIISYWQQRVYICSYEVWVNELHSSVSK

>Tb1.NT.6_5 [636 - 728]

MNSDVIFIFIFYCMQQSLCVMNSRACQSYHQ

>Tb1.NT.6_6 [611 - 754]

MNCTHLSVNEFGCYFYFYFLLHAAVALCYEFSSMSKLSSVTSFMHIKV

>Tb1.NT.6_7 [973 - 1056]

MLSSITIVFRQFISTMTHCYISTYRCIM

>Tb1.NT.6_8 [1601 - 1720]

MFLFIMKVNRNTLWLSDAKRLSVFFYLSYRHYLIFSFGSK

>Tb1.NT.6_9 [1759 - 1899]

MSLFNASECYVLTEVRKLQWNSCVVVWCVFMCVYIYVLCRYSFSSLH

>Tb1.NT.6_10 [1782 - 1946]

MLRIDRSEETPVEQLCSCVVCIHVCVYLCVVPILFFFASLISINFLFIVLLDFNQ

>Tb1.NT.6_11 [1993 - 2073]

MQVYEAVIFFVCMLFIVKEMMYEVHYI

>Tb1.NT.7_1 [233 - 343]

MQLMRSCSILTVPRRSSFAALFPPFCGIGGEGGGAGA

>Tb1.NT.7_2 [449 - 565]

MYLKDTSSWAVSGYVLFPLCEFGVSWAANIRRVDITCVC

>Tb1.NT.7_3 [665 - 739]

MRSVSSSDFNCGIEAADIVSRVVCS

>Tb1.NT.7_4 [1099 - 1203]

MLHNIFLRFKKLVIRYNPCILLFIFCLRKKEKKGE

>Tb1.NT.7_5 [1894 - 1974]

MKRDRMRKIGNLLIISTITFLCNSTMC

>Tb1.NT.7_6 [2408 - 2521]

MYFMHIFQKWKPVAIIFRYSIFMRDYFLLEAASLCLQL

>Tb1.NT.7_7 [2299 - 2532]

MILMSYSNTRNKIRQTLLIVNCNMRSDTLPIWLHIPHVFYAYISKMETRCYHFQILNIYAGLFPIGSSEFMFTAMKFG

>Tb1.NT.7_8 [2391 - 2561]

MASYTSCILCIYFKNGNPLLSFSDTQYLCGIISYWKQRVYVYSYEVWVNELHSSVGK

>Tb1.NT.7_9 [2561 - 2653]

MNSDVIYIFIFYCMQLLLCVMNSRACQSYHQ

>Tb1.NT.7_10 [2536 - 2679]

MNCTHLSVNEFGCYLYFYFLLHAAAALCYEFSSMSKLSSVTSFMHIKV

>Tb1.NT.7_11 [2769 - 2843]

MVLHAIPIVTHVWDYVNYYLKPLKT

>Tb1.NT.7_12 [2804 - 2890]

MGLCELLFETIENLMEKEGNVDRIILLIF

>Tb1.NT.7_13 [3153 - 3227]

MCCFIFCYSNSFVYVSNRKLTMMIQ

>Tb1.NT.7_14 [3325 - 3459]

MGSSINCINFIPIFYNVLYHRYHKRCVNSSNNDGTSDSYECVGLN

>Tb1.NT.7_15 [3525 - 3647]

MMFLFIMKVNRNTHWLSDAKRLSVFFYLSYRHYLIFSFGSK

>Tb1.NT.7_16 [3786 - 3869]

MCCSDNLFLRFINIHIFLLFIVLLDFNQ

>Tb1.NT.7_17 [3700 - 3876]

MQVNIMNCKNGEDIQRSNCVVRIHVCMYLCVVPIIFFFVSLISIYFYCLLFYLILINRI

>Tb1.NT.8_1 [133 - 243]

MYLFEKKKRRKEALHLKQDACEFEEQLSFEYHIPKLF

>Tb1.NT.8_2 [188 - 289]

MHVSSKNNCHLNIISPNCFDYIISSKCLRNLGSE

>Tb1.NT.8_3 [333 - 419]

MKVTLVGFLYIGKWSGFKVFYMMFSWRLK

>Tb1.NT.8_4 [447 - 608]

MIQESFRLLISSSLPRRTIFGLHCRRWYDICNRLFLIYLFLRVYISVVWWRGKF

>Tb1.NT.8_5 [536 - 619]

MQPLISYLFIFASLYKCGLVAWKILACG

>Tb1.NT.8_6 [713 - 934]

MLQLTHSSNQNTFSAFPLSAKNVVQFPNSTPHLIHHRVKRYLYGQHESGEVQKVEIKNIYIYIELCWNCRYHST

>Tb1.NT.8_7 [1069 - 1143]

MLMRDHYAEGRTADDCNLKPVICGL

>Tb1.NT.8_8 [984 - 1202]

MKLQVEGVDSNHSSPNLCNVNMFVERYSYVNERPLCRGKNSRRLQPKTRNLRTVINFNSFILLCKDGISRLYL

>Tb1.NT.8_9 [1180 - 1284]

MASADYTCKLFEVKTTIHGRECTSGDLNRMFMRNL

>Tb1.NT.8_10 [1308 - 1397]

MLCDINTRFFFFILFFFSFFFCLSVEQSEV

>Tb1.NT.8_11 [1426 - 1548]

MCCCLYNTNRYKLNICSKYIKSIRFVTTSCLISLYSNGTFF

>Tb1.NT.8_12 [1762 - 1878]

MAHHYQFVCYELTINHEKGFYDSYSLHKCNALKECNLIN

>Tb1.NT.8_13 [2033 - 2131]

MVRETLICLERADAHSSKGFRKVMGNFNHIFYY

>Tb1.NT.8_14 [2149 - 2283]

MFLFFCIALTFKTNWAALRRQSSGVWKYLRIGGSLNCDDYVPNGD

>Tb1.NT.9_1 [44 - 136]

MTQIKGSFYRSHHFHVCNSNRVIVTIYFLRV

>Tb1.NT.9_2 [255 - 503]

MAERTETVVKHTLQSEKSISPGADTLQRCEHKLFIYEQEASVCLFVCLFLNPFLTPASNIKYREFRNFIMTIIVVSCKMMHIL

>Tb1.NT.9_3 [503 - 703]

MIGTYWAPAANKEIRKNTTVINIYYLEVTCVHLRGISSVVFANSENEENQQSDEMNKIPNVVHSLEA

>Tb1.NT.10_1 [17 - 253]

MYEGFCARSRGVQFTIYLMESHYFECAIRCTSTGYRHSVCYHLPYLSYDYSKMIRVQRYEKCNNTREVQFQLLMGNIYV

>Tb1.NT.10_2 [392 - 595]

MYSITLRMQWDLGVKHFGNCICVCMDELEPIEPAVAYQQEWKLVEQFLYVSLYCGELSLISLLTFVVY

>Tb1.NT.10_3 [702 - 809]

MHEIHYFYIWHMITRQGKYLYSCRILELFSLIPNKV

>Tb1.NT.11_1 [308 - 382]

MLMRDHYAEGRTTDDCNLKPVICGL

>Tb1.NT.11_2 [223 - 441]

MKLQVEGVDSNHSSPNLSNVNIFVERYLYVNERPLCRGKNNRRLQPKTRNLRTVINFNSFILLCKDGISKLYL

>Tb1.NT.11_3 [419 - 523]

MASANYTCKLFEVKTTIRGRECAGGDLNRMFIGNL

>Tb1.NT.11_4 [547 - 642]

MLCDINTRCFFFHIILLFFFFFACLSNNLRFS

>Tb1.NT.11_5 [667 - 789]

MCCCLYNTNRYKLNICSKYIKSIRFVTTSCLISLYSNGTFF

>Tb1.NT.11_6 [1264 - 1362]

MVRETLICLERADAQSSKGFRKVMGNFNHIFYY

>Tb1.NT.11_7 [1380 - 1523]

MFLFFCIALTFKTNWAALRRQSSGVWKYLRIGGSLNCDDYVPNGDGKK

>Tb1.NT.12_1 [184 - 285]

MHVKSKNNCHLNIIFQNCFAYIISSKCLRNLGSE

>Tb1.NT.12_2 [329 - 427]

MKVTLVGFLYIGKWSGFKVFCMMFSWRLKYFMK

>Tb1.NT.12_3 [443 - 571]

MIQESFRLLISSSLPRRTIFGLHCKKWYDICNRLFIIYFFEFI

>Tb1.NT.12_4 [525 - 611]

MIYATAYLLFIFSSLYKCGLVAWKILACG

>Tb1.NT.13_1 [55 - 186]

MLIPHSESRDCRLHFIITTHTHTDILNIRMIMLRRWRIRIFELY

>Tb1.NT.14_1 [85 - 195]

MLSNILSKLCRRIYIYIICIVTYGILKAYERNCSNSL

>Tb1.NT.14_2 [220 - 300]

MKGDHVRKIGNLWIISTITFLCNSTMC

>Tb1.NT.14_3 [742 - 846]

MHIFQKWKPVAIIFRYSIFMRDYFLLEAASLFLQL

>Tb1.NT.14_4 [624 - 857]

MILMSYSNTRNKIRKTLLILNCNMRSNTLPIWLHIPHVVYAYISKMETRCYHFQILNIYAGLFPIGSSEFIFAAMKFG

>Tb1.NT.14_5 [716 - 886]

MASYTSCSLCIYFKNGNPLLSFSDTQYLCGIISYWKQRVYFCSYEVWVNGLHSSVSK

>Tb1.NT.14_6 [886 - 972]

MNSDVIFIFYCMQLSLCVMNSRACQSYHQ

>Tb1.NT.14_7 [861 - 998]

MDCTHLSVNEFGCYFYFLLHAAVALCYEFSSMSKLSSVTSFMHIKV

>Tb1.NT.14_8 [1393 - 1488]

MIVFYEWELLFEFSSKQKCLFILSYLICAKLL

>Tb1.NT.14_9 [1406 - 1525]

MNGNCCLSFPPNRNVYSYCHTLFVLSCCEFLTHDKICIES

>Tb1.NT.15_1 [222 - 302]

MKGDRMRKIGNLWIISTITFLCISTMC

>Tb1.NT.15_2 [257 - 349]

MDYKYHHISLHFHHVLNYTEMFLLITAELQI

>Tb1.NT.15_3 [747 - 851]

MYIFQKWKPVAIIFRYSIFMRDYFLSATTSLCLQL

>Tb1.NT.15_4 [638 - 862]

MSYSNTRNKIRQTLLILNCNMRSNTLPIWLHIPHVVYVYISKMETRCYHFQILNIYAGLFPIGNNEFMFAAMKFG

>Tb1.NT.15_5 [721 - 891]

MASYTSRSLCIYFKNGNPLLSFSDTQYLCGIISYRQQRVYVCSYEVWVNELHSSVSK

>Tb1.NT.15_6 [866 - 952]

MNCTHLSVNEFGCYFYVLFLIACSCRFVL

>Tb1.NT.15_7 [901 - 984]

MLFLCFIFNCMQLSLCVMNSRACQSYHQ

>Tb1.NT.15_8 [1118 - 1225]

MPIVTYVWNYVNYYLKPFGNLMEKEGNVDRIILLIF

>Tb1.NT.15_9 [1656 - 1742]

MVSSINCVNFIPIIFHMLYHHHHGRCVNE

>Tb1.NT.15_10 [1859 - 1954]

MFLFIMKVNGNTLWLSDAKRLSVFFTCPTDII

>Tb1.NT.15_11 [2017 - 2148]

MSLSMKLNIMNCKNGEDIQRSIFTVVQLCVYMYVLCRYSFSSFH

>Tb1.NT.15_12 [2057 - 2203]

MERIYKEAFLQLCSCVCICMCCADTLFLRFINIHKIFCLLFYLILINRI

>Tb1.NT.15_13 [2331 - 2423]

MIMKQGKYLFTAAIFWNCSSQYQIHYDIILY

>Tb1.NT.16_1 [151 - 252]

MHVKSKNNCHLNIISPNCFAYIISSKCLRNLGSE

>Tb1.NT.16_2 [296 - 394]

MRETLVGFLYIGKWSGFKVFYMMFSWRLKYFRK

>Tb1.NT.16_3 [410 - 538]

MIQESFRLLISSSLPRCTIFGLHCKKWYDICNRLFIIYFFEFI

>Tb1.NT.16_4 [492 - 578]

MIFATAYLLFIFSSLYKCGLVAWKILAYG

>Tb1.NT.17_1 [304 - 378]

MLMRDHYAEGRTADDCNLKPVICGL

>Tb1.NT.17_2 [219 - 437]

MKLQVEGIDSNHSSPNICNVNIFVERYLYVNERPLCRGKNSRRLQPKTRNLRTVINFNSFILLCKDGISKLYL

>Tb1.NT.17_3 [543 - 623]

MLCDINTRCFFFHIILLFFFLLVCRTI

>Tb1.NT.17_4 [415 - 630]

MASANYTCKLFEVKTTIRGRECAGGDLNRMFMRNLYCESTVEICCATLTPDVFFFILFFFSFFCLSVEQSEV

>Tb1.NT.17_5 [659 - 778]

MCCCLYNTNRYKLNICSKYKNIRFIATSCLISLYSNGTFF

>Tb1.NT.17_6 [990 - 1223]

MAHHYRVFCYEPIINHETGLYESYSLHKCNALKECNFIYLFITTIIIVSINIQVFIHHYFIIYIFFPTSVSRNMVDWK

>Tb1.NT.17_7 [1238 - 1336]

MVRETLICFDRADAHSSKGFRKVMGKFNHIFYY

>Tb1.NT.18_1 [164 - 241]

MFSAQFRLCTKFITTFLRLQCRCVKE

>Tb1.NT.18_2 [294 - 374]

MPLPPFFIFCFIKPTYLYCGKAIDISQ

>Tb1.NT.18_3 [566 - 670]

MSYLCFETAHGLELTVVAGVVPQVEYGLCWKSKAL

>Tb1.NT.18_4 [642 - 791]

MVYVGKAKHCNDMVKEDFLFLFFARGQPCQLCKNLQKTFHASDSVTINIL

>Tb1.NT.18_5 [866 - 961]

MKLLRAGGGGGQPHLSGIPLFPLQLFMSNLQV

>Tb1.NT.18_6 [1006 - 1092]

MPPDACRNAPLRNVFTTIHANYQNEEELC

>Tb1.NT.18_7 [1016 - 1114]

MPVEMRHFAMFSQQSMQTIRMKRNCVNFQREPP

>Tb1.NT.18_8 [1114 - 1242]

MMQHYTMSCATFCCAQRISIFLFIILFCNFYSINEGHRPKFDS

>Tb1.NT.18_9 [1214 - 1384]

MKGTGLNLTVNKLMSLFVCMCVSWSILIFVRYVISYLCTRFSYFDKCMCCPIELQDS

>Tb1.NT.18_10 [1413 - 1514]

MALMVLHNIRNTHTNLHIYTQNNFFRTQNMFPIF

>Tb1.NT.18_11 [1351 - 1602]

MHVLPNRTAGLVTHVHGVSTVWHLWCYTISVTHIQIYTYTHKIISFGLKICFPFFNSHSTPHKTYNGEGTFLCVVSSKLYLLIY

>Tb1.NT.19_1 [42 - 128]

MEVCYCYRLRVAETVLCCENKMCWTRQRL

>Tb1.NT.19_2 [17 - 190]

MSLQPHKVDGSLLLLSVESCRDCVMLRKQDVLDAPETLIFVGASFFLWVLRTYIYLFF

>Tb1.NT.19_3 [319 - 426]

MYLRTVGQKRFVILCAETEEAMRTCVGAHLFPCYFL

>Tb1.NT.19_4 [390 - 521]

MCGRTSFSLLFFMNTHFRYITFCNQLETSGLPLPCRRLTKALCL

>Tb1.NT.20_1 [158 - 232]

MNTGPYFFLFLPELMGSRRLDEGCP

>Tb1.NT.20_2 [839 - 949]

MKRAIPLFRLLPSLISPSAASCSQRFCVNKTRTILRF

>Tb1.NT.21_1 [128 - 376]

MFRSSMVVRSFTQESFYRMSKYIKSRNPNERYLRTGHIVLETLKRYHSYVLALVFTLGVTCYDQIKHPEKIRVPVHDGRSLVL

>Tb1.NT.21_2 [273 - 383]

MCWPWYLLWGSRATTKSSTQRKLEYPYTMVDHLFFST

>Tb1.NT.22_1 [143 - 256]

MLCPNPSASAQVASHRFRYLVPTQQEHRFNAPNAEWLV

>Tb1.NT.22_2 [46 - 312]

MHSSVCPHNSFTVATMMNRPPPQQAMRSAATKDVVPQPQCVCASCFSPLPLPRPDATGTPFQCPQCRMAGIDMKVLYTRRAFPTEHSTN

>Tb1.NT.22_3 [437 - 733]

MKVGCALAFVGMYVLLILETYPSPSYSFLASASLATRVLKEGDERCSYLQQFYLVSVVAYRAKFIFLHSLSGALSSPSVRDLGTLLFILVLSCEVRDRL

>Tb1.NT.22_4 [564 - 734]

MRGAHIFNSFTLCPWWLIVPNSFSCIPYLERSAARLYVIWAHSCLFLCYRVKSEIDC

>Tb1.NT.44_1 [68 - 343]

MAVIPYLGSQDFYIPFYDPNVQGRGVTISTYTEGNIVCSLSLSGVFLQNEESTCWKGMRGFSSSRRNCARFLVNTIFCVDSTSNPKCQPDGV

>Tb1.NT.44_2 [336 - 431]

MVFSPGYTCMWLGFPSHSLSCLCLFSAMIGYL

>Tb1.NT.44_3 [663 - 794]

MWRRGENEASGPIPYLFFTCWGSRMLTDWSWHLAGMLNGTGTEA

>Tb1.NT.44_4 [933 - 1010]

MCGCRPSRSAMRLAVVTGLVVFLCCH

>Tb1.NT.26_1 [2 - 196]

MYATCIFITVLFASFLLGRAVGRVNVYGIPLPKQLAKLLTCRRISSHFIISATSRGEGRKLTLVI

>Tb1.NT.26_2 [476 - 559]

MPTRSDLAGRIAYFEYGVMNIFFIVMLG

>Tb1.NT.26_3 [771 - 890]

MRSEKTDVGTVDLAFLLLFLARDSNGGIKLLLNHFFSKGK

>Tb1.NT.26_4 [941 - 1024]

MKQVVVTYIPFFFTFMPCKLIIVISDDL

>Tb1.NT.26_5 [1148 - 1228]

MNIQLIELTSFFDCFFFLIRVNALSFY

>Tb1.NT.26_6 [1340 - 1447]

MHKFLFFVFSFCLCNCKHLFIIIIILYLISYGTVTL

>Tb1.NT.26_7 [1672 - 1746]

MLFSSSPWAMSINGAFNTIPFNDFL

>Tb1.NT.26_8 [1736 - 1816]

MISFNISNILNESCTFVVGFIPNWALW

>Tb1.NT.26_9 [1892 - 1972]

MKTINLSILTVKLVHTLVNQMRCGGDN

>Tb1.NT.26_10 [2231 - 2380]

MFYYNCFVISICVKQAPVFLLWRTLKAEEFFTSPIKLNFSLFHYLFKKSD

>Tb1.NT.26_11 [2457 - 2594]

MVLLKNTCICCCCLFLYKLLSNITDILVYEVHSFRHQLLGVCVVCN

>Tb1.NT.26_12 [2542 - 2745]

MKFTPFVINCLEYVWSATDFFLPFICFVLSFLKSVPPKGMPIIYPSWSHVFLFFFLTSNLISSITVPL

>Tb1.NT.26_13 [2787 - 2888]

MTKSTKKERRRREKKMWPFYLNVSLFPCLLFHSL

>Tb1.NT.26_14 [2851 - 3036]

MLVSSLVFSSTAFEVFNHRRYFFILFFIRLEIFFIAVKIFCFSNVCPLLFLCNYVNALLSWA

>Tb1.NT.26_15 [3053 - 3199]

MTVYGNSCVIHLLEAPFPCFLFFGGRGVCERDYQVAPPKLLIIIIYICS

>Tb1.NT.26_16 [3362 - 3457]

MGLLPHKFSILIMYIVSFPNAFTHLTLGRISQ

>Tb1.NT.26_17 [3713 - 3814]

MRLSYSGAATDGMALCDLNVLSGSCIVLSVGFVW

>Tb1.NT.26_18 [3768 - 3857]

MYFQEAVLCYRWVLCGNARTRCFSRLRCCF

>Tb1.NT.28_1 [148 - 291]

MEGEKVSLLHIRIDLGPLVVWVNGQKRRSDNIRSNMLQKWKKKQGSGN

>Tb1.NT.28_2 [264 - 383]

MEKKAGKRELSWNLIGRKTVFVQQYRTLISVFTCFDGSFI

>Tb1.NT.29_1 [109 - 201]

MPLSVSVAYALFHSSTTRIRGRNLLVLQLLD

>Tb1.NT.29_2 [316 - 423]

MMPPFLMWRGKVYIIYYDGMPQGDAYLSKGDINWER

>Tb1.NT.29_3 [772 - 867]

MPVRKPNEYDPTTQLPQRMNWYHGNCHAVEED

>Tb1.NT.29_4 [714 - 875]

MSPAVSVCMGVSWHKSQFANACAEAQRIRPYYAAASAHELVSWQLPRGGRGLKA

>Tb1.NT.30_1 [109 - 201]

MPLSVSVAYALFHSSTTRIRGRNLLVLQLLD

>Tb1.NT.30_2 [316 - 423]

MMPPFLMWRGKVYIIYYDGMPQGDAYLSKGDINWER

>Tb1.NT.30_3 [714 - 845]

MSPAVSVCMGVSWHKSQFANACIIFANHSVVAYPHIMSCVATRM

>Tb1.NT.30_4 [944 - 1147]

MDYCRYLPNFPYICGGTLCRLIMVAVRTLWRQEGAVEIDSNVNFAELVDVPMRKYVFVYLCVRGFGVP

>Tb1.NT.30_5 [1065 - 1148]

MLTSQSWLMSRCASTCLCICVCEVLVCH

>Tb1.NT.31_1 [109 - 201]

MPLSVSVAYALFHSSTTRIRGRNLLVLQLLD

>Tb1.NT.31_2 [316 - 423]

MMPPFLMWRGKVYIIYYDGMPQGDAYLSKGDINWER

>Tb1.NT.31_3 [714 - 845]

MSPAVSVCMGVSWHKSQFANACIIFANHSVVAYPHIMSCVATRM

>Tb1.NT.31_4 [944 - 1147]

MDYCRYLPNFPYICGGTLCRLIMVAVRTLWRQEGAVEIDSNVNFAELVDVPMRKYVFVYLCVRGFGVP*

>Tb1.NT.31_5 [1065 - 1151]

MLTSQSWLMSRCASTCLCICVCEVLVCHS

>Tb1.NT.32_1 [814 - 888]

MMHFTKRRGDSGAGANGFQKEMFIK

>Tb1.NT.32_2 [1052 - 1225]

MNHITVKATKHVLVRSLRGGREGTSNTLHLANTRQRQECLSAPENDTEVKHGINSCNA

>Tb1.NT.32_3 [1367 - 1447]

MKLAGKTVLYIKAQLWECEMAHHPPWG

>Tb1.NT.32_4 [1461 - 1592]

MSPAVSVCMGVSWHKSQFANACIIFANHSVVAYPHIMSCVATRM

>Tb1.NT.32_5 [1691 - 1894]

MDYCRYLPNFPYICGGTLCRLVMVAVRTLWRQEGAVEIDSNVNFAELVDVPMRKYVFVYLCVRGFGVP

>Tb1.NT.32_6 [1812 - 1895]

MLTSQSWLMSRCASTCLCICVCEVLVCH

>Tb1.NT.33_1 [97 - 171]

MFLCRRGHKDVVPYLRVQYCRGIVK

>Tb1.NT.33_2 [207 - 299]

MNLAGHCLRIPFVSVSDKHFRFVFPVEYHHR

>Tb1.NT.33_3 [912 - 1034]

MVTRGVAEIGGVFTAVNADWKCYIKVSKDYFWSKKKRSFAK

>Tb1.NT.35_1 [22 - 126]

MCHFRISLPYKGDVRLGLQHIWLKQNKRSRVSFIA

>Tb1.NT.35_2 [84 - 227]

MAEAKQTKSCIIYRMKTHPSLSVLLKQRSAYVWAERGRIFPNCESLAH

>Tb1.NT.35_3 [175 - 252]

MCGLSGGGSFRIAKVWHTSRKGLQKQ

>Tb1.NT.35_4 [382 - 501]

MFDVEIHECILVGTVLKVGIRIHERNICVTPVVRYSSWKM

>Tb1.NT.35_5 [492 - 710]

MENVRQYDVIHVEKYLCCLPCLMFNGNHLLFLFYIRRVCSCFCFYTQIADLCFRSVFSASASASACACACAMG

>Tb1.NT.35_6 [511 - 852]

MTLYMWKNICAVCRALCLMETTCFFCFTSVESARVFVFTRKSLTCAFGVCLVQVQVQVPVRARVPWDRADKRRKMTTPTINSVLLFPFHPFHCISYKCIHICINVCMFLCEEGG

>Tb1.NT.35_7 [801 - 881]

MYPHMHKRMYVFVRGGWVNGKGRYIFQ

>Tb1.NT.35_8 [939 - 1037]

MHGCFGTIPKCFVAVCISQRVMQLRSHVLKVEK

>Tb1.NT.35_9 [1413 - 1526]

MFRSLFLTVLLCLLGVPVPRPVHCLEPFLLWSQFRLCI

>Tb1.NT.35_10 [1348 - 1788]

MLMCLCFSACGFALRGPFPAGKCFVHYFSPCCYVCLGFPSPARSIALNHFFCGRSFGCASKITNFLFHVGRHNLFCARLLSGIVWHMPPNTCTRMHFHAGYAFVSECAGVNLWEVCVFVCGFFVSVILSRKWYTALVWGKASDPPPI

>Tb1.NT.35_11 [1665 - 1832]

MCWGKFMGGVCVCVRFLCQCYFVEEMVYRACLGKGFGPPPHLSTCAFCCSGGSTEL

>Tb1.NT.36_1 [451 - 531]

MIMMITALAIIIIIIMIIIIIGAVTER

>Tb1.NT.36_2 [577 - 765]

MIFSFFFLLNCLLPAPLLLVFICHFYYYYYYYYYYCVSVFVFLCFFVFFIIRSLLLLLLPSSL

>Tb1.NT.36_3 [642 - 782]

MPLLLLLLLLLLLLCVCFCFFVFFCVFHYSLIIIIIITIIIVILIIW

>Tb1.NT.36_4 [842 - 949]

MRGRTEKRKRKKGQIHTHIFIYLYMHVCMKYLFNFI

>Tb1.NT.36_5 [832 - 1173]

MRANERENREKKKKKGTNTHTYIYIFIYACLHEIFVQFYLIRLFTNRYQISFLFFFFPPPFSPSYCCFYYYYYYCCCFVKISVQAAPTEERKNDMYICIYTCVYLYMFVCIICS

>Tb1.NT.36_6 [1173 - 1307]

MNNCFYFYLLHVRSSFFFFLLLFFNEYLCSCPRMYYCSRTNICKL

>Tb1.NT.36_7 [1204 - 1368]

MYALLFFFSCFYSSMNICVHVRECIIVHVQIFVNYEILDCFSFFVSNKKLYSFIL

>Tb1.NT.36_8 [1307 - 1558]

MKSLIVFLFLFQIRNYIVLYYNANNYIRTFFFLSFFLFFFKSLLLLGARSLLSVIIIIIFIYSYERYAIFKYLFISTERSFYGR

>Tb1.NT.36_9 [1497 - 1655]

MNVMQYLNIYLYQLKEVFMGGEKFPPLHLFLFFFFVLFRFVRLLLYHHCSFFF

>Tb1.NT.36_10 [1739 - 1861]

MKCKPATPPPPSKREKKTTNFVISPLSFLFLFFFSNNDDRY

>Tb1.NT.36_11 [1848 - 1943]

MMTDTERVFYYIVLLLLLLILYFLCFSSFFSF

>Tb1.NT.36_12 [2000 - 2134]

MHFGLYIIYIYIYFTYTLQYLNGIHFLFLLHVISLPFRFCYDYDY

>Tb1.NT.36_13 [1950 - 2171]

MRAGELPRPHEIINKYTCILVYILFIFIYTLLIHFSISMVFTFFFCFTLFHFLFVSAMIMIIEKKKKKKSKENE

>Tb1.NT.36_14 [2206 - 2400]

MKQRRALLLLLLLLLLLLFFFFLSFSVPASSKFFFVLCVCVCFCVFFFPLKLKRKEFKKKKEKKR

>Tb1.NT.36_15 [2505 - 2591]

MQIITMIKRNVLDIILNNINLLLLYHCWK

>Tb1.NT.36_16 [2594 - 2671]

MRLLSINVLMKTQMCEGTERKMREHT

>Tb1.NT.36_17 [2746 - 2934]

MQSLQLFQLCWKELLPSELRLLHKHIQTHTDIYLYIYTYTYILYYINIILYIIYIYIYIYTYV

>Tb1.NT.36_18 [2998 - 3078]

MKVERERKKKKKETSHAQSHIRTREMM

>Tb1.NT.36_19 [3139 - 3324]

MEVTGVTNTKREKLKEKKRNKNTQNYTNIWLYLNLYIFFFFLPPSGALFFFSFFFSYVCLCV

>Tb1.NT.36_20 [3318 - 3434]

MCLKGKRKVEMTVFEMGMSRDDEMKWKRKKKKIQEKMLR

>Tb1.NT.36_21 [3434 - 3598]

MRIINNNDGLKKKKKGADGSNKTKKKRSILYEATLSHTLTHSHTHMSFSSFVNFF

>Tb1.NT.36_22 [3486 - 3662]

MAQTKQKKKGAFCMRRLSLTHSHTHTHICPFHPLLIFSEISFAVIFFFHIFFCVYIYIF

>Tb1.NT.36_23 [3690 - 3965]

MAACTPLLRYHYGNLRHLLFPFYFPLLLLSLFIPSSGFNTFIIFIYIFPFSVYLPPFCVANISSFIYFSRCDIDSFLCTLFFFFFFFFVKLL

>Tb1.NT.36_24 [4389 - 4652]

MRKAKQKKKSKAKQKKMYEEEDDDNKCVWFTPLSHFLPEVSFFSFFLFDCFIAWSNFTSPLFCCFIFFFLIFLLLFLWCFSTFFFSSP

>Tb1.NT.37_1 [305 - 448]

MPWGWYGQQSWKSFTGSACFADKTFADIMFLRVCYPRDIEATGVRATK

>Tb1.NT.39_1 [56 - 187]

MKRAECHTNLYQKHFEYTSMNIKKRIRIGERMGSVAPWACRCVT

>Tb1.NT.39_2 [265 - 390]

MHMFSLWPCMFSVKFGHRSSFSKYPCLLPRCRGLCHGNLCLK

>Tb1.NT.47_1 [292 - 381]

MVFEYPLNESRVIGPHRMSVFELNKAFGDR

>Tb1.NT.47_2 [455 - 580]

MVTKENWMRRVTTLDLSDQAKLRGFQKAAKARLHFIFANANN

>Tb1.NT.47_3 [900 - 1001]

MSNYINARFVITIGRRMLKKRHRGAAQTEMNESH

>Tb1.NT.47_4 [991 - 1077]

MKVIDSDRRKSQSQLRERTHSFFFKATKY

>Tb1.NT.40_1 [565 - 642]

MLCVQHYHPYQWMGINGEIPGFLCIM

>Tb1.NT.40_2 [633 - 821]

MHHVSNCFPCLPSGNDFTTYLVSCLGKYSNISEGRLMRGMRDNTIHFWLNGIVMVHLSAMFTP

>Tb1.NT.41_1 [129 - 242]

MPSGRGMCLWSLTTPITLLWRQCLILRQRGKQYLDYHL

>Tb1.NT.41_2 [616 - 690]

MKGFEVSAPLRSMMVVVKCPKQMKP

>Tb1.NT.41_3 [669 - 839]

MSKTNEALEGVTATLLRTNGKELVLSSAAYFPEGFNAITGRLKQLSENLEPLVISKR

>Tb1.NT.41_4 [1032 - 1151]

MAENSFIYDHSCVALDAEAVNGMQGICTPHTLWVSVFVEE

>Tb1.NT.41_5 [1093 - 1212]

MACREYVRRILFGSVYLWKSESWLIICNEKQVSFRRTERW

>Tb1.NT.41_6 [1242 - 1340]

MQLFFWSRRERSVAARAPHHQTGRRTDSIRKRK

>Tb1.NT.41_7 [1225 - 1401]

MNSMELCSSFSGLVENDPWRHGHRTTKLGAEPTVFEKENKTILGLDEEYGFHLFKKEPA

>Tb1.NT.41_8 [1620 - 1712]

MATNTLFEPFARTKMTQVLWDLCRCGTAGRR

>Tb1.NT.41_9 [1960 - 2103]

MVAGTIVQFCSTIIYNQLRFKGDECDQTRLDRATASPHCSRGACQHST

>Tb1.NT.41_10 [2027 - 2146]

MNAIRLVWIERLLPHTVHEVRANIQLSELSPNSLHMSRLM

>Tb1.NT.41_11 [2594 - 2671]

MRNTVGLTKESVVNLQEQHTGRACVV

>Tb1.NT.41_12 [2928 - 3002]

MGEMLSKICRVISFSVKLEVMLCGR

>Tb1.NT.41_13 [2870 - 3043]

MQKENDAAEGKDQVKHETAYGGNVKQDLSCNFFFSKIGSYVVWTVSMICEEGGCAKAD

>Tb1.NT.41_14 [3049 - 3123]

MTAVVLRRAKPLLAQKIWQKNIVNS

>Tb1.NT.41_15 [3099 - 3230]

MAKEYRKFMNSTYNLKVCGKNSDGKATTSTVKDCNSQNHTTVMI

>Tb1.NT.41_16 [3783 - 3875]

MMTKHLVAKLTNRFARERSWVLAKSQSTFAG

>Tb1.NT.41_17 [3900 - 4022]

MCKAWKRCQEAIQTFCELANRETVIMKRVCGHRTKRKKDTR

>Tb1.NT.41_18 [4176 - 4310]

MDAFLEYQNRSKVERICAARKNWIIVHSGTALKIDWLFQCMLDKG

>Tb1.NT.41_19 [4034 - 4315]

MASHNEYLDIMEFNPKKARDLYVQTVSCDDAAHLSVQEYLQKALNEVNGRILGIPKQEQSGEDLCGEEELDYCALWHCLENRLVVSMYVRQGLT

>Tb1.NT.41_20 [4375 - 4545]

MWPRATRKDFDFAQNKRTELLTDFQFSTSTACSLMNTLSATRKSYHRKRLLGSALKG

>Tb1.NT.41_21 [4883 - 4969]

MNATQKKGKCRNNQPFSNSMRRIRGRKHI

>Tb1.NT.42_1 [399 - 509]

MLVNSDLGKTVEKSQHGDLSTHAGPDTVSRSALYRCA

>Tb1.NT.42_2 [1037 - 1114]

MQSQSSPLAFLRLLHGKIHESASTVE

>Tb1.NT.42_3 [1080 - 1226]

MGKYTNQPQRLNRRRSLWGRGFPRDPKQEALITAARSRGIENCWVHLDC

>Tb1.NT.42_4 [1450 - 1545]

MTQLTTMVKASLMPCQNVRNTEMRSLAVKQRV

>Tb1.NT.42_5 [1558 - 1725]

MCQARARTGCFKQCRYCPCTNRINCVDSIRDMVQLQVQTLATVKGFCFTVEPSLLL

>Tb1.NT.43_1 [40 - 126]

MMSTLPNFSNLSRIFFDCTVEKENLYVLR

>Tb1.NT.43_2 [512 - 721]

MYGYPHLALSYNVTLCHILDLLSRVMLASAGLCRLLHLGDLDGSYAYYDHGQSFFFPYYRTCWVISTVGT

>Tb1.NT.43_3 [975 - 1091]

MTRRIFLELVTNRRRCDTGISVKRRYRCKLSFRYFTAEK

>Tb2.NT.2_1 [281 - 430]

MEDQGHLCFCAHNILHMMSYSSLYLFVQVQLICLRYVWWCYLFELFIGCE

>Tb2.NT.2_2 [387 - 467]

MYGGVICLSCLLGVNNTFARDYTACVQ

>Tb2.NT.67_1 [60 - 173]

MDSRTFKLHKSTPIRNSKISNIMYLFEEKKRDEKKRCI

>Tb2.NT.67_2 [183 - 284]

MHVKSKNNCHLNIISPNCFDYIISSKCLRNLGSE

>Tb2.NT.67_3 [328 - 426]

MRETWVGFLYIGKWSGFKVFYMMFSWRLKYFRK

>Tb2.NT.67_4 [442 - 561]

MIQESFRLLISSGLPRRTIFGLHCKKWYDICNRLFLIYFL

>Tb2.NT.67_5 [531 - 611]

MQPLISYLFFVSLYKCGLVTWKILAYG

>Tb2.NT.67_6 [685 - 762]

MSYQMLNASTDSLFKPKHFLSFSTLS

>Tb2.NT.67_7 [1064 - 1138]

MLMRDHYAEGRTTDDCNLKPVICGL

>Tb2.NT.67_8 [1042 - 1197]

MFVERYSYVNERPLCRGKNNRRLQPKTRNLRTVINFNSFILLCKDGISRLHL

>Tb2.NT.67_9 [1175 - 1279]

MASADYTCKLFEVKTTIHGRECASGDLNRMFMRNL

>Tb2.NT.67_10 [1303 - 1383]

MLRDINSRCCFFHIILFFFFLLVCRTI

>Tb2.NT.67_11 [1419 - 1541]

MCFCLYNTNRYKLNICSKYIKNIRFVTTSCFISLYSNGTFF

>Tb2.NT.67_12 [1750 - 2001]

MAHHYRVVCHEFIINHETGFYESYSLHKCNALKECNFIYFLLLPFLLSVLIFRFSSITILLFIFSFQHLFQATWSIGNNVNING

>Tb2.NT.67_13 [1997 - 2167]

MVRETLICLERADAQSSKGFRKVMGKFNHIFLLLNYFSKMLFFFCVALTFKTNWAAL

>Tb2.NT.67_14 [2366 - 2551]

MSAIFILFILLILVMRIIVRIMIMIGVCCDCVYTNIIRVVVWNNWKPKIEALKTILRILYEK

>Tb2.NT.67_15 [2610 - 2705]

MEESKREEEKNVAVICVASPANMKIGIIHTTK

>Tb2.NT.67_16 [2654 - 2743]

MCSFTCKYEDWYYTHNEMTKEETKERNSPV

>Tb2.NT.67_17 [2868 - 2990]

MILDDKEDNPQICGIILKMDTVFSEMIAVAAALNATLMGVL

>Tb2.NT.69_1 [6 - 86]

MMLVKIHRGISKEAFMVPITCAPHNFP

>Tb2.NT.69_2 [450 - 614]

MRSDTLPIWLHIPHVVYVYISIMETRCYHFQILNIYAGLFPIGSSEFIFAAMKFG

>Tb2.NT.69_3 [473 - 643]

MASYTSCSLCIYFNNGNPLLSFSDTQYLCGIISYWQQRVYICSYEVWVNDLHSSVSK

>Tb2.NT.69_4 [643 - 735]

MNSDVIYIFIFNCMQLSLCVMDSRACQSYDQ

>Tb2.NT.69_5 [653 - 784]

MLFIFLFLIACSCRFVLWILEHVKVMISNFLHAYKSVIRHWKCN

>Tb2.NT.69_6 [980 - 1063]

MLSSITIVFSQFISTMTRCYISTCRCIM

>Tb2.NT.69_7 [1232 - 1306]

MCCFIFCYSNSFVYMSNRKLTMMIQ

>Tb2.NT.69_8 [1453 - 1539]

MLYNHHHKRCVNESNNDGTSDSCEFVGLN

>Tb2.NT.69_9 [1768 - 1902]

MSLFNASEYYVLTEVRKLQWNSCVVRIHVCIYPYVLCRYSISSLH

>Tb2.NT.69_10 [1850 - 1936]

MCVYIPMCCADTLFLRFINIHKFFVFCFT

>Tb2.NT.70_1 [60 - 143]

MLSNMLSKLYICVNVYYLYCDIWNPESI

>Tb2.NT.70_2 [193 - 273]

MKRDHVRKIGNLLIISTITFLCNSTMC

>Tb2.NT.70_3 [713 - 817]

MHIFQKWKPVAIIFRYSIFMRDYFLLAATSLCLQL

>Tb2.NT.70_4 [604 - 828]

MSYSNTRNKIRQTLLIVNCNMCSDTLPIWLHIPHVVYAYISKMETRCYHFQILNIYAGLFPIGSNEFMFTAMKFG

>Tb2.NT.70_5 [687 - 857]

MASYTSCSLCIYFKNGNPLLSFSDTQYLCGIISYWQQRVYVYSYEVWMNELHSSVSK

>Tb2.NT.70_6 [857 - 949]

MNSDVIYIFIFYCMQQSLCVMDSRACQSYHQ

>Tb2.NT.70_7 [832 - 975]

MNCTHLSVNEFGCYLYFYFLLHAAVALCYGFSSMSKLSSVTSFMHTKV

>Tb2.NT.70_8 [867 - 998]

MLFIFLFFIACSSRFVLWILEHVKVIISNFLHAYKSVIRHWKCN

>Tb2.NT.70_9 [1103 - 1189]

MGLCELLFKSIENLMEKEGNVDRIILLIF

>Tb2.NT.70_10 [1626 - 1730]

MGSSNNCINCISIIFHMLYHHHHGRCVNKSNNDGT

>Tb2.NT.70_11 [1829 - 1951]

MFLFIMKVNRNTLWLSDAKRLSVFFFYLSYRHYLIFSFGSK

>Tb2.NT.70_12 [2032 - 2124]

MGRINKEAIVWCVFMCVYIYVLCRYSFSSLH

>Tb2.NT.70_13 [2090 - 2179]

MCCADTLFLRFINIHKFFCFLFYLILIDRI

>Tb2.NT.71_1 [1084 - 1161]

MAKRNYTAPKKKRKMLYHTHTAGKPQ

>Tb2.NT.71_2 [1079 - 1231]

MRWQKEITQLQKKREKCYTTLTRQASLNSGFFFEGTFQRNSKAIPTESHDA

>Tb2.NT.71_3 [1050 - 1313]

MSSDLNYTQPCDGKKKLHSSKKKEKNVIPHSHGRQASIVDFFSREPSKEIQKRYPQSHTMRKYQLGKGYCQCEKSHREEPLEERGRSS

>Tb2.NT.71_4 [1367 - 1567]

MIEHPIKRRALLTRAAEVVAMEHDIDNILLIQLGKHIYTIELPRGTVWYLRCVTASVSKPVLLMFLM

>Tb2.NT.71_5 [1587 - 1733]

MCGLSVREAGTYNGEYLPKLSIAQHSLTLTRGNEGDLTGREKKKTSPLQ

>Tb2.NT.71_6 [1840 - 2046]

MMQKERKRESIKEGVRIAHCRKVCPNNEAAAYHWPSFRETYDRMGCVPLFVMEETQIWKKVLVYCMTKG

>Tb2.NT.71_7 [2259 - 2339]

MDSWNFTHHHKSFFRTCTSREAAAGDP

>Tb2.NT.71_8 [2361 - 2480]

MDMQHIYQCEAHDVVKCRGNKIVTNMEECGAQPLLVTRKR

>Tb2.NT.71_9 [2408 - 2515]

MSWKQNSYEHGGMWGTTIIGDSQKMTQQCDFIGVLV

>Tb2.NT.71_10 [2521 - 2607]

MKSRQYGWDNESDTAIPSIPCYVKKSTIE

>Tb2.NT.71_11 [2611 - 2739]

METVVLRMMYVAAGVRKNNSFLLTTTTATYFFNENLHYEWFWT

>Tb2.NT.71_12 [2708 - 2827]

MKTCIMNGFGLRSAEYYFVRLYFSKHTCTPRLFTQLSARE

>Tb2.NT.71_13 [2727 - 2885]

MVLDLGVQSIILYGYISLNTPVLPVYLRSCLQESEIFRKPTQHNPLRPAQETS

>Tb2.NT.71_14 [2952 - 3077]

MMRKPYCLFSMIIISYIFLVGSKKGKVRTKLFIIFYYCCGEL

>Tb2.NT.71_15 [3281 - 3355]

MIPSLHPKLTLLYRLTLNGWKDVHA

>Tb2.NT.71_16 [3337 - 3435]

MERCSCLMFKRFCMSPLIIYMYKYIYIFMNWKK

>Tb2.NT.71_17 [3933 - 4037]

MLSAVVLYMYIYMYRNKITCEKSCVIFLDFYSSTF

>Tb2.NT.71_18 [4052 - 4150]

MHIFCLCCAWFVTITFHHFYTHIHTYICIFELY

>Tb2.NT.71_19 [4526 - 4636]

MLSNIMSKLYICVNVYIICVVTYGILKAYERNCSNSL

>Tb2.NT.71_20 [4697 - 4792]

MDYKYHHISVQFHHVLNYTEKFLLITTELRIW

>Tb2.NT.71_21 [4789 - 4881]

MVKEISQRYCPNGMFVCLFVENSFVMMYLAN

>Tb2.NT.71_22 [5069 - 5161]

MILMSYSNTRNKIRQTLLIVNCNMRSDTLPI

>Tb2.NT.71_23 [5187 - 5291]

MHIFQKWKPVAIIFRYSIFMRDYFLLAAASLYLQL

>Tb2.NT.71_24 [5204 - 5302]

METRCYHFQILNIYAGLFLIGSSEFIFAAMKFG

>Tb2.NT.71_25 [5161 - 5331]

MTSYTSCSLCIYFKNGNPLLSFSDTQYLCGIISYWQQRVYICSYEVWVNELHSSVSK

>Tb2.NT.71_26 [5306 - 5425]

MNCTHLSVNEFGCYLYLYFFICMQQSLCVMNSRACQSYHL

>Tb2.NT.71_27 [5341 - 5451]

MLFIFIFFYLHAAVALCYEFSSMSKLSSVTSFMHIKV

>Tb2.NT.71_28 [5839 - 5925]

MIVCSEWELLFELSSKQKCLFVLSYLICA

>Tb2.NT.71_29 [5892 - 5972]

MSIRTVIPYLCLSCCEFLTHDKICIEF

>Tb2.NT.72_1 [435 - 533]

METRCYHFQILNIYAGLFPIGSSEFIFAAMKFG

>Tb2.NT.72_2 [392 - 562]

MASYTSCSLCIYLNNGNPLLSFSDTQYLCGIISYWQQRVYICSYEVWVNELHSSVSK

>Tb2.NT.72_3 [562 - 654]

MNSDVIFIFIFYCMQQSLCVMNSRACQSYHQ

>Tb2.NT.72_4 [537 - 680]

MNCTHLSVNEFGCYFYFYFLLHAAVALCYEFSSMSKLSSVTSFMHIKV

>Tb2.NT.72_5 [1528 - 1647]

MFLFIMKVNRNTLWLSDAKRLSVFFYLSYRHYLIFSFGSK

>Tb2.NT.72_6 [1686 - 1826]

MSLFNASECYVLTEVRKLQWNSCVVVWCVFMCVYIYVLCRYSFSSLH

>Tb2.NT.72_7 [1709 - 1873]

MLRIDRSEETPVEQLCSCVVCIHVCVYLCVVPILFFFASLISINFLFIVLLDFNQ

>Tb2.NT.72_8 [1920 - 2000]

MQVYEAVIFFVCMLFIVKEMMYEVHYI

>Tb2.NT.73_1 [17 - 91]

MLSNIMSKFVNIYNLYCDIWNPESI

>Tb2.NT.73_2 [141 - 221]

MKGDHVRKIGNLLIISTITFLCNSTMC

>Tb2.NT.73_3 [652 - 765]

MYFMHIFQKWKPVAIIFRYSIFMRDFFLLAASSLCLQL

>Tb2.NT.73_4 [552 - 776]

MSYINTRNKIRQTLLIVNCNMRSDTLPIWLHIPHVFYAYISKMETRCYHFQILNIYAGFFPIGSIEFMFTAMKFG

>Tb2.NT.73_5 [635 - 805]

MASYTSCILCIYFKNGNPLLSFSDTQYLCGIFSYWQHRVYVYSYEVWVNELHSSVSK

>Tb2.NT.73_6 [780 - 890]

MNCTHLSVNEFGCYLYFYFLLHAAAALCYEFSSMSKL

>Tb2.NT.73_7 [805 - 897]

MNSDVIYIFIFYCMQLLLCVMNSRVCQSYDQ

>Tb2.NT.73_8 [1028 - 1135]

MPVITYVWNYVNYYLKPFGNLMEKEGNVDRIILLMF

>Tb2.NT.73_9 [1142 - 1225]

MLSSITIVFRQFISTMTHCYISTCRCIM

>Tb2.NT.73_10 [1299 - 1421]

MRDVFHFIICLFTLILSITFIYLLYIVSSCLDVLFHFLLFQ

>Tb2.NT.73_11 [1250 - 1435]

MCNCFVIRQLLNIYKENARRVSFYYLSVHAHSLYYLYLFIIYCFKLFRCVVSFFVIPITLCM

>Tb2.NT.73_12 [1393 - 1467]

MCCFIFCYSNNFVYVSNRKLTMMIQ

>Tb2.NT.73_13 [1766 - 1885]

MMFLFIMKVNGNTLWLSDAKRLSVSFLPVLQTLFNILLWK

>Tb2.NT.73_14 [2020 - 2094]

MCCADTLFLRFINIRIYIFIVYCFT

>Tb2.NT.73_15 [1952 - 2104]

MLRIVKMGRIYNCVVRIHVCVYVCVVPILFFFASLISVYIFLLFIVLLDFN

>Tb2.NT.73_16 [2154 - 2234]

MQVYEAVIFFVCMLFIVKEMMYEVHYI

>Tb2.NT.74_1 [226 - 306]

MKRDRMRKIGNLWIISTITFLCNSTMC

>Tb2.NT.74_2 [261 - 353]

MDYKYHHISLQFHHVLNYTEKCILITTELQI

>Tb2.NT.74_3 [730 - 867]

MASYTSCSLCIYFNNGNPLLSFSDTQYLCGIISYWQQRVYICSYEV

>Tb2.NT.74_4 [638 - 871]

MILMSYSNTRNKIRQTLLIVNCNMRSDTLPIWLHIPHVVYVYISIMETRCYHFQILNIYAGLFPIGSSEFIFAAMKFE

>Tb2.NT.74_5 [875 - 1018]

MDCTHLSVNKFGCYLYLYFLLHAAAALCYGFSSMSKLSSVTSFMHIKV

>Tb2.NT.74_6 [910 - 1041]

MLFIFIFFIACSCCFVLWILEHVKVIISNFLHAYKSVIRRWKCN

>Tb2.NT.74_7 [1237 - 1320]

MLSSITIVFRQFISTMTHCYISTCRCIM

>Tb2.NT.74_8 [1394 - 1525]

MRDVFHFIICQFTLILFITFICYTFSSSLDVLFCVFVIPITLCM

>Tb2.NT.74_9 [1704 - 1790]

MLYNHHHKRCVNGFNNDGTSDTCEFVSLN

>Tb2.NT.74_10 [1859 - 1978]

MFLFIMKVNRNTLWLSDAKRLSVFFYLSYRHYLIFSFGSK

>Tb2.NT.74_11 [2018 - 2092]

MSLFNASECYVLTEVRKLQWNSCVV

>Tb2.NT.74_12 [2121 - 2201]

MCCADTLFLRFINIHIFLLFIVLLDFN

>Tb2.NT.74_13 [2041 - 2211]

MLRIDRSEETPVEQLCSVVRIRVCVYLCVVPILYFFVSLISIYFYCLLFYLILINRI

>Tb2.NT.75_1 [344 - 460]

MHLKDTSSWAVSGYVLFPLCEFGVSWAANIRRVVITCVC

>Tb2.NT.75_2 [567 - 641]

MLSVSSSGFNCGIEAADIVSSVVCS

>Tb2.NT.75_3 [944 - 1051]

MKSEEVKKIKKILSRLWYMLHNILLLSKEAVIRYNP

>Tb2.NT.76_1 [178 - 279]

MHVKSKNNCHLNITSPNCFAYIISSKFLRNLGSE

>Tb2.NT.76_2 [323 - 409]

MRETLVGFLYIGKWSGFKVFYMMFSWRLK

>Tb2.NT.76_3 [437 - 583]

MIQEFFRLLISSALPRRTIFGLHCKKWYDICNRLFLIYLFFRVYISVVW

>Tb2.NT.76_4 [703 - 876]

MLQLTHSSNQNTFSAFPLSAKNVVQFPNSTPHLIHHRVKRYFLYGQHESGEMQKVEIK

>Tb2.NT.76_5 [1069 - 1143]

MLIRDHYAEGRTTDDCNLKPVIFGL

>Tb2.NT.76_6 [1180 - 1392]

MASADYTCKLFEVKTTIHGRECASGDLNCMFMRNLYCESTVEICCAILTADFFSSYYSSFLFFFLLVCRTI

>Tb2.NT.76_7 [1308 - 1403]

MLRDINSRFFFFILFFFSFLFFACLSNNLRFS

>Tb2.NT.76_8 [1428 - 1550]

MCFCLYNTNRYKLNICSKYIKNIRFIATSCFISLYSNGTFF

>Tb2.NT.76_9 [1565 - 1699]

MFLSLFHLFAHLILCARCCIYLLLHTHKKKIYIYIYHFETNRGCD

>Tb2.NT.76_10 [1913 - 2029]

MIVSINIQVFIHHYFIFDIFFQHLFQATWSIGNNVDING

>Tb2.NT.76_11 [2025 - 2105]

MVRETLICLDRADAQSSKGFRKVMGEV

>Tb2.NT.77_1 [38 - 130]

MTQIKGSFYRSHHFHVCNSNRVIVTIFFLRV

>Tb2.NT.77_2 [183 - 428]

MQGALSGEGGADSNIQNNLFSHMAERTEAVVKHTLQSEKSISPGADTLQRCEHKTLHLRTGGFSLFICLFVFESIPHISVQN

>Tb2.NT.77_3 [498 - 575]

MIGTYWEPAVNKRNTKEHNSDKHILP

>Tb2.NT.77_4 [635 - 739]

MARVLQSDKMNKIPNLVDQLMYLKIESHAVWSLEA

>Tb2.NT.77_5 [724 - 831]

MVLRGMKIDSITLRCDSVEASVNQHALKVGEMSVNI

>Tb2.NT.78_1 [1036 - 1257]

MAKRNYTAPKKEEYVIPHSHGRQASIVDFGGNLPKKFKSDTHRVARGIIINWEKDIASVRKVIGRNLWRKEEKS

>Tb2.NT.78_2 [1014 - 1385]

MNCIQPFDGKKKLHSSQKRGICYTTLTRQASLNSGFWGEPSKEIQKRYPQSRTRHNYQLGKGHCQCEKSHREEPLEERGKIIAITTKGKSDTILKRPHVMIEHSIKRRALLTRGAAVVAMEARH

>Tb2.NT.78_3 [1408 - 1512]

MGKHIYTMELQRSTVRYLRYVTAAVSKSILLMFLM

>Tb2.NT.78_4 [1532 - 1648]

MCSLSVREAGTYNGEYLPKLSIAQHSLTLIRGNEGDITG

>Tb2.NT.78_5 [1648 - 1743]

MRKKKDFTSPIGNECGNNFLFVDTTEDDASSE

>Tb2.NT.78_6 [1685 - 1834]

MNVGIISFSWIRQRMMQAASKQFDEVWSIMNSTDDVEKEKEREKVSNREI

>Tb2.NT.78_7 [1785 - 1940]

MMWRKRKKERKYQTGKSELHIVEKYAQIIKQPRIIGTVSKRHTTGWVMCHSL

>Tb2.NT.78_8 [1919 - 1996]

MGYVPFFVMEEKQNWKKALVYCMTKG

>Tb2.NT.78_9 [2209 - 2289]

MDSWSFTHHHKGLFRRRRCREAAAIDP

>Tb2.NT.78_10 [2311 - 2418]

MDMQHIYQCEAHDVVKCRGNKIVTNMEECEAHYYHW

>Tb2.NT.78_11 [2473 - 2652]

MKSRQYGWDNESDTTIPSIPCNMKKNYYRINGNCCIMHDVRGSRSEKTIAFSLIILLLFF

>Tb2.NT.78_12 [2686 - 2811]

MVPMRPNDLTVPPHTDTHIHTYALQLTQKWMCILVKKTPVST

>Tb2.NT.78_13 [2849 - 2929]

MLPCKGDGSGFIIYQFGREVLNCKCYL

>Tb2.NT.78_14 [2772 - 2948]

MDVHFSKENTSEHVASCSNCGHDGNGCFHARVMGVVLLFINSEEKFLTASATCETAVHF

>Tb2.NT.78_15 [2963 - 3178]

MKEIEESLTCMSSQHKYDAYYFFKKYGGNYNTHSNAMIHEAVAIDGECCKMEGLILHGVLRDLRRITPWVSK

>Tb2.NT.78_16 [3166 - 3255]

MGVQIASKGERGKRVKDTFRHFRFVSNRGG

>Tb2.NT.79_1 [85 - 159]

MLSNIMCKFVNIYYLYCDLWNPESI

>Tb2.NT.79_2 [209 - 289]

MKRDHVRKIGNLWIISTITFLCNSTMC

>Tb2.NT.79_3 [735 - 839]

MYIFRKWKPVAIIFRYSIFMRDYFLLATTSLCLQL

>Tb2.NT.79_4 [617 - 850]

MILMSYSNTRNKIRQTLLIVNCNMRSDTLPIWLHIPHVVYVYISKMETRCYHFQILNIYARLFSIGNNEFMFAAMKFG

>Tb2.NT.79_5 [709 - 879]

MASYTSCSLCIYFENGNPLLSFSDTQYLCAIIFYWQQRVYVCSYEVWMNGLHSSVSK

>Tb2.NT.79_6 [854 - 964]

MDCTHLSVNEFGCYLYLYFLLHAAAALCYGFSSMSKL

>Tb2.NT.79_7 [879 - 971]

MNSDVIYIYIFYCMQLLLCVMDSRACQSYDQ

>Tb2.NT.79_8 [889 - 1020]

MLFIFIFFIACSCCFVLWILEHVKVMISNFLHAYKSVIRHWKCN

>Tb2.NT.79_9 [1123 - 1209]

MRLCELLFETIENLMEKEGNVDRIILLIF

>Tb2.NT.79_10 [1216 - 1299]

MLSSITIVFRQFISTMTRCYISTCRCIM

>Tb2.NT.79_11 [1378 - 1509]

MRDVFHFIICQFTLILFITFICYIFLSCLNVLFYFFVIPIALCM

>Tb2.NT.79_12 [1688 - 1774]

MLYHRYHGRCVNESNNDGTSDTRECVGMN

>Tb2.NT.79_13 [2101 - 2190]

MCCADTLFLRFINIHIYFCLLFYLILIDRI

>Tb2.NT.80_1 [267 - 344]

MLTDCSCRVTLETLPGDHGIILKMFG

>Tb2.NT.80_2 [501 - 707]

MKCRNLSSIAVIDKCVLIKLNGRWCLCICWVARLPETWIKATKGVVVCFYPCICSNYLCINAYHHCLSV

>Tb2.NT.80_3 [562 - 780]

MAGGVYAYVGLLDSQKPGLKQQKVLSFVFIHAFVQTICASMRTIIVFQYNQPFYTINNNSLPSIIAKQDNFLD

>Tb2.NT.80_4 [653 - 781]

MHLFKLSVHQCVPSLSFSITNLSIPSITTLFHPSLLNKITFLM

>Tb2.NT.81_1 [48 - 182]

MTQIKGSFYRSHHFHVCNSKRVIVTIYFFARVKCEILMRTQNLLQ

>Tb2.NT.81_2 [194 - 337]

MQGALSGEGGADSNIQNNLFSHMAERTEAVVKHTLQSEKSISPGADTL

>Tb2.NT.81_3 [670 - 747]

MNKIPNVVHQLIYLKIESHAVHSLEA

>Tb2.NT.81_4 [747 - 839]

MKIDSITLRCDSVEASVYQHALKVGEMSVNT

>Tb2.NT.82_1 [5 - 163]

MQEGRCKFKKLSFEVPNMSTSVRPSLTETTNLHRVVSLSGSFRTGGKRCEWTK

>Tb2.NT.82_2 [591 - 674]

MIIIFIAVPTKSCPYIMTLVSGANRIAK

>Tb2.NT.82_3 [1020 - 1148]

MNCIQPCDGKKKLHSSQKKRKMLCHTDTKRKPQQWIFFWEGTF

>Tb2.NT.82_4 [1042 - 1272]

MAKRNYTAPKKREKCCATLTRKESLNSGFFFGREPSKEIQKRYPHTHTMRNYQLGKEHCQCEKSHREEPREERGRSS

>Tb2.NT.82_5 [1325 - 1525]

MIEHSIKRRALLTRAAAVVAIEHDIDNILLIEFGKHIYTMELPRGTVWYLRCVTASVSKSVLLMFLM

>Tb2.NT.82_6 [1477 - 1560]

MCDSFRKQVGSFNVFDVRYNWKECAAPV

>Tb2.NT.82_7 [1545 - 1649]

MCGPSVREAGTYNGEYLPKLSIAQHSLTLTRGKQR

>Tb2.NT.82_8 [1744 - 1836]

MMQAASKRFDEGWSIKNSTDDVERESIKKGI

>Tb2.NT.82_9 [1772 - 1999]

MRVGALRIAQMMWKEKVSKKESELHIVKKEVPNNEAIAYHWPSFRETYDRMSCAPFFVMEEKQNWKKVLIYCMTKG

>Tb2.NT.82_10 [2361 - 2468]

MSWKQNSYQHEEMWGTTIIGDSQKMTQQCDFIGVLV

>Tb2.NT.82_11 [2474 - 2560]

MKSRQYCWDNESYTTIPSIPCYVKKSTIE

>Tb2.NT.82_12 [2564 - 2707]

MKTVVSRMMYAAAGVRKNNSFLPTTIATYFLNGNIIFVMMQYGPDATQ

>Tb2.NT.82_13 [2697 - 2813]

MRPNNLTVLPHTDTHTHTYALQLTQKWMCILVKKKPVSK

>Tb2.NT.82_14 [2774 - 2884]

MDVHFSKEKTSEQVASCSNCGHDGNGCFHARVMGVVL

>Tb2.NT.82_15 [2851 - 2931]

MLPCKGDGSGFIIPQFGKEVLNCKCYL

>Tb2.NT.82_16 [3131 - 3235]

MELRDLRKINPRVSKEQAKGSAGGGLKTFSDTSAS

>Tb2.NT.82_17 [3254 - 3334]

MYVSINFVYDMSYISVKLVCTKALFII

>Tb2.NT.83_1 [98 - 202]

METFLHICRYLHFAMGNNNNRTCTHLQFYKARAHS

>Tb2.NT.83_2 [36 - 251]

MLSICSLLSLRENYLVHHKHLWRRFSTSAGIFILQWETIITEHARIYSSTRPELTLDYWMNIYNNIKKIFSR

>Tb2.NT.83_3 [251 - 433]

MTMQEILCYTFMEGQGHLCFCAHNILHMMSHSSFHLFVKVQLMCLRYVCWCYLFELFIGCE

>Tb2.NT.83_4 [390 - 470]

MYAGVICLSCLLGVNNTFARDYTACVH

>Tb2.NT.84_1 [8 - 88]

MMLVKSHRGISKEAFMVPITCAPHNFP

>Tb2.NT.84_2 [486 - 590]

MHIFQKWKPVAIIFRYSIFMRDHFLLAAASLFLQL

>Tb2.NT.84_3 [368 - 601]

MILMSYINTRNKIRQTLLIVNCNMRSDTLPIWLHIPHVVYAYISKMETRCYHFQILNIYAGSFPIGSSEFISAAMKFG

>Tb2.NT.84_4 [460 - 630]

MASYTSCSLCIYFKNGNPLLSFSDTQYLCGIISYWQQRVYFCSYEVWVNDLHSSVSK

>Tb2.NT.84_5 [630 - 722]

MNSDVIYIFIFYCMQQSLCVMDSRACQSYHQ

>Tb2.NT.84_6 [605 - 748]

MTCTHLSVNEFGCYLYFYFLLHAAVALCYGFSSMSKLSSVTSFMHIKV

>Tb2.NT.84_7 [640 - 771]

MLFIFLFFIACSSRFVLWILEHVKVIISNFLHAYKSVIRHWKCN

>Tb2.NT.84_8 [1741 - 1875]

MSLFNASECYVLTEVRKLQWNSCVVRIHVCVYPYVLCRYSISSFH

>Tb2.NT.84_9 [1764 - 1910]

MLRIDRSEETPVEQLCSAYSCVCISLCVVPILYFFVSLISINFIVYCFT

>Tb2.NT.84_10 [1823 - 1930]

MCVYIPMCCADTLFLRFINIHKFYCLLFYLILIDRI

>Tb2.NT.85_1 [85 - 195]

MLSNIMCKLCKSKYIYIICIVTYGILKAYERNCSNSL

>Tb2.NT.85_2 [220 - 300]

MKGDRVRKIENLLIISTITFLCNSTMC

>Tb2.NT.85_3 [745 - 849]

MNIFQKWKPVAIIFRYSIFMRDYFLLAASSLCLQL

>Tb2.NT.85_4 [627 - 860]

MILMSYSNTRNKIRQTLLIVNCNMRSDTLPIWLHIPHVVYEYISKMETRCYHFQILNIYAGLFPIGSIEFMFTAMKFG

>Tb2.NT.85_5 [889 - 981]

MNSDVIFIFIFYCMQLSLCVMDSRACQSYHQ

>Tb2.NT.85_6 [864 - 1007]

MNCTHLSVNEFGCYFYFYFLLHAAVALCYGFSSMSKLSSVTSFMHIKV

>Tb2.NT.85_7 [899 - 1030]

MLFLFLFFIACSCRFVLWILEHVKVIISNFLHAYKSVIRHWKCN

>Tb2.NT.85_8 [1133 - 1219]

MGLCELLFETIGNLMEEEGNVDRIIFLIF

>Tb2.NT.85_9 [1397 - 1552]

MFHFIICQFTLILFITFICYIFFQVVSMSCFIFCYYNNFVYMSNRKLTMMIQ

>Tb2.NT.85_10 [1632 - 1730]

MCYDNLLRFPQLIVLILFQLFTICCIIAIMEDV

>Tb2.NT.85_11 [1700 - 1786]

MLYHRYHGRCVNGFNTHGTSDSCEYVGLN

>Tb2.NT.85_12 [1852 - 1950]

MMFLFIIKVNRNTFWLSDAKRLSVFFTCPTDII

>Tb2.NT.85_13 [1904 - 1993]

MQKDYQFFLPVLQTLFNILLWKSVITRGRG

>Tb2.NT.86_1 [182 - 283]

MHVKSKNNCHLNITSPNCFAYITSSKCLRNLGSE

>Tb2.NT.86_2 [442 - 603]

MIQEFFRLLISSALPRRTIFGLHCKKWYDICNRLFLIYLFLRVYISVVWWRGKF

>Tb2.NT.86_3 [1066 - 1140]

MLMRDHYAEGRTTDDCNLKPVICEL

>Tb2.NT.86_4 [981 - 1199]

MKLQMEGVDSNHSSPNICNMNIFVERYSYVNERPLCRGKNNRRLQPKTRNLRTVINFNSFILLCKDGISRLYL

>Tb2.NT.86_5 [1305 - 1397]

MLRDINSRCFFFHIILLFFSFFCLFVKQSEV

>Tb2.NT.86_6 [1177 - 1440]

MASADYTCKLFEVKTTIHERECASGDLNRMFMRNLYCESTVEICCATLTADVFFFILFFFSFLFFACLSNNLRFSCYIPEFHHMLLFI

>Tb2.NT.86_7 [1561 - 1695]

MFLSLFYLFAHLILCARCCIYLLLHARKKKIYIYIYHFETNRGCD

>Tb2.NT.86_8 [2015 - 2185]

MVGETLICFERADAQSSKGFKKVMGKFNHIFLLLNYFSQMLFFFCIALTFKTNWEAL

>Tb2.NT.86_9 [2283 - 2360]

MNVCSIIIIIKCQVLLLSVSNSESVF

>Tb2.NT.86_10 [2434 - 2526]

MTQIKGSFYRTHHFHVCNSKRVIVTIFLLRV

>Tb2.NT.86_11 [2645 - 2893]

MAERTETVVKHTLQSEKSISPGADTLERCEHKPLIYEQEALVCLFVCLFLNPFLTSASNIKYREFRTFIMIIIAVSCKMTHIL

>Tb2.NT.86_12 [2893 - 2970]

MIGTYWAPAANKRNTKEHNSDRHILP

>Tb2.NT.86_13 [3011 - 3118]

MFANSENKEKRQSDEMNKIPNVVHQLMYLKIESHAV

>Tb2.NT.86_14 [3133 - 3225]

MKIDSITLRRDSNEACVYQHALKAGEMSVNT

>Tb2.NT.86_15 [3177 - 3341]

MRLSTCVEGGGNVSEYLRKRQNYFFLMATKCCSRALWKYFHWIEKCVRKGYRTLL

>Tb2.NT.87_1 [123 - 197]

MAYILFLHSIFHVITSNHIEFRSAE

>Tb2.NT.87_2 [157 - 339]

MSLLQITLSSEAPSEKKKRHVVGALSPIIDEMMMLNCQPVTSPTVTHSLSFLIENKKKVTC

>Tb2.NT.87_3 [346 - 435]

MLSNQYHSFFLPLLFNRWNLPKQLIMILDS

>Tb2.NT.87_4 [497 - 592]

MISNQWNMSSRANVKKTEWIRLYIFIYIFVLW

>Tb2.NT.87_5 [671 - 775]

MNNHVCKQYVFPALCIGVITDYTKLINLFDAWSLR

>Tb2.NT.89_1 [123 - 197]

MAYILFLHSIFHVITSNHIEFRSAE

>Tb2.NT.89_2 [157 - 339]

MSLLQITLSSEAPSEKKKRHVVGALSPIIDEMMMLNCQPVTSPTVTHSLSFLIENKKKVTC

>Tb2.NT.89_3 [346 - 435]

MLSNQYHSFFLPLLFNRWNLPKQLIMILDS

>Tb2.NT.89_4 [497 - 592]

MISNQWNMSSRANVKKTEWIRLYIFIYIFVLW

>Tb2.NT.89_5 [671 - 775]

MNNHVCKQYVFPALCIGVITDYTKLINLFDAWSLR

>Tb2.NT.90_1 [133 - 213]

MKMYRLLLLLLSLHKYGRELKGGPTGR

>Tb2.NT.90_2 [39 - 260]

MSRANSPAAPQGNNENQQVAGNIEGPMRRPRDENVPPPPAAAQPPQIRQRTEGGPNWTMNSEVEDVLLQRYAAL

>Tb2.NT.90_3 [232 - 369]

MYCCNAMRLCNFFAVNPLNINYFPYHIIGKFVPMFEVTSQILLHLK

>Tb2.NT.90_4 [538 - 621]

MSNLNQRIRESCFLQHLQHTLWSLIPSK

>Tb2.NT.90_5 [621 - 695]

MRGPVTPLPTQLVHPHATILQCHSS

>Tb2.NT.90_6 [667 - 741]

MRQYFNVTLLETISSEGTVRQQLRY

>Tb2.NT.90_7 [1725 - 1874]

MEDQGHLCFCSHNILHMMSHSSLYLFVQVQLMCLRYVCWCYSFELFIGCE

>Tb2.NT.90_8 [1831 - 1911]

MYAGVIRLSCLLGVNNTFARDYTACVH

>Tb2.NT.88_1 [5 - 163]

MQEGRCKFKKLSFEVPNMSTSVHPSLTEITNLHRVVSLSGSFRTGGKRCECTK

>Tb2.NT.88_2 [1023 - 1241]

MAKRNYTAPKKEQNVIPHSHEKQASIVDFFGNLPKKFKSDTHILTRCVIINWEKDIASVRKVIGRNPVEREKS

>Tb2.NT.88_3 [1001 - 1495]

MNYIQPFDGKKKLHSSKKRAKCYTTLTRKASLNSGFFWEPSKEIQKRYPHTHTMRNYQLGKGYCQCEKSHREEPSGKGKIIAITTKGKSDTILKRPHVMIEHPIKRRALLTRAAAVVAMEHNIDNILLIQLGKHIYTMELPQSTVRYFKCVTAAVSKSILLMFLM

>Tb2.NT.88_4 [1447 - 1530]

MCDSCRQQVDSFNVFDVRYNWKKCAASV

>Tb2.NT.88_5 [1515 - 1631]

MCSLSVREAGTYNREYLSKLLRAQHSLTLTRENKGDITG

>Tb2.NT.88_6 [1709 - 1801]

MMQAASKQFDEVWRTMNSTDDVERESIKQGV

>Tb2.NT.88_7 [1886 - 1963]

MGCAPFFVMEEKQNWKKALIYCMTKG

>Tb2.NT.88_8 [2436 - 2522]

MKSRQYCWDNESDTTITSIPCYMKKTTIE

>Tb2.NT.88_9 [2526 - 2654]

METVVSRMMYVAAGVRKNNSFPLNTTTATYFFNENLHYEWFWT

>Tb2.NT.88_10 [2623 - 2742]

MKTCITNGFGLRSAEYYFVRLYFSKHTCTPRLFTQLSARE

>Tb2.NT.88_11 [2642 - 2800]

MVLDLGVQSIILYGYISLNTPVLPVYLHSCLQESEIFRKPTQHNSLRHAQETG

>Tb2.NT.88_12 [2870 - 2944]

MRNAYCLSSMIIISYIFLVGSKKGR

>Tb2.NT.88_13 [3378 - 3473]

MNTYTHIYIYIIRCDMKNHLSIRYEHLSIHLN

>Tb2.NT.3_1 [400 - 510]

MQLCGLAVNTGLLRFALLMNCCFVFCLQLTTAGQPFV

>Tb2.NT.4_1 [188 - 493]

MIDGGAAPRPDNHHIQCESPTQLLRTTQKAHSIRWHLSKISAYNRITGGYESSDFESLGFLVTVVTSACSALAVLGNELALKRRACCGMHIAPPQVPTPQIR

>Tb2.NT.4_2 [1094 - 1234]

MEPSVPQCRAVCPHPLGYEVCAKVQVDAARVRKKQPCTFPIRRKQRV

>Tb2.NT.5_1 [134 - 217]

MAELNGPFYIRVSCLNTPVCAVGISAVA

>Tb2.NT.5_2 [248 - 331]

MLFTISQRERQNQSQDSPFTFTHSSDRE

>Tb2.NT.6_1 [194 - 301]

MWNKRKLSAAERRFIKKSGVITSHEYDFKKKKEGKR

>Tb2.NT.7_1 [153 - 359]

MFNPKKMLEAKVQEKAEEQTKKAVDNQLQKTLGDKFSGLVPDKLKNMVAKKATSSLTDQLTKSAAGGAK

>Tb2.NT.7_2 [445 - 528]

MREEPLQLFIIMSETRGAAVIVRKEKKG

>Tb2.NT.7_3 [540 - 617]

MIGGHEESEINYIIYVFMCVCVYIYM

>Tb2.NT.7_4 [781 - 993]

MEDLGDETKPTSEIKKKRKEKKRRGEGCWKRGKNIGSQVKDKANETATKTIVFQDTYALGFEFIIIKYSIH

>Tb2.NT.7_5 [950 - 1066]

MLLALNLLLLNIRFIKRFIQMLFFLLPMCLSPFIHTFSC

>Tb2.NT.9_1 [17 - 136]

MPVCVCNARSGGGSSSLGPKVNCMMLWTMWRTERMKGVAV

>Tb2.NT.9_2 [94 - 216]

MDNVAYGKNERSSGMKSAGESDYRGNGVLKASHAPPLCALR

>Tb2.NT.9_3 [220 - 294]

MFCAMLLRSFSYCFPLLFLHSVCCR

>Tb2.NT.10_1 [335 - 457]

MGQRLSSCWTLCELNSMNNICATLFLLEMFGRRMVPIIHGT

>Tb2.NT.10_2 [450 - 527]

MELSGWNVCVPRPAEEVEFHEHILKR

>Tb2.NT.11_1 [231 - 323]

MMGVFTCRNRTLFLLSSIWVPLWMWLFTSIS

>Tb2.NT.11_2 [534 - 608]

MKVRAARLSKAQWGVSGRRETVVNE

>Tb2.NT.11_3 [383 - 625]

MVVSSTAVAEELRTADIINSVEFNAMRIPIALRKGVVNINKAETTKKQSRYEGQGSTPIKSAVGCFRKKRNRSERIVSTTW

>Tb2.NT.11_4 [848 - 940]

MAANKQTASQQEGIRAQSKDAPNQERGADWT

>Tb2.NT.11_5 [906 - 1133]

MRPTRNVGRIGPEPQPVLMWATRTLGEPNKRVSCSKCAQQRDGELDIKVSGAKRWTSRRRVLNRKQEIQQARIALL

>Tb2.NT.11_6 [1067 - 1333]

MDFKEACIKPKARDPASTNCTAVTNTKDFCTALKSTSYMYSNDGKLHVFEAVPVKPAAQWEFANSIFIAVTTGQSKCLISTREKAGRTY

>Tb2.NT.11_7 [1294 - 1422]

MLDFDQGESRANVLRAREKCLKETGSEIYQLTQQVTESRNKGK

>Tb2.NT.11_8 [1592 - 1684]

MVAVREVIPHYRDVRVVNTGSMQEPERGNVT

>Tb2.NT.11_9 [1692 - 1772]

MDGKCVEPRRLPSTGSCAVSVSTLLKL

>Tb2.NT.11_10 [1696 - 1788]

MANAWSQGDYHLLEVVPSAFQPCLNYNCLYD

>Tb2.NT.12_1 [107 - 268]

MICTDKSVTCSRVCTLVSRKGMEGLDISFLIIIIFTRFAHTHTHTSNNLWISCL

>Tb2.NT.13_1 [55 - 171]

MQAKVRLKKQTYILYIYIYMCVCVCVCVYVISEKEGFNA

>Tb2.NT.13_2 [171 - 287]

MNVRSQMRVIFFYKKKREGKKGSSSFHKSHRFSRTHATD

>Tb2.NT.13_3 [277 - 417]

MQLTKIHTVNGVIIFNKGRIVKKKKREEEKKQRQPPDVLLLISHNFE

>Tb2.NT.13_4 [386 - 628]

MFCFLFLITLNELSLSISLYILYFYLFLYIFFLLLCVFFLCVCVNSVSNWRYYIKKGISLFVFFSPLNALQMQLRHTSIVV

>Tb2.NT.16_1 [212 - 310]

MRIGVELYLTESAYPSHFLPLTCRCVVGGCLNK

>Tb2.NT.16_2 [350 - 550]

MWCCPSAVHRSVLSVLSLVGLVSVNCRAHLRMQEDFGNHNSNNQMKWRGNIVPKQYPLKTFVRASQH

>Tb2.NT.17_1 [59 - 160]

MFLDVLPPARRRTKMGVVLMGKKYWRCRRHVCPT

>Tb2.NT.17_2 [147 - 230]

MCAQRECRDLNTHMILLYFYFHTSSRRN

>Tb2.NT.17_3 [362 - 445]

MFSPRLSADLNILVRQKGWFIFYFFSYG

>Tb2.NT.17_4 [441 - 548]

MGDCTLAAFTPESSTLYRKVLDGPPPNEFVPFKSVQ

>Tb2.NT.17_5 [557 - 712]

MCRGTSQFVPEPTSGAALPDHAMQCRLLANQLFGARLPLYIQQITLGGRLSR

>Tb2.NT.18_1 [209 - 313]

MGNTAGKYLFPSFFSEHLLRRIFFLLKKKVLTYVW

>Tb2.NT.18_2 [306 - 422]

MFGETRRFTSRRGCVVFCALMPAVCQLREVPCGCGSAAH

>Tb2.NT.18_3 [449 - 589]

MRKARWKMWERVGTRTGTGIIGGNFTSTPERAHWMGSSEKTPISSGS

>Tb2.NT.18_4 [597 - 692]

MRPCKACNHFFMHARLLFYPRACRCYANQHGY

>Tb2.NT.18_5 [634 - 795]

MLGFCFIRVRAGVTQTSTATDCPNGSLPKPSAPLCRLCCMKYDCICHSRHCGQE

>Tb2.NT.18_6 [984 - 1058]

MVIPCSGMRAVVYSRDSGSLGALGA

>Tb2.NT.18_7 [977 - 1081]

MVDGYPVFWYACRCLFQRLGFSGSSGSVTLHFWGQ

>Tb2.NT.19_1 [286 - 528]

MPLEDLTSLKLIKCVVGLRVSAGAGIIAILDYGLMGFTPRDAVGGCSSLDSDPLPSVNDTLHRPYGVTCCRFYGRSTCPLP

>Tb2.NT.19_2 [531 - 632]

MIDSVGTSPVVCSRDSATRGGHWWCLYVDVLLLS

>Tb2.NT.20_1 [205 - 291]

MLLNVADTLWSLDFVVRMGRRRRGSSNPS

>Tb2.NT.20_2 [152 - 307]

MLWDPSSCFVFVNFRMGGCCSMWLILCGLLILLFAWADVAEGAVTHRDAACV

>Tb2.NT.20_3 [512 - 625]

MMPVGFENGKKMPPGRVVERLFLALSDCIYPNSLWGST

>Tb2.NT.20_4 [674 - 952]

MFSVSFVAPLLTALCATLGPCFCTILLYCTSVQCRSVGHYNICYAYRLQFFLRVFTGWLRGPFRCNFIRFLTLFHRCLRVTDNLSIRYISFVS

>Tb2.NT.20_5 [1259 - 1507]

MLLCLLLRHYTLLMIMDTTVTMGQLPTPLGQIGLLLISLCASRLLKRTFFICAYHFPKIEGALSTVFLLRLVRRHQVGMKLSK

>Tb2.NT.20_6 [1507 - 1608]

MKVIAMKAEDGWPTMPIWEMRSHSFVLSTRAFCG

>Tb2.NT.21_1 [229 - 429]

MQEDDADETNNNNNNNNNKKKDAMAVTGTESKEKKKYPNNLVIACRKLQMRMSDYISRNIYYINPVE

>Tb2.NT.21_2 [429 - 563]

MMDFTTYLNKIFIFLYLLSCNNVDNNVILMWKTVSSFITKFIIPC

>Tb2.NT.21_3 [684 - 851]

MNELIVISLCVSFVCFFPPFCFPLFFFLLTLLYFTLLYYFFSSFAFCSFFFCISTC

>Tb2.NT.21_4 [867 - 962]

MEVAIKILLLLLTISVLHPFHLSTFPLSFFFL

>Tb2.NT.21_5 [710 - 964]

MCQLCLLFSPFLFSSFFFPPHFTLLHFTLLFFFFVCLLLFFFLHFYVLKCFINGSCHQNSSSAFNNFCFTSFSFIYISFVFFFSF

>Tb2.NT.22_1 [190 - 279]

MGCGFVLLHSVLVIHCSYVCLCVCFERSGR

>Tb2.NT.22_2 [242 - 328]

MCVCVFVLSEAEGNTNSLQISTFFFLLVS

>Tb2.NT.22_3 [482 - 625]

MYLFGLWCIVKYIYIYIFHGFSRRKCVEVSFSFFPSMLQGEYVCVFLL

>Tb2.NT.22_4 [537 - 629]

MDFPDGNVLKFLFLFFLRCYRVNMYVYFFYN

>Tb2.NT.22_5 [610 - 765]

MCISSITKLYLFFFLKKGKINCYLFCRRVCEREAPYVMGAIIHFLSPQSPPL

>Tb2.NT.23_1 [10 - 210]

MLESDTQFPSATDIKTVASLTEVDGQVITFAQLRANRLKAHKLLDNVEELLRIAEEIKWAFMWVSTS

>Tb2.NT.23_2 [146 - 220]

MWRNCCGLRRRSNGHLCGFPLLKRK

>Tb2.NT.23_3 [183 - 374]

MGIYVGFHFLKENNNNSNNIKNKMKKKIKVPTVSFGLWCCCDIMSYFHFRYSSAGIDLHMHTWV

>Tb2.NT.23_4 [433 - 549]

MKAHVNGRRGRKPLGQFFRASASTSVCAFLFFLLMFVCL

>Tb2.NT.23_5 [556 - 630]

MCICKRSASMTSLHNFSQYLRKYMH

>Tb2.NT.23_6 [564 - 713]

MQTFCLHDFTTQFLTVLTQIHALIHTHTYIHIYIYINIYIYIFMYSLRER

>Tb2.NT.24_1 [289 - 438]

MTIIIIIIIITIIIIIIIIITRSTNGYNMCFERNGKHCRVKSAGVNETFK

>Tb2.NT.24_2 [452 - 583]

MFACEMGELERNISVLKLIQNKTAAKKKKRKRKRKRKRTGISYG

>Tb2.NT.25_1 [173 - 415]

MLCFSTVKLLGACGHRCKTHKITQHINYTPRFRAANAVVGLSVALCIGTYGIMADWVYRGYVDLWYGVYHIRDDDNDDACG

>Tb2.NT.25_2 [321 - 419]

MASWRIGCIAVMWICGMACITFVMMIMMMRVDK

>Tb2.NT.25_3 [710 - 793]

MVFLHFVMIRLVGVLLPSLVFFSHPILH

>Tb2.NT.26_1 [351 - 437]

MNATVSFKARLVAVVMDLSRRVARTRWRA

>Tb2.NT.26_2 [275 - 538]

MCDYGKITFKRRLRYCGCPAEVLSADECHCFFQGAFSCGCYGPEPPCCAHSLEGVSVRTCICVYRYLPMWVPAIFLFSSHLISLHVIM

>Tb2.NT.27_1 [202 - 288]

MLCEFFFFLPSLRGDRFNHGAVSRTCPVC

>Tb2.NT.27_2 [167 - 337]

MLSYRHFKVILACFVSFSFSSLHCVVIVLTTVLLAERVRCVSYGAFHSTQPWRKVVY

>Tb2.NT.27_3 [294 - 425]

MAPFIPRNRGVKWFIDPRKGHTSYDDDDDYYYYFSVCTLKRSVG

>Tb2.NT.27_4 [440 - 658]

MRVEGRKSNSGRETRPVVDSKQCLGGGGGGGREVPSFVPFKIKRFFLHVCVCVCVFPPPFPLLGLRVLMIGIG

>Tb2.NT.27_5 [836 - 1009]

MYLHCNDLMHNEAPGDIMLLVVAYPCSFNRKKRMTTTPTKAAVYLGFEEFLLGCGAIR

>Tb2.NT.27_6 [1223 - 1327]

MWQLWKVVFFEGVEGGETEIIYISMPFVFFVRYHS

>Tb2.NT.27_7 [1327 - 1422]

MIVYAAAKMSSLKPTLFAQRADFPGYAWSVGV

>Tb2.NT.27_8 [1583 - 1696]

MGSCGKMVSIGRVCEDSVSLRYQGERRLREVLYIYIYY

>Tb2.NT.27_9 [1823 - 1966]

MQPLRKRRGGWRNIVTFAKEIKQPRLAGSKSGVRSEGSYFNWVYGIAG

>Tb2.NT.27_10 [2033 - 2149]

MLNIVTGTDVANGGVRPIFRGVLFRGKAEARSVCGRLPG

>Tb2.NT.27_11 [2067 - 2252]

MEAFAPSFVAFFFVVKRRHDQCVDDFPDDENTTIRGSLTVVRNKECIGMGTTVGQIFERPKG

>Tb2.NT.27_12 [2324 - 2476]

MYLLVLPCYATVRSGSVEEEDGFPQFSSRRSLVRSLIVGFPIHPQQKQETR

>Tb2.NT.27_13 [2552 - 2719]

MVFISVEDISFQLPRERHFNSLTGYTPIHSPTMRKAAALRTYAFIFKLTPYCCGPN

>Tb2.NT.27_14 [2676 - 2786]

MPSFSNSHLTAVAPTSALLPNEIRQVILHYFPLYDRH

>Tb2.NT.27_15 [3182 - 3262]

MGRKIYALHMCAVPWCCRRVYNPAQDN

>Tb2.NT.27_16 [3012 - 3386]

MAGVLHETATTVSYRGALCKLAGVELQGINLSPPQRLCQLELLISREINWLAAVLEEWEEKYMLFTCVPYRGVVDAFTTLLRTIKRHLETENSSKDNEKEWDALLASSITEFQVEVMEIGKGKSY

>Tb2.NT.27_17 [3301 - 3420]

MKRSGMRCWLLALLNFRWRLWKSERVRVTSIFFFSFYLYI

>Tb2.NT.27_18 [3454 - 3588]

MFFFCHPMVKLPCGDKTKTRFLNSLKVIEYAYLKKSVCGVDYVFS

>Tb2.NT.27_19 [3564 - 3704]

MRSGLCILMKSIIQISNAKRKQLYDGNFFNFCGASINNSFTNRYCST

>Tb2.NT.28_1 [104 - 274]

MHGRSVEYHSLYLLDEFSFGELAYDYLLDVLSRGLVEVDTYKVMGAAVTCGSPIIKW

>Tb2.NT.28_2 [294 - 548]

MGAVRQAFCFVWRRTFLHLLWRDDLSHCWFLLCNRNARGKVCLLKCDYSFWGVFITGMLGMLITAVSTVVRRINSCKCVSDPFSF

>Tb2.NT.29_1 [260 - 406]

MKNTRNDLRVREWHSEPVKCVPCLLLHAMKCKYIPNIQVCFVDIFVTEV

>Tb2.NT.29_2 [478 - 648]

MHGRSVEYHSLYLLDEFSFGELAYDCVFDVLSRGLVEVDTYKVMGAAVTCGSPIIKW

>Tb2.NT.29_3 [668 - 922]

MGAVRQAFCFVWRRTFLHLLWRDDLSHCWFLLCNRNARGKVCLLKCDYSFWGVFITGMLGMLITAVSTVVRRINSCKCVSDPFSF

>Tb2.NT.30_1 [260 - 406]

MKNTRNDLRVREWHSEPVKCVPCLLLHAMKCKYIPNIQVCFVDIFVTEV

>Tb2.NT.30_2 [478 - 648]

MHGRSVEYHSLYLLDEFSFGELAYDCVFDVLSRGLVEVDTYKVMGAAVTCGSPIIKW

>Tb2.NT.30_3 [668 - 916]

MGAVRQAFCFVWRRTFLHLLWRDDLSHCWFLLCNRNARGKVCLLECDNSFWVRLHYRDVGNADYCCFHCSEANKQLQVCFGSL

>Tb2.NT.30_4 [840 - 923]

MLGMLITAVSTVVRRINSCKCVSDPFSF

>Tb2.NT.32_1 [74 - 244]

MHGRSVEYHSLYLLDEFSFGELAYDCVFDVLSRGLVEVDTYKVMGAAVTCGSPIIKW

>Tb2.NT.32_2 [264 - 512]

MGAVRQAFCFVWRRTFLHLLWRDDLSHCWFLLCNRNARGKVCLLKCDYSFWVRLHYRDVGNADYCCFHCSEANKQLQVCFGSL

>Tb2.NT.32_3 [436 - 519]

MLGMLITAVSTVVRRINSCKCVSDPFSF

>Tb2.NT.33_1 [196 - 291]

MNIYIYIYICRKYQHHVVRMTHSMAGVTYLDV

>Tb2.NT.33_2 [397 - 561]

MVYIYIYILFYLFIFLLFYLYAPPHAAWSPSYVGMQKGVRPYRKKKHKTKQKRGA

>Tb2.NT.33_3 [338 - 613]

MGGDEERLLSWGRGETIRRQWYIYIYIYYFIYLFFYFFTYTLHLTQRGLPHMWGCKKEFVRIEKKNTKQNKKEVLSPPPNPLPQFSLPESKS

>Tb2.NT.35_1 [15 - 101]

MFPPSSFRALSTPINAYSLLRLPYKETEG

>Tb2.NT.35_2 [325 - 468]

MYMYMYIYDYVFGYFVSIVSYGRSAFASVFISSPFPLPPPLFFCIYTS

>Tb2.NT.35_3 [347 - 496]

MIMFSGILFPSYHMVAVHLHPYLSLPPSPFLPPFFFVFTLHNCVSQKNNA

>Tb2.NT.35_4 [507 - 590]

MNQQEVIISSQMINPSQLQLKDNAHLSK

>Tb2.NT.35_5 [772 - 876]

MTPLISRCTRVFFFFFDCYWRAAYSRYLVLFGLHL

>Tb2.NT.46_1 [188 - 352]

MASWTTYQSGMRRYGLVFCCFSAALAMLVNQDGAKQNWQSMAERYDGQQLGKKWS

>Tb2.NT.46_2 [321 - 452]

MMGSSWAKSGRKGVRSDTSLAIPVQHLFTFPPISVLIVSRISMN

>Tb2.NT.47_1 [23 - 208]

MSMDTRMVNFGFTRAALLCGLLYLLTAVCSLHVTAADGDDGRVIVNVKEYFSMPNTVCIYML

>Tb2.NT.47_2 [273 - 380]

MVCMIIFLLCILLYWYDHEDDTVVGSAFSSLFGMVI

>Tb2.NT.48_1 [58 - 162]

MGTTEIKCVTSFEGLMLFRRFPKDVTAFARNVDKR

>Tb2.NT.48_2 [359 - 433]

MMTTTVLHVMKDLCKNFTTVVKACH

>Tb2.NT.48_3 [600 - 758]

MLYLHIFFRLCEVTVGVGSAPQLTWIMNRRGFGFHHSRMLRVTSHSRVVPNKS

>Tb2.NT.49_1 [153 - 254]

MVFLVSFLQRHGWKFCSAHYNFLDRRYWCRTLSL

>Tb2.NT.49_2 [80 - 349]

MDFMNTSETASSAARTAAVGAMTTDGVSRQLPSETRVEVLQRTLQLLRQEILVPYFEPVERLRVTNNRAAYVHDIVDALHRSAFGRGTEV

>Tb2.NT.49_3 [309 - 419]

MRCTGARLDGAPRCDVYQAFSLFVTVLLPVLWLTCLH

>Tb2.NT.52_1 [19 - 189]

MLGCVVRLHASCRCEAHHTTSGCETAVAANRPFVAMWRERSGCGSRLAGERMRNQWE

>Tb2.NT.53_1 [22 - 111]

MVHSSQRKAQNASLVAFIPLPPHQFFALNK

>Tb2.NT.53_2 [142 - 222]

MSDCGGGKNGGGTSWRCNISHDRWRIH

>Tb2.NT.53_3 [167 - 391]

MVEAPLGGATLATTVGEYIENHLNRKQRSNPWSKNRFVSYSFLKGVSKRVRLEVCPHSNAHSRRQRTATTALMLA

>Tb2.NT.53_4 [659 - 742]

MKNTQPPHHIKEKITKAQTANIKKHHNS

>Tb2.NT.53_5 [808 - 918]

MIINVLSTCVSLTNLLGYVIWLLSSIIICVILFIFLV

>Tb2.NT.91_1 [221 - 364]

MNPRHTWMGTPPRTSRRPSATSPQSNNLRSPFCWCPTASQPSDMQPTW

>Tb2.NT.91_2 [374 - 544]

MAASSSIRAQPMNVSCVRIHSSAQRLGDRTYRSCAPQRGRKGEHRAMSKVPYTPTAC

>Tb2.NT.54_1 [75 - 182]

MKVEVSGRRLCVWVDVPQRDQNGTLGTRDKRCASLP

>Tb2.NT.54_2 [118 - 216]

MSHSGTKMGRSARVTNGVLPCHEGRNVKGNLLG

>Tb2.NT.56_1 [60 - 134]

MRISNFVKKINLECSFFFERYHLEK

>Tb2.NT.56_2 [562 - 798]

MHVGLKREAGKNKGPGQGMSVLFFFSRCWTVCTKQTIRAAPVVSSSRPLARPLEHNPFQFLGVQEAMTGSVVDCLLRGS

>Tb2.NT.56_3 [642 - 827]

MLDCLHQTNNTCSPSRELLPSFGTSSGAQSLSISWGPRGDDWLSCRLFTARLLGGKGACAKY

>Tb2.NT.56_4 [877 - 990]

MLAIIRAETVEGPTLARKTFVTVLVVFGRYHRLDENLQ

>Tb2.NT.56_5 [977 - 1141]

MKTCNKARCLINVSWMRSICSNGRRVTTPHHLLGCWRKMTQLRQRCVYKAFLTFH

>Tb2.NT.56_6 [1217 - 1306]

MGFLQPDATHRCTTLGFSPIFSPRVSGDAI

>Tb2.NT.56_7 [1352 - 1474]

MLKTVEAADFVAFPLALGQASKNDSGNQASGLSLPLALQTS

>Tb2.NT.56_8 [1604 - 1699]

MQRLWNEAANAIYREGTSFRRGGDFQQCVEGV

>Tb2.NT.56_9 [1877 - 1969]

MPTACMARSISHRKDARKREWHHARYACVCA

>Tb2.NT.56_10 [1530 - 1973]

MIDHSCGCHNQPPCNHLRVPEMWWECKDCGTKLLTPSIGKGQVSDEVEIFNSVLKAFKIQQRKGELEWIVDTASQKLIRLARRRFIIPKGDLWAAAEANGSEPNIILVLMPLHHSPCPPRAWPAPFHTERTRGRGSGTTRGMRACVLE

>Tb2.NT.56_11 [1989 - 2207]

MTSRTHPRPMIPLSLEKVVCRGRGIWDEGFMHAVGATQTKVLPSRVEVDLVQWRCTRADGHAFVASPLVYNCW

>Tb2.NT.56_12 [2068 - 2238]

MKDSCMPLGQHKPKYCLRGWKWTWCSGGVPAQTDTHLLHHLWCITVGESVQHFACLC

>Tb2.NT.57_1 [278 - 388]

MPSRLCGRGQRHCVKLHGPLVTPLCVVKWFVVTFRYG

>Tb2.NT.58_1 [268 - 450]

MCSDLLCSHAHTHARQSSSSVLSARFPDLYDTCFYRRRRGAMPRRLHVSCESHTFLGLSHF

>Tb2.NT.58_2 [305 - 451]

MLGNLPHLCCQHGSQTSTILVFTVDGVAPCLGGYTFHASHIRSWVCPTF

>Tb2.NT.59_1 [330 - 410]

MCFRVLKNRNSNSDVIADGWDKGVRFY

>Tb2.NT.59_2 [416 - 508]

MGYGRECTLLVTDSNHVRKSSDNLSPLFTYL

>Tb2.NT.59_3 [462 - 584]

MLGNLQIIFLPCLRIFNVVGSVVYPWIGCCISSGGALSKDP

>Tb2.NT.59_4 [685 - 759]

MGYVSRVCEVVWTQIPLKASETTQS

>Tb2.NT.59_5 [769 - 861]

MCAFTISFRVNSECFAFFFSSPSSGEKKGSQ

>Tb2.NT.59_6 [903 - 1013]

MIYIRYALCYHHICLSHTDLIVRVRASTLLLGSLDYV

>Tb2.NT.59_7 [1082 - 1210]

MHYFLLVRLEGGGCDANMVRETDVSSCTLCGGFSLGIGYCVST

>Tb2.NT.59_8 [1125 - 1232]

MRTWCEKPMFLVAHYVEDFLSVLATVLVLEWKSVSC

>Tb2.NT.59_9 [1214 - 1324]

MEIRVLLKAASRRLGMWIHTWTHLSVFQTFTGGVGVV

>Tb2.NT.59_10 [1273 - 1425]

MDALISISNVYWRCGGRMTPLSSCVPLRIYHGILVFTYVGFHCIYSRSVNL

>Tb2.NT.59_11 [1385 - 1570]

MLVSTASTAGRLTCSIEVGHGKNHEGPHPVCVRTFPYITLFPTKTLPSAGVSSCPRRRKKVG

>Tb2.NT.59_12 [1570 - 1671]

MTIYHLTNCICMENLGTECCERNMARSTKLHFRK

>Tb2.NT.59_13 [1730 - 1810]

MIEKCCTKYSNYHYWNGIRAFYRENGF

>Tb2.NT.59_14 [1978 - 2202]

MRYAFFALYRSVVWEWAGLTRLSASTKKRMYLCWFINIYVFFIAFTTHYSHVAPLSCIKAIRVCWRCFFFFLIVS

>Tb2.NT.60_1 [57 - 167]

MIYIRYALCYHHICLSHTDLIVRVRASTLLLGSLDYV

>Tb2.NT.60_2 [236 - 364]

MHYFLLVRLEGGGCDANMVRETDVSSCTLCGGFSLGIGYCVST

>Tb2.NT.60_3 [279 - 386]

MRTWCEKPMFLVAHYVEDFLSVLATVLVLEWKSVSC

>Tb2.NT.60_4 [368 - 478]

MEIRVLLKAASRRLGMWIHTWTHLSVFQTFTGGVGVV

>Tb2.NT.60_5 [427 - 579]

MDALISISNVYWRCGGRMTPLSSCVPLRIYHGILVFTYVGFHCIYSRSVNL

>Tb2.NT.60_6 [539 - 724]

MLVSTASTAGRLTCSIEVGHGKNHEGPHPVCVRTFPYITLFPTKTLPSAGVSSCPRRRKKVG

>Tb2.NT.60_7 [724 - 825]

MTIYHLTNCICMENLGTECCERNMARSTKLHFRK

>Tb2.NT.60_8 [884 - 964]

MIEKCCTKYSNYHYWNGIRAFYRENGF

>Tb2.NT.60_9 [1123 - 1356]

MRYAFFALYRSVVWEWAGLTRLSASTKKRMYLCWFINIYVFFIAFTTHYSHVAPLSCIKAIRVCWRCFFFFLIVSNAR

>Tb2.NT.61_1 [268 - 450]

MCSDLLCSHAHTHARQSSSSVLSARFPDLYDTCFYRRRRGAMPRRLHVSCESHTFLGLSHF

>Tb2.NT.61_2 [305 - 451]

MLGNLPHLCCQHGSQTSTILVFTVDGVAPCLGGYTFHASHIRSWVCPTF

>Tb2.NT.62_1 [121 - 195]

MFCWCAVASALVTVLSLCPNCVCER

>Tb2.NT.62_2 [256 - 375]

MCEYLLICPRSHNCINVPSYPSKRCVDKRLGGGCGRKCLC

>Tb2.NT.63_1 [278 - 373]

MPSRLCGRGQRHCVKLHGPLVTPLCVVKWFVV

>Tb2.NT.64_1 [60 - 161]

MRISNFVNFVFFAVRLCAKLSHVCTFSHFFVGLH

>Tb2.NT.64_2 [397 - 480]

MKIGRCPYLLSVTRRLCGTLFCNIYLRI

>Tb2.NT.65_1 [101 - 190]

MENEKNSHSPPTQWRAYVLPWKSRLKYLPK

>Tb2.NT.65_2 [139 - 291]

MESIRFTMEVALEVSPQVEQTNEELAPKGCVVKYVERVMCMWGSYVKDKGF

>Tb2.NT.65_3 [453 - 614]

MRLMFFATALMLSLFCTSYCMLNIPFSIIFSFFCLSFPGETHVVHMRWNTLNDT

>Tb2.NT.65_4 [593 - 682]

MEYTKRHLTLWENVINACNSIVEKCVFSTP

>Tb2.NT.65_5 [770 - 964]

MQSRKVICFLLLLRLLMICVYTCSFLFYIVSNRRQKRGYYILHILFCMLAWLHNLCCCRGVCYGL

>Tb2.NT.65_6 [1101 - 1184]

MRKLNEHVYVSICIFIFAYLLFMYIISE

>Tb2.NT.65_7 [1323 - 1421]

MTRVFKFSILLLLKSCELREDSYQYHMMIFETI

>Tb2.NT.66_1 [81 - 179]

MTRFVLREHQTNRVRWKCRIGSKGKCIIMCRCV

>Tb2.NT.66_2 [173 - 448]

MRMRHCSQHLKKAKSLLTVLIILSVYHNLIISAVEGSLHSCVYSAFRVDILSLTTVASDDDDIVGFPVWKEVSLYSSARISVLCSSVERCCY

>Tb2.NT.66_3 [376 - 462]

MEGSITLLFCSYKRALFIRGAVLLLKRGC

>Tb2.NT.66_4 [621 - 869]

MLVLGYLYIGMARSSREGREVYKPVKGCTGTVSGMKVIEVSKEVSDVIILGKRLLLKVLIIYFPKQSNVAKITLKESLGGAMG

>Tb2.NT.66_5 [911 - 997]

MISGRFGGEARRYTTPNWWESSNGREYLP

>Tb2.NT.66_6 [1329 - 1454]

MVVKREISEKELVEAIVASKKTLRVALVQIEKQPTLKNWFPT

>Tb2.NT.66_7 [1625 - 1726]

MKPYISYGVVSVLPLWYHYVNVSSILSRGGQHVN

>Tb3.NT.1_1 [18 - 122]

MISSRWSPICSFLRVSNGDGCPVVHLLRTRHESGQ

>Tb3.NT.1_2 [67 - 141]

MVTVAPWCTFCVPGMNRDNSLLTVV

>Tb3.NT.1_3 [184 - 423]

MSKASKHFFCKDFLSQAVKIITEDIRIQQQLRHSSNGSCLEDSLHISRNYSLPGLCDIKSMGQHPTLQAPVWPLPDASDR

>Tb3.NT.1_4 [483 - 632]

MTTITILSSRISQSKLFLCGISSFLQVKRGCITWKEVVPHFGVAFLDFSD

>Tb3.NT.1_5 [536 - 658]

MRHQLLPSSEKRVYNLEGGGATLWSRIPRLFGLSVMKWVEL

>Tb3.NT.1_6 [698 - 811]

MQNSSYLVLGVVGIWGQMLVGRRRKWSMRSNISYSPCL

>Tb3.NT.1_7 [670 - 828]

MQGVRTHRTYAKFLVFSVRCGGDMGTDVSRKAEEMVNEKQHILLPVFVKFNFG

>Tb3.NT.1_8 [1475 - 1672]

MLSHGGMAQWLREVKWFRYVSVLMLPAGFKVYECINFLRVRILVNLGLLRGSNVSCYVLRKCARLG

>Tb3.NT.1_9 [1632 - 1706]

MCRVMCFESVLDWGKATNRTRRIGI

>Tb3.NT.1_10 [1787 - 1954]

MHEGRRGPVNESQRKERSGDLNAPCEKTGQTRRERENLKRATCLSCTTRFAGGILL

>Tb3.NT.1_11 [2040 - 2192]

MRLKLRIGNAGVKCSSREFVIIFLSKLSGYGGENFQRRFGEASSTQERLTL

>Tb3.NT.1_12 [2355 - 2528]

MHQRSRFGNQKETHRRLDFPNRRLEEAYVNVCLQGNSDRCHSYSAIKKFLDTVYTYHI

>Tb3.NT.1_13 [2443 - 2688]

MYVYRAILTAVTVTLLSKSSLTPCTLTTSKCSGFPAFNSCHALFKAVKLHQLLLLPTLLPDNTCKCFVANPYFIFNPGTCIN

>Tb3.NT.2_1 [85 - 357]

MLSPRRRMKSTQTPFQPYRAHPAPLLRHQRMQSQLQIRVMMFARKTTTKCMAITHIFVELSAPLLEVQKQERGSSRLVLRSWHGWESFRWY

>Tb3.NT.2_2 [54 - 404]

MAAAVKPEEANAVTTAENEVNPNPFSAVPGSPGSAAAAPTDAVTAANPSNDVCPKDDDEMYGDHTYICGIECPTFGSSEAGEGELSTRAALLAWMGVVSMVLVLVVVSVKCIGDARD

>Tb3.NT.2_3 [497 - 586]

MQTCTYALIVRCYEHFEFARCLFARLHLVH

>Tb3.NT.2_4 [556 - 738]

MPICALTSSALKRCLRRCSHRFVTGVHNLCISFIFIGVPYLWRKRIGSSCGREGASTTTPL

>Tb3.NT.2_5 [845 - 943]

MFKRCVIGGVGCQRLFPQHPPCFPWVSQNTDHQ

>Tb3.NT.2_6 [907 - 1083]

MFSMGVTKHGSPINCREEYKFISKRASLGVSIRVSLKHPPKCSTSRFQRAARSLGGFSS

>Tb3.NT.2_7 [1029 - 1229]

MLHVSFSTSCSKPWRILIIVAVSNPFCSVIRVPALHRLSFRREPVLEVGGRVLGRLVREGKGTQGIC

>Tb3.NT.2_8 [1429 - 1509]

MQSAGTSLSLDRCCEGDAPLCVCALGR

>Tb3.NT.3_1 [128 - 208]

MAPRTYLSSLTGSLDTNNPTLAVTRLG

>Tb3.NT.3_2 [362 - 463]

MCGRPLCRNDTGESDMPIFQAGGVRKGTPFPLDI

>Tb3.NT.3_3 [519 - 674]

MKPSVQLSTVPRTGQLSVTSRNKKTRASIGNRMNIGFTTQQLMEHFEQRRCL

>Tb3.NT.3_4 [704 - 781]

MGPIRVGQTAARSTEAPTQNDLGGNI

>Tb3.NT.3_5 [1112 - 1198]

MYQIPLVIFLGTSLPRSGTCRQRELVSLL

>Tb3.NT.3_6 [1104 - 1223]

MGACIKSPWLYSLELPYPEAGPVDRGNLCLCFNKACSCTF

>Tb3.NT.3_7 [1308 - 1418]

MKILLAEECKCLPPREGSEAHSLRRYDVVPLFGSQTH

>Tb3.NT.3_8 [1384 - 1485]

MMLFHCLDHRHIDRLYVKVGNHPICLEKYFETQS

>Tb3.NT.3_9 [1929 - 2009]

MFALSSCEIKENNNKASGCFEDVMKQC

>Tb3.NT.3_10 [2014 - 2394]

MLILGKGNSRSMSLFLTLQGIVLATRDGSLCRQKEVKWEYGCFRHIRCVCSVAHTDVCSCVGGFRSDRRERTMVVVLRINRSKRRTDWPQICYRCSCSQVAALCHCTLGRGGNPTTSSSNGCRIDER

>Tb3.NT.3_11 [2372 - 2506]

MVAGLMKGDGYVEGKGVLPNLVVVSVNAKRKKKKKWVAEKSGQSK

>Tb3.NT.3_12 [2715 - 2918]

MEGLKGIPKLECMSMDGFEGVRKSRRYMQGLARMWNFFLKPVRWLLGAKVWEDRCNADEVGGIISRWQ

>Tb3.NT.3_13 [2881 - 2958]

MQMRLEALYLDGSETSSKCVWVGGKV

>Tb3.NT.3_14 [2912 - 3019]

MAVRRLQSACGWGARCNMAVCPVFTFSGAGFECVKY

>Tb3.NT.3_15 [3056 - 3130]

MRKPKRCTYSVVELSRAGLVRSPLF

>Tb3.NT.4_1 [122 - 382]

MTVFNVTDIPRSGRTEDYLLWQVTGSSWITLKWDILSLTLRLVKCREDYLSTFCHGSRQKHPFSFQGHAPLCFRGSNISHVISSVSV

>Tb3.NT.4_2 [515 - 679]

MEHSRVTAVSGTNWNNTHSSGLCRIRGHNFTLRALLCSIGLLADSIPSALLITLL

>Tb3.NT.4_3 [689 - 790]

MVVNDEDVSHSIKSNDILASPSIVNEFRRLPFVP

>Tb3.NT.4_4 [933 - 1082]

MTEGTAVTGLNEQEMVDQRWSPCCREGLRLFGFLLPNVRICCLELNQKIL

>Tb3.NT.4_5 [911 - 1171]

MYYRRCSNDRRNGCNWVERAGNGRSALVSLLPRGVEVIWVSTSQCAHLLLGVKSKNTVMIPALNDPLCSAACSKTTPCTGESSGNGC

>Tb3.NT.4_6 [1101 - 1196]

MTPCVLPLVPRQPLVRGRVAVTAVKADKPHTK

>Tb3.NT.5_1 [5 - 223]

MLGKHLHYCSTDFTFYRRHIRGTNKRNASHTLVECHCNGTVLHRKSVDTGSPHRKGEADRSERHYSHRKRAIE

>Tb3.NT.5_2 [260 - 400]

MARACPKETRNIKEVTLLSPVRMETQTTTPKTDTLAQHFLSKELKIF

>Tb3.NT.5_3 [409 - 588]

MTLCDNVSGKVAEKHKVKSAIKGKTQSDGEEKHLRQEPQKQQPSTQPIRNSHRGYKTRKK

>Tb3.NT.5_4 [721 - 849]

MKSGFESLVGAPRNASKKWESNSIYQLVVPARCLSQNGDREYQ

>Tb3.NT.5_5 [761 - 862]

MHRKSGKVIAFTNSLSRPVVSHKTETASINEEIP

>Tb3.NT.5_6 [1048 - 1278]

MKEINLKTHKIPKCAPTRNRMKRIRPMLRGISLFARKKVNTDPVSEKDNKAFVWHMTTIWKKENGQHQIQTVFCCEH

>Tb3.NT.5_7 [1399 - 1515]

MRRVLATDRAINKHQWERKKNMERTPKRKQNTDNIQSGL

>Tb3.NT.5_8 [1524 - 1682]

MHLLYFILFRTNAIGMGMRATTLLREPSRRPFRSKRETYLKKRWRARKGKRLN

>Tb3.NT.5_9 [1833 - 1973]

MSPRNFKRKGELACCRCNKLDHRVKNRWHSHANKLKPKFPIRHNKCG

>Tb3.NT.5_10 [1871 - 2074]

MLQVQQTGSPCQKPMAQSCKQVETKVPHSPQQMRLTLDNGALEKRQPERPKEYQRHVVKKEAQAQLKG

>Tb3.NT.5_11 [2301 - 2456]

MQWEEIEKRSVPSIEDGSKGSPMYATPDKMYGTRYPHRALVALAWAQNRGLL

>Tb3.NT.5_12 [2650 - 2793]

MRHTEIQTARFKGIRWLKPWHVTMQLNRKMPVASLLPARTDLTPQWVK

>Tb3.NT.5_13 [2706 - 2864]

MARNNATKSEDAGGITVASANRPNAAVGEVVMQSRPLAKRCQTKSKHREMLGT

>Tb3.NT.5_14 [3290 - 3400]

MLALNTFIPITVSIVVFIAPPLYSRYSKLLSADLLAI

>Tb3.NT.5_15 [3482 - 3559]

MQGITQPADTCVFHGFEGEKHVSQPT

>Tb3.NT.5_16 [3522 - 3761]

MASKGKNTFPNQREEYFFETTEKQTKQIVSPLNVIFPGVFFVQVVGREGPLSSIQGIFFLPRGWRSRETKGAVTTTKSAI

>Tb3.NT.6_1 [274 - 468]

MILQPEWPSVPLFPNPSHLFSAVLPRIAHQIFESLINVDVKRGTRKRLGGHERDDETSTATTGRA

>Tb3.NT.6_2 [468 - 551]

MRQWKLHALFHRSYSELRGRLGGCCSGS

>Tb3.NT.6_3 [425 - 679]

MSVMMRRAQQRLVAHEAMEITRTFPSLVFRVKGQVGWVLQRLVAILFSDIKKGTRKSRQLQMGTETRRQIVLRCRPTQAILSHLV

>Tb3.NT.6_4 [643 - 732]

MSPYTSHIISPCVISSERFHRRVPTNDVCS

>Tb3.NT.6_5 [804 - 971]

MAMDNGRRTSFANEETGPKLAAADACQHPILRDFIKLSIARKGNETSWRQERHAAV

>Tb3.NT.6_6 [874 - 1263]

MRVSIQYYGTLSSYRLLGKETRPRGVKNDMLRCNFVLFVLLINSMAPFRGRFISKPFSVNVRKNVKTPNAIYFIVHGTKHTNTPSRELVFSRERLTSVSGRILNEKSFPYEVVQRFNSFFFFHPIACVGN

>Tb3.NT.8_1 [361 - 474]

MITLEVWRIPHDVTSDEYLHTITSTHSVEFNFITVQHS

>Tb3.NT.8_2 [571 - 726]

MRNHYEFVECTNIRFYTSGGNISRIYTTRCDVSAQFPSLLVAASVSYVEKFI

>Tb3.NT.8_3 [710 - 829]

MLRNLFNSNVFPLQSDSQRPLLCYDFPELTKKKEEKEGHC

>Tb3.NT.8_4 [938 - 1036]

MHGLKDFITEPPRYVCVTEWREQLFHVCGTPKH

>Tb3.NT.8_5 [942 - 1046]

MGSRILSLNLPVMFALLNGGSNCFMFAELPSTEQN

>Tb3.NT.8_6 [994 - 1155]

MEGAIVSCLRNSQALSRTESRKRSCAFSGGNVEECNKNNFKKCHHRVIYFQYII

>Tb3.NT.8_7 [1243 - 1359]

MIIIIDKVGKICIEILFFFLSSPSANRNGRNECVGCECN

>Tb3.NT.8_8 [1325 - 1402]

MVEMNASDVNATKMKHLNGSDAKRCS

>Tb3.NT.8_9 [1658 - 1804]

MHVGAHSYGELSVNMSNFPFLKRLISSDMHSNDKAAFCDPQEKHISFLS

>Tb3.NT.8_10 [1924 - 2112]

MFLWLELQLCSTALKDVTLLHLAISQRILIVNTVRLELSSGCKWMRFQSKQIPFATLIFCGIA

>Tb3.NT.9_1 [243 - 326]

MIPLDKHAEIFLLHHPKHLLPPDVPIYF

>Tb3.NT.9_2 [262 - 387]

MRKYFFYTIQNICCLLMCRYISDVNVLIKASRHWVAKYVCML

>Tb3.NT.9_3 [335 - 523]

MCLLRPVDIGLQSMCVCCESFGMRVRVFFSIFFSPPLVVMSGVETVFYRSCKIYPVSAPSLLN

>Tb3.NT.10_1 [14 - 106]

MRRRRIIRIRGWGRKEGRKGEFGPPLNYFKL

>Tb3.NT.10_2 [218 - 370]

MSKLKGNEFIFIFITPAHTVTPRTSRICVPEKKKPKGKGGNVGEDDVKKKS

>Tb3.NT.10_3 [339 - 452]

MWEKMMSKKKVEDVDATFWRRFLLFFFAFFPQYFATSC

>Tb3.NT.10_4 [376 - 561]

MLTRLFGAGFFSFFLPFSPNILRLPVESGGQMRLYINSWRYLFSKGWKLRSWVLILLVISCV

>Tb3.NT.10_5 [489 - 641]

MAVFILKRMEIAVMGVDFISYFMCLKPIGGMRAAVLLYFNFICLSSFLFWR

>Tb3.NT.11_1 [9 - 92]

MFLHKFSGSGEMKFSLVSNLLGLTGDPA

>Tb3.NT.12_1 [226 - 348]

MLYAFRMFGEVFFVIVVLRTILVILFGRSPLADGSCQVLHA

>Tb3.NT.12_2 [323 - 442]

MVVAKFYMPEEEGGGGSRVSETPTFHEGREDIFAASANCS

>Tb3.NT.12_3 [399 - 503]

MKGEKTFLQHQPTVVSRRLPLAFCEGWLFWRWHPT

>Tb3.NT.13_1 [35 - 115]

MHNTSRVVNVLQCLSVETEGRSLRNVC

>Tb3.NT.13_2 [60 - 149]

MSFSACQLKPKGGVFGTYVERALLLCCGNC

>Tb3.NT.13_3 [657 - 734]

MVLKTFSLFSSLPSHLPYLPNGGRQL

>Tb3.NT.13_4 [641 - 775]

MCSSLYGSENFLSFLKFTLSSSLSSQWWKAIVRLNFYAAFTYTLC

>Tb3.NT.13_5 [718 - 885]

MVEGNCEVKFLCSVYLYVVLTSDLRFSYCNELCCSAMRRSWRWGVFPCQGKGGSGV

>Tb3.NT.13_6 [806 - 901]

MSCVARLCVGVGGGAYFRVREKEDRVSSTLKN

>Tb3.NT.13_7 [913 - 993]

MLSTFCFIVHLSLGYFFVFEHVCTPLH

>Tb3.NT.13_8 [909 - 1007]

MNVKYFLFYRPPVTRLLLCFRTCLHTTTLTKVL

>Tb3.NT.13_9 [974 - 1060]

MSAHHYINESPMITVLYGLLQDILTTVVS

>Tb3.NT.14_1 [55 - 183]

MILSLCRCLLKACYHATVRLLLYSRAEKALMIKQISDAEQRCF

>Tb3.NT.14_2 [224 - 319]

MRREKLVRTKFYYITLTSTRYILSICLIYIYI

>Tb3.NT.14_3 [322 - 411]

MLSQFDTFVAPPKWFLRGGASEGSRLMSIT

>Tb3.NT.14_4 [360 - 635]

MVSKRRCFRGVKADVDYLAAGVIDGDFFVCVEHLLKAAPQLSTSVNKCYKAVIILKVLLSLVDEICVGFILGVASWVYVVISCHIFFLKLKE

>Tb3.NT.15_1 [70 - 441]

MMRPSTGSGTPRSDAGIKRSVTLGIDIMNEDSFWEISGIPLKSRMRSFISRERLTPKNVGSPCDLYLFPRYDEQPLCQSFKDLLNRHRSQLRETAKSYERYTSEVTNIRKKTAWFEPQKPPKFL

>Tb3.NT.15_2 [491 - 631]

MDEELGCSRSGWGNELLYMEKGFRHRCVCGSTRHLAGGLYSTPSPKK

>Tb3.NT.15_3 [638 - 727]

MVSCLSGGSNSAVVGMCLLDTTMPHVGCRL

>Tb3.NT.15_4 [711 - 785]

MSVVGFEMIPFTCWQESTTYPRGPQ

>Tb3.NT.15_5 [868 - 951]

MPRRAIGGKFFKKSFFPFSPFILEFLLI

>Tb3.NT.15_6 [1175 - 1252]

MRALLSLSVTCSFGLNAQKSFFFFFT

>Tb3.NT.15_7 [967 - 1311]

MAVGKRAVEKLSAILCCFFNCLDPSRYLYDIFMCTSCEPNHFILLMSFTPSLPPALTTKGRVPLQFLSVDASFIIPFRHVFLRIKCTKKFFFLFYLTLLLPFFALVFIPFPHRIL

>Tb3.NT.15_8 [1221 - 1415]

MHKKVFFSFLLNPFITLLRPCLHSFSPSYPVAAQTLSTCVSLPVDDVQICDFVIKEKSFCEPNSM

>Tb3.NT.15_9 [1547 - 1624]

MWWWRTSVKFIPATSFSFFYYYYYSI

>Tb3.NT.15_10 [1452 - 1754]

MTFCLIFFYPLLLLPSAGLFTQWQTTRVSFVACGGGEPVLNSSLRLLFLFSIIIIIPFKEEMKHQAQSKYLKLNRWVIGAKVLCVDQGTWRGQLSFIKNFF

>Tb3.NT.18_1 [110 - 364]

MSSLLSAADAKVAGKGYPPADNSKTYTEVPRHQVPAKGPIRSFLSLVSTEFALNTMDTTERVFTVLLCVAFISLVLWLLNLVFIS

>Tb3.NT.18_2 [461 - 541]

MCVANCSCYACASLAALSTFLLLPIAV

>Tb3.NT.18_3 [486 - 599]

MPVHPWPRYPLFFCYPLQCKKICALRSVRMYIRDSLVW

>Tb3.NT.19_1 [234 - 323]

MWDGNLSITNSNKKGSIFFCFCFWCFMKGL

>Tb3.NT.19_2 [383 - 508]

MMVVNCIVCPYVCIYIYIYTGACVCISEMQGNHEGEHNGMSN

>Tb3.NT.19_3 [414 - 569]

MYVYIYIYIRVLVFVSVRCRETMKANTTECLTDPPLRVACFTLLRLVLMFIV

>Tb3.NT.20_1 [146 - 226]

MKMIMMMMMMIFEYPHVSNYSHGGGTI

>Tb3.NT.20_2 [67 - 447]

MSYIIGHVMKKTVFVLVFAIVAASKSDEDDNDDDDDDFRVPTCEQLLPRWRDDLMCCAEKVRDKNTWCCVNNVSKSMWTALEECAKQSPRDFTTYPCSCLSQQMIEANGWTEEKERSIKNAEERESQ

>Tb3.NT.20_3 [389 - 490]

MVGLKKRKEALRMRRNGNRNKTSSELAAEEEIVD

>Tb3.NT.20_4 [605 - 712]

MKQIHQYQKRRVCPPLPLHLVTPLLFIAFTSLLILF

>Tb3.NT.20_5 [817 - 918]

MFMLLHVYKVGHYSLLSASATSSLFCARRGGEVF

>Tb3.NT.20_6 [833 - 937]

MCIKLGTIPFYPHPQLHHCFVQGGEGRYFSKFFSE

>Tb3.NT.20_7 [978 - 1055]

MMGRCDVMFLLLFLLFSIVVDTANFF

>Tb3.NT.20_8 [1074 - 1226]

MHTFDRFASLMLCTSYEIMCSISSMGFNSFDSSWLSPICKVLSTRFLHNRF

>Tb3.NT.20_9 [1120 - 1314]

MKLCVAYPPWALILLIVVGCPPFVKCCPQDFSTIDFDLLENTFFLYYGTIICHSYKRHLQTHYHY

>Tb3.NT.20_10 [1272 - 1427]

MSLLQTSPTNALPLLVVEYVCPLGEVLEVQMIVNKRIEFLHIYAEVDYIMRK

>Tb3.NT.22_1 [160 - 546]

MAALLPLVLSASLVVVAVVLSILACTVVAGSNVLPLFSLLLSFITPLPFLFFGRSESTFDDDGEINGFVVFLSGALAVSAPSLSIVLYHTGYSSLGAFLLSLGSQVTLLGAAAFLQSGERQDEEGNYDL

>Tb3.NT.22_2 [680 - 784]

MCMLYSYKPLFRSVLFFFLLGCVCACGCATFESVL

>Tb3.NT.22_3 [742 - 819]

MCMCMWVCDVRECIMNEEAGMGGGSR

>Tb3.NT.22_4 [1098 - 1208]

MPFVVVPFFFFDSRSLPLPPSIACELCFLSAKRTFIL

>Tb3.NT.22_5 [1051 - 1272]

MVVNFCALILLFLSLLCPSLSYHFFFSTRVVYPSPLLLRVNYASYPLNVPLSCDRFEDGATPALLTDTMSGFVF

>Tb3.NT.22_6 [1278 - 1427]

MAAVRLVYLFLLCLYFFFPLSFFLSFLYLVFPFLFLQIIVVFNISYNMGD

>Tb3.NT.22_7 [1502 - 1627]

MFRILTGSDIVVVQFTQGNEYIYVYISINICICVYVCAHRCV

>Tb3.NT.22_8 [1591 - 1668]

MYLCICVCTQVRLMWLNGRVARWCWE

>Tb3.NT.22_9 [1605 - 1739]

MCVHTGAFDVAQWTCRKMVLGMSSYGLLCLFSNTCEHRYCLFASC

>Tb3.NT.22_10 [1744 - 1911]

MCCVLQLYLFISSSHMGNTLLLRTSSWQSIHAMMACVTLICMGNAMSNQLNCLISV

>Tb3.NT.23_1 [265 - 339]

MCNSGPCDGEATAAVPQGRLFLRSL

>Tb3.NT.23_2 [189 - 446]

MRCVALSLSPWTVRPPKALQKGSTGYVQQRTVRWGSYCSRPSGETVFEKPVKPHVEGETRSSNRCSAKGLPNGFARENNEPHKLPE

>Tb3.NT.23_3 [349 - 453]

MWREKRDRAIDVVPKAFRMGLRAKTTNRISYRSEV

>Tb3.NT.23_4 [495 - 596]

MIVASFVVRVVYRLPASSLYCTYTSVSLLLPCGE

>Tb3.NT.23_5 [596 - 706]

MTICNVSPVLLCHYRMLLMRCIVHECYLTGNIASKRA

>Tb3.NT.24_1 [77 - 250]

MASFRTPDVSVVSFFFFYGRFFKSSCHSFNNVIYRFPEKPQNRTTIILSTNILFRPSI

>Tb3.NT.24_2 [168 - 377]

MSFIGFPKSHRTGQLSFCLLTSCSVLQFEKEDRVQCRSLRVLRLSHREVWLGEMVSIVCAILHFLNLFRG

>Tb3.NT.25_1 [193 - 327]

MMWSFAAPLSTTFLLCFFYRYQRHVLQLSRHIRNCVFRARAHARM

>Tb3.NT.25_2 [237 - 401]

MFFLPLSKTRSSIVTSHTKLCFSCACTRTHVKAEFADIVMHTLFYYTISRRCIPK

>Tb3.NT.26_1 [262 - 390]

MGDNPPIRIEESMTCCCCIPFVLCKWTVTSLLPSYLSSHNLLL

>Tb3.NT.26_2 [934 - 1032]

MCVGGFSMRLLTTCRTRTATVQLLLFLSATDPQ

>Tb3.NT.27_1 [469 - 663]

MCNMNFHHLIWLLWKNILKRPKQKLAPHLRNEGGKAERVVGGRRHQTCHFAKKFVECFVQISFKL

>Tb3.NT.27_2 [390 - 767]

MPPKKSSNAKAGKSEAVSEVSKINMDYVQYELPSPDMAVVEKYIETAKAKARASSAQRRREGRKSCWRAASPNMSLCKKIRGVFCPNIFQTLKGPVRDFVLGFVVTFFVMYVFFVALYFEKAPSKR

>Tb3.NT.27_3 [828 - 1028]

MKMKMREMSEEVTRGGRERKRKREGRRERNNFKIKQKKREVTTNRRQRKRFTVSYNLVTIFNEKCLH

>Tb3.NT.27_4 [1012 - 1173]

MKSVFIRRGTPFVFCFLYIYIYIYIYLFIYLYLYILSLSLCVPLPFPIPFSLIR

>Tb3.NT.28_1 [166 - 246]

MDSSVQVLQLNYSVMRMSFIREKQHLD

>Tb3.NT.28_2 [144 - 338]

MPETPQRNGFKRPSLAAKLFGDADVVYSRETTSRLTDFAGTFLVGFFAVYFTIAGYLLYREAALS

>Tb3.NT.28_3 [840 - 1079]

MIFHSSLFFISFFFPFFFHCFLRLHTFKNEIKIVQHFRAESQCAPFIKAKRMGECCVRSCVFVRVNEFSSFQNKNTKGRK

>Tb3.NT.28_4 [1036 - 1293]

MSFHPFKTKTQKGENNRDKGGEKSSNVLFIDNPLIYLNCVTEYMIYICVCTRACEISNTHCVILKFIPSFFFVFLKIIPLRLVIAL

>Tb3.NT.29_1 [179 - 259]

MKWNRQFLQKKSVIALMTQGWVFFVKT

>Tb3.NT.29_2 [406 - 516]

MMHLKMGAAGFYYFGPGEYSCKCVERGKRQHEGRVFF

>Tb3.NT.29_3 [471 - 545]

MCGTGEKATRGEGFFLKKSVRGTGI

>Tb3.NT.29_4 [564 - 671]

MVKLAEDGFMGRTEVEFGQNCLEETKRGCNLLEISK

>Tb3.NT.32_1 [104 - 235]

MCSAAYAHRQVPEERRKWPNIVADEHFAEEMTNVCTRRLKAQVM

>Tb3.NT.32_2 [353 - 445]

MMWLPSTISARESEARRLAHRGGVHGKLKVD

>Tb3.NT.32_3 [585 - 740]

MGSEREYKVVFFYIFGNVVVHFPPFNRADVFAQFPVRVSHEPRKRKRTGISG

>Tb3.NT.32_4 [740 - 928]

MKRPFSYNIVPLFLFISIYVVVSHRGTGKPRDLLVVILKRIIVVVLPVIFVILFFFHFVILLS

>Tb3.NT.32_5 [553 - 975]

MWCKRVRMIMQWGARGSTKWCSFIFLEMSWSIFPLSTGQTCLLSFPYGYLTNQEKEKERGYQDEKTLLLQYCAFIFVYFDLRCRFSQRDRKTTRPARSDSQKNYCCSATRHLCHSVFFPLCHPIIIVCFASAASTLPRSPL

>Tb3.NT.32_6 [996 - 1145]

MMLLSFAFLFQVVVTCFPSRMETAAVAGRRGVAASHESGRAIIFYVLLLF

>Tb3.NT.33_1 [201 - 275]

MDARLFSFFFLCERVRVCMMAPHVL

>Tb3.NT.34_1 [129 - 305]

MIYLFTYDHILTNNWAYSDYEVRGILKSSYGFIHFTFLFCRYFSAWMLVYYFTFLYLRV

>Tb3.NT.35_1 [79 - 207]

MHPKKCLRLHESKKKNCTFRAFRLLKMSVKADGVCVVKCTREL

>Tb3.NT.35_2 [283 - 393]

MFNHCFSFVHSFICIYIYIYIMPCGLRKTVLLFVYYS

>Tb3.NT.35_3 [351 - 488]

MWTSKNGLAIRILLVMMLCSALCCVALFDSFFFFYKCKLGSSCAVC

>Tb3.NT.35_4 [458 - 532]

MQARKFVCSLLTSLMLMWLLLCGGI

>Tb3.NT.37_1 [397 - 519]

MLEQGGGQVGGCDCALSFSFLFSFLIVASFDSYLLVYYASV

>Tb3.NT.37_2 [509 - 643]

MRVFRDNAGDLFLAPLQVRRLWKALAWDCQAIVLEHRCEVCAFIV

>Tb3.NT.37_3 [657 - 734]

MHLRVFVGDSGGRLGLPYRHPCSFSV

>Tb3.NT.37_4 [737 - 904]

MPQPETSWLNVTSTPLKNEEIINENNSNVFVYCRLLPAVLSGVCGVVAMVCVCMGP

>Tb3.NT.38_1 [69 - 158]

MTLFSPISTTYLCARYCPPALDVAKVWYIQ

>Tb3.NT.38_2 [133 - 372]

MLQRCGIYNKRLFLFGLFFRYCGIGLAPTILGAASRECLSSVGCVTSSDSPSVTLYQVESEARVYECCHALIFFLKMLSD

>Tb3.NT.38_3 [561 - 656]

MTCLLEYQLSRRPVVFVILVQREGGGIWIIAC

>Tb3.NT.38_4 [641 - 763]

MDYCLLIEMFSFLRSTNSRKKKNQVRMACLLVTSRCVYSCS

>Tb3.NT.38_5 [919 - 1011]

MFSSIEVITHISDCGCFDACVEVERREGWSF

>Tb3.NT.38_6 [1089 - 1328]

MATTDNLIRHYKQPVNRPVRAQFYCSVATGIEYISMNMYRDSVFCPHFYSPIEEFMWCYWGIANVSYFFFLTDLLLDRSE

>Tb3.NT.38_7 [1435 - 1551]

MELVILSPQENFQLLLLLLLLSFLKNIREVVLISFRLLM

>Tb3.NT.38_8 [1365 - 1637]

MVESSGTCLFTSLHIDVNGLGGGNGARYLEPTRKFPVIIIIVIIIIFEKYKGSGVDIIPLTHVTNDFFGRREVPSFSFTVPQSELEFTVRN

>Tb3.NT.39_1 [47 - 232]

MGLSKETARGDAAERITVCVCVWGRRRSLSSRGGRTNERQTPSKCTLTPMFPTCVSQRRNQT

>Tb3.NT.39_2 [178 - 279]

MHINTNVSNMCVTAPQSNLTLYFIERHAWLPRDT

>Tb3.NT.39_3 [295 - 381]

MQRFRLKVTIIIELPMGERFSWTVCCGRV

>Tb3.NT.40_1 [457 - 564]

MPFPSLTVTNERTLSDTITKANGQKVSLTYLVARTI

>Tb3.NT.40_2 [691 - 816]

MCFFYINFYEKIEFKAALASMYFSTFQRKEAVVRCCGASERD

>Tb3.NT.40_3 [831 - 959]

MILDDRIHMVGPLLPLLNGTSYKQCCDVEEPRYELTDLVVTIV

>Tb3.NT.40_4 [1016 - 1111]

MRHTAKNAILIQVSGEVMHRHVRCVTLLWRRD

>Tb3.NT.40_5 [1077 - 1205]

MFVVLHCSGEETESFGVWSPYHWEESTVQDILSRTSVRCYTRY

>Tb3.NT.40_6 [1127 - 1240]

MESISLGGINRTRHFITYFSEMLHKVLENVTVNSSLNG

>Tb3.NT.40_7 [1236 - 1310]

MADFTANDVGGAYEIKRLQRGKELS

>Tb3.NT.40_8 [1321 - 1530]

MCYYKERYRHMRIIFLMLMFCAWHTIKNVTKVGYPYTTHQRQFSILLIRFVCVWGGGGITVKCNVDPQLF

>Tb3.NT.40_9 [1511 - 1840]

MSIHNCFDSCSNRIYRHCVSPDSVIMSIRGIKEALDPRGTFERQSGMGKSSLCVMCTAQEVFEIKRKLHSNEENTNPKCVGIMKARSPPTPTEDGQIDEKLWGNVNHDKW

>Tb3.NT.40_10 [1791 - 1901]

MGKSMRNCGVMSTTINGEVGYRGHQSFYQYSTRYQWH

>Tb3.NT.40_11 [1837 - 2052]

MVKLDTEVISRSINTLHDINGTKKTTHGSEKEKKTVADVIDLFPYTVTSLLNASLDVRFFVRVNTKLYQLSE

>Tb3.NT.40_12 [2108 - 2188]

MPHLLEVQHTFTTFSRYCIFFFRYHSD

>Tb3.NT.40_13 [2052 - 2249]

MRTCIGFHDERLTRHYNMLCHIYLRFSIPSQHFPAIVFFFFDIILIREIGFRIGRQKILGKWVSLV

>Tb3.NT.41_1 [59 - 163]

MSTYDFGGGGRGYRVKFQAPPQVLQHVRVILLMAR

>Tb3.NT.41_2 [383 - 499]

MPHCRVHRAFGSYETLRELYRNLFSRSVRRDGFHTICTC

>Tb3.NT.41_3 [366 - 590]

MFQPAQCLIVGCTVHSVRMKHYVSSIGIYFPAVCAEMGFTPSAPVRNCFVYPPQQPFICVRCPNAELATSPALSV

>Tb3.NT.41_4 [556 - 723]

MRNWRLHLLYRFSGKSSLVGCHSPVISNLCVFSFSLLTLRCHFSKAVMSGIHPTSC

>Tb3.NT.41_5 [738 - 965]

MCPRFFVRQFILFYTTMPKNKLKADAGAMISDTQEYYPSLPQNSCLYRCFSHSQCLEQRPPVSRKRHFTRVSSLQL

>Tb3.NT.41_6 [984 - 1088]

MQNLEHYYALRLVATRYCSGSTVNRRDGIVVPICR

>Tb3.NT.41_7 [1063 - 1182]

MGLSFLYVVEEAPDFIPKETDPSAHTSKCGMEFSWVPFAR

>Tb3.NT.41_8 [1082 - 1297]

MSLRKPLILFLRKQIRLLILQNAAWNLVGFPLHGSALQSISYIIQAERRPRNRHAHPMIVLYVTKNWGRKLG

>Tb3.NT.41_9 [1365 - 1457]

MYLTGGMWWVTYNVCTSLAFLTVCFASKQVS

>Tb3.NT.41_10 [1402 - 1488]

MFAHPLRSSPCVSQVSRYRKPRISNSSRR

>Tb3.NT.41_11 [1563 - 1640]

MLCFPRILLCLVGWMQYHGSVNPCHT

>Tb3.NT.41_12 [1553 - 1666]

MMWHALFSAYSTLSCWLDAVSRFCKPLPHIILARNVAR

>Tb3.NT.41_13 [1836 - 1934]

MGVPFIIRIDGFLRSPPNSVCFVFYTFSCLKDL

>Tb3.NT.41_14 [2384 - 2497]

MPRRGSRSSASRLLPFLSYYSHSHHFVHWFRNVTCDCY

>Tb3.NT.41_15 [2367 - 2591]

MSPIAACPGEDLGHLLVGFCPFLAIILTPTILSIGFEMLLAIVTDQKNEVSDGCFQFSVEGQSPLFYMFWVLLPS

>Tb3.NT.41_16 [3511 - 3600]

MESFLLIRGTCLSHASRLELEYIPTICRPA

>Tb3.NT.41_17 [3588 - 3677]

MPTGMKEIVRPFCGDFPPSSWKIWGSQSVC

>Tb3.NT.41_18 [3656 - 3754]

MGKSVCVLKGLREVYCCMYKFMSESLRHCHHER

>Tb3.NT.41_19 [3836 - 3976]

MKSIYLQSGGAWFRRVERRWLLPKLTRCSTRFSSAGRAQQCPIWCFV

>Tb3.NT.41_20 [3979 - 4092]

MVLGFTSMSVQQSNTEPMASLTARLHVRRLFLGIILVL

>Tb3.NT.41_21 [4159 - 4284]

MEQGAGAQLLLPEQSAWRWRHLEVAGIAGDANPSFGASHLQS

>Tb3.NT.41_22 [4247 - 4423]

MRILHLAHRICKVRVEPPLCLRIGAGSFPSVRSCGLSWSGTRVISELRPPRDCALQQPT

>Tb3.NT.41_23 [4443 - 4532]

MVSFAVTIGGMPCGVKFVRFGCRGRGHRYL

>Tb3.NT.41_24 [5000 - 5206]

MDRTSISLMRGVRGRYSGGPALSLVSVLNKGSAPPLSPPFCAKDLGRGEGGSQPCSRCGCGVGWWLGLP

>Tb3.NT.41_25 [5239 - 5367]

MCSGSGLAIWPIVRRICALRCSLAVRHFFHNVQNVRIDDLIFG

>Tb3.NT.42_1 [300 - 374]

MPFVMCTYHSLVMAPCREAVFVLAT

>Tb3.NT.42_2 [764 - 943]

MQNHCSLGVHFAFPFILSSVGPSASATVRATQVEGRSPVSSTYIFVCFGCGGVLGCFHLK

>Tb3.NT.42_3 [910 - 996]

MWRCVGLLSFEVIERFVCRPMRCLLTYIW

>Tb3.NT.42_4 [960 - 1199]

MQTNEVSINLYLVNYFEIHHMGQHNTQIVRKGREHVNVASTRMTFATLPFGQHRKCLVPCILNLYGNRSALYRKTNIPFL

>Tb3.NT.42_5 [1124 - 1234]

MLGAVHTESVWEPECPIPENKYPFPLKLKLVETASAF

>Tb3.NT.42_6 [1153 - 1293]

MGTGVPYTGKQISLSFKAEVSRDRICLLMLLLSLLLQLPLLPLLLYL

>Tb3.NT.42_7 [1538 - 1636]

MGGDMELDHHTGVRLRDPSHYILRILQFIEGVK

>Tb3.NT.44_1 [137 - 355]

MTSCVRGWTKELRNGCCLGSERALSNVQSVKMMTHMCVLEGQTYGKQREGTKTRRKKGGQNRIYIEEGLLRTY

>Tb3.NT.44_2 [455 - 601]

MQLFFFWACWGLHTKMPSMIYIYIYRHVFLVPGSLTPLLPPLHALNRFA

>Tb3.NT.45_1 [125 - 268]

MIYKERKGKSISERGVTLLISLLFCFFSDTPSFGRQKKNRKKIGSHRN

>Tb3.NT.45_2 [274 - 477]

MAIQHYLREMHGEHWDTIKKQDKNKKQMPTGMTSPAGRGRSGCSIVFFSSVLALSLFLFIFFRFETLR

>Tb3.NT.46_1 [124 - 216]

MCFTNRRICDDRNYASPPLSTQALLKEIFFL

>Tb3.NT.46_2 [152 - 394]

MIAITHHLPSLLKHFLRRFFFCEVFTKCYCESYRCAAAVVQPVDIAALNYTVTCAKINRAYAWEGYMHLVECNVTEGSKLS

>Tb3.NT.46_3 [232 - 486]

MLLRELPLCRCCCATCRYCSFKLYRHMCKNKPGIRMGRLYALGGMQRDRRLKAFVNETIIGAYIIVYIYIYIYTYVCTRGVCVSV

>Tb3.NT.47_1 [53 - 160]

MRMYMLLYMLLYILMYILMYILVYIYIYVTVCHTLF

>Tb3.NT.47_2 [135 - 209]

MSQCAIRCFKHDGESLSSSSSLFYG

>Tb3.NT.47_3 [223 - 336]

MLVEINIPSHLHSVCLPAPTDVFRVRVSFRDAFVGFFR

>Tb3.NT.47_4 [284 - 388]

MYFGFVCRLGMLLLGSSGKGEEKYTTSFRFIVFGN

>Tb3.NT.49_1 [31 - 216]

MEGKRDCLVSVEQFFRRVHCSHAFCGTENPTAARRQVNRALVWMAGGDRASGKDGDAGKRGR

>Tb3.NT.49_2 [365 - 463]

MLFRMLFAAGRLKVASAWGCAFFFIIIMQAGKR

>Tb3.NT.49_3 [756 - 842]

MVLAPFCFFLEEKGETRPTLTCGVALGEL

>Tb3.NT.49_4 [668 - 859]

MRDDGFGKWRVAPSHPFFEVKKVCRENLLNGFGTVLFLFRGERGNTAHFDVWCGSGRTLTAYFQ

>Tb3.NT.49_5 [859 - 990]

MKAFPCSEFRREREARVGVWERGVQQRRLGEHPFKTSLQSFLPT

>Tb3.NT.51_1 [66 - 185]

MVYLVDDLARVRMLMAGVYAGLVVSSTYGFSVFATHLRET

>Tb3.NT.51_2 [121 - 219]

MLVLLYHRLTDSPFLRHICGRRNVSGEQNVLCR

>Tb3.NT.51_3 [527 - 655]

MVVVCCLVAVSRCLFHFSYFLLIGVPTAPFFFCVCVCVWKSCC

>Tb3.NT.51_4 [358 - 678]

MYFFFLCVAYTYVYIFFLLFCVLLYKFRNLVVDILLILLLCKGSGNEYIMFFKYVLNGCRVLSGCSLSLFIPFFVFPAHWRSHCSVFFLCLCVCLEKLLLTLEVWNL

>Tb3.NT.51_5 [732 - 827]

MLKNCLLMSLNLKGAWGVRILEGLRNKMMVSS

>Tb3.NT.51_6 [1076 - 1210]

MSGVFQMHYLSIVLWIHELFPPSFYPQGGAKAASLFSCYVLPCVF

>Tb3.NT.51_7 [1036 - 1230]

MPALLPLASSVNVNEWRVPNALPQYCIVDPRTVSSLILSTRWCQGCFFIFMLCFAVCVLVGGKMT

>Tb3.NT.51_8 [1191 - 1445]

MFCRVCFSWWKNDVNRLPAHFSDALHLPDAFISALFVAYTVLIFSDSRDLLSLLWFLNPDVGVDLAVTLLLQLIIRYDIVFLMER

>Tb3.NT.53_1 [95 - 199]

MDEMFTPFIFRDSWVTLSLFTAHGGALVSGKSRSF

>Tb3.NT.54_1 [85 - 351]

MMLSASSGQMELGLKRTLRINSYQTKRCGSCLLVGSVGGYSVGSLAGHFGGASRRFSFTSVSHALVSWGEKSIGRGHRFVTTTSAGEVE

>Tb3.NT.54_2 [680 - 766]

MASAFLRDRSAAPLPVVIYPTGCARPLKG

>Tb3.NT.54_3 [846 - 929]

MVPVFLCFPPRLMLTSTPVGEEEAASFG

>Tb3.NT.54_4 [958 - 1053]

MLPPVRLNVRLAASLLSDGEVCDGGVGAVTAA

>Tb3.NT.54_5 [1164 - 1253]

MCVKGWVQYCRSQEWSCFSPFRFVWLVWPG

>Tb3.NT.54_6 [1256 - 1339]

MEGGANCAGLALHTTGGRLPTPLAPHYL

>Tb3.NT.54_7 [1471 - 1545]

MARSARCAFPSRSLAPGRVRSRRRE

>Tb3.NT.54_8 [1448 - 1561]

MLSVFSSGWPDLPAAPFLLVLLHLVASALGGGSEGEWF

>Tb3.NT.54_9 [1555 - 1677]

MVLEAPLRGRKWYNRVFVLLLSFSVCHLTSHKINICAHLTL

>Tb3.NT.55_1 [60 - 239]

MCGGNTAKVFPRCNSPPSTPIWMEKDVQFIKTYIRSEQLDKVHGNYTLEGLDSGAFLALH

>Tb3.NT.55_2 [262 - 405]

MPEIEAALLCHLLWVMRGLSSKRIRNMCVCVCVCVCVCLCRVCTFSSE

>Tb3.NT.56_1 [167 - 322]

MRETSCEAGLNLFRPCVVIIFYALSWNPKEGNEYRTNHVSPRRTPLILFHHC

>Tb3.NT.56_2 [386 - 460]

MWFFVRNRQMCRVYAEIPNEGPIHL

>Tb3.NT.56_3 [476 - 640]

MNGVFVSSGAGRCAQESDSEEVMIKCHFDVLLVIRFAIIYIYMCVCVCIYRVGVI

>Tb3.NT.57_1 [108 - 188]

MLLTAARLRVCCPAMQVKVCNERVCGV

>Tb3.NT.57_2 [169 - 258]

MKEYVAFDSLLAFAITCVVFPLFLPSHTLC

>Tb3.NT.57_3 [340 - 468]

MRVRTEPSQTTRNSCLMGENERLTLTLKVETALSCGGTKHSIM

>Tb3.NT.57_4 [381 - 485]

MFNGGKRTVNADTEGRDSVVLRRDKTFDYVTNGSF

>Tb3.NT.57_5 [711 - 911]

MNGHLFSDFLRKEMMRTKTQQQTSKIFLLHGVGKQRNQNRALTIMQLQQITKVQIRAVKPSRNTINL

>Tb3.NT.58_1 [30 - 146]

MTPQTQIIDLSSSSSVRLQGKVLNLCSHTILLTRSTEAV

>Tb3.NT.58_2 [278 - 358]

MWTSPPFFVGVQNPSVGRKRCGVGAII

>Tb3.NT.58_3 [452 - 529]

MEFYVNQPVRFAFFFFFQWTRMFWSW

>Tb3.NT.58_4 [193 - 534]

MERGGSKHPASVDEKNRKRTVPAQPAPNYVDVPPFFCRGSESQCGAQAMWCWSNHLIVLADGAGSHFCSRTRVQILCGHLLHSFLPYGILCQSASTICILFFFPVDKNVLELVI

>Tb3.NT.58_5 [462 - 629]

MSISQYDLHSFFFSSGQECFGVGNLVGDVLSFRGRGGRQLLSIKTPGWVILPLCIH

>Tb3.NT.59_1 [144 - 359]

MERSAVEKFEAGSRGRSYSWVFARGCKWVHDHLVRVTATAADGLAINMDTDAGRRGSCASDVSLEVFSCRTV

>Tb3.NT.59_2 [232 - 426]

MITSSVSLQRPRMVWPLIWTQTPVEEGRARLMSVLKCSHVELSEEPVASILRRTMVCLNCSYSRV

>Tb3.NT.59_3 [475 - 654]

MGGGAAQLRRTPSVLSFFSSAPCVTQDRKCLLTTGTVQVLSPSPPPQWCVWASSNLPAVT

>Tb3.NT.59_4 [465 - 704]

MHPHGRWSGSAAANTIGPLLFFFRTMRHTRPKMFTNHGNGTGSLSLTTAPVVCLGLFQPTSGDMTCSVVRYFLPFYYYSV

>Tb3.NT.60_1 [508 - 612]

MPLVVGCVPVKEACLCLLRAAVCLLCTAAGILSST

>Tb3.NT.60_2 [479 - 691]

MGRIEVRFSLCHWLLDVSLSKKLACVYSERQYVYFVRPLVSCRRLDAADKKTLSPTVQHKVVSGIRPIAIV

>Tb3.NT.60_3 [525 - 734]

MCPCQRSLPVFTQSGSMSTLYGRWYPVVDLMLQTKKLCHLLFSIRWFQEFAPLQLFDRLMHGENGPQVPN

>Tb3.NT.60_4 [990 - 1103]

MRHRPVQWRRADRGFNAGGLHGPLVDRYLHVGSSSQFF

>Tb3.NT.60_5 [1078 - 1164]

MWVPQASFFDRTCGRVNFFQPVSQCGRET

>Tb3.NT.60_6 [1258 - 1437]

MRSRGSLSVEKRSHSLSAAAQGGAKSPFALWYINIVCRLLSHDLWMVTTVSPFSNGPMKA

>Tb3.NT.61_1 [85 - 162]

MRWPHRGNTKTAEISATQTIIVPQDL

>Tb3.NT.61_2 [208 - 351]

MSMVIVFNILDTSGECFFFSSLTTTRPSVTGFMAFVTQITVTIRHLYR

>Tb3.NT.61_3 [527 - 634]

MFSFFLNVCLEVRTNGKQTSFLIYYYVAWARINDLL

>Tb3.NT.61_4 [823 - 1020]

MKRLCVCKVPTISDPLALHVSPPLCKSVWSRQINVLLKGSVISKEFFFNTSFLFAVEKNHCHPPSF

>Tb3.NT.63_1 [169 - 261]

MRRTELTSFLLPHLPIQHRWDALRLLHGVWR

>Tb3.NT.63_2 [369 - 509]

MVRISGCLLLQFCCLSPPFMWLFVQICACGNLWNGGQISKIVAVEPF

>Tb3.NT.64_1 [187 - 300]

MECRVFRSKLKKGHVPTRLLNFFQANISGGYTRWISSL

>Tb3.NT.64_2 [36 - 323]

MKPSSTGCCYRAFDIGKRTAPLPAISPTGALRTLNASTTSGFFRQFGVAGNGVQSFPKQAQERACSHPTAKFLSGKYKRWIHPVDFESLNEPTLSI

>Tb3.NT.64_3 [332 - 421]

MSYVIFFYHVLARHNDIVTSLCFTRVGGAT

>Tb3.NT.64_4 [553 - 639]

MRTLVFHTFYFTSFHTGAVVGIMFTTVSF

>Tb3.NT.64_5 [507 - 722]

MHLISITTSMKSVVFHANFGVSHILLYLFSYWSGSWDHVYDGIFLKIYIYIYIYHLLTQLSVFRCLWRRFCC

>Tb3.NT.64_6 [888 - 968]

MWCSWLIQTCLCVVCWGFDCSACVIWQ

>Tb3.NT.64_7 [1064 - 1165]

MLISSVTLLGGSAVALFAIGEGFFFLECLFVSSL

>Tb3.NT.64_8 [1203 - 1283]

MSLYFDRGKRLKGICEDVFVGGEMLRG

>Tb3.NT.65_1 [24 - 143]

MICDGREPLYFGVGRLLFMMCLFVYTTNVESDSQRWRPLF

>Tb3.NT.67_1 [59 - 178]

MREVGSSSIQTQGARRNIKGRLLHGGSVVSATFFFSFLSC

>Tb3.NT.67_2 [181 - 306]

MLCLLVDITSFSFPPTLYFMAAQFKQSLGIKEGKSGVGVKRI

>Tb3.NT.67_3 [322 - 399]

MCFRRVSKRKKRKLKLKREVNIRHVA

>Tb3.NT.67_4 [416 - 568]

MKITCFVVFFLLFHVLVLHMFFFIMSWLCYLSGEIPSFSWLRTHIFPCRLF

>Tb3.NT.68_1 [157 - 282]

MHSVTNMPLNLFTSISVSVPPFLLQLVCRLRQQTEAVGHNAN

>Tb3.NT.68_2 [332 - 415]

MVHIHTHTHIHTQKICYCALTIIPLVSN

>Tb3.NT.68_3 [599 - 679]

MKDDLYFFGVGTSAEGVVGMLSTLFFF

>Tb4.NT.1_1 [94 - 222]

MRVCCPNRPTVNCISLRNILLIQQAERLLFSLSFYADMTVDRG

>Tb4.NT.1_2 [263 - 340]

MLTDYSCRVTLETLPGDHGIILKVFG

>Tb4.NT.1_3 [497 - 703]

MKYRNLSSIAVIDKYVLIKLNGRWCLCICWVARLPETWIKVTKGVVVCFYPCICSKCLCINAYYHCLSV

>Tb4.NT.1_4 [678 - 776]

MRTIIVFQYNQPFYTITSNSLPSIIAKQDNFLD

>Tb4.NT.1_5 [649 - 777]

MHLFKMSVHQCVLSLSFSITNLSIPSLATLFHPSLLNKITFLI

>Tb4.NT.2_1 [5 - 163]

MQEGRCKFKKLSFEVPNMSTSVHPSLTETTNLHRVVSLSGSFRTDGKRRECTK

>Tb4.NT.2_2 [1008 - 1115]

MSSDLNYTQPCDGKKKLYSSPKRKMLYHTHTKSKPQ

>Tb4.NT.2_3 [1037 - 1180]

MRWQKEIIQLPKKENVIPHSYEKQASIVGLEGNFQRNSKAIPKESHDA

>Tb4.NT.2_4 [1176 - 1262]

MRNYQLGKGYCQCEKSHREEPLEERGRSS

>Tb4.NT.2_5 [1042 - 1515]

MAKRNYTAPQKGKCYTTLIRKASLNSGFGGKLSKKFKSDTQRIARCVIINWEKDIASVRKVIGRNLWRKGEEVHSHYHKAENDTILKRPYVMIEHPIKRRALLTRAAEVVAMEHDIDNILLIQLGKHIYTIELQRSTVWYLRYVTAAVSKSILLMFLM

>Tb4.NT.2_6 [1535 - 1681]

MGGLSVREAGTYNREYLPKLSIAQHSLTLTRGNKGDLTESGGKKTSPFQ

>Tb4.NT.2_7 [1789 - 1932]

MMWKERKYQTGKSELPIVEKDAQIIKQPRIISTVSERHTTGWVMCYSL

>Tb4.NT.2_8 [1911 - 1988]

MGYVLFFVMEETQIWKKALIYCMTKG

>Tb4.NT.2_9 [2347 - 2451]

MSWKQNSYHHGGIWGTIIVDLQKMTQQCDFIGVLV

>Tb4.NT.2_10 [2547 - 2687]

MKTVVSRMMYVAAVRKNNSFLLATTATCFLNGNIIFVMMQYGPDATQ

>Tb4.NT.2_11 [2677 - 2787]

MRPNNLTVPPHTDTHTYALQLTQQWMCIFVEKKPVSK

>Tb4.NT.2_12 [2748 - 2924]

MDVHFCREKTSEQVASCCNCGHDDSGRFHAKMMGVVLLFINSEEKFFTASATCETAVHL

>Tb4.NT.2_13 [3047 - 3214]

MIHEALAIDGVCCEMERLILHGVSRDLRKITPWVSKEQVKGSTGSGLRTLSDTFAS

>Tb4.NT.2_14 [3142 - 3231]

MGVQRASKGEHGKRVKDTFRHFRFVSNRGG

>Tb4.NT.3_1 [98 - 181]

METFPHIFRYLHFTMGNNNNRTCTHLQL

>Tb4.NT.3_2 [81 - 260]

MHHKHLWRRFLTSSGIFILQWETIITEHARIYSYNRPDHALDFWLNIYINIHFFSDDNAK

>Tb4.NT.3_3 [281 - 430]

MEDQGHLCFCAHNILHMMSYSSLYLFVQVQLICLRYVWWCYLFELFIGCE

>Tb4.NT.3_4 [387 - 476]

MYGGVICLSCLLGVNNTFARDYTACVHETI

>Tb4.NT.4_1 [477 - 590]

MYFMHIFQKWKPVAIIFRYSIFMRDYFLLATASLCLQL

>Tb4.NT.4_2 [377 - 601]

MSYSNTRNKIRQTLLIVNCNMCSDTLPIWLHIPHVFYAYISKMETRCYHFQILNIYAGLFPIGNSEFMFTAMKFG

>Tb4.NT.4_3 [460 - 690]

MASYTSCILCIYFKNGNPLLSFSDTQYLCGIISYWQQRVYVYSYEVWMNELHSSVSKLIRMLFLFLFFIACSSRFVL

>Tb4.NT.4_4 [1097 - 1270]

MLFFMAMPHLFIFFAFPYPYICKYIIFSEMIVCSNWELLFEFYSQQKCLFILSYLICA

>Tb4.NT.4_5 [1237 - 1317]

MFIHTVIPYLCLSCCDTPTHDKICIES

>Tb4.NT.5_1 [123 - 251]

MIDGVLTCPSMSKGICTDMIINSHGFPSRMTDGKLLELVSSKA

>Tb4.NT.5_2 [217 - 327]

MESCWNLLAQRRECIHEKSRRWQCLWVPIRMRSWMRN

>Tb4.NT.5_3 [279 - 419]

MAMPLGTDKDEELDEELIQHGYNYLQGDDAYFPLGHNGEDDTHLCFL

>Tb4.NT.5_4 [410 - 556]

MFSLTPFITNTSNTCLLIRLMFMPLGGLRFMLTRSTHSKSIPQLRFAYC

>Tb4.NT.5_5 [337 - 561]

MDTIIFKEMMHIFHSGITGKMIRTYVFFDPIYYQHLKHMFTDKINVHATGRTPFHADEVNSFKVDSAVEVCVLLK

>Tb4.NT.5_6 [592 - 714]

MTHPTFSMRASSCDLIPLHNLYLMLLVWVINGEKRTCKGVC

>Tb4.NT.5_7 [1196 - 1318]

MQLLDHTCETVFFSWQCHIYLFFAFPYIHIYKYIIFFGMMV

>Tb4.NT.5_8 [1363 - 1443]

MFIHTVIPYLCLSCCGAPTHDKICIES

>Tb4.NT.6_1 [39 - 113]

MYISICFRFAFVVLATHCNRLVKHD

>Tb4.NT.6_2 [116 - 208]

MYEMTTCNNHRNKKKQTKTNHNQRIQRLVEA

>Tb4.NT.7_1 [180 - 296]

MLHILVAVRCHSYLMFVHPHLLNEHRIVSGVIFRIFEVS

>Tb4.NT.7_2 [247 - 408]

MNTALFLGLFSAYSRFREGLKQCGPPLIASELLTGRWLRASLQHLETPIKRREP

>Tb4.NT.7_3 [312 - 632]

MRTSIDSFRVAYRPMVTSITTTPRNSHKTTRTVGLCLSFSISTDMPLLIVLYLFFLFAGVLSWMWGTIIINLGSSSQKVPVVLFTTMRCQWCMRVRAFLFLSKRKKI

>Tb4.NT.7_4 [659 - 736]

MVFIIYFLKFRVHVWDCAACALIFHK

>Tb4.NT.7_5 [696 - 794]

MCGIALPVPLFFTSRSGLLNSELYMDPFAPPFI

>Tb4.NT.8_1 [265 - 339]

MCGPFNPLGAGISAKAKNVFVLKSK

>Tb4.NT.8_2 [210 - 389]

MLGCAGDFPPFGCLEVQLYVRSFQSSWSGDICESQECFCVEVKMKVTTVCCLLFNRFAHR

>Tb4.NT.8_3 [421 - 510]

MGRGGGDSGAGANNFCERPHLYAICISNSF

>Tb4.NT.8_4 [413 - 544]

MERWEGEGVTVVQALTTSVSALTCTPFASATLFDVWFCTRVPNA

>Tb4.NT.8_5 [513 - 701]

MFGFVQGFRTLDYAFVVMRWNFIFEGSPPQCLTTFARTALPSSAGLYIRPSKKKTSLTPKGLG

>Tb4.NT.8_6 [602 - 712]

MPYYLCADCASLECWAVHTAIQEKNFLDPERLGLSCA

>Tb4.NT.8_7 [890 - 1030]

MPRDGCGDEATLHSSDTITFCASGSIRMSQIFFFPVGASDAFVCFPD

>Tb4.NT.8_8 [1151 - 1291]

MQQCVKREKNRIGSSAKYVRASQFCTTSAGGCSGSEFAVPTRRLYGG

>Tb4.NT.8_9 [1303 - 1503]

MKTDSGKFCGDSRTVFPLCDLSLRRTVDNKILFFIECCASLSSFKLQYTLISEYVGFLFRYFLFLFV

>Tb4.NT.8_10 [1733 - 1846]

MVPFWCVCVCVCVCEANVWLPNDQAAPQCVFMICRVCA

>Tb4.NT.8_11 [1948 - 2163]

MPLKLEKKCCICHPSQVERQELPNDVNVCGVVVSLPAYYGRAACFHNAEIWCHGCNQLVEGDGKGQCLIQAV

>Tb4.NT.8_12 [2145 - 2249]

MSHSGGVITEERGNHYCGSLTFACVYELSLLLPTT

>Tb4.NT.8_13 [2063 - 2356]

MGVPRVSTTRRFGAMGVINLLRVMGRDNVSFRRCNNRRAGKPLLRLSHIRLCVRTFPPFTNYLKAHLSQTRRRFDCAMRLVFFGHGITNICTLVHEAN

>Tb4.NT.8_14 [2459 - 2581]

MVCSPRPERDMPFFGGCSIHRLRHQQSLLYPTVVLFIYLFF

>Tb4.NT.9_1 [160 - 273]

MVLAKCHGPFRAFSSIVPLLIRLVFFFVVLVTFVFFPW

>Tb4.NT.9_2 [299 - 394]

MQRVGSGDPNDGLVRWVTEGFGCVCVFLWKFI

>Tb4.NT.9_3 [276 - 431]

MYLPALDPCKESEAEIPMMVWCGGLQKVSGVCVCFCGNSFEGLNLSYEVDGE

>Tb4.NT.9_4 [415 - 546]

MRWMENSRVFFKALQIEVGEVNIIISLPLPDPFCVIPSFSCRGF

>Tb4.NT.9_5 [617 - 694]

MLAMKQALGLRHRRRHVSLTSRRHFE

>Tb4.NT.9_6 [804 - 908]

MRLRVFYHPMCSMGLSGSNTMTEELFFVIFLKKKK

>Tb4.NT.9_7 [1157 - 1243]

MHLLCVGVHVLPRRTTHYFLCMRSSGNVF

>Tb4.NT.10_1 [156 - 269]

MRKHRKHMGNTFFYVSGTIPNCFRGTPMQEVAVFSCPV

>Tb4.NT.10_2 [328 - 441]

MNMVGKGGLECLLSELTSLDISLSVSIESKTVFGERYI

>Tb4.NT.10_3 [474 - 770]

MKKTITTRTNVHKRNSRSILPVSFLNVKAMDLIDVTLMWGNHYSGAAVGISQVCSWKLEERCSRKEWFITALHCERCSQVLYLKGDVRYHPYLRSSLLC

>Tb4.NT.10_4 [924 - 1037]

MSETKHKTNFLSIIQIFHAPRAGNGSTPFFFLLLWPKP

>Tb4.NT.10_5 [892 - 1188]

MKARPTSYLLLCPKRSIRLIFYLSFKSSTHQELAMVLPHFFFCCCGPSLESLFEITPCRRKPFISSTTIFVLSWVSSTLSVKYRRQLCHNLLPLLRRFA

>Tb4.NT.11_1 [240 - 332]

MLLVERQNHFFLSFANLPRSDALVRALGSFD

>Tb4.NT.11_2 [154 - 417]

MPRCCFFFFSLVHRNQRQVSSDLIDNRMKCYWSSGRTTFFCRSRTYPEVMHWSVRLGPSINFVHRETILGIVSVVRWITSSEVFGGKE

>Tb4.NT.12_1 [38 - 133]

MCLFLYIYIYIYMCVCVFGVWCIGMERSGVEF

>Tb4.NT.12_2 [3 - 209]

MQHLIPRSNTNVCVCFCTYIYIYICVCVCLVSGVLAWREVESNFKQTRLGAVGFKKNSEGIVEGSLRIE

>Tb4.NT.12_3 [34 - 270]

MYVFVFVHIYIYIYVCVCVWCLVYWHGEKWSRILNKLAWARLDLKKIVRELSKVPCESSKRPPKWNPPPAPPPPHATAD

>Tb4.NT.13_1 [111 - 248]

MIMRVLDRSLVCFSFLRRRVAGSLLLLLLLFFWRRGGGVFKGLCMA

>Tb4.NT.13_2 [143 - 304]

MLLFSEAEGCWIIIVIVIIIFLEEGRGGLQGVMYGMRYMLMWGKDGEIGNGGGQ

>Tb4.NT.13_3 [454 - 546]

MRESCAAFVGDPNRHYPGIPIKRRDGFLYPH

>Tb4.NT.14_1 [242 - 436]

MQVLVWLKKKRCLNHEPLSFFGCSRDESFQRSSALMGRVSTVNSSKRSGGLRHGHLSVNYVISEQ

>Tb4.NT.14_2 [529 - 708]

MVNVSVMCGVADFFFALVVCPCVLGSYCFKCFWPVLWCLPVHHVRPTDVCFVLSSTYVKI

>Tb4.NT.15_1 [20 - 163]

MLVFSFKISSWRFTTESPVEQYGQTASILRWQHFSAIGRRNICLSSCL

>Tb4.NT.15_2 [84 - 206]

MVKLPLFCGGSILVLLEGVIYAFPPVSDASGDEVVFWGGGT

>Tb4.NT.15_3 [145 - 267]

MPFLLSLMLLVMRWFFGGGAPEKRWTDRVIDCVCALGFSPR

>Tb4.NT.15_4 [270 - 344]

MRHVMACAFVVLHMFTGHAECDILW

>Tb4.NT.16_1 [13 - 159]

MTCDPPYLLSDARYITSFVRLYHIIRAGGQLLWNITQAQRGGSIHHLCC

>Tb4.NT.16_2 [44 - 247]

MQGTLPLLFVCTTLSVRADSSCGISLKHNAAARYIICAVENRHRCVSQLHLFLYEWRDALVPRWSIWT

>Tb4.NT.16_3 [288 - 452]

MRESCAAFVGDPNRHYPGIPIKRRTLPGSGGGGTRVHLYSVFLLLFCLRFVLPFP

>Tb4.NT.16_4 [463 - 588]

MMDTCFMYSLGTCRHEAKPQIIGSSAAKNFFCSCSPWYCLMG

>Tb4.NT.16_5 [570 - 710]

MVLLDGLRDCFVTMDGMSFFKLLSLVLRRPPPTLSRLPLDNMGATTN

>Tb4.NT.16_6 [613 - 792]

MACRFLNCFPSSCDALLQPCLDYHWIIWALLLTSYGWSAHLKLGVTGARAQMSYVIISWD

>Tb4.NT.16_7 [720 - 848]

MVCSLKIRCYGGACSNELCDYFLGLKIWTALCHYCLTVVISCK

>Tb4.NT.17_1 [628 - 816]

MWALNLAAMQRNRIKPCANDDHQGGRERGGEERSLQCFVVRKTIMLCGRQCLKSLFTLRSIVL

>Tb4.NT.17_2 [735 - 863]

MLRCEENNNALWTSMFEVFIHATVNCPLAAAGIYIVYLGRDHS

>Tb4.NT.17_3 [928 - 1026]

MPMPVHWSLVMSVPNNKEQLFSCDICRCDFSCS

>Tb4.NT.17_4 [869 - 1048]

MSHRICCRGRRHDFFFHCRSCRCRFTGVLSCLFQITRNSCFHAIFAAAIFHAPEGRLICM

>Tb4.NT.17_5 [1083 - 1265]

MFDCNSCVITLIKLDFCRHHQKRSCRGASTKSNCLLRCEIPERRLRFHFDAFFFLGGGRCV

>Tb4.NT.17_6 [1042 - 1329]

MYVGYPQIMRLTRKCLTATHVLLLSLNWIFVDITKSVRAEVRLRSPTVYCVVKYPREDFVFTLTRFFFWGGGDVYKLVASVAQRGQLRCPVRNVSF

>Tb4.NT.17_7 [1525 - 1602]

MMWNGHLRRWYRGILPVVSAPLSHHY

>Tb4.NT.18_1 [140 - 319]

MPLFSITPMSHSGVGNVLPTLVLGWGEHLNASTLYVPCEGYREHPRAAHAQMFTRWCCHE

>Tb4.NT.18_2 [228 - 359]

MHPLCTSPVKDIASIRGLHMRKCSLVGVVTNETAQWVPVERHLR

>Tb4.NT.18_3 [319 - 414]

MKRRNGFLWSGTCGSAGEPFRPSISCESYAKG

>Tb4.NT.18_4 [332 - 457]

MGSCGAAPAVALESPSGPPLVANRMQRGNANLLSHTPATVFG

>Tb4.NT.18_5 [417 - 533]

MRTYCHILLQQFLGSASNTMTPRLRCCDHIVCVKLLHDE

>Tb4.NT.18_6 [767 - 865]

MMSLVRYQRDGLALIWGATLLHISLPTEANSDV

>Tb4.NT.18_7 [622 - 948]

MSNNVAVRCSRPISGNELRGCTARASCVMRWMSAVPISCVWIAEPSLLYDVVGSVSTRRFGAHLGCDVIAHIATDRSEFRRVNLLMCGVRSVTILRIGRRTERNVAKRG

>Tb4.NT.18_8 [1140 - 1298]

MCSPEGARLRYIDGGTVGFEVHLRGVPMFQGIISGYNGRVAAATVSLIGQEEG

>Tb4.NT.18_9 [1500 - 1685]

MGPKAPCVHSHTPLGASSDPLLLASTTAPADLCSATRNPLGSEFTSAVTADACCDEPASAGG

>Tb4.NT.18_10 [1306 - 1689]

MGSTSHPSKRWSGTGADVSLKILPGMQVGQPGSPLSKGMPSEGHAAVSEGNSVFPLMGMTVSSAIWGQRHRVCTVTHHLGLRQIHFYWLRQLRPPTFARPPVIRSGLSLLLRLRLTHAATSLQARVDE

>Tb4.NT.18_11 [1655 - 1765]

MLRRACKRGWMSDVQITHCIFLVVWWRSETTIFVKRR

>Tb4.NT.20_1 [683 - 868]

MAARLPAIQLLLLFALMRWMTGGIYWKRRRRCLVDSDQRRRPFEGVLVFVFHHFPFHHNSSQ

>Tb4.NT.20_2 [872 - 1042]

MGTFHPVSDGMIMMVMINIIIIILMVMMMMMMMIRTVSFMYTVRSRLLAPASEWRNG

>Tb4.NT.20_3 [520 - 1080]

MTQNKVRKSSSIISTIRGALSFTRKDEGSPAGSLGTPLIRSDPVPMCFRRPQSSHGCPAASNPTSFTLRSHAMDDRRDLLEEEEAMFGGFRPKEEALRGSSGFCISSLSFSPQLVAMNGDLSPSERWDDYDGYDQYNNNNINGDDDDDDDDSNCKFHVHSEEPPAGTRKRVEEWIKTQMPTVKPAAS

>Tb4.NT.20_4 [1038 - 1217]

MDKDSNAYRQTSRFMRRGGEGEKKKKKERKKVLLSPITLISMNIGFNFFIWFFFLVCACV

>Tb4.NT.20_5 [1441 - 1560]

MDNGWVVGEFFFFELILLIFFHFFFFSCCFPFSFFFFYFG

>Tb4.NT.20_6 [1569 - 1703]

MVIAFSSFFFNFFFFACVCLSGFVWPCLCLFFVCLFFFFFWWWWW

>Tb4.NT.20_7 [1922 - 2161]

MYELNNLYLFISTLKYTSTHMCVFMIGENRKERRKCLVGCKSSSFFLSFFFVFLYYYYYYFIYYFIPFPHPVLCPSEANN

>Tb4.NT.20_8 [2247 - 2327]

MKRKVDKLPSLYAYLSVFVFFVFCFVI

>Tb4.NT.20_9 [2281 - 2364]

MRICLFSFFLFFVLLFRVGRRFGRMKKK

>Tb4.NT.20_10 [2368 - 2454]

MLSCLLIRCRARVCVSLVLNIFYVLHSLK

>Tb4.NT.20_11 [2684 - 2788]

MFICGFCNSVSSFPVIVQFGYLYYPYVFVHLRALV

>Tb4.NT.20_12 [2830 - 2922]

MKRHIYLFIYFVYPVALSFVFIYFMFVVVVT

>Tb4.NT.20_13 [2760 - 3101]

MCLYIYAPLCNNSNYGSIEPPHPHEAAHIFIYLFCLSRSAFFRFYLFYVCSGGNVSYCHRFPVPSRTVLYCTVLYCTVLYCTRPSPHTHTYIYIYIYLSWNIFHTTNFAVAQWK

>Tb4.NT.20_14 [3095 - 3187]

MEIRKELAVSVLSSLFQHFITVRCVCVQFSM

>Tb4.NT.20_15 [3364 - 3456]

MCALCFLFPHVVHTSASLLRLLFPFICVLII

>Tb4.NT.20_16 [3691 - 3819]

MFFFSSIFAITYHHWFWRLVNYLFIYLLFCCAILLLLFFRSSK

>Tb4.NT.20_17 [3848 - 3952]

MWVSSENGEGELHSFKKKKRRKGEKNGNTKQHMSC

>Tb4.NT.20_18 [3867 - 3977]

MVKGNSTPLKKKKEEKEKKTATQNNTCRVELFSCLSA

>Tb4.NT.22_1 [19 - 102]

MYLLAQPASHWWSSMMVTWLPRSSACRC

>Tb4.NT.22_2 [72 - 248]

MATALLCMSVLEGRDIFCRLVSTFNVRRLLHPYGVVVALLRCFHGPSHTYACPRNQEMR

>Tb4.NT.22_3 [248 - 352]

MKWLVAFNCLLVEEGCLSHSAPQVIKEDVSDPALI

>Tb4.NT.23_1 [390 - 485]

MFGILNGCSSGYAALAHPHSIVSGGSGGVHVP

>Tb4.NT.23_2 [478 - 606]

MSRNSVGTKLSSTAYGNAGDLSVGSSDKLTIFYQGGAGLPRCL

>Tb4.NT.23_3 [794 - 868]

MRGDLGRGADELGGAGATDKMWVIL

>Tb4.NT.23_4 [688 - 936]

MLLCFWADGNARQPSSCGSSEQSPMMLRSHITWSQYARGSGEGGGRVGRCRRYGQNVGDIVEKVQSPGQSNIGCTNSYKRIYR

>Tb4.NT.24_1 [235 - 351]

MNQQFERQNYVMTAAVHTTGASHSSGGRIGGQKRTEKVK

>Tb4.NT.24_2 [389 - 535]

MGSYQLTHSLFLYRRSDSTLSISSIFLLQIIFAFQCVVRELARERRNIS

>Tb4.NT.24_3 [602 - 766]

MRRQEDKADSGRSFFYLFVCLLHPPDRPLPLIDSIIPWNYLYFTVWMRYFVHAGE

>Tb4.NT.24_4 [756 - 830]

MQASELCRFSVFAAPLVKCPELRDG

>Tb4.NT.24_5 [826 - 1029]

MVENNAGSSQTKWMMGGGGGVRDSEIQRSNEAKYLTSLLFYCGHSTGRLLRVSHSKRKDPCRPTHNMS

>Tb4.NT.24_6 [1048 - 1122]

MYIHTHIIFFLPPPPENAGKSRVTP

>Tb4.NT.24_7 [1097 - 1315]

MQGNHVLRHERCSFVHQLWRKQMMTTRCNFILSVHAQTVLEKGRQRNFRLCCNSIFYSIQVFNPIRTRCELLM

>Tb4.NT.24_8 [1318 - 1395]

MQNAYRCLVIMDISALGWTNAKKTGG

>Tb4.NT.24_9 [1376 - 1465]

MQKKRVDKLDEMHNFTYYYCCYLAGITYNW

>Tb4.NT.24_10 [1543 - 1848]

MRERSKEEEEENCCTYLCTVSGSAKVSVNLLLRSCNALSRFLYVVEVCVSRVLGLICLFGQCIHMSTCSRIMCDNKLCMGHSPFACMLSGRTFPLFPRDAFD

>Tb4.NT.24_11 [1838 - 2032]

MRLIKCSSVRALIRWNSTKRMKRNERTRREERKGRGFSIYRSSSVEWRLAPIFFFSLVYLFSVHD

>Tb4.NT.24_12 [1852 - 2169]

MLFCSGAHSVELNKEDEEERTDQKGGEKRPRFLDISFLLRGVATCAHIFFFTCLFVFSPRLRYDARHGRTYSIPHHQDWNVAKKKEEGRRRQSRRKGDQLVKGRIP

>Tb4.NT.24_13 [2182 - 2373]

MTTQTGGKNHPPVFFFRLPVRLRLLPWSNDDMNHSHWVTTGRNLHLHTQRCLLTAVMHENYNTN

>Tb4.NT.25_1 [183 - 305]

MRSCCKVFCWSSDHPNTKEYFLPLSFVSVGGTSVLAVMPHS

>Tb4.NT.26_1 [119 - 223]

MWLLLYSRKGHFFFLNTRLIPSQLFIRCLYLCSVP

>Tb4.NT.26_2 [241 - 375]

MVQTERQDYCAKGCEVTCAALPAPVKAYMRRDQRVCAGGVLRLHD

>Tb4.NT.26_3 [471 - 587]

MSLCPFEVCVRINRGLYFLARLRSSFLHAITYLFCLYLW

>Tb4.NT.27_1 [23 - 355]

MFSCIFVVKNGKLITLCRSDTCTKTPGYVGGHHIYMLRRGRAIVAFVAVTAMPISLPHSLLLSPVLTKMIRSKRKWLLYPFRFSMLRCAEGETAIKHRTVINPGSKMKQHI

>Tb4.NT.27_2 [355 - 456]

MIKGSRSISHWIRKKKMKMIVTIHLVGPIECLFL

>Tb4.NT.27_3 [598 - 678]

MRSINTCRCAAVLLPPQNLHRWRSGSI

>Tb4.NT.27_4 [704 - 880]

MKLSHCVPFYQILYHLVLLWTLSDHLQERNGVDFVVHKDNQDSYRFLLLETGILKKNTS

>Tb4.NT.28_1 [549 - 680]

MNFRLTLINSTLSDVFISLPQRTRRRGVCPTSVFLTSGCVFHVA

>Tb4.NT.29_1 [91 - 276]

MHNAAVQVEPLSWSGERKRACHTSGECSVETHRYMKMFVVLNAGTAGVRSSSAKVSLHVPIR

>Tb4.NT.29_2 [276 - 404]

MNRGGPHLLRSTTTPSDTFLHPLHIAWSRFYYCPKDFVAAGDV

>Tb4.NT.30_1 [322 - 471]

MRRGTRRKGQYMAVPNTCISMNMGVHTKFNRAVARGASRFRHCLSFTFFF

>Tb4.NT.30_2 [708 - 803]

MDEKPRVTLSSVILINGSGRSRATVQMLLLIG

>Tb4.NT.30_3 [809 - 892]

MSYEENTFAELHPAATIMLAFMFGCFGG

>Tb4.NT.30_4 [816 - 941]

MKKIRSRSSTPPLPSCLRLCLDALEGDSKVLRMYARGRPVCD

>Tb4.NT.30_5 [880 - 984]

MLWRVTVRYCACTLGEDLFAIEGCVCLGAVASRIL

>Tb4.NT.31_1 [202 - 279]

MKFTGKAKGNRDVHLRIKVKRRRELT

>Tb4.NT.31_2 [141 - 287]

MMRNYMYYSRGVLPKYFPLGHEVYRESEREPGRALTDKSEEKKGAYIKR

>Tb4.NT.32_1 [158 - 502]

MSGPRTQLFLLSIWLMSCAVSLSSLRVYDISHGPPRAGRTIFINVCCGGERPVRLWGKNVLWVPYNTLNLCSGRHMLWERSVKRKEVTGGSLVLTGVEETFTTCCDFGRHVEWVR

>Tb4.NT.33_1 [2 - 214]

MPGTKMVRLASNLICGALSNFFSFFGEGVKFIMFCSVNCYIRTIYVYVIFNFVVMFVGDVKALEEWFIFAG

>Tb4.NT.33_2 [177 - 269]

MLKLWRNGLFLQVNFFLIYLSFFLFLVIYCF

>Tb4.NT.34_1 [305 - 463]

MSIRTDICVSTPPYRAIHTILRPGHASASDFLFPKRFAGELRTKTHVTNSAAS

>Tb4.NT.34_2 [630 - 761]

MNGQIYVSSSMRRHQKAAVEQRYHQQLLISPLALQSIGLCSAAC

>Tb4.NT.34_3 [646 - 783]

MCRLPCAATKRQQSNKDTISNSLFPLSPFRALGSAQQHVDISSTRL

>Tb4.NT.34_4 [871 - 978]

MFPFTWAKECRKARKQRQQNEVKSKIATKIHKRKHK

>Tb4.NT.35_1 [83 - 229]

MSRCTNAFGVSFVLDASNPTPLVKFISLFSFPAVVLQNCGVSTSFFYDS

>Tb4.NT.35_2 [303 - 446]

MSYLGVPKGRQQREKRELYNSRWSDRLSVFEFLLTLTLYDVHTHTDTN

>Tb4.NT.36_1 [49 - 141]

MAHLPTVVKVVEVMTLDKGTVASYKCSRLCG

>Tb4.NT.36_2 [123 - 239]

MQPVMWIRKREREINKKESFTSTTADTEKVLFHELMDFK

>Tb4.NT.36_3 [361 - 444]

MMETRGVGNVLQSLKPYRIMRCFFFLSK

>Tb4.NT.36_4 [517 - 699]

MKFKWMTPFINPAPSHPHNAARLRIVSADPQRERRKFTVLSKKALTTAKVFYSGLTAIILC

>Tb4.NT.37_1 [85 - 198]

MSYYLLSDKLRVTKNKNDRLHESPLFNNLMNCANTADN

>Tb4.NT.37_2 [255 - 410]

MTFSLLLCLLVVVFFPVFFSFALHGNTSDADSSLPAVLFSKSLCDDRTVVRR

>Tb4.NT.37_3 [340 - 510]

MPTPLYPLFFSPNLCVTTGQSCAGNSQRHTVRKPAALGVLVQILKWSGCSPPPLSLS

>Tb4.NT.38_1 [187 - 276]

MKSYGTEEVAQSTRNVSKQSLACRNDFMRL

>Tb4.NT.38_2 [329 - 448]

MEITSCALQSSGQLLHSSSTKRREANVICVSPGVCLHLLQ

>Tb4.NT.38_3 [252 - 449]

MPKRLYAFVTPVASEFALKQLYSTLVWKSPRVHYNHRGNCCTAQAQNEGRRTSFASLQVCACIFFN

>Tb4.NT.39_1 [187 - 276]

MKSYGTEEVAQSTRNVSKQSLACRNDFMRL

>Tb4.NT.39_2 [329 - 448]

MEITSCALQSSGQLLHSSSTKRREANVICVSPGVCLHLLQ

>Tb4.NT.39_3 [252 - 449]

MPKRLYAFVTPVASEFALKQLYSTLVWKSPRVHYNHRGNCCTAQAQNEGRRTSFASLQVCACIFFN

>Tb4.NT.40_1 [116 - 217]

MNKLYDLIIWMAGPLSVMVSSVVRKKWLYDTPWL

>Tb4.NT.40_2 [211 - 321]

MVIARGKVPLANEVSGPVHLVRGVCDQEGCIIVDGPS

>Tb4.NT.43_1 [263 - 346]

MTGSSQYLLQFHICCTVKSFFFRGMGGW

>Tb4.NT.44_1 [250 - 333]

MRSTMVLCLFFLHRRCGRSVFGLQRIGA

>Tb4.NT.45_1 [284 - 370]

MLQHNTEGNAVVTQASSEEVMKKKCCGNQ

>Tb4.NT.46_1 [564 - 839]

MLSYRVKPLQDFSTSGIVNPSLRLFVCFLNSIHIFFLYVTHQYFQKKKTFCNEGFHWDLYNTLPSFIFIFIFPSFETKHIRSYSYIFMYLFP

>Tb4.NT.46_2 [718 - 864]

MKVSIGTFTTLSLLLFLFLFFLLSKQNTYAHIHTSLCIYFHDCVSFDTT

>Tb4.NT.47_1 [42 - 122]

MHEQMMMEREEFLSPLSHRCRTSYLHC

>Tb4.NT.47_2 [142 - 228]

MPHPLDSSVFRRPPPQCYGNFFGVQGTRG

>Tb4.NT.47_3 [194 - 295]

MEISLVFKEQGGDVCSELDGVKSAVCSRCDGGVG

>Tb4.NT.47_4 [464 - 607]

MRACSELGRKSGDDVSDAGKSGGVTAEGRCAKRHIFAVAADGAARAAK

>Tb4.NT.47_5 [550 - 678]

MCQATYFCSRCGWGRKSSKMRVEVSCEVCRPPCRGLADTSKRK

>Tb4.NT.47_6 [720 - 944]

MKRVGASLRTTCDDEPVRVTVMWGPPATTRLTLLCFLLHNNRGGVHGVGFQFTVWGLSTSISAGWRCTGGPSSRI

>Tb4.NT.47_7 [917 - 1039]

MHGRAIFENMISDVIYFLSPLFTVCLTCTDGTEVLHPNLPA

>Tb4.NT.47_8 [1005 - 1100]

MAPRCYILIYLLKGKLSQPLGLLPLVTLEVSS

>Tb4.NT.47_9 [1128 - 1343]

MLHQAVALRCFTRQTDLDPLLGSPFWYVEICVQSSEAFAVTISFPLFTVNIRYSYYSCSKRCRDCPAYLLTT

>Tb4.NT.48_1 [34 - 156]

MRIGCALMNVVESRCVVTGGKFWECVGLSQLENEGATKKKV

>Tb4.NT.48_2 [237 - 341]

MSFMFVSLADSPGVCDYGWVPFSTLYLYLHCISCE

>Tb4.NT.48_3 [196 - 408]

MGTAGRHGEAMLFLCLLCSSHWPIRREFVITDGFPFLRCICICIAFRVSSRGIRMCTQLVSCSGNISEGRR

>Tb4.NT.48_4 [487 - 564]

MNIYMLGNVSLPPSSFLRLLVEFLPL

>Tb4.NT.49_1 [214 - 414]

MHHKILFQQNPDSVICTLICGTTHETSAVGNPRSGSLLVSGFGYVCTLLTGDRQVEITGYAAKVFGV

>Tb4.NT.49_2 [462 - 575]

MGGGAKKQSTLRQILSGQTPQGVGTAVFSPLCKYCTMR

>Tb4.NT.49_3 [392 - 730]

MRPRYLAFRHLSITGSQSVVTFTHGGGGEKAKYAKADTIWTNSARRRYSCVFSLMQVLHDEVINNITCFPCWYRVRWLGNLDAEVLSILKCWRYVYGMTSPSAKHMAPKATPT

>Tb4.NT.49_4 [976 - 1098]

MKFLFLLSNAPKGPTPLPAQCSRELFSTTCCEYMHASPCVQ

>Tb4.NT.49_5 [1001 - 1132]

MLRKGQRPCPHSVRESSSVPPVASTCTHLLVYNRKYILIPLIIS

>Tb4.NT.49_6 [1298 - 1441]

MRSCLTTVHSVHDRSRRLIFFFFLEPSFSVSAGCPPSLFRCLAHRKSK

>Tb4.NT.49_7 [1483 - 1587]

MRMIRENRYVMRCWLCTCQSRRTSLGVMLSQHLAH

>Tb4.NT.50_1 [224 - 364]

MVRVSSAKKSGVRLGHPWGGGGGVTGVYVLLTVSCAFLRWSDPLLLS

>Tb4.NT.50_2 [417 - 491]

MVRFGALHSLPPRRSRVKKRLCNVS

>Tb4.NT.50_3 [755 - 931]

MKHVFSLYECLVDRKQLLCTVFDGFVTTTYSLTNLTPFRGTYPLNASLTKKEESWCLFF

>Tb4.NT.51_1 [339 - 470]

MGNTALLRLRHHTAQGSAVSCWDTAFHLSVPGHVPACHQQYHTH

>Tb4.NT.51_2 [259 - 753]

MPATSTWCQGPVPRIIGGSQEPTAFLSWGTLLCSGYGIIRHRDQQCLAGTPLFICRSLGTCQRAISSIIRTKMLLSGDVEENPGPSLRGMQWNCAGLSKGGGCGCAESCAHTGPLSTNQIVVCEFPVVPDRSMLHFIGVALHCRFASQWFLRLSLRRVALFPPSS

>Tb4.NT.51_3 [491 - 802]

MWKRIPARRCAGCSGTAPGYPREVGAVVRSPARTLDPFRLTRSSFASFRWFPTGACFTSSELRCIVVLLVSGSYASRFVGLRCFHRRLRIHSLVGGDRTQALAE

>Tb4.NT.51_4 [1137 - 1241]

MEHYSHTSPSHIICLCFTSHYYSIPSCSNGRNDVE

>Tb4.NT.51_5 [1078 - 1323]

MCTVWEVSLLYMLFVNSSFGWNTTVTPLLLTSYVCASPHIITPYHRVLMGEMMWNDRLGEKRKDQKGMTISLYSVWCTLLIL

>Tb4.NT.51_6 [1385 - 1471]

MYIFSLIYSRRNGECVHVSYYSPPLDPCL

>Tb4.NT.51_7 [1419 - 1511]

MESVSMSVIILHRSIRVCEKAVLAADHQYFY

>Tb4.NT.51_8 [1514 - 1831]

MEEGLASMLASSSVSATVPIKCHLNTLDHAFRAARGPKYQLAPDIFVVSASLSRASSILVPHHLVAYEETMMGLEPALCHASQGGRGSIQLLRIKGRLMRLVLSLR

>Tb4.NT.51_9 [1916 - 1996]

MGFVCQVYVFPAKSCRVQSWPISRNSQ

>Tb4.NT.51_10 [1831 - 2022]

MKCPRRETDLCKRGRWARSARSVAMQWTNGLCMPSLRISCKKLPCTVVANFSEQSMKEMATTRR

>Tb4.NT.51_11 [2054 - 2134]

MAPGQKPDSLLVDLSYAVILMLDSWVS

>Tb4.NT.51_12 [2151 - 2240]

MAIVGVVHWNMSEYADIVESAAAGTGGFLR

>Tb4.NT.52_1 [271 - 366]

MGSSRKEMMACLRISRCIPLLHGMKAITLIFR

>Tb4.NT.52_2 [168 - 629]

MSETNFTLLLVFTFVFLFLIIRVLLMFRPRRLLQDGEQQEGNDGLSPHQPLHPAPPWNEGNYINIPVAQPTTVVPPPPEAIILPGIPIARHHEMTTAAQLQQEEPPRSEIPNNPLEQATSHADVVYGHSAYIPRTTGVSLAASESKGGEVEKTL

>Tb4.NT.52_3 [535 - 708]

MWCTGIRHIFPEPREFRLPLVKAREGRWRRHCNWEAHECRALIHKCVNVFVLFIHLVI

>Tb4.NT.52_4 [762 - 947]

MGMHMATLVGSVYYSYFLNGCCCYRIKVSLLLASWWGLLFVFGACLLVRVFLAIGKTVGCET

>Tb4.NT.52_5 [817 - 1083]

MGVVVIALRFLYFSRRGGVFCLFSVLVCLFGYSWRSVKQWGVKHKTEGSREAKESARRLEALAPVFHTALWCGGTSTSSPPCALLLNNI

>Tb4.NT.53_1 [122 - 244]

MQREVHHHTKGGTALTTEEYTHTHTHTLPRLSSLFNTLSAH

>Tb4.NT.53_2 [261 - 341]

MKTKICVLIVRKEIYEGKFAELRVNSV

>Tb4.NT.54_1 [29 - 136]

MGKVESGPQQQMSQYSKCASSRVQRLERKWLRGGGR

>Tb4.NT.54_2 [167 - 247]

MSLSFELIIVRCRLPFLIVDAETARTR

>Tb4.NT.54_3 [288 - 437]

MNDSEVQKALQMLANELCVCVCVNNRQDPTAAPAATEKFTIEDIRRNVTV

>Tb4.NT.54_4 [698 - 991]

MRLLLNRNDLKLHKFVDCVCTDESNFSNHTPRNTLSVNEASLAVCFSMINLRFCLSIRSFPQAQTRIFCLFVCLFTYTCFCVCTWSTSDSLMWFPKLL

>Tb4.NT.55_1 [429 - 509]

MRSRWTVPDPTNCNRPAKLQTGGPVDS

>Tb4.NT.56_1 [76 - 222]

MRFTSRAGMEGRILPTAAKPCSCMQAMEFQQIPTQSSILFRFLYIAPWG

>Tb4.NT.56_2 [308 - 454]

MKRNNEFTVSTLQETTNALRDSVVVAELTVCRGRTHVLERTLTKRLKRA

>Tb4.NT.56_3 [569 - 739]

MVHRDGTHVMFFSQRPTACTAGEATKRHGFHPLLCVSVNPRWVSGIPKHFLTLLNRK

>Tb4.NT.57_1 [8 - 85]

MVPVMVMVLLAAAPTGCEGGIYRRCH

>Tb4.NT.57_2 [79 - 213]

MSLTVMTGSLCINFSRTGFITLRCPFRLQGSKSVYCSAIFASCLP

>Tb4.NT.57_3 [108 - 221]

MYQFFKDRVYHAALPFPFAGKQIRLLQRNICIMLTVTS

>Tb4.NT.57_4 [322 - 405]

MGAVYNIESMMFSWRNVRRRSTLRMRRN

>Tb4.NT.57_5 [368 - 556]

MCGGVVPCVCEEIRYLESCECLWFLSQWDASPLLALHISWVGTQGGGDSAISGMYPTPFQWFW

>Tb4.NT.57_6 [427 - 564]

MLVVFVTMGCISLIGFTHLVGWHTGGGGFSDFRDVSDTISVVLVKS

>Tb4.NT.57_7 [453 - 581]

MHLPYWLYTSRGLAHRGGGIQRFPGCIRHHFSGFGEILKSTTL

>Tb4.NT.59_1 [110 - 295]

MFGFDQERHEVSCEALAVEATVNACRGSSSKAIITLYMKLLQLRGVLITTNSEDEGNTCRFP

>Tb4.NT.59_2 [355 - 519]

MFSSEDAPSQERVPLRVIFGFRVIAPFTSIGQRDVERCAPTTNIRAVSVSIDAGL

>Tb4.NT.59_3 [509 - 601]

MLGCRKCNTSGYLGLRSFCWGKGQTSCPHPV

>Tb4.NT.59_4 [696 - 821]

MTTSVGTEPHCLLSVCYPGGTFTVANMIVHALLPFPDTSFFT

>Tb4.NT.59_5 [784 - 858]

MHYFRSLILRFSLDFGPRHFVKFVC

>Tb4.NT.60_1 [25 - 99]

MCGCVSLKHVCARVWKSQRRLTSCG

>Tb4.NT.60_2 [121 - 228]

MTVGAGRRFSNDELVSSYNPFDGSWHRMQCNVNSLR

>Tb4.NT.60_3 [255 - 332]

MCVIVGESTQTVGRSLKGINHSVDVS

>Tb4.NT.60_4 [460 - 618]

MLPHLKVLYRKETTRHVNVLRIYEVNPKRFFSLLWRRHVMPARLPPTVFFVFS

>Tb4.NT.61_1 [235 - 309]

MRKKNITPARHATPYRNLHRVKESR

>Tb4.NT.61_2 [219 - 341]

MYVLLNEEKKYYACTPRHAIPKFTSCEGEPVTYVKFLISEF

>Tb4.NT.61_3 [345 - 464]

MEKRMNEVQKQCGVHSWRFFFLIKKKKYIYIYAPRMSVKC

>Tb4.NT.61_4 [647 - 724]

MHKLFKLMNIRPTFMAHRVHKSECEG

>Tb4.NT.62_1 [212 - 325]

MIEEREGPAKCPLRFPLVKLVKGTSPEPCFFLLLLPSL

>Tb4.NT.62_2 [395 - 571]

MLQQQFNEKPIQFGHPTKLVDQAKKVTWYDFFFLFHPNGNLQVCCCCCWGNKSRLVCRK

>Tb4.NT.62_3 [562 - 753]

MPQVTFCFTVPVSLSLCVCFCVSVCVSSRVTSIVLHFYEAEATHLRLFFLLPFMESLQLSNTLR

>Tb4.NT.62_4 [357 - 776]

MDVISICFPLLQICCSSNSMKSPFSLDIPPNLWIRPKRLHGTISFFSSTLTVTFRCVVVVVGGTKVVSYAASDFLFYRSCVSLSVCVFLCFCVCIKSRHLHCVAFLRSRGNTFEVVLSATVYGEPSAFEYAEMNMVLIKL

>Tb4.NT.62_5 [787 - 927]

MEYMYFCHHIVGVCALFFFLRYTSSYFFLISYAFAYILSSLGVHLHS

>Tb4.NT.63_1 [185 - 271]

MGAARGAHKSLTPSERCYLVNQANNITSV

>Tb4.NT.63_2 [396 - 506]

MPQFRVVYSCLLLHVYLIYMLHFFRRELSPPSGSYRY

>Tb4.NT.63_3 [377 - 613]

MQGIVLDATVQGRLLLLAITRVSNLHVALFSKGVIPPLRKLPILKLLPYLHLEIMVHIRYTLYTCLCAWRCHCGWFFFP

>Tb4.NT.64_1 [36 - 326]

MQVATEKTTLGERIIQRRSSLPSLQLPSNSSIPPLSKNSSFAEGRVVSDPKLWTERFNDVFHVPPAHTPPEANCPRGPNKSEEKSPTPEVTCCGLFF

>Tb4.NT.64_2 [386 - 598]

MHVLLFFASPSISLSRRSLSACGGRAFQLRKGFVLFVMLAQQMRAGCFVCMCVFVSSLTSITYHLYISMEI

>Tb5.NT.1_1 [273 - 365]

MRNVIVCPDDLVGEHLKMLNFNEDNSTAARS

>Tb5.NT.1_2 [337 - 432]

MRIIQPRRGRSITETSEIQVAMCGSEASFEGY

>Tb5.NT.1_3 [540 - 659]

MTFVDVFRLENIRCRLSPRKEHILYTIATAKSTTVFWIVP

>Tb5.NT.1_4 [835 - 978]

MFCHRRRRIDCMCTLTPKAHRFHYTLFVGSCDDVFVNVTGNFQQRETV

>Tb5.NT.1_5 [1088 - 1171]

MVEKNVVEARFRTRKKSDMLHRVYLNDS

>Tb5.NT.1_6 [1438 - 1572]

MGYFFKDIPDFRAWWHSRGGEDRNPEVYPVIVLLWKGFRWHIMGI

>Tb5.NT.1_7 [1554 - 1697]

MAHNGHMKCWKRMTTTSLIGLPTLVSDKQKFTSSMPLCAYTSHCHMQN

>Tb5.NT.1_8 [1577 - 1756]

MLEAHDYHISHWATNISERQAKIHFKHATLCLYLTLSHAKLRCDTMRRKEKSKSHSKDAR

>Tb5.NT.1_9 [1763 - 1900]

MSYCVWNSQLTHGIYNNKSLSNMESRTQLLVVPKLLPERLSKILYI

>Tb5.NT.1_10 [1797 - 1913]

MVFTITNHLVTWNPERNCWWFLNYCLSDSPKYYIYNNIS

>Tb5.NT.1_11 [2138 - 2254]

MKLMLLIIMEKTRINVEFVHLYYAKEGRPRNTLWTNTLM

>Tb5.NT.1_12 [2371 - 2481]

MQLRSEIEDRWFQVESRRIPSDTAGADTGQYGAPETH

>Tb5.NT.1_13 [2498 - 2689]

MCDTGERTSNGIRSCGTTTCLTLPGNERNTVTCSLQCIHCSFSTELLGELTSRVGTRYCGKKTY

>Tb5.NT.1_14 [2777 - 2953]

MNGAYGALRLGTNMHRKAGSQPNSRKQALQAFSSSVKSRHYRSGGSHLFVEPVFSVVRM

>Tb5.NT.1_15 [2988 - 3119]

MGKGAPHGIHEIEESTHPGKWEEDPYNMLICQVSKNFAVAFGLG

>Tb5.NT.1_16 [2968 - 3123]

MRQQAFPWVRVLRMESMRSRKVHTLASGKRIRIICLFARSRRISRLLLAWGE

>Tb5.NT.1_17 [3194 - 3355]

MLVLPLCERKMPPQQMQMFHVVSHFQIRFLWRNVGVLLTKVQWHCHSGRYLRAP

>Tb5.NT.1_18 [3391 - 3495]

MDRELAFAPPYQGVCTVCVLQCVDGTHSTAQEILH

>Tb5.NT.1_19 [3461 - 3535]

MAHTPQHRKYYINEEHRFLLNSKCL

>Tb5.NT.2_1 [178 - 375]

MCTLPCFWFVLFLVSMPPTSRNVAVVSPLVGIDTGMQHTAGILIAVLIALKICAAHKRIRRTMLPI

>Tb5.NT.2_2 [454 - 573]

MLPTLVVWWTLLCVGQCEAMLSFANGEDICGVFGHLVVLT

>Tb5.NT.2_3 [581 - 688]

MKQIMWAQKLYLPFKWYALYVLDEVHLRYRSPFCST

>Tb5.NT.2_4 [625 - 717]

MVCVICFGRGALTVSVSILFNMKLTSLSVGE

>Tb5.NT.2_5 [630 - 740]

MRYMFWTRCTYGIGLHFVQHEINITFSGRITMRHSSL

>Tb5.NT.2_6 [766 - 975]

MCVHCMLLLIQLTVLMVYTFEYFLTSVAKLANVLHASVWDTLIFCYLLSFLNVKSTCFFSRILTVHHCFR

>Tb5.NT.3_1 [103 - 243]

MLSCHVAAFITVQFLDVRGVRPFLKFDKGYALFFGNVVSNDETQVRK

>Tb5.NT.3_2 [404 - 553]

MMTVVGGRRMYCALRCKAITVFPLQHISCGRYHRWLVFHFCMSYRLLLSL

>Tb5.NT.4_1 [119 - 199]

MINQKFLSGGPGGRGVWGGFQWVLKTN

>Tb5.NT.5_1 [100 - 180]

MLIEYGRLFMAFLCSRLSAVVCTCESY

>Tb5.NT.5_2 [250 - 339]

MRDLTASLTTFMGKLTVVSRVYARHANKRI

>Tb5.NT.5_3 [314 - 397]

MPVMPIKEFDSIILMPSFRYVTVPYTLV

>Tb5.NT.5_4 [450 - 569]

MSCWIHSFRCINAFLHFTPTINFMEPLRILRSVIQKDVFP

>Tb5.NT.5_5 [572 - 649]

MWVNSDLDCLVYVHIYIYIYIPLFWE

>Tb5.NT.5_6 [606 - 722]

MSIYIYIYIFHYSGNKLLLPSSSSLTGYPPMLFNVEASL

>Tb5.NT.6_1 [164 - 520]

MCARRSPRWNNILFIPQFFFSCSSGRLYHAFLFMCRVHRSRCDKFRRSHTVVKGRLRRVGTAVSYPTRYHDSEIYVQRCRNMSQRRSAFLSSYPQYVLWSDIHFNFLRLRSCFVHRRVL

>Tb5.NT.6_2 [450 - 527]

MFCGVIFILIFCGCVPVLCIVECYDT

>Tb5.NT.6_3 [664 - 774]

MKLRWTFANICLHAFGRFVRQGIVVGRTFCIFCWITL

>Tb5.NT.6_4 [701 - 814]

MHLAVSFGRELLWGERSAFSAGLPSNRQRATTCSHSQY

>Tb5.NT.6_5 [1046 - 1180]

MRNMWEGVVQIHPNLHNMSSNDGICKQYRSIPSSESKNKQFKPIF

>Tb5.NT.6_6 [1487 - 1642]

MLKNKKTFAHLFDLDCDVLRIFLFVMYVFVELFSETLPLKIRFFTVNIFCHT

>Tb5.NT.6_7 [1806 - 1976]

MECADGGGGNVSFVCFLMDFQTMETSGLYVPNIMKLTKSGYGRRNNSHGICSSTITF

>Tb5.NT.6_8 [2096 - 2197]

MYCGSFEWPTFQLVGRFTYWGAQLPALASVYLCE

>Tb5.NT.6_9 [2305 - 2472]

MNGLVKQLGRNNSRKGTGGRYHSLRLRCAFHAKWQTQKTTNMVEVQYNTCHWRTFK

>Tb5.NT.6_10 [2396 - 2506]

MRNGKLRKPQIWSRYNTILATGEPLNRVNPIVYLIGY

>Tb5.NT.7_1 [8 - 100]

MRRRRLPWKGRNRKGKSNNLHSLGRSSSGEN

>Tb5.NT.7_2 [300 - 395]

MECCDVYIFVVGGAVCLDLTSVAVSAAAWLGV

>Tb5.NT.7_3 [688 - 822]

MFLIFLWLCCGLTVTVHQASSPRKAERSVAVFAGSGLPPRRGRIQ

>Tb5.NT.8_1 [86 - 205]

MDEISCLTHESTRTWRCHYTAGRNYANRLRNSIRPYGHSF

>Tb5.NT.8_2 [90 - 221]

MRYLVSLTRVRALGGAITLLGETTQIVSETLYARMVTPFETHCP

>Tb5.NT.8_3 [361 - 516]

MAPSACRPRGWWLWPLLRFTLQSHCRCFPSSRGHRNVFFPFEGAVFWGFAVV

>Tb5.NT.8_4 [330 - 536]

MRGVGPNFLTHGAVRLPAPWVVVVAAPSLHPPVPLPVLSVLSRSQKRIFPIRGCRVLGVCCGLGSPCSC

>Tb5.NT.8_5 [598 - 831]

MVGLRGRVPTQGGWRSFFSFRSLGISMWRSWGIVGREYLDCAIRTDWVQVPLTKEWQPERINAQLCRSEMTTCALCGS

>Tb5.NT.8_6 [1015 - 1170]

MLQLICSPTNQKYCPCLYIFTIILMASLVKFLSTYGHVMSCWMWWWSVCVCG

>Tb5.NT.8_7 [843 - 1199]

MLSAKYRRPPVMAAPTAARWGGCSLFTRDLRCMRPFDGYVDPSTRVVPFLFSFLPLLNASAHLLTYKPKVLPLLVHIYDNTHGKFGEISVYLRSCHVMLDVVVVRLCMWVVHYYKCDQT

>Tb5.NT.9_1 [673 - 750]

MWAIGNSSCGVFMHWRKIVTACQHRM

>Tb5.NT.9_2 [894 - 995]

MNPSNKHNTCCNGDSIISCGKLRPEWYPVAISMD

>Tb5.NT.9_3 [1150 - 1236]

MHNYQLGKGYYQCEKSHREEPLEERGRSS

>Tb5.NT.9_4 [1289 - 1363]

MIEHPIKRRSLLTRAAAVVAMEARH

>Tb5.NT.9_5 [1407 - 1526]

MELQRSTVWYLRCVTASVSKSILFVVFDVRYNWEECAASV

>Tb5.NT.9_6 [1511 - 1627]

MCSLSVREDEIYNREYLSKLSMAQHSLTLTRENKGDLTG

>Tb5.NT.9_7 [1659 - 1796]

MNVRMISFSWIRQRMMQAASKRFDEVWSIMNSTDDVEREKVSNREI

>Tb5.NT.9_8 [1759 - 1902]

MMWKERKYQTGKSELPIVEKDAQIMKQPRIISTVSKRHMTGWVVCHSL

>Tb5.NT.9_9 [2171 - 2245]

MGPWNFTHHHKRNGGACALLFSFLL

>Tb5.NT.9_10 [2249 - 2341]

MGRELILSGKCSIDITGKMVQFLNPPESYNS

>Tb5.NT.9_11 [2373 - 2534]

MKGRGAPSFPLNSLWSFPLPLMGLLSLRSRKCAGECRRELCHTTQFSARSEQKR

>Tb5.NT.9_12 [2465 - 2659]

MCRRVPEGALPHNSIQCKVGTKKITTITADTLNNRRRFIPCIWPFGCLVIVKKEGDVPQRNVSIS

>Tb5.NT.9_13 [2662 - 2904]

MLSRSVIEGSCSYLMLRTLGLSIDGVLIFMVGWGRASLKRDIRTCAAAKLFIAALPEYPWCGSGPNALHPTGPKVANYFVW

>Tb5.NT.9_14 [2838 - 2957]

MVRKRPKCITPYWSKSCELLRVVREKGIYGIASPNASQEG

>Tb5.NT.9_15 [2858 - 2986]

MHYTLLVQKLRTTSCGEGKRNLWNCQSQRVTRGVTFHTALMFA

>Tb5.NT.9_16 [2923 - 3057]

MELPVPTRHKRGNIPYCPYVCLVEEGTGACCLLNSGRWQNRSVDA

>Tb5.NT.9_17 [3033 - 3113]

MAKSFGGCIKFAFATRQEVNCTPAYPF

>Tb5.NT.9_18 [3053 - 3142]

MHKICVCNETRGELHPGLSFLAATLGVFRV

>Tb5.NT.9_19 [3384 - 3566]

MSEKGILHRCLKQFRCAPVRSLLVPSAPIFFLWGAASAHEACAAQLICVPGVTPRVRSLLP

>Tb5.NT.10_1 [260 - 442]

MFGCLPHWGIHCGLQLRAVDPDILLSLAFMIVALSHRCFVFVSHYLPSKKGCAKIFLLFFV

>Tb5.NT.10_2 [454 - 540]

MATTSITIDSLSSLFCHICTYVFVARRLP

>Tb5.NT.10_3 [507 - 611]

MHVCFRSTTFAIIFREYNSKKKNYLVGRVCHSKLY

>Tb5.NT.10_4 [934 - 1008]

MLRGMCRNRFSNIIDFAAPLRLPQS

>Tb5.NT.10_5 [1136 - 1225]

MVTLAFICKGTLAQRTQHHHQGELRWPDGS

>Tb5.NT.10_6 [1218 - 1310]

MAHSMPRFAATTRGVMKDCRGGCPCSGELQQ

>Tb5.NT.11_1 [269 - 343]

MLNTFPPPGLSIHMLVLAAFHHQGA

>Tb5.NT.11_2 [503 - 580]

MLRKRALRILCRSHKFLLVGGKKITI

>Tb5.NT.11_3 [657 - 731]

MTFGDVRLFLPHGFCYTEWLRSRRS

>Tb5.NT.11_4 [670 - 789]

MCGCFSHMAFAIQSGCVADGHKNHHVMVLPLVSCVGRRDM

>Tb5.NT.11_5 [834 - 941]

MFLLAQKHRHYFLAVNLDASLLDSEHPPAAIYVSKK

>Tb5.NT.12_1 [21 - 176]

MMGSLIHATCPYGVLLAGCATFHPDEFAKLKAHGQNLKKKRKPYKATKRGKM

>Tb5.NT.12_2 [552 - 680]

MSKERFHNRRVSTNRLISNSSWSTSAKNQGISSPKRETDFFFQ

>Tb5.NT.13_1 [219 - 338]

MRGGNLTLTSAPALSSLAVMCFCCPLKMKGQKKKTLPLAK

>Tb5.NT.13_2 [396 - 476]

MSVECLPFLSPFFYIIVHITPLACDTL

>Tb5.NT.13_3 [361 - 582]

MKKFTHLRGVGSCLLSVCHSSLPFFILLFILLHLRVTRCKKGASHIILDPGWLARIAARCGTYRLSARCRFCWV

>Tb5.NT.13_4 [662 - 889]

MEEHLTTDQRVAGSNPVTDAVFFPSCSIALRRRRMCEEVCCRFYLKERGLGGPGGGGHMCWGPLSVLFFLFLFFFI

>Tb5.NT.13_5 [955 - 1152]

MRILTTSITCKSVRNHIGIGIFFSICFFFVFEKKQNGMYLLSLFSVICLMDKTIFVTLLRREGGKQ

>Tb5.NT.13_6 [1095 - 1238]

MSDGQNHFCYSAATRRRETIGVLPLRCCYWRYKNINANQLATLEEDFQ

>Tb5.NT.13_7 [1201 - 1365]

MQINLPLLKKISSSKINSALKNSVSDILHVCIYYIIFSPHVALTRIGMSTATRVS

>Tb5.NT.13_8 [1421 - 1537]

MRSVALWCNSFISCHALFTVTSYTRHKYIYLYLSIYIYA

>Tb5.NT.14_1 [442 - 525]

MQSRVYLFISFAVRRMTFKIGHINACIF

>Tb5.NT.15_1 [12 - 131]

MKNSVVYSSKRWCHNSTTVCFALDHILLNGLSCCSFVLGA

>Tb5.NT.15_2 [242 - 328]

MQESYCGTAVSFDLFPYPHFFYFCDLQNE

>Tb5.NT.15_3 [491 - 646]

MMQQRTSTSISCTYSLYRNLNHIISAPKWGEIRPPATRGHHFKQHLHIFFHF

>Tb5.NT.16_1 [185 - 385]

MVPYDAAMSGVPLSMVRSHGRSCPYAPTGTCKSVGRAVRISLPTPGSQFATSPHCSSNQQPTDSPGH

>Tb5.NT.16_2 [624 - 803]

MGVRFLSCFLCVCVVHGYVQHSEAVFFIFAAIVLCIPEVRHVHEGRGFGRERFSHCVYYV

>Tb5.NT.16_3 [397 - 888]

MHTAAQSPPGEVLGGGAMLTGYTPKRWWVFGRVWCATGGAVVAEGLPCSAIFFFFALRIISWWFAFPGLIISVPPRWVFAFCRVFCVFVWSMVMYSIPKRFFLYLPRSFFVFQRCGTYTKGGDLAGRGFRTVFIMCSRGALHLKRFSILFDMHFNLLIIREGCS

>Tb5.NT.16_4 [900 - 980]

MGRTREIKLDGPKLFLCFHVDSVLMFS

>Tb5.NT.16_5 [1163 - 1237]

MTDWWQSSGFSCLSSFFVSTVNCLV

>Tb5.NT.17_1 [423 - 521]

MKLPFVASFNGVYGKFFQSPPLKWKQRWVLCQA

>Tb5.NT.18_1 [99 - 263]

MNTKVVCFAAGGAAHMYEYSHPPIVQQTSLEVMYIPLHVDAFHNVYGYNLRGRQW

>Tb5.NT.19_1 [405 - 524]

MENILQACTIVRRSPTQLWVECCYGMKCRESCLPLAVACT

>Tb5.NT.19_2 [518 - 658]

MHIIHPVRSADSYCCLLGHVTFFIVMTLQYLRHFSSTGNVPLQNCVL

>Tb5.NT.19_3 [633 - 746]

MFLCKTVCSEPRLLLTTDDDVCAVAPDRAIRGIISVAL

>Tb5.NT.20_1 [545 - 694]

MLVFGSQLQHESLLMTTSLILHFLKKKEMATQKKKGKSTQIRVGRTSHVV

>Tb5.NT.20_2 [687 - 767]

MSSDSRGFTYIAYYYCSECDAVSWRYF

>Tb5.NT.23_1 [408 - 530]

MLQCSRDRTGLRHGELVALCCVFLGGCFPLFTLSIIQLGFA

>Tb5.NT.23_2 [416 - 553]

MFSRQDRFATRRVSCIMLCIFRRMFSSFYAINNTTRVRLTCKWSQG

>Tb5.NT.23_3 [703 - 933]

MLGHNYFAERIHYKDLAIQSGACGTDPRRYSFPRRTQRHSWVQIRRRVHVSVTGVGSSYWWQHLWLLCYYLRHSSHF

>Tb5.NT.23_4 [903 - 1043]

MLLLTALQPLLISEAAATIRLWQTEPRVRNCFCSLDCFTHVSLSPPR

>Tb5.NT.23_5 [1062 - 1328]

MKLERALRLGRGLLSRCGFPQPKESYYDMGHGGRFVFFAYSLIALPHIHFALHRLVIKDSKELPRHIFRVKKEVLHYLFCQVAASVMYT

>Tb5.NT.23_6 [1596 - 1760]

MPMRLIESFTIANMDYFLTGTWLEVTGKGSIYAIAQALDRFWNWSHENSAVYFSH

>Tb5.NT.23_7 [1505 - 1825]

MTVSLKKMCRFDAVFALASVSFVGKVYNKNNANEINRVLYNREHGLLFNRHLVRSYGKRFDLCNRTSTGPFLELEPRKFSRLFFPLSCYAQLCFFLPSTFPIWLAIA

>Tb5.NT.23_8 [1690 - 1881]

MQSHKHWTVFGIGATKIQPFIFPTKLLRSALFFSSFHISDMACNCLKGLPFAFRFFSYMCSYFF

>Tb5.NT.24_1 [53 - 127]

MEVVPTFRKSCLPSLRPLRHLTHFE

>Tb5.NT.24_2 [300 - 398]

MVGIPSQHGIGTPRDRRVLGTRLGFCMPCLATA

>Tb5.NT.24_3 [528 - 629]

MLRNCCLEQSPVHCTVHRGVFSSNQTYVRRAVCG

>Tb5.NT.24_4 [672 - 803]

MHGSPHGLPDCHSVGIFDHDVITLRCTNNIHRHRNRIRTCDSVV

>Tb5.NT.24_5 [817 - 1146]

MMLPVCVGLSMSLCTFLYTQHFNTACMFASTRCPLHGRVTFWHLDGLLFLSISTRSAGILCRKLLERDCRVLSREEGKKKNRQTYTASSVSICWLPPGCPLVCCTRGQTR

>Tb5.NT.24_6 [831 - 1151]

MRWFVHVAVHFPLHTTLQHRMHVRLNEVSFARSGHVLASRWATFSVHFNSVSWYFMSKAVREGLSGFKSGGRKKKKQTNVHRFFRIDMLASSRLPAGLLYPRADTVI

>Tb5.NT.25_1 [258 - 359]

MGKSFRWLAAVDAGALVWFRRCVRWCIRCCAMWS

>Tb5.NT.25_2 [4 - 432]

MLVSKQISPFLHAACNEFAALPLIPALFFFVSALPVKPTRKYMLKCALAVICTMGMRLGEKVWKHGSHCDQVAVMINVIHLVAGRWVRVFVGWPRSMQVRLCGSAGASVGVSAAVPCGAKSTVFFVLVVRATRRIGQLCRTFS

>Tb5.NT.25_3 [517 - 897]

MWAADATVCITPTVHTIPYHIISFSSTVIQTGSLVVNLLLRHVCTRLAVMRANCADLQQLVPPRLLTLFFPSPYSIGVGKYTALGVLHKHYLIYRFMPNSDVMRPGSQKEKSWKGVFCSPHGGISWM

>Tb5.NT.25_4 [1006 - 1110]

MGSTAFDVPSGTQWEVTPFHMLNVVWQLCPYLINR

>Tb5.NT.25_5 [1142 - 1306]

MASMIRALDFPWCCTISKGVGCCCATPSSLWSTLAKCSGPFAALLLLSRLLLPLR

>Tb5.NT.26_1 [167 - 445]

MSRYVPHWGRAKSLFKYVGILGLVVEPLCVRVPYRRVLSCETPFMALCVCDFGDALSRSDKGLVRPFSLRCSSFLHTFFYPIICFLFFNEDPY

>Tb5.NT.26_2 [327 - 518]

MRCPAPTKASFALSVCAVPVFCILFSTPSFVFYFLTKIRINSCVVCSLSPLSVLHEEPVGLFFL

>Tb5.NT.27_1 [326 - 433]

MLWSCATNRHEAVNTVEVINDFIRCLVFPHISVCEW

>Tb5.NT.27_2 [384 - 458]

MTLYGALFSRIYPCVSGEFSEVVMR

>Tb5.NT.27_3 [517 - 705]

MEPGQRGVCVCLCLCLCLDSISARVPGDHLRSCQVIVIFFRSFMFNKGVKSLLICEQCTVESV

>Tb5.NT.27_4 [870 - 1001]

MCSPWSGFAFSCCDDGGIRHSQLVVVLLAPVVLSNFCVLIISNT

>Tb5.NT.27_5 [1030 - 1146]

MGEGANVIRDANLRGLCYLISHLERFITILWFAYFSRGC

>Tb5.NT.27_6 [1243 - 1356]

MMWYLCRVNCSDGKGRQSNSFFGLTHNHWFVFVRFLNS

>Tb5.NT.28_1 [210 - 458]

MVMVVCAGPLALGFLEQPCFSLSARKKHFPMPAVFAVSHQVILRSAFVLELCSTSRAIDKCYSQIRVFKDKGDIFFAERTFSW

>Tb5.NT.28_2 [463 - 591]

MKHPESTLICAFRVCNWRGKGFTQWGKKFSSAHSLVYFVMSLC

>Tb5.NT.30_1 [240 - 335]

MQGLTGAVGVGIKERYGKKLNVILWAVGYCGV

>Tb5.NT.30_2 [392 - 490]

MMFASSNESIPEPKREAYWCNVWQATWCIITCH

>Tb5.NT.31_1 [49 - 135]

MFMIATSLLLCVVTSPSYRSAAQHVKRNR

>Tb5.NT.31_2 [256 - 354]

MIPFAHLQKGSRCICLVLLCCVVFCEELLLCGG

>Tb5.NT.33_1 [235 - 324]

MSFAAFVTFPDRRTEILLRPLSVFYIGNGA

>Tb5.NT.33_2 [408 - 569]

MQPQVDNGSRVGYRLCVSFSPLLPCAFFRFGWICLTRFGSVLKVPLFGCPDMVG

>Tb5.NT.33_3 [597 - 686]

MAGSLASGKIGSLSALCLTGTSFYFLCIVC

>Tb5.NT.33_4 [452 - 730]

MRIFQSTTSMRIFPLRLDLSHQIRICVEGAAIWLSRHGRVRVRHPVLWYGGFFGKREDWEFIRLVFDGDFLLFSLYSMLTVLQFHGIDKLMMK

>Tb5.NT.34_1 [73 - 210]

MGEKDSVAYELLLKEYWTRRELIRREEGRLHQLQRIILQVHNLLWM

>Tb5.NT.34_2 [236 - 325]

MFRIFFLLFLTLFQYTAAIQPERLAESISK

>Tb5.NT.34_3 [656 - 856]

MLTSQQLGTLRTYLRHRYFRCGNFLLLLSYPVVVTPFPFSIVSEYIILLYLTFEFLFVCLFVCLFFA

>Tb5.NT.34_4 [915 - 1031]

MTDFWFFFSILCAFADHGKYISPASWHFCWCLIHLTDCN

>Tb5.NT.36_1 [207 - 506]

MFCSTCVSLVGVISALMLLSYLGFVIIYVPVYRYLAITVLLLLLSYRLSVWRFLPTERAHVRRLAEKIVERRRAYALPTSEELRSDVGANALYVPAAPYL

>Tb5.NT.36_2 [674 - 838]

MAKRSVSFCIIIISSGAFLPWHYFCSALRQYVFFGSVLVLRRTTPVTTSTTDKLH

>Tb5.NT.37_1 [385 - 513]

MSLRIRAFRSICHEAGLKMEVRLWKKKKIKDEKAAAAQWLILF

>Tb5.NT.37_2 [498 - 578]

MVDIILAWLLVFGKFPILLFRPYLSWL

>Tb5.NT.38_1 [455 - 685]

MFGLHFNLIHDCCKLRARRLTHKLMRVCFLVPPCSIYRVNWNPRFAFSHFTFMNHFFQGKNLNGVGDLVLSFFFFSP

>Tb5.NT.38_2 [716 - 856]

MKSNQQMCEANDFHAQQTAITNAAEMFGTGVSYFVGIFGFGLAACLI

>Tb5.NT.38_3 [780 - 869]

MPRRCLAPVCHILWEYSVLVWPHVSSNGTQ

>Tb5.NT.38_4 [847 - 957]

MSHLMEPNDPSFFFFYYSKYMHALTPTCQSLSVKQRP

>Tb5.NT.39_1 [81 - 191]

MPYTFLCSASKQFIKMGIRIQIGSFYTCVSECQRGHW

>Tb5.NT.39_2 [310 - 393]

MYAVTAISHKRGLERTVCLGKRGICARV

>Tb5.NT.39_3 [413 - 514]

MLYMGRNRKLSVHLFLSFFFQLVLYALVCAPVLM

>Tb5.NT.39_4 [486 - 608]

MLWCVHLCSCNAIYWLIYVSCVSFTLRINIRLLLKVYWSIH

>Tb5.NT.42_1 [321 - 422]

MLVFQRCERISWTAAMTQEVTTLMSLHFYWQRRS

>Tb5.NT.42_2 [474 - 548]

MHVCELGPLQNIKYNSRVVAFYGFC

>Tb5.NT.42_3 [976 - 1056]

MSFFNSTQHLQLFDSFLSYFYFLLWFF

>Tb5.NT.43_1 [37 - 222]

MSSFPYTYHPFSITRLLGLLESMLVFVRIYLRGRSWTLSLFICSVLRPIFLIPLHARSAELR

>Tb5.NT.43_2 [200 - 316]

MQEALSCGRRNSNTLQTATDTIFNTMVVICCCHYDFYYV

>Tb5.NT.44_1 [109 - 252]

MTATSGAGNDFCGLLCSAHSLSLIADLMVLVVRLRSLGAVVGHSTLLY

>Tb5.NT.44_2 [267 - 341]

MQPLAVVVAFSFFAPWCFCSVASTT

>Tb5.NT.45_1 [94 - 174]

MTSLRVQPTCWVSVHLAVFAYRFLYCV

>Tb5.NT.45_2 [193 - 276]

MSECVVCVAVIRHHTLQFCMVDGRIYVQ

>Tb5.NT.45_3 [201 - 287]

MCGVCCCDPSSYATVLYGRWPYLCTVKKM

>Tb5.NT.45_4 [257 - 370]

MAVFMYSKENVSDVCNIIYWILCPSAVCGRDSMMFVNC

>Tb5.NT.46_1 [9 - 155]

MPARPRALPPPPPPRTGARHGRPTCHGNIPSQQTSPPPPQPGHPTATPL

>Tb5.NT.47_1 [146 - 739]

MAGFNIDGRVGHPGRLSGPPGRVPVPQSGHPNPVAVYWQAAPFPVTAAFAAPQLVGPPPVFFYRRGANHPGPAPRPPAPPPRRHLGVGPSPPPPRFRENRNLFSAGEVVGFIRLARRWDEKVAGRGAAHHARGPPNFEWPPEPEDPWKVLEDWSETRARWWRTSRADHRAPRRRRPSGPRSKKKNLRPLEKGVEVPRH

>Tb5.NT.47_2 [559 - 801]

MAAGTRGPLEGFGGLVGNPSTVVAHQSGGSSGTAAPPALRTPQQKKKFKTFGKGGRGASSLIPPRPRCLKKRRRCPPTRIF

>Tb5.NT.48_1 [294 - 425]

MGNTAVLWPRHHTAQGSAASCWDTVFHLSVPGSVPACHQQYHPH

>Tb5.NT.48_2 [179 - 562]

MRAAAMTATPWRRRPPPRGARVQYPASSGEAKSQQSSFHGEHCCALASASYSTGISSVLLGHRFSFVGPWVRASVPSAVSSALRCCCPVMWNSKKKDCQLTSFGESGVGGLSPHLLYSVQMRKYNKKL

>Tb5.NT.48_3 [430 - 579]

MLLSGDVELQKKGLPIDIFWRVRGGRPLAPSAVFRSNAEIQQKIIEGVLG

>Tb5.NT.48_4 [837 - 947]

MRSNVLFEGAKARLGGSGWRTPICPLHRRYGDACWGP

>Tb5.NT.48_5 [890 - 997]

MADADLPTASALWGRVLGALRRVKVLGKRKVWPSEK

>Tb5.NT.48_6 [583 - 1032]

MKKGDSATVARPIASQGSTVMADGRASGGKLSRRHEENFKKKKMTVTVFAAANTPAHGFLAAFSRRMKGHTSWTAGDQEPAAPEKCVRMFCLRAPKHASGGRDGGRRFAHCIGAMGTRAGGPKAGESSGETESLAFRKMKDGGAGAPGLP

>Tb5.NT.49_1 [153 - 746]

MAGFNIDGRVGHPGRLSGPPGRVPVPQSGHPNPVAVYWQAAPFPVTAAFAAPQLVGPPPVFFYRRGANHPGPAPRPPAPPPRRHLGVGPSPPPPRFRENRNLFSAGEVVGFIRLARRWDEKVAGRGAAHHARGPPNFEWPPEPEDPWKVLEDWSETRARWWRTSRADHRAPRRRRPSGPRSKKKNLRPLEKGVEVPRH

>Tb5.NT.49_2 [566 - 808]

MAAGTRGPLEGFGGLVGNPSTVVAHQSGGSSGTAAPPALRTPQQKKKFKTFGKGGRGASSLIPPRPRCLKKRRRCPPTRIF

>Tb5.NT.50_1 [34 - 273]

MVTVQRRSPAARVRKGASPCLTLPHPAPPCLAFKRNTKNLIPPAQKFAPGAVKRSGATLRLRGGGRRWPGWNPSDFFLRN

>Tb5.NT.50_2 [465 - 596]

MGNTAVLWPRHHTAQGSTAPCWDTVFHLSVPGHVPACHQQYHPR

>Tb5.NT.50_3 [350 - 751]

MRAAAMTATPWRRRPPPRGARVQYPASSGEAKSQQSSFHGEHCCALASASYSTGINGALLGHRFSFVGPWARASVPSAVSSALRCCCPVMWNSKKKGLPIDIFWRVRGGRLLAPSAVFRSTAEIQQKIIEGVLG

>Tb5.NT.50_4 [755 - 883]

MKKGDSATVARPIASQGSTVMADGRASGGKLSRRHEENFKKKK

>Tb5.NT.50_5 [822 - 1118]

MAAPVGGNSHEGTKKISKKKNDRHGVCGRQHARAWVFGCVFKEDEGTHELDGGGSGTRGAGKMRSNVLFEGAKARLGGSGWRTPICPLHRRYGDACWGP

>Tb5.NT.50_6 [1061 - 1168]

MADADLPTASALWGRVLGALRRVKVLGKRKVWPSEK

>Tb5.NT.50_7 [883 - 1203]

MTATVFAAANTPAHGFLAAFSRRMKGHTSWTAGDQEPAAPEKCVRMFCLRAPKHASGGRDGGRRFAHCIGAMGTRAGGPKAGESSGETESLAFRKMKDGGAGAPGLP

>Tb5.NT.51_1 [148 - 741]

MAGFNIDGRVGHPGRLSGPPGRVPVPQSGHPNPVAVYWQAAPFPVTAAFAAPQLVGPPPVFFYRRGANHPGPAPRPPAPPPRRHLGVGPSPPPPRFRENRNLFSAGEVVGFIRLARRWDEKVAGRGAAHHARGPPNFEWPPEPEDPWKVLEDWSETRARWWRTSRADHRAPRRRRPSGPRSKKKNLRPLEKGVEVPRH

>Tb5.NT.51_2 [561 - 803]

MAAGTRGPLEGFGGLVGNPSTVVAHQSGGSSGTAAPPALRTPQQKKKFKTFGKGGRGASSLIPPRPRCLKKRRRCPPTRIF

>Tb5.NT.52_1 [34 - 273]

MVTVQRRSPAARVRKGASPCLTLPHPAPPCLAFKRNTKNLIPPAQKFAPGAVKRSGATLRLRGGGRRWPGWNPSDFFLRN

>Tb5.NT.52_2 [465 - 596]

MGNTAVLWPRHRTAQGSTASCWDTVFHLSVPGHVPACHQQYHPR

>Tb5.NT.52_3 [350 - 751]

MRAAAMTATPWRRRPPPRGARVQYPASSGEAKSQQSSFHGEHCCALASASYSTGINGVLLGHRFSFVGPWARASVPSAVSSALRCCCPVMWNSKKKGLPIDIFWRVRGGRLLAPSAVFRSTAEIQQKIIEGVLG

>Tb5.NT.52_4 [755 - 871]

MKKGDSATVARPIASQGSTVMADGRASGGKLSRRHEENF

>Tb5.NT.53_1 [591 - 773]

MIGRRGNAYPCSLGELGLHWIFGGWAFHLFRWFLGPVRLLGTCTPLGGASGRALRWSSRVL

>Tb5.NT.53_2 [773 - 871]

MIIVFARVRRGIIDIRFFSSKTVPERITAIWGQ

>Tb5.NT.53_3 [911 - 988]

MEFLVGECESVVGVKLLPAQSFPYWE

>Tb5.NT.55_1 [104 - 193]

MELNFLGVITRVGGFTLVLTTMIWCVCAHF

>Tb5.NT.55_2 [79 - 216]

MVLLLYFTHGTKFPWCDYQSGWFYTGPHHHDLVCLCAFLNDPNRSL

>Tb5.NT.57_1 [259 - 351]

MHRLSMCIQQGGRVRLLQAFHVIICLPRLVS

>Tb5.NT.57_2 [354 - 515]

MLSDCACVCGFTPSCHAFRGWCDSICVCFMCFVSLTTLCGEVCYSYDECALCRV

>Tb5.NT.58_1 [35 - 127]

MLAVDVDGTTVLENRVTLFPTFAQERWWREG

>Tb5.NT.58_2 [382 - 462]

MLTALLMDVSNTSCGYSTTLRQYYEVR

>Tb5.NT.58_3 [314 - 532]

MSGIRVAIFSKYFAAGVTVGALLCSRHCSWMLATHHADIQQHYGNIMRYAEQVTSLEEMLGIHEKTTGDARNG

>Tb5.NT.58_4 [501 - 674]

MKRQRGMPGTDDARRQFPRRKKQTKLHVRWFHECSFSLLLFFFFPSYYLFPFNAYMSI

>Tb5.NT.58_5 [599 - 709]

MFFFSFVVFFFSLLLLVSFQCIHEYIGGNLFFWWGGG

>Tb5.NT.58_6 [1027 - 1110]

MHSTHPPLSLSLKRNNNKKAEAFVVFDT

>Tb5.NT.62_1 [57 - 173]

MQAPFHIDVHTPSSFKRGKKEFRGSGALWVTFQCWNGGL

>Tb5.NT.62_2 [140 - 220]

MGHFSVLEWRFVDVITRRSHNLKYLVR

>Tb5.NT.63_1 [104 - 406]

MNVIYRAFLPRRRHPISLRGVILVICILRIPELCLSRSIFISHSPSGESPSQRGWFVASPFYAALPLCFVFGNMADGCSRIYNAPGLVLGPLIFEFGGVGV

>Tb5.NT.63_2 [288 - 536]

MLPCLFVLCSGTWLTAALVFITHPGWCWDPLYLNLEGSACETFFLFICSVGVCIFLGIFFVSLLLIARVLYTLGMLPRFLIDG

>Tb5.NT.63_3 [560 - 718]

MRTRCSAESSCFCVYSLILLGRDGPLIRCCSYAVSCLRGRFLNYAVNASILRF

>Tb5.NT.65_1 [23 - 109]

MKKERVFTEMVTADNLTVDRFARCSSCKV

>Tb5.NT.65_2 [100 - 222]

MQSVIGANGEATAVNLRLIWRLPTACIRICAPLSFNSSGAW

>Tb5.NT.65_3 [679 - 759]

MSGDLQKPEALQRTFFVLQYFCRRSPP

>Tb5.NT.65_4 [850 - 933]

MKNLHATKPGDSNADGGPNWPTVQWKAE

>Tb5.NT.65_5 [863 - 1093]

MQLNPETATRMAGRIGQLSSGKLSDREFSCRVGFFFCLMGEMAALCTANFHKGPQCGTRGSTFASSPVGRHARVFYL

>Tb5.NT.66_1 [87 - 188]

MTLFPKPSPKDKVNIHCCVVENDVVKCLLLAVHF

>Tb5.NT.66_2 [518 - 667]

MSFKAMFSASDTEANIYICSCVWGITSPVCSVDFSCDLQTVERVGVAEAR

>Tb5.NT.66_3 [690 - 773]

MRCSCGVNNLSQMHFIFEKFSFRQQGND

>Tb5.NT.68_1 [86 - 271]

MRCYTPHTGVFDIICTHRCGITSTSLFGGEMTTWGFQMCHISMWHNALYQITKPYHRETLLC

>Tb5.NT.68_2 [274 - 348]

MYPSVAEFSYYNVRKSGCNNEKHVT

>Tb5.NT.68_3 [558 - 674]

MCARLGGSRPTSVVILRNAAFPPIICFFCIFQFFLLPQL

>Tb5.NT.68_4 [610 - 696]

MLRFPRSFVSFAYSNSFCCHSCSGFVQCS

>Tb5.NT.69_1 [248 - 475]

MSLFHLPVVDTPLFFFAILKYFVRRDCSSMFSIFCVHSCIVNKKSFHYGHRQVSAFLKTFCNLSEVPMLDIRTLVC

>Tb5.NT.69_2 [480 - 596]

MPFSTLVVPSLFYRTLFEEPDICMPLLSYIDVCALPFLK

>Tb5.NT.70_1 [95 - 235]

MPDRSPTSFCVVSRSMWDLTPGAAPLSDTGRYFLSRGVTCHSWHGNA

>Tb5.NT.70_2 [267 - 398]

MRVPNKTTRGRISSCEWRPAASAQLSIFFYIVNILWNWADVVVW

>Tb5.NT.70_3 [385 - 546]

MSWCGKLIGTAAFVPVVFPSIAVLDTLSSPIPFWPKLDTSPPFETDWVQRIGAE

>Tb5.NT.70_4 [856 - 942]

MFLYDLYGLRLFLSIAKNVRTLKSPVFTY

>Tb5.NT.70_5 [866 - 973]

MIFTGYGCFFLLQRTSERSSHRSSLTRDERSPKFSE

>Tb5.NT.70_6 [1016 - 1099]

MCMRVCGICCFSTAKCAHMRVHGFLIPL

>Tb5.NT.70_7 [1060 - 1200]

MRTYACTWLFNTSLKGDVLLKRNICGMFLLKISKVHHRLGSRRCEDV

>Tb5.NT.70_8 [1131 - 1331]

MWNVFAKNFQSSPPPRVTPLRRCVTATRQRESDFSLWISNRYLRGCEYIAGSAMSASLLPVISFAVH

>Tb5.NT.71_1 [17 - 97]

MIALGHIKGSNGRDRHCLLIAVNHLNR

>Tb5.NT.71_2 [396 - 509]

MTVVCVTSALSQRSRLSGNCNIWSGLLMVKYLDSDFSC

>Tb5.NT.71_3 [790 - 900]

MSGIIFACFVGLLHDLCTGALIFTISATPHTAMSSHL

>Tb5.NT.71_4 [837 - 911]

MYRCFNLYHFCHTTHGYEQPPVEPR

>Tb5.NT.71_5 [1128 - 1244]

MSECSVASGSWYSVYYGKARGCCSWFRVRYHTFFLFTQQ

>Tb5.NT.71_6 [1174 - 1293]

MEKLAVVVPGFVCGIILFFCSHSSRQCWSSLAIKKIFSLL

>Tb5.NT.72_1 [188 - 283]

MCTSQVWALNILFLREDQLGKWRPCGLTNTLI

>Tb5.NT.72_2 [19 - 381]

MPSRNRFPSLLRPGETIDHKLAYELSSRYKRKAPQLNLANWFYYTWSRWWHDNLNLYVYIAGVGAQHSFPQGGSTGEVETLRTHQYTYLSCRNAEWNRYRHYYHLRYILTYGLDFLAGGRQ

>Tb5.NT.72_3 [303 - 389]

MEPLSTLLSPTLYFDVRTGFPRRWSAVMH

>Tb5.NT.72_4 [478 - 552]

MCAAVTSTFAPAASFFFYVCVWVCA

>Tb5.NT.72_5 [414 - 581]

MAPRTHAHTRVCVKEPCQLFTHVCGCNFNFRASRIFFFLCVCVGVRLRLLNFQANE

>Tb5.NT.73_1 [7 - 309]

MDVNMGRGGQPTLNGCDTVICVLKGKRKETKKKNDITNAPPTGIRRRGKIDIITGRRYHTHIEIYKHVRECFFLFDPLWRRMVLLLRPVSKGGNEAMCAPA

>Tb5.NT.73_2 [206 - 376]

MYVNVFFCLIRYGDGWFCFSDPFPRGEMKLCAPPRNCCFFFVLLPWASAGLREFPTT

>Tb5.NT.73_3 [477 - 608]

MRGATKQIQHTHTHTQTILLLRMVSRSNRCGCCGSGGWHELSAS

>Tb5.NT.73_4 [611 - 730]

MLEYTCGKEEGLLSVEPQVRTLLWMRKRSRQGNVRDSRGW

>Tb5.NT.73_5 [727 - 837]

MVRQVSYLHLALALKHTHIAYMRMNIKKGGEECSKAR

>Tb5.NT.74_1 [43 - 117]

MERSHHTRNRLANIVVKKKREKDAI

>Tb5.NT.74_2 [110 - 247]

MQFNNCIFFLLPSCVMWSIGRDMSHLWILSLNRSSRGGSRTKKKIL

>Tb5.NT.74_3 [278 - 352]

MNKSECRRPIFYPFCNLVEGLKQFN

>Tb5.NT.76_1 [34 - 135]

MVDDSFMGYNLHIRLQKIRHIFLGISCYLRISCK

>Tb5.NT.76_2 [270 - 479]

MPKIVTPNVILGMLFRCAYATPGCACVCPVEGRCGYEDRLCHSGDRYLQVYTYVGIYLLLCCCFGGLVTV

>Tb5.NT.76_3 [494 - 631]

MVFQPCVHVILASLFLCVVSPITVFAFSMRMLNLSIPMICCRYSVS

>Tb5.NT.79_1 [64 - 150]

MCNNAFLQLFRGAESFNMHPKRPSTHALR

>Tb5.NT.79_2 [74 - 163]

MRFCSSLEGLRVLTCIRNGPQHTLCGSTPV

>Tb5.NT.79_3 [255 - 398]

MTFAGGPPFRTPCHCDTSLAHFRTVLWWPPPCRRPSCDSLRFKYSRAF

>Tb5.NT.79_4 [520 - 675]

MFDAPFFLKCCVTDSCRCKCDVDPALRGDDGRWCLWSSRLSGSLHGICTVSC

>Tb5.NT.79_5 [581 - 709]

MWILHLGATMVGGVCGAPVYLGPFTESVPCRVEMASPPPFKIG

>Tb5.NT.80_1 [927 - 1046]

MTVYWRVKIQMKCCRSECGSNNFFRFCISGDIRVEVDEHT

>Tb5.NT.80_2 [1249 - 1521]

MSKLLGGSSLSRDGGVKEVLMWCKRWREMRNYSNYPLSFFLFFLFFFVFSEWRYESFTLVALNFSPFEGVCYSKCRISTLSQIVYISSLFS

>Tb5.NT.80_3 [1458 - 1712]

MLLKVSHLYTFTNCIHILSLFLKTSVHELTVIPLIAFLRVKEPLFPSGAPQIIAQRRQQPAAREVSFSCIFYWLGFLHISRVTHF

>Tb5.NT.82_1 [243 - 560]

MSNTNSSGCMDVSIPVTLRGAALFCTHEHSELQSILCLICRAHLADHCVHCSTSSGFSLPTSDCLVVKGECGHKFHAHCIGDWGEQHQVCPACRKQWVAAERIARS

>Tb5.NT.82_2 [642 - 734]

MFPSFFFLVKLLYFFAISMNLFLPFVFALVE

>Tb5.NT.82_3 [739 - 819]

MFLFSRSLEEFGLCWKPIRHTLFSLLC

>Tb5.NT.82_4 [816 - 953]

MLVCVCVCVCVCQPKKIMKVYVRVDSSGNGVGKFTKKKRARECKSF

>Tb5.NT.83_1 [225 - 320]

MLRYWRIKRRLSAMTLHFNLFCTAIHHPQRTR

>Tb5.NT.83_2 [86 - 373]

MQSSCMRRCLSALQLYVLEQRKTVMSYKYVEDAVGVKAAFNRLSAEDVALLENKAEALRHDIALQSILYSNTPPTEDEMNYIENENDEDSDEVVTF

>Tb5.NT.83_3 [398 - 532]

MATMVQEEHRCVLASAQQNMLCGWGRREGRGGGQPILKNIYAVWW

>Tb5.NT.83_4 [427 - 543]

MRIGFSATEYVVWVGKEGREGGGSTHFKKYICGLVVKAR

>Tb5.NT.84_1 [40 - 150]

MLDSVRPPLTEVEWLTSVRAFMEFVRQKGPTPFQFDF

>Tb5.NT.84_2 [261 - 398]

MKTINGKLHISKECGFPHGKRRGASSSTDGVGDNIGSHRISALLSV

>Tb5.NT.84_3 [299 - 487]

MRISPWKAKRREQLYRRSGRQHWEPQNQRFIISLKVHVPKWLMKIFGCNRMQANCCFGACSNN

>Tb5.NT.84_4 [274 - 522]

MVSFTFPRNADFPMESEEARAALQTEWATTLGATESALYYQFESACAQMADENFRVQPNASELLLWCMQQQLTQMQEKIKRQA

>Tb5.NT.84_5 [612 - 704]

MMMESLNLWTNGYWRDTRNNRRQQLLLFPEW

>Tb5.NT.84_6 [643 - 786]

MGIGGTHGTTGDNNCSSSQSGKYERCFPGLTREGVAVWDKKRGKDALL

>Tb5.NT.84_7 [635 - 955]

MDQWVLEGHTEQQATTTAPLPRVVSMRGAFQGLREKASPFGIRREEKMRSFDGAVDYTKRRGPNQLITAYQYVFPATVSPDPVYRETRKIFVSWLMGPQNGIISEGY

>Tb5.NT.84_8 [789 - 992]

MAQWITRSAGVPINLSRLTNMCSPRLYHRTQCTGKHGKSLCHGLWDRRMELYQRGINDWENTFIRSCA

>Tb5.NT.84_9 [1028 - 1135]

MQDIRDAIEFPHKKDYIFLQSALKAKKQKQSILLNQ

>Tb5.NT.84_10 [913 - 1182]

MAYGTAEWNYIRGVLTIGKTLLYAVALDSNCLRQPWGENAGYPRCYRIPSQKGLHIPAIRSKSKETETIDSTEPVTMLHMRSSGALQCNL

>Tb5.NT.84_11 [1243 - 1344]

MNSNALHDSVAKVSQNKCLVDHFAVEKHKPCRRK

>Tb5.NT.84_12 [1377 - 1577]

MKIKHSATADQRMGRGFPQGGRVALLTVLHRKTAAKSINGVTIVIPVPTRNDPYILALASEANRIAM

>Tb5.NT.84_13 [1677 - 1874]

MMWKERKYQTRSLKLHIAEKYAQIMKQPRIIGPVSERHDRMSCVPLFVMEEKQNWKKAFVYCMTKG

>Tb5.NT.84_14 [1619 - 1933]

MMQAASKQFDEVWSIMNSTDDVEREKVSNKESEIAHCRKVCPNNEAAAYHWPSFRETRQDELCAIICDGRKTELEEGVCLLHDQRLKRAGMIMRHISHWVTFPSI

>Tb5.NT.84_15 [2317 - 2433]

MRFYWSFSLNMKTRQYGWDNESDTTIPSIPCYVKKTTIE

>Tb5.NT.84_16 [2554 - 2733]

MVLMRPNNLTVPPHTDTYTYTYALQLKKKWMCILIKKTPMNSPRVVATVTMMVTDSSTQR

>Tb5.NT.84_17 [2640 - 2816]

MDVHFDKENTNEFAASCSNCDHDGNGFFHAKMMGTVLQFLNSEKKFLTESGACETAVQL

>Tb5.NT.84_18 [2883 - 2957]

MHNIIIIKNMVGIATHIPMPWFMRL

>Tb5.NT.85_1 [388 - 504]

MNRLFAISCLVEHIPLKKFSFCCQPTSSQCVVVYFMTHL

>Tb5.NT.85_2 [669 - 773]

MSLNRVWSVTLPCAFNNPSFLTRCKCMNALMDFLR

>Tb5.NT.85_3 [743 - 859]

MYECIDGFFTVMLRFMHVIIYQPHEKKVLEHCFRGNEKK

>Tb5.NT.87_1 [130 - 210]

MMPTYQSGATLTIAGPLLVVLFICFLV

>Tb5.NT.87_2 [221 - 301]

MFHDLSVFKEAEGCICYLWLLLFMWPG

>Tb5.NT.87_3 [259 - 348]

MHMLSVVTSVHVAGVISFFFFRGNIHDADI

>Tb5.NT.87_4 [335 - 511]

MMLIFRLFPSGTSMDAGVSIQRMVKVYFAVVLCSRGSRRNDFIEVGFSLESPSLGWSSE

>Tb5.NT.88_1 [79 - 180]

MGFVQQHSVETNSMLSLHLVNTKAIPRVSASVLR

>Tb5.NT.88_2 [212 - 466]

MPVFKIMNVLDKIITRLYRSCGRWRWMPRFPTGPSNSNIRSSLDRIGGNEDILYQGGYGFVNTRDFTILLPNRSVVKLISPNNSG

>Tb5.NT.88_3 [466 - 555]

MMFNLKLSLSHYRYLCSMSGVICAVAMLPV

>Tb5.NT.88_4 [606 - 701]

MYLLVLERFCDKCRGGLAKPLYDLPLHVTGYF

>Tb5.NT.88_5 [571 - 723]

MVRSSYAHPHAGCTCWYWNVFVTNAGGGLLNLYMISLSMLRDIFRLMVHEK

>Tb5.NT.88_6 [716 - 820]

MKSEVRAFGVHNKSFLSKFQYYLIAFRKNKNSFFV

>Tb5.NT.88_7 [917 - 994]

MLEREYRPSKRLFNSVSSFTHLFLCW

>Tb5.NT.88_8 [1145 - 1225]

MRLSTTEILRRNVVVECWLLDGVSAFV

>Tb5.NT.89_1 [211 - 303]

MSDYTEKNIINTKLREKPVCAVPFSGCTIHS

>Tb5.NT.89_2 [697 - 807]

MRGGSKKKLKVTNSFKRWSCYTSSHIMFCRCQRVHVT

>Tb5.NT.89_3 [786 - 1082]

MSEGACYLIEREYTETFKLLFEASENGVVIGSSVIRGVPVTKTFGSIASSLLHTILEYHFKEVNASLVKIYFVLNLFPFPLVDACSSKGISRDVCLVLC

>Tb5.NT.89_4 [1033 - 1197]

MLVLQKGYRGTYAWFYVSLCGVRKYLIWNMSPSLALFPLLFCLRGCAFCLIPCQP

>Tb5.NT.89_5 [1113 - 1229]

MEYVALFGIVPIVILFTRLRILSDSLSTLVLYFPLVPAA

>Tb5.NT.90_1 [234 - 386]

MSLLLASACVNSQGTAAVFIPVVASGVLPPPFFYFCRGVGATTRQLSSIGL

>Tb5.NT.90_2 [209 - 562]

MTLRVACAYVTFVGFCLRELTRNRCCFHPGCGVWCVTSSFFLFLQGGGCHYTAALVHWSVVVRFYLIFAAVVVLSALLLAGNKTLGAHSSSMRSKSCLLQYFEFWGSGERYVRQIKEK

>Tb5.NT.90_3 [691 - 786]

MNTPFTLMQALHGKVKSMEGICLLIPLYCVGG

>Tb5.NT.90_4 [786 - 884]

MNCEKHYACSYLFLLTLTIPCGAVEFITIDETA

>Tb5.NT.90_5 [1072 - 1155]

MLCHVFHIFFGGVVKFIFFLWTVFVASR

>Tb5.NT.90_6 [1082 - 1168]

MCFTFFLGGWLNLFFFFGLYLWLHVNLYF

>Tb5.NT.90_7 [1433 - 1543]

MRGGSKKKLKVTNSFKRWSCYTSSHIMFCRCQRVHVT

>Tb5.NT.90_8 [1522 - 1818]

MSEGACYLIEREYTETFKLLFEASENGVVIGSSVIRGVPVTKTFGSIASSLLHTILEYHFKEVNASLVKIYFVLSLFPFPLVDACSSKGISRDVCLVLC

>Tb5.NT.90_9 [1769 - 1933]

MLVLQKGYRGTYAWFYVSLCGVRKYLIWNMSPSLALFPLLFCLHGCAFCLIPCQP

>Tb5.NT.90_10 [1849 - 1950]

MEYVALFGIVPIVILFTRLRILSDSLSTLVLYFP

>Tb5.NT.91_1 [234 - 386]

MSLLLASACVNSQGTAAVFIPVVASGVLPPPFFYFCRGVGATTRQLSSIGL

>Tb5.NT.91_2 [209 - 562]

MTLRVACAYVTFVGFCLRELTRNRCCFHPGCGVWCVTSSFFLFLQGGGCHYTAALVHWSVVVRFYLIFAAVVVLSALLLAGNKTLGAHSSSMRSKSCLLQYFEFWGSGERYVRQIKEK

>Tb5.NT.91_3 [691 - 786]

MNTPFTLMQALHGKVKSMEGICLLIPLYCVGG

>Tb5.NT.91_4 [786 - 920]

MNCEKHYASSFFFFLPTLTNPWSAVHKQLPVRLVCCRLPMSQAKA

>Tb5.NT.91_5 [805 - 984]

MRLLSFFFCRLSQIRGQQCISSCPCDLFAAACPCHKRKLRSFSQFFFCPIYHMKVEGKCQ

>Tb5.NT.91_6 [994 - 1116]

MVCRSVRWCVVLIKYVFLFLCWRTVWRMPLLSIFAHRYSSY

>Tb5.NT.91_7 [1037 - 1153]

MFSSFYAGEPYGGCHFFQYLHIGIALTKLTWKAFLNIVE

>Tb5.NT.91_8 [1126 - 1320]

MESIFKYRRIVLVYFFLALAFLPTISLGPIIFRVELAVFSTFSSLLCTAVQFTHVFIGNGALLRP

>Tb5.NT.92_1 [198 - 284]

MSSFINTPGRNMRTFGGSPIRHRLGGGQS

>Tb5.NT.92_2 [8 - 313]

MTDTFEGILKEIHSLEEMNDMYSYQIASLMVEVKENGGDTKKEEEVLELKRRLSENLKCVSELDVLFYKYPWKKYAHLRRKSHSASSGGGAVINFPDQGKTP

>Tb5.NT.92_3 [540 - 647]

MAMQNNDETLLIGYLTPLFYFLSRPIECVTSIALTT

>Tb5.NT.92_4 [620 - 724]

MRYFHRFNHVITFVFPDFLYSYFGCFPVSVLINLF

>Tb5.NT.93_1 [111 - 191]

MEGNLKLLALRADLEAGRYASGEARSC

>Tb5.NT.93_2 [198 - 290]

MKEELEISQKLCQALRLGGVFGCLGAGKSTT

>Tb5.NT.93_3 [230 - 436]

MPSVTARGCLRLPGGREKHYLVMELPLQTAVHTYVVLSVACFLQSTGVRSIISSGVVWTGRAVNRGMKG

>Tb5.NT.93_4 [751 - 843]

MEEHCNVQFPKYCYVNASRGRGWRSYLLKSD

>Tb5.NT.93_5 [1013 - 1096]

MHNTCGYVGTAAYFEWQRSSLFSYPQCD

>Tb5.NT.93_6 [870 - 1157]

MHDHLNMKLLGTLRSFPYGPPRALMLISRSGTLCELHLVCHGDNSHCQCTIRADMWGRPPTLSGSAVLSFRTHSVTETAAMNHFGTLHKTPVSLPC

>Tb5.NT.94_1 [218 - 316]

MRTKDYRVLVCFTISGSRKFVLTASMGRGTVIM

>Tb5.NT.94_2 [109 - 450]

MTALSPLCLRTRLVVMCVCMPVPTAGAGERYRSKSNYAYQRLSRFGLLHHIRKQEVCVDCIHGEGDGNYVVAVDIRLRGSIWKYGCIDAVIAYALVVYLQSCATDRGKFVGLSL

>Tb5.NT.94_3 [359 - 649]

MDVSTRSLPTLWWFIYSHVPLTAVSLLGFHYSRIVSFPSQHNIRFSKRGSLLCVKAAHVRNMIRAGINLFIMNISHLNAVDTIAAPVTVLRSISITS

>Tb5.NT.94_4 [666 - 755]

MATFLHLRAAPEAMETFSSSYCYKCCNACW

>Tb5.NT.94_5 [737 - 814]

MLQCLLVGVSGIPLFEDSNGLLCSFP

>Tb5.NT.95_1 [31 - 171]

MFLSLIYEVSIAEGAFSFFRKKKVSFTDMPTATPQTILDVEWTKALT

>Tb5.NT.95_2 [153 - 302]

MDKGLDINKSFCIKEGTNPIFNWKWFVAQKLHVACRRYVEIGKWGVSSIL

>Tb5.NT.95_3 [265 - 390]

MLKLENGEFQVFFENKEVNFIYLRNSLVSFNMPQSAVFFTVF

>Tb5.NT.95_4 [484 - 606]

MGDRRWRIQTQLIGINVENCRIRQGNFSIERRVGSDAFAYR

>Tb5.NT.95_5 [590 - 718]

MHSHIGRLEEINESSIPEVNHWKTIAHLQTFSNDSVGFTEGSM

>Tb5.NT.95_6 [687 - 794]

MIQLASQKEVCKWEEGCTTRCFLPTVFEREKIHSPG

>Tb5.NT.95_7 [836 - 916]

MVLHFMPRKRKYFSSRKVGFGHGNNET

>Tb5.NT.95_8 [909 - 992]

MKLECRESIGIHTHEYKNPKPLSAWVTE

>Tb5.NT.95_9 [1028 - 1192]

MKTINKESYSKSLQPTKTLVNDTIIIRKLLSHDPVTVAWASGTNHIVKCIWTQPT

>Tb5.NT.95_10 [1171 - 1266]

MHLDAANMRTCGSDTEKVLRFLEKAQVRVRIG

>Tb5.NT.95_11 [1295 - 1408]

MQLAKNNTTQHTGTLLLMVALGREWFVATGIRKDSMKL

>Tb5.NT.95_12 [1623 - 1745]

MGYFYKDIHDFRVWWQPHGGEGKNLRYFLLSCYCGRDSDGI

>Tb5.NT.95_13 [1774 - 1941]

MTTTSLSELPALVSEKQKSASGMPLCACTSHCHMKKLRCNTMRRKEKSKSHSKGAR

>Tb5.NT.95_14 [1948 - 2097]

MSYCVWNAQLTHGIYNNKSLGHMKSGTQLVVAPKLLPERSPKSYTYNNIS

>Tb5.NT.95_15 [2230 - 2334]

MCGIEPKSACGITSDYTVVTHASQIEVRFPSDRCY

>Tb5.NT.95_16 [2552 - 2662]

MQLRSEIEDRWFQVESRRIPSDATGAETGRYGAPETH

>Tb5.NT.95_17 [2707 - 2805]

MEYEAAEQRHAQLSQEMRGIQWRVQYSVYAVAS

>Tb5.NT.95_18 [2834 - 2965]

MRRGPMLREKTYQEQHRHRASPNSCKIRRDIAWANAVGKEWRIW

>Tb5.NT.95_19 [2616 - 2993]

MQQERRQGGTVPPRPINNFKGMCDTGEKTSDGIRSCGTATCSTLPGNERNTVACSIQRIRCSFLTELLGELTSCVGARCCGKKPTKSSIGTGHRQTVARYEGISLGQTLLEKSGGFGNRSGDRNVH

>Tb5.NT.95_20 [2986 - 3114]

MCIDRRDIGSLREDGGHGPFHKMSKLDIIVLELHTYVLSLCLV

>Tb5.NT.95_21 [3140 - 3259]

MRQQTFPWVKVPLMESTGSRKLLTLVSGKRIRIISLCARC

>Tb5.NT.95_22 [3160 - 3291]

MGKGAPNGIHGIEETTHPGEWEENPYNKLMCQVLKNLAVAVGLE

>Tb5.NT.95_23 [3291 - 3392]

MSDVKRLFSEDILFLVYDQESHLWHWWFPLERRT

>Tb5.NT.95_24 [3512 - 3628]

MGLLRKPSNAVKKMYEWIRDYLGHPLLSRIMLGVCASTC

>Tb5.NT.95_25 [3537 - 3677]

MLSKKCTNGSGTIWGIPYCQGLCSVCVHQRVDGEPPSVYDTEIKRKW

>Tb5.NT.95_26 [3559 - 3678]

MDQGLSGASLTVKDYARCVCINVLMANLPQFMILKLKGNG

>Tb6.NT.1_1 [90 - 215]

MYLFEKKRKRRKAALHLKQDACEIEEQLSFEYHIPKLFRLHY

>Tb6.NT.1_2 [148 - 249]

MHVKSKNNCHLNIISPNCFAYIISSKCLRNLGGE

>Tb6.NT.1_3 [293 - 391]

MRVTLVGFLCIGKWSGFKVFYMMFSWRLKYFRK

>Tb6.NT.1_4 [489 - 569]

MIYATAYFLFIYFLRVYISVVWWRGNF

>Tb6.NT.1_5 [407 - 580]

MIQESFRLLISSALPRRTIFGLHCKRWYDICNRLFLIYLFFASLYKCGLVAWKFLACG

>Tb6.NT.2_1 [328 - 402]

MLMRDHYAEGRTTDDCNLKPVICGL

>Tb6.NT.2_2 [243 - 461]

MKLQVEGVDSNHSSPNLCNVNIFVERYSYVNERPLCRGKNNRRLQPKTRNLRTVINFNSFILLCKDGISRLHL

>Tb6.NT.2_3 [439 - 543]

MASADYTCKLFEVKTTIHERECASGDLNRMFMRNL

>Tb6.NT.2_4 [567 - 659]

MLRDINSRCFLFFILFFFSFLFCLFVKQSEV

>Tb6.NT.2_5 [820 - 918]

MFLSLFHLFAHLILCARCCIYLLLHARKKNIYI

>Tb6.NT.2_6 [1014 - 1193]

MTHHYRVVCYELINNYETGFYESYSLHKCNALKECNFIYLFIYLFIYYYYHFYYHYYCQY

>Tb6.NT.2_7 [1293 - 1391]

MVGESLICFDLTDAQSSKGFKKVLGKFNHIFYY

>Tb6.NT.2_8 [1330 - 1404]

MRKVQKVSKRCWGSLTIFFITELFL

>Tb6.NT.3_1 [46 - 144]

MTQIKGSFYRSHHFHVCNSKRVIVTIFFCACEV

>Tb6.NT.3_2 [190 - 435]

MQGALSGEGGADSNIQNNLFSHMAERTETVVKHTLQSEKSISPGADTLQRCEHKTLHLRTGGFSLFICLFVFESIPHITVQN

>Tb6.NT.3_3 [667 - 744]

MNKIPNLVDQLMYLKIESHAVYSLEV

>Tb6.NT.3_4 [744 - 872]

MKIDSITLRCDSNEACVFQHALKGVGNVSEYLRKRQTIFFNGH

>Tb6.NT.4_1 [75 - 233]

MKEGRCKFKKLSFEVPNMSTSVRPSLTETTNLHRVVSLSGSFRTGGKRCECTK

>Tb6.NT.4_2 [657 - 746]

MITIIIIIIIAVPTHKAIRTSWHWFQEPTE

>Tb6.NT.4_3 [1100 - 1198]

MNYTQPCDGKKKLHSSQKKSKMLYHTHTASKPQ

>Tb6.NT.4_4 [1117 - 1263]

MRWQKEITQLPKKEQNVIPHSHGKQASIVDLEGNFQRNSKAIPTESHDA

>Tb6.NT.4_5 [1259 - 1345]

MHNYQLEKGYYQCEKSHREEPLEERGRSS

>Tb6.NT.4_6 [1122 - 1598]

MAKRNYTAPKKRAKCYTTLTRQASLNSGFGGKLPKKFKSDTHRVARCIIISWKKDITSVRKVIGRNLWRKEEEVHSHYHKAENDTILKRPYVMIEHPIKRRALLTRAAAVVAMEHDIDNILLIELGKHIYTMELPRSTVRYLRCVTVSVSKSVLLMFLM

>Tb6.NT.4_7 [1550 - 1633]

MCDSFRQQVGSFNVFDVRYNWKECAASV

>Tb6.NT.4_8 [1618 - 1734]

MCGLSVREAGTYNGEYLPKLSRAQHSLTLTRENKGDITG

>Tb6.NT.4_9 [1734 - 1829]

MRKKKDFTSPIGNECGNDFPSVDLTEDDASSE

>Tb6.NT.4_10 [1813 - 2025]

MMQAASKQFDEVWSIMNSTDDVERGKQREKVSKKESELRIVEKYAQIMKQSRIISTVSERHTTGWVVRHYL

>Tb6.NT.4_11 [2004 - 2081]

MGCAPLFVMEETRNWKKVLIYCMTKG

>Tb6.NT.4_12 [2395 - 2499]

MDMQHMYQCGPRKVVKCRGNKIVTSMKKCGAYYHW

>Tb6.NT.4_13 [2524 - 2640]

MRFHWSFSLTIKSRQYGWDNESDTAIPSIPCYVKKSTIE

>Tb6.NT.4_14 [2644 - 2769]

METVVLRMMYVAAGVRKNNSFLLTTTATCFFNENLHYEWFWT

>Tb6.NT.4_15 [2738 - 2857]

MKTCIMNGFGLRSAEYYFVRLSLSKPTCTPRLFTQLSARE

>Tb6.NT.4_16 [2757 - 2915]

MVLDLGVQSIILYGYLCLNPPVLPVYLRSCLQESEIFRKPIQHNPLRPAQETS

>Tb6.NT.5_1 [209 - 319]

MISERMSCDNHVVAAADGEPTAENQMKASILYKMEDR

>Tb6.NT.5_2 [379 - 510]

MSKCSHTNTHGACDNYIKAGSIFRNEMSVAKKTTTHMTYIYVFV

>Tb6.NT.6_1 [100 - 234]

MFGNRKAIHTIREYPYTNTFRCSTRGFKLYLFLLVVNPRNCVQQM

>Tb6.NT.6_2 [264 - 386]

MHMICMIMRYLTHLRLEKMGDTATCVEQNSRCQHYGQLMKL

>Tb6.NT.6_3 [435 - 554]

MPTLMLHIVCMPVLHCKCAMCPSVLVPLLKGTDVKHVNNH

>Tb6.NT.7_1 [36 - 134]

MDTLNCWHLLVRCSIYSHTFVTSPPSRLIHFAQ

>Tb6.NT.7_2 [263 - 352]

MSPSDISLITVDWRNQYFSGLVPSHLAPSV

>Tb6.NT.8_1 [140 - 325]

MSAYISLRVCDNCSCVQYHRLAACPVTPFRNDPSGVLIVFQTRRYITHTFTSHDLPMLIHSL

>Tb6.NT.8_2 [808 - 918]

MLSLTSYRFLSSLFSGVVSDDLYHYVFSDRAVPRFDF

>Tb6.NT.8_3 [866 - 943]

MICITMFSRIEQFHASIFDGPCCIDT

>Tb6.NT.8_4 [921 - 1013]

MAHAASIHESIYLTFLFLPVGTARTVLLIPY

>Tb6.NT.9_1 [365 - 448]

MFTNSTACMSLHTTTDFIFGDVGGLMLH

>Tb6.NT.9_2 [289 - 474]

MLLGCRLCFPQVSCKMFCVSLGSLTHVYEFNSVYVAAHDDRFYIWRCGRVDVALGIRYWPIH

>Tb6.NT.9_3 [480 - 563]

MPRVTLYSIVITLLPHKNGTRHSTPVAI

>Tb6.NT.9_4 [570 - 677]

MLCVMDSGHVMMSFPSRDRKNEKKTVLGDNVVFICC

>Tb6.NT.9_5 [716 - 790]

MRMCVVAVFFFFACVPQACANRRHR

>Tb6.NT.9_6 [688 - 804]

MRNMQRTTTYAYVRCCCVFFFCLCATGMCEQTTQVISIQ

>Tb6.NT.9_7 [861 - 1010]

MNIRSDHRMRLVLLRFRNDGSVVAGEEKRKEIACSENKCYGCALWVPTPW

>Tb6.NT.9_8 [916 - 1212]

MDQLLQVKRNVRRLRVAKISVTVALSGCQHRGSQRGYNGRIPSCRTVSYPALQKWVWETATVTYTVLPHWSGCFMKLVDRRMSCGDAVCQYRYSAALHC

>Tb6.NT.9_9 [1172 - 1330]

MPYASTDILLLYTANVKWRPKNGTRLHLTQTAAALLDPRNRVRSNTFRTCRSL

>Tb6.NT.9_10 [1222 - 1356]

MASKEWNQAPSHTNSCGTVRPTESGTIQHISYLQIAVGCLRHRQL

>Tb6.NT.9_11 [1335 - 1433]

MPPPQTVMTHLPCKRLIIPWLCRYDMASCALCF

>Tb6.NT.9_12 [1485 - 1565]

MKYVLATESLKPHHHGRYKGNNRCLNA

>Tb6.NT.9_13 [1565 - 1657]

MNLVNTPPGHLRLSFCPQLKRKPHQSRIHIS

>Tb6.NT.9_14 [1561 - 1671]

MHEFSEYTTRALTTIVLPSTQTKATSKPDPYFVMKCQ

>Tb6.NT.10_1 [110 - 259]

MDKGLDINKSFCIKEGTNPIFNWKWFVAQKLHVACRRYVEIGKWGVSSIL

>Tb6.NT.10_2 [222 - 347]

MLKLENGEFQVFFENKEVNFIYLRNSLVSFNMPQSAVFFTVF

>Tb6.NT.10_3 [441 - 674]

MGDRRWRIQTQLIGINVENCRIRQGNIQLREELDHVHSYIDRLEEINESSVPEMNHWKTIAHLQTFSNDSVGFTEGSM

>Tb6.NT.10_4 [643 - 762]

MIQLASQKEVCKWEEGYTTRCFLPTVFERKKKSILLVSRV

>Tb6.NT.11_1 [203 - 406]

MVRFRARSMRVALWWCVFCTVQFSLFMSCEGAFAHEKTGARNAGNEFEHCEFMLVSIVIHARVFVHLG

>Tb6.NT.11_2 [468 - 560]

MSLVITCPRSLFAPQGPILHPSNLKVECSVE

>Tb6.NT.11_3 [446 - 694]

MNYKMKKHVSGDYMSEKPIRASRTHTASQQSKGRMLRRIAVRQPGERQNNLVVENITLPLCWDSCETVVGTSPHFPVTREVPP

>Tb6.NT.12_1 [100 - 234]

MFGNRKAIHTIREYPYTNTFRCSTRGFKLYLFLLVVNPRNCVQQM

>Tb6.NT.12_2 [264 - 386]

MHMICMIMRYLTHLRLEKMGDTATCVEQNSRCQHYGQLMKL

>Tb6.NT.12_3 [435 - 554]

MPTLMLHIVCMPVLYCKCAMCPSVLVPLLKGTDVKHVNNH

>Tb6.NT.13_1 [36 - 134]

MDTLNCWHLLVRCSIYSHTFVTSPPSRLIHFAQ

>Tb6.NT.13_2 [263 - 352]

MSPSDISLITVSWRNQYFSGLVPSHLAPSV

>Tb6.NT.14_1 [42 - 134]

MSMHELSYVLKPALPRSITKFPHRHSYCSLY

>Tb6.NT.14_2 [334 - 462]

MRSYGELWREGASTSLQCVTSTEHLAYLLHASLYIMCVCVHGL

>Tb6.NT.14_3 [422 - 643]

MLPFTSCVFACMGSDCYLRISAALTWGFSPHAGVYALAAALEYQKYCYVLKILFGKGTNGGKHCRPSGGKDKLT

>Tb6.NT.14_4 [597 - 728]

MEGNTVDLQGERINSPESQRNSCGCRHPTFFPQGQLLSLSGLFY

>Tb6.NT.14_5 [847 - 1071]

MRARLIFWFGNEIGGTNSDEVHWLIAAALLRCVFIISYFFPTLRGEGVVTVAATIGSAKKRAQSFQSGKGPITRN

>Tb6.NT.14_6 [939 - 1082]

MRFYHFLLLSNATWRRCGDGGSNHWKCEKEGSIISKWKGAHYPELKLV

>Tb6.NT.14_7 [1117 - 1272]

MKFVLRSKYETCWVDPRKEIKWINTCSLLHETRNMRKMVRMDVEGVSNRMSE

>Tb6.NT.14_8 [1298 - 1396]

MKEKGGVTMHPKRQMIDVIRGDQVFPHDCGVCK

>Tb6.NT.14_9 [1442 - 1549]

MRFGSSVAHVLTPFFHCMCYATFVPKGKLFLSGKRL

>Tb6.NT.14_10 [1467 - 1688]

MFLRPSSTACAMRHSCPRGNFSCLESAFKKYMRGTASQAATQKAKGTTEIIKSRDALAEVEAGGNKTTNKRLRH

>Tb6.NT.14_11 [1766 - 1960]

MKYIYVFVCLCVCANVPLILFFLSLGNDESGCGELPCEWDKLAATCGVARIFITHQQPARVKVIK

>Tb6.NT.14_12 [2191 - 2358]

MPSAGLSVDATSPQLTTIFPSSNVLFTRRVPLAETSKCVFHSFHDVLGRGVLPTHT

>Tb6.NT.14_13 [2258 - 2473]

MFCSRGAFHLPKHRNVYFILFMTCLAVACYQRIRKAFGSGFPSRVCNFKSTALASTCETQKCGCAHWSSWGS

>Tb6.NT.14_14 [2760 - 2882]

MRDIMFFNCFLVFVFSIEEAFMSFPTLIFLWKTKWQHIFLL

>Tb6.NT.15_1 [88 - 216]

MQRQRISPESRCVIGNNNGKVSWERMKEPPVFGSRDRIGESNS

>Tb6.NT.15_2 [414 - 512]

MEGPYLSVSTISAVVAVVAVVAFCEKKIHKGIL

>Tb6.NT.15_3 [550 - 630]

MPSRNSYTSWRYVQPESCNSCHNINNN

>Tb6.NT.15_4 [515 - 664]

MCFHLVLTPEYACPRGTPTPHGDTFNLSPATVVIILIIISVQVVYLFFLM

>Tb6.NT.15_5 [671 - 814]

MNVEVQPLNQKMCPGFPFFLFFFLLQSSRVTILGLCVVRLLKEKIGGK

>Tb6.NT.15_6 [814 - 897]

MKDRISIVVCIKLQRFKYITLLFIEFSF

>Tb6.NT.15_7 [1170 - 1268]

MFFVQEHFVSLLTSLLSFTFAKYLLMFLMQHFL

>Tb6.NT.15_8 [1330 - 1422]

MTGQLRHSLSVVIAFSKKKTLNPSTMNATVC

>Tb6.NT.16_1 [215 - 343]

MPRAVSSRRWWGLRRTIASEFLVDEGNALVFVNTEFPAETKTD

>Tb6.NT.16_2 [285 - 581]

MKEMLLSSLILNSLRKPRPIDITTHGGFSLEVKIGNTVAAVGYLQCAMAVFAEVTPFFFFVKRSSAVNNLDEMSGSIAMLKQRCVYGVKCSRPFPSGLG

>Tb6.NT.16_3 [646 - 726]

MCCPCKGCIFVQAQHHQICLYRITLQF

>Tb6.NT.17_1 [14 - 91]

MSTGRIARGSRWNFAGTLTVKLLYTW

>Tb6.NT.17_2 [96 - 179]

MLAWEGITFCLLCCVTIPPSGDGLVPSS

>Tb6.NT.17_3 [220 - 456]

METYVVSALGDLLMEVGATTVFARKFFFLAETRSTHSLQLVFISVRSPLTGKALQRNDQTTHSGLSQSKRYGSELNCLM

>Tb6.NT.17_4 [511 - 600]

MDNKCNTKSMGRCTEYNRGWGISSVPHVNI

>Tb6.NT.17_5 [870 - 1142]

MQLLLQIDMERNIGTTFGVNSWPKRKIRQSISQPTLNESTSRYGSEYTWLRSPPKRQCGGNYDVTVEGWSKWNILREASDLHRLFDLTGKG

>Tb6.NT.17_6 [1212 - 1310]

MPICYSTWQGVSTYPAGYANLLVRVVSVRYFCW

>Tb6.NT.17_7 [1220 - 1366]

MLFHLARSFYIPRWICKLACKGGIRTLLLLVRGDVVPNSGTIVHIAVGC

>Tb6.NT.17_8 [1320 - 1532]

MSYRTRAQLYTSQWDAEDPFQLKRFASEKCLYPKIFKKGSLCIFSGNMFVCRSSVTCNKTGVPTHRNSGIV

>Tb6.NT.17_9 [1755 - 1862]

MNSLRLLISRGGDEAGQNKAKKTEEATDLLFPSLYL

>Tb6.NT.17_10 [2315 - 2452]

MRLCEELSRAAGVISGTHHGRLRVLFCPVLGGAGGSTPCGKKHKKE

>Tb6.NT.17_11 [2370 - 2471]

MEGCEFCSVPFWEGRVAQPLAGRSIKKNDPHYSQ

>Tb6.NT.17_12 [2471 - 2575]

MTAMVLFTDSSYDHSVAQIFLSFSFSSFFLPPPPL

>Tb6.NT.17_13 [2505 - 2633]

MTILSLKSSFPFPFPPFFSLPRHCKFSYACGRRERKREGLWMK

>Tb6.NT.17_14 [2710 - 2949]

MLYVTFSTNSCKLYLRGCHEAKVNCSLGNSPHDNITVHELVATPLGDNGELDINLKHHWLSGVCTVTKITVCHFFFVQTI

>Tb6.NT.18_1 [42 - 134]

MSMHELSYVLKPALPRSITKFPHRRSYCSLY

>Tb6.NT.18_2 [334 - 462]

MRSYGELWREGAATSLQCVTSTEHLAYLLHASLYIMCVCVHGL

>Tb6.NT.18_3 [422 - 643]

MLPFTSCVFACMGSDCYLRISAALTWGFSPHAGVYALAAALEYQKYCYVLKILFGKGTNGGKHCRPSGGKDKLT

>Tb6.NT.18_4 [597 - 728]

MEGNTVDPQGERINSPESQRNSCGCRHPTFFPQGQLLSLSGLFY

>Tb6.NT.18_5 [847 - 1071]

MRARLIFWFGNEIGGTNSDEVHWLIAAALLRCVFIISYFFPTLRGEGVVTVAATIGSAKKRVQSFQSGKGPITRN

>Tb6.NT.18_6 [939 - 1082]

MRFYYFLLLSNATWRRCGDGGSNHWKCEKEGSIISKWKGAHYPELKLV

>Tb6.NT.18_7 [1117 - 1272]

MKFVLRSKYETCWVDPRKEIKWINTCSLLHETRNMRKMVRMDVEGVSNRMSE

>Tb6.NT.18_8 [1298 - 1396]

MKEKGGVTMHPKRQMIDVIRGDQVFPHDCGVCK

>Tb6.NT.18_9 [1441 - 1548]

MRFGSSVAHVLTPFFHCMCYATFVPKGKLFLSGKRL

>Tb6.NT.18_10 [1377 - 1559]

MIAAFASKVGYKGEKKEKAELDEIRFFRRACSYALLPLHVLRDIRAQGETFPVWKAPLRSI

>Tb6.NT.18_11 [1466 - 1675]

MFLRPSSTACATRHSCPRGNFSCLESAFKKYMRGTASQAATQKAKGTTEIIKSRDALAEVDAGGNKTTNK

>Tb6.NT.18_12 [1765 - 1854]

MKYIYVFVCVTPLPTSVSVVPFYHLLAIYS

>Tb6.NT.19_1 [334 - 417]

MFTNSTACMSLHTTTDFIFGDVGGLMLH

>Tb6.NT.19_2 [258 - 443]

MLLGWRLCFPQVSCKMFCVSLGSLTHVYEFNSVYVAAHDDRFYIWRCGRVDVALGIRYWPIH

>Tb6.NT.19_3 [449 - 532]

MPRVTVYSIVITLLPHKNGTRHSTPVAI

>Tb6.NT.19_4 [539 - 646]

MLCVMDSGHVMMSFPSRDRKNEKKTVLGDNVVFICC

>Tb6.NT.19_5 [685 - 777]

MRMCVVAVFFFFFCLCVTGMCEQTTQLISIQ

>Tb6.NT.19_6 [657 - 821]

MRNMQRTTTYAYVRCCCVFFFFLLVCHRHVRTDDTADQHPVRYVMHVKAALSLFR

>Tb6.NT.19_7 [834 - 983]

MNIRCDHRMRLVLLRFRNDGSVVAGEERRKEIACSENKCYGCALWVPTPW

>Tb6.NT.19_8 [889 - 1032]

MDQLLQVKRDVRRLRVAKISVTVALSGCQHRGSQRGYNGRIPSSQTVS

>Tb6.NT.19_9 [1145 - 1303]

MPYASTDILLLYTANVKWHPKNGTRLHLTQTAAALLDPRNRVRSNTFRTCRSL

>Tb6.NT.19_10 [1195 - 1329]

MAPKEWNQAPSHTNSCGTVRPTESGTIQHISYLQIAVGCLRHRQL

>Tb6.NT.19_11 [1308 - 1406]

MPPPQTVMTHLPCKRLIIPWLCRHDMTSCALCF

>Tb6.NT.19_12 [1458 - 1538]

MKYVLATESLKPHHHGRYKGDNRYLNA

>Tb6.NT.19_13 [1538 - 1630]

MNVVNTPPGHLRLSFCPQLKRKPHRRQIHIS

>Tb6.NT.19_14 [1534 - 1644]

MHECSEYTTRALTTIVLPSTQTKATSKTDPYFVMKCQ

>Tb6.NT.19_15 [1638 - 1790]

MSVARKTTTHMKYVYVFVCVTPLATSVSVVPFYHLLAIYSDIINESPLATL

>Tb6.NT.20_1 [539 - 724]

MSAYISLRVCDNCSCVQYHRLAACPVTPFRNDPSGVLIVFQTWRYIMHTFTSHDLPMLIHSL

>Tb6.NT.20_2 [664 - 762]

MALHHAYVYITRPSYAHSLSLTMEIKGVSGITS

>Tb6.NT.20_3 [958 - 1041]

MCLVDMMVFPFPPLIFHLDCSKVLLFFF

>Tb6.NT.20_4 [1198 - 1308]

MLSLTSYRFLSSLFSGVVSDDLYHYVFSDRAVPRFDF

>Tb6.NT.20_5 [1256 - 1333]

MICITMFSRIEQFHASIFDSPCCIDT

>Tb6.NT.21_1 [36 - 134]

MDTLNCWYLLVRCSIYSHTFVTSPPSRLIHFAQ

>Tb6.NT.21_2 [263 - 352]

MSPSDISLITVSWRNQYFSGLVPSHLAPSV

>Tb6.NT.22_1 [84 - 218]

MFGNRKAIHTIREYPYTNTFRCSTRGFKLYLFLLVVNPRNCVQQM

>Tb6.NT.22_2 [248 - 370]

MHMICMIMRYLTHLRLEKMGDTATCVEQNSRCQHYGQLMKL

>Tb6.NT.22_3 [419 - 538]

MPTLMLHIVCMPVLHCKCAMCPSVLVPLLKGTDVKHVNNH

>Tb6.NT.23_1 [135 - 221]

MNCTSVEKAGKAWYMYKYCPILHVITNDK

>Tb6.NT.23_2 [214 - 324]

MISERMSCDNHVVAAADGEPTAENQMKASVLYKMEDR

>Tb6.NT.23_3 [384 - 515]

MSKCSHTNTHGASDNYINAGSIFRNEMSVAKKTTTHMKYIYVFV

>Tb6.NT.24_1 [41 - 133]

MSMHGLSYALKPALPRSITKFLHHHSYCSLY

>Tb6.NT.24_2 [333 - 410]

MRSYDELWREGASTSLQCVTSTEHLA

>Tb6.NT.24_3 [421 - 642]

MLPFTSCVFACMGSDCYLRISAALTWGFSPHAGVYALAAALEYQKYCYVLKILFGKGTNGGKDCRPSGGKDKLT

>Tb6.NT.24_4 [596 - 727]

MEGKTVDLQGERINSPESQRNSCGCRHPTFFPQGQLLSLSGLFY

>Tb6.NT.24_5 [846 - 1070]

MRARLIFWFGNEIGGTNSDEAHWLIAAALLRCVFIIPYFFPTLRGEGVVTVAATVGSAKKRAQSFQSGKGPITRN

>Tb6.NT.24_6 [938 - 1117]

MRFYYSLLLSNATWRRCGDGGSNCWKCEKEGSIISKWKGAHYPELKTHLIANGVWVATRK

>Tb6.NT.24_7 [1117 - 1272]

MKFVLRSKYETCWVDPRKEIKWINTCSLLHETRNMRKMVRMDVEGVSNRMSE

>Tb6.NT.24_8 [1298 - 1396]

MKEKGGVTMHPKRQMIDVIRGDQVFPHDCGVCK

>Tb6.NT.24_9 [1442 - 1549]

MRFGSSVAHVLTPFFHCMCYATFVPEGKLFLSAKRL

>Tb6.NT.24_10 [1467 - 1676]

MFLRPSSTACAMRHSCPRGNFSCLQSAFKKYMRGTTSQAATQKAKDTTEIIKSRDALAEVNAGGNKTTNK

>Tb6.NT.25_1 [136 - 291]

MKFVLRSKYETCWVDPRKEIKWINTCSLLHETRNMRKMVRMDVEGVSNRMSE

>Tb6.NT.25_2 [317 - 415]

MKEKGGVTMHPKRQMIDVIRGDQVFPHDCGVCK

>Tb6.NT.25_3 [461 - 568]

MRFGSSVAHVLTPFFHCMCYATFVPEGKLFPSAKRL

>Tb6.NT.25_4 [486 - 695]

MFLRPSSTACAMRHSCPRGNFSRLQSAFKKYMRGTTSQAATQKAKDTTEIIKSRDALAEVDAGGSKTTNK

>Tb6.NT.25_5 [806 - 916]

MISERMSCDNHVVAAADGEPTAENQMKASILYKMEDR

>Tb6.NT.25_6 [976 - 1077]

MSKCSHTNTHGACDVKLTVWLCLVEAYKQFFLFF

>Tb6.NT.26_1 [42 - 134]

MSMHELSYVLKPALPRSITKFLHSHSYCSLY

>Tb6.NT.26_2 [334 - 411]

MRSYDELWREGASTSLQCVTSTEHLA

>Tb6.NT.26_3 [422 - 514]

MLPFTLYFFMCFFSMLLVFTATLTFQLCFPL

>Tb6.NT.27_1 [145 - 270]

MFLPLCFLRREMVTWFLCTEHVLFHIIIFHEDFKYCHILCPW

>Tb6.NT.27_2 [206 - 334]

MYFFILSYSTKISSIVTYFARGRCYPGHASRGNNDSFSEVCCR

>Tb6.NT.27_3 [334 - 447]

MMLSAVLHNRLSCFICCGNLVNIQLDVNLHILRFGHKP

>Tb6.NT.27_4 [791 - 901]

MYKTRLPQGRKVYTAGDGVGMCICACTEALSILSAIK

>Tb6.NT.28_1 [109 - 357]

MDRSHCCRHYVVPCGLCHHRRGGGGEQPVRTRHTQDSDGSSVVVEDRKASLLNSFSLLRVTKHVKLLYLVLLSGQCLTLPKSH

>Tb6.NT.28_2 [333 - 488]

MPYAPEVALMSPTKTKYKWGPLLSPPSAAVLSYIGAAYMSHSASFWSPRGNK

>Tb6.NT.28_3 [661 - 735]

METEGLLQLVSIGFVCGSACRNAMC

>Tb6.NT.28_4 [817 - 975]

MQRQPVEPKLMELSRRRAIGRGRGGKAIRGDNGFLRSDPHGRINGGEESILAI

>Tb6.NT.28_5 [985 - 1125]

MRSLYRCAAHVPPLRKTVPRVDGGGGLVVGGFCGVRSYPSVGGIVGR

>Tb6.NT.29_1 [103 - 237]

MPALSNALRERGCALRLTTCFPTALLLASRLPRCVGPQTQSNLEV

>Tb6.NT.29_2 [96 - 353]

MPYACSVKRPSGKRLCSPPDYLFPHCVTVGLPFASVCGSPDTVKPGGMTAQRYNAYQGVSHRWRVALWRVLRCGVGSTAASVIEQG

>Tb6.NT.29_3 [675 - 791]

MFVPSDLRRQVCEVQKWHDDKCERTAASRRCGTPCFHRQ

>Tb6.NT.29_4 [727 - 954]

MTTNVRGPLLHVGVARLVFIGSEEARAFWHTWPALKFDASRGGERVCNQQKLPSSGKSYQLTKRGHKRERLRMSRE

>Tb6.NT.29_5 [1227 - 1310]

MLAWQHNLWITVRRQQTVPVFELAFPWS

>Tb6.NT.30_1 [42 - 134]

MSMHELSYVLKPALPRSITKFLHSHSYCSLY

>Tb6.NT.30_2 [195 - 335]

MPERDVCATFAVVLSCTVRVLLSECCCYGMPSCAFAFFCPRLASLLD

>Tb6.NT.30_3 [208 - 399]

MFVQLLPLFYLVQFVCCCLNVAATVCLVVRLPFFVRVLPLYWIESSKVGPLVSLAVFLKILQWT

>Tb6.NT.30_4 [393 - 662]

MDLECVEKLQMSDLVAISLCIASAAAKTLLSNGGGFVPTAASAWGCCVSNFVCLRVGDNKDVVKNVFAWWCTFTRVTIGDVGRACCGDYR

>Tb6.NT.30_5 [487 - 753]

MGGGSCQQRLQLGAVVCPTLFVYVLVITRMWLRMSLRGGALSHVLPLGMWEERVVATIDDHIGKVAALIWRGDTAYVLLRAVGGLKQGA

>Tb6.NT.30_6 [803 - 937]

MRLPLPACFCHEHFLVTTSCVLCGSVLPVHFHLWYKACVMTSAVS

>Tb6.NT.30_7 [1132 - 1236]

MQRSAFDVFCLLTAHLDLLLSEASTLINTEWLFEK

>Tb6.NT.32_1 [102 - 206]

MPPTASSRVNHPCHCDPSSVTVLSTSRRCAGDAGY

>Tb6.NT.32_2 [453 - 599]

MCCWGTTHPHNLYTTKNSAKWEWRRGGGAWVKSALRNESNEMNLCFRRD

>Tb6.NT.32_3 [657 - 779]

MNHVLRLSWLLPTSPCVMNARSGIFSYLNECTSKDPPLEAC

>Tb6.NT.32_4 [746 - 829]

MHIKRSALGSMLNAENAMGFRLFFCARK

>Tb6.NT.32_5 [783 - 893]

MLRMLWVSACFSVLASDAESLAKNQIDTVILYYDCYC

>Tb6.NT.32_6 [920 - 1102]

MPVVLCLHKRAVVGGLSVTSLSSRVQLAPAPIVTTPPCLNPPFMSRELFKNEETNVGRYGL

>Tb6.NT.32_7 [1195 - 1365]

MLLNNLVSFGGERESPSPGNCTSAFPVPGVRILCNDLSSTMCLCQQRSHPYYVKDIT

>Tb6.NT.35_1 [546 - 629]

MLYLGAVIHLEGMCVWYVHSRMCCAPVQ

>Tb6.NT.36_1 [41 - 154]

MEASLGTSQRNRGLFSVAFQVSVRRSFEKWWGRVLRTL

>Tb6.NT.36_2 [127 - 234]

MVGSCFTHTLAMKNAGSSLCSFSIRERNQPLLNKYH

>Tb6.NT.36_3 [284 - 361]

MHDNIRHIKGDYNWGGTYKTRKFRAF

>Tb6.NT.36_4 [438 - 563]

MLVGSACTATKLGSLSEEETKLIIVLVFQTRRTRQLFSDKAK

>Tb6.NT.36_5 [455 - 646]

MHSYKVRLTLGRGNQADYCACVSDTEDKATIQRQGEINISRMRFFLHITGALILSFRVVDRSLW

>Tb6.NT.36_6 [689 - 841]

MFAEASSKVDDRHTAENIVAHREVKRLFVVVNLIFIRLIILRSRETIYLSK

>Tb6.NT.36_7 [972 - 1061]

MVAIRFFFWGGMWSQRVDGLPRFYHERSKR

>Tb6.NT.36_8 [1064 - 1186]

MVDNLWRVGLDNLRPCCTVQKTPGRFPLQFETMSNVFIFLI

>Tb6.NT.38_1 [263 - 364]

MVIATRTHTHTHIYIYIYIYICMCICMNTRDKCI

>Tb6.NT.38_2 [325 - 417]

MHVHLYEHSGQMHLSYVMIQLICAFVSKSHR

>Tb6.NT.38_3 [515 - 736]

MFPHHHRYEITPQGALPLAVEEKGTKVVFCCLTTVFFLLYVTDVRELGSDATTMLVAVPGNPVAFVQLDHYYYH

>Tb6.NT.39_1 [574 - 672]

MEGVPLHVLRDSTESTWRNDGSHSVFAQGLFGV

>Tb6.NT.39_2 [593 - 673]

MYSGIAPRVHGGMMVHTPSLPRVCLEF

>Tb6.NT.47_1 [263 - 373]

MFVKREKLFKRCVLQLIPHPPSFPPFIFLRSTEESQI

>Tb6.NT.47_2 [373 - 453]

MILHSRAKVEFPPSIPSFPYDLFFSCC

>Tb6.NT.48_1 [85 - 240]

MLNSLRTGEYDSINRYYSKLLHETGLMLRSIGQYLQRYGSVFNTQSAHSEAK

>Tb6.NT.48_2 [240 - 449]

MSCAHMTQFPHKEFGIFCLVAWSVESAPPLRQHREYGRMNIPCFCFCSSADKMYACVADFWFPWDVSLLS

>Tb6.NT.48_3 [433 - 522]

MFPSFRSSGVYCGFHVHGTSNCAFATFWSE

>Tb6.NT.51_1 [219 - 428]

MLLSQLLGFRYSARRIGLDAVAGVGVRRRGRGSSQHAWYFDLLRVPAETLHWVVALRVCLAGPSLFMVCR

>Tb6.NT.51_2 [325 - 489]

MLGILTFCVCPLKLFIGWWPCGFAWRVPRYLWCVGDVLSGVRRRSLARQRLEMLV

>Tb6.NT.51_3 [515 - 625]

MRPKGGGLCVCVCGRPGFLWCLHPRGTVPGGGGSGSL

>Tb6.NT.51_4 [782 - 928]

MFPTLHLTSNSAPLVLLPLPEEVSAVVFLAFCYVVMAIGCGRLLQSKRM

>Tb6.NT.51_5 [691 - 951]

MVWEGGCFSLRYGNPSFGAVGVLCVLLESSNVSHPPPDFKQCAVSATSTSRRGVSGGLPSVLLRGDGYRLRSSSPVETDVTVVQAWC

>Tb6.NT.51_6 [1022 - 1096]

MTVLSVALRAGRIKLLGRQYYVFWV

>Tb6.NT.52_1 [60 - 224]

MKDIVRGGRSCVARLSILFSRPFSLPRLLFQLVLPVVARLLAVERQDYFLLCVRL

>Tb6.NT.52_2 [242 - 316]

MAVICLNQTLAEGTDGKACRTYFIL

>Tb6.NT.52_3 [503 - 634]

MSTGCCTAYGTGDGGLVPYVGTSNQDAVVVILRIYGRRVRFYCI

>Tb6.NT.52_4 [791 - 874]

MEVPLLFLLAGLYELLYSGVVPTHSGIG

>Tb6.NT.53_1 [828 - 929]

MQHVPILFGLLCGAAYVSWLHLFTAWVSDVLAVL

>Tb6.NT.53_2 [716 - 946]

MLRQWVHWCHRLHYPLRRIVLYSVVGGKKVSGGGEGVDAARPHFIWPTLWCSVCFLVAFIHRMGFRRSSGFVVPVIH

>Tb6.NT.54_1 [91 - 165]

MHVLVGEQSGTLVCLLECGSIIYWV

>Tb6.NT.54_2 [95 - 199]

MYWLVNNRAHWCVCLSVVQLFTGSNFVYLTQHCNL

>Tb6.NT.54_3 [221 - 343]

MSNIGTTASSTPLPLAAVITLPFGIYSFVGQLRSTIVYCSS

>Tb6.NT.54_4 [343 - 441]

MKSSMNCNGPSGGGNAKFTLFSYYKECGASAML

>Tb6.NT.54_5 [365 - 460]

MGPVVEEMQSLRCSATTKNVELRRCFSKRSLS

>Tb6.NT.54_6 [420 - 551]

MWSFGDALVSGLYLNSFLTGRSAFFFFFERRGGGEATCPKHRVV

>Tb6.NT.54_7 [912 - 1007]

MSFFKKYVSVTTFSVGKPSSSPHATDVALPVS

>Tb6.NT.54_8 [1063 - 1146]

MRVSFGGGEEEEEEGHLLELVFLYLSLL

>Tb6.NT.54_9 [1340 - 1423]

MAFRNTFTTPGKFSTVSKSIVLLLIWRV

>Tb6.NT.55_1 [158 - 289]

MSSDPLPLQVFFFDVGEWEGREVLTLLLLMPRFHVIADQVVALS

>Tb6.NT.55_2 [331 - 471]

MPKWSGLSGCVAKFFLRYLHVSGSDSLFWLLQNLCDYIGWPRSSVCL

>Tb6.NT.55_3 [389 - 568]

MSRVPILFSGCCKIYVITSVGRGALFACSFEGPLFWIEGRGYFVEPSGISKSVLLWCAVY

>Tb6.NT.56_1 [40 - 138]

MLLMLHSRLLLYIFLTPSLTTSSMRGRRGCGIS

>Tb6.NT.56_2 [213 - 356]

MCTFEMKKKREAVRPSNAQAVNTSFGSHFPPSLVLSYFSALLVKRYPI

>Tb6.NT.57_1 [24 - 122]

MITIIMLRLLTCKPAYIQKIQNKKHIHIKLNYL

>Tb6.NT.57_2 [195 - 404]

MYKLSLGCLRLHPLTARVWIRFLCLVDLISTLLVFAPVSLCACLASTTTTKYIHIFRNIPASSRAREYYL

>Tb6.NT.57_3 [404 - 481]

MIEHLYLHLQMHVHLHILSVNWVLLR

>Tb6.NT.57_4 [768 - 863]

MWCLRSLKLKKKERKEGRKEGRKEEEKKKKEE

>Tb6.NT.57_5 [907 - 981]

MTSKCGARTFISEGTYWIFVPHELR

>Tb6.NT.57_6 [1005 - 1079]

MLFSSWSLPVANSKRDSSVVAFGHR

>Tb6.NT.57_7 [1137 - 1220]

MRINNVFRKLKKKSGGVKWVTGSQKQRV

>Tb6.NT.57_8 [1082 - 1297]

MQLSCNFMPSHYPFKKKMNENKQCFPEAEEKEWRGKVGNWKSKATCLVSFFFVVVVVDYFISCKTVAVIFVV

>Tb6.NT.57_9 [1335 - 1466]

MVWETAPLPVGDNYSFNFPFFFLFFLGGGEGDEERLGLPSVCVH

>Tb6.NT.57_10 [1511 - 1621]

MRLICKERVRERGDCFFFVNLHNGGHDLVSFFFFVLV

>Tb6.NT.57_11 [1630 - 1716]

MVRRHFKYIYIYICILLVLNALTNFSLVF

>Tb6.NT.57_12 [1898 - 1975]

MSVEFEEVQKDNPVSQVLYDRSRFML

>Tb6.NT.58_1 [120 - 416]

MEFVCKKGLTPFSFDFRPALNPRTACHADDMLTTDVENPNSVYFDRERKMDLGSLRQLMVSFVLPRNSDFLKAFLSGGRSTACGMSCVRSRRMSTLLSV

>Tb6.NT.58_2 [490 - 597]

MQQQLTYMQETKAQQAAEIGGLRTIDFYDTAKIGHN

>Tb6.NT.58_3 [449 - 706]

MFECSTNQMSIWVGCNNSSHICKKQKHSKQRKLEVYVQSISTIRQKLVITEGKRDKTGECQEQQSKSETGKAKHIDECAWKGCFSS

>Tb6.NT.58_4 [1133 - 1291]

MKARRQQQQQIGVPPRRSQCFTCGQLGHYSNTCGVFKHKLNTRQKRDIRPQKQ

>Tb6.NT.58_5 [1291 - 1383]

MNLGAVNISLPILHHKGKTNTTEKESYSGGR

>Tb6.NT.58_6 [1410 - 1640]

MSYPTRTGIVRPLRAEKSTAAQRMRGAFQGIWEMSNGNQQHATKVFLVNDVTVILASAHDTYIMALASEANKVAKRM

>Tb6.NT.58_7 [1898 - 1972]

MLRWELDLACDTLTLERDRVALKLT

>Tb6.NT.58_8 [2177 - 2296]

MEGNEKLTSITTKGMKEILRSLGMTAHSIKRGKLAHVELA

>Tb6.NT.58_9 [2296 - 2451]

MSGVKTQALDLRLLTQLGKHADPQKLSRSTVRRYLCRWNTTINKSERLTTLM

>Tb6.NT.58_10 [2486 - 2596]

MQYAGILTPSSVSFIDKKKLSRAHHHSTFARNAGSVR

>Tb6.NT.58_11 [2406 - 2627]

MEHHHQQVGAVNHVDVKHLIGRKADMPCNMPESSRRAVLALLTRKNYRGHIIIRRSQGTRAVSDEEERHYFFRK

>Tb6.NT.58_12 [2596 - 2676]

MKKRDITSSGSKCGYIVFGIGTKTNVG

>Tb6.NT.58_13 [2631 - 2711]

MWVHCLWNWYEDECRMRHSNGLTMYGT

>Tb6.NT.58_14 [2714 - 2806]

MIATERNSKERDRESHLRIAEKDADLLPGPR

>Tb6.NT.58_15 [2927 - 3016]

MNKPQKTFERVSAVAQKSGELVELNRFPMG

>Tb6.NT.58_16 [2704 - 3132]

MELDDCHGTKFERKRQGVSFADCGKRRRFIAWSKVKNARNDYEVGVLLGHISLYLSAVYDESAGLFDLKASFFPDEQATKDIRASFRCCTEKWGTGRIESIPNGLINEAMKLYRHHESFGRRLRGSETTICGRQRIEGSHVDR

>Tb6.NT.58_17 [3093 - 3197]

MRPPTNRGFTRRSITSVSVNHTKRSKNGGTTLLKT

>Tb6.NT.58_18 [3197 - 3445]

MKECGATVAEQQKMVTRYEFIGVLFDHDKQTVLLNAKTTRRIRESAPLETTTIKQMERVVFCMIYAAGIGGGSHFSLTIFSLK

>Tb6.NT.58_19 [3544 - 3714]

MSELLKNVAVVPPQVHPSTATLVTDTSVQGWGGMLFRDKGEIIIAGGSWETAPHMIS

>Tb6.NT.58_20 [3941 - 4024]

MGCLEGYLSGDRTWRWGGTCGGERGRRF

>Tb6.NT.58_21 [3946 - 4053]

MSRGIPVRGQDLAMGWNMWRRAREEVLKTLSALSYR

>Tb6.NT.58_22 [3906 - 4070]

MVLRTFRGKSNRWDVSRDTCQGTGLGDGVEHVEESAGGGFKDAFSSFISLVNYRS

>Tb6.NT.60_1 [184 - 297]

MLNGAVRKSSCEFKEGPVVYLTMGLKLKKKEMWKGKNI

>Tb6.NT.60_2 [72 - 302]

MYCESLCISFSMVEAVGGVEPACARSAIPLHPQNRKHNVKWSSSEEFMRVQGRPSCVFDYGFKIKKKRNVEGKKYMI

>Tb6.NT.61_1 [48 - 131]

MGEEENLNLKSNFFFLLCYLMESLLLSL

>Tb6.NT.61_2 [397 - 504]

MKAEVFERTRTMGEHVNGSCRYYHYCVLDNFYSYYL

>Tb6.NT.62_1 [176 - 355]

MKKLRGRHPLRSVIFEENYRIETGVSYTALDESFPVVHGFFFRIEGGKVHFCRRASEFST

>Tb6.NT.62_2 [397 - 528]

MNSNHFPTGGKYQRNLRGRLYVYIKRTGKALRMFRIARQRVKRE

>Tb6.NT.62_3 [267 - 683]

MKAFLLFMAFFFVSKGGRYTFVGVQVSLARSRHTNTEAFSGLVNELKSFSNWREISEKLTWEIICVHQEDWKSVADVQNCEATGEARMKRIRTQSGSTNCPREDRRGVNKANALWKMFFFFSTVFGWERMRFPLCFLPL

>Tb6.NT.62_4 [601 - 756]

MLFGKCFFFSVPSSGGKECDSHFVFFRCDGLQCEYLTDTSSLIMSSYQHHLF

>Tb6.NT.62_5 [1100 - 1186]

MIIIIIINIVCLTLSRKRRRSETNAICLS

>Tb6.NT.65_1 [165 - 260]

MYKYQYEIRVVLSLELWLSPLPSTHTHTHQYS

>Tb6.NT.67_1 [3 - 95]

MRVNIFLRRCDGYGEGVWFGGFVCVQDDRIT

>Tb6.NT.67_2 [147 - 227]

MTYCSYSHLLIVVSRTMTLKEISRTLI

>Tb6.NT.67_3 [297 - 380]

MRGHIAKEVFVMFTIHARAALIATVTSR

>Tb6.NT.67_4 [248 - 505]

MYNDAPAFANHANFLRDARAYCERGIRYVYNTRSRRLNCYSYLTIAAWITHNPYDKGDLSANYEICLNYSLSSPRYGKGTTGHPPL

>Tb6.NT.67_5 [474 - 557]

MEKEQRDIRRCEKDMCVAASLAKLMRML

>Tb6.NT.67_6 [647 - 763]

MKCCEEAFLPLQQYNILLSCQPVDRERQRTILKTSDVHS

>Tb6.NT.68_1 [10 - 87]

MKLMILSLTRFSRHQLSSPSIQHHSW

>Tb6.NT.68_2 [174 - 293]

MVVGGGIRHAIPCLIMLVYKLNTEEAFTPTIMFVCLFRLL

>Tb6.NT.68_3 [419 - 514]

MRYSNEVETPFIMIANINIKQQQIINLFTSSV

>Tb6.NT.69_1 [63 - 173]

MEATGGRILVVTACHYYYYYYCYYYSVLLLLFLLSLL

>Tb6.NT.69_2 [261 - 533]

MGSAFTSCHIRITEYIYVLVRCAACGLLHQVALTCVDVIERFFLFFFCVCMFVFMHVYVVFRFPPLPRCQRFIRQPSLFVLYCTRPCGVVI

>Tb6.NT.69_3 [310 - 534]

MYWFVVLRVVCCIRSLLLVLMSSNVSFCFFFVCVCLSSCMCMSCFGFRLFHVVSGLFDNLLSLFYIVPGPVGLLF

>Tb6.NT.70_1 [110 - 208]

MTFQRTVNVVRGVWPRYVRFRRAVGVGWQLFCG

>Tb6.NT.70_2 [132 - 239]

MLYVEFGPDMCGSEERLEWVGSYFVANACAYNEPFV

>Tb6.NT.70_3 [100 - 324]

MGINDFPANSKCCTWSLAPICAVQKSGWSGLAAILWLMHVHIMNHLFKMVVSWEFCSAVCMSIEGKCGCDHNCYG

>Tb6.NT.70_4 [297 - 413]

MWMRSQLLRLTGANSLVKDSVVENKRLFHTSTPCVKIST

>Tb6.NT.71_1 [310 - 429]

MAMFFGTVVTAAVDCVEVAYKTMRNLTHPHIFIFIFMLLI

>Tb6.NT.71_2 [449 - 595]

MMQFHTLRINTEASRAFFFFCTMWLPHNLLTPCQNLYWWIVRESALFLM

>Tb6.NT.71_3 [621 - 707]

MSFFLLLVICFCPMRVRVCGRQEKVSNIS

>Tb6.NT.72_1 [218 - 331]

MKCRVDELSNNSNKISTTFSLCFRVWLEKWCINMCYEV

>Tb6.NT.72_2 [234 - 356]

MNYRIIVTKYLRLSPSAFVCGWRNGALTCVMRYSTGRKRIK

>Tb6.NT.72_3 [378 - 524]

MMPIGREKVIQYRRGDAAKYMYLSLCVLRIDMRNIRVWKPETDGDVRGD

>Tb6.NT.72_4 [612 - 689]

MILKFTLLWSIRWEVTCSFIYPFFFR

>Tb6.NT.72_5 [659 - 889]

MFVYLSFFLSLRAHDNVRNYQHVSAPLSFVMLLLLPYFISPSEQVAHAFAFAFVGLFFICSQFILPTFQVFAYGGSP

>Tb6.NT.72_6 [876 - 965]

MAGAHDYGSFVSSIFPHFCMLFQRQHSDSI

>Tb6.NT.72_7 [835 - 999]

MFSVYLTHFPGICVWREPMTMGHLFLPSFHISACCFNANILILFERDRRGEKKGE

>Tb6.NT.73_1 [319 - 423]

MTPVSFVCGSPVCCFVFPVAGSAWADLRLMISSPP

>Tb6.NT.73_2 [339 - 506]

MWLPCLLLRIPGSGQCLGRPSIDDFFASVNVTVSRELRFSHEPNSVRRYFPRDSIS

>Tb6.NT.73_3 [460 - 606]

MNQTAYDGISLGILYPEVLKMRWWWLLLSATAEGSCLPCSTAALLSGLH

>Tb6.NT.73_4 [671 - 757]

MSRLDLTSEGPPLELLFSFPVSVYLCGFS

>Tb6.NT.73_5 [770 - 1015]

MVGLRLRGRSGITVCVTEWQCLLCFLKIYIYIYICICICLPFCVLLVLTQCYSILTVAVVIGFIYLITYTSFRSLLHEGYYI

>Tb6.NT.73_6 [823 - 1290]

MAMFTMFFKDIYIYIYMHMHMFAVLCTTGPDSMLFNINCCCRYWFYLFDHLHLLSFPPARGLLYISVSVGAVVGDKLLYLATGCLLSPHHNFIILYVNVSVLVVTVVGFAYLWLGLLLRINSAPRLSGNIWIINTYLGCLEASFCTLAWSVWPFVK

>Tb6.NT.73_7 [1236 - 1394]

MFRGVFLYVSVVGLAFCKIKISYFCSSLFRIGWVYFRFFVEMVMTLFFFWTAL

>Tb6.NT.73_8 [1403 - 1498]

MFGRIRSCIFFVVYLCVFVVFLAFERLLYRVL

>Tb6.NT.74_1 [392 - 481]

MASINSDEKTRRRTERIRDLLFICVVSLVM

>Tb6.NT.74_2 [655 - 801]

MSSWRTVTEWSVGCCCLAWSLVDSSAWCVATPRHNFCVLTFGAILVTLC

>Tb6.NT.74_3 [693 - 953]

MLLPRMVSRRLQRLVCGDAAAQLLCANFWSYSCNFVLSDPRGHTSCFRSSRREIGAPSPLGPTRERSAVGRVCGSAAGDAGGGALGG

>Tb6.NT.74_4 [928 - 1011]

MQAAALSAVDPVRGEMHFGHQSLSSAPP

>Tb6.NT.74_5 [1125 - 1268]

MCSWKGIGCAPQGGSALLPFRCSLMLAACPAWLLAAGGRAGQRGLVTP

>Tb6.NT.74_6 [1034 - 1303]

MFTYVSLILRAFVWMVLLIACVRSPRATKGDVQLEGHWLCATGWVGAFAFPLQSDASGMPRLAFGCRRARGTAGPSDSVRQPSTAGPALD

>Tb6.NT.74_7 [1581 - 1661]

MFRMFVSVCVCVCVCVCVWKGDVTKHC

>Tb6.NT.74_8 [1565 - 1708]

MSFAVNVSNVCECVCVCVCVCVCVEGRCDQTLLIVLLLSLRFIFQWYA

>Tb6.NT.74_9 [1699 - 1803]

MVCLITAHYQYHLKNRFIYIYMNLLIYSCVRSCMR

>Tb6.NT.74_10 [1704 - 1892]

MLNYSTLSISFKKSLHIYLYELVDIFVRSFVYAMRLKKKKCTAGGGGNRPRYPFRVTFRSFSH

>Tb6.NT.74_11 [1998 - 2261]

MCDEISLVYHRCFICCAGTWWVKLIDWHLEFRTPEVYLFWDYSLTVTVDSYIVHCCVVPPCQGSSPWMVAFFCVYIVCVHLLIFCWMI

>Tb6.NT.74_12 [2177 - 2347]

MSGFFSMDGSVFLCLYCLRSPVDILLDDLTACLRGKTLACFLHLAIPFSDLIDANYV

>Tb6.NT.74_13 [2347 - 2442]

MMTKDGEEKKNFWFVIVPALLPLWSNVLLLFI

>Tb6.NT.75_1 [195 - 275]

MVVTVHLLVLLFEAGFDWGVDHVCLRV

>Tb6.NT.75_2 [311 - 391]

MIVASCMVWGGMLLVSKDLGLLRCLVL

>Tb6.NT.76_1 [14 - 154]

MKFPLSYLELTGGIPSVVFSVEGGITLPHKYTSSGISYCLVGRRRCC

>Tb6.NT.76_2 [169 - 438]

MGRRLMLLLSCKRGGWIMPLHHPAASMFSAPLSYRRLSAWRVGPSTEIFSACNYTSFYTCGGVFHVAMPELFRIQLRRSPFASPGLSYTR

>Tb6.NT.77_1 [40 - 255]

MIRRTVVTSVRYTRKFAAAATRPSANIAQKVSKVNGATGADGDSIVDKLFVLGCVSAVSAWAVFGPAVQSHH

>Tb6.NT.77_2 [324 - 440]

MIYDLYVCLFVAVIVLSQLLLRKSSLYTCQMRACDMWNG

>Tb6.NT.77_3 [340 - 537]

MSAFLWLLSFYHNFFLGNLLFIHARCVHVTCGTGRGEMGVWIIGFFPFLQLSRLVGIPSTKFQTVS

>Tb6.NT.78_1 [264 - 353]

MLGGRNYSPYLHSSPILSNFTVVETMLPTW

>Tb6.NT.78_2 [553 - 690]

MDGKSCYKIMASYRNQPYHLIVWVTDSDAVCAGGRGIFSCKLLFRY

>Tb6.NT.78_3 [794 - 1015]

MKLHFYGYSMTILVISAQVCQKEHKSIFFRVSQWNTGVFSQSGRTNLAKVCSRKEEWVFALYRNYICVKKGNTQ

>Tb6.NT.78_4 [892 - 1047]

MEYWCLFPVRKNKPCESVLEKRRVGFCTLQELHLCKEGEHTMTYLYQNTRMT

>Tb6.NT.78_5 [1201 - 1392]

MLLGISVITRAPLEKAHKGQLKDVQKRNRISMRGKWKLEHLTYHLECYLRIGFSRISGINDIGI

>Tb6.NT.78_6 [1614 - 1730]

MLCCELCVFLLRNLLNMPRVATRRGRIEIKKLQVTQLSQ

>Tb6.NT.78_7 [1973 - 2143]

MELIHFVCKIFEQQHLEITPKITYSSSVKCANTKWKGQSFPSNERLLASSDLFTIYI

>Tb6.NT.78_8 [2168 - 2242]

MVRGGHVRKCVEWRTKWCRPISAND

>Tb6.NT.78_9 [2401 - 2481]

MHYYRAKLFLLVLGMVGEMCNKTESVI

>Tb6.NT.78_10 [2552 - 2629]

METGVSTAWDLIYQLRKAEMNDLFSY

>Tb6.NT.78_11 [2675 - 2920]

MQLLGHTCETVLFFFFFFKGETETFTAEDVRLYSLFVTCYLTVPSVVLSSGGHYAKKLQFFEMMLCIWMGNVKQMLNKDEYN

>Tb6.NT.78_12 [3227 - 3331]

MLRVQLLEVVSTNRLHLLTLSFFRYTFIFYFFINA

>Tb6.NT.78_13 [3331 - 3414]

MKYIYIYVYISCSYFSILILIMFPYSVV

>Tb6.NT.78_14 [3327 - 3440]

MHEIYIYICLYFLFLLFYLNIDYVSLLCCVVLTFLNMQ

>Tb6.NT.79_1 [253 - 378]

MCFHDVTINGFIQMHIGSTYHVFDSNNVKMNLFMDMPTLIAI

>Tb6.NT.79_2 [404 - 532]

MNSAAYRNHQPERENFINKKFGNGRCCQVTAQNRCITFCILRK

>Tb6.NT.79_3 [478 - 564]

MLSSYCTKPMYYILYIEEVRMEWLLSFLE

>Tb6.NT.79_4 [543 - 821]

MVTVFSGVKFRLCCAFRIVIFFKNEKVMTVTTSRQEYTSDNLFNWKMESLVLDVLLESYAVCFFIYKSGVAFIVSLQSVKKATQRSVQELYCK

>Tb6.NT.79_5 [912 - 1016]

MHKWEIHPKCDSLDNVRNRVVMHLVWITTSWWKGI

>Tb6.NT.79_6 [821 - 1081]

MMAVESSNNSYVSLSSTEKSRGISSKTNPANAQMGNPSKMRFTGQRKKSSSHAFGLDYYQLVERDIALLRRENLIAHLHNKDAIIVD

>Tb6.NT.79_7 [1241 - 1402]

MTAPSTKRGELSPSRNSSSRNRERFGIPHFSRNVKACKHQGISSQHRSPSRVAG

>Tb6.NT.79_8 [1478 - 1558]

MISHPTRKVAGHVFIPEAQHFVICSSN

>Tb6.NT.79_9 [1459 - 1563]

MGSFLVDDFTPHEESSGTRLHSRGSAFCYLLIKLK

>Tb6.NT.79_10 [1677 - 1796]

MMKCSKLRVKPRSVGRLGTITEIDCFQSRCIYFGDSSWDG

>Tb6.NT.79_11 [1895 - 2002]

MASLEHVFRYLIAVCNEAAAFIFDSKAPFLSFRWNL

>Tb6.NT.79_12 [2075 - 2149]

MGYEYGSGYPTHDYTQAPERDTEVV

>Tb6.NT.79_13 [1941 - 2207]

MKLQHSSLTQRLRFFLSGGTCSVYLRMSPVAKRRMENLWNQSIFQWVMNMVQDILRTIILRPRKETQKWFKENVSHRIAKNMCELITFA

>Tb6.NT.79_14 [2352 - 2432]

MPPFGGANKRQNSGRPLRWKKQKRENS

>Tb6.NT.79_15 [2405 - 2548]

MEKTKTRKQLKNIISRMMYTADVRGNSLFQQHYLFLKMVRRRPLKLNT

>Tb6.NT.79_16 [2248 - 2565]

MIREHMVQYGAMVGESKLFAEYNFIWVHFDHERKICHLSVEQTNGKTQEGRFDGKNKNEKTVEEHNIPHDVHGRRTRKFSIPTTLSLFEDGAPTTFKAQYMIIGGN

>Tb6.NT.79_17 [2594 - 2740]

MKRKQQWLNIFLNSEYVNSPSYLPTPSKLFAGTSMKDWEAIPFRVSGKL

>Tb6.NT.79_18 [3062 - 3196]

MLELAMRIVEGRLSTVTRFLSCNFYLKIMYKIYNNYFLSLNTSLV

>Tb6.NT.79_19 [3203 - 3280]

MKTIYLYLLLLSVFCFFYFFVRCASH

>Tb6.NT.79_20 [3429 - 3515]

MIVYSSWEMLFGFSSKQKCLFILSCLICA

>Tb6.NT.79_21 [3482 - 3556]

MFIHTVMPYLCLSCRGSPTHDKICI

>Tb7.NT.1_1 [498 - 698]

MSERTMGIIRMHEQAARGSGVRERVSQPALQEVNITNPLGLVSLRSKVVEITLRNDLPKGQCAVPLV

>Tb7.NT.1_2 [661 - 792]

MIYRRDSVQFPWCDGTQLVNSCGVRKGLRRRRHQCSQVSLCRPH

>Tb7.NT.1_3 [1234 - 1317]

MGVYETPLAPENPELVMGTVAVVSPICG

>Tb7.NT.1_4 [1435 - 1560]

MFASAIGHPPSVLVVMRYNTHFYPRFNAIYGNCTSLLPDMSR

>Tb7.NT.1_5 [1343 - 1810]

MRKIRAVISVTSDGAAFRHIQSNGSSKVKLSCLPLLLVIHLPYLLSCDTTPISIHVSTQSMATVLRCYPICLDNTVANISNFTVQKILSDTFLSLQDKIHARLCLCRPTCFVSLIIHFVGVSLLCVILIVSCDNRGLRPLSVFWGGGVSEFSLYVK

>Tb7.NT.1_6 [1923 - 2126]

MITYVTSFFSTCQSINMVIILRVVNDINDGGDKEALLLLWSCFFFVLVTEIISRNVTVSSRASIISVQ

>Tb7.NT.1_7 [2164 - 2244]

MYCLRKWEAGNCPCLQREDQRVLSICV

>Tb7.NT.1_8 [2181 - 2372]

MGSRKLSMLTKRGPKSIVNMCMKGGGYSKWGSVAKFCSLDEGNTCVQIKHLICWRVKPLSDNGS

>Tb7.NT.1_9 [2384 - 2482]

MKDAEKHSINLQVPKERIGKLVEEIKKTAEGRS

>Tb7.NT.1_10 [2605 - 2688]

MVRSSDCVYLGALPLSLGAVVVGCVMRL

>Tb7.NT.2_1 [359 - 454]

MINTTAEAYREGCFFFFVVLTLNSQSDTSTVV

>Tb7.NT.2_2 [653 - 745]

MGSASCSKVVADVLGVSIVTVRGCSLLCPFY

>Tb7.NT.3_1 [63 - 311]

MRVGAACWHRIIFPPSLFYFLWDASAIPLSNASHVWECGGRLLTNALREKLNCAARFFFIQKNKKKVSGGAKSVGSFGRATPR

>Tb7.NT.3_2 [173 - 373]

MRRSVAHKCVEGKIKLCCTFFFHTKKQKEGVRRGKVSWFIWARNSAVKRRRWTHTKICGDNRHYWLD

>Tb7.NT.3_3 [443 - 643]

MRNSGFPHYFPTPHRTENFMGFGLKKAIGMRVVEAPPQGSAASDVCGLFFFYFLMGTEESNAGRRTG

>Tb7.NT.3_4 [717 - 827]

MEPLRALNYFFFLLGTALPTFKPAVGERNKTKLKSGI

>Tb7.NT.3_5 [1032 - 1106]

MKKCGGRVMRRRGPNKFPRRCAFML

>Tb7.NT.3_6 [1124 - 1210]

MLRGVPHLLSPPLFRKKKRKNEDKKYKYE

>Tb7.NT.4_1 [83 - 265]

MDYQFLWDVEFVFPLASLLLSSAITWVGAPIVSRWLPHLPPVEENEPRFTRTPRKTRTKGD

>Tb7.NT.4_2 [216 - 386]

MSPDSLEHRGKPVPRETKKLRCCQLPPPPIFHIHMCTFVGAPLRKGLSADYPHEPQE

>Tb7.NT.4_3 [517 - 624]

MLSSKLRFLISCDVFTLCRRGSECGVQKVLSHCSVC

>Tb7.NT.5_1 [96 - 185]

MLTLCIIKVLCCWLQYCWEQWSGEKMCILQ

>Tb7.NT.5_2 [65 - 238]

MLAHVGSGIVYAYSVYYKGSLLLVAVLLGTMEWGENVHSSVSYDEMSHVLDMSAYPSA

>Tb7.NT.5_3 [259 - 426]

MACTHVRYFSLSLLSFIVIYCPHLYFCRCYRRGYPRVYKSVKRKWIVSANGLKRCG

>Tb7.NT.5_4 [195 - 434]

MRCRMYWTCLLTLQHKLLHSGNGVHSCAVFLAEPLILYRDLLPTPLFLPMLPERVSTCIQECKKKMDRERQWLKEVWVSG

>Tb7.NT.6_1 [108 - 368]

MATTRAPNSGTNPNGVGTCPGGKDTQEATSLPFAAFQLVMAALFILSGFAVKRMRRLSELRAQQRHERLGRVEVGFGMESGSTRLVI

>Tb7.NT.6_2 [586 - 789]

MRGGRPRRSWSSGRILACHAGGPGSIPGLRTFFSHLVVLHLGSSGRGVVSDSGRGSKESGKMEKKRGV

>Tb7.NT.7_1 [129 - 215]

MNFFYKEKSQKESTHLMTSIHCKRHCHAV

>Tb7.NT.7_2 [369 - 449]

MIILFTYFFPLHYCYFSCAISFANCGK

>Tb7.NT.8_1 [98 - 220]

MGAERFTNFTALRAFAEAYAQTILSPNIWCWYVTCEKFNDI

>Tb7.NT.8_2 [153 - 329]

MRKRFCRPIFGAGTLHVKSSMISEVREMTSVFLSDGGGGKEAWFHMFGLILDILQRNKT

>Tb7.NT.8_3 [382 - 510]

MLGSHVMQPQSFSTLQNDLRTTALIKMGYTRKLTVEAGTQAFD

>Tb7.NT.8_4 [527 - 610]

MMKGDNYKMQENIMLAAGAGLMKGDRVT

>Tb7.NT.8_5 [652 - 756]

MTKVQHLAQLKEMLCGNRCVSHFVFYFYLLLFFFF

>Tb7.NT.9_1 [143 - 559]

MVENGDNRSLEGQCPALCTLLHYIQHSTWQTFEHPCHASLQLGKNRNIKPLPPCFPHRPFSSSAQLVVHRRVRSRWLLVVIPTQSVGPRSLSFPVGKYAMKALAMPATSTWCQGPVPRIIGGSQEPAEFLSWGTLLCSG

>Tb7.NT.9_2 [535 - 645]

MGNTAVLRLRHHTAQGSAASCWDTVFHLSVPGKKKMP

>Tb7.NT.9_3 [645 - 944]

MKGSASLTSDGASYQSLKSTGKALSNAAAVQIIAGTEGRYISLPLHQLSPLAVHYGYVFRLPTEETPPQACSNPFCFLRLLVISITSPPHTHAPTAVPAE

>Tb7.NT.9_4 [970 - 1125]

MARADASNREPLTMGTLVAGILNPFFPDAFCVRIRWRLRPPPFRTWATLFYA

>Tb7.NT.9_5 [1074 - 1160]

MASAAAAISHVGNSLLRLSNGGWRTLHGC

>Tb7.NT.9_6 [1132 - 1410]

MVDGGPFMDADFGNAQLFSVTDGSGVSPLFTLLETIFVGNAFLRSVKVVLSVGLQNQFVLSAGASRSVVKILTVIPRRFPFGLFLAPSNPLQM

>Tb7.NT.9_7 [1413 - 1490]

MASLVFSTAIDTYLYLFAELRCAMFW

>Tb7.NT.9_8 [1862 - 2188]

MSRGWGGGGYSPFLNSGVAAALSATPFIFRRLATPILLQMIILHDAALGGTHRSNASVFSYVCVCSISLGCEFVFPFHDAPADPAFGRSPDHDMFAQLGEAKIDSVPSA

>Tb7.NT.9_9 [1992 - 2300]

MMPLLEGRTVPMRRFFHTCVCARSLWGVSLFSRFTMHQPIRHSGGRPITTCSRSWEKPRSIQSLPRKSGFRSRTAFPFGLSINFVVSGEAPLRVPRGEAALSR

>Tb7.NT.9_10 [2311 - 2448]

MTPPGDRAPSAAKTSVLPDVRRVECASHLALISFPCGEPDPCATMD

>Tb7.NT.9_11 [2465 - 2644]

MGWVGGPRIYTYVHMLWPRGCSPYRPCRLRYPWVSSSMCHGSLSPRGIPGVYWGWGVCWC

>Tb7.NT.9_12 [2382 - 2804]

MREPPGFDLLSLRRTGSLRNDGLSGTIAWVGWGVLVYIRMCICCGRGGVLLIVRVGCGIPGLVLLCATVPSPLGAFLASIGDGAFVGVEGLLRGHWIMSERYGCFRFALRGYGWCAYADYLCVCLCFHSALLLRTGSCQQG

>Tb7.NT.9_13 [2686 - 2835]

MVVSGSRSGGTDGARMRITFVCACASTLLCCLELVVVNKGRFCCVCSIVS

>Tb7.NT.11_1 [522 - 596]

MNAGEWEKGSKTMFFVFNTREETQY

>Tb7.NT.11_2 [526 - 621]

MQENGRKGRRQCFLFSTQGRRLSIRWYTFFIR

>Tb7.NT.11_3 [536 - 622]

MGERVEDNVFCFQHKGGDSVLGGILSSYA

>Tb7.NT.12_1 [15 - 95]

MFFPFHHFPFPRQETLKGGTNPHSDIL

>Tb7.NT.13_1 [209 - 316]

MTVFIFISLNMYLYTKYACAKGREVDEQENRGSGRR

>Tb7.NT.13_2 [258 - 347]

MHVRKEGKWTNRRIGEVDEGDGTWKERKKN

>Tb7.NT.13_3 [262 - 495]

MCERKGSGRTGESGKWTKVTAHGKKEKKIKQKFFFSNIQPLCALSRAFGPCCFLFYFTVHNNNNNYYYCYYYYYYYFD

>Tb7.NT.13_4 [326 - 598]

MERKKKKLNKSFFFRIYNHYVHSHVHLAPAAFCFILLFIIIIIIIIIVIIIITIILINVITFILLNFFFFFWGGGVSRLVFFFLARVFYLC

>Tb7.NT.13_5 [498 - 671]

MLLRLFCLIFSFFFGGGGFPDLFFFSSRVCFTFADLRGNDVHLLSITSPTTHTHKRKK

>Tb7.NT.13_6 [679 - 756]

MRGRDAFFSRLMSVFYYFYFPVFFFF

>Tb7.NT.13_7 [826 - 963]

MCERVTTYSTTFISRINMSPHILFSDVSNVNFLFFFFPLSRSFFLL

>Tb7.NT.13_8 [902 - 985]

MFRMLIFFSFFSPSPVHFFFFDGLLRNI

>Tb7.NT.14_1 [228 - 470]

MMSRRICIRCLPPAFDENSLLRICSSFGEVTDFDRRTTTTAKKEESVVEVGFEEADDAIAAAGNMNGMEFSGEFLRVFVKR

>Tb7.NT.14_2 [424 - 537]

MVWNSVENFSASLLSDDALSQLPFPLRVNAYEETYHHY

>Tb7.NT.14_3 [559 - 651]

MATESLLVVITRAGEAVGIVVIQRRVYNHLK

>Tb7.NT.15_1 [45 - 137]

MGGKEDEVESEINFSERQHHTNVRNRNIKKK

>Tb7.NT.15_2 [288 - 452]

MIIYKTEVWFDCEPKWRTRNKVGLHMEVDCLSIAVIPHSCYLYSYALTSFMVVRF

>Tb7.NT.19_1 [88 - 417]

MCRRKCSDRQGGNKCRTNNSNCVFSYNGCEGSQKIFPKFHMRARLRKQTNKIYMKQQQQRKHCLHLLSLIAVIFSTFFFYSCVARLYHYCAACACVSNMSFLTCCSGSCR

>Tb7.NT.19_2 [171 - 434]

MRRQPKDISQISHEGTTSQTNKQNIYETTTTKKTLFASSFPHCGYFLHIFFLLLCCATVPLLRCVCVCIKHVILNVLQRVMQMINDEK

>Tb7.NT.20_1 [181 - 303]

MDVHNCRLISSFFSFSPSLRSLLLRFVSLGYHSRLHRLHLE

>Tb7.NT.21_1 [75 - 152]

MRFFVFYKVLWVSGRHVLYASFDRKD

>Tb7.NT.21_2 [121 - 198]

MYFMPRLTGRINTFGNSVSPRSFDRF

>Tb7.NT.21_3 [234 - 323]

MWAVGRTVILHILVQGVALGRRLLSPPVGL

>Tb7.NT.21_4 [640 - 759]

MDGMYVFVRGNENGGGSTPKYLSKLYSIGSSLFPLFAVFL

>Tb7.NT.21_5 [584 - 763]

MYSHGTVYENSFAISHFPLWTVCTCLCVEMRTGGAVRQSICPSCIVSGLRFFPSLLFSCS

>Tb7.NT.27_1 [174 - 695]

MAFNRSLIVTLFFTLCDLILLCILPIYSHGLSGHILVALHVGTLVVLASMMSLFTLRTSFLVEGRSSKALFPQRVTGPLWLLHVVLTPAPLVYKEFIMPWVYTSVESAPRAWADSAYITLSVLNITTYVAFSVSLLYFIFFIAERRLYKFHCHISRTGTATDNATVALNCNPLE

>Tb7.NT.27_2 [661 - 741]

MRRWRSIAIHWNDSVEKGGEVFWPWGV

>Tb7.NT.27_3 [695 - 847]

MTQWKKGGRSFGHGESETGQGVGFISQHRVTGALVKAQRHQLGATSLLRSL

>Tb7.NT.27_4 [732 - 905]

MGSLKRGRASASFLNTELRGPLSKHNGISWVRPRCYEACNGGKGRPKGKFRDGRYCMI

>Tb7.NT.27_5 [1146 - 1268]

MPFCNVPVPSGGSQCFSPPSSNGSVPSTVLAPHLPSTFDTA

>Tb7.NT.27_6 [994 - 1269]

MRCGNSEENKARLISGALLHPFPSTQKQAALEVRGGGNTSHFATAPFGVQICPSVTFLFLAVVRNVFPHRVLTVQCLQLSWLLTYLPPLTPR

>Tb7.NT.30_1 [139 - 258]

MRIIFLIFLVVVGFTYRLVIIHGRRARSKRQGAAMKGRVV

>Tb7.NT.31_1 [81 - 176]

MKKSIRLLFHVPMGKEGRVVSRTGTNTKERMR

>Tb7.NT.31_2 [241 - 333]

MYSNLRGICSFEVYCKCERLSSMKTRSCYHP

>Tb7.NT.31_3 [390 - 494]

MKITLRSEVEVSVSILQSQVEINFSDLTSTFSHIL

>Tb7.NT.31_4 [513 - 641]

MREGSTLIDGRAIAWVVGPLLSGSLTPNPVHEACGGCGALFLM

>Tb7.NT.32_1 [204 - 296]

MTAATRITLKLGEGMRCDVFFFLNAETSRFV

>Tb7.NT.32_2 [366 - 488]

MSFSSSLVIPSFRKKNCVTYTRVRYMCTFLRVHKKIASLPF

>Tb7.NT.33_1 [103 - 177]

MEVIWCSLLPLSGAAHACGRWLRAD

>Tb7.NT.33_2 [149 - 235]

MLVDGGCGPTETLPSVMVGLRARLTTEQL

>Tb7.NT.33_3 [281 - 397]

MMLSSAPQKEGRTPPPRSALCYLISVRRTPGKRCLYVVI

>Tb7.NT.33_4 [644 - 745]

MSPGFSIVSLFAFMRGVCGVRTGYFAFLTNLCVV

>Tb7.NT.33_5 [555 - 782]

MRSQTARAGIGLSFVRFIECFFFRLYFQLVCLRDLASSVCLRSCVVCVGCGQGILRFSLICVLFSEVHCLRICYLW

>Tb7.NT.33_6 [945 - 1058]

MLCLFFPCGWFNLIWCRPSRYNCMCGVCFFRTTYHYPV

>Tb7.NT.34_1 [122 - 217]

MDKASVNDSLGRYCIYMCLCLSMVFKKYDDFT

>Tb7.NT.34_2 [141 - 233]

MTVWGGIVYICACVCLWYLKSMTILPELRGA

>Tb7.NT.34_3 [267 - 419]

MNVKQQPLYRFVLVVGRATFVVEVNPHVLRVSHHNPVRFVMLGRDRVTFLP

>Tb7.NT.34_4 [580 - 678]

MFAILFFPFTTGIIFPFLLLSLCVLDATCQCMS

>Tb7.NT.34_5 [509 - 691]

MVKCNHEQIFKTKKEGGGRVGTSTCLQFYFSLSLPVSFFLSFYFPSASSMPRASACLNASC

>Tb7.NT.35_1 [83 - 169]

MNSSWVLVVGSSKCVNAANEDIGDLPQRR

>Tb7.NT.35_2 [138 - 263]

MRILEIYLNGGNWCSTNYEALLYWQGNASGGCFFFFWSRAII

>Tb7.NT.35_3 [190 - 408]

MKLCYIGRGMQVAGASFSFGVVPSFEMERKGCASVKFAEGGIVQMLSGPDAFRAGASWRWMILFYFFLKLVYV

>Tb7.NT.35_4 [366 - 494]

MDDFVLFFFEVGLRMSKQHSTLNKWLGRLGPVVLCDDKSVEIF

>Tb7.NT.36_1 [129 - 239]

MKLSPGTNYAGVPSWKMSSHTLPLMRKLHLYVSSEHR

>Tb7.NT.36_2 [273 - 383]

MTTAEAKGGDSRLGAKMIKRIRIHIYISMWLIFFIVM

>Tb7.NT.36_3 [820 - 897]

MQVCVCVRIWRMRGVAMSKAQFRLDA

>Tb7.NT.37_1 [51 - 143]

MMTHGNEAAFVPVKSEKLPMMMERECYRSSM

>Tb7.NT.37_2 [125 - 211]

MLPFIDVMPTERSKYKGEEKGWRSPFFHL

>Tb7.NT.37_3 [187 - 342]

MEEPVFSLVASSWRKSSFSLFFPFSSTFFIIIYIFIYTYVSCPIHVWRDATE

>Tb7.NT.37_4 [342 - 476]

MNREGACTFFRWEKCLIPMSSFCMFCPSEKRGIITDCGIVPLTAA

>Tb7.NT.39_1 [74 - 172]

MEGNIPAHCPSTTKIVWRNVVSNTPGSTKKKLL

>Tb7.NT.39_2 [45 - 215]

MSGGGRAAIEWREIYLHTVLQQPRSCGAMSCRTHREAQKKNSYKLGADAPASPFNDK

>Tb7.NT.39_3 [675 - 749]

MRRPRPVGLAPLPVGHTAWVEAVAT

>Tb7.NT.40_1 [48 - 143]

MACEDTKARATADHSFLSQSISYVLLAKLLTD

>Tb7.NT.40_2 [455 - 658]

MLLYSIFRTRLRAVSTSIHIRSSLQAGLVHIILLNCHFTLQFYLSDRYCFMCVFLQRTLYNLVLIRRN

>Tb7.NT.41_1 [521 - 649]

MQHTTQRGVLGSYTDHLEGILLVLFVALAQIITIAVATVVMCF

>Tb7.NT.41_2 [499 - 666]

MEVFFFLHAAHHTEGCIGFIHRPFGGYTSRSVCCSRANNHNSGCNSRYVFLTPIIL

>Tb7.NT.41_3 [730 - 813]

MDIRDSKSFTYTSEYRHKGTVRSDCGRT

>Tb7.NT.41_4 [842 - 1012]

MACRLCCFESFGVVDIYIYIYIFVGLDISLLMVPLRLIITHFVGVVIMPHFNDAPRV

>Tb7.NT.41_5 [1101 - 1238]

MLWAHTKQNCPPRVCVCVCVDVTTARCSLLPERTSIIVRGGGFTGP

>Tb7.NT.41_6 [1019 - 1267]

MFWRKVPVTMAISTTFYMLLITAAAGSNVVGSHKAKLPSSCVCVCVCGRNYSEMFIVTGKDINYCAWWRVYGTLNGLVCVAAE

>Tb7.NT.41_7 [1310 - 1549]

MSIKLWQGWRACTYGLKQRVGSVTNMGMGEAFLFSPLVLIALLDPHTFTMAFFLENISPSFVDFLVSGVVFPVAVSIANK

>Tb7.NT.41_8 [1709 - 1852]

MCLNTYACVLEESGFSTLLSPERAPIFASSSLFWVELPHPLDARSSVR

>Tb7.NT.44_1 [29 - 103]

MGMCVLYTSQLNPYSGSFLSPHSPR

>Tb7.NT.45_1 [95 - 175]

MLPLNYGLHTIRKDSAEELTEHVTWAY

>Tb7.NT.45_2 [392 - 517]

MKLPHDSEPTLSSFGALWKHHCALKIQKGRNHKLTRSFRRAA

>Tb7.NT.46_1 [20 - 109]

MRITYGIDASDGPKLACNSVCTRAKCGEAM

>Tb7.NT.46_2 [395 - 508]

MTKNISLVFMCFLSYIFFILTGENLSVHRRATQRSAYN

>Tb7.NT.47_1 [190 - 273]

MPSTTKLRGMNVPSNHRIFQVVQGNEQQ

>Tb7.NT.47_2 [221 - 316]

MFQAIIAFSRLYKGMNSSESTGLGISANNNNN

>Tb7.NT.47_3 [409 - 489]

MTSVETVFFFLHFVHVLIIYCSLAVTA

>Tb7.NT.47_4 [384 - 506]

MFVGPDREHDLGRNRLFFLAFRTCSYYLLLPGSDGVIAVYL

>Tb7.NT.48_1 [198 - 383]

MCCAFVIEISIGRSFRNCYTFSTKYFHFPALLAALSTPDTTYEIVTMISQHGGWNTFLFFIS

>Tb7.NT.49_1 [123 - 221]

MRRSPRKFFRRPASFSTNRKSQRDFFVPQSCFQ

>Tb7.NT.49_2 [92 - 379]

MEQPQQRAGSDETLPAEVLPTTGFVFHESEEPKGLLCPPKLLPIKSFALERLEHLEKRMADETKAKRQERQQQKATPQWNTSPTNLRPPLPKPVQP

>Tb7.NT.50_1 [282 - 446]

MRVKSLSLRLHPQCTPLPSVQLLLQKYFLSLSTGGWGLTAATGLRSQGRCLKLSV

>Tb7.NT.50_2 [320 - 478]

MYPFAVCTASFAEVFLVIVHWRLGSDCGNWVAFTGEMLEVVGLAHFSLRSNAT

>Tb7.NT.51_1 [149 - 370]

MNITICPNLFPLLPQVICCSGHLVSGIRFMMNAVVVFFLLLLSKPLLTKKIPKLTRILFCHWRLPGPQSSKGTT

>Tb7.NT.51_2 [243 - 518]

MLLLFFFCYFSRSLCSRRKYPSLLEFCFAIGGYRGHKARKAQREGYRGGGIFMSVMESINEPRPVCIPILLSLRICSRCRLNAPVYLFMLIY

>Tb7.NT.51_3 [487 - 573]

MRQFIYLCLFTRFLLRRWEGATPGMIPFN

>Tb7.NT.52_1 [264 - 356]

MRRTRVALRNRKREAPVEGGTRRRNRGGWMS

>Tb7.NT.52_2 [212 - 460]

MKGRGGAAKPQAGGSRGDAQDAGRAPKPQAGGAGGRRDEKEESGRVDELKSELDRRTNLIFATAKLPKNVLKAVKEVRNEATK

>Tb7.NT.52_3 [482 - 574]

MLSTERALTSERGTGRGGIKRRKENGIVWGK

>Tb7.NT.52_4 [731 - 934]

MINSGLNIRSQTHKPSPKIFGRAVLCVRARVPFIYLFFSHTLTIFPGCSSLSLLSILLLFSFCVSAGV

>Tb7.NT.53_1 [82 - 207]

MSRTEEGGGKKRAQEVIQMRRLFSFLFFFPNFPDHQPQMRAK

>Tb7.NT.54_1 [190 - 264]

MLLSLLLLRTVRAWLAVKHKMEWKG

>Tb7.NT.54_2 [255 - 341]

MEGLIELLRLSENTVFKLCMNSYVEFATR

>Tb7.NT.54_3 [308 - 400]

MYEFLCGVCDPIATKVLTFINRFVMSFDFCL

>Tb7.NT.54_4 [322 - 522]

MWSLRPDSNESFNLHKQICYEFRLLPVNKGFVPHVCRWLNLLMMLKEKKEKVVFLVPFCKLPTLLVI

>Tb7.NT.55_1 [321 - 482]

MVDFFALRWRVESPVSSRILRLPGAVAYLSCPCVLMMSATRCFFLGFLGVVHLP

>Tb7.NT.55_2 [101 - 517]

MLLLQTLMLMGVSILGGALPSSCTQCTSVNESNRWCPVDMTCETQGDCSCGGTACLNFTDCFLQEVSCGDCIKNGGLFCPTMEGGIACVFPNTTAPGSSCISELSMCPDDVGDQVFLSWVLGCGALAVIASLIVGLKVS

>Tb7.NT.55_3 [531 - 671]

MGHEEDGFLVKVTEEADAALSSHTCPKVIVKTGAPQASTRVASLMLY

>Tb7.NT.56_1 [264 - 407]

MRTELPPSVLYCSYFASGIEYCLTVVESMPIFHLFCYKLNCLLPLSFF

>Tb7.NT.56_2 [633 - 776]

MEQINFGVVTLFHARNLINTSRIVQAETLRTQVRLWSIVRRPKRWFEF

>Tb7.NT.56_3 [990 - 1079]

MAFFDSTYNRPRVYEANIVFVIIFLKNNHG

>Tb7.NT.56_4 [1075 - 1173]

MVSSISICNSRNVKTVAPFQCTIVFFSNLFKCH

>Tb7.NT.59_1 [31 - 105]

MGSSECTISVKGQEGRDMLVVAAVS

>Tb7.NT.59_2 [198 - 317]

MPSSLSIFLPAFHRLLQVIFLLLYSLPLLVVVVHHRICAP

>Tb7.NT.59_3 [357 - 449]

MHGTDVQYDMYVVRICRRLRDELFGPLLSFV

>Tb7.NT.59_4 [361 - 519]

MELMYNMICTLCVYAVGCGMNCLGRYLALCSGNISVVVPTGSYVLFTVFSTVT

>Tb7.NT.60_1 [198 - 311]

MCPSFAGATLTCPKVKNVGDTTYLFGEVVDPGNCRSEK

>Tb7.NT.60_2 [247 - 327]

MLATQHTCLGRWLILEIAARRNNSVNI

>Tb7.NT.61_1 [25 - 108]

MYLLELDLKWFCRFSLCDIVGVLSHSMY

>Tb7.NT.63_1 [158 - 244]

MLLYHFFCRCFPCVYYAGSWNMPGPSFLF

>Tb7.NT.63_2 [91 - 474]

MFLHNRGLAVDRAVLRLNPLIVYVIIPFLLPLLSMCILRWKLEYAWAELFVLNALVSAFTGLVMWLHSERVANMFLQYSGHSYGKANEGAAALLGVHRISPVLPACVSLFLMLISFWHVVVMILLAVM

>Tb7.NT.63_3 [493 - 582]

MAHWFPDCLLSYDSFPCRFTIHKHVCIRAK

>Tb7.NT.63_4 [527 - 688]

MTLFLVVSQYTNTFAYVLNSGGCAYLIFCLVPQLLLLTLFCMCVCVCVCIGYGR

>Tb7.NT.63_5 [670 - 816]

MYWLWALALISVPTALDKPICESGGHLMVSFHNLLWPRSTFPFSFFATA

>Tb7.NT.65_1 [263 - 421]

MQFWPDFKFPVCRLFHMRNKRGNEPRWRLARGDAVIIIITDRNCEKWELAAKW

>Tb7.NT.65_2 [418 - 528]

MVTNSLKGLWREITSASAIIEVIFFSTFAFRDILPSS

>Tb7.NT.66_1 [513 - 635]

MMINIMSIAPNRNVHTCEVPFVWIITFKLVRRAPCQQLIIA

>Tb7.NT.66_2 [335 - 844]

MAYSDYNKQEASTETSHNDYYEQCYGDAAFDYHEEVGEAIAEEIRINMGRRNVKAYVVHNDDQYNVYRTQPERSHMRSTICMDHHIQAREESALPAVNHRLATDVLGRGAHRSLICRPLDRHGFQTAVSSPTCLILPNIGACNRSPTYPQNYQHNGRDNFMLPVITMRKR

>Tb7.NT.66_3 [1114 - 1194]

MQRSVSWFVEYMEMCKERNPREIYKLV

>Tb7.NT.66_4 [1517 - 1726]

MLCFPSNFHLALQNQFLLLSCCNQKKEKEEEMLLFCCNYHNYYHYYQYFSLLLLPEVGCGFVVTLLFDLT

>Tb7.NT.66_5 [2413 - 2526]

MFGYFINFIPFSLNYQQGATFRIGFVVCFFFVMWFSFS

>Tb7.NT.66_6 [2691 - 2858]

MCFCMFFVRAFSYGFFSFFFLILIYIFLIPFSIPLIFTSVISAAALFFPMLFPLSM

>Tb7.NT.66_7 [2975 - 3127]

MLRQFNLLYHFFFVLLNFYYEKRKNFPDFVMASPPLSSFNIRPINARGKKV

>Tb7.NT.66_8 [3108 - 3194]

MRGGKKYKQIYNVCLYTKGKENTIPQFST

>Tb7.NT.66_9 [3142 - 3495]

MYACTRKEKKTQYRSFQHNPPLVYIKHKHTRAVSLYFFFFLFIYIIFCIFIVPEPLLISGFNSFVSLFLSVSFYLSFFPLSTPPPTPPVLLHFHLYLSIPKRRRLINFFYTFHVINIF

>Tb7.NT.66_10 [3538 - 3687]

MFSSFFAVSITLHHHLPFSHIFFKLLPSLLPYTPMFWLLFKKKIKVSPKI

>Tb7.NT.67_1 [71 - 151]

MSAVMMKGAEANFRCFVGEPVCSEGSS

>Tb7.NT.67_2 [46 - 183]

MRAWHSLFNVCGDDERCRSQLSLLRRGTRVQRRFLLTVGCSAFDRQ

>Tb7.NT.67_3 [241 - 384]

MLAWESADADVIAHVYICVYVYAHLCTRGRSMWAYPSLPERKPVAPKR

>Tb7.NT.67_4 [263 - 490]

MLMLLHTFIFVFTCMLTCVQEEEACGPIHHYQSGNLLHLKGDNPLKKYCYVGEKQTAKKKRKGKLSKAYPSPRRLR

>Tb7.NT.67_5 [543 - 623]

MVSSLSPNATQREDGTTQRYEAPKPYL

>Tb7.NT.67_6 [565 - 654]

MPLRGKMVRPNATKHQSPICKVAKGDEERG

>Tb7.NT.67_7 [1000 - 1257]

MGVWVCRKGGKISRSNSYIFVVSNSPTQQDEGTAHWGNRGLHFAEALNRHAQITHVCWMFDHAHVVEKVDAHPSAHQARRDQGFHP

>Tb7.NT.67_8 [1863 - 1967]

MVRGPGRKGLPAFGFGGETAVRPLFYIFLCNRISV

>Tb7.NT.67_9 [1970 - 2053]

MRSMKGLSVSLLASYSRSSCLFAEQVGV

>Tb7.NT.67_10 [2222 - 2353]

MIHPWAPSTHHHKKDCEFITPEGLPQYLLDNSAPLRLHTSDVSL

>Tb7.NT.67_11 [2356 - 2433]

MFGDVISHSLQLQDLLRLYAGIAPVY

>Tb7.NT.67_12 [2540 - 2731]

MCPAGSRHVAVILSVKGVPDGITSLNRAVFSCATMAIISVNRSLPEGIVGKRLGACEHSPTTNV

>Tb7.NT.67_13 [2727 - 2819]

MFSFLPPNYVPHCVEDVFAPCGEIGAFSAER

>Tb7.NT.67_14 [2925 - 3014]

MPSASNNIINTVVCHKLRVMLTLTVEAERV

>Tb7.NT.68_1 [12 - 86]

MRSFLPLSDGCFSKAFVDQNVVSKK

>Tb7.NT.68_2 [97 - 171]

MLIWVVCSYVMLLRVRSMLRRPEAY

>Tb7.NT.68_3 [269 - 361]

MMKYLRKTQTLAWGTHGETHYYYFFFDLTQH

>Tb7.NT.69_1 [51 - 152]

MVDSRMFLAPLFLSGVCFTTSPCRNKSGSHALLS

>Tb7.NT.69_2 [98 - 220]

MLYNISLSEQVRQPRTFKLRWHISESPVHSPILIRYSTEGC

>Tb7.NT.69_3 [550 - 645]

MSLWRVTFLLGYVVSFYLFSGFVGDGKVACVR

>Tb7.NT.70_1 [589 - 750]

MNFGRQLVGTESAVKILLCIAILSIDHAHVIGFVCGSRIAPAAEGYSVRLHVTR

>Tb7.NT.70_2 [874 - 1119]

MTTGRPSKSLNRMSGIQYYRFAEDMPILCWKADISQFRWILYDGWGLVSRQSKGCFAWVGRRTERIHIVRCVGARLTEFHSV

>Tb7.NT.70_3 [998 - 1171]

MMGGVSLVDSRRAASRGSADEQRGYTLYDVLVRGSLSFTVSETKVRKMRRPKLPGVSL

>Tb7.NT.70_4 [1248 - 1439]

MLSSLQWGARKEWLRAFFLRLVQAKMCYGTEYRWFSTCLPGINTMAKLLSRGANIIMYVCALWY

>Tb7.NT.70_5 [1420 - 1518]

MCVPSGTNRSGVPKEVRLISLDNLAQACNRARP

>Tb7.NT.70_6 [1751 - 1837]

MPPHTARFRRRGKTESIIWCASYYTGWCR

>Tb7.NT.71_1 [389 - 475]

MPAAKKHNIIIPFLGPSKSRVDVMLFRYH

>Tb7.NT.71_2 [590 - 751]

MNFGRQLVGAESAVKILLCIAILSIDHAHVIGFVCGSRIAPAVEGYSVRLHVTR

>Tb7.NT.71_3 [875 - 1120]

MTTGRPSKSLNRMSGIQYYRFAEDMPILCWKADISQFRWILYDGWGLVSRQSKGCFARVGRRTERIHIVRCVGARLTEFHSV

>Tb7.NT.71_4 [999 - 1172]

MMGGVSLVDSRRAASRGSADEQRGYTLYDVLVRGSLSFTVSETKVRKMRRPKLPGLSL

>Tb7.NT.71_5 [1249 - 1440]

MLSSLQWGARKERLRAFFLRLVQAKMCYGTEYRWFSTCLPGINAMAKLLSRGANIIMYVCALWY

>Tb7.NT.71_6 [1421 - 1519]

MCVPSGTNRSDVPKEVRLISLDNLAQACNRARP

>Tb7.NT.71_7 [1752 - 1838]

MLPHTTRFRRRGKTESIIWCASHYTGWCR

>Tb7.NT.72_1 [389 - 475]

MPAAKKHNIIIPFLGPSKSRVDVMLFRYH

>Tb7.NT.72_2 [590 - 751]

MNFGRQLVGAESAVKILLCIAILSIDHAHVIGFVCGSRIAPAVEGYSVRLHVTR

>Tb7.NT.72_3 [875 - 1120]

MTTGRPSKSLNRMSGIQYYRFAEDMPILCWKADISQFRWILYDGWGLVSRQSKGCFARVGRRTERIHIVRCVGARLTEFHSV

>Tb7.NT.72_4 [999 - 1172]

MMGGVSLVDSRRAASRGSADEQRGYTLYDVLVRGSLSFTVSETKVRKMRRPKLPGLSL

>Tb7.NT.72_5 [1249 - 1440]

MLSSLQWGARKERLRAFFLRLVQAKMCYGTEYRWFSTCLPGINTMAKLLSRGANIIMYVCALWY

>Tb7.NT.72_6 [1421 - 1519]

MCVPSGTNRSDVPKEVRLISLDNLAQACNRARP

>Tb7.NT.72_7 [1752 - 1838]

MLPHTTRFRRRGKTESIIWCASHYTGWCR

>Tb7.NT.73_1 [389 - 475]

MPAAKKHNIIIPFLGPSKSRVDVMLFRYH

>Tb7.NT.73_2 [590 - 751]

MNFGRQLVGAESAVKILLCIAILSIDHAHVIGFVCGSRIAPAVEGYSVRLHVTR

>Tb7.NT.73_3 [875 - 1120]

MTTGRPSKSLNRMSGIQYYRFAEDMPILCWKADISQFRWILYDGWGLVSRQSKGCFAWVGRRTERIHIVRCVGARLTEFHSV

>Tb7.NT.73_4 [999 - 1172]

MMGGVSLVDSRRAASRGSADEQRGYTLYDVLVRGSLSFTVSETKVRKMRRPKLPGLSL

>Tb7.NT.73_5 [1249 - 1440]

MLSSLQWGARKEWLRAFFLRLVQAKMCYGTEYRWFSTCLPGINTMAKLLSRGANIIMYVCALWY

>Tb7.NT.73_6 [1421 - 1519]

MCVPSGTNRSDVPKEVRLISLDNLAQACNRARP

>Tb7.NT.73_7 [1752 - 1838]

MPPHTTRFRRRGKTESIIWCASYYTGWCR

>Tb7.NT.74_1 [297 - 386]

MGGRSKVTTVQFIGLIKHGRNGGVRKTIIR

>Tb7.NT.74_2 [544 - 648]

MVVTIRKKILPSLWTLQKKKSVKCRPHRDRTFLTR

>Tb7.NT.74_3 [861 - 974]

MKMEVNVNKIKLCYNCLFKKKLQRHTYFFASVYENYNN

>Tb7.NT.74_4 [1117 - 1284]

MLNIITCKGASKLSDVWRRVCVVLCFPLDSDYTMPYILLFSKLACISSFGILHTGA

>Tb7.NT.78_1 [158 - 262]

MRQIMHVFLSVTYTYAHCICGSCRGSSFVVAYRSG

>Tb7.NT.78_2 [201 - 317]

MLIAFVVVAGDHPLLLPTGVDNMCTFRRFVCVCLCRGYD

>Tb7.NT.79_1 [33 - 140]

MLQFRSCSDPHIVRAHVCLCTYKLWLGPFIISACHS

>Tb7.NT.79_2 [83 - 202]

MFVYIQVVAWTFYYFCVSFLRGQGFCTSTIGAYGRVAARV

>Tb7.NT.79_3 [219 - 308]

MCAFVDLHRRRQEKKMDGTAVVATASRRSD

>Tb7.NT.79_4 [79 - 333]

MYVCVHTSCGLDLLLFLRVILEGSGLLHEYDWRVRKGSCACVTEQRGCVPLLICIGAVRKKKWTVQQWLLLLPAVATSCPRAGTC

>Tb7.NT.79_5 [446 - 529]

MMSRKRLYYTAGTCCVATARAHNIAVFL

>Tb7.NT.79_6 [570 - 674]

MRTVLAFKRFCFTVECKSALHCYIGVWVADWQLPC

>Tb7.NT.79_7 [532 - 747]

MYFCLALFTMESGCAPFLRSNGFASRSSVSPLYIAISEFGWLIGSCRAENDHSSYKNFQGQSVFVAVATYVC

>Tb7.NT.79_8 [680 - 967]

MIIPHIKISKDRVFSSRLRPTCVKLIWYRCIFTPEMNKQSKEFGSCCYCCYDHGGTRGFLYTAFPCVGSAGPTTFCDGSLPYSMFPAGMLHNGWFK

>Tb7.NT.79_9 [831 - 1211]

MTMEVHAGSSILPFRVLALLDPPPSVMGRFLTVCSRLVCCIMVGSSEEVVGGMLGNLRLSCVRLPNLLRKLIDLLFPTLFWLLLDTVSNPLGSFSGLHLLLAGPREGDVVYVRVVDRRRQRVAVTGV

>Tb7.NT.81_1 [43 - 132]

MFWFVPGLATGRMQKHSPPPPFATHESIIL

>Tb7.NT.81_2 [454 - 591]

MSHKWNTHFGSNHVGFPCCFPAPNSRTERKQYVSAVDTIKCKWVFR

>Tb7.NT.81_3 [434 - 610]

MWLLLFICLTSGIPTSGRIMLASRVAFLRPTRVPKGSNMCQLSTRSSVSGCSVSSCGTK

>Tb7.NT.81_4 [610 - 876]

MKVLTIPADLVNQDFFLCALWVGCGRECLVPCLTKCFSCFSLNSCGRYQYKVQKGRYLHSGFGLVLATPVSVNTATFTRHHMRVPCGSS

>Tb7.NT.81_5 [690 - 896]

MFGPLPDQVFLMFFLKFVWSVPIQSSKRQIFALRFRASTGDAGFSEHRNLYSSPHESSMWLFIISTSCD

>Tb7.NT.81_6 [1105 - 1236]

MASPGHFFFYIPLSHTDGYKLPIKLFKIICVTNSRFSYVSNSGN

>Tb7.NT.81_7 [1257 - 1370]

MYVSWGEIVMVRSALLRCSGKAFSSTTLARVCFAGKGV

>Tb7.NT.81_8 [1349 - 1498]

MLRREGGLALMRLREECRGVAVVTTAKVLPWGGDECFTVDAANNLVVIYN

>Tb7.NT.82_1 [80 - 178]

MCSASFKGSHMQTHSGTRLPSQGLQSGRSQITK

>Tb7.NT.82_2 [64 - 318]

MWRISNVLRQLQRQPHADALGYSAAFAGATERPVTDYQIATAPYSPAATQLARRKGHLERQRRYTEKIRMISSTARLKLNMCDKG

>Tb7.NT.84_1 [71 - 169]

MTRKGTTPRDAVEGYNYFASHASSFAVLGCAPH

>Tb7.NT.84_2 [157 - 279]

MRAALTGLRSEQVSRSSNFFPCLFVCVCVTCGMGPLRIDNS

>Tb7.NT.84_3 [283 - 444]

MSVETCLLTHYESYGSKKTVANRTFGFSAVILHGSDADSDREVRQLSSFTGIRA

>Tb7.NT.84_4 [699 - 824]

MRCDGDISAAVRMLQRWCRHFGCGEWSSLPRRPVFHSLSSLC

>Tb7.NT.84_5 [592 - 972]

MSIQGKPVTLIDKISRCSLLTVSARYLVLSTREIFQCVVMETYQLQSECYNVGAATSVVGSGRRYRDGRYSTPCRHCVSLQREPGVWGNRVDALPRAVSRMDVQLREECSVMVSLYCCCCFVLIGST

>Tb7.NT.85_1 [110 - 190]

MTCSRLCRMPSEKLINAYGQSAPLRTQ

>Tb7.NT.85_2 [103 - 321]

MKDDLFASLSHALGEVNKCLWSVSSPQDTVTSLMFSSVPRHREERSDECTYASTVTVIASLNDRWKALLLIFP

>Tb7.NT.85_3 [346 - 441]

MCFRCMRRKGRKLCIVNEVPYGCIRACTGGVY

>Tb7.NT.85_4 [411 - 521]

MYTCMHRRSVLSMALAMDAVCGERLSCDSSTCGVLLT

>Tb7.NT.85_5 [739 - 813]

MERCKLPWFVLSCRSQVGGRVEGLA

>Tb7.NT.86_1 [287 - 385]

MITKTHILPFQKLRGKEDRRFLYLARGLSVRQG

>Tb7.NT.86_2 [422 - 589]

MLVFLRQYHVLELIDGLHLTRRMMTHHFYMCLTKVAVLNVCHRICVGHPHVQACFF

>Tb7.NT.86_3 [537 - 626]

MFATAYAWDTRTYRHAFFKNYSVTILFQHI

>Tb7.NT.87_1 [37 - 210]

MMILKRMYWVLQSYSLEGWTSIVTHVKQSGSLFVFSCVRAHMSIQRAAALRSCVGVKF

>Tb7.NT.87_2 [90 - 338]

MDLHRNPREAVGESFCFFVCACAYVYSTGSCTSQLRRCEVLKRNSYCRLKLPVPHFLWYLQDGTLLMTKLTCRLTVWVRVHLV

>Tb7.NT.87_3 [317 - 412]

MGPRTLSVISHYFWNWWRWRLDPFSYCFLDAL

>Tb7.NT.88_1 [185 - 271]

MNVNGRWDSRWNARFNTRFSSRELEKYTY

>Tb7.NT.88_2 [202 - 291]

MGQSVERPVQYKIFLSGTREIHLLRRSNRW

>Tb7.NT.88_3 [401 - 550]

MLDTISVVTEFVSHNPSLRAWHYLTHCDPLTWMIGAVLMRVQHLVLFPRL

>Tb7.NT.88_4 [578 - 676]

MVFPPIFGPSSHFCAYTCTSSLLVTAARREPKT

>Tb7.NT.90_1 [217 - 309]

MTVASVGTNFAGAYDLLGSISTTYPDKRVYS

>Tb7.NT.90_2 [309 - 407]

MILLSPPQCNVAAYSVPSVRFVGCLASGGNRCL

>Tb7.NT.90_3 [627 - 728]

MMGRIIRPLTCLSLLPATVVVSTVALTGLKSSLN

>Tb7.NT.90_4 [756 - 830]

MPPNPSLLSLNSAAGTSESDFFKLA

>Tb7.NT.90_5 [870 - 1136]

MIDSYRVSCAGVGHNCVWQKNRLLRIFFTLVSVDVHQKTVSTMSIRRRCCSEAPMCCKKKRGLYTVFFWLVSVFGRLYPFARRGGLLPS

>Tb7.NT.90_6 [970 - 1143]

MCIKRRYLQCLFGEGVALRHRCVVRRNGGCTPSFSGSFQCLVAYTPLLGEGGCCHRDV

>Tb7.NT.90_7 [1143 - 1310]

MKRFNICSYISVSQDHVCWGCRCLCWWTPLDCAMHVGCCGGQFYRTTLQFTVAIAS

>Tb7.NT.90_8 [1139 - 1363]

MYEAVQYLFIYFGVARPRLLGVPMPVLVDPAGLRHARWLLRWAVLSHDAAIYGGNCFINSYSFPSCVSLWSTCFG

>Tb7.NT.90_9 [1383 - 1520]

MEDTIAHEFKHAPPVFLLSGVVFALRIDNGDLLWRLLVPSYGRVRW

>Tb7.NT.90_10 [1481 - 1603]

MEVTSPFVWASSLVVRKCVVWTFFFFENIIFRSRIFPRLSL

>Tb7.NT.90_11 [1790 - 2077]

MCAARLMCSFVLQEKITTPLRFQSIPLFCRKLPRFCHKRLPFALEELVPLPRDISVEQSGVMTSITVGTRILLYTQVLHNIVFLKERCNRLSCAFL

>Tb7.NT.91_1 [107 - 211]

MKFGASLCVSTWLDALYNWNSSAYFLRGFPFWKAS

>Tb7.NT.91_2 [82 - 297]

MITLRYVNDEVWCFSVCINLVRRLVQLELERVFSARLSILESLLILVLERVGCTCNAFTSSGCICLYTLACD

>Tb7.NT.91_3 [305 - 430]

MRVTAAKQLLYMGFGVCVVFFEVNNPQRGIAPILSRYSAWFC

>Tb7.NT.92_1 [247 - 357]

MERVRTTSVTCGKSSLNIGQLLILLLLCMCDRYVTRD

>Tb7.NT.92_2 [344 - 448]

MSPGTKAGTCWRWIPPKLYAVKKVMSCLTVPVSSR

>Tb7.NT.92_3 [544 - 621]

MQSHELYVQRIRSSEYDPGSVFKKDG

>Tb7.NT.92_4 [590 - 754]

MIRDRYLKKMDNTDSFFMTMCYGAAPYCAVMGCLFLLLQQKLWVLFRGWGSNISI

>Tb7.NT.92_5 [654 - 815]

MVLHHIALLWVVCFCCCSRNFGCFLGGGDQTSQFDTFLLQYFHCTKKKKGLQTL

>Tb7.NT.92_6 [879 - 1046]

MKQHGIFVSSFTNTDERHYSTVKIINSQVTTHPVHHTPFFSPSSADVSLSVARKLC

>Tb7.NT.92_7 [889 - 1158]

MEFSFRLLLTQTSVTTALLRLLTLRSRLIRFIIHLFSPPPVLMFLSLLRANFVEVAQLWGRLSTAFFCVWREFFNLMYAVVAYSFFYLDR

>Tb7.NT.93_1 [425 - 511]

MEWKHRQGLMSAMEAIGTEDQRGWRSGVL

>Tb7.NT.93_2 [352 - 534]

MCGRYGGKVSGVLRLLWLRSGAGWNGMETPPGLDVGNGGDRHRRSKGLEVRRALSIVEELK

>Tb7.NT.93_3 [629 - 715]

MACRMVEAGWQRNWREGGGRNVWVGLAHY

>Tb7.NT.93_4 [600 - 731]

MKTEHFPPPAWPVAWWRRDGRGIGGRGGGGMCGLDWLIIESIQP

>Tb7.NT.93_5 [655 - 759]

MAEELEGGGGEECVGWIGSLLKVFSLSDAATKLTI

>Tb7.NT.93_6 [784 - 870]

MNGSGMMTIDDGKWRPKATSTGVREMSEG

>Tb7.NT.93_7 [909 - 1025]

MHTWEIQRKRKKRATVFFLFSYRDSAASMVKVLRVCDIL

>Tb7.NT.93_8 [1320 - 1400]

MEVTLLFSAWEGHLQLVVRERKFCDPN

>Tb7.NT.93_9 [1497 - 1730]

MKIGLAEMPGHDYVTATIHQLESNGMDLTSTCFCFEKRRSTQNFLDELETTVVHIVQEGVLQWRSETSLFSWGQGEFD

>Tb7.NT.93_10 [1633 - 1851]

MSWKRRLSTSSKKVSYSGAVKLHCSRGGKASSTKFKCILRNKIVAACNWSRSHHNAVSTERTIWHAAVGVVLT

>Tb7.NT.93_11 [1919 - 1993]

MKRKKRKLSESNQRKCGEKVSKQHN

>Tb7.NT.93_12 [1963 - 2097]

MWREGLQAAQLMLLNMVVDATTLLLEAWYYPLLEEAPKNWGSIMK

>Tb7.NT.93_13 [2603 - 2710]

MSWEANFKSSRSSESVPLPRHRGYLCAVRRGNYVLY

>Tb7.NT.93_14 [2485 - 2895]

MERDSKSNPIRVVKLRQKQNITPPLSFVARVSSRSQLTSHELGSKFQEFTQFRIRASTSAPWLSLRCAPRKLCSLLRALLQPLLKTQRIEVVTILMCVGVCVCGGGVKLMMRLYSGSSQSICSPLSAVAKISAELPL

>Tb7.NT.94_1 [51 - 146]

MSHGRMNMSFVLARHGSHLNCVCCRMRCCDRI

>Tb7.NT.94_2 [393 - 494]

MWYSISFSLLLTLWVVLKNVREHRRGENPRWLRR

>Tb7.NT.94_3 [621 - 704]

MTDSYSLITLCSLPCSHTNADILFFRNA

>Tb7.NT.94_4 [650 - 814]

MFVAMLTHKCRYFVLPQCVIEHYYLEWCSSWGVRLTTRALRYLRMYGMYVCSQRR

>Tb7.NT.94_5 [859 - 936]

MRSGSSGVHFLFVHALCLYRHRLRRD

>Tb7.NT.95_1 [227 - 469]

MLRQLKINDVRARVFGHNFHGDRRPAFVNVTVLIATVPTRFRSAAFPLALAMLSTFCNPSKKCAQMLRLWDKCTMRSAEVL

>Tb7.NT.95_2 [541 - 789]

MTAQTWRYGGRRLWFEYCSVRLRRHGQLSVPTTSICLTNEVNNTGVFVGLSLTILNSRGCSLYPVNRPLSQVSGLVLSSASSK

>Tb7.NT.95_3 [1129 - 1320]

MEETRIYAIVVSSSSFHWEEGLRRGGAAACVVALLFGCTFTVARSCPSQWCRCPSCAPRGGGDS

>Tb7.NT.95_4 [1331 - 1429]

MLRSLGAHPQLQPKRFQPRLGRCSWCFLFTFSF

>Tb7.NT.95_5 [1432 - 1539]

MSPGPVRECDWRWVASCTEKRYTLSIFSVTGDCPLH

>Tb7.NT.96_1 [98 - 268]

MEIQNFPFSLSKTLPPGAGWTNVPRCRGSINTLISWAQHSVVVSRQSNSPKFMIHKV

>Tb7.NT.96_2 [288 - 374]

MEGVNTLKVPVIVHAVLDQVRHREGVCSR

>Tb7.NT.96_3 [630 - 713]

MCNTTLSWGKTLCVGILIYAYSLCRVSG

>Tb7.NT.96_4 [602 - 847]

MLSGENRVFHVQYNAVLGQDAMCWHLDLRLFSLQSQWMIAHLHGLSSKKEKSFMKTTTPPSTPNAQAARRCSLKKKMWFSCY

>Tb7.NT.97_1 [88 - 465]

MWTWNIFEATTFYLHSPSVMQRSLLLTSRHFDPLPPHTQNLWCHLQYTCTSKSFRVWMSHQSGGINIFCCRCSGSETTYLFGTDCMNNDLRQTLVTFYYFIVRSVVVDVTGSQCFSAAFDITHDIL

>Tb7.NT.97_2 [455 - 529]

MTFYDTVFCLLSTEGRGSGRWLPLL

>Tb7.NT.97_3 [742 - 984]

MLIHDVMTRSKSNVLIMEWSGSCIVDGMTLHIRVMPVRSWRESESFRTIPRRRSKNRFSRGAHTELEESVKNLYGKMLTVY

>Tb7.NT.97_4 [989 - 1075]

MHDGTSCGVYALPDSLRGVGDRGGGGEGT

>Tb7.NT.97_5 [996 - 1118]

MAHLVGFMRCRILCVAWGIGGGGERAHRETMVDNYGEIGCR

>Tb7.NT.97_6 [1474 - 1563]

MTQLPSDICGRCFGYLSFLTCCGAKAYTPW

>Tb7.NT.97_7 [1713 - 1886]

MQRSSECILIWGGTVVFHFRQLWCIHFHQAMQFSVPLLLFLNLRCTEGTWNAVLSLMR

>Tb7.NT.100_1 [57 - 158]

MKGTFCFGVSSHQWIYKLLQPYVRYTVGKMYTKG

>Tb7.NT.100_2 [121 - 369]

MCATLWGKCIRRVDNLLCLCVCALITFVALRWLLLAPECPLLCALWCMRKCNKGLMGKECLTCWNDSSISNRSGITFVTRFPV

>Tb7.NT.101_1 [101 - 211]

MRCGRFRTFCRCQRRGECIVFLHLNLLLIERAFAYPI

>Tb7.NT.101_2 [306 - 386]

MSLTNRNEYLNRKKYLRLKNVCSLGIS

>Tb7.NT.102_1 [179 - 328]

MSASEAVCWCVCVCVCVCVFALNRCIIGDIFSQEEEVSVVVTDALRRLLG

>Tb7.NT.102_2 [250 - 432]

MYHRRHLFTRRGSFSCGHGRITQVVGLKQHKRGNHLGCFGPLAVVVLHFVYSFKRRNGLKV

>Tb7.NT.102_3 [653 - 757]

MLEWALLCPYACFFFLFCQKSLLATRVSALHTLIF

>Tb7.NT.102_4 [531 - 758]

MYESGARMLWVQVVRGCRSLLGDKTCAGIHYIPVAKYPQSQCWSGRCFVHTHAFFFCSVRSPFSRREYQLSTLLFF

>Tb7.NT.103_1 [49 - 231]

MPSISVSSPLLAVVYEQLHPLLCHLLDETAGNWCRKGFGGSSVRRVWRPYTRPTNTIASPP

>Tb7.NT.103_2 [128 - 268]

MKQPETGVAKVLEGQAYDEFGAPTPGLRTQLLPLHEKGGRFGWGRGF

>Tb7.NT.103_3 [272 - 361]

MHISFPLAQWGEKRLYTPAKPQRNCAPHKG

>Tb7.NT.103_4 [231 - 455]

MRREEGLGGGAGFECIFLFHSPSGAKNVSTRPRSHNEIARHTKVESTYRPSVRFFLASEVQRLLPNPNQHADVES

>Tb7.NT.103_5 [439 - 546]

MPMLNPKPRKPSTAHAPPGREGVKRRGLHGTGFRTM

>Tb7.NT.103_6 [482 - 604]

MRLRGGRGSNGVACMEPASELCDWTRHAAGFVGVDETQAVF

>Tb7.NT.103_7 [675 - 761]

MGRNRSLPIRQNLMDSVLNSGFCPFVRYC

>Tb7.NT.103_8 [821 - 988]

MREERKGSSVGFLWATRVSRRVGKSVDMSQRIRPRWCGKQGFSCLAFAAPMARWVG

>Tb7.NT.103_9 [1011 - 1178]

MQPRRMKVPPPNQAGARSLSQEIWELHFFVVRFLVYFFFLGVYSIPPSLQYFFWRV

>Tb7.NT.106_1 [125 - 214]

MSCRRIKRVKGMGRGKWVDGRFRSRKCMAA

>Tb7.NT.106_2 [180 - 290]

MGGSEVGSAWQLRPQKLKDMRKEKGEEAGRTAFSHQH

>Tb7.NT.106_3 [473 - 628]

MNYISMGQRMAQRIRIDQSHRHHAITSNHLVDLRNETHNHALSAQCYTRLYC

>Tb7.NT.107_1 [167 - 241]

MCSTHVCPISKMLASTRRGGIHSRS

>Tb7.NT.107_2 [132 - 317]

MSLAYLKRMVAECVPRMFVLSVRCLPAPVEEVFIPVADCPACMTVPLRRSRATQSESCETSL

>Tb7.NT.107_3 [254 - 505]

MHDCSSTPQPRHTVRELRDILMKNIIYQPLKRGLLIFPSEYLIDTSCSCAHDCTGSCVRRHLSSGDTLQDRREYLVVFLFRGSL

>Tb7.NT.107_4 [397 - 519]

MCTRLHWVMCETPFEQWRHITGPARIPCCLLVPWEFVKLHY

>Tb7.NT.107_5 [632 - 724]

MGGEGMEVVMMDSIGCVSPALYVVACGGYGL

>Tb7.NT.107_6 [696 - 776]

MLWLVAVMACSHATVNECSAPIALRGV

>Tb7.NT.108_1 [84 - 161]

MSCRKREYKLVCITWQICDVVESARA

>Tb7.NT.108_2 [139 - 306]

MWWRAHVRETQTCTCVLSLLFCCCWGGGEVFMRLHIHEEVRKGSNRNWTVRCWLLE

>Tb7.NT.108_3 [180 - 314]

MCAVFVVLLLLGGGGSVHAPPHTRRGSKGQQPQLDGKVLVIGMMS

>Tb7.NT.108_4 [394 - 474]

MQITACEEPMEIILPANITVLEGLRLG

>Tb7.NT.108_5 [565 - 648]

MCISREGERKKGEICKAKIRRRTRSDSL

>Tb7.NT.109_1 [208 - 354]

MLAISAVDEVCMEMVKRETLLSDVVVPCPAVRLLPLWSRGKKRTGSDPS

>Tb7.NT.109_2 [275 - 436]

MSSFRVQLSGYFHCGHGEKSEQAATPLESPSVAPSYCFCNYFERDMRLPASLPM

>Tb7.NT.110_1 [30 - 116]

MCDFVGRKGGDIRGRKCMTCLHGERKLKC

>Tb7.NT.110_2 [304 - 453]

MTRPLSSRGGDKFSKGYSFSVLLRYKLFSPSVGGGGVVPGIRFSVPAKGS

>Tb7.NT.110_3 [1013 - 1132]

MSPLGGGGAEAKHDCDHGRWFAAAHHEVVCLFCLRARERG

>Tb7.NT.110_4 [1050 - 1151]

MIVITAGGLRQHTMKLFAYFACVRASGGEKTRSL

>Tb7.NT.110_5 [1219 - 1317]

MFSFKPFPLVPIRVSCAELMAVSRTVIRHFTLV

>Tb7.NT.110_6 [1426 - 1524]

MCKRCHMFCFSKMRGRDYARAALRSSPSSPIDP

>Tb7.NT.110_7 [1382 - 1546]

MFETFRDANAHTYQQCASVATCFVSLRCGAGTTPVRLSGLPPHLQLILSIFPVPF

>Tb7.NT.111_1 [67 - 237]

MCKHLGEGCESTVATAVKKPCYLRGGGDDFFFLNGLHHCVSPTCFGGSRSELASVIG

>Tb7.NT.111_2 [432 - 527]

MFGHRPTLLGHYYYYYPTDSRPTSQGQLWKQL

>Tb7.NT.111_3 [664 - 738]

MGGSPHGGERGLSVSMRPRIRQWHR

>Tb7.NT.111_4 [902 - 1033]

MRKSGRHDQDYDRNSEREKRSPTRGVHTKTSLSLCVVLARVSSR

>Tb7.NT.111_5 [921 - 1166]

MIKTTTGILKGKNVAPQGEYTRKLRCLCVWYWRGFLADEKERGDGGPSCCTKPKCHGVLPDSIPLAHIWTQVAGRICARLFF

>Tb7.NT.111_6 [1033 - 1266]

MRRSAVTEDPVVALSLSVTGYSRIVSHWLISGHRLQVASAPAYFSEAVGYCLIIVSISCSSGRLLVTKADDLIGEKRK

>Tb7.NT.111_7 [1697 - 1843]

MPQQTSAWKMGIIAAPKGWKRSEEGKRLRVIKKLSHAVVNPWVAPHSRW

>Tb7.NT.111_8 [2021 - 2134]

MMPACPPGNDVNATNDSTHGPNNVLKALHYIIMERLGN

>Tb7.NT.111_9 [2425 - 2742]

MCITKIIPAYIETGTVNTPMETGREMDAQRLVSCVSFRIRNGDNVGEVVAHRVAKRLVKQYASYEGSTCYPGYDVPRLVDVYLFVTVHDWTRGRGFGGWIRRYYNL

>Tb7.NT.111_10 [2615 - 2755]

MKVAPAIQDMMYHVWWMCICSLRFTIGQEEEALVVGSEGIIIYEDGL

>Tb7.NT.111_11 [2929 - 3072]

MTDKAGNARDSSISFPHCLSGDAVKSATTRPLICAFGIHRLYNAIVAR

>Tb7.NT.111_12 [3216 - 3344]

MGRGGEVARHFCNLNGPCTFTVFVFARVHNVTLQVIVWQKERC

>Tb7.NT.111_13 [3097 - 3423]

MRSDVNGKCPYIADETEKVWSARLLSLHPSAPSGTRFTLEWEGAAKWRGIFVILTDLVRLLFSYSREYTTLLYKLLCGRKNDVEAKWCGSCTHETVGTRNLPVLRRSFI

>Tb7.NT.111_14 [3341 - 3430]

MLKQSGADRAHTKRWGHGTCQFYDAASSKR

>Tb7.NT.111_15 [3825 - 3941]

MPHVCRAVVAFTSPFIFMLCFPLAHRCLPHGIFCGFFGR

>Tb7.NT.111_16 [3941 - 4030]

MRQVCAQTNVPYLKSNLLFRQIILAISLPK

>Tb7.NT.111_17 [3913 - 4182]

MAYSVDFLGDETGLCPDKRTVFEVQPSIQTDNTRHQLTEVMLTDEFRCRAVFFVQRVEVRFVCQGRRFHTLYISQRWVDGSDYYYYFCFF

>Tb7.NT.111_18 [4043 - 4207]

MSFGVGLCFLFRELKCDLCVRGAVFIPCTYHKGGWMEVIIIIIFVFFDAKEHTLF

>Tb7.NT.111_19 [4185 - 4274]

MQRSTLCSSHVTRRRAEVTFEAYYPVRKAR

>Tb7.NT.111_20 [4213 - 4311]

MSRVGVQRLRLKHITQFVKHAEDIEKHITSYVD

>Tb7.NT.111_21 [4426 - 4503]

MEEGGHACRKCYLVVRVKAVKLVQLS

>Tb7.NT.111_22 [4455 - 4538]

MLLGSSCEGCKARAVELIANGVLMLRQR

>Tb7.NT.111_23 [4593 - 4793]

MVVFHIFYFPFLHTKFTVVGKQLSRASPWLYKCCVHWKWLPKLPHYMKEEACVLSKPRNTCLSTCFA

>Tb7.NT.112_1 [307 - 387]

MEVLGIGHPVSYFLCCSHHNHHTDTFP

>Tb7.NT.112_2 [267 - 488]

MFSLTGAGHYRHCDGGAWNWTSGFLLFMLFTSQPPHRHFPVGMMSFKKSGTDGSRRASTMLIFCLDGIVKADQL

>Tb7.NT.112_3 [651 - 749]

MQLIKFEVTAETAILHCSGLCQRVLAGVMISWW

>Tb7.NT.112_4 [710 - 835]

MPTGACGGYDQLVVSAFLKRIVGYEPSSSAGCVYKEAGFFLH

>Tb7.NT.112_5 [802 - 927]

MCIQGSRIFSALIGAMPINGCLMDALNETFSVELEVLTMLAE

>Tb7.NT.112_6 [881 - 1039]

MKRSLWNLKCSQCWQSSFTGRTKMYLTERRYRSALAEYSEYIDSDDPWWDKLN

>Tb7.NT.112_7 [1053 - 1148]

MLIMSTRMKRRVLVISLYKSCLAANLPRYRRR

>Tb7.NT.112_8 [1151 - 1279]

MEKLKCLTPNPEMSHIRGEHGARLVRHGGAVLTISVSFTEHMR

>Tb7.NT.112_9 [1458 - 1586]

MPMGRRQIYFPARVLLERPKELKVFTGRCWLRGRSPVTDVLGS

>Tb7.NT.112_10 [1426 - 1617]

MNGTGIETTWLCRWGAVRSISLRGFCWRGPKNLKSSLGGAGCEAAVQLLTCWGLDESHLEPINR

>Tb7.NT.112_11 [1767 - 1913]

MRLFFCFRFFSRKFHIIENLWCDIGTDKSLGVQSNGDTYELQSNGTSDE

>Tb7.NT.112_12 [2042 - 2131]

MLNTTPTYAPLPFVNPADCIRGERQAKGAL

>Tb7.NT.113_1 [11 - 85]

MFLLCGQCNMICASPSIGARAFGAH

>Tb7.NT.113_2 [22 - 228]

MRTMQYDLCQPLDWCESIWGTLKHKNVTSELCASDLGCWCVVHPFLQCLVQSAALFIFMGSVKKRDVPS

>Tb7.NT.113_3 [162 - 302]

MSRAIGCALYLYGLCQKERRSVVNALCLDVQSYVPRCARMVLPQSPL

>Tb7.NT.113_4 [232 - 306]

MPYAWMYSHTSRDAHAWYFHSLHCK

>Tb7.NT.113_5 [239 - 373]

MPGCTVIRPEMRTHGTSTVSIVNETQRLGLIAEGGTDYFFSVIVH

>Tb7.NT.113_6 [506 - 604]

MYPIKQAASVRISEDNSPIGISGGGWELVAKMV

>Tb7.NT.113_7 [722 - 1003]

MGVEHRFFGGRVSPARFHTRSVGAEEFGICEAIIFAPHGIRMLVLPHEYSLLLRTFAMLILCCRMRGGVKLWLSFLFHLFRCPDSNMYLWCVCA

>Tb7.NT.113_8 [1011 - 1103]

MAERNCKPTQTHIYIYIYIRNKALSLSLSSI

>Tb7.NT.113_9 [997 - 1290]

MRVMQWLRETANLHKRIYIYIYIYVIKRFHCPSPRSDGTWISQRHKTEKEATCVSDLSYLLQIVFVIGEWSSQERVRWRVRSTPAIFFLFGRLNFDET

>Tb7.NT.113_10 [1444 - 1608]

MPSSSEEKQCVPETNNASRRCSRGGNWVLCHLPPPMGCALHIRSNYWDAVVTNGT

>Tb7.NT.113_11 [1283 - 1609]

MRPDGSYHKVLPRFPIGGIFHEHRPRFPSGCCHLDERGRWDCGLLIDEEVGFIQCHRPRKKSNVCLKLTTPLGGVREGVTGCCVTSRHRWGVPCISDPTIGMLLSRTAH

>Tb7.NT.114_1 [51 - 299]

MLQNQQSENEWWELNSLPQDNTGNQPPVDGHHCPMTRPREGEEDIRAQPPSHRQKTEGNEQPRWKMSYGEGDASLTKWLRRFA

>Tb7.NT.114_2 [445 - 600]

MWGFPLPRPFGSRRTDRYDSFTKRRQWTIILEQLGAACRWGGGEDGVLGAEE

>Tb7.NT.114_3 [642 - 728]

MRSHIAVLQAPGHVPLKEGDDEAPLTHRD

>Tb7.NT.114_4 [764 - 850]

MKLDDTLPCGSITGNVEATSRAGSLRMGL

>Tb7.NT.114_5 [787 - 861]

MWQHNWKCGSHITSRFAAHGIMNSP

>Tb7.NT.114_6 [807 - 890]

MWKPHHEPVRCAWDYEFPLRNRVPTSRC

>Tb7.NT.114_7 [1147 - 1263]

MIIMAGLNAIMVWTSIGILTMVSEEGLERRATFDMADNI

>Tb7.NT.114_8 [1291 - 1365]

MLRYRGSGDKWRKKKINTERFIRAV

>Tb7.NT.114_9 [1551 - 1631]

MCLGAGGKERATAMWGLRKQGRDQTSP

>Tb7.NT.114_10 [1529 - 1720]

MCPEITFDVSGGGGEGEGNSDVGIKEAGKGSNFSLGAHEAIPCATSRQTRYRLRRRNDTKSIIL

>Tb7.NT.114_11 [1641 - 1772]

MRQFHVQLRGKLATVFVDAMIRNPSFCEGKESQENLFECLTAEM

>Tb7.NT.114_12 [1871 - 1957]

MGRDMFRCPTSVSLVCFWVQWKFRYRLRF

>Tb7.NT.114_13 [1852 - 2034]

MDEMVKNGARHVSVSYFSIFGVFLGAMEISLSFTFLIRFLHYLGALRRTLSTGSSCDVAAG

>Tb7.NT.114_14 [2021 - 2125]

MWQLVNALLTSACTLKYSALHFFMDISPARCRYIV

>Tb7.NT.114_15 [2215 - 2301]

MSLLCSCFQTLMMIENSFHVTSVGNRVAL

>Tb7.NT.114_16 [2391 - 2588]

MSARPATKNFCERDSDGLHMFVVLFTAVLWQRIEEAELVELRVHSLLLKCMAYDTSLHPSSFSLTH

>Tb7.NT.115_1 [217 - 309]

MTTAIPKSEGSTVFFSGEVDEPRTSVMDARP

>Tb7.NT.115_2 [342 - 530]

MHMYACISVCMFFFPFTHICSAKCARSCRVIGLFLLGVQPHTFVFFAEPCAAACNCIDSTRNF

>Tb7.NT.115_3 [556 - 675]

MLRFLVTRMGYFCGIDCTTLLVTNPARFSCGDNLLAWDPF

>Tb7.NT.115_4 [763 - 852]

MADKDVRCDVRQYVLGDACGLLRPVRAILR

>Tb7.NT.115_5 [689 - 1033]

MDLGNITHIRAVGTLSANIIATEVGWLTKTLDVTYANMYSVMRVDYCGLSVQFCGKVTCDSWTGACLGCGVEVAFKQRIFLIRLWRKLPSLSNGGGGENRDTLRLGLIPIKSRCK

>Tb7.NT.115_6 [1045 - 1206]

MQGELIFCGQVEGDGNKMMGSVRTNEFQGKEGGECVISLVSAGGGLSLSGRESG

>Tb7.NT.115_7 [1461 - 1589]

MGRPCKHTKVKRGVPLDCWGDGCIFALHVLLFTCLMSKPPFQE

>Tb7.NT.115_8 [1802 - 1921]

MVALLEVFFFFHPSSCTFTDTDTQTVNSVTSEAHCTMDLN

>Tb7.NT.115_9 [1934 - 2020]

MILNFAGQRGGCCTVYGLGIGIWLLPIPH

>Tb7.NT.115_10 [1966 - 2064]

MLHCLRLGYRYLVAPHSPLKYDFPPPPPRISDT

>Tb7.NT.116_1 [165 - 377]

MYCSVRCPMPSFKKMCVCVCASRVMEALVCALPTSAPSDSYMRVVRGEGRRFSAVHQHLLGMSNASSRWQN

>Tb7.NT.116_2 [355 - 477]

MHPAAGKTEVVFRISGHTITAHVAFHTSVTCFVFFFRQPVT

>Tb7.NT.116_3 [419 - 607]

MSHSTHQLLVLFFFFVNRSRNDRSCKAVVPSETTPETAMAAGVYARFSLREKRHLCGMVGLRC

>Tb7.NT.116_4 [480 - 827]

MIARVRLWCPAKLLPRLLWRPAFMPALVCVRSAICVEWWVCGAKHIQAQFCVCVSGSLVVRNCFFFSLSFPVTYDPDHFFFFFCMWGGVTFVHKWGSTLSLLRAPVVCAQADVGVG

>Tb8.NT.1_1 [5 - 79]

MQEGRCKFKELVLKCQTCPLQYVHH

>Tb8.NT.1_2 [55 - 162]

MSTSVRPSLTETTNLHRVVSLSGSFRTDGKRCEWTK

>Tb8.NT.1_3 [589 - 672]

MIIIFIAVPTKGCPYIMTLVSGANRIAK

>Tb8.NT.1_4 [676 - 948]

MWLRTKTCEQQGIHHAVFLCIGEKSLPHSSIGCNYTSICCCKFATRKEQRHFSTGELFHHFYWTHRRGCCFWVRLYNTVTGEPIKQTQHML

>Tb8.NT.1_5 [1018 - 1197]

MNCIQPSGGKKKSHSSQIKKEEKCYTTLTRQESLNSGFGGKLSKKFKSDTHRVTRYVIIN

>Tb8.NT.1_6 [1321 - 1440]

MIEHPKKRRALLTRAAAVVAMEHNIDNILLIELGKHIYTI

>Tb8.NT.1_7 [1541 - 1657]

MCGLSVREAEIYNREYLPKLSRAQHYLTLTRENKGDLTG

>Tb8.NT.1_8 [1693 - 1998]

MNVGMISPPWIRQRMMQAASRRFDEVWSIMNGTEDVERKKERKYQTGNLKLHIVVKDAQIMKQPRIISTVSETYDRMGCVPFFVIGETQNWKKALIYCTTKD

>Tb8.NT.1_9 [2087 - 2203]

MFDIKSFLSQVKLPENIRAGFRYLSENEELVNLNRLPIS

>Tb8.NT.1_10 [2357 - 2458]

MSWEQNSYEHGEMWVTIIAGTTKVIQQFRVSLAI

>Tb8.NT.2_1 [151 - 348]

MSGSEAAAGKKVVVKRLFFNIKLISDIVKQVSHYTLKQRRNVLGIIEKMVFQRTREAETIFFPLCG

>Tb8.NT.2_2 [342 - 479]

MRISCTKTEFMFRPLKRAKDTWFIVRQPLPPLYRFTVGISSVTLLV

>Tb8.NT.2_3 [489 - 581]

MSVYSLFLWDFSILVVEKNCCACPCAQHSSP

>Tb8.NT.2_4 [641 - 871]

MVSVLVEEVWNSEYLFLIYTVPGHIGSIGVHIYIISSHTCITDLMKYGKCTGGIQKRLVIRFNTVLFICCLTFYSMM

>Tb8.NT.2_5 [844 - 921]

MLLDFLFYDVIYRIIIKLHTYSTPFQ

>Tb8.NT.4_1 [109 - 213]

MGIKLWYRIIIFFWGGKGGECDSKLMVLHSRGTDS

>Tb8.NT.4_2 [83 - 286]

MRTKGGDNRWELSYGTGLLFFFGGEREGNVTVSLWYSIPAAPTPKWTADHDGKPIGVPYRFPFRYAKC

>Tb8.NT.4_3 [502 - 675]

MPTTHLHCFSFSLNLSALYSCLRILNSKSLKQVRNSLPRQHPQPFFLTTLWGCRLYLT

>Tb8.NT.4_4 [829 - 987]

MLPGSGTPAATVLRRGYIFYVRDLFFFLLFLKNILSPLYLLFRPSPLFLSFFF

>Tb8.NT.4_5 [1165 - 1401]

MFYSLVPFYTIDSRAFVFSTFCFLFFFQTILLFSSTLCILGRFFFFSLPFVFLLYVFFVLFQPVRASEPFLGNSSLVLF

>Tb8.NT.5_1 [170 - 268]

MEERRVNPLVLLPTHFPFYCQCQMRSLLFASLF

>Tb8.NT.5_2 [232 - 312]

MSNALVAFCILILKQRQARMSGVILFL

>Tb8.NT.5_3 [337 - 477]

MLWCWPNPNPNPNPAHVIMLWKLPVRVRKNIRKGTHYQISVDRSSGP

>Tb8.NT.5_4 [946 - 1146]

MPVPTVSPTVDFIFLFHLGLRGRAAAVIRRGNARCRKVATGVIASLRWHGSGFRWKKKKLHRLHLCC

>Tb8.NT.5_5 [1086 - 1223]

MAWFRIPVEEKKAAPASFVLLNFSTRLCSRRGKVFGYLFLMFHLLL

>Tb8.NT.5_6 [1309 - 1422]

MLCCGGGAYEFSLLHLFKPRSSWKPRFCCNNTVQILKR

>Tb8.NT.5_7 [1091 - 1429]

MVQDSGGRKKSCTGFICAAELFDPIMQQAGESFWVPFLDVPLAVVMKFGEKSCVWAHVQRVRSRDFVYVVMFLCCAAGGVLTNFRFFTFSSHARRGSPDFAAITRCKSLRGNV

>Tb8.NT.5_8 [1425 - 1538]

MCKLVSSPRTFFFFALAVKYMSILCLRLRVSSSRAGVE

>Tb8.NT.6_1 [50 - 136]

MYVTMSDCLFKDWFKEKCTDVLLHAVFLA

>Tb8.NT.6_2 [54 - 188]

MLQCRIVCSRIGLKRSAPMYCCTPCSWHKVCVYIGNTCSYHQVAS

>Tb8.NT.6_3 [351 - 458]

MDRLCSLHQLFLAHSLLILLVSTTGKRLLQPVFVCP

>Tb8.NT.7_1 [55 - 297]

MGHSAKITRGGDKNRVNQGRREAKMRAQGAKHHTVSAKESIEIKRAHKKNTQAIAMELRAKSKAQSHAIVTMAPKVKGPEA

>Tb8.NT.7_2 [320 - 448]

MRATCNKLMSNNGWERLSSGNATVDVVVRGCKSMLGAKYSFFF

>Tb8.NT.8_1 [453 - 530]

MVTLLYIYIYIYIYSCPNTLCPFSIA

>Tb8.NT.8_2 [332 - 532]

MLNGLIENNGQTSPYELHVTLLLGDWVSASYPVSFIFVQADGYVVIYIYIYIYIFLSEHTVSLFNRS

>Tb8.NT.10_1 [13 - 90]

MPSFWEVCRIYLTHAISVCVIFLPYT

>Tb8.NT.10_2 [253 - 402]

MMQLMSCSFDMLQCVHMLKEFGGRPLALPHCYLIFVMDAFFFFAFVFFFG

>Tb8.NT.10_3 [237 - 410]

MPLFRNDATYVLLLRHATVRTYAKGVWGKTTRFTPLLSYFCNGRLFFFCVCFLLWLNL

>Tb8.NT.12_1 [271 - 363]

MRNLLLGCFADNNSHRVGVYNRRRLVSQQYG

>Tb8.NT.12_2 [363 - 602]

MKRKAIFTVRRGTMCVAQEILSTPFCQRFAHPTEAVRRXXXXXXXXXXXXXXXXXXXXXLWEVSLLKMIYVPCDVPHCCN

>Tb8.NT.12_3 [618 - 701]

MEYVIYASLLFPFMHLTTRLTSYYTRVL

>Tb8.NT.12_4 [701 - 820]

MIDTCDDDISPHVRMLCCTWRHGEIVILLLMFVLVLTCTA

>Tb8.NT.13_1 [111 - 266]

MRERHPTNDQNGVKVSFGFFIKTINFLMYVCFFFLPLPLGVLRLLFLLLLFL

>Tb8.NT.14_1 [231 - 350]

MIICLHAHARWRIKSWKKQGEKVILCEAIYRVTQGCLFFF

>Tb8.NT.15_1 [165 - 275]

MCSLELLRLAIVGGSIGDMLKSLVVIYSVSVAKCKTL

>Tb8.NT.16_1 [272 - 358]

MCGARAKAVCKCAKPTRSLLVHYTVLSRS

>Tb8.NT.16_2 [265 - 423]

MRDVWSASQGCVQMCETNTFITGALHRVVTKLRTMLGHTMKSTRITQRRNIVG

>Tb8.NT.16_3 [243 - 578]

MNEKRTAHERCVEREPRLCANVRNQHVHYWCTTPCCHEAKNDVGSHNEINKNNTTSQHCGVKKRVRACKSISAARKNVRVPTKFKMTSSEGGGDDQLPLNCAFLPTRGAVKC

>Tb8.NT.16_4 [582 - 656]

MTILFFLFSTEPGAVPLMNALLSCG

>Tb8.NT.16_5 [637 - 771]

MLSYRAVDAPNLRDCVDPKNMGGKGLSDLSRLFLFFILVIDCETS

>Tb8.NT.16_6 [575 - 802]

MLNDHSFFSFFNRAWCSSTYECSPIVRLMRPICVIALIQKIWGEKVCQTSRVCFYFLFWSLTARHHSYCVCYRAPG

>Tb8.NT.17_1 [20 - 136]

MLYFLTLRRGEGKGTGHVKVCHYRNLREASGAENVYGVL

>Tb8.NT.19_1 [16 - 114]

MLWQAKRTQGRCSSLLNTGFVVIISTTVPAVVV

>Tb8.NT.19_2 [229 - 327]

MYPQSLPLHAYGASAAAIGCTRHGYICVAWAMT

>Tb8.NT.19_3 [369 - 461]

MASFALVASSFAGDIFAWWAEGGRRRRNACL

>Tb8.NT.20_1 [115 - 276]

MSRGTECVRCTDTRSRRRSNGTNGGTSDSFISHSFSSPFSVSRVGSRVSSSVRL

>Tb8.NT.20_2 [182 - 283]

MVEPRIPSSPTHFHPHFLSPEWDLVFRRASGCEW

>Tb8.NT.20_3 [105 - 386]

MRGYEQRDRVCEVHRHALAETEQRNKWWNLGFLHLPLIFIPIFCLQSGISCFVERQVVSGRTAGGDVHVFNWQCRSPRYSPDVSGEGEEREAVS

>Tb8.NT.20_4 [301 - 396]

MCMYSIGSVDPHDIPLMFLERVKKGKPFPSAT

>Tb8.NT.23_1 [137 - 244]

MLQPVYLLGRCVYVCVYVWLLVRWSGSRAALPLSIH

>Tb8.NT.23_2 [166 - 273]

MRIRMCICLAACALEWLPCSSSIIYSLTHSGHTVAM

>Tb8.NT.23_3 [328 - 402]

MWICVFFFFLRPEVEVRTLQFYSLA

>Tb8.NT.30_1 [289 - 372]

MRSTTIAVTHFLHQQRTCLCTLNSTGNN

>Tb8.NT.32_1 [97 - 204]

MISFRCEKIMKEKKEVKVKLLICAVPRGIITEPLHN

>Tb8.NT.32_2 [360 - 437]

MAAIKRKASASELNNCAHSYHIESPK

>Tb8.NT.32_3 [457 - 579]

MVLFFSVGICFCFISCFIFYLNQSAVFECSTLPHVNVLCSC

>Tb8.NT.32_4 [540 - 647]

MFHVTACQCPVLLLEERCGRESDNNTQVMSVLPEWI

>Tb8.NT.32_5 [757 - 891]

MLSWGSANPNRSLEIKWFILEAFVCCVYCLGKGRVEVGVMGASAT

>Tb8.NT.32_6 [1166 - 1309]

MLTSGHILGEWMMSSRGRGFIYSFHIPGASLGMEVGAGGGEKVGLCSV

>Tb8.NT.32_7 [1162 - 1320]

MNAHKRAYSWGMDDELQGKRIYIFISHTGSVLGNGSWRGRRRESWALQRVGMQ

>Tb8.NT.32_8 [1537 - 1626]

MRDKGEHASNATINSNVSSATGLLFNSASE

>Tb8.NT.32_9 [1982 - 2095]

MGEGYVSSVVFYVVVMVEVFRFIFCLLLKQCNDVLQPL

>Tb8.NT.32_10 [2223 - 2567]

MLTYCLLGRDSEVRRRTFTPDRRWRMWEVEDVLGRMKEKVEGERQKFCNEMNSLFLKELSLGALGAAERQRFIELGDAYQENCVFLERLSNLKKKQYEIPYEPRKLGAFFNTRDV

>Tb8.NT.32_11 [2744 - 2833]

MQRNVYYRLNGSAWGCTLYGQYIPIKIFRR

>Tb8.NT.32_12 [2782 - 2871]

MGLHALRTIYSHKDFPTISLAAVQRKATVA

>Tb8.NT.33_1 [327 - 413]

MCTVCGCSGIGTRQQVASDFSVCWNALFS

>Tb8.NT.36_1 [67 - 168]

MLDQIGSAVSERFTPPPCPLGCMLVSIECESPRK

>Tb8.NT.36_2 [183 - 260]

MLTAHTVFTYSLKCKQGFTCFLLTYS

>Tb8.NT.36_3 [266 - 358]

MLPPPNVGRGHILKIFWRSAQYKMELWLQSC*

>Tb8.NT.37_1 [23 - 457]

MFSLREILSQLPSDGATKWIRVENVKSPMSSLDVSQLFPDAVSWDFLSSVQRTDYLVTFRSESFARKAMRSACGFSYLGERLKFSYAEPGDIAEGASPARVTIAHPRLYLLPPEFSYEERVAEADRIIASVSTTDSYNNAWVDTY

>Tb8.NT.39_1 [56 - 307]

MTRIPGVITSLSQGKCLSFASAVHYSTTTTPVLQSKLTLSQKRLRPKRKPKLGPSVPLCILILLGASYMTYTTRKSSNDDRMFR

>Tb8.NT.41_1 [138 - 212]

MKQEPCQVPYALFHLQCEKSLRLYF

>Tb8.NT.42_1 [135 - 224]

MTSSRGRKKELYSCLNLLTGDPCFRELRLL

>Tb8.NT.43_1 [132 - 221]

MCCEGIEDTNPRRLPKSGETLAKNNIYIHI

>Tb8.NT.43_2 [231 - 350]

MRRHKRRYGVRLILFILWRAHLYAVGLRFFFFFWNGTPQK

>Tb8.NT.43_3 [334 - 519]

MEHRRNRRDLGDASGGCVGLMSRRNHDVQRRYWRENRKRYTVIQPLLSDRKGSQPLDLRGDA

>Tb8.NT.43_4 [589 - 726]

MLACVSGGKAAYPLGRAPSTPPIPETVRRDVASFFSFPTADSTGMC

>Tb8.NT.43_5 [677 - 823]

MLLVFSHFPPPTPPECASLYPLLSPFGASQPTSRRVDGCHLYLNLPSFS

>Tb8.NT.44_1 [147 - 236]

MVVLSPSKQTRSRILLMNIALTGRDTSVDL

>Tb8.NT.44_2 [380 - 553]

MVRFFRGLHTTFLHSIPQSPFAISFWDKLFCCPRVNSELLSVVEKGLTEDVGWLSRLE

>Tb8.NT.44_3 [713 - 823]

MHTYSVHQLVCACQSKYRSSRRVIYTKRRTMCLLAAS

>Tb8.NT.44_4 [679 - 921]

MVTGNNFFAISYAYLFCAPTCVRVSVKISVQSPCDLHKAKDHVFAGSLVGCKLRSRRCRILMCFIEMCELTACLVHCTVTK

>Tb8.NT.44_5 [1105 - 1191]

MAHVLHIYKKSSPICIAWLSNACVGSIWP

>Tb8.NT.44_6 [1155 - 1253]

MAFKCLRWFHLAVRVLKHRHGTFLSKFFWYWGV

>Tb8.NT.44_7 [1112 - 1408]

MSSIYTKRALLFASHGFQMLALVPSGRESTETPSWHLFIEIFLVLGCLNESYDDVRSVFRRTKGFLICHRYWWMGLSTGYFFLLVLKPLNIIFIVTLLD

>Tb8.NT.44_8 [1774 - 1893]

MELDRVVWISTGVRRGELPAVSMVVDRKFAQFLPDVNLNA

>Tb8.NT.45_1 [117 - 407]

MKRFIQSRRVWNLMDIRRRPPVLNRLRGVLQFAQQPGALSRHRQGGDYNCSMRVSIYSRPGKVSRLNNADATWHNRSRKEKPPDFDPSAFRRRYRDS

>Tb8.NT.45_2 [325 - 447]

MPHGTTAVEKKSRPTLIPRPSGDGTGTRELKPVVKTIHFRL

>Tb8.NT.46_1 [225 - 335]

MHPYSGPAVNSGGGGGRGWQRPDTSEVEVCRRGMREE

>Tb8.NT.47_1 [298 - 435]

METNKIRERTYIYIYICVHILCRKEAEIYTEAYRSKIESAEPRVSA

>Tb8.NT.48_1 [105 - 224]

MWHCCAVSGSRRTCASPVETGSQRCLRPMVPCASREITGQ

>Tb8.NT.48_2 [272 - 367]

MRTKACRQTPFPTISRPALVSPGYDFPGNLMK

>Tb8.NT.48_3 [376 - 576]

MMRHLSPLSLATQATELSEKRAIGGGPCVHPTWTASLTQANADAGPLVGGRRAPLQCFVIASLPVAV

>Tb8.NT.48_4 [543 - 707]

MFCHSVAACSSVSSKWNEEVRTSFPNSPVIPNKCRRGVKCVIVWLIFETECYSFL

>Tb8.NT.49_1 [535 - 633]

MVSFHHLRQCVGDLVVMMSALHAERRGFEPRPA

>Tb8.NT.49_2 [670 - 795]

MVPINEIVEEHVALLAQSVERTALNRVVVGSSPTGGAFLRFP

>Tb8.NT.49_3 [849 - 926]

MCQQAFLQQRHSDIVVTKGEVPSSGC

>Tb8.NT.49_4 [936 - 1109]

MLLAEGSCGGRRIPPPPPPPPPRDRACELFGYTPHTLFPVVCHGLIYATRKRANRPLT

>Tb8.NT.49_5 [1109 - 1222]

MKRCVGPCFRGGRVIRTLGVPSFLLVSDVTVQLSFPKD

>Tb8.NT.50_1 [125 - 310]

MQKHDIYQCSRLCEAPASTYFLLPQTHTLHPSPCGETMFFPAHDGSRVINCSWAESMYPGPL

>Tb8.NT.50_2 [252 - 506]

MMGAVSSTVRGLKACTRGPSDGSACVVKSPPQTRQWVGVVRLYAPIIPHWSTPQAPDLPTLTDVHEQRNTLGFPAAPGHRFFSQR

>Tb8.NT.50_3 [439 - 672]

MCMNRETLWGFRLPRGTDSFRRDKFPLACHRSGFTFCGSRLQTNRHPIFLFPTFFFHRCLFNHPRSVQKRLVKKENLI

>Tb8.NT.51_1 [111 - 239]

MSMGWSELSKVTAASVKTAPGLPNENGKLQVQKHQHTLTHIRI

>Tb8.NT.51_2 [187 - 285]

MANSRCRNTSTRSHTFAYKQPPPSQCRAKENTL

>Tb8.NT.51_3 [261 - 341]

MPSEREHAVTYGSGETQLSLTLLCGHC

>Tb8.NT.51_4 [350 - 583]

MGRKIRADSWKNNTNRKGKEWWLRGSYVRAFKLVLSAISWGNIALVLVPLSGATAITLEGRMCKATYLHSLLKGGRSS

>Tb8.NT.52_1 [63 - 197]

MPTFRGRYERSKVTPFCLATEAGRDRHELDRHSRTSRRMAVYGTG

>Tb8.NT.52_2 [377 - 478]

MLSLLVCYQSVSALSGDACETRWVPAQQRDSLWA

>Tb8.NT.53_1 [2 - 91]

MSFKKVLSCRIFTEIRRVYMWRLFYLGYLF

>Tb8.NT.53_2 [255 - 335]

MLMFLGAIGASAVSPDFFFLKQISFEC

>Tb8.NT.53_3 [332 - 427]

MLTFAWFTWCYRISRVHHGVWSIVCCDGAKYH

>Tb8.NT.53_4 [384 - 800]

MECGALYAATVLSITDSEGTRELRWSTLYRLFSTQSLSAPSWILTRVLRFPTPEGWEHRKWKSNCNQARLRRFSLPDAVFFFLLCRWLNFFFSCCALKMRSEWKEERCSGRGAKGVGCHEEVISRRMIARILVYINDQQ

>Tb8.NT.53_5 [964 - 1068]

MRRYITVIHVTFFALFFTDESRHRETSCLWSFNLR

>Tb8.NT.53_6 [1019 - 1132]

MKADTVRHPAFGPSTYVEGCVRVVREIRCVSSLDWVTE

>Tb8.NT.53_7 [1154 - 1300]

MRLQCNESFHQNRVMPTGTLDMKAVDVKPFGPFFFTLSPPFLHRAYILR

>Tb8.NT.54_1 [141 - 272]

MFRCSRLFHQVKLGFTKAQREHQRSKRIRPNVCYRPPILKKRNQ

>Tb8.NT.54_2 [484 - 591]

MKEAKKAYKKIKSLIVKSVIDSCIIIRRAKTLIEHS

>Tb8.NT.55_1 [17 - 316]

MLPYPAAPFRLLCRRINVCVCSSPCVPHRGSLIISDFLLTFVRGMLCALVPQGGSAGWFRCVLKVRMMCDMSVGATLCVRVPGCDSYGFHCVADQNVSSV

>Tb8.NT.55_2 [319 - 450]

MSYCVNAVEQTLVIPTSGHDVRSRPVEVDISNFGVCTGCVFAFY

>Tb8.NT.55_3 [688 - 870]

MRKDTPSSFLHLSRVRRSERRGIFYPPTELPWCKCLLSETRLGVRRKRWCAPLPHVSLFPP

>Tb8.NT.57_1 [425 - 790]

MSALYLDETCPRVCVCVCVCVCVCVCVCVCVCVCVCVCVCVCVCVCVCVCVCVCVCVCVCVCVCVCVCVCVCVCVCVCVCVCVCVCVCVCVCVCVCVCVCVCVFAQCIASHQVGLFSQGSIF

>Tb8.NT.57_2 [742 - 990]

MHCFTSGRSVFPRKHLLKVTRVRSIGKFPWSSNSMKWLTYLGAEFCCACSKNVIRHPTTCCTFFFVAVFPSLLFVNVGTIIFY

>Tb8.NT.57_3 [896 - 1018]

MLYDTPPRAAPFSSWRCSLPFCLSMLELLYFTEAWSDMFCT

>Tb8.NT.58_1 [167 - 304]

MLHSELVIPARVIFVVSFSFWGCVCVCVCVGWGWRRVVRGTQREFL

>Tb8.NT.58_2 [148 - 336]

MTQPPSYVAQRVGDTGTSHFCSFFFFLGLCLCVCVCWLGMAKGCERNSERISLTTDRRWGDCV

>Tb8.NT.58_3 [435 - 518]

MGRIVEKLSAPAVCTRPKAFPRRGGASL

>Tb8.NT.59_1 [104 - 250]

MCCYFTYWKYALMMYSSQVRMHSSVRGRPAASMQSLPCVVFPKTTLSAR

>Tb8.NT.59_2 [480 - 584]

MGLPSTISKKGAPVRDKIALVPLIPSAIWDLFCPF

>Tb8.NT.59_3 [458 - 787]

MGGVRVKHGPAIHYIKERSARKGQNCFGPPHPFSHMGSVLSILIHPPGGKRVALGIRYFSPTTTTPMPLIARVFFFHVFVSGRRSVIWLRTGEKSGGVCTIIYCPVFSPR

>Tb8.NT.60_1 [13 - 261]

MSSCVLLPTFGIQCCSALRPNLTKKLGHLKNVVSIKPNRYTAAPTPRRCLPHFGFATIVRLAKRQKKLHRNDRKLKGCISPRK

>Tb8.NT.60_2 [6 - 302]

MTNELMRPVAYFWDSVLQCVAPKSDKKTRTPKKCSEHKTKSIYRCTNAETLPSTFWFCNNSAAGQKAEKIAPQRQKAKRVHFSEKINVVLIQGINRGSV

>Tb8.NT.60_3 [489 - 611]

MYAEGCILKLEPLVIPEAGFHPVLYQIIAANDRVQLVRAGV

>Tb8.NT.60_4 [580 - 762]

MIGFNWCVLECKRPREDEAPCEFGKKHPYVCVVWKQGCDKTRSTKSGYNRAGEFPHKNSSI

>Tb8.NT.60_5 [665 - 766]

MCAWYGSKGATKRVPRSLGIIELENFHIKTVPYN

>Tb8.NT.61_1 [201 - 311]

MRRVMHTILTGKEFFFMPHIFPHSRHHQAYCTVPYVG

>Tb8.NT.61_2 [304 - 429]

MLVRWVGVCFSFSVEHACRYVFAPYGGFFNHGNAYFPLEWKL

>Tb8.NT.61_3 [188 - 481]

MFGVHAPCDAYHTDGKRVFFHAPYLSPFPPSSGVLYSSVCWLGGWGCVFLFRLSMRAGTFLRLMVVFLTTVMLTFPLNGNCKGGKAHTVSVIAALELI

>Tb8.NT.63_1 [93 - 167]

MYFRSTEPQGVKRESDLFCMDICTC

>Tb8.NT.63_2 [185 - 415]

MCVSILSTFSIPAFFPRLTCLASTSDANFTLLIILLSYYCARVDISNRWPANPLRNVVSFITSVVSHSGGGAFCSLN

>Tb8.NT.64_1 [185 - 286]

MILHNIIQYRKALCIQLTYGTSKCCSGFTRYLTA

>Tb8.NT.65_1 [192 - 371]

MSLLSFNKEGKGERRIQKTRRLEEGYGALGTGIRGNGTSGRFFFFDLSLIAIGVVYFLHL

>Tb8.NT.65_2 [298 - 525]

MEQVVVSFSSTFLLLPLVWFTSFTSKMRRSTLFGPPPSYTSVVFFLLLGGDRLSKFSQHTNRADAEGQIHQGSTNT

>Tb8.NT.66_1 [253 - 552]

MYFVVMRYVGMCDYRGTDASSLSGALLFSRNVLRRRLKTSKDHSTLLLQLESYVCVGGGRLFRVIGFLILKEVGDAHRCSSGSGLSPGKGCAGLCHRFPW

>Tb8.NT.66_2 [476 - 580]

MHTDALPVLVYPQVKDVRDCVIVSHGDVRSALLRR

>Tb8.NT.66_3 [580 - 663]

MIVYLVLVFDLWGICRSEVFFEFIGSVD

>Tb8.NT.66_4 [683 - 877]

MAVGDGNVVNGLGSTSCLLLLNHLKGVMIPRSWERPWSNCFFRVSTVCTFHLTFSAPVMKAMRYA

>Tb8.NT.66_5 [1004 - 1105]

MTLLYTATRNVSKRWDYCVGTVENVRGKTRQRLN

>Tb8.NT.67_1 [253 - 615]

MYFVVMRYVGMCDYRGTDASSLSGALLFSRNVLRRRLKTSEDHSTLLLQLESYVCVGGGRLFRVIGFLILKEVGKTKRANDDGYLKCAGGCKGHSADKVRHFDGYPCVMVGYIAFKVQSVG

>Tb8.NT.67_2 [622 - 714]

MRDLVHGWLLVGKDCVIFVCTIFYPSACVSR

>Tb8.NT.67_3 [820 - 894]

MSVAASFIVIDPQPVDCMRAVVVDV

>Tb8.NT.67_4 [618 - 920]

MDARSCSWLVASREGLCYICVHYLLSKCVCFSLSERLLCCSTRYLFFCNANFWSFCLDFNPMWLSSTNECGRVIYCNRSSTGGLYAGSRSRCLKRQYFASG

>Tb8.NT.68_1 [253 - 615]

MYFVVMRYVGMCDYRGTDASSLSGALLFSRNVLRRRLKTSKDHSTLLLQLESYVCVGGGRLFRVIGFLILKEVGKTKRANDDGYLKCAGGCKGHSADKVRHFDGYPCVMVGYIAFKVQSVG

>Tb8.NT.68_2 [622 - 714]

MRDLVHGWLLVGKDCVIFVCTIFYPSACVSR

>Tb8.NT.68_3 [820 - 894]

MSVAASFIVIDPQPVDCMRAVVVDV

>Tb8.NT.68_4 [618 - 920]

MDARSCSWLVASREGLCYICVHYLLSKCVCFSLSERLLCCSTRYLFFCNANFWSFCLDFNPMWLSSTNECGRVIYCNRSSTGGLYAGSRSRCLKRQYFASG

>Tb8.NT.69_1 [250 - 345]

MELPHWTPCGLLDTHSSETPCCRRAPAPFTPP

>Tb8.NT.70_1 [380 - 484]

MNGLWGTAAVSWNGVCVRSYSLYEGILSYAYVCRN

>Tb8.NT.70_2 [417 - 506]

MEFVSAVTLSTRASFPMLMYVAIRKLCRWR

>Tb8.NT.70_3 [376 - 516]

MHERLVGDCCRELEWSLCPQLLSLRGHPFLCLCMSQLENFAVGGNLF

>Tb8.NT.70_4 [574 - 705]

MGMSGSYIILSFQRLESTNIHLPFFVSWVGLATPVTIDCFVLAV

>Tb8.NT.71_1 [215 - 301]

MLRCLSFHLPRRRGVATLLTGVLVAGGYV

>Tb8.NT.71_2 [126 - 314]

MGLENVPTFIGKIILVILLMRSFFYVVSFSCCAAFLFTYPDVAGLQPFSPEYWSRVDTCRVRY

>Tb8.NT.71_3 [652 - 822]

MMRCSPTFCVLSDRYAYEWVREKNHKIDSLQPNHVGWVSNESHRGEDFKNLVSTWRK

>Tb8.NT.72_1 [145 - 222]

MCSLRQTLRDVGYLKTTLRKEEEKIC

>Tb8.NT.72_2 [239 - 337]

MVTCCCCSIPDRGTNEFVCLMRWQAIQLETSGE

>Tb8.NT.72_3 [459 - 551]

MIKVGLAPEIILVGNLLPYNILSNSLNFLQI

>Tb8.NT.73_1 [282 - 356]

MRCLSHFFFFARGGVHVSTLLGLLY

>Tb8.NT.73_2 [448 - 534]

MELWNVALFFFLCVCVCVFVLFFRFIAGR

>Tb8.NT.74_1 [23 - 148]

MISCAAVVCSPRASLRDCGKLGVWVTTSGHAVFSEVISPVSY

>Tb8.NT.74_2 [111 - 311]

MRFSRRLFHPYRIDVSSLERAKRQQGAQGIRTSPRVSLIHCAKRAWRKAMSGYEFFFPSSGKGGPAK

>Tb8.NT.74_3 [325 - 480]

MQTEHLCLYRCCGLLTGPVMCGRTCEHYVSIVPLRVLFPPLRFCSTHLTFAF

>Tb8.NT.75_1 [120 - 227]

MVLLQLAASFFSSQEKKSREDSSWSLLLRTLLVTVC

>Tb8.NT.76_1 [147 - 254]

MWKYQRRLSGLLLLLILLYLLVGNHGLFTLRSRIKI

>Tb8.NT.76_2 [143 - 469]

MDVEVSASAVRTVVVIDTAIFVSGQPWFVHSAITNKNLTNRLVATVAVVKKGSFFIRCCRSYVVFSISISEESTSFSAFCTCKCICMVVRTYVDRLTLMWAKYFSFPTP

>Tb8.NT.78_1 [99 - 230]

MCNYVADPRERRRTYSALPQFAGPASEECPLVRTGVTRRDHGWG

>Tb8.NT.78_2 [430 - 570]

MISLAVVCGSHIDGVLGAREKSRCGVLLPDLATFLCLTLRASVPIFF

>Tb8.NT.79_1 [158 - 253]

MPRAGMHWTERECSETPLLSIVGLTLPACQRR

>Tb8.NT.79_2 [13 - 318]

MTFKFFYNVPRQVHSFRGGSRGPRRAEVFNRLTRQQFRRRLMSGTAASNAKSWDALDRKRMQRDSVVEHRRLNVARMPAAVIRQKLKRGPPHKGDDKYLLRP

>Tb8.NT.79_3 [377 - 484]

MRTLYVLANYIIPFLFPRTLLTICSGGFLVGILHGG

>Tb8.NT.79_4 [390 - 539]

MFWLTTLFLFSFHALCLLSVRGVSSLASYTEVDLESKFVGRLREICTMRL

>Tb8.NT.79_5 [664 - 792]

MQGCCALSANCHCFLYKGYFKSLVFIAKVVTAGVEVNGASLSA

>Tb8.NT.79_6 [773 - 856]

MELLFQLNNQKKRSMRHLFCCVINTSCR

>Tb8.NT.80_1 [472 - 561]

MMQRWRSDFFTDFVCEGFRPFILTCNIAEE

>Tb8.NT.80_2 [623 - 742]

MSCGGDVTEGRLCFFISYVAFLIFLPRCQCTHIKIHISFV

>Tb8.NT.81_1 [43 - 117]

MTSGRRIPRKLPPYGSDGQLWRTSW

>Tb8.NT.81_2 [83 - 178]

MDLMVSCGGRVGENAFRLWCDIVFMKVVRNFL

>Tb8.NT.82_1 [128 - 232]

MVEEVLGAMRLLPPREPERDREGILGCRKGSCLRS

>Tb8.NT.82_2 [99 - 266]

MGYGTSSVTPWLRKCWVPCGCYRRGSQREIERGFWGVGRVVVCVANEGFPPSTILL

>Tb8.NT.83_1 [185 - 307]

MSGWSSRRGTFFGLYRVVVNVTSFEFVSGITAVVSTIKCSV

>Tb8.NT.84_1 [144 - 218]

MLNISRVDICDMCDYVELCLVTYQG

>Tb8.NT.85_1 [47 - 214]

MMSGPCAGAARSTKAWQKTNRTSQPRLQVRKLRMTVRIANVKKWKESHHKKGEIPA

>Tb8.NT.85_2 [366 - 497]

MLAHTPVRRTVRISPHFQVIVAVCSGGNKLYTPAHDVPTPRKRR

>Tb8.NT.85_3 [472 - 696]

MFPRLASEDRKKKNEYRKDPSSHTLRVARRIQTILLLVLPYLLTSFDFLSSQISSLENRVTALHEAENFTARTAL

>Tb8.NT.85_4 [662 - 760]

MRRKISPHVRHFSNLCVQPSFYQASVISPFLSF

>Tb8.NT.85_5 [963 - 1238]

MYLSRGGGKQPTGGVQPLVLAPCSEPCLCSGGEASGITKGVCQSVVYFLVKAESMLHNSLRFHVTNFLNDKGLPCKFESERMESEHPSPILQ

>Tb8.NT.88_1 [96 - 206]

MQCCVVFSVQSTGIESLSDVFENRVIVSGLSFCWMRE

>Tb8.NT.88_2 [303 - 389]

MYAPGSGIFYPSNDCTVIAGGIGNEDGFS

>Tb8.NT.88_3 [340 - 525]

MIVPSLLGESGMKTVSLDINSMFCLNFISLPRLVVKLSRFASHWVSAINIFWSSATAVTQLS

>Tb8.NT.88_4 [1173 - 1352]

MIISKKSKENIASTKRIKKKKPTSSTPTTKFIYKTLPSYVHIREKISTKQQKLYKKTHLL

>Tb8.NT.88_5 [1288 - 1365]

MCISGRKYQQNNKNYIRKHIYYNNYL

>Tb8.NT.89_1 [400 - 501]

MNPLVAVSCAEPCWPPSHRSTGKCNYDERSLYYQ

>Tb8.NT.90_1 [119 - 268]

MVHIDIHKYACAGCSSAKDKTTGYRDPGNVLRFGHRAWFCKVLSLMIEGI

>Tb8.NT.90_2 [539 - 712]

MCCLRFFPLPQSVAGDTSLCAVTMRLHVFPLLLRVPITLKLMAPWRLGVISEERSLHR

>Tb8.NT.90_3 [595 - 825]

MRRHHAATRFPLAVTGTYNPEADGAVAIGCDFRRAITASLMAITVWWWLVERCFYHSCIWLFSLLYRHLPVLLWVYL

>Tb8.NT.90_4 [833 - 1051]

MCLPYSFVHVAVRLILICFFCSGEDLPTPCFLHIFRCPPANFVTSDKFYCIEFVQLHGDFWIMYDCSNSGMVH

>Tb8.NT.90_5 [1002 - 1139]

MEIFGSCMTVQTAAWCTKLQEVAIHSVSSSVSACVTSLYAAFSVPS

>Tb8.NT.91_1 [20 - 109]

MRQKKNYLFHSLRRCTTIVVSHNLQLHVPE

>Tb8.NT.91_2 [116 - 235]

MKIMISLLCRKNHTCCSDDGTGSEQSVLKKKMFYSPIAEG

>Tb8.NT.91_3 [342 - 545]

MISQRCFIRWRGLTISMQVNGERRYLLMLGGHCACTRRRGELFVRFPQELLKIFLKDGVFVRLPYFFF

>Tb8.NT.92_1 [299 - 469]

MVAFAFHVHYHKLPPVNYLSLFLFLFFFCFSPAHDCITSCNHAGSSHVVVMLVVSLR

>Tb8.NT.92_2 [485 - 583]

MSLVHKHAGNRNALLGNSMLQTVEMLSRHSHSL

>Tb8.NT.92_3 [469 - 591]

MMMMSNVFGTQTRREPQRSVGQFHVTDSGNAKSPQSLTLSF

>Tb8.NT.92_4 [715 - 858]

MGALRLVFEILLTCHALLSQRVHSHNIYIYIYIYIYILIICSALVTCR

>Tb8.NT.96_1 [196 - 294]

MSLFVCLLRSALKCTEAEKKSDGRSVWWKTPRW

>Tb8.NT.96_2 [427 - 567]

MSPCGSAVMPDSTKRKNQQPPADKFFFSFPLYLLPSLPLFLCVPSKF

>Tb8.NT.98_1 [90 - 230]

MPDPYQAMMALMGMVAGGVMARGGRGGGRGSRGGNSQRVMGGFQPFQ

>Tb8.NT.98_2 [401 - 547]

MEWNGGTMTKKRCFDCCCHSRRVIVLIPIALSSSTPFFLFAVLLFFFLN

>Tb8.NT.98_3 [600 - 689]

MPLGGRRWCRKSNTERNTDLKSTVCGWLCG

>Tb8.NT.98_4 [862 - 1044]

MERQNKKDEKCVVQVMKVWREMSRCAIITFVYHHFSFHAFVCLFVFVFALIPLSIASMMLW

>Tb8.NT.98_5 [933 - 1313]

MRHHHICVPSLLLPRFCLFVCFCIRSYPSFHCFNDAVVTENPLGRPVAWHRCPSNRFFQRGISFTFPKPLILPFLKKKKEKKDRKVWGVGKNWKRDSENYGEDKRYRETNSRVQLHECQVVQSLNEK

>Tb8.NT.98_6 [1231 - 1326]

MEKISDTGKQTHACSCMSARLFNPLTRSDKII

>Tb8.NT.100_1 [36 - 218]

MNSGILCRGMFGASITEEGGGLSGNRVRWRVYVDQGETPQRVRERVLAHAYPPSKDSKYFS

>Tb8.NT.100_2 [181 - 264]

MPIPHPRIRSTFPKCGLRAALRWGRRQC

>Tb8.NT.100_3 [309 - 383]

MWVRYCGANSQRVGLVGPWRRLRLW

>Tb8.NT.100_4 [272 - 394]

MDSLNLKVLCTRYVGAVLWCKQSARRPGGTLASFETLVKLR

>Tb8.NT.103_1 [185 - 271]

MGAARGAHKSLTPSERCYLVNQANNITSV

>Tb8.NT.103_2 [377 - 541]

MQGIVLDAIVQGCLLFLAITRVTDLDAALRFAKSYPSPQEVIDRNVFMCLLSGTM

>Tb8.NT.103_3 [510 - 674]

MSSCASSVERCDDNTSASCTANLGSGTAFHSRVICQQPPSSGVATVLIAFLCARR

>Tb8.NT.103_4 [936 - 1103]

MNGSYAVRGKLPRFSTCRRLVANGMKELVTVVRELSEVFRAPPHLSWTFSCFLAVA

>Tb8.NT.103_5 [983 - 1105]

MSATCCKWYERACNRSSRAIRSFPRPTTLVVDIQLLPRCGS

>Tb8.NT.104_1 [42 - 146]

MPIWCARQYNPGVTTSLGKITNGSIEKRSVAMKGG

>Tb8.NT.104_2 [382 - 462]

MKLSSLLNHKKNDLITNDNHVAAGGKH

>Tb8.NT.104_3 [668 - 766]

MRKQAKKLFAMNPLPSVCDSLLSAICLNRLQQF

>Tb8.NT.105_1 [246 - 329]

MADGGFFFSSGCGVATLRCVTRSNKMTL

>Tb8.NT.106_1 [22 - 102]

MWCRDGESVRSHTRKTEMVTDRTENLY

>Tb8.NT.107_1 [169 - 252]

MLMYVSMTSVRKTNVPTLTRTRTAHACC

>Tb8.NT.107_2 [156 - 467]

MIGLNAHVCKHDQCTQDQRSYAYSDKDRTRLLLKTQLKDVSHTVTRDRTLGMQLFSMGFSVSHATKLLGSFMSRSHQHRCGGCCDSNVLHLTEGFLGRVLLILC

>Tb8.NT.107_3 [415 - 495]

MCCISLKGSLGAFFLFYVDCYFWIAYC

>Tb8.NT.107_4 [667 - 747]

MIVKATKEASSVHPFVCSTAVNIHFVH

>Tb8.NT.107_5 [648 - 767]

MPRIRTNDCKSDEGGKQCASLCLLYCSQYSFRTLNPLAHS

>Tb8.NT.108_1 [34 - 252]

MTHKVRLDDGLVEVDTAVRGQFVLYLDERSGVWSVAPAEPPSSTVKSNPESALAEVAVKEDSIDDEALMFFAD

>Tb8.NT.108_2 [470 - 544]

MQTALDISKVRWQQLSPSLSDTPPI

>Tb8.NT.108_3 [647 - 727]

MGRKFFTIWIQQCSPNRYTARASQLLP

>Tb8.NT.108_4 [463 - 819]

MVDANGIGYQQGAVATTFTFPFRHPSYLRERLDTLTYSEMGILKVVGLRRNKISVQSLCVLDGKKILHDLDTTVLAKSVHRQGLPTAAVMEICWWLFRLAQAFCNVQIVCPAYRPLVNL

>Tb8.NT.109_1 [465 - 560]

MKRSSHPQNSAFIDGRICCTVVKGTSRQNHRL

>Tb8.NT.109_2 [632 - 745]

MSSGEQHSIRDVLRAKWIKLWGGGLCVSLAKGVARPTG

>Tb8.NT.109_3 [691 - 846]

MGGGVVRISSEGSRKAHRLVWYLANGNSKYRANLGGGIPVAATTLSSSGWKF

>Tb8.NT.109_4 [962 - 1045]

MGENMKLFFERRQVCTGTYVYHVILLRR

>Tb8.NT.109_5 [1066 - 1197]

MLGARFPSVVVCWSSFLCVHVKRMNFFGLLKKKNMFRVTVGSVS

>Tb8.NT.109_6 [1206 - 1349]

MRFLGTQTNERYCHTIHKKCTIFRLTGYFYPLLVDHLSRRFFLLQMFL

>Tb8.NT.110_1 [137 - 247]

MYISFRRPFCFGGQVMKVLFASSFDGTKRVNIAIGRR

>Tb8.NT.111_1 [7 - 192]

MFISLKCIAASLTGSHAVAPNSRAYKKLPMHKRRAFPPLRLWEIMWENRIEEGGSLGWVIGA

>Tb8.NT.111_2 [364 - 606]

MRWVTRLLWCVFVWLRESEGFSMLSVAAQYYVNLLVLGRAAEPQKCNCTFEEKAGALFKQPERWFTVDCANQNDVSPTKSG

>Tb8.NT.111_3 [581 - 694]

MTFRLLKVDNADVAARVEANSLFSNRRCKNTRPKMEKQ

>Tb8.NT.111_4 [609 - 926]

MQTSQQGWKRTLFLATAGVKIHGLRWKNNEGKLPRDTGTFCTTQNWRMLTKTVAMPDGGSADPPSPKGAIHSVEAHPHSNLTSGNNVRDSRETSTSPVEDEFEGKE

>Tb8.NT.111_5 [1230 - 1436]

MGSYSFGLALIRRGWESNRPVILRALLTLLNLAGGGCSGMKMRMLSLALFSTAFSGFIQGSAHTNESPR

>Tb8.NT.111_6 [1875 - 2006]

MWLAAAPRSRAPRQGTWKLEQISPVSILRDGWGQGDLENLRVPW

>Tb8.NT.111_7 [2078 - 2272]

MHVLMASCDTGSAGHYMDVEQFKPKVLEGPLKQYNVLCFPRRRDRSHIENKKYINSVSKTSFEKL

>Tb8.NT.111_8 [2387 - 2620]

MGKSGVADSEAASATCAADCKAGKLWRPPTPLITYFLLALVVYTKIGKSYFLMHTVLIAIIMTTKEVVIESKVTPHTL

>Tb8.NT.112_1 [93 - 269]

MVAYLSRCEAQQDVFSPLRKQSGHPVRFYRGLIDAHDVAKKRSERNGCLWNAWKSKCGQ

>Tb8.NT.112_2 [244 - 489]

MLGNQNADSRAADAGGNKMLCYSVGCPLTGKQVPGSNSSTCASSDGAGARPCWPTKVPSVGTTHLLFITFDDTPSLKPQGYR

>Tb8.NT.115_1 [122 - 277]

MCTSEHAKRCFVWVPVFMIAFWKARGVGPEGWHILPWWTPQASRPPTKPSAA

>Tb8.NT.115_2 [97 - 282]

MCKLAVKLNVHERARQAVLCLGACVYDCFLESKGCWSRRLAHFTLVDAAGKSPTDKTFGSVK

>Tb8.NT.115_3 [432 - 530]

MGKHGMAQITDQWISTGLPVCAYRAGCPPEKVA

>Tb8.NT.115_4 [467 - 550]

MDFNRIARVRVPRGVPSGKGSIKEMLRY

>Tb8.NT.115_5 [587 - 733]

MRHRAARVQWFRGPDSVGNLNFTTERHLAVTSGATTPYCCGKGEPHKAP

>Tb8.NT.115_6 [737 - 895]

MKNSDAPQIQPQAVQKLTVPRRNAVSLPSVSARVPARHQQNVSLRIAVGRKHK

>Tb8.NT.115_7 [858 - 932]

MCHSVSLWEESTSSRFRLPTGQGKC

>Tb8.NT.115_8 [1075 - 1200]

MAWRSPLLALFETELSGGFLFEGLDKSSFYIEQDGAGHIIIM

>Tb8.NT.115_9 [1366 - 1566]

MTGCIWRRNHCCLYRITPPVRRRWGKRLGSQSLSNHFVSGSEESKANALTHVRQKRNRWPFQLLLQV

>Tb8.NT.115_10 [1434 - 1646]

MGKKTWKSEFIQSFCVGVRRVESKRVDACAAEAESVAVPAASASVATEFLIRLITLLQWSWVTRKGSYSLL

>Tb8.NT.115_11 [1517 - 1666]

MCGRSGIGGRSSCFCKCSYRVFDPPHYFTAVELGDEKGFVFAAIAAATVD

>Tb8.NT.115_12 [1795 - 1869]

MLSAMRERANLSMPRFQVRGKELRS

>Tb8.NT.115_13 [1914 - 2009]

MRVSVILSVGTVLVCSARRPFTRGSACGTGGP

>Tb8.NT.116_1 [147 - 233]

MRAVVMVNITIASTVATMGPQLQEQSASM

>Tb8.NT.116_2 [316 - 417]

MEKKMLRYRHDILGYREQPPLVAILPRHLRFPCV

>Tb8.NT.116_3 [465 - 572]

MPKYSTAVADVHTSGKLTPPPPNKGGGKSEEKKKKR

>Tb8.NT.117_1 [154 - 255]

MIVTWIYSKLNFKSRPSFLWGHTLVGSCIFLRER

>Tb8.NT.118_1 [370 - 477]

MQYHCTLRYGRDERAPSFLISTVFWDEKQNKQVRQW

>Tb8.NT.118_2 [395 - 508]

MDATNALPVSSYPPYFGTKSKISKFGSGDYVNLIFATL

>Tb8.NT.118_3 [521 - 706]

MFSFYIKTSWKDGLNFILANDSGVLMFEGSSCGSAHMFSYVRSHSRSRKPCPFMVIPPPNHE

>Tb8.NT.118_4 [827 - 943]

MICSPLNTSTFVSSPFCFLMSRSQTKKVTSAPPVPSLCT

>Tb8.NT.119_1 [34 - 243]

MCFRCVHAGPCMQTGLYTLWDLVTRITGRCRRGTSCEWRCCSLGLTTLFSVLFLLARLAVRENFRTKCCG

>Tb8.NT.119_2 [63 - 284]

MHANRFVHLVGSGNENNRKMQEGHIMRMEVLFFRVDHTIFSPFLACTVSCSRKFSHQMLRVNSDNRFLQCGDGY

>Tb8.NT.119_3 [318 - 467]

MPNSTCGRLRRLESWKENEGSPSCSATHHRRGDNLYSGRTAVSHGGTTRV

>Tb8.NT.119_4 [578 - 811]

MSLNWQRLFYEQHASQKPRTTERTVHRRARYSLYPSFTLSRATRKKQNVSPECDAAQRPFRHRNLSVPPPPAVQAIRS

>Tb8.NT.119_5 [606 - 926]

MSNMHHKSRAQPSALFTDVRGTHCTHRLHSHELPVKNKTFHRSVMLPNGHLDTVTCQCPPPPRYKQLGRRTVTGEHTEWIVHICAYHFKHESLERWYHRRNENDQHT

>Tb8.NT.119_6 [952 - 1161]

MSVIISRSAESSPLGAGERSTRKGKKTKRIICNPEMLNTTRSSDEIRVEPLILQIYLQQSTPKSRASKVF

>Tb8.NT.120_1 [2 - 76]

MRKYTNKIDWCRSHPGTTIILECTG

>Tb8.NT.120_2 [96 - 260]

MTLGFTCVSSGSIRFANTVEHEHASDSNDADHAERLIALLLTAFGNCTSTIMCSE

>Tb8.NT.121_1 [123 - 218]

MLQMHVPVEHHCGLFPIASPGLICMHCVCPES

>Tb8.NT.121_2 [218 - 307]

MNAAAQLLENGVWYVLCFVPCRGCMTVDSY

>Tb8.NT.121_3 [482 - 565]

MGCRDNGEEEGLTHALLFVMHESQIICC

>Tb8.NT.121_4 [367 - 585]

MCSNNHKWMFLRHFTPNRMHLLSSLVMSLGLGRWHPSAHGVSRQWGRGRVNARTTVRDAREPNNLLLTKGASV

>Tb8.NT.121_5 [588 - 671]

MGVPHYHYCLAPSYVLALKAVSRICAKY

>Tb8.NT.122_1 [197 - 559]

MGGVKFAVLALLVFALLYVVTRVLLTRHRSNPRNDGRQQHTEPSWDITNGANIPVVQPMESTPPLSEPGVLLAIPLAWHREIPLSSRPSRSDTGNRPVNPLERAPDGPEVVYSHAKYIPRA

>Tb8.NT.122_2 [840 - 1004]

MLYVYPKGVGYVYFNSIFVAVECLFVTLLFCIIIIHFVCANPLQMKDFLEVGEEK

>Tb8.NT.122_3 [905 - 1078]

MFICDIIILYYYHTLCLRKSATDEGLFRGWRGKIIVTMTIMTFNGGVVHVTGWVDCHC

>Tb8.NT.122_4 [1035 - 1136]

MGGWSTSLGGLTATADDLHDTILLMLVCRWSLAA

>Tb8.NT.123_1 [282 - 431]

MYTYIYKPSAEWFKHEPVYWVLHQTYRSLLVAVTFFCSNECRPCRSSLSR

>Tb8.NT.123_2 [397 - 615]

MNADLADLHFQGDSGWVMLAVSSAETRESMQVVLPQSARFKGGKMLETRSRPVRLAVMVFVCCTEAECFSSFH

>Tb8.NT.124_1 [206 - 397]

MVCILLDVGFAQPVKSLSMLGISEVCNASPGVAFALKRLNSFGNEQNKKKTSLSIKVFRRASVG

>Tb8.NT.124_2 [487 - 600]

MHLKGYRVSLNESDRASRYKKIRVKVPPTFFCCCCYCC

>Tb8.NT.124_3 [608 - 709]

MGLLCMLMQRSGESNGCRKESGYLGYGMQGTVGM

>Tb8.NT.124_4 [829 - 981]

MLLKKKVEVLENDDNLNRYGEFVYATQMPGHRAAFGNWGRGKWNLSGRQLF

>Tb8.NT.124_5 [994 - 1125]

MYIIAFVHVVIIVLPLDCLQGIFSPKHEETAAKPSRRLRERMLM

>Tb8.NT.125_1 [79 - 159]

MCLVTLTGMGPGAELFLRTVQTTCSSL

>Tb8.NT.125_2 [169 - 291]

MRFWMMDKTQVLSMCFARIPLPFKVRQDKLVEGKASDMLAH

>Tb8.NT.126_1 [235 - 366]

MCRLNIHALAAFKKMKYENSYELVTLTRCTRKVTKRMNHTRLRL

>Tb8.NT.126_2 [482 - 592]

MVYSLSMGGPFWGKVEHDKLGAVAENVKCTVVHHQMF

>Tb8.NT.126_3 [558 - 635]

MLNARWCIIKCFSFLHNEKMEIVRTR

>Tb8.NT.126_4 [639 - 782]

MVWNIMLFWEECNATNHAQSYLKYHVGGKLDGLMASRSTPNITWLFFE

>Tb8.NT.126_5 [782 - 874]

MKLILIARDKARETINHVHQRGLHILCYGDL

>Tb8.NT.126_6 [886 - 999]

MEANVTKRKLTPRSDGMSTASPIPRCGAYGDKENTEGT

>Tb8.NT.126_7 [1015 - 1116]

MYARNPYTDTLSGKEIKTNFRMLRIVAVPPLIWF

>Tb8.NT.127_1 [179 - 313]

MTALCNPFQLRGTSLGTVVGFSKMYGLLTTEGCLGRAALLNIWSP

>Tb8.NT.127_2 [427 - 504]

MSRVLRHICFEAFVLVVVCIRGILAN

>Tb8.NT.127_3 [781 - 915]

MIRCYLGCRRKILYQSIKVFRHSIFFGNKAKCCGAAATSQAGREK

>Tb8.NT.127_4 [873 - 1073]

MLWCCCDITGGKGKIKIIMYGSVVYDRGFDYRLAAFFFLVSITSCVFTFSQWRWLGSSFRCLGLQRP

>Tb8.NT.129_1 [113 - 253]

MNASSASEAGEISKQRTRAVVQVVEYNMHTAFTLDVASFCGNNVAMN

>Tb8.NT.130_1 [142 - 219]

MIFPKLVVFTRCALANDHSYCCFQTT

>Tb8.NT.130_2 [188 - 265]

MIILIVVFKQPNKKDRKTEVVSWRAV

>Tb8.NT.130_3 [333 - 452]

MLKTFLEGYVELITVLMRLYGGLHLSVFVPTNGGSCAVQN

>Tb8.NT.130_4 [513 - 617]

MRIALRRSSFSKCLKASVSLFTIGVLCHPKSAAYL

>Tb8.NT.130_5 [548 - 622]

MLKSLGIPVYNRRAVPPEVCSVSLN

>Tb8.NT.130_6 [952 - 1071]

MQVPRKAFFFVYGPSRDVTTCDVQPHSVTHLEKGYWRSRW

>Tb8.NT.130_7 [642 - 1112]

MTLSGSSARGGFLIPIHSMVGNHFACRTKSRLCFISLLYSTHSTGVALLVLDCAYLHHTLPGNSGLLLMLCDAGSLPRCFPPPCFDLIASEVMEVVARVSISVHAGATKGVFFCLRTLARCYHVRCATPFCHPSRKRILAFSLVRGRPFGLSHEFSA

>Tb8.NT.130_8 [1141 - 1284]

MQFRRGHPKGIVFDPSHAGRNPNGSWAFEVITRMCTACKLTVGATAID

>Tb8.NT.131_1 [65 - 196]

MDRNALSGVVLKGFTSSNLFLTRVLMNREVAARICEGLRCVLFV

>Tb8.NT.131_2 [235 - 351]

MVSYTFAFCRYINASTLPPLVFLLLSFGLETDAWLIRCV

>Tb8.NT.131_3 [378 - 476]

MMPLNYLVALMQVLPLLYLNVRAPVVRILHCYC

>Tb8.NT.132_1 [218 - 319]

MFKLKQPTETFLFQYVRGEISQISINFSSNSTCR

>Tb8.NT.132_2 [734 - 835]

MLTMAFKACYALCVSDESESNLMIFTFADPLALR

>Tb8.NT.132_3 [1003 - 1254]

MPTGKGTSQGLFPPLRGEAEDTANVSKPQCTPAKKYKKGRVLRSSWEGTHQEGKPLDGRGMTKKETLQIHIKNAFAPSTPSSIL

>Tb8.NT.133_1 [206 - 289]

MRLVRVAINDGMCVCVTAICRNCGRWRI

>Tb8.NT.133_2 [231 - 362]

MMECVCVLRRFVVIVVGGVYEPVGLRQRSVCLSAWPHCGSNSKN

>Tb8.NT.133_3 [289 - 414]

MNLWGCVSGASASARGPIVAATAKINIVTVVTSIYVFMYCAV

>Tb8.NT.134_1 [33 - 185]

MQRRRRHRRYHRIVIDEVAGGSRVRQSFAPHYINTERIYIYIYIYMRQING

>Tb8.NT.134_2 [181 - 270]

MDEYGNYVLLLLLDLRVAEDFKICDYICFL

>Tb8.NT.134_3 [185 - 301]

MSMETMFCCFSSIYESQRILKFVTTFVFFKTVAEDEDGS

>Tb8.NT.135_1 [133 - 219]

MVRPDIIWRRVNCARVIVCGVQTLRVCYV

>Tb8.NT.135_2 [210 - 371]

MLCLTDTSFIYRKKNSMERRRFVTWERHDMMRGGVEKKKKKRYGKTLDVSFRFR

>Tb8.NT.135_3 [337 - 462]

MVKPWTLVFVSAKAKIPSNQKDMRFGNEWDGCALFFFFFCSK

>Tb8.NT.135_4 [416 - 496]

MNGTVVLFFFFFFVVNKISKRESHIKE

>Tb8.NT.135_5 [750 - 836]

MEENCVRYHLLGVEKKLIEPLRFHKINWS

>Tb8.NT.135_6 [849 - 932]

MKIIIVAGQMRHRKKKKKGRIKGRKKNN

>Tb8.NT.135_7 [1030 - 1218]

MMQSICRTLIFMAMIKKKKDRLIFFFKRDGNIIVIFLKKNTFLLIASCLGYILFVFHYFFPIN

>Tb8.NT.136_1 [83 - 178]

MTVATVIMIINVVRCRRKQCDRSFFFPRSPLC

>Tb8.NT.136_2 [343 - 429]

MRDLQGNFVLWCSRYGMDVTQLNGGVGPS

>Tb8.NT.136_3 [467 - 541]

MFVMSRLKQTNKHPNPREVLPLLLL

>Tb8.NT.137_1 [158 - 427]

MNCMLNSTQSNMQPVALCGPRLYTVILQEKKAREQIGVKGTDLKAHFVSVTTINNRVAAPLILVSPCRKREAVSDTVSIEVITRGILGYD

>Tb8.NT.137_2 [436 - 543]

MVLCDFVGMLSCLLVTTWHAKSLTICTVFIVASTQS

>Tb8.NT.139_1 [379 - 468]

MPLYCLKETCCLRLLLSSLLLRRNQSVTIG

>Tb8.NT.139_2 [375 - 494]

MDAVILFERNMLSAPLVEFPAFAEKSKCNHWLEETAFTYV

>Tb8.NT.139_3 [512 - 601]

MASCADVAFLHVNLSLSLSLFYRMLLTSHS

>Tb8.NT.140_1 [100 - 192]

MHHPLFFVFPSVCTAVVLLCLRLKKKTATVL

>Tb8.NT.140_2 [274 - 354]

MVCILVGCDTVWCTRRHCITELFDAGS

>Tb8.NT.140_3 [344 - 424]

MQVVKRNLYRPERIIEGVKIFFFFCSA

>Tb8.NT.140_4 [679 - 819]

MFPASGTYVYIYIYIYGAACVLRLGAYKFNNSVAYIAMCFCVHCCSY

>Tb8.NT.140_5 [725 - 886]

MALPVSFGWEHINSIIVWHTLQCVFVCIAARIEGFRKSPFIYVAESPLGFETSQ

>Tb8.NT.140_6 [849 - 962]

MSRNLRWVSKPLNRGCDVGVANIVELVRLELLVVFRLF

>Tb8.NT.141_1 [31 - 159]

MANTALLEREYALPPMGELCLATASSGGAATVTLLAHSVGESL

>Tb8.NT.141_2 [353 - 442]

MNGLYETLSLTQGKSCCYKNVCYSTYSNTR

>Tb8.NT.141_3 [464 - 580]

MPISQCHHLFHGYKTFTSHIKVSQSISPPPDVTNKKILP

>Tb8.NT.141_4 [411 - 599]

MFATLLTQIHGKISPSCQCQYLSVTTSFTDTKLSLLILRFRSRSHRHQMSPIRKYSPSSIHAP

>Tb8.NT.142_1 [104 - 211]

MSRLLWNLFAKRDQHLFLSTLNQYRTRGMHTTWMEF

>Tb8.NT.142_2 [40 - 339]

MFYDIKDAASYTIDFQRERVINVTAFMESVRQKGPASFSFDFEPVQNPRDAYHVDGILTIEDENPNGVYFDREQKMDLGSLRQLMVSFVLPRNSDFLKAF

>Tb8.NT.142_3 [483 - 596]

MSYPTRTGIVRPLRAEKSTAAQRMRGAFQEIWEMSNGN

>Tb8.NT.142_4 [711 - 920]

MWTHTTWDKQKGSIIRRFYAFADKHGLPAVETTIPLFIVSLQLAKSTTIQYTRTLLSLLAAEHTPARMYL

>Tb8.NT.142_5 [359 - 967]

MNLGALIFPYLYSTTREKTNTTEKVTLEDGNNYYYSYPLSANELPDTDGNSTTLTRRKVDSSSTYERSLSRDLGNVKWKLKTCNKVVVGERRQGYSSFYARFVYCDTGFRRKQSSKKDVDTHNLGQTERVHNPKVLCVCRQTRTTGCRNDNPLVYCKFATGKEHHHTVHKDTTIITSRRAYTSANVFVAGLCRMTGGNTIKQA

>Tb8.NT.142_6 [974 - 1048]

MLRWELDLVCDTLTLERDRVALRLS

>Tb8.NT.142_7 [931 - 1059]

MQNDRGKYHKTGLTHVALGARSRMRHFNTGAGSSSFKTILNHS

>Tb8.NT.142_8 [1253 - 1381]

MEGSEKLIAITTKGMETIFCPFGVTAHSIKRGALAHPELALVA

>Tb8.NT.142_9 [1482 - 1637]

MDRHHQQVGVAHDCADVKPLKRGKTDLPWDMPDNSRQAGIALVTRSKTFSRA

>Tb8.NT.142_10 [1690 - 1953]

MTLPLQEVNVGTLYLERVRRRMKEETRQRFEKVWNLKIATEGKSKERDRESHLRIAEKDAVIIKEAGVISQPSDLPTKGWVIPFSVVE

>Tb8.NT.142_11 [1788 - 2015]

MELEDCHRRKIERKRQGVTFADCGKRRCNHKRSRCYKPTFGSSYKGMGHTFFSCGVEVGRKKTPIYCLVQGKKCQK

>Tb8.NT.142_12 [2120 - 2215]

MNMPQKTFECVSAVAQKSGELVELNQLQLVNK

>Tb8.NT.142_13 [2408 - 2629]

MLMSKEMATRYEFIGFLFEHDKQTVLLNAKTMRRIRESAPLEVATIQQMERVVFCMIYAAGVRGEPLFSYYTFF

>Tb8.NT.142_14 [2633 - 2902]

MMRWRLSRLNMGLVNPSDLVFLPKSVLKLEKQWMSELLKNVPVVPPQAQPTTATLVTDASVQGWGGMSFKGTGEIIIAGGSWETAPHMIS

>Tb8.NT.142_15 [3094 - 3219]

MELRTFRDKSGRWGVSRVTCHGVGLSEGKRGRRDENAFSSST

>Tb8.NT.142_16 [3416 - 3523]

MFFPPCAKFLFRWYSSFHRFVASYRLERLRTPTHNK

>Tb8.NT.142_17 [3375 - 3689]

MYCIKVLFGYCDDVCFSLLVLNFFFAGIPLSIDSWLPTVWNDYGPLHTISKFLIYFLYIFPLYIFFIYLFLISLEFLSLFLVCFFVFLSRICHVVIMSVTEIVVE

>Tb8.NT.143_1 [69 - 152]

MNKMEQQLKINRAIGFEVSALTCTSRCV

>Tb8.NT.143_2 [598 - 735]

MEATETHCFLLFINCYLFVYFIINGTDVKYNDVTKHGEGWEGLAVG

>Tb8.NT.143_3 [747 - 836]

MGKCMRRFICYLKYGNIGFFRSVVTHLGHQ

>Tb8.NT.143_4 [743 - 859]

MNGKMYEAIHMLFEIWQYWLLQVSGYTFGPSVSLCPYFC

>Tb8.NT.143_5 [787 - 873]

MAILASSGQWLHIWAISESMPLFLLNSLP

>Tb8.NT.144_1 [85 - 201]

MMRYDHRCVVFPLFEKLLSSLFFFLRGYTLKFNDMLLCP

>Tb8.NT.144_2 [182 - 262]

MICCCALKKTPQVLLSVFFNKYKNNNI

>Tb8.NT.144_3 [429 - 509]

MVVMPPLLITCGVICCKRAREVVFLNI

>Tb8.NT.145_1 [95 - 184]

MRLRIAPFTTASPTLIVYSSTSATKHAMFI

>Tb8.NT.145_2 [301 - 429]

MKIQKTTEEACISVREMHENVRFLLFPEEQQLALRALLKSSEG

>Tb8.NT.145_3 [461 - 580]

MKEREGGFIMRVRSLIFSFLFFIYRSGRLLREFWPLFFSP

>Tb8.NT.145_4 [439 - 759]

MPVGKNINEGKRRWLYYACTLVNFLFFIFYLSEREVTSGILASFFFPVIAAARIDELSTTYMLFKVSEAEGRGGGGSESKKKKKENSHHCAKSVMLRAEGNDKLLRV

>Tb8.NT.145_5 [849 - 983]

MYSFLCAQCRMSHSYPCLFLFLYLSIYFALFPRFLLAGGKALSCC

>Tb8.NT.145_6 [863 - 1012]

MCAMPYVPQLPLFIFIFIFIHLFCVVSPFPSCWREGSVLLLTMAPSSASS

>Tb8.NT.146_1 [24 - 122]

MSSPTLPRKHIFIVVGFFFVGGRLQGFLFGLCA

>Tb8.NT.146_2 [288 - 386]

MCLCECLSFLRNSCGSVDFFKGTQKSIWGCSKI

>Tb8.NT.146_3 [280 - 573]

MEACVCVSAFHFFVTHAAASTFLRARKKVYGVARKYERHSGRFPLFPSLQPLNPIDQAATYSTHCINKAGVVMIIQHVGIRIALKGRMGREEGDMNIM

>Tb9.NT.1_1 [100 - 207]

MMWNTYYTDCFATMILSHHRTAGHITNAFLDKRKNV

>Tb9.NT.1_2 [179 - 379]

MRSLTKEKTFEVNRQVLHPLTTQAKTETSIGESRCPLKITNARMLAKQTLLCIWWLELFLRKSVSMR

>Tb9.NT.1_3 [487 - 594]

MRVGCQRRLRRCTCRLNSPPKGENVTAIDFVDRDNV

>Tb9.NT.2_1 [267 - 350]

MKNLILSLKLRKLLRRNQSAWKKLKHWF

>Tb9.NT.2_2 [194 - 367]

MDNEAQLLGVEFVDGTRSGNIMWKDEELNLVPEIEEAVEKKPERMEEVEALVLTSQMG

>Tb9.NT.2_3 [638 - 844]

MYLKPTDGPFGMIKGDRRYAALMLDGSFPSISHFFCYAFNESVNLSELSVNWGSALTSSFNEQTPMAER

>Tb9.NT.2_4 [819 - 899]

MSKLPWQSVEHTSLRCLNPVHLREGGD

>Tb9.NT.2_5 [863 - 946]

MSESGASSRGRRLNVRAHPVLRIAAVGQ

>Tb9.NT.2_6 [999 - 1106]

MVESIGVILYVCKHDSFGIFRVLSMLRSLVLSRIQV

>Tb9.NT.2_7 [1595 - 1687]

MTRERPMCNWFSPHVMVVYRLCGIDENNREL

>Tb9.NT.2_8 [1668 - 1778]

MKIIGNCEKKSFKRVFRRNRHHSSESQYMGCYYGGAN

>Tb9.NT.2_9 [1765 - 1860]

MAARTRPVRRRPLHFLPATTDIFSALSLSKHF

>Tb9.NT.3_1 [13 - 237]

MEADANTFNPRMLRSASIGLAQPLDFVSTKGKHVVGNLHGVVFNETTTGITGGVNIAATTGNRTAARGRRRRTGV

>Tb9.NT.3_2 [110 - 241]

MWWEICMALCSMKPQPVSLAASILQQLQEIVPLLEAEGDGQAFE

>Tb9.NT.4_1 [128 - 205]

MFVGSTISQCVMSGTANTKLRNNSSL

>Tb9.NT.4_2 [281 - 406]

MYDRRMLLELSSLELISIPSVLFFVVVLLTRTRNCFRTDSMT

>Tb9.NT.5_1 [51 - 266]

MAAEEIPEEGPLVYALREYIIPFVVFGYMILWLFRNVISPVYSQRIIFTRGPLPDDLPDTSRETDTLDERRR

>Tb9.NT.5_2 [253 - 402]

MREDVDILISVPYDSWIHCTVLIILIFFWGDSSSPPPKKRKNSAGGGAKE

>Tb9.NT.5_3 [415 - 525]

MFRLRLHKLFVVSAVAVTFNVIFNVIVHVLSPLCSPI

>Tb9.NT.5_4 [473 - 553]

MLFLMLLFMFLAPFVLPSDLLLIAKGW

>Tb9.NT.97_1 [349 - 447]

MVAKRRREVIGQLLSFDSVPLSAMMVLCPFSDL

>Tb9.NT.97_2 [595 - 690]

MVADFPPQPRTHHRRLARPTFLRRELKHANRV

>Tb9.NT.97_3 [745 - 939]

MAAAFRPPPHIALPYGVPPISITFGRNVMVSPPIFPATLPPPGKHRCTFTALTFLFPPLPSAHLS

>Tb9.NT.97_4 [1006 - 1104]

MIVPFYRVVVRRMSSLSYQTALWQRHQKPNSSL

>Tb9.NT.97_5 [1180 - 1323]

MLWHPHLFPCLLGTVVLRNRTRCLGAPARNKSDIIFSMLVGCSFLGSS

>Tb9.NT.97_6 [1373 - 1549]

MQRVLLLRFCFMSPTKISTLHVRCAFLQLFLTVISSDIALSFQTTWWKKISCIEITSFF

>Tb9.NT.97_7 [1672 - 1776]

MTASQYSAIPLILYFSMLPKITISAPDELPKLTIC

>Tb9.NT.97_8 [1825 - 1932]

MNPPPFVFCVCFLHISQKLPFSYRQGCPLPTGLSHV

>Tb9.NT.97_9 [1928 - 2041]

MCKISSCSYIEEPPAKVVVSMAQCGGGGRTACCGNVGL

>Tb9.NT.97_10 [2199 - 2324]

MRYASYDATGFVLRCCKEVATSFPSIPVPYLNAIHGVCLYEC

>Tb9.NT.97_11 [2020 - 2328]

MLRECRALTGCLLRYTSSALQRKHPPRDSHISMNFMLRTRVVVLSGRRFSSSRESSSRVPCGMPAMMRLALCCAAARRSRLRFQAYQYHTSMPYMVCACTSAR

>Tb9.NT.97_12 [2377 - 2958]

MAAICSSLKSILRPVSVACVAIPCRHWNVTPRSFGVLSAPILSPSSCNVRGWRSIHPKSVNFVFATLIDMKYSFDHCNTTFRPHCSVWLMTSLSVCLASSVRSSAKNPCCSNGTSARHWLNEFMTIMNMIGPRTVPCGTPRSNVLLLESFSLNLTLPVRLLGNDTHHFTMWGDAFILCISLAILSWSTVSNAFE

>Tb9.NT.97_13 [2931 - 3041]

MVYCIECLRVVDEYSTRTILVRGTAEGSTDVQELFER

>Tb9.NT.97_14 [2864 - 3100]

MIRTTSQCGVMPSFCAFPSQFCHGLLYRMPSSSRRIQHPYDIGAWDGRGQHGRAGVVRALSIRGESLIAAASAGTQQCL

>Tb9.NT.97_15 [3205 - 3498]

MGMITPVFHAGGTTPVRRLSLNSLRTTFSAVLPICCSASLYKQSGPAADPLGSSLIDLRSSAIVMGLNSLAVVFMGAGGCESGCLLARDEYSLMNLSA

>Tb9.NT.97_16 [3750 - 3908]

MWWRCSAATGDGDLRRNADGGAQFLFQGVDVLLSTNFPAVLVNLQTEIPPVSF

>Tb9.NT.97_17 [3514 - 3990]

MAALSTSAAGVVSGRGAYTFLTMCQLQRSDTASLEHSVLHLFFVVRSRICLRVATNFSLRWEGPHPAISSSSFASAGVQCGGVALQPRGTETFVAMRMAELNFCSRVLTFFFLPIFLQYSSTFRRKFLQSAFSHAYMGFRGRGQLSASVSSPTITSKNM

>Tb9.NT.97_18 [4136 - 4333]

MHHCVRVSPLVFGGLARSHANACALASAPMSWAPSVVRSVSRSVAVKVCFGGMCADVTVSAVPRAR

>Tb9.NT.97_19 [4358 - 4450]

MRPTTTIPVSTGRSSLTSIDIAPPLQLRAIT

>Tb9.NT.97_20 [4453 - 4560]

MLVASNAKASRLSRRHPCLAQQTKGNPLINKGFVEC

>Tb9.NT.97_21 [4664 - 4780]

MILLMARAQGSTNKKRCPSKTLLIPVLYDAVAGAQQCSP

>Tb9.NT.97_22 [4967 - 5077]

MLRERLRQQQKMLHECARLTLRRCFSPRFYAHKALLL

>Tb9.NT.6_1 [189 - 272]

MTRSIITIACSHFFIPFFRLSLSSSSFY

>Tb9.NT.7_1 [98 - 253]

MFSLLSFTPPPSSCCGRCPPCYSGWLPVTAVFFYYSDPFHLLTASYSLLPRC

>Tb9.NT.7_2 [82 - 405]

MFSWKDVLSTVIYSTPQFLLRQMSSVLLGLVAGYCSILLLFGPLPFAYGILQLTATVLIAYALAVATRYGVRWGTCLVLALMDGGVWRVSFRRKNNTGSARSGETQLD

>Tb9.NT.7_3 [428 - 574]

MRNELTKSTVQRCNHHMNGQGGVAKRTRKKGNRGRLIFVLLSSKHTKVK

>Tb9.NT.8_1 [59 - 340]

MLHAFIHLKEKVNRIYQWDFILYVRALVWEKECTCRVKGGKVVANTPFKSSFQLDGSGFCVNSCLELTGRAEQTAQLQQALNALTCELPSPSRC

>Tb9.NT.8_2 [337 - 480]

MLIFLKERKTPPSRPAISYAHTSTCEVVGDWGNNGGGLELSKACWLVY

>Tb9.NT.8_3 [465 - 548]

MLACLLTSTVEQSTKGPGVASCRVKLLL

>Tb9.NT.8_4 [392 - 565]

MHIRPHAKLSETGEITGGDWNFPRHVGLFTNFNRGAINKGTWSCLVSCEVIVVSGICQ

>Tb9.NT.9_1 [68 - 163]

MRAVGRRTNFQTLKLLGSERHRSAKPRQPKIT

>Tb9.NT.9_2 [509 - 676]

MRRRILRSAMWQKHRTLRSAFDHRGRRVWQKSGGLSALREDAVRRSVAPLILCRMG

>Tb9.NT.9_3 [751 - 1356]

MEDDPVYAALFTAFPQNNKNSTKVEDGSQNHDTKETESEVEHGMHPSLIQLLRYYHTGSSTPSIPLVKFDEENVKNEARQQTRPSTNEEELEGQQAGPSVLCTNGVEKVTTDVEKTGRSDVCNPESSSPLMHQSCPFSALTYAQRCLLTWEASLLLDTQQPQPARKRQAAASGQTHNAVPLEPSPRRNYYDYWTCQRMPKPS

>Tb9.NT.9_4 [1280 - 1402]

MLCHWSLLRDATITIIGRVNECPNPHEGSVGVETILSLFLC

>Tb9.NT.9_5 [1356 - 1439]

MKAALAWKRYYHCFYVDFLSLHFLTLYL

>Tb9.NT.10_1 [141 - 227]

MKFRTVGNKEEEGAASSGRWIHGNAFLLF

>Tb9.NT.10_2 [205 - 414]

MEMRSYSSDSFLILPPLLPVCRLTSTRLLATPLNYNNETHNTEREYACSRRKELEGEWGWDRHHGIHYFA

>Tb9.NT.10_3 [314 - 430]

MRRTTLRENMRAPVARSWKGSGDGTGIMGYTILHNLVTV

>Tb9.NT.12_1 [77 - 166]

MVNVPHRSVATVCQVGFSVISKLSSHPSNF

>Tb9.NT.12_2 [64 - 186]

MGSTDGECASSLRGHRMPSWILSHLETEFTPKQLLVTSLEG

>Tb9.NT.12_3 [186 - 266]

MRYPLRKKNKLMYKRVHSCMIHQRQRV

>Tb9.NT.12_4 [277 - 384]

MSSLRGTLTFVLFTLDGTWDTVQHAIMPQPVLATKR

>Tb9.NT.12_5 [407 - 505]

MRRRNVTCHHPKRDCMTRVWNFNPMMEEKVAPR

>Tb9.NT.12_6 [463 - 762]

MEFQSDDGRKSCATMSSAGAGCGLQTRRRVGLAAVRFGLCGVFNVVVVLCVYPCYIIFSSFPFFSNLTTPLNDALWVVYSILLFFFRCLFLISLYNTTLL

>Tb9.NT.12_7 [687 - 764]

MGRVFYTAFFFSVSIFNLIVQHHTIK

>Tb9.NT.13_1 [152 - 226]

MALEFSRYFFILSLWLGLSILNSLF

>Tb9.NT.13_2 [340 - 462]

MLNKIVSISIIYVLSYVVLMLNKTSSRREGNKRDKYLNIYI

>Tb9.NT.13_3 [823 - 900]

MTPVDPSFHILSFCLHSHLYFYYCHS

>Tb9.NT.14_1 [19 - 126]

MVIGKVICQSKGKCISAIADKRSSNTSTGNAIKKKR

>Tb9.NT.14_2 [57 - 134]

MYIRHCRQTEQQHQHRQCHQKKKVME

>Tb9.NT.16_1 [35 - 115]

MNTNNATSAPWKLWRQQRLAISDCWCS

>Tb9.NT.16_2 [516 - 602]

MYVCCHMYMTLRITFNLCRTRRYINKTEE

>Tb9.NT.16_3 [602 - 697]

MNWYRIFVADVLHTPLSYMCHIDEITLFSVTH

>Tb9.NT.16_4 [630 - 860]

MFCTHRFLTCAISMKLHYFRLHIDKGRLHTKTVMHYNQDVIECNVKTGCPAHHLIHLPAIMPPLRKKKHQHLQQEKK

>Tb9.NT.16_5 [873 - 1058]

MTTVTVSTCKLRLKEWYSIVLARRPIPTNTHYLIYYSQPATCIFNDSLFITTTVICFPITSF

>Tb9.NT.16_6 [1006 - 1086]

MIVYLSPRQLFASQLLVSNMSDELFSC

>Tb9.NT.17_1 [401 - 520]

MRCTFSIAITIIIIGVVVVVYIQIRIFVNLVIIYIYVVIS

>Tb9.NT.17_2 [543 - 701]

MVRLLQPCLLNGDCRRRACFLVEERLPPPLFKTDLLPFTHSLIGCRTCSFHFG

>Tb9.NT.17_3 [674 - 763]

MQDMLISFRVKNVTSDNLVGFCARGWGGGK

>Tb9.NT.17_4 [574 - 774]

MVIAEGEHVFLLKSDFLPPFLKQTFFHSLTLSLDAGHAHFISGEECNQRQFGRILCARLGRGKIVEP

>Tb9.NT.17_5 [990 - 1064]

MCLFKKKKQKQTNLNISPMYAIVLK

>Tb9.NT.17_6 [1064 - 1222]

MSRYQTSVHLLTISYHCMIVYIYIYIYIYFCTLFLKIAYLFLRLILLRSVFNI

>Tb9.NT.17_7 [1338 - 1532]

MVLSRCLALLFILFQVFIMHMFILYAQLCVVRAYGSWKVYPFFVFIFSVFLLPLFFTFAFSGCVM

>Tb9.NT.17_8 [1421 - 1642]

MCSSCLRFMESLPIFCIYFFRFFVTTFFHFCFFRLCYVNFSNFLFAVAKQKFSCLSACCFCRDGRAEPSRLRFF*

>Tb9.NT.18_1 [42 - 164]

MNMRHGMSAAPTNRTRKHIGRLKKKKRGIYSPENPFELLRP

>Tb9.NT.18_2 [488 - 613]

MITGVTGGESGEDAVELAQLIIVRGVVCKRGVECYLKWEAVD

>Tb9.NT.18_3 [670 - 786]

MSWMLPSVSVTKWGRLTAKGKLGVDRFRGCKHGPIPRVG

>Tb9.NT.18_4 [877 - 984]

MPSGGETFIFDKAERRLSYAEGSVSCHVTSEGGGLQ

>Tb9.NT.18_5 [984 - 1175]

MKIVLERPLRETAYKAKGGVRVRDVQQIRTVPLFFKGGTNTYKRLRPSPYMEKGSVQLVRNMPL

>Tb9.NT.19_1 [34 - 171]

MGPHPLSHSRTVESFLQACYEHSVDKQRVVCKRIYVPRERVLVEIR

>Tb9.NT.19_2 [419 - 562]

MHRDIAGFTNTVIFLGPGSKKRGGSEIRLTIALGGNCFAGCTASQLGS

>Tb9.NT.19_3 [1024 - 1107]

MRELPGWRKYRVQRQLEGRENLMILFIH

>Tb9.NT.19_4 [1020 - 1124]

MNEGASRMEKVSSAAPVGGEGKPHDFVHPLRILVP

>Tb9.NT.19_5 [1131 - 1226]

MLTFFRPSLRSFSYFTAVDVLCPTVCCSRHPY

>Tb9.NT.20_1 [70 - 210]

MLYFTSVSCVTTGSPTPSPLREDRVVKAFSVLVSCSRVIRMCLSRIM

>Tb9.NT.20_2 [561 - 638]

MLRHSPSPLINTVVCTPTVLTSVSAA

>Tb9.NT.20_3 [512 - 661]

MVFGVVLSSHPASLPYHAASFPFATYQHSRVHSYCSHISQCSLVTSFGLL

>Tb9.NT.100_1 [70 - 210]

MLYFTSVSCVTTGSPTPSPLREDRVVKAFSVLVSCSRVIRMCLSRIM

>Tb9.NT.100_2 [561 - 638]

MLRHSPSPLINTVVCTPTVLTSVSAA

>Tb9.NT.100_3 [512 - 661]

MFFGVVLSSHPASLPYHAASFPFATYQHSRVHSYCSHISQCSLVTSFGLL

>Tb9.NT.21_1 [546 - 626]

MPFATIQQPTRNRLHLCLSWSCVGRVS

>Tb9.NT.21_2 [593 - 715]

MPELVVRRSRVLKDGAGLTWASDSHEECRFLWYALVLFILE

>Tb9.NT.21_3 [666 - 755]

MKSVDSCGMRWCYLYWSDVYCVESQHDGFV

>Tb9.NT.21_4 [782 - 874]

MYCFEQVVWLCPLSLIVFTRGSCSSRARTHS

>Tb9.NT.21_5 [877 - 1062]

MHLVLLTHAKGLGLDPCGLYKSQLGFRESVALECERDIFAALQLPYVEPIHRHAYCRVHNLF

>Tb9.NT.21_6 [1034 - 1183]

MHTAAFTTCFESARAFLLFVLHGILLFFNFLTVWFSVACVVTDAHHFLYD

>Tb9.NT.22_1 [75 - 257]

MVVKRRGNGRLGKPPTDTGVSENSTGGTPPPESASQRGGAKQTTVASAMEVLPFVFLWCLD

>Tb9.NT.22_2 [371 - 475]

MLGRCFCPSAGKEGADAFCILCACLLLPTWLVLGE

>Tb9.NT.22_3 [516 - 638]

MCPFLLFCEGAYDSFPPRVSPPLHCLSSVSVTEAEVLSCLL

>Tb9.NT.22_4 [949 - 1050]

MTSALVDLRCDRSGCCRVRNTLCGNWCEGCPRNA

>Tb9.NT.22_5 [1046 - 1141]

MLKLAFWEDGSINLFVLSVNAIFSFLCVKGIR

>Tb9.NT.22_6 [1071 - 1148]

MVVSICLCYLLMPFFLFCASKVSVSV

>Tb9.NT.24_1 [188 - 313]

MTAATRKCMLADPGARVCMCVGCASSIVTNLHISHFLSSREH

>Tb9.NT.28_1 [40 - 129]

MWCAHVTHTYILVCMYLFVLRLPLPHCIDF

>Tb9.NT.29_1 [41 - 127]

MVAMCGFLKRCCRPGDELGARVRWKVKRH

>Tb9.NT.29_2 [398 - 622]

MSQRTAVGTTESWGLVSVCCSIVTAYIQPPGNLLIDDSVRYIWFTPGAARKGTHPFVEMYIHVYILLLFSSLRFD

>Tb9.NT.29_3 [1298 - 1432]

MNRDLHYLGAFFVSLQQHFLRKSVGVYRCLLKPCTWCENFFRHVG

>Tb9.NT.30_1 [70 - 219]

MKQVCALAQEKTKGMMLFYPRCFVVRLIVLQCPTYCPKGGPGTICSIFRG

>Tb9.NT.30_2 [162 - 350]

MPHILPERWPGHNLFNLQRMSCPVLIHSQQYIPSQPLPTSVVFNPMCGLGKRTVVLMYGGFMT

>Tb9.NT.30_3 [357 - 473]

MNHKLCFILVAPTNVRRLVLATLVKIIRVLFPFLSARAL

>Tb9.NT.30_4 [509 - 610]

MPYRLDCSWRMSPLFVIVLLPALLRFRLLGRVLY

>Tb9.NT.30_5 [642 - 800]

MRKEAAMKCALFAQIFWLVLSGVDWKPDTSRLFCQVLFYKNSTYSAPPFGSGV

>Tb9.NT.30_6 [825 - 977]

MTLCFLLFVIVVVIRCTCLCGEEKCHSTFFLLKFVSLLEFGWTCCHVRINV

>Tb9.NT.30_7 [881 - 1000]

MWGREMSFYLFSFEICLFARIWMDLLSRTYQCVIIDIRNK

>Tb9.NT.30_8 [1184 - 1342]

MFIICLRYVAATKILVIMTFLLPSVMTYLEGSNVTFFFKKNQNCLKILRCSRL

>Tb9.NT.31_1 [270 - 377]

MRVEYLLAVAPFISLTFFDLVSGSLPLVTVPPPLSL

>Tb9.NT.35_1 [249 - 410]

MPLTSRMSGGADIDDDKGMSGKRKGGSGGGIIIIIIIIIIWMMRLLVSPLYVES

>Tb9.NT.35_2 [468 - 563]

MLDCLIMQQRLNSFKSHRFFTTSNNYECFYSL

>Tb9.NT.36_1 [121 - 231]

MCADARYSQESMELLIHSSYILVLRRKPKKKIIAATL

>Tb9.NT.36_2 [131 - 256]

MRVILKRVWNYSSTRHTFLCSGANQKKKLLLPLFDLWRGQSH

>Tb9.NT.37_1 [197 - 358]

MQRKKKILRRSDAVMATCVRNMVLLFAPFHLTNIKFNFILGRWGWGVDFDCVQL

>Tb9.NT.37_2 [322 - 399]

MGVGGGFRLRAAVGTSGPECILPVLT

>Tb9.NT.38_1 [56 - 148]

MWKYRNRLAASDQADFHSLIPYGCRPCMVSP

>Tb9.NT.38_2 [120 - 248]

MGVVPAWYPREVYSLRYNTSSPTPPPCPSSHSLAVNDLLPSSI

>Tb9.NT.38_3 [226 - 336]

MIYFLPRSDFLRGVHNAAKETASIYVFAMSSILTACI

>Tb9.NT.38_4 [594 - 668]

MWVGVCQISREVYVLFFLIGCEISY

>Tb9.NT.39_1 [512 - 664]

MLGNLIEGTMNILTYSSDCFSLWHRTEPTHIFKSSTRKVCLVEPMCLTSPY

>Tb9.NT.39_2 [705 - 788]

MPLYHDAFAEVVPLHPCHMPMTRASVRL

>Tb9.NT.39_3 [869 - 1063]

MREAGGVKSLKAAQQSTVPSAKSKPTDGFFKRDAFVVFFFFGGCCRTVLLIAVEVIQSRRTMLTM

>Tb9.NT.39_4 [1123 - 1341]

MRQGPLNPDRSEGGQSVMDVSPKPNRPRWWTSVRRRAQNWGVGVGLRRPRARRATRSLPRCGCGAAAFALWRG

>Tb9.NT.39_5 [1308 - 1412]

MWCSSFCPLERLRGVSEAPLGVTDGVILLNSRERK

>Tb9.NT.39_6 [1453 - 1728]

MRKGKTPPPGAEFASKHCGQTVFWGGKPVPFGLQLSVMKGRGVFHSKELSASDPFGFRLKCSIRRCCYTLLTPNVANAPPQPCGVVQMNMYG

>Tb9.NT.39_7 [2192 - 2275]

MQPHSLPRSDGSGAPGVLTSGFAAPSGL

>Tb9.NT.39_8 [2154 - 2291]

MVVPASASADARLCNPTVSHEATVQARPASLRVASQLRLASECSCP

>Tb9.NT.39_9 [2092 - 2298]

MLLLILKARTSDALSGPNSASWWFLHLPARMLGYATPQSPTKRRFRRARRPYEWLRSSVWPLSARALNI

>Tb9.NT.40_1 [17 - 109]

MQHPLSRGGALNSMFLVTINSLPSPKVSVKG

>Tb9.NT.40_2 [259 - 345]

MNVPAVLPHTDRCKRHKTHVRHSSSLKWG

>Tb9.NT.40_3 [243 - 386]

MECKTDECTCRFAPHGPLQKAQDTCSALQQFEMGVTLRKTCRPQSFFL

>Tb9.NT.40_4 [525 - 734]

MASPDYWSNSGEARPAGVSSEVAMPVITSSVEYNVYGYCWSMELCLLFVWSICWSQLFFAFVCACADHCC

>Tb9.NT.40_5 [625 - 735]

MFMVIVGLWNYVCCLCGVYAGRNFFLLLCVRVRITAV

>Tb9.NT.40_6 [656 - 736]

MSVVCVEYMLVATFFCFCVCVCGSLLL

>Tb9.NT.41_1 [2 - 106]

MVFAGTHGPTHGRFSVRFGCCCIGGGFPRAVRRTS

>Tb9.NT.41_2 [21 - 221]

MGPRMAGFLLGSVAAASVAAFLVQYDVLRRKDITEREIDDMETQAALVKDRFYRVQSRLLECQGGDE

>Tb9.NT.41_3 [136 - 294]

MIWKPKLRWLRIVFTGCRAGYWNVREVMNNVLSIFDGCHKRFNEGKSLFLWRV

>Tb9.NT.41_4 [224 - 310]

MFSPFLMGVINGLTKERVYFYGECEMRRW

>Tb9.NT.41_5 [328 - 411]

MLETEVLASSLALPLVILVGPPFLTCRR

>Tb9.NT.41_6 [402 - 479]

MPTVNSAVRGLPGPLPFRDEYVTAYR

>Tb9.NT.41_7 [483 - 605]

MFHRRIIPPSLMSTAKSIEERKKCGDRNTVPFFKDARFQHK

>Tb9.NT.41_8 [586 - 744]

MPDFNTNDSGATACSAIPKGVRKFFFVVFTVSVAHYEVFKVLQCCFSGIRGCW

>Tb9.NT.41_9 [692 - 802]

MRCLRYCSVALVESEVAGEPVLVLNCHGESFPIVLVD

>Tb9.NT.42_1 [63 - 143]

MYVCVCVWGRGLIHLNHRKGRKLICTS

>Tb9.NT.42_2 [77 - 163]

MCMGKGAHTFKPSERSEVNMHQLKERRQG

>Tb9.NT.42_3 [306 - 395]

MCQRRAPCLELRKRANLLLFVISKRHRQIT

>Tb9.NT.42_4 [441 - 746]

MHANVFCCCLSIRTQMPFPKETNGMRVVVHRIARHCYFVNMIVEDAVTPLFSPPSEVKRTYVRVLESGMTISPWLPAYWNDTNGLASSKRLKFWRCSATRGS

>Tb9.NT.42_5 [750 - 923]

MFSGKKKCLNFHRVLASEKCKHIFSFHSFPLWCCDNEVPSLMGAAHTSASVLKFNNRS

>Tb9.NT.44_1 [31 - 312]

MCGGDAAVERKVIEVERECLDMLYQFSDVQSAPAPTQKPRTATAEYGILIETIEYLETLHSYLARGSQEGKNSRHTVVKSVVRDLRGFKAARNL

>Tb9.NT.45_1 [138 - 350]

MPFDNVTASEVQRMREGLGDANVNETRVINYAIAISASAMQRYVRKRCDVVDTTFCQISNNRNVLACIHRI

>Tb9.NT.45_2 [283 - 441]

MLWILRFVKFLIIATFWHVSTGYSCGYMCYFLLLLLIGMFHVIAVGRPFCFIV

>Tb9.NT.46_1 [41 - 130]

MDATHRCVAEDFLKDIFAALMSRGSQGTVK

>Tb9.NT.47_1 [128 - 295]

MREGLTLPTNGVPILQYQLNEKEVLIEFLQNTNLFGCLGFWGLHLKDLYATINKWK

>Tb9.NT.47_2 [426 - 518]

MNIELHNFCGRGRVLTSVRTRQIFDRAVRRR

>Tb9.NT.48_1 [154 - 237]

MGGEIFQRCVATVCVPGVPAVLINDMRQ

>Tb9.NT.48_2 [264 - 422]

MDWDVKNFDVFVTVQLGTHAFSFCKGLPRPTRDGGNRACGIFNNASRKYSTLY

>Tb9.NT.48_3 [319 - 534]

MPLVFVRVYRDLPVTVGIEHVVYLTMRAGNTAPYTNVIGFVSRFSSYRGLGDEWKEGKCPSLTSGNIGKAYS

>Tb9.NT.48_4 [749 - 835]

MHIGWICVSALEGHCHSLPPKLGVTAGCL

>Tb9.NT.48_5 [852 - 1016]

MVHVIFLGRAGGAMEEKSYIPPLLRYSFANFLWILFVECACNIRLFNAFFILAFA

>Tb9.NT.48_6 [991 - 1110]

MRFSSSLSLEIFFLKRDVTLYVNFFPRDGVAGMRNPQIYP

>Tb9.NT.48_7 [1240 - 1320]

MLYALCVDVMSHTPVQYKASAILDGEQ

>Tb9.NT.48_8 [1310 - 1546]

MENNKESGSNNNNTQPWVAVCPPFATGSCAVERTAVRVCGVGSLKVTLRGTREHHFTFNAICFIIHSFSIFIVTTTGPL

>Tb9.NT.48_9 [1485 - 1622]

MPFVSLYILLVFSLLLRQALFNYFFSVHMTSLNDLLMRRQGRRCEV

>Tb9.NT.48_10 [1659 - 1748]

MPLQETVWMPSPLPSVSCASHFFFFAMTFQ

>Tb9.NT.48_11 [1709 - 1906]

MCFTFFFFCNDFSVTVRNQLCTGINMKICIYFARVGGTKEVNYDRKKIVSVLSSSSNGQEVSVLYS

>Tb9.NT.48_12 [2058 - 2231]

MQKLNRRVLCLNIHVSGTNFHQCTLDVDDLLTFNIFFRVKGPAKLVLRLVLKSERVFV

>Tb9.NT.48_13 [2944 - 3033]

MLVCAVGYVIILFTSSPCTTYVSRIRKYVI

>Tb9.NT.101_1 [2 - 148]

MYTCSVVEWVAVSLEMWHQLQPVPSLDTAFHICSSTAMPRLHNPLSCNR

>Tb9.NT.101_2 [25 - 294]

MGSGLLGNVASVAAGSVIGHGISHMLFDRNAPPAQPTELQQVTQQVGNGACAAHVQTYAKCLEANEGNAAPCKWAWETFTQCQSEQQPTQ

>Tb9.NT.101_3 [185 - 307]

MSKLMQNVWKQMKAMRHHVNGHGKPLHSVKVSSNQLNKLSV

>Tb9.NT.101_4 [477 - 596]

MIMSLLLSTCFLSSWGSFTRHGAFIKTGSSRFEWLYNEAN

>Tb9.NT.101_5 [538 - 612]

MVHLSKLVVRDLSGFITKRIEGLEK

>Tb9.NT.101_6 [686 - 871]

MRGKTYLCGHNHFTELRSTYNSTDTMFSSNQFQPVKYSVLHFFLFLLLSLLLSRIRHFLFHI

>Tb9.NT.49_1 [86 - 220]

MSQSSVVSTHDAASFEPVQHSASMRRVKKCFVAHCIAVPGVEASI

>Tb9.NT.49_2 [117 - 230]

MQRHLSLYNIRLACEGLKSVSSLIVSQSPVSKRVYKWP

>Tb9.NT.49_3 [230 - 355]

MIDSIYIYMCVCMYAYLPLKATQHPIVFAIVCMDPLSIVKPR

>Tb9.NT.49_4 [444 - 605]

MRNVSRMLCEVPTDPFELTLIFYLGTFSPRMDKFFFSTCATTRKSNFTDREMVG

>Tb9.NT.49_5 [557 - 661]

MCHHKKKQLHGPRDGWLTRGGEFFVHQKGGPKGGA

>Tb9.NT.50_1 [54 - 242]

MKRAREELLSPIVASIINSWEASDVSTARDRNWPRAAFLLFCFRLVVLIIWVRNEKLFFAHTS

>Tb9.NT.51_1 [55 - 279]

MCTCDAEDSTMSRPFRLPPLAPQATALRDDSAAGCGGKQNATSLPALESWRGLLPSKGVKRDLPVLPSLGAPVGE

>Tb9.NT.51_2 [304 - 384]

MCVSAEGPGEGEVLQADKALSPLFCER

>Tb9.NT.51_3 [505 - 687]

MLDPCTLVRKLKGFVLLGTSTALSEHKYIYIFIPLRILLYIHAYIYLCFFFRNLVFRIFFF

>Tb9.NT.51_4 [938 - 1057]

MRDSFFFPSSSRCYPIVVFSKKDDFLFAVAVYVFFFAYCV

>Tb9.NT.52_1 [214 - 327]

MSKLLSPAASVRWQIRLRAFTPFVHFRLLDASSLRTPW

>Tb9.NT.52_2 [156 - 413]

MLRRSYSRLGHQLIKEPLGHEQTTFARGQRTMANTLKGFYALCAFSAVGCVFASYSLVTGMNRVYPCVNSKGTLSKDNSPFLWWKY

>Tb9.NT.53_1 [175 - 336]

MKTHMVFETMCSRNLRMLLSSSTCWLKVAFGEACGTLAGHFCFFFPQSHHNDAA

>Tb9.NT.53_2 [326 - 403]

MMRLDYLVAVSKCSKVVTFKTLGVVG

>Tb9.NT.54_1 [176 - 262]

MSCVFRAYIDVLFTRFTIGIVVSTFLIHT

>Tb9.NT.54_2 [181 - 312]

MCFSSIHRCFIHPIYHRHCREYVFNSHLSLSNSAERKSTDQCVS

>Tb9.NT.54_3 [362 - 454]

MLVEIKLFFETCINHNLSRILCFKSITLCTR

>Tb9.NT.54_4 [635 - 763]

MGYSPLVKVIYLISFFVFKTVSRIWDDRARVLRIYSGIPPASS

>Tb9.NT.54_5 [706 - 861]

MGRPCPGASHLLGYSTCIFLTPFNLVFFSPRYVGEIYILWVLNIIWEGLLQR

>Tb9.NT.55_1 [539 - 724]

MCLLLSRKYMGLRDNCRKVGDRYFFYLLKLLVYNSVRMIGQGFCSFHLYHLQHIFKNRPKSL

>Tb9.NT.55_2 [760 - 846]

MLHVDLLWVKVSAWRCLTQPAGSVCTVLR

>Tb9.NT.55_3 [798 - 926]

MALFNPTGWECLYRFEVTLSVVTYFHVAFSINLYNDAGEGIKG

>Tb9.NT.56_1 [13 - 288]

MALFSCFRCGYMYEFAVSNSYCRKLTLRNDHCPRCDQLTLFRFMSVSGMVGNMPFKPIGVPGPSYATLWWRKTREGKEASAPLDAVCKSDRW

>Tb9.NT.56_2 [206 - 352]

MPHYGGVKLGKERKHLLHLMPSARAIVGELFGCLFRTIFLSSRCCRVVM

>Tb9.NT.102_1 [167 - 250]

MDETIMSLCGGIPSVSRVHLLHNSVIRG

>Tb9.NT.102_2 [73 - 303]

MKAVKHRICTSVPLLCRRALAHARKSLTQRSHGRDDYVFMRRYPQRFARTFAAQFSDTRLIQENVTAASIDWSTHGY

>Tb9.NT.102_3 [263 - 340]

MSLLHRLTGARMVIEAKNQGSEDRQK

>Tb9.NT.102_4 [373 - 459]

MCVYVCDLLESASRANAWSVWVFYRGGEG

>Tb9.NT.102_5 [365 - 565]

MYVCACTSVTCWSPPHAPMRGPFGCFTEAGRGDETILNSQWYWLRKCRCYGSGGKVLTCTMMTNFLP

>Tb9.NT.102_6 [484 - 606]

MVLASQMQVLWIGRKSFNVHDDDKFPSIAQRWIEQGYGGFF

>Tb9.NT.102_7 [593 - 769]

MAVFFNFIFDIGSGALRLTPYQSVGDERSCWKQVTRLFSAHNKDGEGEEERAVYPPSFE

>Tb9.NT.102_8 [669 - 803]

MKGLVGSRSQGYFLPTTRMEKGKRNGRYTLHLSNKVIVANMLYSS

>Tb9.NT.102_9 [814 - 921]

MVINASLITVKYLMRIIRSAGVIQTSDHHRNNGSEA

>Tb9.NT.102_10 [932 - 1039]

MKAHTIGSSGKGPMYERSQYIVTKLNIITFCGPCRI

>Tb9.NT.102_11 [2070 - 2219]

MCACTPCETKQTKGAALSVDDEVDVSAGERICRSQRPPDATVRCSPIFTS

>Tb9.NT.102_12 [1942 - 2250]

MCGAFELRRPRLSLAITTCTNLFGRSFRTNKTCAKKKAAWRLRCAHARLVKQSKPKAPHCRLMTRSMFPLESVFVAHSGLPTRPSGVPPFSRLELCFLVWVLQ

>Tb9.NT.102_13 [2563 - 2664]

MGPTRDENSHGMSYPRPYHTEESVNGLGGFDLVA

>Tb9.NT.102_14 [2950 - 3129]

MLNPCAPISASYYASIITATRSGSRTNSRHTPKVEEISWLRVAHLHFGFSVMRQLSYPSR

>Tb9.NT.102_15 [2921 - 3190]

MTIGPFARKVCLIPVLRSVPLIMLPLSQRPEVVRGQIPATPLRSRKSPGYALLIFILDFRLCGNCPIRLAKRRCASQRFKTTVSDPPTIH

>Tb9.NT.102_16 [3216 - 3338]

MKAVAVSPFLHLVSPNVKNLVSTAWCFGINGGFFDVSNRSK

>Tb9.NT.102_17 [3790 - 3978]

MCSTSVVQVPRQPGLTFLPRPLPVFLFCIRVALGSFLSPLSSPCPPLLLLRGARKFFCCWVDK

>Tb9.NT.58_1 [70 - 225]

MCGGNILTKKIRESQAKYTSNADDACGCMNGFPFAACSPKGWIYYQSPQLMI

>Tb9.NT.58_2 [150 - 269]

MYEWVSICGVLTEGLDLLPVTATDDLRCNRHFWKKRSKSA

>Tb9.NT.58_3 [275 - 400]

MRWGMRVRVNFYFKRSVTGVPLFCVLNPGISNFFFFFETWMD

>Tb9.NT.58_4 [421 - 501]

MYLLNEETIASCWAGMCYLLLHVVTSC

>Tb9.NT.58_5 [498 - 584]

MLIIPESGVEMEVFTAFHERFCGIVTGVP

>Tb9.NT.60_1 [314 - 412]

MLGTAKHLLQTHNRSWQCKESVSPSTVVVDKFS

>Tb9.NT.60_2 [653 - 754]

MRKESATKRLQVPVACNWLYLDAVVFVQSEKHGQ

>Tb9.NT.60_3 [645 - 797]

MFICEKNPRPNGYKSQLLVTGCTWMQLCLYNRRSMDNNGRGPYANPCFPCK

>Tb9.NT.61_1 [177 - 332]

MSCVLATHPSDCDERMNLQKKTSLGRRIDGSTSRVEVSATAFLPNVDACSLY

>Tb9.NT.61_2 [811 - 906]

MRRFGAVQAHFSTVYSFFLRPFSAMFRRSGQL

>Tb9.NT.103_1 [105 - 212]

MFLKKTIGVLRASQQHEKVSQLRNVVQYFTYCYVMC

>Tb9.NT.103_2 [151 - 228]

MKRCHNYVMSYNILRIVMLCVSWDKM

>Tb9.NT.103_3 [253 - 348]

MFLMWFRPLGELVLCTFYSDTHSSLVELFFLI

>Tb9.NT.103_4 [401 - 478]

MLFLSLIIFFLLLLLLFVIIIIAANF

>Tb9.NT.104_1 [530 - 610]

MVTRRRLQWNRQQGIRRCLRLPFADII

>Tb9.NT.104_2 [307 - 681]

MIFPSAAEPQPTQPFFLAAPMMYNPLPPTPPQTSPVVPQCVYMWAAVPVPPVVRPLMMPNNNLSTTVLLLGTSGDGYTKEASVEPAAGNSKVSPSSLCRHHLRSRCNRRNCRFSHGEDPNEWVAK

>Tb9.NT.104_3 [755 - 925]

MKALGKEKEKETMQKKKGKMVHASSTYDDVKVSSFFFYFFFSLLHYFHFFCFVLFHL

>Tb9.NT.104_4 [1262 - 1378]

MIYSGGKKKKQQQQQANRISLKGKIYKGKCAVMPQIGKD

>Tb9.NT.104_5 [1926 - 2135]

MYICIYLYIYIYSLYSFISLCLFIDSGVSDVSFSTWFPFFFTFFVTNLFILLHFFSSPLLSFPFPFFVFF

>Tb9.NT.104_6 [2418 - 2531]

MDELWGDFVFFFLLSSRYKVRRKLLFIFDYYENKKKEK

>Tb9.NT.104_7 [2531 - 2683]

MNSATYFIYFLFYTSSASGTYLFFKKKKFHFFFFGYKCLDFVCGFFQLFFF

>Tb9.NT.104_8 [2917 - 2991]

MYAVYKFYTRIARSSFPSFFFCLSA

>Tb9.NT.104_9 [2786 - 3058]

MEEKQKKEKNVIILLKSQLNMMVIAFIATVIFVIMFIFVLVLYLCMLCINSTRVLLGPLFPLFFFVFQHESTIYFCLIYTPSSLFILLFFH

>Tb9.NT.104_10 [3166 - 3288]

MIFKAGGKKKQSPFIYLFIAVSSFLKKKIFFSSPQCVPTFP

>Tb9.NT.104_11 [3270 - 3389]

MRSNFSLVVTLALFCPLHAVSKCSSTYLHTHIYIYIYIYV

>Tb9.NT.104_12 [3335 - 3436]

MLKHIFTHTHIYIYIYICLTKEREKKNMSGKNIA

>Tb9.NT.104_13 [3385 - 3516]

MFDKRKRKEKYVWEKYCMRELELTQTGGNKKKGGKKEGKKKEGW

>Tb9.NT.104_14 [3551 - 3634]

MHHKEEANNNNNNNTNYYNKGEVIKMSE

>Tb9.NT.104_15 [4045 - 4191]

MNAAFSTFRFCFSFKHFASIELFIFDVPYLYFHTKEKPLFLFFLFILSV

>Tb9.NT.104_16 [4049 - 4237]

MLLFLLFVFVSPSNTLLQSNYLFLTFRIYIFTQKRNPSFFFFCLYFRCNNLSCKRNENCKKEK

>Tb9.NT.104_17 [4545 - 4625]

MIQHSLCTIISYSAAITYIDEKTYYYY

>Tb9.NT.104_18 [4603 - 4695]

MRKLITIISIILIDNAAQKGLSLYCLFVFAN

>Tb9.NT.104_19 [4959 - 5054]

MKNVEVTSTTFFFRRFFFLKEKKKEEKTKRMH

>Tb9.NT.104_20 [5237 - 5404]

MRGTMKGINSRKGYSKVKHRRPPPQCGSFSSFSFFFLQKKGKKTLSTSLPSFYLFI

>Tb9.NT.104_21 [5445 - 5534]

MDTVQTSLWFVLFYWNYTSYNLHCGTAFNS

>Tb9.NT.63_1 [329 - 475]

MLKGCVLGHRIVTMLLKWITRHRLRVLLFFIRRFEAAIPSVRLKCVKTF

>Tb9.NT.63_2 [613 - 693]

MDGVANVPLLKNTLALLSVLQNVGPPR

>Tb9.NT.64_1 [31 - 117]

MTGDVRPSLLLIVFASAFITMLNGAVAGV

>Tb9.NT.64_2 [230 - 370]

MWYVAYVCMWKYDELPVFVVGALKGRRIPFPVGVVWTQLVFNNCMKG

>Tb9.NT.64_3 [501 - 620]

MANICQQCAVSLLSWLPLCCRRGCSVASEGFTVLGPTEAV

>Tb9.NT.65_1 [35 - 187]

MHEITPEKNLKKTKRERGWAIHRFRYGIMRYHRRSRFCPDVCVCVCVCVQS

>Tb9.NT.65_2 [111 - 191]

MELCVTIDEAAFVLTCVCVCVCVCRVK

>Tb9.NT.65_3 [194 - 268]

MANVSSYVVAHCAASVFPVEKRRTE

>Tb9.NT.65_4 [201 - 293]

MSAVTWLRIVQRVYFQWRKGGRNKRWKREIV

>Tb9.NT.65_5 [293 - 415]

MNCCSSESATHGSFFPLRLLFVNVNILFIYFLVPSPRLLQN

>Tb9.NT.65_6 [757 - 858]

MLLIFTFSDFNVHIRWVVVPERFYFGECGRASYP

>Tb9.NT.65_7 [788 - 883]

MFISDGLLFLSVFILANVEGLHIHDRIDCFVR

>Tb9.NT.65_8 [1066 - 1245]

MLFKIFEYMLLNTHSYVCCRESHRHAACTRILTPEGRKGRGVCVCVCVGFVSNRYVCTRW

>Tb9.NT.65_9 [1335 - 1472]

MGMAFFFLPHCFEEGTAVTLPVPLQLIHYALFLLRYFRHYFYYYLF

>Tb9.NT.65_10 [1420 - 1518]

MRCFYSAIFVIIFIIICSDVLLKDRGCFSPVTF

>Tb9.NT.67_1 [83 - 268]

MDMMSRLRGVGNLRIIGAVYATRDEGVFFVFFLLETCSLLGRSNICCVAGVMKDKTAKNKTR

>Tb9.NT.69_1 [2972 - 3160]

MEGTGGKVKGLSSLELKALSVPFALRFLLGVECDVVLCVTEVGNRRPFSIRFPSLSNAFKELL

>Tb9.NT.69_2 [3072 - 3161]

MLYYALQRWEIGDPSPLDFRAFPMHSKSCC

>Tb9.NT.70_1 [46 - 171]

MGNVSAVIRSSSMVLLVTFGFIQRQRDSTSAFLSVLMRAHFW

>Tb9.NT.70_2 [352 - 489]

MGFPLILRWTMTFTICFHIFATGSCTNPHLETYPRFPNNLLIMAII

>Tb9.NT.70_3 [348 - 557]

MNGIPPNIAMDNDIHHLFSYFRNRQLHEPTLRNVSPLPEQFVNNGNNLKKTLLYTLPVDFTITFRIVVIA

>Tb9.NT.71_1 [226 - 309]

MGPPFVGILPAGRFHYSGWRAVTDLWSM

>Tb9.NT.71_2 [508 - 621]

MATAYVTQKKKIAQKVTQRDRLQRLPFCELSVTCDTAI

>Tb9.NT.71_3 [722 - 808]

MPEKWRSRAHTAPTKNSPLDTKREDADRA

>Tb9.NT.107_1 [138 - 353]

MVSWVGDGETCVNGPFRPTSGLNEFRPFSSLRFDVPSGLAVPPGDAHISTAPSVSVSFANVETKRGGELSSP

>Tb9.NT.107_2 [316 - 399]

MWRQSVAGNYRHRSEMMANKQQQPNKKK

>Tb9.NT.107_3 [446 - 646]

MRQKNDAQSGNRTRASRVAGENSTTRPTAPRVNTKNLYGIVPGTEISKASLLRLRHGRWRCNPVCVF

>Tb9.NT.107_4 [432 - 737]

MVGSACGRKTMRSPGIEPGPPAWQARILPLDQLRRASTLKICMVSFLEPKSARLRSFVCDMGVGGVTLFAFFEGMEGRVVYNDVPSQSLTFPHHAVLSPPPH

>Tb9.NT.107_5 [676 - 831]

MMSPVRVLHSRIMRSYPHLRIEPREHPLGTSTLRHSCRHAQTHALGKTTSYP

>Tb9.NT.107_6 [803 - 1114]

MHWGRRRVTPEYLTSCEGLMWSFGGGEGERIRNHISSFKACFRKTFFTFYTKRGCIRNIFPLGGGSGTAVVSSRPGPFPTECGRQTQHSGGAEVCPNRTKPLGW

>Tb9.NT.72_1 [21 - 161]

MCFFVVYLAAYTGNRANEVPSPNKQADKGSDRGNTHTVKGDIVCGII

>Tb9.NT.72_2 [204 - 464]

MVVVVVKNRRWGLMSGRGRICESRRRLVRSALSKTHRFSGDWRGAACVHFLCLAVVWPPSGMPCGGSAHEKKQWTPVVIQRNGTLRK

>Tb9.NT.72_3 [188 - 538]

MAMSGDGGGGGEKPPLGLDERTRQNMRIAEKIGSECFEQNTQILRGLAWRGLRAFFVFGGGVAAFGYAMRRQRSRKEAMDASGDPTQRYLEEMRGLGFDVDTVEEELEVSRRSGESA

>Tb9.NT.72_4 [618 - 716]

MQTPRDAVTIDSVAQQLQFPFNTLVRGNSAIRF

>Tb9.NT.73_1 [82 - 234]

MERKKKESYSHLFLVEKRRWWSNTINKTFKYIYIYIGGDAHVHDADDYYYY

>Tb9.NT.73_2 [32 - 256]

MVAVAHLEGKGVRFSIKWNEKKKNPTVTSFWWKNVAGGQTLLIKLSNIYIYILEVMRMFMMQMIIIIIKVGKGEL

>Tb9.NT.73_3 [276 - 383]

MWTFPLGPLKIILIIGCFFFFEIIKVTMCHSLLKYP

>Tb9.NT.74_1 [134 - 220]

MPNRLFIIFFAPFFLKKRKKKKVLQLILH

>Tb9.NT.74_2 [106 - 252]

MKACGRFHRNAEPPIYYIFCSFFFKKKEEKESIATHSPLKDYIIHQGWR

>Tb9.NT.74_3 [246 - 353]

MAVKVIFVKLRRSILCAAASLLVFPDFRCATNGEYV

>Tb9.NT.75_1 [147 - 260]

MRRILRFFLHPITRNPLSHIGDSKFVVPTFFFNFKITV

>Tb9.NT.75_2 [268 - 381]

MQMSVCPSHARSTCATHNVFKICKVHNASDLSHHYVRQ

>Tb9.NT.75_3 [392 - 484]

MISSCFTCTSGTLVVLQVCCFCSVVETEPYS

>Tb9.NT.76_1 [392 - 517]

MWRRSEPAPLPVFTSLSLLYISSVLLLFVCKSVMISHWLGYM

>Tb9.NT.76_2 [522 - 605]

MFLLLLTRVSAGPVVGIVIIVIIVVIAV

>Tb9.NT.77_1 [86 - 178]

MKNCRGTIIFQGSAGGNHKSRDLSHTLGCQT

>Tb9.NT.78_1 [368 - 475]

MNIEARIIVGGETIKIFLFYMIPTSSVCARDPIKTF

>Tb9.NT.78_2 [492 - 587]

MSVFSCFIEVICSFLEQSIACITSAAKLEIVN

>Tb9.NT.79_1 [229 - 309]

MEAANNSKETIVTTSRVLHATIRMGNK

>Tb9.NT.79_2 [284 - 376]

MLRSGWETSEMGHLHLHKRKDVPIACYYIQC

>Tb9.NT.79_3 [435 - 512]

MCWASLCYVRYSCLCIINLNSMRGGG

>Tb9.NT.79_4 [457 - 528]

MCVIPACVLSTSIVCVVVVNRLIT*

>Tb9.NT.80_1 [11 - 100]

MYINIRYPMTYLPVQCEVKRGGKLICQLLL

>Tb9.NT.80_2 [152 - 247]

MRVLNCMEVNLLTIRKVLEVPLTLTLTPPQRR

>Tb9.NT.80_3 [85 - 327]

MPTFVVTIISILCIVKGRVLLVNASPQLYGSKFTDHKEGTGSTPNPNPNPPTEAVTVGLGGRFKNSLFATLLLSVIWKQEL

>Tb9.NT.80_4 [416 - 502]

MLRVWETATVRDSFTCNSASLYSLCAIRN

>Tb9.NT.80_5 [312 - 524]

METRIMMVISSHLTMSKGLAIPFVGPRKKKMFIYTCYGCGKLQQCEIASLVTVRHFIPYVQSETRESPRSH

>Tb9.NT.80_6 [610 - 702]

MESHERQSTREILVKSSCPRRGICDRMQLRE

>Tb9.NT.80_7 [1247 - 1327]

MKSAMGKVAYVISLLYNAYISLVVTLY

>Tb9.NT.80_8 [1186 - 1416]

MKPHINMVKTWLRTLLSRRRDEKRNGKGCLCNFPIIQRIHLISCDIVLIMVFRYCREYHQALFIFSLYLFLLQIFES

>Tb9.NT.80_9 [1469 - 1552]

MFVEFRAELLENSRKHIKDGCICFYFQL

>Tb9.NT.80_10 [1649 - 1726]

MSNTALGRVRELSGVYFCAERGNKTD

>Tb9.NT.81_1 [351 - 449]

MKDTSAILLIEKQKLFIMPLIGLTQQVSHLKEQ

>Tb9.NT.81_2 [828 - 932]

MNRPYLRFNGTKHNDILMWTEDWREWNVSRRGLCT

>Tb9.NT.81_3 [868 - 1002]

MTSSCGQRTGESGMSREEGSVLDRPNGMEEWRPSQAPTLRLGALV

>Tb9.NT.81_4 [957 - 1253]

MEAEPGPYIAIGSSGIGKLLSIGSLLLHTLATLATSTWCQSPVPRIIGGSREPAAFFSWGTLLCSVYGIIQHRDQQRLAGKPFFICRSLGTCHQQYHPH

>Tb9.NT.81_5 [1130 - 1276]

MGNIAVLRLWHHTAQGSAASCWETVFHLSVPGHVPSAVSPALTYCSAVM

>Tb9.NT.81_6 [1156 - 1314]

MASYSTGISSVLLGNRFSFVGPWARAISSITRTNILLCGNVEEICGPPLCVTG

>Tb9.NT.81_7 [1302 - 1394]

MRDGVKLRWVLTGKNFVHWISLYGYLFVSFS

>Tb9.NT.81_8 [1737 - 1829]

MIVAEFPLTYHFVERVPQEGSFFLITRIQLK

>Tb9.NT.81_9 [1709 - 1852]

MDRQLPSGKNDCGGIPADLPLCRKSAPRRKFFPYHAHSTEVTVSVDWV

>Tb9.NT.81_10 [1877 - 1951]

MAVRNGSFPGKTLDKIFYQTQTRLR

>Tb9.NT.81_11 [1986 - 2129]

MNTNTNPPPCPNGRHEGGAISDVHIEPSSCLPFGAVGGDGSSTVARRI

>Tb9.NT.81_12 [2012 - 2137]

MPEWQARRRCDFRRTHRTVFVPTIRRGGWRREFDRCETHLDV

>Tb9.NT.81_13 [2133 - 2258]

MCRSCCLFQWQSYQRAWLSLKQVFSPPFRGSNRLVWRGRWGK

>Tb9.NT.81_14 [2020 - 2316]

MAGTKAVRFPTYTSNRLRAYHSARWVETGVRPLRDAFRCVEAVAFSSGRVTRGHGFRLSKYSVRLLGVPIAWYGAVAGVSEDSMGTEVSSSSFAPSSPT

>Tb9.NT.81_15 [2322 - 2504]

MVLGTSLRLGRHKSGVMRSMEERHADSFGVRQWVSSIQTPLVRKAKGVATATLSWRLCCQH

>Tb9.NT.81_16 [2544 - 2627]

MTKKKCAYPKRAERLFFPTPWSDASRLP

>Tb9.NT.81_17 [2492 - 2668]

MLSTLTTGTLKCEAATPDDEEEMRIPQTCGTPLLPYTVVGCESLALGAFADFFATYHLR

>Tb9.NT.81_18 [2611 - 2787]

MRVACLRGLRRLFCDISSALRNSGARADERSASPSTETVSLSAGFTLAGILKEGSYVPG

>Tb9.NT.81_19 [2825 - 2962]

MLYCVEVPPFCNRCIAPLHHPFVVRVRVKTCLAMTTSTQRLSGEGL

>Tb9.NT.81_20 [3188 - 3265]

MPRLLPWNWEYKRAFICGGKQVLSSE

>Tb9.NT.81_21 [3486 - 3647]

MKKGCECTVVPRSATAIYEPRGRPGDSSSISQLFGAPLTPAKRQLKLRCLHNRA

>Tb9.NT.82_1 [293 - 391]

MHMLDKGAVRIAVGHSACSYNSGDSTILLRGPN

>Tb9.NT.82_2 [500 - 631]

MGGQPSSTGGKGGGRCRFISSRVGEYPCAPLLSTVKGTAKRKEW

>Tb9.NT.82_3 [705 - 839]

MSRHSSTMPGGSDTEARQLRFLFGCFSSDINIDSCVLQYPESLGQ

>Tb9.NT.83_1 [60 - 185]

MKGNSESVIIKSVVDKVAYAGRAYSVISTLISSAMDSVATAY

>Tb9.NT.83_2 [326 - 445]

MSVSASFAHTVAPKSAPRSVFHSECINALPPAQLQAVRRR

>Tb9.NT.83_3 [667 - 810]

MVLCVPENRKMHNGVSERRNYSPWNMVSLAESPKRFSSRNRCCLMIPP

>Tb9.NT.83_4 [965 - 1069]

MTHIFLLPSCATHGSAFLSVEAYIPLVTSHKKNLK

>Tb9.NT.83_5 [1233 - 1334]

MTFLRNGAHYVPSSFISNYVSLPITLSQGESHQQ

>Tb9.NT.84_1 [55 - 159]

MVRTARCVSPNSKLSRFRKVVTRNINRQGRLKKLF

>Tb9.NT.84_2 [410 - 568]

MVRRPVHFVSPSSCTCAPFPGSAPMRLSLDLPQLLSADLGRCFRRSRALDTSP

>Tb9.NT.84_3 [571 - 762]

MSVTAYVSNPAMRMSGLLLLTSFRRGSVAHWNPQRRRPFANTLLMMSCRICLGLLCTLVHVWMW

>Tb9.NT.84_4 [1075 - 1269]

MLTGSAAIFRALSVMGCPRICSITWVVLSSNQEATRCVCCISWFRNRNVILGIADIARILHRVLL

>Tb9.NT.84_5 [1134 - 1277]

MLHYLGGPLIEPGSHPVCVLHIVVPQQECDPWHCRHRQNPPSCPVVEG

>Tb9.NT.84_6 [1787 - 1885]

MCVCGWHCTRSDHPAWPHCPVATLSNLSQPFAL

>Tb9.NT.85_1 [52 - 165]

MHSSWLEGIPNGRSKLKVSQICVTCLRRGHRRMNCVGF

>Tb9.NT.85_2 [123 - 356]

MSTSWAQTHELCGLLKPQSGIFPKKAHGRWRQTHRQMSEFCERMSCELSSQVDDSNNDKKHCTVMRETVRVNVVFFLF

>Tb9.NT.85_3 [645 - 788]

MQIALHKGKLHNFDSFADRSKVVQPSAPRSLYHSNTNNGNTQHKRESQ

>Tb9.NT.85_4 [1028 - 1276]

MKQAKCGNLPELGCMVLTHVTRMHSEATVRALGSVLWLRSKTEGENRKPPASSLRQDTGKVSLAAVLPWGMCTVTQKTALAQS

>Tb9.NT.85_5 [1231 - 1443]

MGDVYRYAENSTCTELERVGRQPLSHTIECSARGTRRKHWVANLSERALNIWVGLVKFLTALSHPDDSVSL

>Tb9.NT.85_6 [1701 - 1814]

MSIFSPFAVSFFYRYAKGVSLMFFCTAGAFSTHVTPPH

>Tb9.NT.85_7 [1687 - 1827]

MPRSSCQSFLHLLYRFFTVMQRVFPLCSFVQRVPSLPTSLLLTNTDS

>Tb9.NT.86_1 [112 - 342]

MLFLMPGNGNPSAGPLRKTLNAALPVARHTPIKKMYYEGTSARKHLADTSFPRKGVFGKHLSYRRAFPLIAMFWMRR

>Tb9.NT.86_2 [820 - 918]

MNRFYRPSSCDKNVPSPHARFVFFFATVWKTRW

>Tb9.NT.86_3 [857 - 934]

MSLLPMHALFFFLLRYGKHVGDIEGG

>Tb9.NT.86_4 [938 - 1018]

MLGKLYCGARAHEIMQRRDILRTYKHF

>Tb9.NT.87_1 [288 - 449]

MLAGKIEQRHCRTVTIADSVDASHHVKHRHILERLLSTVGRETTFCAAPTFFLY

>Tb9.NT.87_2 [498 - 626]

MSPRTFEEESGVLFTRRALTAVRHLSMAPIWGMSPISTLLKKI

>Tb9.NT.88_1 [130 - 222]

MSTATYDRARMCVCVSRCTIFVVCCSQDEDC

>Tb9.NT.88_2 [146 - 274]

MIERGCVCVFLVAPFSLCVAAKMRTVEPVSRNACGVYDKEGHT

>Tb9.NT.88_3 [255 - 371]

MIKRAIHDELKQSVGRMERWFTARLLVTAHWSYMHMHAC

>Tb9.NT.88_4 [364 - 468]

MHVNKQVNNTCYSKLMSDIRRWRVAPCNTRFAYYY

>Tb9.NT.89_1 [298 - 498]

MAFPASSNFKRQRERDDGGGEGKIQLTDFNLLTFFKIFSPFFYIYIYICIFPVFKCKFILIQRDVLD

>Tb9.NT.89_2 [592 - 666]

MVSFYDIDIISSSNNTVKLLSGSKC

>Tb9.NT.89_3 [663 - 761]

MLASFAEDSHHHHHHLKRKKKRGPYASHKLSIV

>Tb9.NT.89_4 [859 - 954]

MCVYFLIILNNYRYRSGTEWDELYSSLSTLVI

>Tb9.NT.89_5 [1535 - 1615]

MNIYPSHVLPFTLPNNARSCIALERTK

>Tb9.NT.89_6 [1554 - 1694]

MSYHLRCRIMQDHVSHSKEQNKKKTHKRRHAKKYIYIYIYIENNHNK

>Tb9.NT.89_7 [1591 - 1743]

MYRTRKNKIKKKHTNDDTQKNIYIYIYISRTTTINNALVSKKKGGERKTKF

>Tb9.NT.89_8 [1768 - 1872]

MSGVSDNQFSTPYTVLRLKKKQKKIRNGKHLPIVN

>Tb9.NT.90_1 [266 - 352]

MPEFINIIIIDYMMHRDRVVSHSVLLDMK

>Tb9.NT.90_2 [422 - 541]

MEHFGSILSGKICINAYTYACKYTNAHARNWFSLDAIQSI

>Tb9.NT.90_3 [525 - 626]

MQYKVFSRSLGFTLFCVRVLLLMSLSLLVGCTYI

>Tb9.NT.91_1 [269 - 424]

MTIKSTLAVDRQVQNNEMLHSTDLVAPAGACVMASSTTFLKIFISAFCRAWM

>Tb9.NT.91_2 [447 - 551]

MTPLEDFYVQIKHLLWIYELLRLGHRFVLEDPSLP

>Tb9.NT.91_3 [623 - 841]

MVAPASDVVACVICLEQWSDPVELLPCTHIFCRGCVSTATVCPICRAEVTGLRTANRYLVDASMSLVKIEKVV

>Tb9.NT.91_4 [978 - 1052]

MTHFVGTEDISGNQDGSDTETEKGK

>Tb9.NT.91_5 [1199 - 1279]

MKRGKNTNITNNKATHLNANNRVGGEC

>Tb9.NT.91_6 [1251 - 1409]

MRTIVSAENAEHIRGRAALLAAPVRCLRELKKDFNAIANRACVMEVVEEQNGW

>Tb9.NT.91_7 [1426 - 1503]

MFKQRCSGAFGVAGGADGWGWVSKQK

>Tb9.NT.108_1 [125 - 214]

MGPTSLHLANCSLSRRTFCSLNEMTLTSVG

>Tb9.NT.108_2 [375 - 482]

MLMGNAHCPPQDSSGKNNQVVNISLFLLTNMGNIFE

>Tb9.NT.108_3 [545 - 619]

MPPAPAINAVSANGSAVDLFFSCCC

>Tb9.NT.108_4 [582 - 725]

MAAQWIYFFHVAANVERYVTSHAIMCFNFVMQGRGHPLSENKPSMCYL

>Tb9.NT.108_5 [779 - 907]

MISEDTTLLCLGAQMSNETALKTVVTVIGHITFSVFFSKIKNE

>Tb9.NT.108_6 [932 - 1042]

MHHGNGAPALLVPFFGSTQMCVMCYEQKKPGRSIGHC

>Tb9.NT.108_7 [913 - 1056]

MQKSTEYAPREWGTSSVGSIFRFYSNVCYVLRAKKTGAKYWTLLILSS

>Tb9.NT.108_8 [945 - 1073]

MGHQLCWFHFSVLLKCVLCVTSKKNRGEVLDIVDTVIVRFGAW

>Tb9.NT.108_9 [1128 - 1262]

MVSLYLFMLVRSRDIIIRDSTIIIIISLQFPPQKLKTHVLSFPPH

>Tb9.NT.108_10 [1394 - 1468]

MKSIYSSARGWYFFFFFASHGFVSP

>Tb9.NT.92_1 [18 - 119]

MIHFCCCDSNDNANSAIPKNDSGVCFGKVMKEGM

>Tb9.NT.92_2 [239 - 319]

MSSGSCALFNHSFDLSEHSAQYFTFGL

>Tb9.NT.92_3 [319 - 456]

MICALLHLSLLLCCSLGNLDHSCSSILSHIFFHGTTAAVRWRDLSS

>Tb9.NT.92_4 [416 - 490]

MARRLPSVGVIYPLDVQILIVFLTL

>Tb9.NT.92_5 [493 - 681]

MVVVVLCGHTDRQCIIPIVYDVLHCLFILSVNWTEYLYHQTHPRLTCCSVMRKVYGALQRRCL

>Tb9.NT.92_6 [630 - 710]

MLFGHEKSLWCTTAEVFVIFSVQMFYR

>Tb9.NT.92_7 [656 - 868]

MVHYSGGVCDIFGADVLPVTFGNCCCCQKLFARSARGLICDISHVGRCAATGYVQSDTCIKFDCLFVVCSI

>Tb9.NT.92_8 [949 - 1023]

MNWSIWDYLRVCGSPFLSFTFPSRL

>Tb9.NT.92_9 [945 - 1070]

MYELEYMGLPACVRLSFFILYLSITAITPLLKCSFLQCDGDI

>Tb9.NT.92_10 [1040 - 1147]

MQFSPVRWGYMIARVAIIGILFLLGKRSAFHYHNQM

>Tb9.NT.93_1 [144 - 221]

MEYQVAVAAIFRGKKKKKRHHMVALT

>Tb9.NT.93_2 [344 - 481]

MSGTADALYTRIRWYPHEETLRLGFKKELFTTHLVTNWCVAKSYAQ

>Tb9.NT.93_3 [918 - 1097]

MFYNGVDGGALTAVSIYHRSGCCEHVCMSFVFSTELLCLLDKTLRVRVPLVALENFSSNR

>Tb9.NT.93_4 [1163 - 1270]

MKYPFVSLFLVCKNAVCLRCLLVPPLSLQKKKRRCT

>Tb9.NT.93_5 [1319 - 1411]

MGNWIYLPFLSRGEKQLYARGLSVIISLESG

>Tb9.NT.93_6 [1371 - 1454]

MRGGLVLLFHWKVVDGYLPACGANCFLA

>Tb9.NT.93_7 [1608 - 1742]

MLNWWGYGAGLVYSLPQKGPTRGQQQCLEVRWRLVELRIHGLCAK

>Tb9.NT.93_8 [1495 - 1770]

MTGVHKERVVRVKYGGMRFSGAKTVQKLLLVTTPMNFKCSTGGGTAQDSCIHYPKKVPRGANNSAWRSVGVLWNSEYTDYVPSNLYSRYLCF

>Tb9.NT.94_1 [367 - 477]

MPDNLCMQNKDGALPGLEERLKQIDNWAPGRVICSFT

>Tb9.NT.94_2 [788 - 925]

MRYPRATTTRGLHLTEQCMERNTVTRSVNENARRSRPNTGVFYKCV

>Tb9.NT.94_3 [961 - 1080]

MATLKKIVVEGLHQGYQGVENDVQSAKFKETLAEPNGNNC

>Tb9.NT.94_4 [1022 - 1171]

MMCNLPNLRKLWLNLTEITAEYQIFWGKSQEAGSAVSQRMPQAGEDNTCG

>Tb9.NT.94_5 [1248 - 1370]

MAVCGVRSTEGTYVRGTCISEKELQDMSGNGNGLGLDISSC

>Tb9.NT.94_6 [1727 - 1876]

MCNFPYSFTKCKMSCSRRERLEDLSNMNCEYRYLLERLTKIPEDYTCDRG

>Tb9.NT.95_1 [198 - 320]

MPHLYTHNNIVLYKRITTTLRLWFGRHVLLHVLRVVHILDA

>Tb9.NT.95_2 [869 - 952]

MQKCLKLLLREFMLSCGSIQVDGYFTGL

>Tb9.NT.95_3 [913 - 987]

MWFYSSGWIFYGPLRGDKRKTKRSI

>Tb9.NT.95_4 [1027 - 1107]

MSCILCWVAVWCVLLSSYIAFFFFCLY

>Tb9.NT.95_5 [987 - 1172]

MTFQPAISFGGFMYVLHFVLGRCLVCIIEFVHCFFFFLPILVWTKSVLCSIKLGEAFLCCIA

>Tb9.NT.96_1 [119 - 253]

MLCIYLFEKKKKRRKAALHLKGDACEIEEQLSFEYHIRKLFRLHY

>Tb9.NT.96_2 [186 - 287]

MHVKSKNNCHLNIISANCFAYIISSKCLRNLGGE

>Tb9.NT.96_3 [331 - 429]

MRKTLVGFLYIGKWSGFKVFCMMLSWRLKYFMR

>Tb9.NT.96_4 [445 - 528]

MIQESFRLLISRALPRRTFFRFTLQEVA

>Tb9.NT.96_5 [528 - 614]

MIFATAYFLFIFSSLYKCGLVTWTISVCN

>Tb9.NT.96_6 [823 - 912]

MNWPFAKNKKKDAVGKYSFRRFARVEPCKE

>Tb10.NT.1_1 [39 - 116]

MTTDAPSLLSCGDHPVLWQVGVKAWN

>Tb10.NT.1_2 [629 - 712]

MFTWARAISFSVRCFETLVHFTCANVNR

>Tb10.NT.2_1 [227 - 325]

MFICFRQYNLYYYFWCLSSLVLLTLLHIYIFHT

>Tb10.NT.2_2 [408 - 626]

MIELFMLCLLSKLPASGGLLSLSCHCWMIADCFEVFFLFIVTHFFVGCFETSCYPVTLYTLSLLPGRVLSLSL

>Tb10.NT.3_1 [129 - 350]

MYCAHIWYANFKLLGVTAFGFFPPLYCVIKKKNKISFATTVPQQCGLRYGNGILGPCLYYCVFLKHIFVRVGVW

>Tb10.NT.3_2 [260 - 451]

MWFEVWEWHFRTLPVLLCFLKTHFRTRWCLVVAHVPLTSFKFNSHSMLFIHLSRCKRVTTTVEY

>Tb10.NT.4_1 [19 - 147]

MGRVTFETFNPRSSLHPNKQTHKHTYTKRKQKGAYVLVIQGLH

>Tb10.NT.5_1 [172 - 276]

MFSLFRRLCLLCWASPLALSLSISLSSDIASIFLM

>Tb10.NT.5_2 [93 - 392]

MWACICALCSFQAWRAAFMSVLLYNSYVFPLPSVMPLMLGFPSCSLSLYLSLIGYCVHISNVIRVPVRSCKTTVARFLEHLRTNVSQLIMLKLLAVTDIP

>Tb10.NT.5_3 [569 - 649]

MLRRKSRGSFIGSIRVFMQNRYQPWSI

>Tb10.NT.5_4 [658 - 732]

MFTELGILLHVFFFVNSTLMSLFTA

>Tb10.NT.6_1 [182 - 265]

MVGMGVKSTKGETKWGKVNSHWLHEFLC

>Tb10.NT.6_2 [252 - 392]

MNSCVNQVLKRQLLSLLELGVGMRFSRTNLTVISPAVFCVGNFLTST

>Tb10.NT.7_1 [343 - 471]

MARTGQWSDDHSTKKKNRLPNTSGHTHTYANRTVSLCFYINEA

>Tb10.NT.8_1 [259 - 348]

MRRVVALCVDLCAAFVYLQSCSTARTIRGG

>Tb10.NT.8_2 [358 - 441]

MFVGSRSLDIYLRFYISFLFTSICCAFN

>Tb10.NT.8_3 [444 - 605]

MVLCWPLGVRKLTEARRLLQIKVFPPPLIVAHRMCEVLKTCGTRVGECVDALVY

>Tb10.NT.10_1 [73 - 165]

MKKCTDFQLHVHVYIHIYVCVCVCACFCMHV

>Tb10.NT.10_2 [81 - 227]

MYGLSATCTRIHTYICLCVCVCVFLHACVSRVSCGDAHPATAKQHMRCT

>Tb10.NT.10_3 [227 - 307]

MKMSCILHIPFFFLSFISPSGHFGAPG

>Tb10.NT.10_4 [335 - 451]

MRLNTTAMYFQSVPSLGSYGTPVSFKVISSIKATTTFGY

>Tb10.NT.11_1 [172 - 300]

MHILFSTFSSSPPPLFLFNFTILVFETLLLEYIVGPSFLSLSS

>Tb10.NT.12_1 [96 - 176]

MRCVPNASGGRVSLNSAMQSMLPALGN

>Tb10.NT.12_2 [74 - 265]

MLMCGYTYALRPERQRWKGQPKQRYAVDASRAGELMCSLIQCYHSSCLFFIFWCAGVLCDCPVL

>Tb10.NT.13_1 [49 - 234]

MPTGHVIYDDDGEVFEEDVTVGRGVPPDVIGRMEWPPLVAEPPTEEMKALLRKVLAELAETA

>Tb10.NT.13_2 [150 - 245]

MASVGGRTTDGRNEGFVAQGACRVGGDSVTFG

>Tb10.NT.14_1 [58 - 213]

MEANCHHMNTGISTTVGSCWGAIVSALLDVEDIYVCEAQINTRICGYFMFYS

>Tb10.NT.14_2 [189 - 290]

MWLLHVLQLSTFASRTHRSRIRAISCRASGEFTG

>Tb10.NT.14_3 [331 - 420]

MKTIPLYDRANHGLHALAKWVGNNLHLRGF

>Tb10.NT.180_1 [414 - 785]

MRGYVERPKCGGWGELRLHVPQRICADQALETLVSEAEDASAEPFPAVVLDHHAAPYLRFLRGNSFGPPALCVSHCSEKDLQFVHRLLASRQGPHRAHGKPSIPRREPKKKETFESKGKRSRDS

>Tb10.NT.180_2 [424 - 867]

MSNAPNAEAGGSFGSTCPRGSVLTRRLRRSLVKRRTLLRSLFLQWSSTIMQRRTFASCAAIRLAHRRCVSATARRKTSSSFTACWPPVKARTARMASLRYLAESRRKRRRLNRKGKEAETVDNEQLNGTMRVRHRNTSTDSTTFPQQC

>Tb10.NT.180_3 [791 - 982]

MNNLTAPCAFATGTRPPIPPLSPNSVEAGNKRTATRFMDGRGFHQTRTLTRHTAPLASTRPQSE

>Tb10.NT.180_4 [982 - 1062]

MTVAAGGEVNQKSDPLTVSISLPRHSV

>Tb10.NT.180_5 [1237 - 1467]

MVFVLGATIPVTETCATRQGQALADIRLQGRYIAHNVKGMSLSVPRLERWRGVIFRQLVQQIAKGFFSGRAATHRLR

>Tb10.NT.179_1 [21 - 239]

MLWRFALQGAEAEKCWALLTTQPIWGPALCQLGFTTIASLSVSGACLTIGGSGNGLGAKKDPTDAYFHVRNPI

>Tb10.NT.179_2 [396 - 686]

MPKGMKVVPSETATGYFTFRSFRRAPATRKRKLRFALAYEIYSSVHTRPSRPSRDGPKCEVAKRKVDQVTGKKRVWCHCGQHISSMPGRRVAGSDAA

>Tb10.NT.179_3 [679 - 795]

MPHSAHKKANGNNKGNAIALCLRQPSNENLENAPPNRGA

>Tb10.NT.179_4 [843 - 1001]

MCLRRSVATDYSFPRHSNTTSSVYRPGRKAGKAPIRIRRYPPPLPTPNLPMKK

>Tb10.NT.15_1 [281 - 361]

MLCMIWSIIGYSLNFPHAPTAMELRHT

>Tb10.NT.15_2 [522 - 647]

MTPLGGEIPIALSARAVSRFHSFAPSYPNWMSCAGRRVTTII

>Tb10.NT.15_3 [768 - 890]

MHTKRGDDLTKDYTEHGRVFALCNFTRCVELYLLLEVQQTH

>Tb10.NT.15_4 [814 - 900]

MGGCLHYATSLDVWSFTCYWKYSRRISAR

>Tb10.NT.181_1 [79 - 378]

MLGLKHRCWKMQRSVIPLALHSNRLVGRLLGGSQLFIISRALFCYISGKVNKVCFLALLYYLTVAVKLTFGGTFPPSVADLLVGGAAVLPIFSFFFCFFR

>Tb10.NT.181_2 [389 - 466]

MLFLNEYVSLRSHFQCIDDLDNLKPH

>Tb10.NT.181_3 [546 - 620]

MFKFEYFFCFVVTAIAGVALMIECK

>Tb10.NT.16_1 [226 - 405]

MPFNVRCYETKKLLSRSCTKILGVQLPVQLLLSRYWAPRSRYPRVLCVFAITMGSVAASA

>Tb10.NT.16_2 [410 - 484]

MGHVKTLLKGFMVIERVFARHAVGF

>Tb10.NT.16_3 [471 - 572]

MQLDFSVSSLNWISLPEGAFCILKFSHAYVSLHD

>Tb10.NT.16_4 [550 - 675]

MLMCRYMIKCLPANRKTATAVRRRCTYGSHVCGRQELFPVLC

>Tb10.NT.16_5 [629 - 718]

MGHMFAVAKNYFLYCVNYYVVIWVGMFFAC

>Tb10.NT.16_6 [694 - 780]

MGWYVFCLLIQDHTGCWCGLEIYSGCCFA

>Tb10.NT.17_1 [162 - 266]

MKYAHSCQRFSVTFIMVMLPVWQRACFPFILLSCP

>Tb10.NT.17_2 [272 - 418]

MTLNIVVVFVSHYVGFMGLGLGLCGSYIVGCSGGTAVWNAGVTLVVEHK

>Tb10.NT.17_3 [482 - 604]

MISPVAADWLLGNGLFLFVLIRWVIWVIFSFGTLHYSCVLL

>Tb10.NT.17_4 [619 - 756]

MSMCEMDGSAMESRTRVIRVESHVPVCGEGIILLYVWGRSLRAIVV

>Tb10.NT.19_1 [56 - 139]

MGCLLTDKHTHIYIFFFQIVWLRDICCG

>Tb10.NT.19_2 [45 - 143]

MFSTWVVCLRINIRTYTFFFSKSFGYVIFVVDK

>Tb10.NT.19_3 [222 - 317]

MEININGKGTVVFPLLWRRKAIQRNNNNNVKC

>Tb10.NT.19_4 [334 - 501]

MSYQTFFCIVTMFFVLSVVQTFQVCAFERGIMFNEVGTVNFSMWSHWRTFPNILHF

>Tb10.NT.20_1 [161 - 331]

MTASIKVIPSAYANDNPVTILSCFATASVPPFRPKKNTYTVNLELHHPFSVLLDGAK

>Tb10.NT.20_2 [321 - 500]

MGQSSGSISQRLQFLPPPPPPETAVTGHYNIVLSKFLFVEGDKRCSAAATNRKWRLVYSN

>Tb10.NT.20_3 [551 - 640]

MHATTYSCVGYHKEWNKITGLLEACRAALI

>Tb10.NT.20_4 [711 - 809]

MRTEVKKAVPAEMNLKVGASLNERGNTTKNVGN

>Tb10.NT.20_5 [877 - 1053]

MTSHCDAFRVRNIIMHLTLIEQCGTYNICQFSCCQSPREEPSADSQRTQLHIVMNAILS

>Tb10.NT.21_1 [72 - 167]

MNACTCTQNVNSIMLKRKGEYGREEGKRRRKV

>Tb10.NT.21_2 [133 - 318]

MGGRKARGGEKYKCGNTSRNFFFSFLSSSCTATFRRRKKYCKRCQFLFSCGFTASICVPMRL

>Tb10.NT.21_3 [171 - 341]

MWKHIAKLFFFLFVFLLYCNVPSTQKVLQKMSVFILLWFYRFYLCTDAPLGLLSNRL

>Tb10.NT.21_4 [485 - 604]

MHLPSFNYGVHIHAVEIGGISFIQFSLCSYLNDYPVCVCV

>Tb10.NT.23_1 [130 - 297]

MVDKDENILANATRLCGVTMWRRIGVRARKCRAYSDVTVTLLRLAFHSHDMHYTSV

>Tb10.NT.23_2 [275 - 394]

MTCITPLCSPEMSNLSYPIFFQVVVHVDRSYKVILWVVHH

>Tb10.NT.23_3 [409 - 513]

MRGVTTIIRLLSQVRACVPQKLVLFCCLITNRRYR

>Tb10.NT.23_4 [456 - 587]

MRTPEASVILLLNYQQAVPLECKPCALTDLWGSRFALPLLVIVF

>Tb10.NT.23_5 [518 - 778]

MQTMRTHRPLGVSLCIATAGHCFLTYTHSFSAAVSGNVNSKIRQKREVAKLLYFRLAVFFFKKTFVIMPLSIFFSRGCLCGISPSIY

>Tb10.NT.24_1 [43 - 144]

MEAQCHSSFLQNCATAYRRSNGCAVRGRGPGYGL

>Tb10.NT.24_2 [104 - 325]

MGVLLEEEDPVMGFSRVGKVLAAAMMGIFLFFNGSRGVVPASQRIANELWVGHVLRVWSFFLSPVSRRFFFFLL

>Tb10.NT.24_3 [353 - 439]

MFDEKWWTHCAIPGGKAGGLVGWVWLLGI

>Tb10.NT.24_4 [243 - 446]

MNFGLDMCCVSGLSSYLPFRGDFFFFCFKGQQSLKVICSMRSGGRIVLFLEARQGALWVGFGCWEYRS

>Tb10.NT.24_5 [619 - 705]

MLSQVPCSVFGGNSRCFAHRSLRFCASGS

>Tb10.NT.24_6 [1025 - 1138]

MFHRRFRGRASGDLEVMNNFPLSTCIVFIILRLCARGV

>Tb10.NT.25_1 [626 - 718]

MLSVFGLGLIFTFQRTCFFFSCMCPCRFPFY

>Tb10.NT.25_2 [598 - 798]

MCCGTKLTRDAFSLWIRVNIHVSTHMFFFFVYVPMQVSLLLSYNWQFFRVDALSVRVCFADYVVKAL

>Tb10.NT.26_1 [180 - 269]

MGRGNYTEPAMGAVDPSSSRYTALLPPPPR

>Tb10.NT.26_2 [269 - 490]

MKAPWLCFIHYVHFAFSTPGEVLPSRSPKSAVTLSFLSNSTNKLLLAVLQSCGWAYNITRSLNNLLSVCVGTGP

>Tb10.NT.26_3 [527 - 607]

MISANCCYGVWLLDASGFGHMTVLTWR

>Tb10.NT.26_4 [556 - 636]

MVARCEWFWPHDCFDVAVSFINEVSVT

>Tb10.NT.28_1 [349 - 435]

MCPDGFCPLLDVYGYCIACTQLRLLKQRN

>Tb10.NT.28_2 [402 - 656]

MHSVTPVETTKLGEIAAVLVGFLLFWRQCYSAISFSTHRGYREGVFTFPAIMFRWLRVVFFKARGLYHVPIRGDTNCIVLSVTID

>Tb10.NT.29_1 [36 - 218]

MPWDNAWIAGKSRRKYMMNNTDIRGANRKRDFFTFLYSARCACYVRTCYFFRDCIASLILT

>Tb10.NT.29_2 [155 - 289]

MCMLRSHMLFLQGLHRLSDSNLTFSYFTLSTTIHYYYCIFFFYLE

>Tb10.NT.29_3 [347 - 454]

MLNYFHGLARVKSRLYRTGDVRAVLACFSKMGYSRD

>Tb10.NT.29_4 [574 - 669]

MEYFLYFPVCVFPCVAHIVCMVMRWAVSNECC

>Tb10.NT.30_1 [72 - 200]

MFLRCTATVVTVLRNNKRKGMDYIILPVPLPIFAGVEVAEWVC

>Tb10.NT.31_1 [226 - 402]

MSARKKSLLNFSFSGEALLLDRLISVFYVVRGSRSTYFVIVHGDTLIGMLLYLRVSRDH

>Tb10.NT.31_2 [308 - 511]

MLCAAVEVLTLLSFMVTLSSVCCYIFAYLVTINVSFTSRWMCCNQMKGTGGHVVQTAIIISIFLPLGL

>Tb10.NT.34_1 [63 - 263]

MSMMPVKKKKGNSIRKAKKKGMNAAVVILFLCVCVCRGERGDIRNRHSAMDGMRRSTTAVIDSKMNK

>Tb10.NT.34_2 [275 - 442]

MEGGKKGACVTDVFCPCEQVALSYIIAIRYNIIDSAPKHTLLTNVFSRYFSFFLAL

>Tb10.NT.34_3 [405 - 572]

MSFPVIFLSSSPYNTQCAVCHGLLHWLCIKTPLFFYFYCYCCYSPLRCHGFIIIIM

>Tb10.NT.34_4 [452 - 607]

MCCLPRTFALVMHKNAIVLLFLLLLLLFTTPLSWFYNYNYVMYPTLSRVCWC

>Tb10.NT.34_5 [610 - 780]

MVVKPQKGGLNIYIYIYIFETKRQSTVCFLAANGVYVPSHTTHTRVCTHASLLLFFF

>Tb10.NT.34_6 [707 - 940]

MACMFPHTPHTHACVHMRLFFFFFFNRILILTCYLCLFFYSKIKRIGRKCGGIWLDSTTGSSVYVTYRFLFLFFFISF

>Tb10.NT.34_7 [1150 - 1311]

MLLYRHYYLTPNTHFCHFSLFFFLLDLFFLCLCVCTRFTSFPGPHNVSELVIMK

>Tb10.NT.35_1 [67 - 144]

MDIKRKLNLTSLGCGFHLRGTVEVSR

>Tb10.NT.35_2 [612 - 791]

MSTEDTLRNINKTINLSGGCWYKCPKRHLYVVGECGSPAGEGIGGVGHISFPGNTSVGRF

>Tb10.NT.35_3 [884 - 1060]

MEHNLNGTNDGMGFLWIGVHLLLIMQSISLNEGTALLISTFTFTLLLLFLFLLLLLLVL

>Tb10.NT.35_4 [975 - 1070]

MRAQHSLLVLLLLLCFCFFFFCFCFCLFCECY

>Tb10.NT.36_1 [27 - 104]

MDIKRKLNLTSLGCGFHLRGTVEVSR

>Tb10.NT.36_2 [572 - 751]

MSTEDTLRNINKTINFSGGCWYKCPKRHFYVVGECGSPAGEGIGGVGHISFPGNTSVGRF

>Tb10.NT.36_3 [844 - 1020]

MEHNLNGTNDGMGFLWIGVHLLLIMQSISLNEGTALLISTFTFTLLLLFLFLLLLLLVL

>Tb10.NT.36_4 [935 - 1021]

MRAQHSLLVLLLLLCFCFFFFCFCFCLFC

>Tb10.NT.37_1 [176 - 298]

MPTVVHCCRQACVRRSAVTLQETPLEIGAVRASFCGSSSFA

>Tb10.NT.37_2 [612 - 689]

MGEPRCSVVPRSHVLGRTPFSTRNVS

>Tb10.NT.37_3 [308 - 733]

MRWRFPFLHLRTSQQAALARHVLGYAFSRARWRKLAPTPGNGGSSGTFSFSEVSAFALKPVGKRTAVAPVFALRLGAPTAIGFSDSPALVVNLEPRAVASGDGRTTLFRRAEVPCSWKDSFFDEKRLLAGSVASPQNVGTGT

>Tb10.NT.37_4 [649 - 804]

MFLEGLLFRRETSLSRERRITSERRHGDVTEADGSSVAVLQLKAYVHWGHVA

>Tb10.NT.37_5 [782 - 889]

MSTGGTLLDGCGDTSVVGSDRCETVAGSAPVPCGGG

>Tb10.NT.38_1 [344 - 433]

MLCFSGEWLAVKRIAIRVSYNLDPRNLLFK

>Tb10.NT.38_2 [745 - 969]

MSMMDLWHCKCYKIMVHVRSTAVSRLKCQHMLYNQHFPFPNGIYVSPVSHQEDFAEPTVDSFDTLFFFFSWVSRW

>Tb10.NT.38_3 [1440 - 1649]

MSPTTRLHGSCPVICNFRIMSVVHLRFFPCIVCYVHFIAGWYVLISSWFVYWRGIVCVLVTVDIFCSCIR

>Tb10.NT.38_4 [1815 - 1934]

MHGSYIRRGWGLYSLKDVLEDISDSLVQASYCRQNVVLGA

>Tb10.NT.38_5 [1780 - 1971]

MSTILGVKHQPLCTARIYEEGGDFIRLKMFWRIYLIHSCKPLTVDKTSYWALESRVYFLLFLRR

>Tb10.NT.39_1 [33 - 116]

MYLLLRCFLPLFSFVHVICLPFSYGGGG

>Tb10.NT.39_2 [103 - 216]

MEVVVEGKQCSRERLESVRELNETSAERVEVALDHFFT

>Tb10.NT.39_3 [264 - 338]

MYKDIKYSEETSLRSLLSIYFVFFA

>Tb10.NT.40_1 [73 - 168]

MVQVSPFVCNRRGNATPTLLIIPHPLNVTQHP

>Tb10.NT.40_2 [63 - 254]

MLPNGAGFSICMQPQGKCNPNVAHNSSPSKRNAASLSVRNNNNWARGERHLFCFAFLFSVLCIM

>Tb10.NT.40_3 [324 - 410]

MVLKRIVKCFSSPFFSSAAVLRADPRHLI

>Tb10.NT.40_4 [245 - 535]

MHHVKKELWKAKYLRGLIIRRKVRGIDGIEAHCEMFFFPLFFVSSGVAGRPPPSHIIAVLEGMWWKQKVEGPPFSCFFFHLSLSLFPCGATVASIPL

>Tb10.NT.41_1 [348 - 482]

MLFCFVLLNFFFFFLHPSSGKRVRKLVEGGKFLVKVGGAAPLKRD

>Tb10.NT.41_2 [569 - 676]

MLHMRHSERSVPDHSAATAKSVWKVARWCAGKSFFF

>Tb10.NT.42_1 [361 - 480]

MKITKNMTIGAGCGVCFGTGTHTLGEEYVARIRTARYWMK

>Tb10.NT.42_2 [568 - 642]

MCLTKMAVSRIFLPYHSFVLVIPLF

>Tb10.NT.43_1 [86 - 160]

MGQEHIFIASYVCITFRLSETIILK

>Tb10.NT.43_2 [238 - 330]

MLIYVSIGGERGTKRLPVDQHSSSVASMLLP

>Tb10.NT.46_1 [91 - 270]

MVMFLATAPLGAIELFNQRAGCSIRRHPGEILFVSEKKKIRLVCRHPSVLCLTKPSAGWT

>Tb10.NT.46_2 [633 - 812]

MLTQRAGPDFVLRPLTSAINEKVETLPKLLVKCWMVVMLVVVLCPLVCVAGWTSPPCQLR

>Tb10.NT.47_1 [153 - 248]

MSFTLNNSTVFLIEGRMVLYDGGVSKLPDDDK

>Tb10.NT.47_2 [211 - 306]

MTGEFQSFLMMTSRMSFFFFCVVVCHEGFSTL

>Tb10.NT.47_3 [406 - 498]

MHIQKVFVCRVLFGFSFLFDNVSILVTFLLP

>Tb10.NT.48_1 [83 - 202]

MYVDIRWFRKCSCHLLSNTCSAGMVWSCVRPYLTVIFLGF

>Tb10.NT.48_2 [423 - 551]

MVGFISMYSFIYSIVNILITKANFNEMNVVFFYFLIVLYDTLL

>Tb10.NT.48_3 [551 - 643]

MIFLCETVIRFFVLENSATRLQIFEGCSVSG

>Tb10.NT.49_1 [98 - 253]

MSEATFCRSLTWAEWNECQMNRESSCSTNRGPYWGFAPTRLAALRGVQPRRI

>Tb10.NT.49_2 [278 - 508]

MWAGAARGPGGRMGSQKNAAYRSFVSWRPSAWSTVSVKMGLVDVIPLFSEIYKCICAVDCCQRLRCALTDVCYLKMR

>Tb10.NT.49_3 [762 - 881]

MFRLFFFIFDYKHFTRNNRCLLVYTGPTCLCVYERVCVLS

>Tb10.NT.50_1 [166 - 261]

MCQPTQHFVEPLRKGIEQFGGGLRCLFERCWK

>Tb10.NT.50_2 [261 - 557]

MTVSGACSDGSFFLGGGVGTVQEEGNCGVMFRGYYCGVSVSEGASATHFYFADACTHWYNHPRRNPVAVFSVFLRQKFFSSFASSVAAGFVSGDAVATK

>Tb10.NT.51_1 [253 - 462]

MPDTPTSVGRRPVVVMVSPSHTRLLLGFEGNLATGCALIYVSCEGKFESDRKLTRWFSQGRGASSFVEGK

>Tb10.NT.51_2 [489 - 566]

MHWLGVHSRYRLYFTDATWAALPSLV

>Tb10.NT.51_3 [482 - 631]

MGYALAGSTLSIPFVLYRRYLGGSPIPCVVSFIRGTAEWILVWHAFTYQG

>Tb10.NT.51_4 [595 - 684]

MDISLACFHISGVTTGVSLRFNVVTSPLTL

>Tb10.NT.51_5 [697 - 783]

MRFGERRGTPTGAAVCEKTLFFLITVVSL

>Tb10.NT.51_6 [925 - 1002]

MRVHCSLVVKKKKNCRACWLHRGFVP

>Tb10.NT.51_7 [840 - 1082]

MVKHFLSGWLQIRSSPTTVRWFTKLFYRNAGTLFTCRKKKKKLSCLLVASGICALILFRAVECEHEHGDLCHQNVCIQATC

>Tb10.NT.51_8 [1033 - 1107]

MSTETYATKTYAYRRHVDSPLRNQS

>Tb10.NT.51_9 [1152 - 1244]

MDAVAICIFVQRLQIAVNNKLLFFFHFPGSK

>Tb10.NT.51_10 [1493 - 1594]

MCEGNKSCLIIFCVCVCVFVFVREVHVQKFMCYI

>Tb10.NT.52_1 [60 - 185]

MQLPDRGISFSRKHQQWNCHREICEVLGYATRRSNRMAMQKK

>Tb10.NT.52_2 [312 - 542]

MERERAAFVFTPVVALLFQFLLLSVNLLLCCVLIYSNDVVCVYVCSMRHATIFIIFFKNSPCAILIPPLVMNKMYTR

>Tb10.NT.53_1 [116 - 256]

MVRATHCSIICYRPLITISYKCGAQVLVKQFVLKMLVVECVFVCLWG

>Tb10.NT.53_2 [317 - 496]

MVIPVTKHIQHSSEAEVRCAHDYCGLQAVYVRESPHFFFCAAVSGLATLVDVTRVCVCAW

>Tb10.NT.53_3 [481 - 630]

MCVCVVSTSLWETSMTCICVCCCLGVVGTLFFRRWQPQWCHGVALFNWQQ

>Tb10.NT.53_4 [510 - 689]

MGNIDDLYLRVLLLGGCWDTFFSEVAATVVPRSCIIQLAAVAGTSEPCPYTFSLTLCNRW

>Tb10.NT.55_1 [109 - 198]

MVRLLFFLYVWEGNSLLLSVGGLTRCNFLL

>Tb10.NT.55_2 [206 - 610]

MSRVSCVTMIGQSDGEGHMLLLCNMCAGEVIIPCVCLCVIYLLIGIIYVMLWCVTCSSGCMNKMRLRQNISFLRFLMLLSYTTPQCSQVTNLLMCPHLLFPILTAISRQQHALHVNMQRFVQLCSLWNSASCFRG

>Tb10.NT.56_1 [39 - 161]

MMCTASSGRALFAPRRTPSARGREVLARKLLKFSMRSLQKC

>Tb10.NT.56_2 [251 - 424]

MRKYLCLDGDVMPPRDKVDAEPHKSDATNGSCGGRGGGGRRGGFGFFWEGWSSSYSRR

>Tb10.NT.56_3 [306 - 452]

MRSHTKATPQMAVVVGAEGVGDAVDSVFFGRGGPPPTAEDRNGFPSVEK

>Tb10.NT.57_1 [104 - 223]

MLLFFFFSLYTITMKYQQTKCIRPRGFAGIAHCDDGVCGA

>Tb10.NT.57_2 [204 - 320]

MMACAVPNVSHSYYEVVRVCVWVIIRNLLLESFSLLHGM

>Tb10.NT.57_3 [260 - 379]

MCVGNHPQSFTGIILTVARDVIFHLHSFDLLLWQRMLRRM

>Tb10.NT.58_1 [188 - 385]

MLRNSKKKGNVLHTRIVVACHIRRFTSAHVPSYRIFFTPVALDTVSCEGATLTKQNKAGEKQLTAK

>Tb10.NT.58_2 [401 - 478]

MPRFTTPFQSPLACLCFWMLPQHNKV

>Tb10.NT.58_3 [494 - 610]

MLNRAVFRDTSATSANWLSLSYLPLTCYMRSPCDGESKG

>Tb10.NT.60_1 [57 - 218]

MTVPQGFHTIRSFVSFHPSLFHIFPLPLPLIRSDNSRKGGGRWDGKRGGDGYLR

>Tb10.NT.60_2 [187 - 261]

MGKGEGMDICGSFIAHSCCYTPTDV

>Tb10.NT.60_3 [567 - 707]

MDVGQGVNTRVAFLFGFIHASFCLWFWKKKGGNPFFFFWLCVRPMKL

>Tb10.NT.60_4 [638 - 739]

MVLEKKRGKSVFFFLAVCSADEALEYSFELLSLP

>Tb10.NT.60_5 [770 - 868]

MLPFSLAVGLAVLCVLCLVFGGAWPRRGGFPCS

>Tb10.NT.60_6 [882 - 1004]

MLIYLIAFSLRWRLAVVSLQEGRTVLSLTTFYRRCRHVGVF

>Tb10.NT.60_7 [1239 - 1319]

MHIYVCVCVSVCPVCVSVWGHVPVYVL

>Tb10.NT.60_8 [1190 - 1351]

MYSHTFCLCLYIYIYIYAYICVCVCVCVSCVCVCVGTCTRVCTLTYLYMCCRCI

>Tb10.NT.60_9 [1213 - 1362]

MLIYIYIYICIYMCVCVCLCVLCVCLCGDMYPCMYFNVPVYVLQMHIKSD

>Tb10.NT.61_1 [38 - 142]

MADLGIVWVCSSRRVNLPGSKGMQTMPTTATTWSM

>Tb10.NT.61_2 [203 - 304]

MGRHPRKSVVGECASVLSPRNPLNCVHEENVTIL

>Tb10.NT.62_1 [62 - 139]

MAVVPVSLTVPNCVLVVTLPAYHTLR

>Tb10.NT.62_2 [139 - 246]

MSAAPFPILSLVNLSSLSLVYICIPDLLLLLLVLRG

>Tb10.NT.62_3 [296 - 415]

MLFLLRSVRPPSQLCMLECEVLFSVSTQLVHLMLLPRYFL

>Tb10.NT.62_4 [415 - 528]

MNLFSFFLITSVCLSLCGCRIFGLKSDDLFRGACECCG

>Tb10.NT.62_5 [494 - 583]

MIYSVVRVSVAGNFFLFFFAVLCCPPCNVV

>Tb10.NT.62_6 [626 - 745]

MRWYVSSFFFLVNECCLPLKPRGGHKVLHLLRRYPAYPLN

>Tb10.NT.63_1 [197 - 295]

MIIAVHTNRCWAPYEQFFAVCRTFEEKKLIGAF

>Tb10.NT.63_2 [338 - 481]

MFSFIPQKKKGTTGIIHDYNYGALFYFSFRLFLVCMVFHGKPRPPMFC

>Tb10.NT.63_3 [439 - 606]

MYGISWKTEASNVLLKTFMDFSFLTGNTLVHTCALRGKWYELSVLALTFWGFGATF

>Tb10.NT.63_4 [387 - 623]

MTTTMVLCFIFRFACFLYVWYFMENRGLQCFAENVYGFFFPHRQYARAHMCSAGQMVRTFSPGANFLGVWGHVLVYRCW

>Tb10.NT.63_5 [739 - 813]

MNICFHDLTPFSQLITRMCHHYKPD

>Tb10.NT.63_6 [938 - 1027]

MYRVHPLRLYSILSQVRGSRYMFLSPSERW

>Tb10.NT.63_7 [1039 - 1113]

MIFKHQRDYLLFLFLRLPSWLTLYV

>Tb10.NT.64_1 [75 - 161]

MVFFNFRFPICVNISRFCRVCGHNIPLVC

>Tb10.NT.64_2 [262 - 498]

MRCAEFPEPGHQLLIFFNYLSSTNWFIVRMYRCDCAGIFFLPFCEATGAICVSKEVLHATIVFPPLPFNLYTYNPFIVP

>Tb10.NT.64_3 [723 - 797]

MHIFTSSPFCVFCPRFCVSQKAPKG

>Tb10.NT.64_4 [713 - 808]

MMCHAHIYLFTVLRLLSSVLCVPKSTKRVNGC

>Tb10.NT.64_5 [706 - 822]

MAYDVPCTYLPLHRFASFVLGFVCPKKHQKGERLLSFIP

>Tb10.NT.64_6 [970 - 1089]

MQFPLSLVIVQLCYFRYITKRCIVAVFTCYQAFRCFLTVF

>Tb10.NT.65_1 [157 - 249]

MRKYVLAVRCVCTAACKSCSNQSIPVYQYAA

>Tb10.NT.65_2 [80 - 298]

MLGRRFLLGKVVLNLEGELYNRYSRPCANMFWRLDVCVPLLVNRAVISRSQSTSMQLNTDLDSGPKIPHCLNG

>Tb10.NT.65_3 [380 - 586]

MPHPQSPPCTSVRFFLLACSRYSFRSALSLDFAASLRHGGNFSSISPYPVVRLSRMLSLTGRNALFSCF

>Tb10.NT.67_1 [275 - 391]

MELFLKASQEGPFTLYCSTLAPTRLVFTVFVRRGWLRIL

>Tb10.NT.68_1 [187 - 291]

MPCVEVSFVFVVWWHCLSFLQPSFHFLNGLFSLHF

>Tb10.NT.69_1 [227 - 379]

MAADQKKLRKSKKEKVLRTLSPQCIAIPRSPRRLTVRAAIVQLPGVASLVI

>Tb10.NT.69_2 [465 - 698]

MVWGVTVKGGEECITSQLSAYELSVCVGQTLALKSVCSATKCTLRGGVVYCILFPEIIIVLNRLSVSPWRCVSCVSFA

>Tb10.NT.69_3 [572 - 709]

MFCHQMHTARRSCILYTFSRNNNCAQSSFRIPLEVRVVCVIRLTVG

>Tb10.NT.72_1 [60 - 212]

MILFLSVSLCAKLSRRGYRQKVMPTWVSCVIYMFNWAMCMLRYFTFTQLMK

>Tb10.NT.72_2 [362 - 442]

MHKGCGVQFVAALLLSSATRRCQRKIN

>Tb10.NT.74_1 [211 - 336]

MIDAGKPCCSVVYPGERHSLRKKKASRLHHDAMLAKTLMEVL

>Tb10.NT.74_2 [299 - 382]

MTRCSQRHLWRCFNCGLLALCSVLVAVA

>Tb10.NT.74_3 [428 - 544]

MCLTNNDDNICGSARVHFNLFLYGKSHTPFLPSLPSVLQ

>Tb10.NT.74_4 [444 - 545]

MMITSAAVLACTLIYFCMGRVTHHFYLPCLPCYS

>Tb10.NT.78_1 [4 - 129]

MGQANTGVHLFFVTLFCVAIVCNALVLSFYHVQPERRRIQSY

>Tb10.NT.79_1 [21 - 257]

MLGITPQPCGSNSDSDGDGAGQELLMASLDYELRVKALRNEAYRQALSEVAGEVYESVYTKTFCEAMRKHRTEWGITPL

>Tb10.NT.79_2 [328 - 444]

MAPDTHALRHGDASSILQSMSCFKWTSGVGGSTKGEGIV

>Tb10.NT.80_1 [99 - 182]

MLLAFSTRLCGRRLHSATTQNHHLPWRH

>Tb10.NT.80_2 [222 - 323]

MLRNCGWFVAVSLTAVSKMVGLHQVPFFSANYHG

>Tb10.NT.80_3 [385 - 510]

MWVVHAVGRPILWCHNSSTKGCDAQCDLSGVWSVYHRTILGI

>Tb10.NT.80_4 [459 - 569]

MRFVRSLECISPHYSRNLRGPLCFSRVSTGSSVERFA

>Tb10.NT.80_5 [173 - 598]

MATLNVRCPTCLNGVVYAEELWLVCCCIAHCCEQDGGVAPSTVFFCKLSWVRPLPVRKCSLLPFELSSTRSCGLCTQLDALFCGVIILPLKVVMHNAICQEFGVYITALFSEFERSSVFFSSEHGVFCGEICVSGCVVSVGA

>Tb10.NT.81_1 [94 - 204]

MGQVRGTSIRFSPCSFIPSCSLHQKSIVPALSYHFWI

>Tb10.NT.81_2 [277 - 471]

MMRYCFLSHCCYLILNMYTTCHEARKLFTGGFSHRLLPLRVRCVEREGCCLLCTCERVPRVFPPP

>Tb10.NT.81_3 [933 - 1013]

MRDQLFISFLSTLSLKLVACVSGGICI

>Tb10.NT.81_4 [989 - 1090]

MCERRHMYLNPYILLNGFVTTRTTFQSTCITVFF

>Tb10.NT.183_1 [55 - 276]

MNHNVFLKRIENCRNRCLFDPLIGTCCWGAVFALLDCGWAGLAAPCLFSVIDVGCLLSTLTLSCLTPVPSPKEG

>Tb10.NT.183_2 [718 - 792]

MPCTHDFDLKLSSLPHRAYLRSWVC

>Tb10.NT.184_1 [140 - 232]

MRSTTDRRCAMLSLSVYAGLRASFTPVLQNS

>Tb10.NT.184_2 [232 - 330]

MKRKWFGSPDRGHLRLACLPSLLSVYLLPCSPV

>Tb10.NT.184_3 [645 - 752]

MRRVIMKEKIYIPGPLRELHSFCVCVCVCVNSRLRG

>Tb10.NT.184_4 [1000 - 1146]

MCAGQDILFLFRVHAIAVASFQLFSCAAILYTHHPNAGKGMRRGVAEVL

>Tb10.NT.184_5 [1040 - 1369]

MRLRLPVSNCFPAQPSFILIIQMPEKVCVGAWLKCSSSPALTLAVLGQRGSPYSLYHHEKLSRMCLWRPKCLLICKPGFSIPNSNPPPFRFHFSVILSCGCARSPSRSNS

>Tb10.NT.184_6 [1399 - 1500]

MAARHTICPPVGEARRPRLGAGKRFQNRGQKLCG

>Tb10.NT.184_7 [1598 - 1726]

MLVARVSSVYAQKGCDRPAFAPRPAASVIFPAVDYYRLGKVEP

>Tb10.NT.184_8 [2007 - 2102]

MLTRCVETACALWGKVEKLNGNSHGFSWSSKC

>Tb10.NT.184_9 [2099 - 2281]

MLKEGCTMGGRVWPSSALTLCPVNGHMRHSPFRAAAFTRQPVRKKKLHLSADEKGPPTRGG

>Tb10.NT.82_1 [67 - 147]

MRQAVELTQARCCSVTDNGCMRQLLEC

>Tb10.NT.82_2 [119 - 220]

MVVCASCWSADTHYHWCHTNRLRIVPVNFSCCRF

>Tb10.NT.82_3 [251 - 373]

MVLVVCDRTLQCVLLKRAHASVSHICSVMRTLPGPQKVERS

>Tb10.NT.82_4 [620 - 772]

MHTGYRLLYPHGGVVKGVCVCIRFGVGLGMVLLHSIVCFIPSSLSLGFCSC

>Tb10.NT.83_1 [5 - 103]

MLPLFACASSSLIASTLSVTSCSTYVTEVAVAY

>Tb10.NT.83_2 [78 - 152]

MLLKSPWRINRFSAITSTPCAATVP

>Tb10.NT.83_3 [537 - 632]

MGSFTHAVGAKEGEVKDTFKERPISGFDLRAF

>Tb10.NT.83_4 [461 - 745]

MLTGCGSVLHLYYHSGSRCRLKVLRYGFIHPCSWRKGGGSERHVQGAANFWLRPSCFLIVCHRYHPFDTKSEAVIFCVCVCYGHLHTFPARSGRL

>Tb10.NT.84_1 [146 - 232]

MSPGQKKDKPTISEQRRGIELVPKRHFGT

>Tb10.NT.84_2 [283 - 360]

MRAADKRTARGSQASFCFEVGIPTPS

>Tb10.NT.84_3 [360 - 485]

MIWCCGACLLCLEYMWAGSCVRKGGVGESKMLRCWTTLQLRR

>Tb10.NT.86_1 [114 - 392]

MGTKENATKVVAGNYPYSSVARGEHLVSHRSPPRALKWLAPAALLFAAGGFYFTCRWNNERAKDGPCINCKRQQEIIEEVYFNKQTVQKGNRL

>Tb10.NT.86_2 [491 - 604]

MIVCAVSQCASKRACGDFPGTFLSCAVHFFFFERHFIP

>Tb10.NT.87_1 [119 - 310]

MFSLTQTWLIAHWYCGHKFRHRFMRDKRFHPSLQASHDARNRFSKRRHFKTNRWNYQQAYRDMP

>Tb10.NT.88_1 [192 - 386]

MFVLIFEERHDRGNSVLHVNGNGITLLLLSLLVVIERASIWRSLSHRASLAAANDTPTSHLGAPM

>Tb10.NT.88_2 [352 - 474]

MTLRQAISVLPCKAAPNGVEEQKCPRCGTVTGCVPILSKQG

>Tb10.NT.88_3 [594 - 692]

MQRLRVTTATDSVAGCATATGSSSLSCAVVRAL

>Tb10.NT.88_4 [554 - 760]

MCELICNRRRASSHAAVTRHHGNRLCCWLRYGHGFEQFELRCRKSPIRTSVSGNRKGIVSSVVGNSRES

>Tb10.NT.89_1 [144 - 353]

MSLIFYVNEGCNYVSRENMKCRQMIRLRLKRDTSSTLCCAEFSPPFRGYMMSSSINTLSSMRVCLKSEGM

>Tb10.NT.89_2 [490 - 591]

MWMICFVKPSSSKQRYLVEQYKPPGRCHLEPVTK

>Tb10.NT.90_1 [22 - 222]

MSEAVDSSAVQKARDDYMQHFSDVFSDELLKTYESDGSAAAVEQLTACIEVGVAVWGHPISIRHPLS

>Tb10.NT.90_2 [285 - 383]

MQFDPSVGPSFSFYSFLESFTRSRPRRTPLFSV

>Tb10.NT.90_3 [420 - 527]

MCIHNCPGKLCKYIYIYICASYFCMLNMHAPFHQGA

>Tb10.NT.90_4 [449 - 529]

MQVHIYIYMCIIFLYVKYARSLSSGCQ

>Tb10.NT.92_1 [327 - 401]

MHATQRTHAATNSRLPYWPTKNGIS

>Tb10.NT.92_2 [404 - 517]

MLFFHDWALAEARTPQTHLTVACHKHFLGCSPHGMHKS

>Tb10.NT.92_3 [417 - 551]

MTGLLPRPVHPKPTLRLLVTSTSWGAHHMGCINRKGAHILIFVLS

>Tb10.NT.94_1 [101 - 313]

MRALLLFFAAASLIQTVLVGGASGNSQNTVSISGDTLGGILLGSLVGGILIFAVFMLANIESPEVLGHPDR

>Tb10.NT.94_2 [482 - 598]

MPYYLSSLTLQLSKDSPFSFPLPPLLLNSCERDVMQKKK

>Tb10.NT.96_1 [9 - 182]

MEYRIKVRNEILVSTHNGPTFVGCGSFQPLSVLTSRADVVCGSNSRLSAPQYSNSVGN

>Tb10.NT.96_2 [121 - 255]

MWSAEATLASAHHSTAIRLVIEGIQLVAEKVMTKCFLHCPRRFCF

>Tb10.NT.96_3 [222 - 404]

MFSALSSPVLFLTGLTACAVLLLPFVPISLAGGEVIRWANAWGVNAGTPCRFFFTSLASFY

>Tb10.NT.96_4 [407 - 544]

MEVRLIGVGYTPYGVVLIIIHSCYWRPVVALKKFSCGRRSQQLFRS

>Tb10.NT.100_1 [4 - 111]

MHIHTLHVKERYTALQASSIRLQAKTKSPRVRLTGG

>Tb10.NT.100_2 [181 - 327]

MIYDITTPQYQQFLRSCGRRREDYVKGSSTGFSGNKQTTKPAGAASTGL

>Tb10.NT.100_3 [376 - 546]

MRAVTSVRKGSAGRINKRKRNVFPSTTLVGRLSANKSKAIFLFDLVGGSAKQNDEKK

>Tb10.NT.100_4 [533 - 616]

MMKKNNKRRRNSDTAKRGWGILRKRGEN

>Tb10.NT.100_5 [639 - 716]

MMIFLFLRLASFLSVNLVLPRRCTSF

>Tb10.NT.101_1 [128 - 325]

MRKSHISVSSMLSFISFFRFIISCAFLNKNVLFFWGGGGGNNSVYTKNIRKNRVTCHSDFAPFVSG

>Tb10.NT.104_1 [181 - 291]

MGWVQTMRSGRDEDPSICLFVLFCFVFFGGGRKRIRP

>Tb10.NT.104_2 [310 - 495]

MSLHYFLARAKGVCGGLVDGNETVAVAPAQLSFHHPLHLAGHSCVCRSKGVMHVGVNNTIFW

>Tb10.NT.104_3 [571 - 669]

MKGASQSANCWESVAASLSMVPTTAVLRDGDRS

>Tb10.NT.104_4 [531 - 680]

MQVLVSVLFTRIIYEGSKSVSKLLGIRRCISVNGSHHRRTPRRGPQLIDT

>Tb10.NT.104_5 [941 - 1084]

MLVKLEVVSLSWWQTGRGILGTSRPSLFPHYYLLKLCLNLHKTKKLTS

>Tb10.NT.104_6 [1094 - 1186]

MPHGGERNCVWKEAELATPRGRKGGKIIRGK

>Tb10.NT.104_7 [1622 - 1903]

MEMNFKGLLKTMRHEVNVAMLNIFISLPPRRRKGRIFCIMWKSTLKVTNRQVFVFMQHSFSLFFFIFLSRCNGGDHRSEYVGVHRPKNLAAKQW

>Tb10.NT.104_8 [1860 - 1964]

MLECIAPRIWQQNSGSTSNLVFLTIFTTGIITLVI

>Tb10.NT.105_1 [63 - 254]

MKKGNRRGRYRKRRSSRSPLFWVALAALVLLTLSTGLNVWLAVEYASKKKVETNLELAEKACGL

>Tb10.NT.105_2 [175 - 312]

MYGLLLSTHPRKRWKPIWSWPRRHADYSVAMLGRYQPCSPCGDAHL

>Tb10.NT.105_3 [302 - 427]

MHIYNGLFFLVVVVTLSVHAEEGLLVNFFVCLFLTGCCGKSI

>Tb10.NT.106_1 [160 - 234]

MQGGNTKFPQSNIGGNSVVLVQTPH

>Tb10.NT.106_2 [440 - 625]

MACGGGNAYGFERDARVLGQYKGNTPTRATRTAATRGEAQDKYHTFLFTDGCVDCSPLPARK

>Tb10.NT.106_3 [480 - 662]

MPEFWVNTRGTHQRGQQELLQHVVKRKTSTTPSFSQMGVWIVALSLQESKVHHTNYRCWSS

>Tb10.NT.106_4 [885 - 1013]

MLPPTKITGLEYTRVVNDRVQTKGLGERRLVVLRSRGGKAVKE

>Tb10.NT.106_5 [1067 - 1261]

MTAGNYPIRSKSYWCMLTVPRKEVRRTWFCLTRALFGGSYTTQNDNFEQFWGVRRLDLGRRRRGV

>Tb10.NT.106_6 [1361 - 1438]

MVGTALGSSTRSKRKGEKMPKYLLLH

>Tb10.NT.106_7 [1345 - 1611]

MEVSANGRDGPRKQHQEQKEGGKDAEIFIVTLKMVHFMSYISVVNKTVRINKHNFILIPTERKSKGKGDVFKINFFFHRLLNASPEVLF

>Tb10.NT.107_1 [72 - 200]

MHNLNTIQHHTFGRICAYLHLYIPAAPLAVTPSSESCLRAGNK

>Tb10.NT.107_2 [249 - 389]

MCVVHWRCTVCFSLRTCNLDSKMQRKRRSVLYSRTLATNSFIFVAVG

>Tb10.NT.107_3 [671 - 805]

MCRHRLGFTKICSVLVFARVLQHWCLMGLGVVFFFLRNQTEKKRS

>Tb10.NT.107_4 [860 - 964]

MQGKITPTTLYAAGKFFLRLRWKLWQNGEVKDGGL

>Tb10.NT.107_5 [703 - 975]

MQRVGVCEGTATLVLDGVRCCFFFFAQPDRKKEVVEYERRFECAKSTQLIWHHAGEDHANDALCGWKIFSTSQMEVVAKRGSKRRGIVRGG

>Tb10.NT.107_6 [1219 - 1515]

MKRKIMSLDYETRYFHYPYGKVIKRGAILHFAGSSNVWKETIPRYLSEMSWFKALKNSFTEREAYRRLLETTSVEIHSARGAFRYVKLGTVCPISNVSN

>Tb10.NT.109_1 [16 - 105]

MLRPRHIITSTYPPCRITSALSCCCRVFSR

>Tb10.NT.109_2 [105 - 227]

MNRPLVCKPLLRVQKLSLGARADVWWCCILVCLCRLHQKVP

>Tb10.NT.109_3 [172 - 576]

MFGGAVFWFVYAGCIKKFRERLIVPCLVVKTETCRPYFFELVCVPRYNQSSAQNELQLFIGYTRYSLILSQGGHPDVQGMNCARINRFNSEAANEEGFVDVLVHEGVNRKTSSTISFPRYAAPPKETVSKRKVRG

>Tb10.NT.109_4 [530 - 616]

MLHLQKRQSVREKCGVSGVAGCTMVHIPF

>Tb10.NT.109_5 [670 - 762]

MLLANFPFGLHGDTCYAHRGTVRSLVLSFFE

>Tb10.NT.109_6 [716 - 931]

MPTAARCGHLCCRFLSNGFVVYTEQFAQLVAKSLFFFGGFCGWISLLWLVSWCDQTVCRPSLPFTLVAPWRL

>Tb10.NT.109_7 [1277 - 1414]

MVVGSKRPVVQRRCLRFNTFYKDVAAWICSFPATPTGTKRKVHVVV

>Tb10.NT.110_1 [31 - 108]

MYFHPLHVVPLLAPDVCIGQTLFGSY

>Tb10.NT.110_2 [50 - 139]

MWFRCLLLMSVSVRPYLALIRWEADHKGRG

>Tb10.NT.110_3 [286 - 381]

MMGLFGPHGAHVGAFCKELWCCLGCCVPSPPT

>Tb10.NT.110_4 [357 - 509]

MLRSLTAHVRCLLATDKRRLRCDARGGQVCSFEFENDVTHQTEFVCSATAA

>Tb10.NT.110_5 [466 - 561]

MLPIKPNSFAVPQLLSSIITSLFFVIIVTGLC

>Tb10.NT.110_6 [636 - 776]

MFLPAFCFFFDRDLSRDQTRFASFSLEVTIAADFFLICSVYSILWNE

>Tb10.NT.111_1 [154 - 279]

MLTQTHAIKTTKNKFCGTERSTHTPLPFWDPQEGCSWCVVAA

>Tb10.NT.111_2 [170 - 295]

MPSKRRRINSAVQSAAPTLPYRSGIRKRVVVGVLWLLSSRFA

>Tb10.NT.111_3 [457 - 585]

MPEMFSGVLSWHLLLFLPVGGGCCAKEEKLDGISVVCTHHLQS

>Tb10.NT.112_1 [16 - 96]

MTKLLRHMRRYNCILLGCGMKTPPKCQ

>Tb10.NT.112_2 [96 - 197]

MKERERKFFIPVFRWGGALHTKSCCVNTSEQQFL

>Tb10.NT.113_1 [32 - 106]

MLRCVLGLSQLSEPNKEVMAPPNIW

>Tb10.NT.114_1 [319 - 417]

MFYRQTSSRYCLPVFGYDTASTVRVYVCVSAAE

>Tb10.NT.114_2 [161 - 562]

MYKLELLCTWIGSWFAGDTPSRSTERQGKENMQITKPLREHQEEWVGGVISSICFIVRHPLGTVCRYSDMIPQVLCVYMCVCRPQNSGGMRKRGVINTFMRLLLYGNLPSFTLSNHHIHVDEYVPLLLLFLASL

>Tb10.NT.115_1 [184 - 288]

MEAVLLITILLKGHHLNKCLYYFEGVDWIFGLHVE

>Tb10.NT.115_2 [429 - 545]

MPSSRILQSRSPFLVYHGEYFGRSERVYIVADVVLKGVK

>Tb10.NT.116_1 [77 - 304]

MRHSALDPTAGNTSPPQQLMAMGRVPTTAYPMVLEGNKTVSRMISCCRLSFKFQLQKGALHLPFPANTNAVAVAEA

>Tb10.NT.117_1 [48 - 152]

MTQAICPSIFPPSQLPFDTVELQHGKQRLKEAHSW

>Tb10.NT.117_2 [448 - 558]

MLGQSMESVTFFEILLGHSPARRWSYVLAVFSLPHRL

>Tb10.NT.117_3 [524 - 619]

MFSQCFLSHIAFEMMCVARRRGAFSFRSQLNK

>Tb10.NT.119_1 [154 - 246]

MLIVSPCISEGMVIFRNEFGVCVACRCEGTV

>Tb10.NT.119_2 [203 - 316]

MNLVFVWLVGARERCKSCPSCWCVFCPFGCWAVVLNTP

>Tb10.NT.119_3 [289 - 429]

MLGCGTEYAVGWSRTSDFFVPLCDCRGWEGSASSDHSGWKVCQPLSC

>Tb10.NT.119_4 [715 - 804]

MFLTFKGEGGERAGKSAGTFYDCASFFYFF

>Tb10.NT.119_5 [776 - 982]

MTVPVFFTFFDACRRVCSKTVWVVGAGLAAPHFHNFWGIVESPYSTHVCKHALAAVLAPLGVSSLSVEQ

>Tb10.NT.119_6 [807 - 1037]

MPVGVFALKPCGLWVRVLLPPIFIIFGGLSSRHTQRMFVNMPWPPYLRHSGCRPFRLNSDFAHGELQGSLRCAAPVH

>Tb10.NT.119_7 [994 - 1083]

MENCRVLSAALRLCINKCKQRLFRCLCFLT

>Tb10.NT.120_1 [7 - 81]

MLVNPFNVCSVTINGERLGESNVQF

>Tb10.NT.121_1 [313 - 444]

MQREADTCGNISKHKHGYVQPTSPVACDLNILSQKRRTTRLFRK

>Tb10.NT.121_2 [456 - 560]

MGRHLCALRLFPSSLISVWTTRVIRTMLIWVQSFL

>Tb10.NT.121_3 [365 - 574]

MCSRLLQLPVTSTFFPRKGVQHGCSGSEAADGSPFVCSATFPLFPHIRVDYSSDSHNVDMGTIFFVSSSF

>Tb10.NT.121_4 [675 - 860]

MQQPAPQFVVLKVEYGVSSAQQLITGEDNHVSYHFLLFSRTLVPCPGFVSRYHWCVALFMVF

>Tb10.NT.122_1 [783 - 878]

MKNDVLRNKIRLCGNFFLAPTVHSYYIWSPMR

>Tb10.NT.122_2 [964 - 1038]

MLSDWKLTRSSKMMALGDRARTFIG

>Tb10.NT.123_1 [56 - 142]

MDGMRSTRYITLFTVTARALIAVCRLLLS

>Tb10.NT.123_2 [173 - 253]

MLIEGHGLYDSAGFNFVRGSGFHRIDA

>Tb10.NT.123_3 [1349 - 1462]

MVFVVFVFVGRWGLAEQRNGGQFSLHVCQAVHGVIDHF

>Tb10.NT.123_4 [1294 - 1533]

MVFFTTRIIRVAPVRCAVNGFCCIRICGQVGVGRTKEWRTVFPSCLPGCPRCNRSLLMMLRIMSPWNLMVSLCFNGPIRY

>Tb10.NT.123_5 [1517 - 1606]

MVLLGIENFSFSHLSLETFLFIFHYNRVSG

>Tb10.NT.124_1 [102 - 407]

MGTFMRWYHRYARYLSCSLGGGGFKDCVVLSQRPRVHREWRAVPQGLFPFLSLALCHVVISRLPHWLRCVYVCAHPLKRNGLWLVGGRERHHLAFIVISLFS

>Tb10.NT.124_2 [576 - 689]

MNVFCYSSFVTTVLIVARGGVFVNPFVDTINAAVSIRS

>Tb10.NT.124_3 [1074 - 1316]

MSMLVCRIFGSILPVKPCSPIHLGERVISFLLGVCNYSGVLRYRFWGCRGRRRILCYGSAKGFQWNCNLLLSPVMKYFCAF

>Tb10.NT.124_4 [1370 - 1468]

MKELLKGKSLATKSVHSIQAAFRTKFRKGHLLF

>Tb10.NT.124_5 [1647 - 1733]

MPWFGSGYLRGIFHFFFLPLHFRNWLCVF

>Tb10.NT.125_1 [44 - 262]

MTAPHWLSFLCIAPRSVNVEERMCWNGLGALRNTFRTMDKSQSWSVKSVLVCRSATGGASHPSGSPVLVCFAV

>Tb10.NT.125_2 [386 - 550]

MCQVKNNFLLCTCRMFSFPRPFSGTRVFHLKSRSCRYMNVHFFVHIHTCPLRTRR

>Tb10.NT.126_1 [100 - 357]

MTLHRCGMRESSMFLLPPPLSAPGRRGVLNVLLAIWWIFRRPAMREIVHRGGHQITALFISVELPSTRCRSDACDAEQREGSYRQR

>Tb10.NT.126_2 [318 - 419]

MRCRTAGRQLQAKVVDETLLCRVLSSVPKCLSEA

>Tb10.NT.127_1 [244 - 348]

MEIKLHAVLAPSARVTGRTSPFVLREKNCRWPLPP

>Tb10.NT.128_1 [98 - 181]

MVLACVPRHLRYIVLAGSMKCKDRRQGK

>Tb10.NT.128_2 [157 - 231]

MQGSSAGKVNLWKSTGNVIFFRYNV

>Tb10.NT.128_3 [258 - 464]

MIKQPQKHQNFTMRPNKKKHKNDPEVEPPPQTHFCYSGSKTVALGVACHSSDTAFAPTLKLSRAGWGLP

>Tb10.NT.128_4 [606 - 680]

MVCLVASRHVPHYRFVASVCWVRLL

>Tb10.NT.128_5 [723 - 908]

MRSNAGSVSAPGDGRKQLLFPLFYGFWCGNSTALGKREQYLVAVLWYANSAEVSGNMRPSYR

>Tb10.NT.128_6 [862 - 954]

MLIPLKFLGICDQVTGSTECRTTMAKVLCIY

>Tb10.NT.128_7 [918 - 1010]

MSYNYGKSFMHLLMPSRVAGGFHGYEGKIFF

>Tb10.NT.128_8 [1494 - 1610]

MVALVRKSLNQEVLVTSLAECVFSGRLVASAVCLRSQCR

>Tb10.NT.128_9 [1800 - 1907]

MENDLERSVRLHLSYGRIFEGKSAPALAIGLFGNET

>Tb10.NT.128_10 [1807 - 1929]

MIWNAASDCISPTVVSLKVSQPLHWRLGCSGTKPDTDLRHQ

>Tb10.NT.128_11 [1929 - 2003]

MILVLLLCYQKNGSELWRRVLGPPP

>Tb10.NT.128_12 [1963 - 2040]

MGRNCGVECWAPPPKGLQLPLRSGWL

>Tb10.NT.128_13 [2121 - 2201]

MRTLRFHSHPIEAVCSALAFLPQQPVP

>Tb10.NT.128_14 [2247 - 2519]

MCGSSFLVEGSGSTAPKPVSDLTLRWSVGTCVAQIPSQAGSLLLWAFALHAFDRLLHLVYHFLRSPPALVLYSWCASGTVTPTCVETSFKA

>Tb10.NT.128_15 [2495 - 2593]

MCGNFFQGLVDWCLWMALTQLGAESPGICFHVW

>Tb10.NT.128_16 [2672 - 2788]

MLPAASSDPDLFVFLIQFVLLMRFPLVLLPATRDGGGPW

>Tb10.NT.128_17 [2865 - 2951]

MGVAYRFLHRNVICGICTDADSVESPCHC

>Tb10.NT.128_18 [2957 - 3031]

MFLIVFQFCFSVIPSNFYVDGALSS

>Tb10.NT.128_19 [2920 - 3099]

MLTRLKVLAIAKYVFDCLPILLLSDPLQLLCGRSSVQLTFLICAGALAASLWMPQTAIYS

>Tb10.NT.128_20 [3009 - 3146]

MWTELCPVDISHMRGGSCRITMDAANRDIQLNECVLARPCSVLRPR*

>Tb10.NT.129_1 [146 - 304]

MLDIVLAEEGKYGSDERQPYYRCWAFSFSLEAWMRWLTWRVPTVEGKVRFGAS

>Tb10.NT.129_2 [328 - 447]

MGPLAWCPDELLLYACEFGGALVACRYSVSVSVEYLCLCV

>Tb10.NT.129_3 [353 - 568]

MSFCFMRVSLVVRLLLVDIAFLYRSSICVCACRGCGGCQLLAEANALSNRTHAHTHIHTQGSLKKRYAVRRH

>Tb10.NT.129_4 [507 - 686]

MRTRIYTLRDPLKKGMQSEGIDIVDGGQGVGKLQFTKKTTGKMLFILRMILERVIFQWTS

>Tb10.NT.129_5 [708 - 896]

MLTPAGKCNGQARKVTIQECKGILLNTQRCPRRRLLVSDRGAAPFLLQHQTSLNSSGKCTIFA

>Tb10.NT.129_6 [1237 - 1329]

MIFLPFKRCMCVLGIMPPFLPFWFGFPSVTE

>Tb10.NT.129_7 [1260 - 1394]

MYVRPWYYAALPTILVWFPVRYRVGAGGRLMWWQGKTTRYKSVSW

>Tb10.NT.129_8 [1481 - 1561]

MFYLLIFVRAPATSFPHVPSGGVYTGR

>Tb10.NT.129_9 [1530 - 1682]

MFLLEVFTRAGNHVASCVWKTFACLGLVPVLFCFVLSFLVVRIVVWGTRVL

>Tb10.NT.129_10 [1598 - 1837]

MFGIGSCFVLFCFELLGRPHCCLGYPGAVSGGVLVFLPCMMGYIYLGFCGCTAVSHVGALGITAVMHARESLFLLQLDIF

>Tb10.NT.129_11 [1764 - 1838]

MSVHWVSLRLCTPGKAFFFFSWIFS

>Tb10.NT.130_1 [184 - 321]

MMWIRCSTVVEGIDRVGWIIFFFILLSQTLKWESVRGANTTLTCAS

>Tb10.NT.130_2 [276 - 455]

MGVGAWRQHYPHLRLVKVYRVTYGAAKLFGHLSLFARKLPFFRCCSVILVARKHFKVQNS

>Tb10.NT.130_3 [464 - 562]

MAMQLLTFFFFLIDVGPKLLLSLLEARVYFYIF

>Tb10.NT.130_4 [621 - 698]

MEKTETCRGVLSRLPRFPLPLLWIPS

>Tb10.NT.131_1 [42 - 176]

MCWLTVYAVSQRGTGCALMTGRRPIFRPDPQYPAPRASQRNTLGL

>Tb10.NT.131_2 [484 - 567]

MRCRALLNDCAHSCVLMVYALPTGSNQG

>Tb10.NT.131_3 [621 - 761]

MPGNVPACLVDSRDKIVCCMSQIECNSEVLQLGLPSLLRWTVSEECG

>Tb10.NT.131_4 [755 - 829]

MRLNIACGKCPVRWICVGEVIIPMC

>Tb10.NT.131_5 [946 - 1023]

MIDFDTGRMLSVKVLVRHIFYKFKFK

>Tb10.NT.131_6 [1209 - 1295]

MEYVAGDQLEDIINKTQVRPINVPITCTF

>Tb10.NT.131_7 [1273 - 1563]

MCQSPVHSKTTAGFGIPLIILCYPLKDREYQHAFVSDREVKMSWMWSNLLYFSLFYLFKKLSSVFIFNIFQNWIPFPLWFDRVSSLPSLFPCGTIQI

>Tb10.NT.131_8 [1367 - 1621]

MLLSAIGKLKCRGCGAIFFIFRSSIYSKNLAQFLFLIYSKIGFLFLYGLTVYRRYHLYSPVGLYRYKLFSSHCHRFDPFHTIGLF

>Tb10.NT.131_9 [1923 - 2021]

MYKCERQCTFYTNQWGYLFIFSFFFVIIYYFFY

>Tb10.NT.131_10 [2024 - 2155]

MCWIILFSLLGFYSGNVCLWRRKTSTPQKKKKSAEHMRIRERER

>Tb10.NT.131_11 [2523 - 2630]

MKLRDVCVCVGGQCFIPFIHLHAQCASAKFMCVNVP

>Tb10.NT.131_12 [2787 - 2882]

MRVSHFFLWLLRPCVCTGFVDCGRIGYAFQSH

>Tb10.NT.133_1 [41 - 319]

MQRKHRCPNNQRYCTWREVCTKKHSHLDVIATSCSPRSTRPLNGYGLIGLSRRLCYGRASPNVSFSSFIVPLICSTGGRSGRCGGGGSAVPWS

>Tb10.NT.134_1 [21 - 104]

MWTVMGEAHCNSNLRTVTNVTSDLFCCV

>Tb10.NT.134_2 [76 - 168]

MSRVIYFVVFDNGSFHCWAAVDLKGETRGEQ

>Tb10.NT.134_3 [372 - 494]

MCKYTNTVSILKSPVAIKNVCLHSVSLSSVGRTFVLSLVGS

>Tb10.NT.134_4 [427 - 525]

MFAYIRSLYPALDAPLYFHSWAVSFPHSGYFAV

>Tb10.NT.135_1 [113 - 205]

MHTIVFLERSYTGFTVTVRGGNCRMKEVAKE

>Tb10.NT.135_2 [282 - 383]

MSRRNRTKCSISSQLIFEFPHMFSAVHAVFVLWF

>Tb10.NT.135_3 [735 - 818]

MGTDHMPKMGSNVVRGEVEATAIALLCS

>Tb10.NT.136_1 [359 - 436]

MRAVLVPNINDNSSRMLFDCFSPFAL

>Tb10.NT.137_1 [412 - 534]

MSYLLRFTSTNACAAHVLVPVSSTHCSFACARIRIHLQALR

>Tb10.NT.138_1 [210 - 299]

MGAFGYCVGDWCRWWRYFFSRLDIVICVGV

>Tb10.NT.138_2 [130 - 306]

MCHSRKYLLPSGAAFRVPPLSVEAVHIWGHLDIVSVTGAGGGATFSLVLTSLSALVSDR

>Tb10.NT.138_3 [602 - 754]

MKGTECRCWWTMPVAGNDKRTRPSVLTKWPLLLLVFLVKLMTFFLVLTADA

>Tb10.NT.139_1 [96 - 170]

MDVVLSGKVTFDSAVNVRRPNITGR

>Tb10.NT.139_2 [422 - 538]

MVLQTPGANPSTAFSATHLKFAVTNVEIKHASLRTPGGA

>Tb10.NT.139_3 [142 - 654]

MFDARISRVGEFTIIKGRQFLYFNVNIQRLYSGQLVAVRKKLCSRDLESRERTTRSYVSCEMYYHKWRGKLQGLVGSRGTGSVGQSPQIGGGGDGPPDTGCQPFNCILCNAFEICSYQRRDQARFSSNPWRRLAVIRCLNKNINLLDLGGLDMLLPASALVASLISTSLYL

>Tb10.NT.139_4 [814 - 897]

MNPYRLLRFFFLPLYSAIFAHSPPSLHT

>Tb10.NT.140_1 [236 - 316]

MYLFVFLLMHMHVIILCVMLHSMEMCI

>Tb10.NT.140_2 [217 - 330]

MASWQLNVFICLSAYAYACDNTLCYVTQYGNVHLSICC

>Tb10.NT.185_1 [145 - 267]

MGRGGEMARHFCNLNGPCTFTVFVFARIHNVTLQVIARQKE

>Tb10.NT.185_2 [26 - 352]

MRSNVNRKCPYIADETEKVWSARLISLHPSTPSGTWFTLEWEGAAKWRGIFVILTDLVRLLSSYSREYTTLLYKLLRGRKNDVEAKWRGSCTHETVGTRNFPVLRRSFM

>Tb10.NT.185_3 [267 - 359]

MMLKQSGADRAPTKRWGHGTFQFYDAASCKR

>Tb10.NT.185_4 [614 - 709]

MGAMSCCFAEGAIFFALVIVDRWCHSHCTYTL

>Tb10.NT.185_5 [750 - 866]

MPHVCRAVVAFTSPFIFMLCFPLAHRCLPHGIFCGFVGR

>Tb10.NT.185_6 [866 - 955]

MRQACAQTNVPYLKSNLLFRQIILAISLPK

>Tb10.NT.185_7 [968 - 1096]

MSFGVGLCFLFRELKCNLCVRGAVFIPCTYRKGGWMEMIIFFL

>Tb10.NT.185_8 [838 - 1188]

MAYSVDLLGDETGLCPDKRTVFEVQPSIQTDNTRHQLTEVMLTDEFRCRAVFFIQRVEVQFVCQGRRFHTLYISQRWVDGNDYFFSLMQRSTLCSSRITRRCAEVTFEAYYPVRKAR

>Tb10.NT.185_9 [1340 - 1417]

MEEGGHACRKCYLVVRVKAVKLVQLS

>Tb10.NT.185_10 [1369 - 1452]

MLLGSSCEGCKARAVELIANGVLMLRQR

>Tb10.NT.185_11 [1483 - 1707]

MFQGSFCWMVVFHIFYFPFLHTKFTVVGKQLSRASPWLYKCCVHWKWLPKLPHYMKEEACVLSKPRNTCLSTCFA

>Tb10.NT.141_1 [215 - 316]

MIACCRMRQFISAPCVGCVLPTVLVSGASSYDVK

>Tb10.NT.141_2 [336 - 419]

MAEKWPDVVQHNSNTQHRAIFPPSRLAV

>Tb10.NT.141_3 [427 - 510]

MVPLTFTNPERRCLLPHGFSLQEMGSVL

>Tb10.NT.142_1 [224 - 409]

MHDVVTSASVFFLFFHDHTAKNCCFFIWGGGEGRKNCEGQTPIYHIWKSLFLPCFHPKQRWC

>Tb10.NT.142_2 [361 - 465]

MEIAFSPLFPPEATMVLNWEKWLWLKQVTRVTMIV

>Tb10.NT.142_3 [270 - 503]

MITLLKIVVFLFGGGVRDEKTVKDKHQFITYGNRFFSPVSTRSNDGVELGEVALVEAGDTRYHDCVDFNCVTDHTDVR

>Tb10.NT.144_1 [107 - 457]

MNPPEESEDANAPLDRLAAHLRAPPTPPKVPVYALPDQPYLETTVLPLLLRGLEEVVKVRPADPLAFLAAYLLSNNPQRVSHPLLTEEGRRVPLQEIAQRAADVIKQLAYQPPAQPK

>Tb10.NT.145_1 [309 - 383]

MPRQRLSAMVALPCSAAPLVVRQSW

>Tb10.NT.145_2 [161 - 418]

MGFFDSDLSYPTVRTERMKHKRRRLVQGPNSYFMDVKCPGCKNITVVYSHATSEVKCNGCATMLCRPTGGKAILVTGCGFRKKPDH

>Tb10.NT.145_3 [435 - 545]

MGFHRIGVVKQMLSYFPLGLQWNEKQIFRFLFFFACS

>Tb10.NT.146_1 [159 - 239]

MEHIVSISLYPKVSTLYCRIDFYGSGR

>Tb10.NT.146_2 [239 - 364]

MNPFVLYIVLCLTEAFTLRMCLMLTMLAAFEKIVTLNPLLPA

>Tb10.NT.146_3 [382 - 462]

MCVHFPYCWQPVVHGLRTEGAVLLSAS

>Tb10.NT.146_4 [546 - 668]

MMLFLPSLLFAATFYYSVLRPSLLFFHFVYFDAFVLKGESF

>Tb10.NT.147_1 [171 - 260]

MEATVVDMCALPQRMRVLLHVEELRNCATS

>Tb10.NT.147_2 [397 - 597]

MWCIFRCLSCYCFGPISLSGGWRSDCANLLPLRCPHYVNTMGIYGGVFQSEGNIVPPLAPFFQPQCF

>Tb10.NT.147_3 [527 - 601]

MVASFRVKGISFLRSPPSFSHSAFE

>Tb10.NT.147_4 [898 - 975]

MPWRCFLVTIAIEHRLTYPDATMLLR

>Tb10.NT.147_5 [996 - 1100]

MKAFRCLSRPLHNPFPKRGAHKGKRGLLFNYTCPF

>Tb10.NT.149_1 [172 - 249]

MCGLPRGGSVINPLFLSCTESSVVTT

>Tb10.NT.150_1 [228 - 431]

MHPSVHCLLNTGIVVWSSLACPQLTVRRSILRCMEELPTGKGYSSFGLQTRSEAQMSTQKRCCYDANQ

>Tb10.NT.151_1 [116 - 205]

MPEVPHVARACPVKRMRERTVGAGPAGCDS

>Tb10.NT.151_2 [340 - 429]

MLPCICTAGRGRRLLDQYFFCSFFFLKREV

>Tb10.NT.152_1 [228 - 374]

MPLSGTNLSLLQPQALFVFISCGERIVRFPFWVLFSVPTSSVPLTYECV

>Tb10.NT.152_2 [381 - 491]

MCVFFFFHFPFSSFVIVINIVIIVLFFTFSEPIPKEG

>Tb10.NT.153_1 [35 - 115]

MFPTSTNKDSVHYIRSRCISSVMSWFP

>Tb10.NT.153_2 [177 - 326]

MRLPVSAVVNALWWKSRHYMARDTSQKCEPHSGASAAGQQQWKHKILKLR

>Tb10.NT.153_3 [899 - 982]

MAIEDHIPTHTHTICAIWLLPGKRVYVK

>Tb10.NT.153_4 [769 - 1056]

MTRSVDGDPLFFWWKVRAWGLGPAFNFRPVAPFRVPLLGLLFVDGYRGSYTHTHTHNMCNMVVARKARLCEMKPTEVAERLCGQRTVSCFLLFFRS

>Tb10.NT.155_1 [138 - 248]

MTGEFCWWCIVDSAKTTERGCALDLDFFSTLKGGVVI

>Tb10.NT.157_1 [443 - 550]

MGLKTLLDPEAIIPNLHNRLFIFGVTFRHCTHNCRE

>Tb10.NT.158_1 [255 - 353]

MGFLNFSLFLRYIPVGLLTYRNHIYISFFIYIV

>Tb10.NT.158_2 [357 - 494]

MSHTCARRGCRSGLKEGEFYRCERSLCVYISMFYPCPYFRSHADLL

>Tb10.NT.159_1 [106 - 342]

MNSMEGLCCNLTILTEGSKCKHFFFSISFLRQHCNSCQRVVTDVVPHYKADYLHCRGLTEFLCKCTQRIFSREAERHKV

>Tb10.NT.159_2 [233 - 583]

MLCPITKRTTCTAVASPNFCVSVRSVYSHGRLRDIRFSFIVSFRAYIRARTCIICSKNKRDGVALVLFIFLFLTGGGGVMQLLGCCCEEKRLSQAYYMRPLIYAGGGGEKKIEAFVQ

>Tb10.NT.159_3 [540 - 749]

MLVVVGKKRLRLLCSDVVAKHVLCNVGPLKLVILLTLFCCIPLFSPFAATSCYFSLPLNIVCKNTQGNVG

>Tb10.NT.160_1 [24 - 176]

MQQADYYAILGVPRTASKDGIREAYKLKALELHPDKNPEGEAIFKLVVNSW

>Tb10.NT.160_2 [173 - 280]

MVNSLDVVLLLCMIIFFVGDPSLCIFLFLFFFKNSG

>Tb10.NT.161_1 [125 - 259]

MTDVNGLSVSLHAVSPGGGVCEVKRSLSNGTATGMISHFRAHQVR

>Tb10.NT.161_2 [496 - 570]

MMDFFFPFPSVRCCPLCADSWILNP

>Tb10.NT.161_3 [309 - 767]

MSNTKWSIRFSGRGVLERSCCCFLRRGIDSGRFEERDSNELSHHALCRIKIKKKLENRSVPCHDGLFFSVSLGEVLPFMRRFMDFKSLTDTGIAFCLLVFYFPLSLFLIPFLRLGLCGSNNMLVSTWRLYRGSEGGQCFAPVHLCVVITLWPH

>Tb10.NT.162_1 [396 - 479]

MLLMVITGIFWWCPVATRKKDAKGDKRN

>Tb10.NT.162_2 [457 - 540]

MQKVINEIKKTLSVKKGIWNQHESIITA

>Tb10.NT.163_1 [118 - 237]

MVRKAGWSHEKLCVRAMDTLLLPTSGNNHCRLFAAVNRKK

>Tb10.NT.163_2 [143 - 277]

MKSYALGPWTLFYYRLRVIIIVACLPPLTEKNDVWSGFVSMLSNA

>Tb10.NT.163_3 [521 - 658]

MTTVRFHNERSTDNVADTPNVLMKAGPLLRERRLSFTPRMPQSNEL

>Tb10.NT.163_4 [700 - 804]

MPNLEYRVRLSAPIHPIYYWKRCRGLVDAGFSLFL

>Tb10.NT.163_5 [959 - 1036]

MGFGTSRYFPSGSFPMFRPSPSWKRC

>Tb10.NT.163_6 [895 - 1086]

MLISLCTPVTESSIWTAGFSPDGLWNLTILSVRVVPHVQTISILEAVLIYELRFRTVTTGRILL

>Tb10.NT.163_7 [1226 - 1456]

MQFLMRIGTLFLSHKGFTWGGGVGLDLQIVVDAPKRRISPPVVGCSRGCEITSPVSKTLERFEFIHWGDLNAFYESM

>Tb10.NT.163_8 [1791 - 1880]

MLLYLRLMVTFILKGEKKIKGDLYGRHSQN

>Tb10.NT.163_9 [1861 - 1977]

MEGIVRIEIVAWPTTRTLLNGGPEVLEFRYAMILIPKYS

>Tb10.NT.163_10 [2119 - 2247]

MSAISHCFTRFQVLRSDFCRPLSVREFLLTNRGSCHLRMCAPS

>Tb10.NT.164_1 [38 - 247]

MLSLFILYFPDPQNPLKKKREKRKRERKKKKETPLPPLFPISLYRPTSHLFSPSFVYLMWLFKPVPACRK

>Tb10.NT.164_2 [767 - 868]

MIFFIRLNDSIVLLRVEECSALHGLATESFVVCL

>Tb10.NT.164_3 [745 - 936]

MFICSNFDDLLHQVERFDCAAEGGGVQCAARTCNREFCCLFVIAILFPPSPPQQMIPPTLFRVL

>Tb10.NT.167_1 [57 - 287]

MILGSERFKRLKGDGGKRKEEDKQTNKQMLKYIKKSTRDKMLVAVVARGDNKRGETLPNRRYEMEIRKCHTRRRASA

>Tb10.NT.174_1 [271 - 345]

MSHLGRQVVEQMETAMFSYFFFDCN

>Tb10.NT.174_2 [348 - 467]

MCRARHLPAVWTQFHRIPLARSHACATVLCLFFLRVYVCV

>Tb10.NT.174_3 [158 - 487]

MAWGNSVVVTVFILFLFSLHYSPPACGNRVQLIPFNIACPTWVARLWNRWKQLCFLIFFSIVINVPRSTLAGGVDSVPPDPACTLSCMCHRVVSFFFACVCVCVTSGGYE

>Tb10.NT.174_4 [483 - 590]

MNEICERRLNGLFSPIHPFMGNYVRLRRVCSFLYST

>Tb10.NT.192_1 [66 - 149]

MLWPGNTVAHDVLNWNYHLNAGYRKSMN

>Tb10.NT.178_1 [215 - 295]

MGLLVSSVKPRRSWRSVVAAERHPPGA

>Tb10.NT.178_2 [614 - 727]

MHCDSTDSRPILLLHPLQDTVLQSVEAHQCPVCHKRSL

>Tb10.NT.178_3 [591 - 860]

MLAEHQVTCIATQLIPAPYYSYTLCKTLCCSRWKPINVLFATNGLFKIVDFDWVPCEMSLYVPSDTYGSLYGRAIIGGRTARTGHRKFAK

>Tb10.NT.178_4 [860 - 940]

MKPQPREISTFCSALWIPPSPIITLNG

>Tb10.NT.178_5 [790 - 948]

MAPSTDVQSLGVALHEPVTGNSQNETPTARNFDILFCSVDSPLPNYYSQRLKS

>Tb10.NT.178_6 [1289 - 1372]

MTGGGSCSLRSDQRALEVLLRMWILFFF

>Tb10.NT.178_7 [1381 - 1470]

MDRPVSDPIMRRGRAAEETGWGESDGNFVT

>Tb10.NT.178_8 [1549 - 1719]

MYKGIVDYLLMCVFPFPLILSSYRLFYVDDSYFFLRLSSRVPASGNIFFFKRTWHPL

>Tb10.NT.178_9 [1628 - 1723]

MLTILIFFSASLHVCLRRVTFFFSSALGTPYN

>Tb11.NT.2_1 [25 - 285]

MLRRLHVPITSNFLVHMRSMSRPLLATSVRCGTNGCDGQKKPANDDEDYGALFDSDFEFLDEFFEDGIDVLDYIPESEASLLGGDGR

>Tb11.NT.2_2 [125 - 292]

MVVMDKKNQRMMMRIMVHYLTQTSNSSTSSLKMGSMFSITFPKVRLLFWVETAAKY

>Tb11.NT.3_1 [112 - 339]

MPLSPTRMIRVKAQKQNSQISMPRLSDEEKKKLREKRENASALPSWLGATILFIVLGSTVVQIYFTITSHRGGVVE

>Tb11.NT.3_2 [191 - 379]

MRKRKNYARSVKMPLHCPVGLVQQFYLLFSVQRWFKYILPLLPIGAVWLSEFRKWSFLTKRFK

>Tb11.NT.3_3 [379 - 483]

MTSNGKMTKPVHRLHSVLVMFYNLQRVGTPVTEVN

>Tb11.NT.3_4 [569 - 649]

MVGWKDCTPFFVTVVIVFVLISTFLFF

>Tb11.NT.3_5 [577 - 705]

MEGLYPFFCYCCYCFRLDFHFPFFLIYSEKKKKQLEKNKMDIC

>Tb11.NT.3_6 [773 - 847]

MRHRTLEGRIIPVHVSAPSLPYKEI

>Tb11.NT.3_7 [851 - 952]

MQITVYFNILYIYLHFTSRVVSPSTTLPKAFAIT

>Tb11.NT.4_1 [186 - 392]

MFATAPIKVCCEMCCNLAFEETVFPLSLSRYIFIYNALFTWWWRFITQYFTSFRDVLNPFIGVFMAIWL

>Tb11.NT.4_2 [552 - 632]

MCVYNFDWDYQFPFAVINASSQGGMLK

>Tb11.NT.4_3 [667 - 765]

MLICEKLSAQCFQRWVLEYFLECFFCPSAFGCD

>Tb11.NT.4_4 [930 - 1160]

MPFGVVHILYCITLYNIIFRIVLLLTFFGRCFVTEKLFFFALTRLAFREAGGITCLYKYMYVYVPLFERNVHCQIPL

>Tb11.NT.4_5 [1182 - 1271]

MCLINALWLFFFFTNVLLTRSPNFPPLSHL

>Tb11.NT.4_6 [1335 - 1445]

MRPRSRMLCDRYISYGDVWEFLCSPTVLPLHFKWHCL

>Tb11.NT.4_7 [1282 - 1449]

MIPSLVSIPDYYYYFFFLCGHEVACCAIGTFLMVMFGSSCVRPPCFPCISSGTVFE

>Tb11.NT.4_8 [1449 - 1529]

MSSVCACAQSSHVSFFFLSFSDRDEKP

>Tb11.NT.4_9 [1556 - 1630]

MKRKHHRVFFAYWRGCSCRFFFFPF

>Tb11.NT.4_10 [1519 - 1647]

MKSREVMAICMHNEAEAPQSVFCLLAWMFLSLFFLSVLTLFAP

>Tb11.NT.5_1 [28 - 135]

MRMRLMSAVVLMGSCLQLRGVQPGTAGSPRRGLFSD

>Tb11.NT.5_2 [12 - 287]

MTLSSDADAADERSSVDGQLPPTERCATGHGREPEEGTFLGLTADERRAGFIVISFLLFQEVDSNVFRGLLACSIAAYTVKQYVGWYRSCQP

>Tb11.NT.5_3 [259 - 363]

MLGGIAVVSHKVKRLWIVTTLFLSPLATAYSTSFL

>Tb11.NT.6_1 [162 - 437]

MNFVKRLVYSLAVEKGAESPLIRQVAQRTAHMERHAVKRHSLWLSAAYAEVKRDVSNLWSKLQEGARKENDPQERISHAKKSCEDKSGVPRN

>Tb11.NT.6_2 [322 - 567]

MFLISGRNCRRERERKTTHRSVSHMQRKVAKTKVASRVTNGVREDYLLLWFSFSCWKSEIGGMRSYLQDMVVCTAKRYIPKV

>Tb11.NT.7_1 [138 - 224]

MSPLPRRFMSNSNKNNNANNNRRSHGNGI

>Tb11.NT.7_2 [122 - 334]

MENITNVSIAAKVHEQLQQKQQRQQQPEIPRERYLVAYLLLIGIVGTMVFLFIRRGAIRRLGREYSGVSNL

>Tb11.NT.7_3 [587 - 664]

MYMSSLMFTALLWAHVRVCYQFLPVL

>Tb11.NT.7_4 [664 - 768]

MIDVSSCRMRGVLCRIFFLFPLLLLFLSSSLPSLS

>Tb11.NT.7_5 [671 - 862]

MSAAAGCAEFCVAFFFYSPFSSSSFLPPYHHYHKISPQLGKLFVFIIFTCTHLFVCLLFDEVAV

>Tb11.NT.7_6 [817 - 921]

MHTFVRVLTVRRGCGLSEVNAEPPSLQWFVSSRRF

>Tb11.NT.8_1 [6 - 92]

MLGINDRSNDGVVLKVAAKEILSGLFGLH

>Tb11.NT.8_2 [301 - 495]

MCWSLFSSSYLLHFYIIWYYNLNGVMLKSILRADEGRNEPFFIFFILIIRRCIFDVMELLQNSCE

>Tb11.NT.8_3 [464 - 598]

MLWNYYKTHVSNCDGVINFAFLFFFFCRHDIVTSKRVRKSLWFNL

>Tb11.NT.9_1 [141 - 344]

MLMVLMGLSVISENGSIYTCICMLQIWLHHVCACKIGGRENTQEVVISMLFLSCSLGKFLGALFRRFR

>Tb11.NT.10_1 [165 - 323]

MDNDRHVSAVFLYWVYPNGDYRVFVSCSIHAALLALPGQELLRVTGVHKGVYS

>Tb11.NT.10_2 [242 - 337]

MQHTCRTTRTSRTRTTTRHRCTQRSLFINTSV

>Tb11.NT.10_3 [73 - 357]

MLRRSLIVRGHATSNLFAPPFSGRWFNPPHQWTMTDTFLPFFFIGFTPMAIIVYLYHAAYMPHYSHFQDKNYYASQVYTKEFIHKYKRLERWRWY

>Tb11.NT.10_4 [423 - 539]

MRMYTYTLPFQIAIGKGPDREVGEPQGDPSELFANGALA

>Tb11.NT.10_5 [650 - 724]

MRVCFVGSAMRHLMDPLLPDAACPL

>Tb11.NT.12_1 [119 - 295]

MVSVVLWPLRVRWTLGAGTDTSVLTDSKKRQKTSQLFPKESEVYPLKSSADLSLCTRAG

>Tb11.NT.12_2 [472 - 546]

MCNPLSAGASCGWDADRFFFCNGRE

>Tb11.NT.13_1 [75 - 200]

MDPRVKAVCFHQQCPRVGCFPFLSFHYSTVSSLLELRFDKRN

>Tb11.NT.13_2 [309 - 389]

MCMLFLGVVSWHFCGQMMFVYVYGTSK

>Tb11.NT.13_3 [399 - 482]

MYELTKGICASVTSVFSSCTVMSLWYSY

>Tb11.NT.13_4 [588 - 677]

MPLLTVEVGVANIMICPFDNTVLKEELSLF

>Tb11.NT.13_5 [835 - 924]

MIEFFLIFVFTHLILKDLHMPSFSLLLSCS

>Tb11.NT.13_6 [981 - 1079]

MSRLLRSLRHQRLLFVIYVYLGTTVFHSFPFVL

>Tb11.NT.14_1 [4 - 114]

MYSKNNGPGNSSKGMSRKCSFCHNPTAQEYRGSINKK

>Tb11.NT.14_2 [199 - 321]

MFVCLCVCVCANPLVVEVLLDYIAPLLLVVDGIWRIDHLKG

>Tb11.NT.15_1 [26 - 100]

MCIFKFCIRLGAVVNCDGVAQIFMA

>Tb11.NT.15_2 [75 - 233]

MGSRKFSWHKSLLRRVGLNRPTMRSTRCDFVGLKVYVMSYLASGVSCRLRRRR

>Tb11.NT.15_3 [247 - 339]

MPFCSFNNRFTFFNAQIRGGDSGETEGDTYR

>Tb11.NT.15_4 [287 - 385]

MHKYVAVTAARRKGIHTDETLLFIVFFFALRVM

>Tb11.NT.16_1 [119 - 220]

MKQWLRIILCLKKRREKMRKSRTQKRKDIGGVND

>Tb11.NT.16_2 [278 - 379]

MTHFTNGATTVGFPQEAFSNVLYLRIVTIARDRK

>Tb11.NT.16_3 [476 - 562]

MHIHMCKFACVYFPSPESNYIVTLCLVLC

>Tb11.NT.17_1 [44 - 157]

MHYNTAYVILFSFLKKKTYSYRSSAHFMLLGRGKDINV

>Tb11.NT.17_2 [153 - 272]

MYNTCIYIYIYIVHLKGGRKRKRALLRYVSSVICVAGGGA

>Tb11.NT.17_3 [164 - 361]

MHIYIYIYCASKRRKETKESVVAICFFSYLCCWWWCVSEKIIRIRVLDELPEVSAPTTKKNNISTA

>Tb11.NT.19_1 [220 - 303]

MLGWKMDRLWCVGLSLFGVEALCAFLLL

>Tb11.NT.19_2 [203 - 340]

MRYRLVCWAGRWIACGVLGFLYLVWKLYAHFCCCRGGENHILCTWW

>Tb11.NT.19_3 [285 - 485]

MRIFVVVEVVKITFYALGGRSIGRVLMFFMPLFTHSNGATSAGELGSLRSVAFFFLRLSIQYLLASW

>Tb11.NT.19_4 [394 - 492]

MAPPQPGNSALFEVSHSFSSACPYNTYSQAGEN

>Tb11.NT.19_5 [690 - 773]

MPKRTHVPYSGRNASSALWPLPRVYWGV

>Tb11.NT.19_6 [664 - 903]

MPFERRLRVCQSGPMCPTRAATPAARYGLYQGFTGEYSRFSIFLHHSSLWLYFPLSFYNPSALVDEWMCYVGSCSITAKW

>Tb11.NT.19_7 [743 - 916]

MAFTKGLLGSIVVSPYSCIIVPSGCISHFLSIILLRWLTNGCVTWAVAPSLRNGRRWG

>Tb11.NT.19_8 [1163 - 1351]

MRRMTTLFFFPLYPQRGPSQGPTHCLRQNAQLLICFVSCWGKISVRASFEERKQLCIYALKGI

>Tb11.NT.19_9 [1327 - 1509]

MYLRFKGHLIKRKYLSTEFLVVVSVSDVPVKVNNWSVDVSRNAKHFHLVVEAVASSEHVFS

>Tb11.NT.20_1 [96 - 188]

MFISRVYENDLSSRNELCSATGFTREAPHHL

>Tb11.NT.20_2 [203 - 322]

MKLRGNAREASMIFPSFTTFGVWRFSLRWGYPLHTKIQWF

>Tb11.NT.20_3 [70 - 486]

MSFFQLDWECSYQEYMKMIYHHATNYVPPRVSQERHLITCRELLNEAQRECKGGKYDLSFFHYLRCMEILTKVGIPSTYKDSMVLKKQCMDAIEALMNSALKNHYEKMVKELKSKKEGVDAVDYKLYGDRLAHVSGGNS

>Tb11.NT.20_4 [428 - 646]

MRLTINCMATDLPTYLGETAKDGYRICRLMNPHGQGVGNAKSKSTKTAKSCVTRVSSGVALIFTLRTTDPEYA

>Tb11.NT.20_5 [505 - 735]

MSFDESTWARRWECKEQKHKDCEKLCYEGQQRGRAHFYSTHNRPRICLAKRQVPWWICAPMNFLLISRSLLQKMQLS

>Tb11.NT.20_6 [525 - 893]

MGKALGMQRAKAQRLRKVVLRGSAAGSRSFLLYAQPTPNMLSETSGAVVDMCTDEFSIDIKEPSPEDAIVVASLTLRKKKMQKWREEKRTAAKPPKRAQSGASTCTLNFNKQETRWPLKISEC

>Tb11.NT.20_7 [890 - 973]

MLNCSGHRIVLTHPKVFLFLGGGLYILN

>Tb11.NT.20_8 [976 - 1068]

MLNVVSSSQCAAAPQQIQSQNELHIKAKASY

>Tb11.NT.20_9 [1078 - 1185]

MPTLIVARGVPWLLFEGETIRARTTDPIYIYIYMYI

>Tb11.NT.20_10 [1522 - 1596]

MVINQTSVGEEFKRIAQTPKRSNYM

>Tb11.NT.20_11 [1611 - 1790]

MRKETLESHRYRKWVMTTDGEINASLSVGVGTRLSWSKSREAIMSAPTVDPVLSAMYQNL

>Tb11.NT.20_12 [1874 - 1960]

MPRSYAKQRPNKPEKLSKERNVDEGASLS

>Tb11.NT.20_13 [2172 - 2363]

MGSRTEHLELKLLAAPHHVEVNTKKYIFTTPKRAAFYNIQRRTSKIYKTVRALTVQARKPQDDG

>Tb11.NT.20_14 [2359 - 2457]

MVDAKPRSIWIRTLIITKFSNRFRSSELNKTNE

>Tb11.NT.20_15 [2453 - 2620]

MNKHIYTTSPFICFPHVSICFCFLYATILELLPMCHCRMKKCSLEQMHEFAYKYHT

>Tb11.NT.20_16 [2595 - 2675]

MSLHINITHNLFHITSVCNIMENMERK

>Tb11.NT.20_17 [2683 - 2787]

MNMRRSCKRFVLLLCLLVSKTPSQLFSMKQIVVAV

>Tb11.NT.20_18 [2791 - 3000]

MRKKIHSPLCLFQQQFILFQNASHTIPEKNYLFFLSVLTFLLRNVLCRIGSLKQLKGPPHTHGMASVLPS

>Tb11.NT.20_19 [3003 - 3131]

MIVSFLRGLWRDILRFSSFFQFSRYWSPFPLPSFFQINYKGIK

>Tb11.NT.20_20 [3284 - 3388]

MNVELTCADMILQRQQFRLTFVCMFNFYNWRNIHY

>Tb11.NT.20_21 [3301 - 3423]

MRRYDTAKTTISANICVHVQLLQLEKYSLLKFSHSCNNSNA

>Tb11.NT.20_22 [3450 - 3536]

MHDTTRGVNSSNGRILDTCGNELTQLAIF

>Tb11.NT.21_1 [1 - 147]

MHRQAHTHTHAGWSAPFSGEIDMKCKRERIYANVCRWGGGGDANASFYM

>Tb11.NT.21_2 [92 - 235]

MPMSAAGGGGVTRTLHFICEGCFGPYFRLNGVPHRVRQLRLPPVTFVY

>Tb11.NT.21_3 [437 - 574]

MPASRSFSGATHSRYNGLNAYPFKPPASKKKVSFIIRRVLPQRGNT

>Tb11.NT.21_4 [786 - 1013]

MEVNLLAVKKVVEVPLTCLPRGGGDGWVRWAFQKLTLCDASLNRGMKMGIDEFCTLLGNGYGLGSSSHSPLKNKTE

>Tb11.NT.21_5 [937 - 1209]

MSFVHYLAMGMGLAVLLTAPSKTKRSKQHFIGECRVDMHCSSYVCVGSGLIMMGRPGKELNVIPGLAHSLFSYEMELTVTQFGRGEFRGKL

>Tb11.NT.21_6 [1336 - 1443]

MVSDANTPNLFGAALWQPKGYQSMCGKWRRQRRQDS

>Tb11.NT.21_7 [1346 - 1468]

MPIRQICLAPHCGNRRGTNLCVANGGGSDDKIHKAKSCAIT

>Tb11.NT.21_8 [1311 - 1517]

MRFHESWKNGERCQYAKSVWRRIVATEGVPIYVWQMAAAATTRFIKPRVVLSPEQNSQETPTAFHSRPI

>Tb11.NT.21_9 [1527 - 1619]

MDYGIISTVLRFHFKRGESEKQGSSVGIHDC

>Tb11.NT.21_10 [1534 - 1659]

MVLFLRFSVSISSGEKAKSKEAQSAYTIAEISSHANKQDMLK

>Tb11.NT.21_11 [1792 - 1869]

MAEYSGGLVLRRRKSGCTPHLKLLGC

>Tb11.NT.21_12 [2060 - 2137]

MEKYRIKGTSEYWDAVAAWKQRQQQC

>Tb11.NT.21_13 [2100 - 2246]

MQLPRGSSANSNVENGTENAKRQTCTEEGETHQGDENYFFLVLPAPPNT

>Tb11.NT.21_14 [2134 - 2316]

MLKTVPKTLKDKPARKRVKLTRVTRTTFSWFCPRLQTHKVLLRAHPPQQLQNSVNAMLPLE

>Tb11.NT.21_15 [2375 - 2467]

MRQPFVNHRSKLHSVLRDPSQPLFRVSVLTS

>Tb11.NT.22_1 [22 - 243]

MCCVRCVPDMPFRMRRPHAFCPEVKTCAVEIGPGRHDNSHTLLPSGVCIRPAKSFSHVFRVAQDTFYVMDQGSR

>Tb11.NT.23_1 [469 - 576]

MLRLCHRLMLCPRGVATDLLGVLHMLLCGQFVVPKQ

>Tb11.NT.23_2 [498 - 704]

MPTGCGNRFAGCSSHASLWSVCCTKTVTEARPGQLITLKLLLCNTVGHESELYYSFPWGSTADHSFIAG

>Tb11.NT.24_1 [378 - 527]

MKGSCFVSLHLLRHRSVVTVFCFLFRCYFLSLSHHGPFELFSVLLLRSEK

>Tb11.NT.24_2 [455 - 544]

MLLPFVVAPWTIRIIFGSFVAFGKVGSADC

>Tb11.NT.24_3 [547 - 657]

MCLGFCVLARMHMNLGRISVGKWAFAGTLNLVVDWCE

>Tb11.NT.24_4 [668 - 748]

MRLLLIFLCVTGRAAEGNGVRRLGRLW

>Tb11.NT.25_1 [109 - 186]

MQWEAPLRSVFFPLFFSLLTVVWCFR

>Tb11.NT.25_2 [114 - 215]

MGGSTTQCIFPPVFLIVDGCLVFSVICFGDHTDN

>Tb11.NT.25_3 [488 - 562]

MCIYPGKNECCGILLLTHLVLHVVT

>Tb11.NT.25_4 [514 - 624]

MLWDIAIDPSGVTRSYLMSFIFGILLPLTSGGWLVGG

>Tb11.NT.26_1 [231 - 470]

MRATVSALLSYGGGVFMRARDGIVCGSNAALISRSVGACCVINVLPPKKYLINYLALWEVYGGAKEPRQHSWMQRRQEIP

>Tb11.NT.28_1 [105 - 272]

MFPGMQQPKALPMGEAHTQFWTNFNRFLTTSALSITVSLFIRYCEGRTSAVLLNGI

>Tb11.NT.28_2 [311 - 406]

MFLVVLEYTGGRDVVEVKGFSFFFSKKIKKKK

>Tb11.NT.28_3 [704 - 799]

MTLSHFVVFVLKTHSIFRGLKVSNCTSESPQL

>Tb11.NT.28_4 [898 - 1173]

MNVCVCGGGGGGGVCATTQITSSTSRAVDKEILTAAAYSSEFVLSPCFIILYQTFVDTKWNKIIKQGKERKNTRGTMFRMFLFSSFLFLRCC

>Tb11.NT.28_5 [1188 - 1286]

MQFSALFRFVPPFCCPVISISFFVLFCFFVSAF

>Tb11.NT.29_1 [145 - 330]

MLLGGFVPRRFSQFNRDPCWMFFIFSVGFWLGEYPAMMIKYNARDLVYDPHRYVWSHHDDHH

>Tb11.NT.29_2 [269 - 391]

MPVTSCMTRTGTYGPTMTTITKPSCVQGGKEEKKNKQTDKL

>Tb11.NT.29_3 [444 - 611]

MRIHIYIYIYMCVIAFVCLGKWYFVSLKCRMDGVIEVCRFGVEGQKGTRREKKPLE

>Tb11.NT.29_4 [648 - 752]

MRTNATVAVIFIASISHCGLFLCCLCARPSCALLW

>Tb11.NT.30_1 [314 - 409]

MWKSTAFNHRHVMLGLVVWCTGTAYSVEGDTN

>Tb11.NT.30_2 [435 - 581]

MRCLGIRFFISFVYVVNFWFDAPGCSNTLSAPHPSCLCCQVRIGSVALC

>Tb11.NT.32_1 [220 - 534]

MLTTGPLNIPRAERLCRIYFYSGFAGLPLLWFTTWLFFRHHAQHSAAIRWYTNTSLRLSVVGGLVVLLWYVVALIVLPVTSPLFALPPSQKGEWRPGFFTELVDE

>Tb11.NT.32_2 [530 - 613]

MNDIAVNKQILRLLGARAFCCAALNMPI

>Tb11.NT.34_1 [250 - 432]

MPLIKKVNITPKSVYFLNPDILDTFYPFAVARAVDCLKIDAEAPLLPCLRKLNWSLRALFW

>Tb11.NT.34_2 [368 - 475]

MLRHRFYHVCASSTGLYGPCFGSNGGAEDGDCAFLK

>Tb11.NT.34_3 [390 - 506]

MFAQAQLVSTGLVLVAMVVRKTATALFLSDEKCRGAALA

>Tb11.NT.34_4 [588 - 764]

MMLYGVPSQLLLRCFFRLRRLLLTFPTTLSFLCNVFAEFSLSNCGYLCRYVCDDLSSGL

>Tb11.NT.35_1 [173 - 280]

MVKRFRCRRSTSTSSTVTCVHSGLRGFTDAMTVYRG

>Tb11.NT.35_2 [280 - 360]

MTGAVFQYRLMQMGHCQWGYNDFYNKS

>Tb11.NT.35_3 [386 - 523]

MPILFGFSLGDPSVPCRSLHTVPVKVVAARLTVHPLVTLVQTLYFS

>Tb11.NT.35_4 [747 - 842]

MGTFRTLGATVLVAALHRTHLRIDLYTFLYLS

>Tb11.NT.35_5 [1004 - 1078]

MCLCEQGKAPASCAQTCKKFFAGAA

>Tb11.NT.35_6 [924 - 1151]

MCNCSGSCIKRQLRNINHCSASEMACECVCVNKVKRPPVVPRRVRNFSRVLHSWQGYFFFLIFFSDAFPSTDGKST

>Tb11.NT.35_7 [1120 - 1266]

MHSLPQMGNLRSGANIAPVTGVFIFIFFNVRLLAPPPPNSSSGGAKYEM

>Tb11.NT.35_8 [1333 - 1473]

MLRGLLWRSMSQGRSCRKKKVIINEGWKPISIAYITVGASSPELEGS

>Tb11.NT.35_9 [1403 - 1543]

MRVGNLYPLLTLRWVRRPQNWRGAEKIFSKGGILESDNGHFLLNFYS

>Tb11.NT.36_1 [6 - 104]

MTMLSRGHCIGKQYSETLAMALFIKKKKYGTCG

>Tb11.NT.36_2 [169 - 288]

MFYFLCHFRYNTYRSLHMGIHVSSHVSQKVQISRWALLNQ

>Tb11.NT.36_3 [183 - 359]

MPLSIQYLSVVAYGDPRFVTCFSKGSNFSLGPFESMKDIGLGELTSYTYYLMWDCLAFE

>Tb11.NT.36_4 [602 - 835]

MIITATIVRSFRFDKVGREKLKRFECLHSPSRCVPTKTSLTIWGGILKRSSSKFVCEKLLYIILYFFLKKKSHISKYW

>Tb11.NT.37_1 [85 - 216]

MMHTMHLVSLNVNFVVPKVYRFVSQRHYISYTTVVHEARQCRWK

>Tb11.NT.37_2 [1162 - 1332]

MLVVGACGLLLFLFLSVAARNGSFCWVVLMLRWSPEGIWDNMRHKLSGYHNSFCVLF

>Tb11.NT.37_3 [1275 - 1361]

MGQYASQVIGLSQQLLCSFLKILLTATRA

>Tb11.NT.38_1 [382 - 492]

MYPIYAPVRNTGEKGYELTLFAGVNVTHIVVILTALC

>Tb11.NT.38_2 [513 - 611]

MISFTLRVPAKELLFHCCRLKLLLTDFVAALFI

>Tb11.NT.38_3 [881 - 1045]

MSPHILFAVAGFTPTSYCFTPRAATTFCLLSLVCGVGLDGTFPFRTPFFLGGGCI

>Tb11.NT.38_4 [715 - 1164]

MARSGLWIFLYSSDAHSYCLNCLSLLTTRAHTYGIHLTCHKFLFSVYSSRNVVPRDVSTYPFCGGRVYSDFLLFYTPSCNDFLFTFIGVRCGSRRDISLPYPLFFGGGVYLNPTSNFVGDQSRVQRETIFSYSFANICYDQECRSSNFTR

>Tb11.NT.38_5 [1202 - 1498]

MGSHVVIANHLPRFILIYRTGTVTELVIVNCCGGGAVYWARSFLLFFLNILLYYLRPFILNIGVACIFIRLEDPIEVVLIMVRCADISSIGIWNILCNF

>Tb11.NT.39_1 [70 - 183]

MHVSPTGRQGSRPLRVMHCCVVWRGSFAASPFMFFHEA

>Tb11.NT.39_2 [436 - 537]

MLFDCLLLHINVYYVVRRFSNVAVEDQYTYEGGK

>Tb11.NT.39_3 [467 - 556]

MYITWFAAFRMLRWRTNIPMKGGNEHLVIQ

>Tb11.NT.40_1 [168 - 383]

MGEWKVYIGAICDGLCENITSFLAGALRGQMFFFLFPWASSHQLQVVLDAAVEILARMLTCRIPPIAQYVDL

>Tb11.NT.41_1 [37 - 135]

MGLPLGFIAGAVPLAPAVEENRTKGLKCSRARE

>Tb11.NT.41_2 [202 - 279]

MRSTHRETRDVAAGFLSELTGKWKGK

>Tb11.NT.42_1 [65 - 268]

MKHYEAQKRWELVTGLHVKGKVCYFTEGIENQGIGEKQPLFMGIGWRGPFEVTSTAGQCRAAERGPVH

>Tb11.NT.42_2 [272 - 367]

MALGFASCWSSLGSRYRIPTHHHNPTRNQTQK

>Tb11.NT.42_3 [447 - 593]

MLSKSRVRSLQVKGELPVIHIALAVSTHTRTRRKKNRTLRRHGQKPVLL

>Tb11.NT.42_4 [571 - 774]

MGRSRCCCKFSVSMHNDRACGHLYIPFFPPRFCCTDSAVVAQLIQMAFRKVRLATVPFPPSNFATCDI

>Tb11.NT.42_5 [617 - 811]

MIGRVDICIFLFFPHAFAAQIALWWHSSFKWLSGRLGWPRYRFPPPISPPVTFKRADGGLSVLLP

>Tb11.NT.42_6 [906 - 980]

MALVHHPTILFHFVPFRLGALQWPR

>Tb11.NT.42_7 [889 - 1014]

MRHLSAWRWCIIQPSFSTSFLSAWGLCNGRVSSKMKFPGVLP

>Tb11.NT.43_1 [44 - 145]

MVVSQPILLKSYGSYFLVKYFFCLIQEYRPVRGM

>Tb11.NT.43_2 [160 - 234]

MRSPLFRLRDIVEVRVSQNVFEPSM

>Tb11.NT.43_3 [560 - 637]

MFETRGDAVVFPRAACKKKKKDGEVQ

>Tb11.NT.43_4 [474 - 689]

MMLNIGKLLCPRLTQGVQPTMIPLPMTNACSKQEGMRSFFRGRRAKKKKRTAKCSNNKTLYKNNATLSDTCC

>Tb11.NT.43_5 [556 - 726]

MHVRNKRGCGRFSAGGVQKKKKGRRSAVTTKHFIKIMPLSATRAANRDGATPWIIIR

>Tb11.NT.43_6 [1138 - 1341]

MRSSLLDTCYCVGLEFGGLLFRFFEYFHCGPYKSFGQLYVEGCLKIFSLRYFFVLFTTAIILFPHFTS

>Tb11.NT.43_7 [1465 - 1545]

MCCDVSIVLVFTLVIYLNIIHYIYIHI

>Tb11.NT.44_1 [142 - 270]

MNLGGHILTKIKILFMELRYVIFVLFRSGYYQMTETLLYCHFA

>Tb11.NT.45_1 [72 - 158]

MVLLDMVFRSFWRTYCICPCCCTVVFAFR

>Tb11.NT.45_2 [17 - 277]

MYVVVVVLAALLRSCWLTNGFTGYGVPKFLEDLLHMSMLLYRCVCLSMNSRGFACSCFCFLLLLCGNGGCVYDGDIHIYVIRMVQNR

>Tb11.NT.46_1 [66 - 152]

MVLLDMVFRSFWRTYCICPCCCTVVFAFR

>Tb11.NT.46_2 [11 - 265]

MYVVVVVLAALLRSCWLTNGFTGYGVPKFLEDLLHMSMLLYRCVCLSMNSRGFACSCFCFLLLLCGNCGCVYDGDIHKICDKDGA

>Tb11.NT.47_1 [243 - 455]

MIIPVRCFTCGNVIADKYLLYLDLVAEGSSEEAALDAFNLWRFCCRRMFLTHVDFSDLLLKYNPADSSVAV

>Tb11.NT.47_2 [617 - 694]

MCVRVLCITFHEILAVCGTCSTLQFY

>Tb11.NT.47_3 [746 - 889]

MLRFIFSCFFFKKRHMHEKKKENGVGRGVKVYVLSTRVCIFCVLSPHF

>Tb11.NT.48_1 [209 - 337]

MVYLSFILPHLYFLAPYKFSCDVLSLLPPLFFVWGRKEGGTGE

>Tb11.NT.48_2 [126 - 575]

MIKCLFFEVKAKKKTTTTVGLQVRESFGWCICRSSFPIYIFWHPTNSHVMCCHCYPLSFSYGVGRRVELESELISRVGSDCKTGMIVIGNADAEMNENNYDVVCMCRHMSIYSYIYIYIYIYIYIYECSSAGRACAATITVVVFVALPCV

>Tb11.NT.48_3 [506 - 592]

MFLSGPRLRCYNYCCGVCSLTMRVRYTDG

>Tb11.NT.49_1 [87 - 176]

MKERVEVPWNVSWVPATAVRSAHGNIYIYI

>Tb11.NT.49_2 [461 - 592]

MGNNALHRLRHHTAQGSAASCWDTVFHLSVPGHVPACHQQYHPH

>Tb11.NT.49_3 [321 - 731]

MPYARGKLLSVGFLRKESLAMPATSTWCQGPVPRIIGGSQEPAAFLSWGTMLCTGYGIIQHRDQQRLAGTPFFICRSLGTCQRAISSIIRTKMLLSGDVDLRKKKGLPIDIFGRVRGGRLLAPSAVFRSYAEIQQKL

>Tb11.NT.49_4 [115 - 816]

MYPGYPPPLLGRHTEIYIYIYELGRFITCCGFVPYLVVKVYFPLIIRRDLRDLVEGCAVFLYTIWCSAVCRMPGASCLALASYERSPWRCRPPQRGARVQYPVSSGEAKSQQRSFHGEQCFAPATASYSTGISSVLLGHRFSFVGPWARASVPSAVSSALRCCCPVMWTSEKKKDCQLTSLGESGAGGFSPHLLYSVHMRKYNKNYRGCVRMNEKGRLCHSRQTDSISGLYGDG

>Tb11.NT.49_5 [752 - 907]

MKKGDSATVARPIASQGSTVMADGRASGGKLPRRHEENSKKKNTKGVLFSSP

>Tb11.NT.49_6 [819 - 911]

MAAPVGGNSHEGTKKILKKKIRKEYYFPLPE

>Tb11.NT.49_7 [955 - 1041]

MRVLVGFISYYEAVWRVCVGLALQVTHLV

>Tb11.NT.49_8 [1164 - 1253]

MPLVRLNFKFGCLGSFFVNLNLVSTVGIHV

>Tb11.NT.49_9 [1256 - 1330]

MILFDSHHYRMWSGGGGVNVPCSFP

>Tb11.NT.49_10 [1311 - 1409]

MCRVLFRDVDSFFASRSKHNLLLPCIRCLAVCR

>Tb11.NT.50_1 [35 - 238]

MYINMYIFPPTFFFNFFAEPVHTQTININRLHALTSRDPLRIITSDQFTPILGVNISHTSPSLYSFLA

>Tb11.NT.51_1 [55 - 153]

MHLHINKRPTFCECKKRKTQANTHTEKTNAYRI

>Tb11.NT.51_2 [45 - 182]

MQTYASTHQQAPHVLRMQETQNASKHTHRKNKCIPNLNQIHTYMGC

>Tb11.NT.51_3 [140 - 214]

MHTESESNPHVYGLLTIEKAKGRKC

>Tb11.NT.51_4 [271 - 438]

MGRTYEDVARHLRITVRGVPMNIYVYIYTYISNLFHRATERWSTKESQCGPASVDS

>Tb11.NT.51_5 [341 - 454]

MYIYIHIYRTFFIARLRGGARRKVNVDLHQLTHEENAA

>Tb11.NT.51_6 [571 - 765]

MLPLNYKPWSFILLCSLPSAYSNALGYSLVLLSLRWLPRARASIYSAHLSPSAVTERGKKREIGG

>Tb11.NT.52_1 [134 - 235]

MHAHIVTVLDSTAPACSTWSASLIYIVSLSSFSL

>Tb11.NT.52_2 [295 - 483]

MFGSWCLAAVTKFYKSYGTAPLQRVPKKSYDQGLFKLYLKLALPSTYSKTLLVKCGGGRTHTL

>Tb11.NT.53_1 [210 - 299]

MVVCRKLRGFGGGGNIYIYMSRCVIKRCSK

>Tb11.NT.54_1 [271 - 423]

MWKSENNSSCRNTNINCNGPNDGHYEACTVCFIERGRRREIVKPRYFFLLS

>Tb11.NT.55_1 [197 - 343]

MLGSITTQHIFFLPVELLGGWKKGNCALTVYLFFKNFPLLYSCVIAVLS

>Tb11.NT.55_2 [478 - 579]

MKVLKGRELDLQGSVVCGESATGFNSVKRRVCLR

>Tb11.NT.55_3 [635 - 814]

MTSAAKQVVLKIREPEVTISELVFNGLGIGCRWRYYVSAGTRPLVGSVSKYRSEKSVESG

>Tb11.NT.56_1 [220 - 369]

MCSDTHMFRTPEYSGVWEWFRPKKCDERGPKDVHVSMFELMQLISYFLPQ

>Tb11.NT.56_2 [460 - 537]

MGLLKGAADTCPCVDTFVRFKNSTMW

>Tb11.NT.57_1 [327 - 404]

MRMGLRRALVDAGSRLSTTKTTCSYS

>Tb11.NT.57_2 [516 - 731]

MMWMSKADLHRCALRATPRGRWLVMCVPVLWMNGWELPRIRQMNSPTVTREKHSRQIQCLQPMIRVPLCLIR

>Tb11.NT.57_3 [65 - 841]

MRLHTVRAPIITRAAMRGYSEARSNYDGTSLPAWPAPGKKPTYPAALSELRLPQPRMRKTRTEWMYYHGHGGCPGKYGPSREIADFEYADGTPASISGRRFAFKHHQDHLLVQLIRAAATVERYDASGLLPRIPGTAEQRNWDPAIPLFLDDVDEQGRPAPLRTAGDAPGTMVSHVCSRVVDERMGTPTHTPNELANRHEGETLEANTMFATNDPSAFVSDTVKLRDDKRPYWSRRRWALTDKFLVPKSPKPKNTIKDE

>Tb11.NT.57_4 [1056 - 1193]

MGRAVVCRWLRLRYYRRDSLYGQAVFFSSLSLVVVVVVYVNTTIVS

>Tb11.NT.58_1 [90 - 257]

MAPASIFQRGVEEGKGGKGLSAFFYFYFCHIYRTRINETVPGFRSDVRLLKVYCGD

>Tb11.NT.58_2 [199 - 273]

MKLFLDSVPMCVYLKFTVEIKEMKM

>Tb11.NT.58_3 [337 - 420]

MLWSINVITHVVACFGDGPFHFDLYVVT

>Tb11.NT.58_4 [432 - 587]

MCAIFCCCLPTVRVSARLSLQQIRVSGENGTGGSPPKRWIFLKQLFPPPLAY

>Tb11.NT.58_5 [545 - 715]

MDFFKTAFSPPPRILSGRKSVIQLTWCRVEIVPTCAIFLSFFSSRRMSGARRCPSGC

>Tb11.NT.58_6 [619 - 726]

MVQSRDCSHLCHFSVFFFLASDERCSPVSQRVLTEA

>Tb11.NT.59_1 [74 - 184]

MSWLECNNGWSGEGAYWIPSWSSPAQTSAETPRKSHC

>Tb11.NT.59_2 [100 - 189]

MVGGGGILDPFLVIASTNIRRNTAEEPLLR

>Tb11.NT.59_3 [210 - 287]

MSWLPMGLHWFKKKKENTERQLHRAL

>Tb11.NT.59_4 [319 - 615]

MLRCIHAFSSAGCVCVCICMCALLLETVNILRITFDITHIPFSYSLKKVMTISFMQIIMKFHNYRYRFSIFLSRNCFNLFCHCIQKYTSHDSVPHLTTF

>Tb11.NT.61_1 [368 - 460]

MMLLSSSFFFFERVLWLSSVFYSVGLMLFLG

>Tb11.NT.61_2 [584 - 685]

MCGCVPSLTGDRVSCESLYYSSAQRFCRVNAAGI

>Tb11.NT.61_3 [717 - 860]

MFAAKVVRSVVPLLCCVSKGSTALVAGVGSNESVHAFPCSRLWRMGCV

>Tb11.NT.61_4 [808 - 1038]

MSPCMLSLVRVCGVWVVCKSLFFSVNHREEHVDIIFGLFRSFVTGGQLQVVQKYQNEWCNAEADDSTAAPYDAVSKG

>Tb11.NT.61_5 [974 - 1084]

MSGVMPRRMIPLRHHTMLSQKVEASFHSVFGGGVLLC

>Tb11.NT.61_6 [1141 - 1221]

MIALEVSVYTLMGERSTFLYVRRRLWC

>Tb11.NT.61_7 [1462 - 1599]

MDLMFSSNHSDWTHCCIGRRRICRSFCFNIYLAVRGILRITCKQKC

>Tb11.NT.62_1 [210 - 323]

MPSCRLREVPPLPETESKYVGDTLIEIHYFIFCGVDSG

>Tb11.NT.62_2 [349 - 600]

MREGFIKCIGCCCTTYFWLSCLLGLEGTRQCFIERLHPPSIGRRSKISQDLWRSSHTAYAFLKPCGVAVRLRCASDASRAINAE

>Tb11.NT.62_3 [524 - 637]

MRFLNRVGSPYACAVLQMPAEPSTQNEEEMKELRWRSP

>Tb11.NT.62_4 [625 - 747]

MAVSVMVCVCVCVERSPATCVTAAARHVCLGPSGSSTGIIQ

>Tb11.NT.62_5 [756 - 914]

MRCYSPYRFLFCWITVLRGLTHLSKPFRNRALPIKKKERDRETVIYTVKQIGV

>Tb11.NT.62_6 [1057 - 1146]

MLAFKRPKIQKLALSNRFSRLGRVCGCAVE

>Tb11.NT.62_7 [1233 - 1340]

MLWFCILTHTMPFTPRRCFTTVCMTRKASGLCLLPL

>Tb11.NT.63_1 [365 - 475]

MCHLVLFLIRWSERSSVGSTSLLPLAVNLNGSVTPQQ

>Tb11.NT.63_2 [497 - 595]

MRCIMSFVHIALLLCTLFNIKVRINSVSSCIRI

>Tb11.NT.64_1 [323 - 436]

MDARIYSQWVKNDLTSQVWRLWSLFDLGRKLSVLFLHF

>Tb11.NT.65_1 [205 - 294]

MRCGWVNIWDRCSVAATFKFRSGRVIRAFF

>Tb11.NT.66_1 [83 - 364]

MFRQRLLFSAVKPSRNLLRDCGREKRLMESEGDFVDRHLASLVAEREKAYAGLFGGSLAATCLTFVSMGLFGLTFGYHAGKAVIDAYGKTRISA

>Tb11.NT.66_2 [342 - 434]

MEKREYRLKDDGKQSRNNFSPPCESSGWAYH

>Tb11.NT.66_3 [370 - 474]

MMESKVGIISLLPVKAPVGHTTKRKFIVLRSYIYY

>Tb11.NT.67_1 [97 - 180]

MFKLGKLSDFTELALTIRMRHNLVVYIT

>Tb11.NT.67_2 [347 - 466]

MHKGKGMNMCCKMCNAQSHAPKMHSGILATNEKISLRGCI

>Tb11.NT.67_3 [594 - 743]

MTGSRTSREVNHLRDNETVYLPLDGCFSFCSHEQQLLYLRTEIKRGSGDQ

>Tb11.NT.67_4 [664 - 855]

MGAFPFVLMSSSFYISVRKSKEAVETNDKLKGDTTVNAKLRRFLLANMFLKNQTYINVMRRIYV

>Tb11.NT.67_5 [858 - 947]

MSRQNLCLTRLLIGLHDDSHTVSRLVLNDY

>Tb11.NT.68_1 [208 - 297]

MCVLCDRRCAAQEKRRDECPILIFFTDEYR

>Tb11.NT.69_1 [10 - 111]

MKEIITLREFNWGWIVFHDNVHRAHAHTLSVLTI

>Tb11.NT.69_2 [354 - 455]

MNRTSPFNRETGYLFPFGTSDAPSVRSGKRLQGR

>Tb11.NT.69_3 [1618 - 1710]

MGSTLNFSFTSIPHMSECSSKAQIFLPACLC

>Tb11.NT.69_4 [1707 - 1781]

MLVLGMLWACDTLFVRGRRQANNWD

>Tb11.NT.69_5 [1833 - 2021]

MQLLVLLNICTGFTSIREAENCFSYPFRAGACHGLVSTHQVVILYLYTQRFSMWFLGKKNFFL

>Tb11.NT.70_1 [84 - 236]

MNCVPETPGKKTFKEHVVFRVVSCGGQIAFFNAMQWYLPPCLCARDAIPET

>Tb11.NT.70_2 [220 - 459]

MQFLRRDRTDVKVVLLLTMTHMIFPPQVKIKKRGNTSLSITRSVETTFTTVMCLTMCSPQRSIWKRIIFNLLPTFATTAN

>Tb11.NT.70_3 [565 - 660]

MSSCGNSAWITKIKSFLISLQKDQRRVGSIKL

>Tb11.NT.70_4 [695 - 940]

MTLRRVVCILLLTKFFRFVVNKCEHNQLEDAVSEAVYLIDCEIMFFFTVADKNLFYWLVIVTLWCSPFVYSNVPRRAAFAFC

>Tb11.NT.70_5 [1000 - 1089]

MYAFFWTRRNSELLTRLRLIVPLSQRLVLR

>Tb11.NT.71_1 [232 - 324]

MGVIVTVEGNKPPSFPPSLFLHEISKHEEDT

>Tb11.NT.71_2 [296 - 415]

MRYPNMKKILRGDHSKAPGSVRYRVRCFNRFNCSFFLLPL

>Tb11.NT.72_1 [346 - 486]

MLSSGDFKAPSMRSPGVTLAGGVRCGVLTVLRPDDNKSPLFGAVQDL

>Tb11.NT.72_2 [941 - 1111]

MHSVQTLAPCPLCHSIRKLLDTTVREDTFPCLLLRAGGGLLVPFCAVPWATGEMIVR

>Tb11.NT.73_1 [173 - 418]

MQAATGGVHPLRRAMHRNLLLLMRSGPPTGINEGELTLTLTERLKERQKRSRKKQTQPRLILSLTIHLLSQPFKFLCDRVLV

>Tb11.NT.74_1 [157 - 240]

MYSFAPLHVCAMPLSPYTVLVRFPLLAF

>Tb11.NT.74_2 [135 - 299]

MRRCVGMYVFICAFACVRDALESVYCIGAFSPFSLLDIPCGEGKVVGVDSHMLDG

>Tb11.NT.74_3 [299 - 382]

MNCSLVKVDACHYRRGCVDNVALWYLFM

>Tb11.NT.75_1 [99 - 176]

MLPSSCHVGVLVLPRELLRFRTRLFC

>Tb11.NT.75_2 [118 - 375]

MWECLCCLVSSCDSEHGFFVRAAHNVSCTVRGMGDGVLGRFVPRFREAKQTPLCLLPTDRYLASSRSWCHAAASVTVHSGIIIILN

>Tb11.NT.76_1 [73 - 234]

MSRNPSLYPRDGVTAVEQILFDNSLPSLNVLNPFRWYGLCQNSELFVCLQRCAV

>Tb11.NT.76_2 [225 - 308]

MCGIVPCKTALVSNMCVCVSSYRLNQRV

>Tb11.NT.77_1 [36 - 182]

MMANRKRILSKSFPLVKYWNEDSFDLIYRRNDLLTTDAFWINSVGAISY

>Tb11.NT.77_2 [479 - 565]

MSCEEYCLGAISFAEAKDNLECKKNSEIC

>Tb11.NT.77_3 [348 - 659]

MVKPCICGRSYLFSVERPRYIVSDDGMACLTFLGVKYRAYCAELCHVRNIVLERLVSPKQRTTSNVKKILKFAKLPLWLPSVDLRVYNLITPTGRWCCVKMTSG

>Tb11.NT.77_4 [762 - 908]

MCSRVVMYCNVRTEVPMDSRCWYLRFVTNTLLEGRRQYPSFYLRCRHYG

>Tb11.NT.77_5 [893 - 970]

MPALWLTHVSVDGLQFCSSTGLGSYP

>Tb11.NT.77_6 [904 - 1170]

MVDARFRRWVTVLLLDRFGVISIVLITDCIPNLLSHTHKDTRNMKTYFVWKQIEISFDFFSARIAVTLWIDGSFFHLLLHYMKHADDVS

>Tb11.NT.77_7 [1154 - 1282]

MLMMCRKGRNGAHNVLLLYLRTRMRREQNYPLYHIIGVTAFPL

>Tb11.NT.78_1 [146 - 349]

MRHVCVCVCVCVCVCVCRSLASLFQYCFLMSYSPARFFFFLNKRVQHRINSCRRHSLWLTRLVALVSM

>Tb11.NT.78_2 [563 - 670]

MLWRYRGACSFYSSFCCAWFLNYWFCLTYTGLFVPA

>Tb11.NT.79_1 [28 - 390]

MSFSLSVGVARSGSYERDVLITRANVLSGYRSVMRPHRIKLHSLAGSGSPDTFAEGEIGWHLRFWNEGWRRPHKIDQPWWFADRMRLPCEVRRDIGSLDVALCFFAGRLGDWLPFSPRERR

>Tb11.NT.79_2 [224 - 664]

MRVGGVLIKSINRGGSPIVCGYRVKCVVISGPSMWLCVFLRGGWGTGCPFHLGNAVEPGRQHDTALRPAASCRNAVIYLPVPRAPPILPLVALNEDPSKKPGGGVNVLLWHLTGGLVASAPGPVVRGKGPLSYLGAPYVFFSDASFV

>Tb11.NT.79_3 [787 - 927]

MLAGSGWEERLMVQWARYQRSLCLRSMRVEVSCYAWWMRNMHGDVSH

>Tb11.NT.79_4 [911 - 997]

MAMCRIKSTSSFQRFVHLFDFSGPLPETR

>Tb11.NT.79_5 [1066 - 1200]

MSRCGFARVVPFFFCCTLPDSVSCDGGKKTPGRLQQIFFAHISSY

>Tb11.NT.79_6 [828 - 1259]

MGPLSAFFVFTEYARRSLMLRVVDAKHAWRCVALKARVRSSGSFIYSISPDPSRRRVRLRQRVDGSDGICDLFTATAYLNEPLRVRPCGSIFFLLYAAGLGVVRWWKENTGAPSADFFCTYFFVLVESSVSESCFRLSLFFPES

>Tb11.NT.80_1 [11 - 151]

MRCILEAGRGGLEVYGRIGDQICGSVLRGANTCDRVLFIPLRRGGMC

>Tb11.NT.80_2 [325 - 447]

MRPRRPSEDRPLFPPPEEEGICVTLLNGAVPNRIGAHWLEV

>Tb11.NT.80_3 [309 - 464]

MKEPLHAPPKAFRGSASLPTARGRGHLCNSPERRRAKSYRCTLAGSVVIATR

>Tb11.NT.80_4 [636 - 836]

MLPLLRLALPKRGGSNPLLRCENRDKYTIPASRASIHFCNAVRWFAFFTFYQFVRACLTFVSTRGIL

>Tb11.NT.81_1 [236 - 349]

MRDWCACFAFLKLVKFAEDYLFFSFFFTFLWSLSDHHS

>Tb11.NT.82_1 [86 - 196]

MRSHLCLTCLMFSAHMNTFTSVDMYLRNECVCVCVCS

>Tb11.NT.82_2 [168 - 254]

MSVCVSVCVAETHRNARWGHSEVKWCLIN

>Tb11.NT.83_1 [63 - 170]

MLQLSIPCSLPYLRKCQRFKYRITRTDCGGAVFYEQ

>Tb11.NT.83_2 [228 - 314]

MTPPPHVKAVCSLKSCINCGAGAPIAGGR

>Tb11.NT.83_3 [486 - 614]

MAVSVAFPLPWVIFILEVVCWLRCGARPSAIRYKRLICRLELF

>Tb11.NT.84_1 [115 - 225]

MRDHVSSQLPSFPSSLLICQHRCSILDCRVVTLTPFC

>Tb11.NT.85_1 [126 - 305]

MRQFEDHRLDCGFDDRKYELYLFMCVCVCVCVESLPSLEYVAQFYFGLGLARLFHRRSYW

>Tb11.NT.85_2 [244 - 381]

MLHNFILAWAWHGSSTGDHIGESLYVPKTLLVREQKKQGEYIFKPR

>Tb11.NT.85_3 [317 - 556]

MFPKLCLFANRRNKGSTSSNLVELTRQPLLFICRATCKLWDASLVYRRFETVQVLYQTVRVEVNDVLTRHDGFFSLTKVL

>Tb11.NT.85_4 [615 - 746]

MKLNEICDLFIVWIHHDLGTLTVWFDPQDGRSSVVFGPCLWRRT

>Tb11.NT.85_5 [700 - 834]

MVGPRLYLVLVCGAEHKLIVCGLLHVDLLGLPVYDTAILIFWYVL

>Tb11.NT.85_6 [773 - 850]

MWICWDCLFTTQLYSYFGTCYKKYHS

>Tb11.NT.85_7 [936 - 1097]

MFVVRFVSRLLPSFFSLNHGCRVADDNYQPHHWVVVGLLLNFLTVVLSPSVFLF

>Tb11.NT.85_8 [1121 - 1288]

MSASPHIDFFFFAILVYSHVLRADAVFQSKVRTPFPFFPQKEAQCFEAGARSYLSV

>Tb11.NT.85_9 [1329 - 1622]

MHAHMRANNKFQLPLYGTKVNEEECVGIVCDQACPGAGRRRKLCFFVFFHTILPFLIVSPFRTRKEMKNYYILLKWVVQTHTCLSFDTSMVGQFCFVS

>Tb11.NT.86_1 [105 - 212]

MKGWESVIRHLLPCTSNLTVLGRAFCDISHSITGAT

>Tb11.NT.86_2 [239 - 349]

MEVVPECVNTICPRKCVVRSGGAMICIGLDVIVSTAV

>Tb11.NT.86_3 [511 - 615]

MRQYCLGCDLTGRRRSFGSNAEKSRAAAPTDCTGR

>Tb11.NT.86_4 [825 - 935]

MVGQLTGLYCNVDFFFQSSFHLYSQTNELRAHTNASV

>Tb11.NT.87_1 [93 - 245]

MFADCCDGVSETSHHAYIPVYLHISVCVCVCVCVHVSFYMLLRGRPSYSCG

>Tb11.NT.87_2 [112 - 369]

MELVRPLIMHTYPYIYIYRCVCVCVCVYMSPFTCSYGGAPHIHAVKCLDSAIITATVFTTNRVTKTAQHSTFLYASFFFFFSFFPF

>Tb11.NT.88_1 [189 - 272]

MWNPEKPVRPCGRSRTTWRMKWNSWRLR

>Tb11.NT.88_2 [185 - 406]

MHVEPRETGAAVRALADHLENEMELLEVEMNRLQDTLRQMVEDEKELLALKQLIEGDSPILCSSDTRVSKGRRR

>Tb11.NT.88_3 [406 - 513]

MSGGGTNVLVECRSTATFLPVSLQQCLKGTSVQMCV

>Tb11.NT.89_1 [61 - 222]

MRSSSKEETHGVGMLILTSQLFYVWFSAIQLRKGSKWGPVSFGVSSRYKCFNFF

>Tb11.NT.89_2 [343 - 465]

MCRRTSNPIDSGVNDARACALIPGGNFCCVHMAAPSIVYPG

>Tb11.NT.89_3 [249 - 512]

MPLYLTCHLFGGRARRIVHLSSKCNKNSFHVYVSSNVKSDRFWCKRCSCMCLDTGRKFLLCAHGSTIDCLPRMKLPVAVCTPLNHIFF

>Tb11.NT.90_1 [90 - 746]

MGKRDLEKERQIELAAIWGAEVDGENRFPRRRGLSGKRSTGASCLPVGVSVEDVDEVPSDLGSETRSEECNNAKSDIKKKKAGKDRKTNSTYRLHCFVGPGTERNDVRTVFEAYEPKVELRTSQQGSLLNKTHFAVLTFRNKAMALHAVKVLDGTNQYDRLGVRKLKLGLMLSRKEHSKIRQKLRKRMREDHERRLAEEVSEEAEFVKKFIESYVPSAG

>Tb11.NT.91_1 [278 - 355]

MCRRKLFVFVRACVCLCVYGAPTAVR

>Tb11.NT.91_2 [685 - 774]

MPRRCFTYEYVSKHLRAFISLRPRAVFAAG

>Tb11.NT.92_1 [9 - 119]

MHLLLPRSFFCTDVGLPPFPRITPSFFHFPGTLTPRL

>Tb11.NT.92_2 [159 - 242]

MAIALPTPSRRPRCFFVLFFLFCAHSLL

>Tb11.NT.92_3 [5 - 292]

MDAPSIATLLLLYRRGAPPVPSNYSVLFPFPWNVNTAALDEIPDVLTNPPGYGNRSAYTFPPSSVFLCAFFPFLCAFPVMKSSPMKTARQRWSDDG

>Tb11.NT.92_4 [285 - 368]

MTVNGHRKRTGPRFKRNQSPIMSSTGII

>Tb11.NT.92_5 [373 - 480]

METAPVDVAGRYTITQKYRYSLHFGAAACSDIKISS

>Tb11.NT.92_6 [562 - 699]

MPPSLALREHQVSCAIHMKLGTCLVRTVSSKTETHCRYPRKRICTC

>Tb11.NT.92_7 [802 - 930]

MPADSPAKAGFIRFRGMLFIRGPARGGNNSQRRARGCGQLYCG

>Tb11.NT.92_8 [940 - 1188]

MNHGEGLLKEGVKPCMVINFFHGVRGRMNGGPLFSLTPLSPKKKSSLVRQAPSILKDSEYYSKAARSRQSKSVHRVLKGGMTS

>Tb11.NT.92_9 [1499 - 1645]

MCLLNCLILADGADSIVSELSCPPRPLYGHDRSLFLCSSPSLPLHVFVF

>Tb11.NT.92_10 [1530 - 1664]

MAQIRSSASYPALQDLSTGMIAVCSCAPPLPFHSTCLYFDLGWNV

>Tb11.NT.93_1 [91 - 366]

MKSEKRGFSFPNFRHIKLKVACAALFPVKKSMLCQAIHWQLRCFPVTREPRWFSWLSTLSRKRSCNPNCHLQLRIQEHTSVWCKPTISREGR

>Tb11.NT.93_2 [411 - 608]

MPLDSCIRIATWRQHTHGRFVVIIDMQDCHPGGNRSTSVCICRNPPCPANRSLYATLLHTAWRSNA

>Tb11.NT.93_3 [571 - 657]

MPHSSTQRGVLTHDTGLISDGQAHKKEIR

>Tb11.NT.93_4 [664 - 828]

MKMDRSSSDVLSVSVSVLLADGNGSVQHCNVGFPPSDLVGPHQVFSSLLLCLFVF

>Tb11.NT.93_5 [689 - 841]

MYCQCQCQFCWLTVTGQYSTVMLASLHLILWVLIRYFLRYYCVCLCFEAAV

>Tb11.NT.95_1 [169 - 258]

MAAVTGLRMWHNGRQLPTQRSNGSSESISA

>Tb11.NT.95_2 [57 - 338]

MSVVCAFKGCSNLTYTALPACEHCSQRMCTSHLLPEVHGCGDRAKNVAQRKATADAAEQRQQRKHIGLDDAKARLTRRREELAAQRQKKPIKKK

>Tb11.NT.95_3 [369 - 479]

MMQFTVECSPCTSRKRDKYLLVYKVLRNVHNCCIIVT

>Tb11.NT.98_1 [277 - 468]

MAAGLLRPFQKKHSNRVHGTSFTHSYLLRNSNLLVTYRKGGQTKNINAGKRCCWVYRKDLHPSF

>Tb11.NT.98_2 [416 - 502]

MQVNGVVGYTGRTYIPRFNYVNSVVRIAP

>Tb11.NT.100_1 [141 - 434]

MSQKGLKGKTRGKSEGALRKYVGEAKKKTVFNKGRKTLQTKARANYISAVESHMASRVPSDQRDRLRVVKATGGLQPKKKHMKKPLTRGRKRKGDKKG

>Tb11.NT.100_2 [434 - 550]

MNCIPWLCGCRVVSTCWTHPLVALLTPITSSGKKGCGER

>Tb11.NT.100_3 [454 - 624]

MWLSGGLHLLDSSPRGSPHSNHLFWEKRMWGEVTVIIGGFEMYGTAVSSKQVLFDAL

>Tb11.NT.101_1 [102 - 302]

MSSVGSNRNSSSREDNSTCMKCHKERITYCTNSCGHALFCGKCAMKVATGGKCKLCGTLYSGVKRIS

>Tb11.NT.101_2 [344 - 505]

MGEVRGLWKCAGGLNPVMFALSAPFTGTLALQGYFSRIHSHLHDWCYLWVMCFD

>Tb11.NT.101_3 [552 - 650]

MPLPILPPRSRCDLQKGSQNMSNVFLYVVGTVK

>Tb11.NT.101_4 [370 - 735]

MCWWFEPRHVCSLRPFYRDVGPPGVFQSYSLTSTRLVLFVGDVFRLMYKKKHSAVLSVGYRCRCRFSHPALDAICRKAVKTCPMFSFTSWAQLNKFIFVPSFLLLHPPRCLWIVVVFLCVGG

>Tb11.NT.101_5 [702 - 821]

MDCCCFFVCGWVKTSPVCYKNTVVLKSVFYLFRGCYTTAT

>Tb11.NT.102_1 [66 - 182]

MTLPWWPMATRYSYDSPPVRTVVRNIYTYQSCFLSSCSL

>Tb11.NT.102_2 [158 - 343]

MFSLLLLLVGARVYGMLASKRYLSDLTHTMLLHCVMSACKGMAANVCVYVRNVGTVYTATVL

>Tb11.NT.102_3 [390 - 476]

MEGNDLRGLYCVMPSLWRLNVTACDSNTL

>Tb11.NT.103_1 [32 - 157]

MRELPFSISRFLGQQLSTAEWLQCRRTCTEYCTVCDTKLYDE

>Tb11.NT.103_2 [172 - 315]

MNNFDEEEPQLKRGGSIWCCIPHKIKRRGLTHLILQFRCHALVLICCG

>Tb11.NT.104_1 [141 - 257]

MGVVELTGEAVVVTEGAGVGRGGGALRGEWKRKEGTLHI

>Tb11.NT.104_2 [264 - 395]

MGGVRLPADAQNFHRLRTCACLVHVALELWSICMCVQLAAETGA

>Tb11.NT.104_3 [350 - 424]

MVDLYVRAIGGGNGRLNIYIPLFFF

>Tb11.NT.104_4 [289 - 639]

MHKTFIGLGRAPAWFMSHWSYGRFVCACNWRRKRAPKHLHPPFFFLNPHDCWLLSAVSLPFVGVTVTQLILIHCMSFLSSTHGTIGFCIVTRRLQLCKGLFIPSSNMCQLYCSCASS

>Tb11.NT.104_5 [627 - 797]

MRIFLTVPSGLVSFPKTFLIASLTFDTMHIISFAHIHEFRSVGTQCSAKHVIVPLTV

>Tb11.NT.105_1 [62 - 223]

MLLYLAFCLMLSDFPSDMQKHAGKDKVRRFFRCFGMFAALWLGAALMAVLGKWV

>Tb11.NT.105_2 [240 - 314]

MCVPMDCPLHFLFSSYSFSSPLFQL

>Tb11.NT.105_3 [157 - 459]

MLWDVCGSLAWRCVDGRVRKMGLSTHCSCVCQWIAHFIFCFPRTLFPAPFFSCNDAPARVLRGPVSHCALPVACVRVVLEVCLCMYIHICICIYTCVCFLS

>Tb11.NT.105_4 [599 - 682]

MFPNIYPAFRRDLHEYIHEHMCCVYACP

>Tb11.NT.105_5 [519 - 896]

MRTECRMFSGAKHKQLLTCLYACVLRTCFQTSTLPSGGTCTSTYMSICAVFTLVLDLVTIYNCVHLHFTTLCSFVHLLVFLNSNAHHPRSYSTVLVRGTKLQTRGVRIYACVWMCVHVFLCASMRV

>Tb11.NT.105_6 [769 - 924]

MPTILVVTLPCWCGGRSCRRVVYEYMHAYGCVCMCFCVRQCAFECLPARIFK

>Tb11.NT.105_7 [848 - 943]

MRMDVCACVSVCVNARLSVCLRVYLNSDLADI

>Tb11.NT.105_8 [982 - 1104]

MRLFLPLLSFCAGAGAGVCLCVRSCGWDGGKCIALLLFPSL

>Tb11.NT.106_1 [171 - 266]

MILRCDQFSPLIYSVLFGWGVGANIHYHCKKN

>Tb11.NT.106_2 [304 - 417]

MFLGCQLKAPATGSRKSISSNTPPPKHVFSIFSVFLES

>Tb11.NT.107_1 [237 - 326]

MRINVKLWRYISVTRCPCDRMAPEWIRKAS

>Tb11.NT.107_2 [308 - 412]

MDKESELMAGRNVNPIICCVEIICGCESESEVGKL

>Tb11.NT.108_1 [92 - 379]

MGDSEATAAAALEQSMRSGRTVVYYTHPQKLPSTSRGLTNIVSGDQQGSGADSRHYKHEEVVAAGDFSFLPFPSYCGRTTANTVLYKHFLSFDVHT

>Tb11.NT.108_2 [264 - 404]

MKRLSRRVISLSSRSHRIVEGQPPTRFSTSIFCLLTSTHEVNLSDAR

>Tb11.NT.109_1 [2 - 535]

MEGEEKVELSQLELKLEELQVIWEDVQATCPATHSSKRRNSSDAVASPPRVRSTSGAAQRQGANTQEPLQLPSPQSKHPSLRLLHANNKGKLRCTNPVASEGAVLGGIVSVSPPHSPANASVGACPHGRDGGVRNCVPSRLPPLDASHAQELLKACSNCGVEQRRPTAQFCWNCGTRI

>Tb11.NT.109_2 [466 - 567]

MFKLWSRAETANGAVLLELWDPHLKRSLPLSVCL

>Tb11.NT.109_3 [608 - 754]

MERGNLKQSFPLPPPEGERLMMFSFFLLLNAKSMWGGTEPCQGKEEKRW

>Tb11.NT.109_4 [727 - 855]

MPREGRKTLVITVRCVSRCYQYYLVIFHVSGREIFFFFFLSPL

>Tb11.NT.109_5 [696 - 857]

MRNLCGVEPNHAKGRKKNAGDYCALCVSLLSVLFSYLPCVWEGNFLFLFPFPSD

>Tb11.NT.110_1 [109 - 213]

MALLAVACVFTSSFPRVRGFIGSLHFVIASSYWEQ

>Tb11.NT.110_2 [66 - 419]

MDILQNIALIFLWPYGIISCCMCFYVFFSSGEGIHWFTTFRNCFVILGAVVATAALPFFRVPSLSLFIAFSVWSVYAALVAAHYWRFRLWRRHLIPVNMPLVVMFLWEAYRVAEQMWN

>Tb11.NT.110_3 [332 - 565]

MEASPHPRKYASCGNVSVGSVQGCRTNVELTVVTEVPTPTRVVVTGFSSFTVSLFRLVPPLFIHIFLCSFFIPFYQFS

>Tb11.NT.110_4 [532 - 612]

MFVFHSLLSVLVIIVKVTQLNPTCGDP

>Tb11.NT.112_1 [82 - 438]

MEPREMSEEELELRFERAMLLDEREFLVRETESRAELTARASTARRNEAERDSELLRLYLNGLLRGNLDARRKAEAQMREKVKAKRTHLAELRRIFAELQKAAVELRERCAAYGAGRTF

>Tb11.NT.112_2 [551 - 664]

MSGKNCAYALIIDACVYIGCRCQHISRLPWRLSSLCGL

>Tb11.NT.112_3 [588 - 665]

MRVCILGAGVNIFHGCHGASLHYVAF

>Tb11.NT.113_1 [183 - 290]

MQRDKNNLEKLQVFCRWLFSPDSYYHYTYTRKFIRG

>Tb11.NT.113_2 [356 - 517]

MRCTPSSLSSNRGGVRVLFLLAVRLVADAGCERLGTSQLFRMVGHFIYFFVETF

>Tb11.NT.113_3 [438 - 527]

MLDVKGLGHLNFSVWLDILYIFLLKLFKSR

>Tb11.NT.113_4 [527 - 613]

MKCFFFRFLNPPSLMIEVPFVCSCKLPSY

>Tb11.NT.113_5 [1401 - 1526]

MLKKLNEQSLHKTPGEGGWWGGALQKILCSKICSFTTDRLHD

>Tb11.NT.114_1 [22 - 183]

MGSCSFSLPVGFKHVLEADSALLELKAVCRTSKTKRIPRGRRWKAARNYTDIEN

>Tb11.NT.114_2 [147 - 221]

MEGGPQLHRYRKLNEVKATRWCRAA

>Tb11.NT.114_3 [352 - 453]

MASRFQFIGQSPCFFFSVTPRVNRVPAAVGVIGT

>Tb11.NT.114_4 [468 - 689]

MSLAETVWVGWDAACSSLLLFVVPVAFHCLVRGATFASRGKAFGKEDGVLARTVATFLPLVSVVKVGRKGRWGR

>Tb11.NT.114_5 [793 - 933]

MCEWAGDLLDQVPLLWRGSEVWWWGFLPEGCAAAMVHVREVSPGMGW

>Tb11.NT.114_6 [951 - 1067]

MCSPALGRPEEGLGVCGSFALLVLRFHYFLPVSVHSNSP

>Tb11.NT.115_1 [190 - 288]

MPFALVLQTSGDRCSLQRNTCSLIPQLCAVVVP

>Tb11.NT.115_2 [249 - 341]

MLSDTTTLRSGGTIETALRSTAVCPVGWAHP

>Tb11.NT.115_3 [345 - 455]

MGNNTQALVLTDLKHLCTWGCVNYVLEFGAHLGMCFT

>Tb11.NT.115_4 [415 - 546]

MCWSSVHIWVCVLRNYCEVCSPTAHTALAAPEGGGTIVTITSKL

>Tb11.NT.115_5 [572 - 679]

MGSCQRQNGPTRFTMVLAQALMTYVESMAKTYARSN

>Tb11.NT.115_6 [642 - 740]

MLKAWLRRMRDLTEGLFYQWRLHPVVLLVCFAD

>Tb11.NT.115_7 [813 - 932]

MINFSVLAVFAFMCTKQLSYHRAEVFPTVRRDSLPFLERV

>Tb11.NT.115_8 [959 - 1033]

MRDSEIAQHLEPTLPFPMRVVWLMN

>Tb11.NT.115_9 [1048 - 1149]

MQISTFVSVWLLCGTFLTQPGSDSFSCGLNECAA

>Tb11.NT.115_10 [1140 - 1235]

MRRMKSFFIFPNNLLFSKPYTHALYIQLIGGD

>Tb11.NT.116_1 [272 - 478]

MCYTLIFCLSFFFLACNQLVDHLFLFRLAPDDGGGEHQNMYPASCYLIKKRRGAARRVVCVHTCVNMVV

>Tb11.NT.116_2 [549 - 647]

MSVAVSVLFCQKLGGGKRKRESVCCSGYVPASI

>Tb11.NT.117_1 [198 - 350]

MIFLHTFSTFIGEYYCKDDGGGVSYNGLSPASPTQHTKWSVQGQSDGGRCS

>Tb11.NT.117_2 [253 - 423]

MGGGFHITAYRLHRQPNTRSGQFRASLMGAVALRHFLRCCVELLSTRVCVGTTSVGS

>Tb11.NT.117_3 [396 - 566]

MCGNNLCWLMMPCVLAAAGNRAERQGAIRAAATTTKFVSFSFSSLFSLFPLPHHWWR

>Tb11.NT.117_4 [588 - 746]

MHMYVCRRMYASVCERFASFLVSLFFSLRFGRFFSFPPQFCVRFASTTEKRST

>Tb11.NT.118_1 [70 - 153]

MGLNPRCRCTCTTRSAVGGCRPSLTPDV

>Tb11.NT.118_2 [15 - 227]

MAKSKNHTNHNQSRKNHRNGIKPPLPLYMYNSKRGGWLPALVNTRRVRKNNQKAALKARRERLAAHQAAQK

>Tb11.NT.118_3 [251 - 415]

MVKRVVTFLMAPLSILLLYCCDFLFHTPLSVLLGCFCVPSQSYFCGRAALATFSC

>Tb11.NT.119_1 [136 - 372]

MTDVNRYLQSDVATALNRLASAMARDLDADKQQAAAAFAVGRNTSPEAFMRVGEEVPLPPRVAELRRSQGRLSCGLPFQ

>Tb11.NT.119_2 [449 - 661]

MVKFFFPFSPTVIVGSPSYFLLLCRVCERGTTYRPCDVEFVQPQPNPLVRGIFSTTFGERSSSFILPRCYS*

>Tb11.NT.119_3 [558 - 665]

MWNLYNRSQTRWSEAFLVPHLGNEALHLSFHVAILE

>Tb11.NT.120_1 [192 - 290]

MMLFFLCEIPSKPSEKGVYLHFLIFCYSGVFCC

>Tb11.NT.121_1 [143 - 238]

MRGHVGFVPVHTYVGKCHAYRCGCSVQYTFEW

>Tb11.NT.121_2 [153 - 275]

MLDLCRYTLTLESVMRTAVAARCNTRLNGNFCLFCYCLLVL

>Tb11.NT.122_1 [327 - 401]

MEGDGKTSREEAASVDAGVLYGFRF

>Tb11.NT.122_2 [268 - 402]

MRSCEKIWTGEIQKKNWLKEWKATVKQVEKKPLPWMPECCTDFVF

>Tb11.NT.123_1 [135 - 221]

MENEGREIEKEVMSYNQSTRRRNESGEYG

>Tb11.NT.123_2 [655 - 810]

MRGATSEPLILNTGVPFFHSTKLGPFHIWGHRTLWKYIGLSPHVVRATRRYS

>Tb11.NT.124_1 [288 - 377]

MLCFVKADAFRFRWRLRKVMQLDNRYDFVL

>Tb11.NT.124_2 [544 - 732]

MHIKVADAVRAYMCVMRRHPYCLCPEVFPRFEYMVIILGGGMGEFFKYYKPPLHVECLHIVLL

>Tb11.NT.124_3 [711 - 953]

MSAYRSFVATGVPFFFFFFVNFPDRSCVRLGGDTDPPLDGICLPPAKGRLRNRFVVFFFPRVQCCTLVTQFCVHWSAPSFE

>Tb11.NT.124_4 [993 - 1193]

MKITYLSFYHKLQVVFYTFQLSAFPLLVITRVSHRTVTKKIIAVCFNIKAWLFGSFISYRVSSLKKH

>Tb11.NT.124_5 [1310 - 1393]

MLSLLKYCQQPEFFLRSYLQVCFCCCHH

>Tb11.NT.124_6 [1429 - 1509]

MKLPPVVIFFLYFYPFSAFYAMGGLTN

>Tb11.NT.124_7 [1543 - 1629]

MCSSRFVQRISCTCVCKGAQPHARIVNVH

>Tb11.NT.124_8 [2077 - 2163]

MLDAVVVIKHYISLVLLVAKYRCVGLTEC

>Tb11.NT.124_9 [2224 - 2340]

MLTTGWFGASSCVLPLFNLIGMYFVLFVPAREEQVEKGL

>Tb11.NT.124_10 [2452 - 2535]

MQTRQVCECFLATRGVLLHSYGVPLHTA

>Tb11.NT.124_11 [2475 - 2615]

MLSCNAGCITAFVWGSSPHSLMSLTFFFAFDPKYLGSCSSSPEIWVK

>Tb11.NT.125_1 [122 - 244]

MRCTASSNSHRKRREIPNIRKKASCHRAPPTAGNLEQHFYA

>Tb11.NT.125_2 [250 - 390]

MLEDVHCMIKNLPILHNDYTRSCALFDRELNGRGCGSAWLTVGSDKS

>Tb11.NT.125_3 [54 - 443]

MPHRSTVSKKRSNSVSRPTAAQKCAAQPRPTATASEGRFPIYGRKPAVTEPHQPQAIWNNTSTREDVGGRALYDKELAHIAQRLHEELCSFRQRTQRARMRECLAYSRLRQILTTRLSVRNIVEEQSEGR

>Tb11.NT.126_1 [177 - 263]

MKVRNAQTKVYPNLRCMTLRRSYWRQVRT

>Tb11.NT.126_2 [20 - 358]

MGGPPRKREREGEALKPKSKEEQLPIKNECTLDRREGKQIVAEKWSEVVVDDNESQERSNESVSESQVYDTEEELLAAGEDLSGDALKDFLRKNFVDFIDEKVARVGDLDLMN

>Tb11.NT.126_3 [437 - 526]

MLARCNAWGREEVPALGVSSVCFVFTSQYG

>Tb11.NT.126_4 [448 - 663]

MQRLGSGRGASSRSLFRVFCLYLTIRMKSGCSGCFDMFLALSFFFVLLFPSISFFIHYLWRFETPAGEPASW

>Tb11.NT.127_1 [217 - 303]

MCFASLTAITRMAHCPLCYDGMVNLAVTP

>Tb11.NT.127_2 [320 - 457]

MLLTFCFHGVKCFTVLSFSTGRISYEILRQTTVVVAIFLAPRGACV

>Tb11.NT.127_3 [352 - 489]

MLHRAIVLNWSYLLRNSSPNHCGCGNLPCTSRRMCVSVCVCVLALH

>Tb11.NT.127_4 [494 - 580]

MAFVPDYPSVCTATRVIVSTASGYHVFSR

>Tb11.NT.127_5 [567 - 713]

MCFLGSVQCPNWRLKKKISKEPTLFGGRLVLFSPVLVFVSVVITYACTF

>Tb11.NT.127_6 [700 - 819]

MLARFNPTFLVVVAVCLQEVSFYSLRYEETVGDTDHWFNV

>Tb11.NT.128_1 [172 - 462]

MAEKIASDKITLHLAAKTGITEALGNMECDPAETLGKVLSRIKKKLKRPALYLFLMRGSEGFIPTPDQTIGSLLHAYAESSDQRHLSFAVSTAIFHG

>Tb11.NT.129_1 [37 - 117]

MPSPPEPSSVLLRRGKAYFICRGRERL

>Tb11.NT.129_2 [235 - 315]

MGIVWQNREVASAVAIAPKTGRLQSAV

>Tb11.NT.129_3 [328 - 486]

MRRVRLSPPLLRFPGSLFPKKNLFSKAIPLAIQTCRLFVSYSEPSLLLLLLSQ

>Tb11.NT.129_4 [494 - 790]

MCEVIRSARDSAERMGFGEGWGVCGASTRRQHRVGLFSSGSSSEHLPCMKGWSGRKDVSLIYAYHTCLLHFLLLHTCVREDVVTTPVFSPSTSCALLEC

>Tb11.NT.129_5 [967 - 1140]

MCVATLVAAACFVFRLKVAVAQKSTSCLEATVAHGVCFVALSNVAFCHLCCVHVVSGG

>Tb11.NT.129_6 [1210 - 1284]

MSLRDLANFGRRRIRGVPNAKVLLH

>Tb11.NT.129_7 [1417 - 1512]

MWQSGRIWYMSTPCCHGEVCDRCACCFHVRIV

>Tb11.NT.129_8 [1670 - 1774]

MVLITLPPSRIISKRREAGAGGHLLTVWPLARRGA

>Tb11.NT.129_9 [1627 - 1791]

MFFVLDGEGCRKYQDGVNNPPPIKDNKQKEGGGSGGPFADCLAFGAERGLTRYNV

>Tb11.NT.129_10 [1787 - 1861]

MCRNLKLLFSIIGIIYISPCSEMIL

>Tb11.NT.129_11 [2080 - 2166]

MVPAKRWMTGIKLRYHCGTSRGEIKLVLP

>Tb11.NT.130_1 [587 - 688]

MYVMFDGIWPISKWSSFWWVRTKHGLNLLKTVSR

>Tb11.NT.130_2 [965 - 1072]

MCVGASYFFQFYFIRATKSNNTIRSKLWNPCVVIAI

>Tb11.NT.130_3 [924 - 1079]

MCCIILCSFLSMGACVWALRIFSNFTSSEQRSQITPFEANYGTPALSSQFKK

>Tb11.NT.130_4 [1234 - 1380]

MYAFRYIYILGSVLAVSVLLGLWLLLPYCYLVICQRLGSLHFYSGGACG

>Tb11.NT.130_5 [1299 - 1403]

MAAATLLLPCYLSAVGVSSFLQWRCLWIRGTYELT

>Tb11.NT.130_6 [1629 - 1817]

MERYFHMYITYNLISRECAQCMNAVHILFFFCILCLETAVNVNCLKVCLSYFFHSLHRPTRCG

>Tb11.NT.130_7 [1679 - 1858]

MRSVYECCTHSFFFLHSLPGNSGKCELPESVSKLFFPFTSPPNSVWLSFPWPSRCDICYY

>Tb11.NT.131_1 [186 - 260]

MLRVKRIPRDRLPASTYSAAAVRLL

>Tb11.NT.131_2 [310 - 396]

MKSVYKLLDCTSDDRRELELKTVGVPVSG

>Tb11.NT.131_3 [421 - 501]

MFFVLRSCFCMRDGGSTRLLLCGRGCC

>Tb11.NT.131_4 [458 - 541]

MEVRQGFCFAVEVAVSTDFTRTWGEPCH

>Tb11.NT.131_5 [553 - 693]

MFEGAAHLSYYCAVPRSTFLLGPRSSLSNVCADLIHRFKERSLFRRS

>Tb11.NT.131_6 [1054 - 1149]

MKTRFLKNMVSVPLSLLFRKRVCFGVVLPPAT

>Tb11.NT.132_1 [93 - 176]

MPAIYTAVLTHFFSCYQQRFLCTGRRLV

>Tb11.NT.132_2 [830 - 922]

MQVRRCHPHQVVMLLVPWVVISTEANKNIWL

>Tb11.NT.132_3 [1109 - 1309]

MCVVVSGFGPPATFNGGYVRMIGEGFRFFATIWVMWVVALSTEIQLLFARAGVSKGGYRYGLSTFII

>Tb11.NT.133_1 [140 - 409]

MTQLPSATIKLIEMPKHMENYAVNCAQDGFTKYYTEKDVASHIKKEFDKKYGPTWNCFVGRNFASYVTHETKHYIYFYMGQMGVLLFKSS

>Tb11.NT.133_2 [336 - 461]

MLPTKRNTTYISTWGRWVCFFSSRHEDLLLFRTFYVSAFTTL

>Tb11.NT.133_3 [409 - 492]

MRTCYCSAHSTSVPSLPFDAQWTKGWHH

>Tb11.NT.133_4 [464 - 550]

MPNGPKGGITDDFCSPFPSLPFSVCFVSS

>Tb11.NT.133_5 [471 - 668]

MDQRVASLMIFVPLSLRSRLVCVSFLHRPRVVRSVGRAFVSVSVLHLSVKTALESYPTLKAPFVVV

>Tb11.NT.133_6 [691 - 861]

MKQTFKKRVLRGKGKRENVYVGQNRRGGDSCDFFPSGLPPSVASICSVHVVIRKYCI

>Tb11.NT.133_7 [743 - 880]

MYMLARTGGVATLATSSQVVFPLLWLRFVLYMSLFVNIVYRIIHWL

>Tb11.NT.134_1 [100 - 399]

MQGLVRNDGRCCAVRETSCSCVFVCVCCSLSVQLSVMLWECPRFLLLGRCSGRDVGWAPLVGKDNLHVSPRGETWVFTPDGSRGQFLSMVWQLRNFGVRS

>Tb11.NT.134_2 [299 - 418]

MLARGARHGCSLPMDREVSFSRWCGNCVILAFAVNSCLEV

>Tb11.NT.135_1 [79 - 165]

MALPPKDWHGQRIPPLRHLLQPKGSHPQV

>Tb11.NT.135_2 [62 - 232]

MGHLHQWRSRQKIGMGKGSRHCVICSNQKALIRKYELNVCRQCFRENAENIGFVKLR

>Tb11.NT.135_3 [201 - 335]

MPRTLVSSSCVKIADTSSNDERNGGTDDSRDWLYGRPFSFCLFFH

>Tb11.NT.135_4 [256 - 387]

MMSEMEGLTTPVTGCMEGPSLFVFSFTDRGTQLTCFFPLFFSWG

>Tb11.NT.136_1 [220 - 330]

MSLMRVLCWWHRHFVGTTILKLVVVDWSTLTPGGLKV

>Tb11.NT.136_2 [500 - 577]

MCWTQAVNFARTKRQGGGICMMCSEG

>Tb11.NT.136_3 [577 - 702]

MNQGMSKSTRGGLGAGQQAMQEELQVGICSLFLRTFVFLYRL

>Tb11.NT.137_1 [28 - 105]

MNEKGRFSRCDIYVSNIIPSCAQLRR

>Tb11.NT.137_2 [87 - 437]

MRAASQVNVGRGRSEAMGERCSSSNCWSCSVCACIFDPSCFRVTLRNFSRYPTLFFVVVPASLRLRAEDLLSFEMWKLDGVLGNWRYCLQHAASFMHWLPPPLSSPSLWLTLLEIAC

>Tb11.NT.137_3 [410 - 565]

MANTFGDCVLMMFLSFWLPLSAGRLFCRLMQFRTCFIIVVMLVEVLPTPVAG

>Tb11.NT.137_4 [511 - 645]

MLYYCGDVSGSTSHAGCRLTLNAPFLFLFILLSLHHRFAFVFFCS

>Tb11.NT.137_5 [575 - 661]

MPHSSSCSYCYLYIIVLLSYFFVRRTAVW

>Tb11.NT.138_1 [112 - 198]

MALPPKDWHGQRIPPLRHLLQPKGSHPQV

>Tb11.NT.138_2 [95 - 265]

MGHLHQWRSRQKIGMGKGSRHCVICSNQKALIRKYELNVCRQCFRENAENIGFVKLR

>Tb11.NT.138_3 [234 - 368]

MPRTLVSSSCVKIADTSSNDERNGGTDDSRDWLYGRPFSFCLFFH

>Tb11.NT.138_4 [289 - 435]

MMSEMEGLTTPVIGCMEGPSLFVFSFTDRGTQLTCFFSSFFLYLVKMRG

>Tb11.NT.139_1 [314 - 394]

MKKLCFTRECCGGGSFPYTRKLYLTHS

>Tb11.NT.140_1 [22 - 126]

MYRLVTVVSGHQACLGDSLFFFHKLVRRNERVCRF

>Tb11.NT.140_2 [166 - 270]

MKSKEIFKCGSYRLLPCLLPCSRDKWRVPSLFPLL

>Tb11.NT.140_3 [694 - 783]

MIVLTQLNALRRNDLICFRSCTTGTCSACK

>Tb11.NT.140_4 [986 - 1066]

MCFATTDTVRGNIGKKNTTDPISRGSR

>Tb11.NT.140_5 [1393 - 1482]

MTLSPCMLHWRERAVAVGVYTISLSHEPRA

>Tb11.NT.140_6 [1469 - 1612]

MNPELSKCGICTNAAEHPSGGTWQVPPGAKAAKKRQLQGLAGGNSQRF

>Tb11.NT.140_7 [1489 - 1788]

MRHMHQRCGAPQWWYMAGTTGCKSSKEKATAGISRREFAKVLGTPETALPGVGNYMHILLAMEELKLVLSGLSVRTVSTGKMRAWDYRSSAGTKKPTETV

>Tb11.NT.140_8 [1935 - 2042]

MEYCSQVERLPEPFTSAATIRALTQEIDGRRCGKAA

>Tb11.NT.140_9 [2096 - 2185]

MVYTRTFGSVNHMCTVVALNHYKVDPRPTR

>Tb11.NT.140_10 [2185 - 2325]

MNGTASVLWKISSEDDQDMVSKLYSSPAEWSAKQPRERYFSLSQKNC

>Tb11.NT.140_11 [2228 - 2455]

MIRIWLVNYIRHLRSGAQSSRGNGIFHYRRKIAEKNVKHPRIGTAYFQTTPSSFAFKPGNRHLLTNLAITGRESFK

>Tb11.NT.140_12 [2614 - 2922]

MILPHHVIIEDFDNIFVWCSVPLSSVLHLLTLPICAYVDTCALIYSVKCTGWVMDSLFNFHKADFHSLYHSTFLLCMRRFNASLMSVNNGIFYKEQDKMLLRL

>Tb11.NT.141_1 [264 - 359]

MVDERCFAKCTPLIFLLSIMLMYFLLLFFYDF

>Tb11.NT.141_2 [508 - 624]

MVTEGFFIFLFNPILLAVAVFVGGGFLFCLLLWVTCFFF

>Tb11.NT.141_3 [794 - 889]

MFFLPLADLPKNVGAHIVITKICHLCPLVVLS

>Tb11.NT.142_1 [266 - 628]

MDRRLIEMQAVITRREAEFRKQNMDLMTELMGDMRSQDNLFDKNMKDLQFRLKGLEKQKADIIKAEDEVKDKLSKLSAARTKLAEQMAEMKEKMDGIRRQRPSGSLTARGKKPSKEPSVDM

>Tb11.NT.142_2 [771 - 896]

MKEERTYKVKNNFLEFFLLVWGVAVTSAALCINVGRLSSVAV

>Tb11.NT.143_1 [7 - 144]

MKPLGFRYPSRVPEPGSTFGWPASPGETKLYHFPRYTPKLNKYTRG

>Tb11.NT.143_2 [253 - 405]

MQVPVGAGVPESFGTVASTLRIKRLRVAGPSRFEHYVLVGIEVTVLAAVTQ

>Tb11.NT.143_3 [410 - 526]

MPKAVSHSLLLLLPWWRREGCVGRCAGCRVLVVPSFTKW

>Tb11.NT.143_4 [481 - 573]

MCWLPCVGRSIFYEVVMSSQFPIWVGQCYMG

>Tb11.NT.143_5 [680 - 754]

MSGHPSMAAGFQSQRGRSGVRVCLA

>Tb11.NT.143_6 [796 - 933]

MALFYFCPSFQVARPLYWGSITWVELFSEVVIAPYHFFRRIECEHQ

>Tb11.NT.143_7 [1170 - 1478]

MILPHHVIIEDFDNIFVWCSVPLSSVLHLLTLPICAYVDTCALIYSVKCTGWVMDSLFNFHKADFHSLYHSTFLLCMRRFNASLMSVNNGIFYKEQDKMLLRL

>Tb11.NT.143_8 [1435 - 1521]

MGSSTRNKIRCCCGCENVYQRSGSATGVW

>Tb11.NT.144_1 [260 - 442]

MVDERCFAKCTPLIFSADLLWYPLIVSVTWPYWVPLCHLLVPLNCLVVFILAHPPSERQSS

>Tb11.NT.144_2 [454 - 552]

MPLGFTIVFDLIPYRSHACRRVFASELDGSSIL

>Tb11.NT.144_3 [503 - 577]

MHAAVYLLPSWMVVASFNGTDRFPA

>Tb11.NT.144_4 [555 - 704]

MAPIVFLRDPAEPPTPTLVIFVGSLILQITGGIILALVFDHVLCKRQTKD

>Tb11.NT.144_5 [676 - 771]

MYYANGKLRIETNCSSCARLANTNECSTNNCG

>Tb11.NT.144_6 [750 - 833]

MLHEQLWIVMCAVLGWALCCLLLCVLRY

>Tb11.NT.144_7 [746 - 862]

MNAPRTIVDSDVCCFGVGFVLPPSLCVEILSCNESCMPY

>Tb11.NT.144_8 [1027 - 1107]

MQCVCMCVCAPWVRGFKVNLSFFCSSS

>Tb11.NT.144_9 [1427 - 1507]

MALVHLAAAVFIRLIFRYLFVDVKVCW

>Tb11.NT.144_10 [1612 - 1713]

MGLSESRIIKIEKLVGAVTARCCIVVWCVCVVLL

>Tb11.NT.145_1 [60 - 236]

MDPRRGGTRSNVGASSACDKDFLQTIRNYSMRVQQEEWMYSGPSAYFSLPKNSAPSGTK

>Tb11.NT.145_2 [293 - 388]

MVAATRCDQICAFTFPLVMHVFPLSLFLPRMN

>Tb11.NT.147_1 [192 - 293]

MCRVGADQPQLVTRPLCRGETSAKGVRRCSCEGA

>Tb11.NT.147_2 [85 - 330]

MEGNFYVSDFVIKRVLRSNYGHCFTRCRDEVAAYGRCVESGQINRNLSQGLCAEERRALRACVDARAKELKQKTICDSGSKQ

>Tb11.NT.147_3 [378 - 482]

MLGTFVVVGWLTMMSAGKVGPMRGHSVQTQRWTTV

>Tb11.NT.147_4 [275 - 514]

MLVRRSLSKKRSVIRGRNSRDLRVSSYIRVCTCTYVRNLCCRRLAYYDVCGESWSHARPFSPNSAMDYSLSVFRVLCPFV

>Tb11.NT.149_1 [62 - 196]

MEGCASAHPPQTLSHPSLKNRKTPYTSGRGNCSWMQKGSVSSLRR

>Tb11.NT.149_2 [49 - 342]

MHNSNGGLRISASTADPQSPKPQEQKDSIHLWSWELFVDAKRKRFKPSAVTVPISFYLHYVIQSLHFPLLPHVESRSVDEHHWRESYGVGRHTHLSSP

>Tb11.NT.149_3 [263 - 349]

MLKAALLMNTTGEKAMESADTLTCHPHNI

>Tb11.NT.149_4 [799 - 903]

MAPGRLHQGSFARPIQITPLPNLHRPKLVSSRPSW

>Tb11.NT.149_5 [1060 - 1233]

MLLSSYRVRRLIAPPAACRALADLRQKKNNIWRDGLKKRLSATSTGKNQIEALHGGGR

>Tb11.NT.149_6 [1431 - 1811]

MRLASVLRITETECNWLNKMNYRGMCKRWRHWYGAMCEDGIMTLHPKISTKSETVLDDGWGERVWKPRGSHKIACKGRQLSGCKTVYGRGVPKYHLQKTRTHMRMEYQGPLVVTASLPKAANRLNEM

>Tb11.NT.149_7 [1863 - 1967]

MANRLIMSTRTNSRKDEKRGRKIRPLEARGSRGGK

>Tb11.NT.149_8 [2128 - 2229]

MQRGKQWGARNGPKAPENTKIKVNTCRPTRKCMD

>Tb11.NT.149_9 [2254 - 2355]

MVIPEGRYGKGLCIRHRAGKSPGCVGKGFRAPRR

>Tb11.NT.149_10 [2276 - 2401]

MAKVYALDIGRASHQAVWEKAFVRRADDVQLEGGIQLFGKSG

>Tb11.NT.149_11 [2355 - 2564]

MMFSWRAEYNYLGNQAEKGGEIVITKKKHRREAQRRGRITPNIWGTRLKRSMPTVHSAFRPKKERNLVWT

>Tb11.NT.149_12 [2593 - 2718]

MPCSENSAPQRGYYLKQVPNNRDGDILSFGTKVFINIRKNAH

>Tb11.NT.151_1 [254 - 436]

MCWLSSRQAXXXXXXXXXXXXXXXXXXXXXXXXXXXXXXXXXXIFWLKKRGLMPGLTFSTS

>Tb11.NT.151_2 [28 - 828]

MPPKSHKRSRKEGEVEEPLLTENPDRYVIFPIKYPDIWQKYKEAESSIWTVEEIDLGNDMTDWEKLDDGERHFIKHVLAFFAASXXXXXXXXXXXXXXXXXXXXXXXXXXXXXXXXXXHFLAKEAWLDARAHLQHELISRDEGLHTDFACLLYEKYIVNKLPRDRVLEIICNAVSIEREFICDALPVRLIGMNSQLMTQYIEFVADRLLVSLGYDRHYNSKNPFDFMDMISLQGKTNFFEKKVGEYQKAGVMSSERSSKVFSLDADF

>Tb11.NT.151_3 [880 - 996]

MGLMPFTFIELDICYSGKVSDCTSLEYWLLFLTDIITRK

>Tb11.NT.151_4 [996 - 1106]

MSLWAAARMPPYACMQTHRIVHRHTREPACVRKYERG

>Tb11.NT.151_5 [1271 - 1378]

MAVSMCEGGEREMCKRHTILHFCTCQVPIVFPFDLF

>Tb11.NT.151_6 [1460 - 1588]

MAMYCARWLGCPLLSTVSFRYFKVVMSCRGLLGSVHIFFLFHF

>Tb11.NT.152_1 [247 - 333]

MSSWHYGLLISAGRWSDFTVVKQTVIHIQ

>Tb11.NT.152_2 [255 - 377]

MALRSTDICWKMVRLHSGKANSNTYSVDVGAATLVCLLPSA

>Tb11.NT.152_3 [469 - 591]

MSFFFRCCCQRAFPKSNYRPYLEPLRTINFKEAKNRNSNVF

>Tb11.NT.153_1 [249 - 335]

MSSWHYVLLISAGRWSDFTVVKQTVIHIQ

>Tb11.NT.153_2 [257 - 400]

MALRSTDICWEMVRLHSGKANSNTYSVDVGAATLVCLLPSACSAAALH

>Tb11.NT.154_1 [41 - 424]

MQIFVKTLTGKTIALEVEASDTIENVKAKIQDKEGIPPDQQRLIFAGKQLEEGRTLADYNIQKESTLHLVLRLRGGVMEPTLEALAKKYNWEKKVCRRCYARLPVRATNCRKKGCGHCSNLRMKKKLR

>Tb11.NT.154_2 [443 - 538]

MLSGYLRIILLKFYLFISFTDSGLYVFIDLTC

>Tb11.NT.155_1 [119 - 502]

MQIFVKTLTGKTIALEVEASDTIENVKAKIQDKEGIPPDQQRLIFAGKQLEEGRTLADYNIQKESTLHLVLRLRGGVMEPTLEALAKKYNWEKKVCRRCYARLPVRATNCRKKGCGHCSNLRMKKKLR

>Tb11.NT.155_2 [521 - 610]

MLSGYLRVICETCLISFFFSPCSCSLLFVR

>Tb11.NT.155_3 [556 - 693]

MPHFFFFFPLFVFLAFRSVKVRVIVNGLRVLSSSRTPVAGLVITLL

>Tb11.NT.156_1 [74 - 160]

MEIICCLWCRGAIPTDSETTASCFEEIWG

>Tb11.NT.156_2 [232 - 390]

MRSLIWSTEGCGICRVVMAAGGATGIQVLVGSVACMLLFSHGSLCHSTGWSAV

>Tb11.NT.156_3 [246 - 443]

MVHRRVWHLSGCHGGWGSYWYSSTCWLRCLHAALFSWFLMPLYGVVCCLETLTKQQQNWLQRGKCK

>Tb11.NT.156_4 [566 - 649]

MLTFRFGGGGQGAVSRASFPFTGTVVSF

>Tb11.NT.156_5 [493 - 675]

MYVSPSVTCDSYMQRYRGFIWSLMDAHVPFRWWRSRCSFACKFPFHGDSSQLLIVNSTHSV

>Tb11.NT.156_6 [825 - 899]

MNLESLGIYQLRDEVYKRLRDFGFG

>Tb11.NT.156_7 [779 - 1033]

MYAYVWLVMVCNNSCDEFGEPGYLSASGRGVQTTSRFWFRVSAVITKTFLCILAACVFCIFILTVQNHIITLRMHIRCHIVLRCF

>Tb11.NT.156_8 [1283 - 1384]

MYRVCCFPPLPSQKNNNNKKRSYIVDICKLRWGA

>Tb11.NT.156_9 [1462 - 1539]

MCCILSSLRLVICQETVLDAEPLICG

>Tb11.NT.156_10 [1446 - 1574]

MRGIVHVLHFILVTARHLSGNGSRRGATHLWLKVTASHLFLKK

>Tb11.NT.156_11 [1406 - 1735]

MWHHWYLTHNVLLNEGNRSCAAFYPRYGSSFVRKRFSTRSHSFVVKSYCFASFSKKIVCAERHVVTDVLDSSNADDSSPLISGVSTGYVYRTWGSVLVVCSFKPLFVFSF

>Tb11.NT.157_1 [156 - 248]

MLGERVYRACQKNCETAFSITTQAIDVRNKV

>Tb11.NT.157_2 [232 - 333]

MYETKYESVTTIPAGTCSLQRGKENDLHAHFCAY

>Tb11.NT.157_3 [248 - 367]

MSRLQQSPLVPVPYNGEKKMIYMLIFVPTDIRHLLKHLSC

>Tb11.NT.157_4 [699 - 782]

MGRIVSTLKELGVASDDIIFTLDEIFYH

>Tb11.NT.157_5 [891 - 971]

MLRCGLQFPQLKWLKCVCMGTQVSRGF

>Tb11.NT.157_6 [1008 - 1118]

MMGIKDKLELEPLCIHVIYIQPCWCDTAPFQRGIKLA

>Tb11.NT.157_7 [1118 - 1195]

MKKIIPVGCCIVGLPAWKKLYCLFMC

>Tb11.NT.157_8 [1165 - 1242]

MEKIILFVYVLVFLLTVPWGEVTSCM

>Tb11.NT.159_1 [36 - 383]

MLRRAGSRVACACSVPQARSLHFPITPPPIEIEYLDNDPLEFAVRTEARKWRFDDMGYMRELAFVRINNNPTVGDFRNMSPDERRNLFWGSDRQDFFRHLTCTLTGSPEHLYHRGW

>Tb11.NT.159_2 [402 - 509]

MGWSPTTTFLPHRRLWCCVELNVTPLSVNVGSEYVS

>Tb11.NT.160_1 [282 - 422]

MFVLRDHVLSANPGSIRPFLHSVAPVTVSWPISSRRRLLATSANSRV

>Tb11.NT.160_2 [603 - 701]

MDGMRWRRAVGGGYGSCRQVQFFLAFDPNLFQK

>Tb11.NT.160_3 [643 - 729]

MEVVGKCSFFWRLTPIYFRNNSQGCCVGS

>Tb11.NT.160_4 [886 - 1137]

MCGRSLAQASVLMLPFSSTCSVAVAAWRVGGEVAATKSLINRSCISVCVCVCLCVCVCVCVCVCVCVCVCVCVLRSSFGWYGVK

>Tb11.NT.160_5 [1122 - 1283]

MVWREVTDSVRSKGYLFARSVVLWLQLCIPKACAREENKISFALFPSDGSGSAH

>Tb11.NT.160_6 [1264 - 1350]

MGLGVRTETSSIGPDNVYLVIKLLVYDAS

>Tb11.NT.161_1 [151 - 234]

MRLPDCCFTHAQIGSGLSLIMCGPHSIS

>Tb11.NT.161_2 [537 - 656]

MPCGFYPAVHLPMLPMPRIGTLQFIPLLSIQRRCRPRAGR

>Tb11.NT.161_3 [542 - 700]

MWILPGCTPPDVAHASYWNVTVYSPFVNSTSVQTTCGPLNGCVSKCAASRVCS

>Tb11.NT.161_4 [660 - 767]

MGASPNVQRRASVRRRALAESRISQLETGGCAPDGA

>Tb11.NT.161_5 [676 - 990]

MCSVARLFVDGPLQNQESHNSRPEGVPPMVLRFPIGHHICRQVSRVLCERCVFAFAVPLIPASLVRLREDDHKVRLPMLRSTIPTEFFCISWQCFYVGPDHISAG

>Tb11.NT.162_1 [44 - 148]

MVVLRAFFPTTDVSLPSGSHAVVGVRKYKRLRCYG

>Tb11.NT.162_2 [144 - 308]

MDDAKLPKCRESMTSLPTSLSSNVYLLPPQHFVDFRLHMLWVRITRKSAFYKASV

>Tb11.NT.162_3 [565 - 654]

MQNCPNILKACSRQIVNAADDPTVGYRCPL

>Tb11.NT.162_4 [831 - 992]

MIEKQNTPQRLATDTGRVMHLSTKKATVSLKAFKIVRLSTKVTRGKVAEAIIFF

>Tb11.NT.162_5 [1036 - 1209]

MLKLSYDTKCIVLTANAHHISMTSVSSNCGWPKTLNGILKDIQQHRATRHLKHSSTAI

>Tb11.NT.163_1 [135 - 215]

MGITHFFVGVPFRPCNQRPLVILNVKL

>Tb11.NT.163_2 [176 - 268]

MQPAATGNTEREVIVDLLYTVWTAAHILLNS

>Tb11.NT.163_3 [488 - 571]

MYGTRSLRIIFLFACANPSGVRDDKFVS

>Tb11.NT.163_4 [555 - 632]

MINSYHSPRNKLVLWLPKMLTDGTSA

>Tb11.NT.164_1 [255 - 341]

MADWTFLTSPLVMIRGGNCFEQIVTSLVC

>Tb11.NT.164_2 [460 - 546]

MVVKCGAGVADAVALWDGSASSCLRQFLR

>Tb11.NT.164_3 [491 - 601]

MPLPFGMEVHQAACVNFCDDMAVALLVSCLSLSSLGH

>Tb11.NT.164_4 [546 - 635]

MIWLWLYWYRACLFPRLDTDLIAYRPRWRS

>Tb11.NT.164_5 [626 - 718]

MAFLSDVLIRGVTYSSGASMGLLSFRCCAVA

>Tb11.NT.164_6 [642 - 719]

MCSYVVLLTLLVRRWGSCRSAAARSL

>Tb11.NT.165_1 [316 - 621]

MEPYANDSGDVAQIGGEGRCHSHIPTLNGSCLSFSVRPIFLSDFGREKGGNRSQRAGGMLSLCQAAERGVWPYHRPSWSRRTQGYPTIKEMELIAPKHAKGG

>Tb11.NT.165_2 [501 - 668]

MPSSGEGRLALSPPVLVKEDSGIPHYKRDGIDCPKTRKRWVTTVKVHAGLSAMQKS

>Tb11.NT.165_3 [713 - 790]

MFCISGQFNEFSLFNRLHPHQSHHTA

>Tb11.NT.166_1 [335 - 463]

MLRGALLNGGNCVQVRICLPCLFVSLAIRNHRQFTFGFSVWPQ

>Tb11.NT.166_2 [357 - 488]

MAETVCRFVSASLVCLLASPFVITDSLPLDFLCGPSSPTITNGG

>Tb11.NT.166_3 [557 - 658]

MYPEFYVHLYRILGCFAQEHCNVTVGEEGMVCKK

>Tb11.NT.167_1 [94 - 288]

MWCGECPEKKKNEKHCIVRSVKARQRKWASGSWKRGLRPSCGFTYWSVEAAFRSVADFIHEGRGS

>Tb11.NT.167_2 [294 - 443]

MYVDHPPFGGELFGYPVAIETSREQLLESCQIVFRSATNERSGCGSVRNR

>Tb11.NT.167_3 [517 - 591]

MVHLSWEEMRFTIFLSIVLSPNDTS

>Tb11.NT.167_4 [482 - 691]

MLSNVVDRWEKRWFTFLGRRCVSRFFLALSCRLMIRPNVVECVYRGGGAAAALCELAAMVSSIRLDLRSE

>Tb11.NT.167_5 [798 - 920]

MRAKTEPIYWAERSCRMIPPCLRRDDLLRIVGTVAVAFYLS

>Tb11.NT.168_1 [99 - 185]

MAIEEGIARVLHAQMEVMGQENLSVRNFL

>Tb11.NT.168_2 [566 - 712]

MGIVCNVTTLNMRCRKLGMSDSVTKTTEVFSWERGLISQPNYILQVATQ

>Tb11.NT.168_3 [604 - 780]

MPKTGDERLSHEDHRSIQLGAGAHFSTKLYSPGRDSVGDSIRGLIRLLVFLLDVMLAFV

>Tb11.NT.168_4 [823 - 930]

MPQIVLDSRLFVINSLRPPHGTPHRSMNRCLSFGPL

>Tb11.NT.169_1 [151 - 231]

MTYEKGPTMYVFRNEVMWENVHILGAT

>Tb11.NT.169_2 [179 - 478]

MCLGTRLCGRMYTFWGQLDFFFLPSPLCLHLIFVYVERIKLFSYTFSKFLFRIVSIYILDDTVYRPRRCIKFDDRSAGAPKPFSALRIFLTKECSAARAQ

>Tb11.NT.170_1 [20 - 193]

MHLCRGGYVCSCGRLMCPGCSRLCLACNQLVCANCIPLTMTHCLRCSLAYIRNTRCQM

>Tb11.NT.171_1 [43 - 198]

MHLFFYYYFVLGCGSVLSDVRVCATAVSVVGNQGENQGAHAHSSEVHFTSRG

>Tb11.NT.171_2 [98 - 202]

MCACVPQPSPLLATRGKTRGPMRIRQKFISHPAGS

>Tb11.NT.171_3 [78 - 248]

MRECSLGCARVCHSRLRCWQPGGKPGGPCAFVRSSFHIPRVAEGCQEKHGQVPPTQS

>Tb11.NT.171_4 [223 - 309]

MARYPQPNRDYDECVCVHLPVLWHITGDD

>Tb11.NT.171_5 [254 - 367]

MMSVCVFTYRCSGILRETIDVPLTRQVLGGAFETIQQA

>Tb11.NT.172_1 [227 - 307]

MKLFAIFPVLVNVGTEGAGDVGSPAVK

>Tb11.NT.172_2 [214 - 357]

MIGSYEVICYISGAGQRGDGGGRRCWLSRCQMKVMVRWRGTSQANSVV

>Tb11.NT.172_3 [285 - 407]

MLALPLSNEGYGPLAWDKPSQFGSVRAPLNFSTRLMGANEL

>Tb11.NT.172_4 [429 - 515]

MYCWTLARFRLSLLSPLLVVGRSPEFTVL

>Tb11.NT.172_5 [582 - 731]

MLNTTINIEIQGFVVSFAEQHLRMASSAEQWVVHIELCLVRVRASIFFVL

>Tb11.NT.172_6 [568 - 786]

MIKEVCSTLRSTLKFKVSLFPLLSSTCVWLHRRNSGWFTSSYVWCECALAFFLSFSAAETRGIPPDASVKAGR

>Tb11.NT.173_1 [142 - 291]

MLQPLATSDGMRVTPRKHHISVKQPLRNYMEKKYINIYMCARLCTRALRS

>Tb11.NT.173_2 [301 - 432]

MDSNFMCMRAVQGEKVVQRSGVLCCRVSKLIIGVLRFGNFIRKL

>Tb11.NT.173_3 [565 - 687]

MLLSTLSCYMVFYRILFCHISGIEFWVSLIVLFCDLEKYHI

>Tb11.NT.173_4 [844 - 930]

MLAFALDDMGFLVPGSWCCRCLFLLFSWG

>Tb11.NT.174_1 [13 - 189]

MPSCAFLRNPNACLMLKDLVIFILGQRGIAHISSPTVPLQKQKYLFSLSVRDEGDGCKG

>Tb11.NT.174_2 [201 - 410]

MSVPLLRVDLLSVTFTDSAAGHLKGLLDGVVARASRIYFYFYFLFFIFIGNQHKTDGSFTVVIIIFLIVF

>Tb11.NT.174_3 [167 - 415]

MKEMGVKGEGGYERSSFACGPFICNFYGFGGGASERLIGWSCCKGFAHLFLFLFFIFYFYRESTQNRRFIYCCYYYFFDCFLT

>Tb11.NT.174_4 [427 - 582]

MKWNLIAQMGVCEFASLIPIYIYVCIIVISFTVWPPSCLRLSQSQLPLLVCY

>Tb11.NT.174_5 [700 - 834]

MVLVCVHCYCLLLRPEQCERKGDMPFSPFIKRIYFNEYQHPQRNK

>Tb11.NT.174_6 [623 - 838]

MEFGGYITNKHASVYIYIYIYVGMHAWFWCVCIAIVCCYVPSNVKGRAICPFLPLLSVYISMNTNIRNEINK

>Tb11.NT.174_7 [852 - 965]

MTPAHTHKRNQNLCTFIFSSSLFPSCLLGYHWRVCKVL

>Tb11.NT.174_8 [1219 - 1293]

MLHIVTHICVFTVSLAFALECLSLM

>Tb11.NT.174_9 [1100 - 1357]

MMFNLLLPTACITLRRRRRSCPLRVHNYFLFHFPPSVTHICCILLHIYVYLLSVLHLRLNVCHLCDFCTSAPFLRMCWCQNTQTQL

>Tb11.NT.174_10 [1278 - 1481]

MFVTYVTSVRLLLSCACVGVKTHKHSFDLSLRCVFRAVINYWQSPTRGIFVVMRSCVRSKIPYMMMIQ

>Tb11.NT.174_11 [1605 - 1763]

MLTCMRGGLGGCFGTTPTISVLTNLFSIFLFSSPFLWILLQSAVVMDTRNYQF

>Tb11.NT.174_12 [1613 - 1921]

MYAGRVRRLFWHHANHQCFNQPLFYFFVFFPVSLDPSTKCGCNGHTKLSVLVLSLLLVLFLPYFLCSPDCSCCPLFFFCLRCWNCLLGLCVNNCSFLRLSLFM

>Tb11.NT.174_13 [1825 - 1950]

MLPSFFFLFEVLELPSWALRKQLFIPATLPLYVDADCTFRVS

>Tb11.NT.175_1 [256 - 342]

MRMMFMVFNLIWTGEFVPQSMDQTLRLWH

>Tb11.NT.175_2 [336 - 446]

MALKWRLIIPPLSTMLTTYECVWRIIFSYIFVVDATQ

>Tb11.NT.175_3 [446 - 580]

MMPCVGPRTGLRRKVNTREGAFVIAVFFCLNGWAAPHAVFWDSLV

>Tb11.NT.175_4 [436 - 669]

MRLNDALRGAPHWFEEKSKHKRRCICDSSFFLFKRLGGSTRCLLGFISMTHNKEMQNVNIYVSLLRRRRQRESLTEIS

>Tb11.NT.177_1 [384 - 470]

MNPLKGPRHLLSVTASVLEVSATACPPTP

>Tb11.NT.177_2 [311 - 700]

MSISQVDLEHSPHPAFQVATFSATDESAEGAATSPVGDGECFGGVRNGVSTNSIKSAAELLSVHQKTLIDISLTFPARAPTLDLLDMHGITLSRRTLRADLPDPDGRDAAFSKLEEFRFGDDHSDDPSNL

>Tb11.NT.177_3 [624 - 731]

MEGMRHSVNLRSFGLEMIIQMIRQTFDMFLSLFFFI

>Tb11.NT.177_4 [802 - 909]

MHCVRIRLTDDTFFGIPFPVCIFCLHFSYIFSYIYE

>Tb11.NT.177_5 [1537 - 1626]

MLQIKYIPIKGDANTRVPYVVGKGEKEVNK

>Tb11.NT.177_6 [1732 - 1824]

MEGDAPFPCCLFFFSPSFYFISFYFIFFTCG

>Tb11.NT.177_7 [1857 - 1943]

MLLFCRVRVCTYANKYKYTIHKYMCGTVC

>Tb11.NT.177_8 [1891 - 2013]

MQISINIQYINICVARCVRVRERGGETKKRGKKEDNKYYKL

>Tb11.NT.177_9 [2784 - 2870]

MVTGKEKKGKGNAAYFFLFFLFFPFLVSL

>Tb11.NT.177_10 [2910 - 3218]

MVMLPHKTGNERSTIKKEKKRRKMVNIHVSCISCLFFFFPLEHRNSHSVEQTKLFFSSFFSFFLFKKFFSLLFNLFNVFIYCCFHLLLRWYQLAHTHRSVSYR

>Tb11.NT.177_11 [3176 - 3256]

MVSVSTHTQKCQLQVVGDELKEREKKN

>Tb11.NT.177_12 [3272 - 3382]

MGKFTAVEVGEQKKATIKSLKEKQNKTEITTTTTTKY

>Tb11.NT.177_13 [3637 - 3729]

MFLHLFVFFIFVGAKQKGNSFVILFFLSTQR

>Tb11.NT.177_14 [3984 - 4091]

MADKSIGLLMLPSIAFDEQHTNEYLLPHLLFFFLFF

>Tb11.NT.180_1 [34 - 114]

MWIGRKDARGLKSLSVWFFYHLEATYW

>Tb11.NT.180_2 [380 - 463]

MYCSVASGVLPFILNTSNVFRGIFLNAI

>Tb11.NT.180_3 [562 - 639]

MFLSHMIPRCALVVLSRFSCCSISSA

>Tb11.NT.180_4 [456 - 686]

MLFDFPSLRECLRRYQRRKYIEYFGCCHCLWHSADNVFEPHDPAVCASSSFPFLLLFYLQCLKILLFPFLLLRYFSY

>Tb11.NT.181_1 [23 - 310]

MQHDNVGDRESKRTSELDDKRRSAYRWSLAILGLATVVTFAFAWRKLYRFNQRELVLQRKLQMDRSLAAALAEVPGRRFTVMDVNPEALRKSALIT

>Tb11.NT.181_2 [377 - 463]

MCHFSLYRSCAYCSKFFLIFFFYSARTKL

>Tb11.NT.182_1 [73 - 213]

MCIYSCKSEFTSRRQNPTARSSSIQNSALYKIVQWEKSSSCTAHAVV

>Tb11.NT.182_2 [41 - 256]

MVLALTSPAEKCASTRVNLSLHHGDKIRLPEAAAYKTVHCTKSCNGRSLQVVPRTRWCNSIQNWSKFRWWAR

>Tb11.NT.182_3 [174 - 326]

MGEVFKLYRARGGVTPFRIGANFGGGHADNFNRLPLFLINGVKILFNEVEW

>Tb11.NT.182_4 [525 - 605]

MYQNNDNNNNNNSRELCAERPLLCVSE

>Tb11.NT.182_5 [642 - 725]

MKLPNEQRTHGRSVSCTDALILGSSGTL

>Tb11.NT.182_6 [885 - 980]

MEFELIKMMLLNVVSKRRPQRRGQKEKNSGKT

>Tb11.NT.182_7 [848 - 991]

MWKFGVLWAFSVNGIRINKNDAFERRFQAEASKARTKRKKQWKNITLD

>Tb11.NT.182_8 [1170 - 1370]

MHFQVRKLTYVELAVFCYYISQLFAVCPTQYMSSQWRIVCPDTLTVDAEGKARKGMAVLCEFMQHQH

>Tb11.NT.182_9 [1387 - 1659]

MALLSTEVRVWFTKKNIGSSFVERHFKLLDFPLPRDCEGGGNSKENHTAEALEITIILLRLNTCTEHVHKFIFIENSDFGCKGIAALVTVG

>Tb11.NT.182_10 [1586 - 1813]

MCISLFSLRTATLGARGSLRWLPLANPLRLFLHIFFNEFFYVFSTCFCSYCFRCVSEIQKECRRRYEEKIISKKTS

>Tb11.NT.182_11 [1695 - 1817]

MNSFTFSALAFALIVFAAFPKSKRSAADATKKKLYQKRHLE

>Tb11.NT.182_12 [1820 - 1918]

MSNDNGGTIFRHIYERTSRLHLFSFRYFPPRPA

>Tb11.NT.182_13 [1827 - 2039]

MIMGVQFSGIFMRELPVCTYSRFVTFHRGRHSTEGNLSSCLHFLSSIFHGNNCFVKLSVQIVRWRHRTMST

>Tb11.NT.182_14 [2437 - 2724]

MKYRKVLKETITRGPNSLSFEMQQEDNYLCVVCVKVEWERVMREELWMLSKRSRDVKLRGMVGIEMGSSVNKVMVAFCLVRISDGNDIFCNVVEYE

>Tb11.NT.182_15 [2820 - 2924]

MLVSWRKGNDFSDAKADIFRRRRTEIDGVLYRMNE

>Tb11.NT.182_16 [2920 - 3090]

MSNKIFIFTTGMQYLCVFIQILIYYTHREVENISLQIVGCSTGTHLLESQSGLGSPV

>Tb11.NT.182_17 [3326 - 3538]

MFLAAVRGVISRLMSVGPQHRRQNLFLRCFPMRQSSANKTGNPLPPNLAEPHAVIKSACKLQQPLCTIFFF

>Tb11.NT.183_1 [93 - 245]

MAGATLELSIVGYIMGMVLVCAGILFPLAVMGLRSRSVFRRRGLRPQSMIQ

>Tb11.NT.183_2 [255 - 365]

MHLFFRDTETIVRLMSVGIGSAAIWLPATREGVTHHV

>Tb11.NT.183_3 [326 - 424]

MAARNTRRCYSPCLNFPYFSIWCVVRTTSIYIY

>Tb11.NT.184_1 [115 - 192]

MPLIFAVYYYTIKLVSQFELATGSRH

>Tb11.NT.186_1 [159 - 605]

MRGNRLARRGDPMKTRSERRRERKKKISSSSWAQRARKRLCRVIAKRCAREEDSHMTAIMQSYAIRRKAQRDAERAAEINTEEVSQSTDQDHQESVEGKAPRSRGGDVREGLSVSAAKSHRRVDARRYPGREAKALRERQSKARTQSLY

>Tb11.NT.188_1 [312 - 488]

MCSVLWADSATKLDMEGNHLRPPVSNLVPLSVLPCGRHFLNLLTMHIVQLLLILWSFQM

>Tb11.NT.188_2 [685 - 825]

MEFGGCWTICLTSRKGLVVWGATATVPFVIPTPHFYWRRIQAGVSSL

>Tb11.NT.188_3 [672 - 941]

MPSTYGVWWVLDNLSYQQKRSCSMGCYRYCSFCNPHTSFLLEKNSSGRLFIMTFSIAPQGNEQELCYHCTPNDWHRSLQIATIAGSVDAW

>Tb11.NT.188_4 [853 - 993]

MSRNYVTIVPLTTGIAVCRLQRLPVPSMPGNGESLTGMHICTHPFWC

>Tb11.NT.189_1 [89 - 262]

MSMRMTPGKIYAGSAIIITAMTLWNRYSTQNLRTIVDDAKERQLKEAALIREASNNNI

>Tb11.NT.189_2 [300 - 410]

MDTSFFFPPRIGVCCGRAAKVCCCRSAVDNAFMFQSF

>Tb11.NT.189_3 [388 - 531]

MLLCFNPFNYTIGGVLQDFLLKCLAVFYFTEFPPAVSFVISIGCELLP

>Tb11.NT.190_1 [254 - 346]

MDTGDKEGRYNVSYVCRMLSRLPRPSPQASG

>Tb11.NT.190_2 [346 - 423]

MKKFTSLTHLHTRSSIMVRAGDSLTE

>Tb11.NT.190_3 [285 - 509]

MCHTFVVCCHGFRDPRPRPADEEIYFSHAFAHPIFYYGSSRGQLDRIVTYLSSTCSFYCYRVKGGDYIVQSFLES

>Tb11.NT.190_4 [673 - 786]

MREMMLLPNTVVSVSAPQVTLPGAHLYLYLFHYHTHFQ

>Tb11.NT.190_5 [1089 - 1376]

MWPALPLCKLCSNVRETLTMLPVFSVLQVATPGVCLRPRTHQPSFCFSMEVIYATMKQITAYFLLPVLLLLLVRVFTQSLFFFVCWFGFYGDSTSS

>Tb11.NT.191_1 [101 - 202]

MRMSRQLSMHRVREKMSHICVKTSSQHSFQPCTN

>Tb11.NT.191_2 [22 - 411]

MEVSADTQALSEAMMIRLIEEQIWCGYEDVETAVNASCEGENVTYLRENVFPTLVPALYELVKRQQQCALEPDEVSHRYGPTGNTHPISWLSQYLLRNNTRHSKKICGHPYVMVNNELLRKEKGTRTKES

>Tb11.NT.191_3 [353 - 451]

MLWSTTSCLGRKKEQERKNRDICILCRFFHFFG

>Tb11.NT.191_4 [418 - 510]

MYFMSFLPLLWLRRYSLILCSLFFSWVTGLY

>Tb11.NT.192_1 [230 - 412]

MSIYFFLPVVIFLPFKRENAKYFSMLLPSNHISRTVTYVSPLLTTVSPHPRIFTCMWIFFR

>Tb11.NT.193_1 [106 - 210]

MLDTLSHRCRNHWRCGDCFTHSENVPSPYPSWGLQ

>Tb11.NT.193_2 [546 - 716]

MRILNGLLNHERGMITFIHSGLCLPSIQWSCASFFFSFFVSTRQSLPSSLIQIVPLT

>Tb11.NT.193_3 [719 - 823]

MWVLSGNAFLSLSLMHRNKCYVRVWLCCDETFEIT

>Tb11.NT.193_4 [738 - 836]

MLFCHFRSCTATNVTCVCGCVVMKPLKSLESTV

>Tb11.NT.193_5 [988 - 1065]

MEKIPSFGRILTWIPPYSPAACRPYV

>Tb11.NT.194_1 [79 - 162]

MRLRYVSCLSKDSHHLCSFLICCTVGLG

>Tb11.NT.194_2 [312 - 410]

MSATGGACVPFSLHSCNELLNTWEKLGWWTAPH

>Tb11.NT.196_1 [25 - 339]

MFCSTPLHINGSVNCPLQGDDKVVRKDDNPLFVCLGDASFNDFEHSDDAPIPYILPDVAGWCSTSGQGEGLLALEKALEVIVGRLVPPADVVFCSEATELPEPPG

>Tb11.NT.196_2 [104 - 526]

MITRYLYVSVMPPLMILNIRMMLLYLTFYRTWQVGVQLLGRARACLRLKRHWKLLWGAWCHRQTLCFVAKLQNSPNHLDKVCWLVPMGIDGCFVEALFFPFSVLVCRLGCRFRLKALLSFWECRRVYENISYRCLEQILCV

>Tb11.NT.196_3 [530 - 628]

MWFPVHFHPHYFVLPHRKQVRCVSCLHQDVPAI

>Tb11.NT.196_4 [372 - 629]

MVASLRRCFSPFQFWFAALDAAFGLRHYFHFGNAAGFMKIFRTGAWNKFFVYRCGFPYIFTPIILCFHIVNKCDVFPVCIRMYLLF

>Tb11.NT.198_1 [483 - 578]

MVRCLERLFPSSSPLLLDFLRLGLWVGKPLGV

>Tb11.NT.198_2 [587 - 736]

MVETLHYVLYSVFYFIPSFCPLNPFATIPFPASNTNFPFVCTHRTWCLVV

>Tb11.NT.198_3 [752 - 871]

MKCGTCEEPCESGFIYPLTFVCGETDDQMLQFDSCFLECG

>Tb11.NT.199_1 [170 - 277]

MLLINYPGRSRTVSQVEAFGGKKNHQYCLGGTARKT

>Tb11.NT.199_2 [305 - 394]

MHSPLACWLTLPIPMEVSNTFETLNSKTVE

>Tb11.NT.199_3 [301 - 549]

MNAQPSGVLVNFTHTNGGFQYIRNPQQQNGGVSTTKASLFYFPLRCVFDFSDGSVVPFNRGVCLSAHPPGPSAISPACAPWRW

>Tb11.NT.199_4 [540 - 647]

MEVVSPSVKEGRSIVKRGEFFFLRENRIFVINLQSF

>Tb11.NT.200_1 [61 - 183]

MLSCSFIAPFLSVCVPLFGVPHLYGQGEGCCGIGFARLTAA

>Tb11.NT.200_2 [131 - 373]

MARVKAAVALALLDLLRLDSLAFGLPGGVMQRNGGGGRTSNTLEKFLSIDPYAYMCVDSRGAGSFFFFRNYRRGCSSCVWL

>Tb11.NT.202_1 [3 - 131]

MEAQRTLMPRDLLVLCFCTKLGQGESWNLLLVVYKYSIFWHPC

>Tb11.NT.202_2 [237 - 383]

MPSDLFCTTHWGTRKVTRLAVKKQSLCVCLSKPRGNRFPFLRFRKLCVG

>Tb11.NT.202_3 [134 - 418]

MPGTISGFDFCERLTAYLHRWALQFREALWIPCGNAFRLVLHHSLGHEEGDQTCGQEAEPVRLLIETTWQQVSLPPLQEALCGVSNFRNVIESMC

>Tb11.NT.202_4 [399 - 521]

MSLSLCVRRCCAWTVGIVPPHTAHMFVKARDVDAVPFLRGA

>Tb11.NT.202_5 [496 - 576]

MLYPSYEVLSARSRDTKTVVPSPSSVG

>Tb11.NT.203_1 [106 - 231]

MLSVTGTSGLYLISLIVYVLHMLCGDQGNNICMKRMSAVICR

>Tb11.NT.203_2 [348 - 482]

MCLSAVARGVSDGTSFAEYGCVSSTRHRIECCSPLVPSRRLVGFE

>Tb11.NT.203_3 [382 - 507]

MALRLPNMVVFLQRGTELNVAPPLYPLGGWLVLSDQGNTKGC

>Tb11.NT.203_4 [570 - 734]

MFTQHRCSLLFVISPFLFVLSAESPNKSTDRRLLLVNERFRIIVIISRFDNYPTA

>Tb11.NT.203_5 [679 - 753]

MKGSVLLLLFLALITIQQRKHEEVG

>Tb11.NT.203_6 [805 - 903]

MRFENPRSVSNRWYRVLSLTFVSMVFDNILGAT

>Tb11.NT.203_7 [948 - 1139]

MPLAYIRIYVPGYSQYVIHPLFYVRVCVCGGGGVGLHKPDIAFRFYTETRCGVVVGVVAYKILS

>Tb11.NT.203_8 [1314 - 1466]

MFVVLGTTSTRDTFFFAHESTLWWWWWQRDLPLEPCDRGRGGGEGSAFVLF

>Tb11.NT.203_9 [1487 - 1591]

MLGVCAYVCTFPMFLSSVESLKGGGDGVSSVVRCC

>Tb11.NT.203_10 [1818 - 1931]

MVGSTSFSFVIYLCGREDIAHRMEIRDRMMVGCDFCAA

>Tb11.NT.204_1 [28 - 132]

MWPTFTYSYFRSRRVSFCAHASLGVAKCGKKRHIN

>Tb11.NT.204_2 [182 - 319]

MLRPNGGKEIKKKENREEKRYERAKKNLSISQPSCCTPGIIPILLQ

>Tb11.NT.204_3 [352 - 435]

MRYIQLKHWRKATHAFFFLGGGGASKCT

>Tb11.NT.204_4 [243 - 464]

MSGQKKTFPYHSQVVAHPVLSPFCFSSNSNHYGPTADAIYTIKTLEKSDTCFLFFGGGRGLEMHINISSSMRTK

>Tb11.NT.204_5 [653 - 745]

MGSEMPEDYHTTFVQGGCVTVDGTVKVTGKK

>Tb11.NT.204_6 [881 - 955]

MKLVSRGIRIRINSFRFSPLRGGPE

>Tb11.NT.204_7 [955 - 1143]

MKTRAASLLLESGKRLPHNIPGSNREHYYEGYREAKSQHPEKVQLTTVVEGIVPVRFLCNHFQ

>Tb11.NT.204_8 [1040 - 1264]

MKVTVRQNRSTPRRYNSPRWWRESSLCGFYAIISSNFSRFHGEAHRRVEPRMWMIQPLESTAEVGKGWTKLSNTS

>Tb11.NT.204_9 [1488 - 1568]

MIILTHLYSFEGVRKHRKGNDDSGRHG

>Tb11.NT.204_10 [1821 - 1976]

MATLARVTPLISSSTGSEQRWNESKEGERHHESALLKVVHESFAKFNQPAGR

>Tb11.NT.204_11 [2091 - 2189]

MPFTPEQKVKGCGVAPWPKVVLAESVLSVAKYF

>Tb11.NT.204_12 [2541 - 2705]

MRAPKNTLTRPNSGKYVKGCRQLGPTSNRYTPAADIHPSLGLTGLFVTWEEERRE

>Tb11.NT.204_13 [2123 - 2836]

MWGGTMAQGSVGRKRLKCSQVFLRQVGPTWRICKQGKIYIYIYIWRSDAPPQKKNKTFSDEVYSTYSTKYFYGFITWAFPASITPEMGEHRNFAELVLSSGRGYFSLALLLAHIRTRVSAKAPNPYLIIKHVNLFFATPDASTEEHADQTEQWEIRERLPPTRTHKQQIYARCRHPSIPWFNRPFRYMGRRAEGIIRPVPPHCSSVSCRTCDEHRKQRPPQLSASHTSGYLGVRPFIS

>Tb11.NT.204_14 [2757 - 2861]

MSTESRDPHNYPRLIPPATWVFARLFLSLTHRQRY

>Tb11.NT.205_1 [156 - 488]

MVPEGRVVVLVGKALEVRLLAPRTISRRDQMRGCNTVTAAARVHGNVTSAPILMYACACPSVGNYMMAVIGTAGFASLILLSVPQKKYGSRSFTFTHSFYPFLLFIFVPLF

>Tb11.NT.205_2 [418 - 624]

MDLVLLPSPILFTLFSCLFSCLFFETMLTIAEASTQTSVPIGTLTCVSDAFWSQKESILDVYGAIFFLM

>Tb11.NT.205_3 [552 - 632]

MRFRCFLVAKRKHLRCLWSNFFSYVGK

>Tb11.NT.206_1 [169 - 291]

MEYGRTVFTFGVGYHQFCVCVCVSLLLRGCVSSACIEVFFL

>Tb11.NT.206_2 [302 - 478]

MSNIKGNNFLLQAPSLKDRKLRRASGIKNRHGSHEWKSAWVLPPLSMNVALLLLFCSLS

>Tb11.NT.207_1 [111 - 293]

MQAYIYKFKYVFVHVYPCRSMLVSTGEKGSGPKSGVHLAGYVLLLFPCRTPSHFNTSKRIN

>Tb11.NT.207_2 [399 - 485]

MCAYVCVYVYVCGAQYFFSFTLGVDPHRH

>Tb11.NT.207_3 [139 - 486]

MSLCMCIRAEACSFPQVKRDLVLNPGCIWRGMFYCSSHAVHLLILTHRNVSIELLCSVCGELMQQKYEDGKRKEGRKEGSACAATGECVRTYACMCMYVVPSIFFLLLLGWTRIGM

>Tb11.NT.207_4 [338 - 487]

MKMGKGRKEGRKEARAPLPANVCVRMRVCVCMWCPVFFFFYSWGGPASAW

>Tb11.NT.209_1 [30 - 122]

MWSSNAECLLSIINCRGRYPSEPRSSSAPHC

>Tb11.NT.209_2 [50 - 133]

MFAVNNQLSRKVPFRAPILIGASLLTPS

>Tb11.NT.209_3 [307 - 525]

MFLRGEGAHVYVVTCSYSLVLQALQGEGTQHRYFYCRSFINGCPNFEYESTSTVSGALPPPLPAPFFFFDKVV

>Tb11.NT.209_4 [547 - 639]

MFRDRHEFVTKFHRRTRQAPKFGQTLRTVRQ

>Tb11.NT.209_5 [449 - 655]

MSPPQRFREHFPPPFLHHFFFLIRLCEKTDSGRCFGTGMNSLQSFTDEHDRPQNLGKLFVLCVSNMHKL

>Tb11.NT.209_6 [684 - 794]

MSVSRNSELLEKTCHAAVLVFTYAVVLLTTEHRISAS

>Tb11.NT.209_7 [751 - 882]

MLWFCLPLSTVSVQVSTSVCREVSICSRGRALDSIDSVECQCTS

>Tb11.NT.209_8 [867 - 1001]

MPMYKLTDIRSPMSLRQLCMPKLLYFLVMISVNVGLPSSVQLHRP

>Tb11.NT.211_1 [196 - 396]

MPALTIACSGATYYGSSVLDIMRHRCKETQPRNEAVESSEEEVGDEVEEVVEDNAGNDENAAEADEE

>Tb11.NT.211_2 [293 - 508]

MRLLRAVRRKWGMKLKKLLRTTPAMMRMRRKPTRSKYIVECFEACRGPWYLLLVIYIFCFFSSHFTRWYFLS

>Tb11.NT.212_1 [177 - 461]

MFLPTHLCRRNPLIRFTYARLKMGGSGTSPSSSTSGSNSSGIRAATSGSTSSILDSINDLPAHFRPRAFSDEEMEVVMMGGATPYVPKSLARKAK

>Tb11.NT.212_2 [430 - 576]

MFPSLLRGRRSKSLDWGIVLTFYLTVCIFFLFFFSFLSIFYLTFSVLFF

>Tb11.NT.212_3 [763 - 1104]

MFFFVYVCVRYEVLYERKGGGGGSQIRRTEKERSDGCTHRSFHNRKKKNVKKKKRWRQRENQFFEMTGAHDIEGSQSASYLSRDSAFPLILSSYLSPLKKKFYLFCYYCHNCFE

>Tb11.NT.212_4 [1141 - 1344]

MLIDIIVIIIVVFIIISFFTACIVSVRIHGGLLPSHYAGKCDGAKMISGCSFPDYLPSFFCLFLLIPM

>Tb11.NT.212_5 [1347 - 1466]

MKYLIIYICANAYLLVCAYEGKGGGGMARRGGGGGKCCGC

>Tb11.NT.212_6 [1378 - 1527]

MHICLYVLMKGKEEEEWRGEAGGGGSVVGADGRVAVSDNEPAVIKRGKIL

>Tb11.NT.213_1 [134 - 274]

MDEMCICASRFTRCCVTKLYHGITRMCICVRLHFGVHGVLRKGLVLC

>Tb11.NT.213_2 [217 - 333]

MRTFAFWGAWCSSKGIGFMLSWHFLFFIFVRFDGSPAVG

>Tb11.NT.213_3 [333 - 413]

MITRTTFFETCPAHSRDQIVEVCHGLL

>Tb11.NT.213_4 [575 - 703]

MESISGLKGVCAVHHRLHGHPCASLSSFFLSGNLKFECRCGHP

>Tb11.NT.213_5 [757 - 1005]

MYLGTDTTRKHDVSPPPPAVLELFPAFFLFFFIAKREVVLPPSSLTGNRANAVLNAGRAEKRERRHYSLLLCMSVRILMFVAL

>Tb11.NT.215_1 [254 - 394]

MVLFSSCHVKMKGACNATRNLRCCIGDDFNSDMPRLRFNRYVSFVAF

>Tb11.NT.215_2 [333 - 473]

MISIPICLGYDLTDTFLLSLFRNGVMCCTSCCYFSYGHSYFPPFFSL

>Tb11.NT.217_1 [33 - 155]

MLHCLSCIELPLKQMMMMMMAMPFSVEQWRRWRKETTVEVM

>Tb11.NT.217_2 [17 - 205]

MYTQIYASLFKLYRIAFKTDDDDDDGNAIFRRTVEEMEEGNNGRSNVNATRQLQCGDCFNNHE

>Tb11.NT.217_3 [159 - 233]

MLRGSCSAVTVLTTTSRRGEEEQTV

>Tb11.NT.217_4 [432 - 518]

MKVTELLYIPFHSQIIEGSRSIFQVFPRF

>Tb11.NT.218_1 [263 - 352]

MYVCMYACMHVCVCVRVCVLPWPGDTRFSQ

>Tb11.NT.218_2 [267 - 422]

MYACMHVCMYVCVYVCVFCRGQETQDSLNSVYFPSFCSAYRTVPTLYHDVLT

>Tb11.NT.218_3 [271 - 654]

MHVCMYACMCVCTCVCSAVARRHKILSIAFTFPVFVRLIGRYLRCIMMCLRDMRASLWWSCAFEGGGVRNCVLHVRRCCSSSSSSPSPHAHTPTHRLWLDYCESVLLHVFFIFANISAIAAASSPFFF

>Tb11.NT.218_4 [593 - 760]

MFFSFLLISLRLPLLHHPFFFEGGGGVPHPRPTAASFCFLSSHSSAYVHGNYYSFF

>Tb11.NT.219_1 [218 - 433]

MAVCDPLVCLRGIFVENRALTARRWSNIQFRWARALIKVGVHYQGQQGVHRKVLHNVPHTNWGNFLGVIRPF

>Tb11.NT.219_2 [545 - 670]

MATVGSRISPYRCRRSCTSCCDSCIRVVRVTHAHICAFMQRL

>Tb11.NT.219_3 [747 - 863]

MGGDTPKKTFKLRAVWRTKHGIGGGSQRHFIVVTSSNSH

>Tb11.NT.219_4 [1139 - 1249]

MNDFFCLASFPPTFLTLRALHTGLWGFTVACCVLNFP

>Tb11.NT.220_1 [237 - 311]

MPCQLDYFFRKAEGKGNDTFHAVGP

>Tb11.NT.220_2 [112 - 327]

MQNAHKRELCYEARDSYHRCLDSLPEMPEKKCAEQLNLLSAACPASWIIFFEKQREREMILSMQLGHNNTSE

>Tb11.NT.221_1 [82 - 204]

MLLLRWCCWWLILVIVEDLVGFSATQLVGIGYLLVNRDLRR

>Tb11.NT.221_2 [317 - 424]

MGCFRVLDKWWLCIVDVFERNFKCCADQRYSSCVCL

>Tb11.NT.221_3 [385 - 486]

MLRRSAIQFVCLSLIATCKRSRFQYGGRKGIIFV

>Tb11.NT.221_4 [45 - 611]

MPIIRKVQCALPYVAVAVVLLVAYFSYSGRSSRILRDAVGGHWVSAGKSRFEAINISCHSDYFNQCQLESSAKGSELYGILVKFISSGVREWVASEYLTNGGYVLLTYSNGILNVAQISDTVRVFVFDRHLQKEPVPVWREKGYHICVIFVVIVLFRLIQARNSPVGVKRRTTPLRLASIYRCSANKKG

>Tb11.NT.222_1 [279 - 368]

MVSGKSKIGSWHGYATGKCTKVFGNITVYI

>Tb11.NT.222_2 [112 - 390]

MAVPRWMRRFEIYAVCMIWPISTIVGIVGTYKLFMWSYGGRDCFRYVVIEEPMKGQWFQANPKSALGMDTPLANVPKFLETSPYTYDDNTGSD

>Tb11.NT.222_3 [332 - 598]

MYQSFWKHHRIHMTTTPAATEHLHKYIKWNEYAYEYKSIYLFVCLFVYSCLHVCAVTFSRLFFSIPLLFPPPPTPNLHTHARKKKKQQQ

>Tb11.NT.222_4 [420 - 656]

MNMHMNINLFICLFVCLFTHVCMFVRSLFHACFFPFPFSSPPPPHPTCTHTHEKKKNSNNDNNNEKGKEEMRCSIMRKR

>Tb11.NT.222_5 [478 - 801]

MSACLCGHFFTLVFFHSPSLPPPPHTQPAHTRTKKKKTATMTIIMKKERKKCVVQLCENGDAAVEWLVQKCNTSVCMQQKKIIEEIIKIYSLIIVMTINIVTIVKHTH

>Tb11.NT.223_1 [308 - 427]

MRWVRCILRVTFTSPQQLSAPALGSHPRMASLMAPPNSRE

>Tb11.NT.223_2 [121 - 438]

MRRLSAPVVGGYCSIPHQVAYREAASATWFGGTEATGSWKRSLKGAAVCLCGIACINVCFRGNALGKVHTESDVYIPAAAFRSRTREPPADGFVDGASQFEGVSVK

>Tb11.NT.223_3 [576 - 650]

MVEYIDDTLIYDLHVWSKSYMYSCV
