# Supplementary material for: On the extent and role of the small proteome in the parasitic eukaryote Trypanosoma brucei
Source: BMC Biol. 2014 Feb 19;12:14. doi: 10.1186/1741-7007-12-14 (PMC3942054; doi:10.1186/1741-7007-12-14)
Supplement: Additional file 2: Figure S1 — Length distribution of the predicted 4,699 ORFs. Figure S2. Growth analysis of the five small proteins with faster or slower growth in procyclics following induction of RNAi. Figure S3. Growth analysis of the seven small proteins essential in procyclics following induction of RNAi with and without expression of an RNAi-resistant construct. The data are based on three independent experiments. Figure S4. Northern blot analysis of essential ORFs. Northern blots for six (Tb11.NT.108, Tb10.NT.90, Tb10.NT.86, Tb10.NT.87, Tb11.NT.28 and Tb3.NT.18) of the seven essential transcripts. Marker sizes in kb are indicated on the left. The predicted transcript size is indicated below each panel. Northern blot analysis for Tb11.NT.29 can be found in Kolev NG, Franklin JB, Carmi S, Shi H, Michaeli S, Tschudi C: The transcriptome of the human pathogen Trypanosoma brucei at single-nucleotide resolution. PLoS Pathog 2010, 6:e1001090 [28]. Figure S5. Semi-quantitative RT-PCR analysis of RNAi knockdown. RT-PCR was performed on the transcripts encoding each essential small protein from procyclic cells un-induced (-) or induced (+) for RNAi. Histone 4 (H4) was used as a control. The small protein transcript and H4 are indicated to the right of each panel. Figure S6. Listing of the seven essential proteins in procyclics with a C-terminal GFP or HA tag. All constructs rescued the lethal RNAi phenotype. Figure S7. Western blot analysis of GFP-tagged proteins encoded by an RNAi-resistant construct. Size markers (kDa) are shown at the left. Figure S8. Growth analysis of bloodstream-form cells following RNAi against four small proteins. Growth for un-induced (tet-) and induced (tet+) cells are shown in log scale. The data are based on three independent experiments. Figure S9. Listing of oligonucleotides used in this study. [file 1741-7007-12-14-S2.pdf]

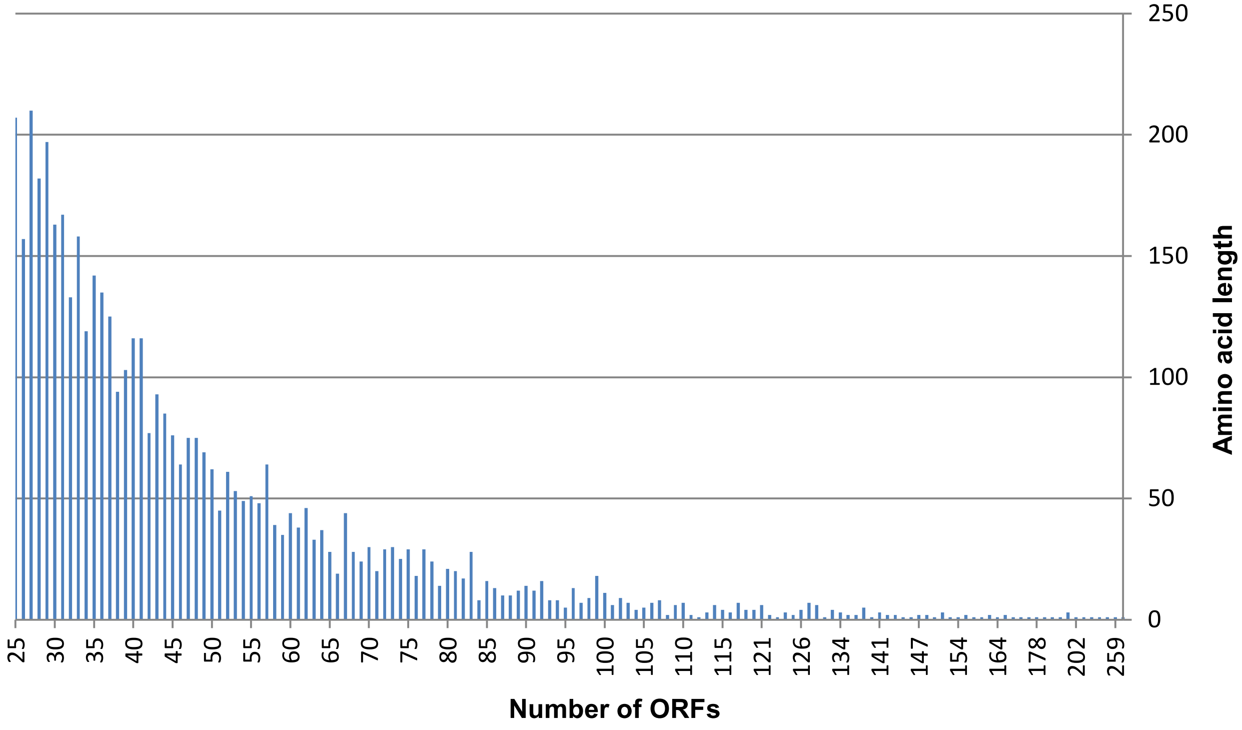

Figure S1. **Length distribution of the predicted 4,699 *T. brucei* ORFs.**

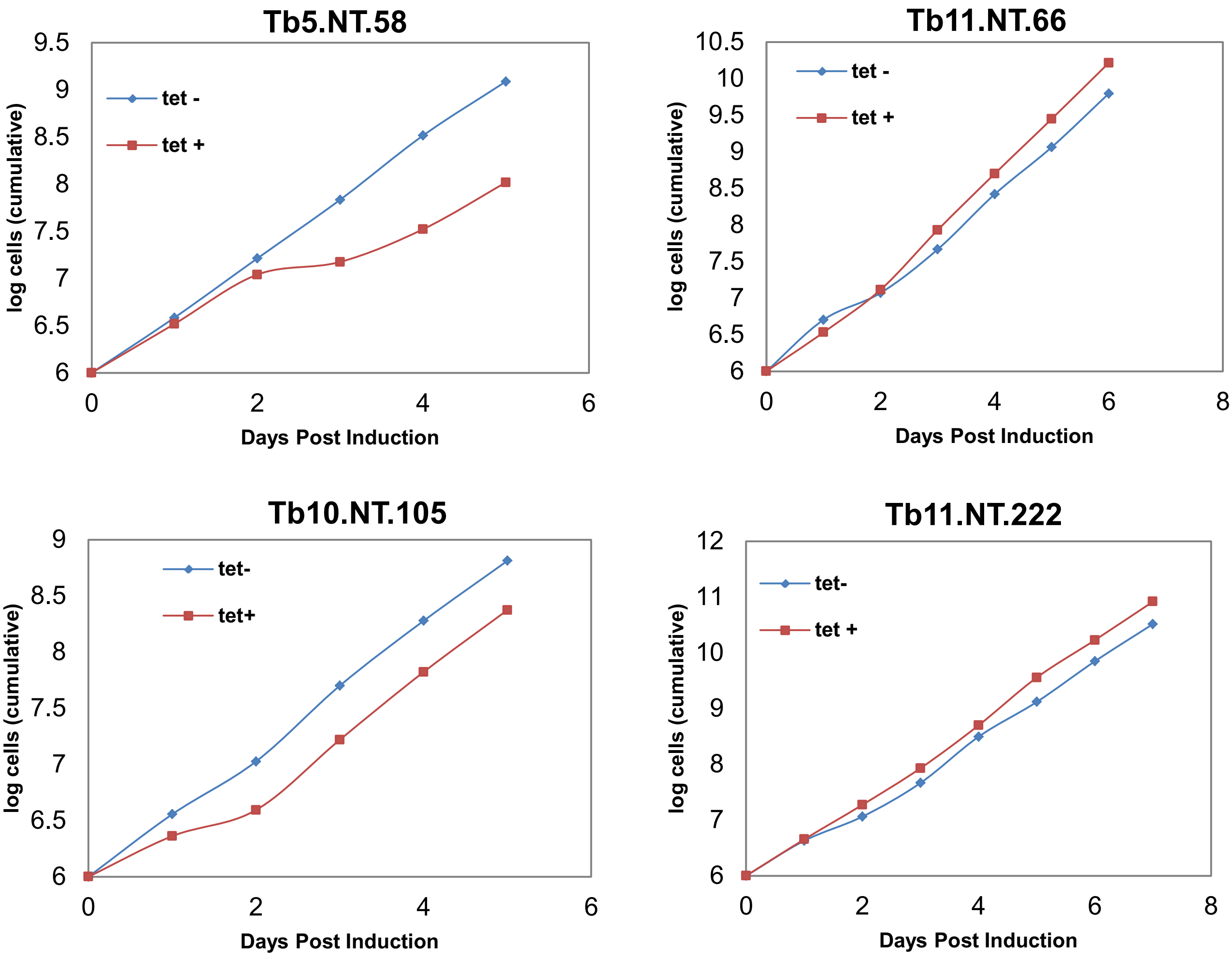

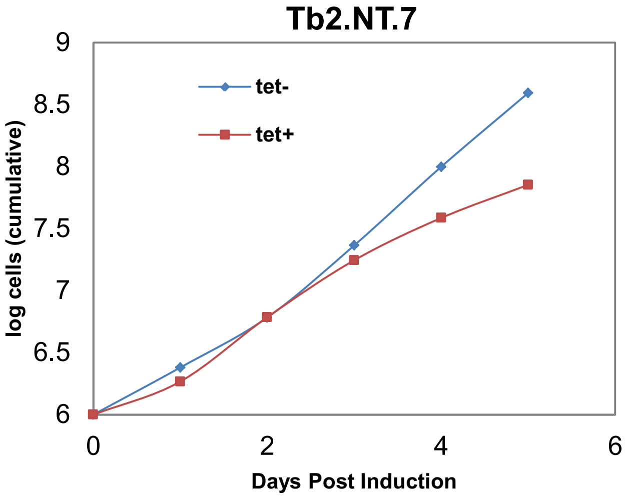

Figure S2. Growth analysis of the five small proteins with faster or slower growth in procyclics following induction of RNAi.

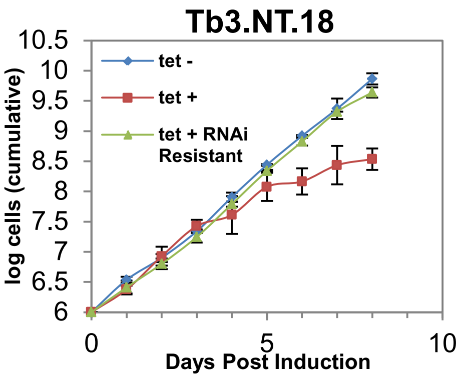

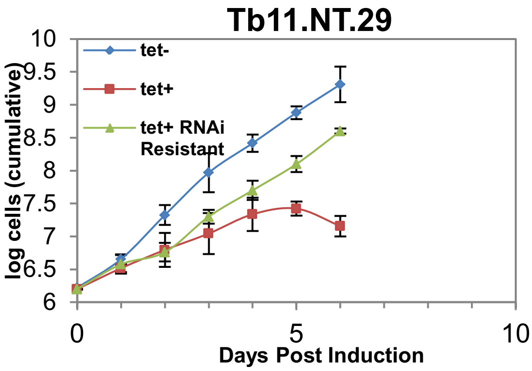

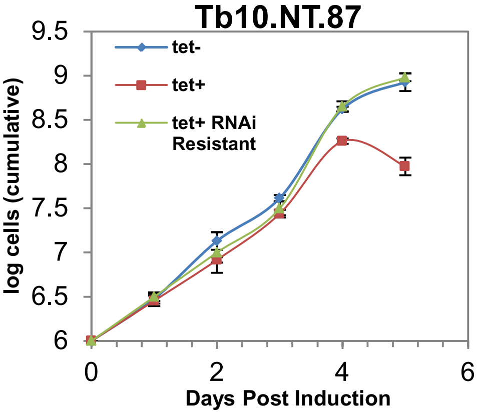

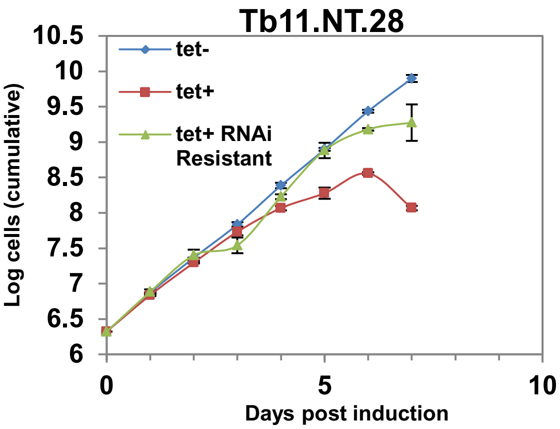

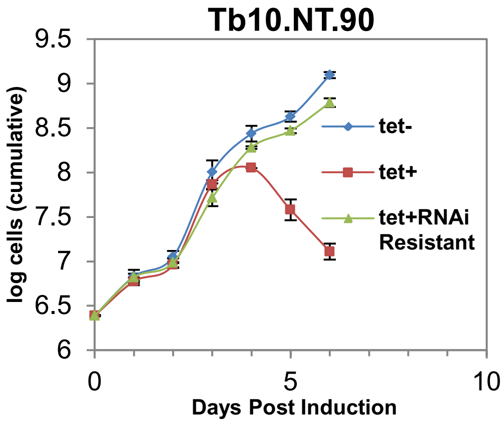

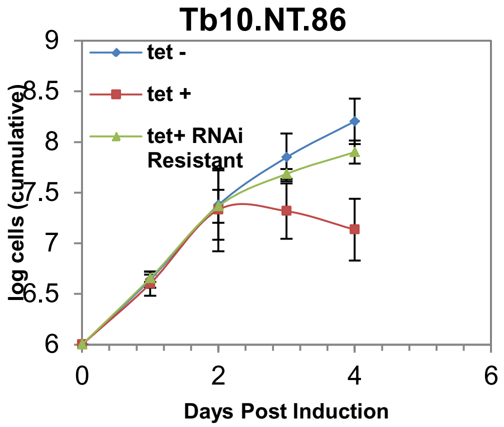

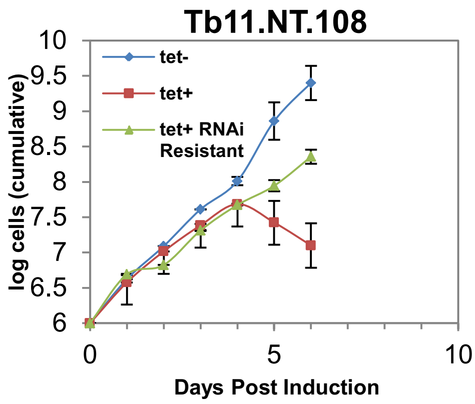

Figure S3. Growth analysis of the seven small proteins essential in procyclics following induction of RNAi with and without expression of an RNAi-resistant construct. The data are based on three independent experiments.

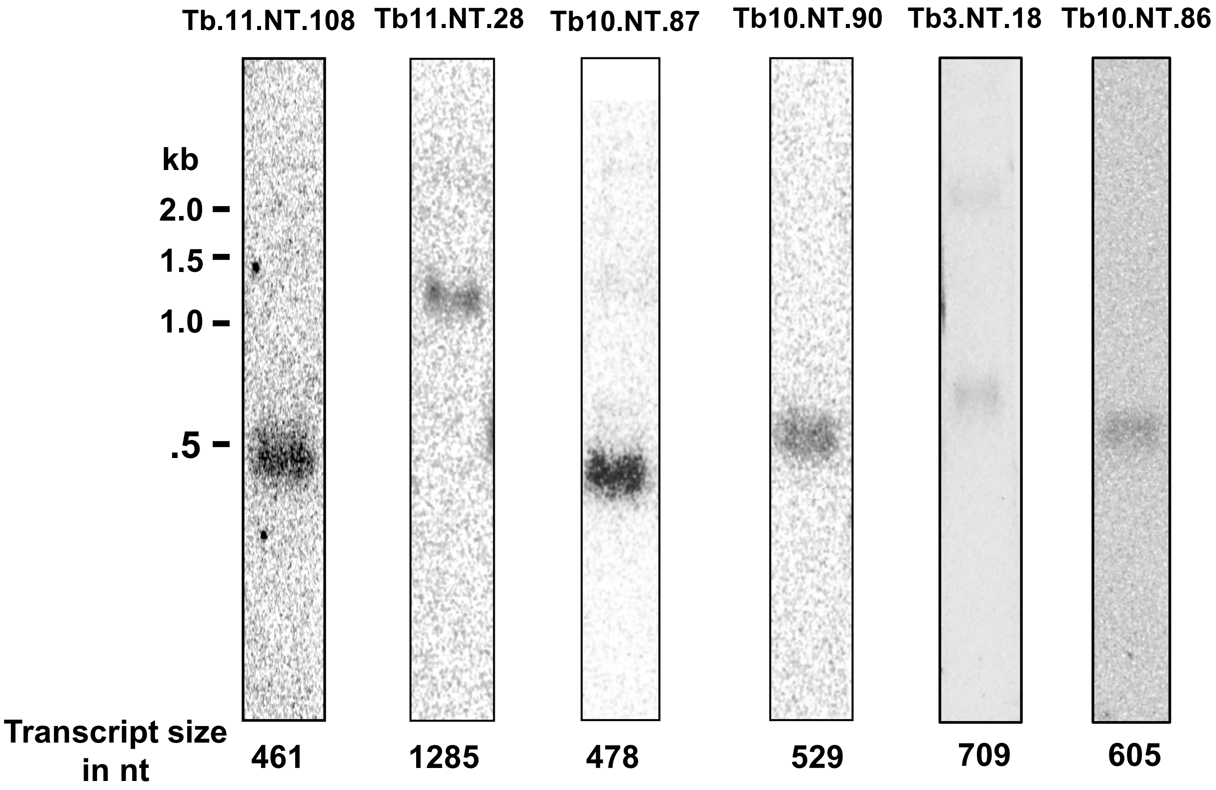

Figure S4. **Northern blot analysis of essential ORFs.** Northern blots for six (*Tb11.NT.108*, *Tb10.NT.90*, *Tb10.NT.86*, *Tb10.NT.87*, *Tb11.NT.28* and *Tb3.NT.18*) of the seven essential transcripts. Marker sizes in kb are indicated on the left. The predicted transcript size is indicated below each panel. Northern blot analysis for *Tb11.NT.29* can be found in Kolev NG, Franklin JB, Carmi S, Shi H, Michaeli S, Tschudi C: The transcriptome of the human pathogen *Trypanosoma brucei* at single-nucleotide resolution. *PLoS Pathog* 2010, **6:**e1001090.

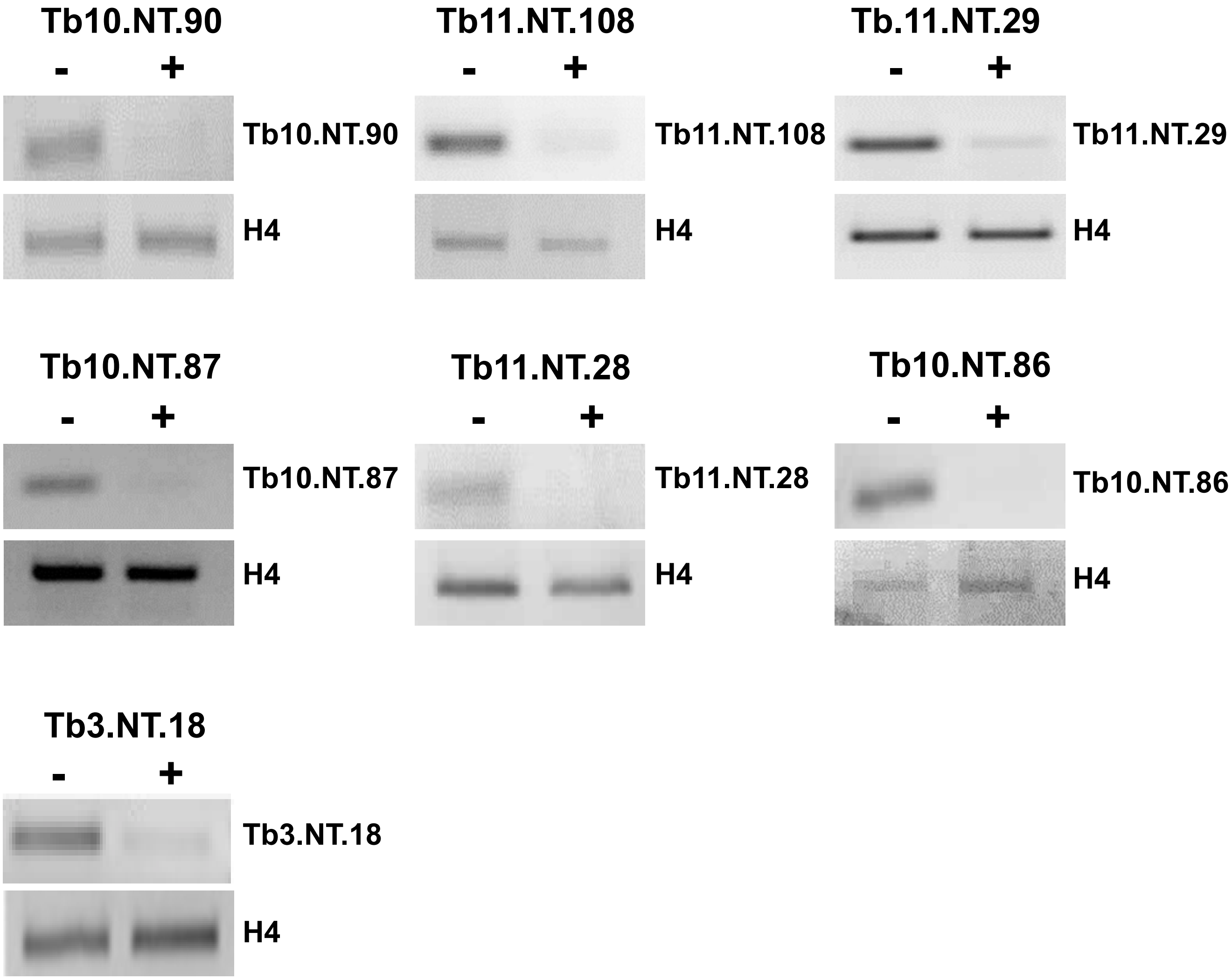

Figure S5. **Semi-quantitative RT-PCR analysis of RNAi knockdown**. RT-PCR was performed on the transcripts encoding each essential small protein from procyclic cells un-induced (-) or induced (+) for RNAi. Histone 4 (H4) was used as a control. The small protein transcript and H4 are indicated to the right of each panel.

Small Protein ID	RNAi Resistant-GFP	Rescue	RNAi Resistant-HA	Rescue
Tb3.NT.18	Tb3.NT.18-GFP	+	Tb3.NT.18-HA	+
Tb10.NT.86	Tb10.NT.86-GFP	+	Tb10.NT.86-HA	+
Tb10.NT.87	Tb10.NT.87-GFP	+	Tb10.NT.87-HA	+
Tb10.NT.90	Tb10.NT.90-GFP	+	Tb10.NT.90-HA	+
Tb11.NT.28	Tb11.NT.28-GFP	+	Tb11.NT.28-HA	+
Tb11.NT.29	Tb11.NT.29-GFP	+	Tb11.NT.29-HA	+
Tb11.NT.108	Tb11.NT.108-GFP	+	Tb11.NT.108-HA	+

Figure S6. **Listing of the seven essential proteins in procyclics with a C-terminal GFP or HA tag.** All constructs rescued the lethal RNAi phenotype.

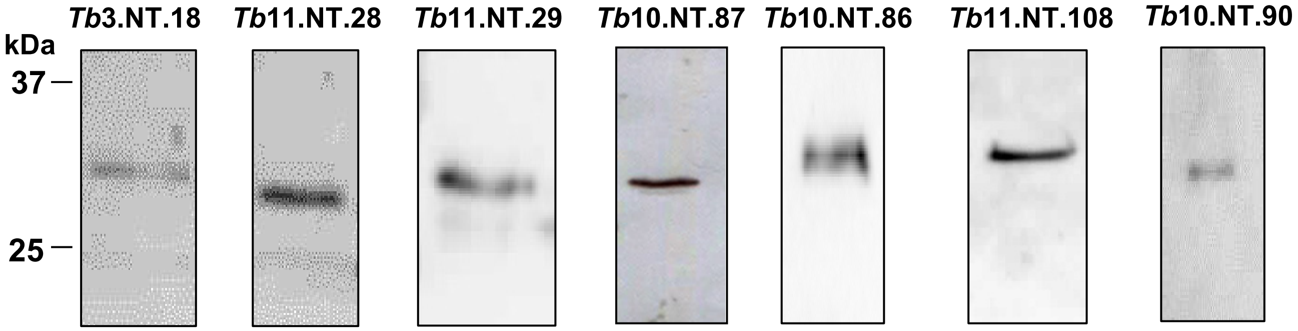

Figure S7. **Western blot analysis of GFP-tagged proteins encoded by an RNAi-resistant construct.** Size markers (kDa) are shown at the left.

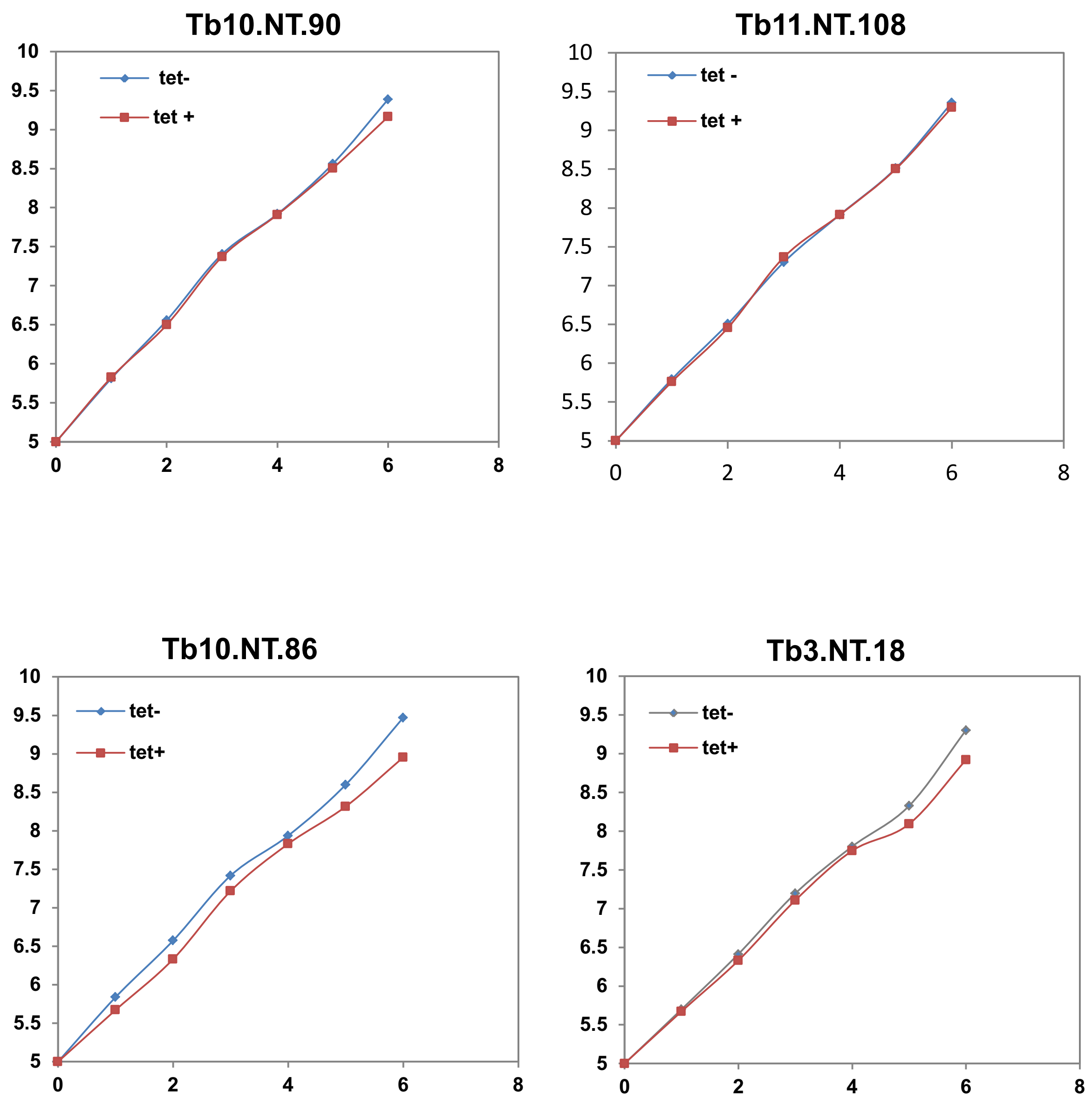

Figure S8. **Growth analysis of bloodstream-from cells following RNAi against four small proteins.** Growth for un-induced (tet-) and induced (tet+) cells are shown in log scale. The data are based on three independent experiments.

Figure S9. **Listing of oligonucleotides used in this study.**

Oligo Name	Sequence (5' to 3')
**Hairpin Oligos**	
Tb3.NT.18 HPF	GGGCAATACACATCTCACAAACCGAGTTAACCG
Tb3.NT.18 HPR	CCACGTGGCTAGGAAATAAAGACAAGGTTGAGC
Tb10.NT.86 HPF	CTGCTTCACGCCGCGGAAAGTAGCAGCAGAGCTGTAG
Tb10.NT.86 HPR	TTTCTGCACAGTCTGTTTGTTAAAATATACTTCTTCG
Tb10.NT.87 HPF	TGATGGTTGGGGCCTTGTTTTGGTAATTAGAAAAATC
Tb10.NT.87 HPR	ATGTCACGGTACGCCTGCTGGTAGTTCCACCTGTTCG
Tb10.NT.90 HPF	GTTGCGAAAGGGCACTGGTCATGTCAG
Tb10.NT.90 HPR	CTATGATAGAGGGTGCCTGATTGATATGGG
Tb11.NT.28 HPF	GTTTATTGCGTCAAGGCGAAAAAG
Tb11.NT.28 HPR	CCGGTGTACTCCAAAACCACCAAAAACATGTGACCGC
Tb11.NT.29 HPF	GCGATTATTTAAGGAGAGCGAATAATACGGTAACAAC
Tb11.NT.29 HPR	ACCAACGTACCGGTGCGGGTCATACACGAGGTCACG
Tb11.NT.108 HPF	GTACAGCACAGGGAAATAGTAAGGAGCG
Tb11.NT.108 HPR	CGTGTTGGCGGTTGTCCTTCCACAATAC
**RT-PCR Oligos**	
Tb3.NT.18 RTF	GAGCGGACCCAAACCTGTTGAAG
Tb3.NT.18 RTR	CCAACGAATCGCGGATATACATACG
Tb10.NT.86 RTF	GGACAACACGCACACACACAGTGGGATGTCTCG
TB10.NT.86RTR	GGGGAATGAAGTGCCTTTCAAAAAAAAAAAAGTGCACGGCGC
Tb10.NT.87 RTF	GGGAGGCTTGTGGTTCGTGGTTGGTGTTTGG
Tb10.NT.87RTR	CACGAAGGGTAGGGAAAACATTCGTGC
Tb10.NT.90 RTF	CGCTGTATTGATCACGCTCTCCCTC
Tb10.NT.90 RTR	CAGTTGTGGATACACATGTGAAATTGG
Tb11.NT.28 RTF	GCGGTCACATGTTTTTGGTGG
Tb11.NT.28 RTR	CTAATAATTCCACCATTTGTCGAGC
Tb11.NT.29 RTF	GGGGTGGAAGGTCAAAAGGGGACGAGGAGGG
Tb11.NT.29 RTR	CCACAACAACGCACAACTAGGGCGAGCACATAGGC
Tb11.NT.108 RTF	CTCTACAAGCATTTTCTGTCTTTTGAC
Tb11.NT.108 RTR	CATTTGGTGAGGTGGAAACGGC
**GFP RNAi-Resistant Oligos**	
Tb3.NT.18 RGF	ATCACAAGCTTATGAGCTCACTTTTGTCTGCAGCTG
Tb3.NT.18 RGR	GCTCTAGAGCGGATATAAAGACAAGATTGAGCAACC
Tb10.NT.86 RGF	ATCACAAGCTTATGGGAACG
Tb10.NT.86 RGR	GCTCTAGATAGACGATTTCCTTTTTG
Tb10.NT.87 RGF	ATCACAAGCTTATGTTCAGCCTTACAC
Tb10.NT.87 RGR	GCTCTAGATGGCATATCGCGATATG
Tb10.NT.90 RGF	ATCACAAGCTTATGTCCGAAGCC
Tb10.NT.90 RGR	GCTCTAGATGATAGGGGGTGC
Tb11.NT.28 RGF	ATCACAAGCTTATGTTCCCCGGGATGCAG
Tb11.NT.28 RGR	GCTCTAGATTATATGCCGTTAAGCAATACAGCGGTCG
Tb11.NT.29 RGF	ATCACAAGCTTATGCTTCTAGGTGG

Tb11.NT.29 RGR	**GCTCTAGAGTGATGATCGTCATGG**
Tb11.NT.108 RGF	**ATCACAAGCTTATGGGAGACTCTGAAGC**
Tb11.NT.108 RGR	**GCTCTAGATGTATGGACGTCAAAGGACAG**
GFP pLew100 Rev	**CGCGGATCCTTAATTTATTTGTATAGTTCATC**
